# Impact of lifestyle on cytochrome P450 monooxygenase repertoire is clearly evident in the bacterial phylum *Firmicutes*

**DOI:** 10.1038/s41598-020-70686-8

**Published:** 2020-08-19

**Authors:** Tiara Padayachee, Nomfundo Nzuza, Wanping Chen, David R. Nelson, Khajamohiddin Syed

**Affiliations:** 1 Department of Biochemistry and Microbiology, Faculty of Science and Agriculture, University of Zululand, 1 Main Road Vulindlela, KwaDlangezwa, 3886 South Africa; 2 Department of Molecular Microbiology and Genetics, University of Göttingen, 37077 Göttingen, Germany; 3 Department of Microbiology, Immunology and Biochemistry, University of Tennessee Health Science Center, Memphis, TN 38163 USA

**Keywords:** Data mining, Oxidoreductases

## Abstract

Cytochrome P450 monooxygenases (CYPs/P450s), heme thiolate proteins, are well known for their role in organisms’ primary and secondary metabolism. Research on eukaryotes such as animals, plants, oomycetes and fungi has shown that P450s profiles in these organisms are affected by their lifestyle. However, the impact of lifestyle on P450 profiling in bacteria is scarcely reported. This study is such an example where the impact of lifestyle seems to profoundly affect the P450 profiles in the bacterial species belonging to the phylum *Firmicutes*. Genome-wide analysis of P450s in 972 *Firmicutes* species belonging to 158 genera revealed that only 229 species belonging to 37 genera have P450s; 38% of *Bacilli* species, followed by 14% of *Clostridia* and 2.7% of other *Firmicutes* species, have P450s. The pathogenic or commensal lifestyle influences P450 content to such an extent that species belonging to the genera *Streptococcus*, *Listeria*, *Staphylococcus*, *Lactobacillus*, *Lactococcus* and *Leuconostoc* do not have P450s, with the exception of a handful of *Staphylococcus* species that have a single P450. Only 18% of P450s are found to be involved in secondary metabolism and 89 P450s that function in the synthesis of specific secondary metabolites are predicted. This study is the first report on comprehensive analysis of P450s in *Firmicutes*.

## Introduction

Among the bacteria that inhabit the human gut, species belonging to the bacterial phylum *Firmicutes* and *Bacteroides* are dominant^1–3^. *Firmicutes* species are gram-positive microorganisms with rod or sphere shapes and reproduce via binary fission. This phylum contains bacteria possessing diverse characteristics that are adapted to diverse ecological niches. Some members are saprophytes that live in soil and aquatic environments, performing mainly decomposition and recycling of organic matter, some members are commensals of humans and some members are pathogens of animals, including humans, and plants^4^. Some members have been subjected to thorough investigation to gain understanding of endospore formation and survival^5^ and the biotechnological potential of these organisms has been explored for the production of dairy products^6^, enzymes^7^ and antibiotics^8^.

The *Firmicutes* phylum is further divided into seven subphyla, namely *Bacilli, Clostridia, Erysipelotrichia, Limnochordia, Negativicutes, Thermolithobacteria, and Tissierellia*^4^. Species belonging to the subphyla *Bacilli* are well known for the production of secondary metabolites valuable to humans, organic compounds that have medicinal properties (Table 1). *Clostridia* consist of species that produce short chain fatty acids in the human gut, such as butyrate, which is essential fuel for colonocytes (enterocytes referred to as colonocytes in the colon)^9^ and also species causing botulism, tetanus, gas gangrene, food poisoning, pseudomembranous colitis, and antibiotic-associated diarrhea in humans^10,11^. Quite a number of studies have explored the relation between the changes in the percentage of *Firmicutes* species in the human gut and human conditions such as obesity, inflammatory bowel disease, systemic lupus erythematosus and psoriasis but results are not conclusive^2,12,13^.

**Table 1:** Information on important secondary metabolites produced by the species belonging to the subphylum *Bacilli*.

Secondary metabolite	Species name	Biological properties	Reference(s)
Macrolactin	*Bacillus subtilis*	Antiviral, anticancer and antimicrobial	^14,15^
Bacillaene	*Bacillus subtilis* 168 and *Bacillus amyloliquefaciens* FZB 42	Antibiotic	^16,17^
Difficidin	*Bacillus amyloliquefaciens* FZB 42	Acts against the rice pathogen	^18,19^
Aurantinins B-D	*Bacillus subtilis* fmb60	Antimicrobial	^20^
Tauramamide	*Brevibacillus laterosporus*	Antibacterial	^21^
Fengycin	*Bacillus subtilis* subsp. inaquosorum	Antibacterial	^22,23^
Surfactin	*Bacillus subtilis* and *Bacillus amyloliquefaciens*	Antiviral, antimicrobial and antifungal	^24,25^
Lichenysin	*Bacillus licheniformis*	Antiviral, antimicrobial and antifungal	^25^
Bacillibactin	*Bacillus* sp. PKU-MA00093 and PKU-MA00092 and *Paenibacillus larvae*	Siderophore	^26,27^
Bacillomycin	*Bacillus* sp. PKU-MA00093 and PKU-MA00092	Antifungal	^26,28^
Basiliskamides	*Bacillus laterosporus*	Antifungal	^29^

Because of the potential biotechnological applications of secondary metabolites produced mostly by *Bacillus* species (Table 1), comprehensive analysis of cytochrome P450 monooxygenases (CYPs/P450s) in the species belonging to the genus *Bacillus* has recently been carried out and their association with secondary metabolism has been unraveled^30^. P450s are heme thiolate proteins that play an important role in organisms’ primary and secondary metabolism. Because of their diverse enzymatic reactions, P450s are found to play key roles such as conferring diversity on secondary metabolites^31,32^. The study revealed the presence of 507 P450s belonging to 13 P450 families and 28 P450 subfamilies in 128 *Bacillus* species and 112 P450s were found to be part of secondary metabolite biosynthetic gene clusters (BGCs)^30^. BGCs are groups of genes clustered together that are responsible for producing secondary metabolites in organisms^33^. However, this study was limited to the genus *Bacillus*. None of the species belonging to other genera or a comprehensive analysis of P450s in *Firmicutes* species has been reported.

Studies on the role of P450s in organism’s adaptations or changes in P450 profiles according to an organism’s life have been conducted on eukaryotic organisms such as fungi^34–41^, oomycetes^42^, plants^43^, and animals^44^. However, this phenomenon is scarcely reported in prokaryotes; currently data are available only for bacterial species belonging to the genera *Streptomyces*^45,46^, *Mycobacterium*^47,48^ and the phylum *Cyanobacteria*^49^, suggesting that more research is needed to unravel P450s’ role in other bacterial species. The presence of species with diverse characteristics that are adapted to diverse ecological niches, such as in *Firmicutes,* will enhance understanding of the role of P450s in bacterial species, especially with respect to their role in those organism’s adaptations. Since *Firmicutes* species have diverse lifestyles, it would be interesting to see if there are any changes in P450 profiles with respect to their lifestyle, as studies have shown that organisms lose a considerable number of P450s owing to their lifestyle. Examples are adapting to utilize simple carbon sources, as observed in *Saccharomyces* species^37^, or readily available abundant carbon sources in the host, as observed in mycobacterial species^47^, or making more copies of specific P450s both at P450 family and subfamily level in their genomes (P450 blooms) owing to adaptation to different ecological niches^34,36,38–42,44^.

In order to address the above fascinating research gaps on the impact of lifestyle on *Firmicutes* species P450s, if any, in this study comprehensive analysis of P450s and their association with secondary metabolism in 972 *Firmicutes* species belonging to 158 genera has been carried out.

## Results and discussion

### Only a few *Firmicutes* species have P450s

**Figure 1:**
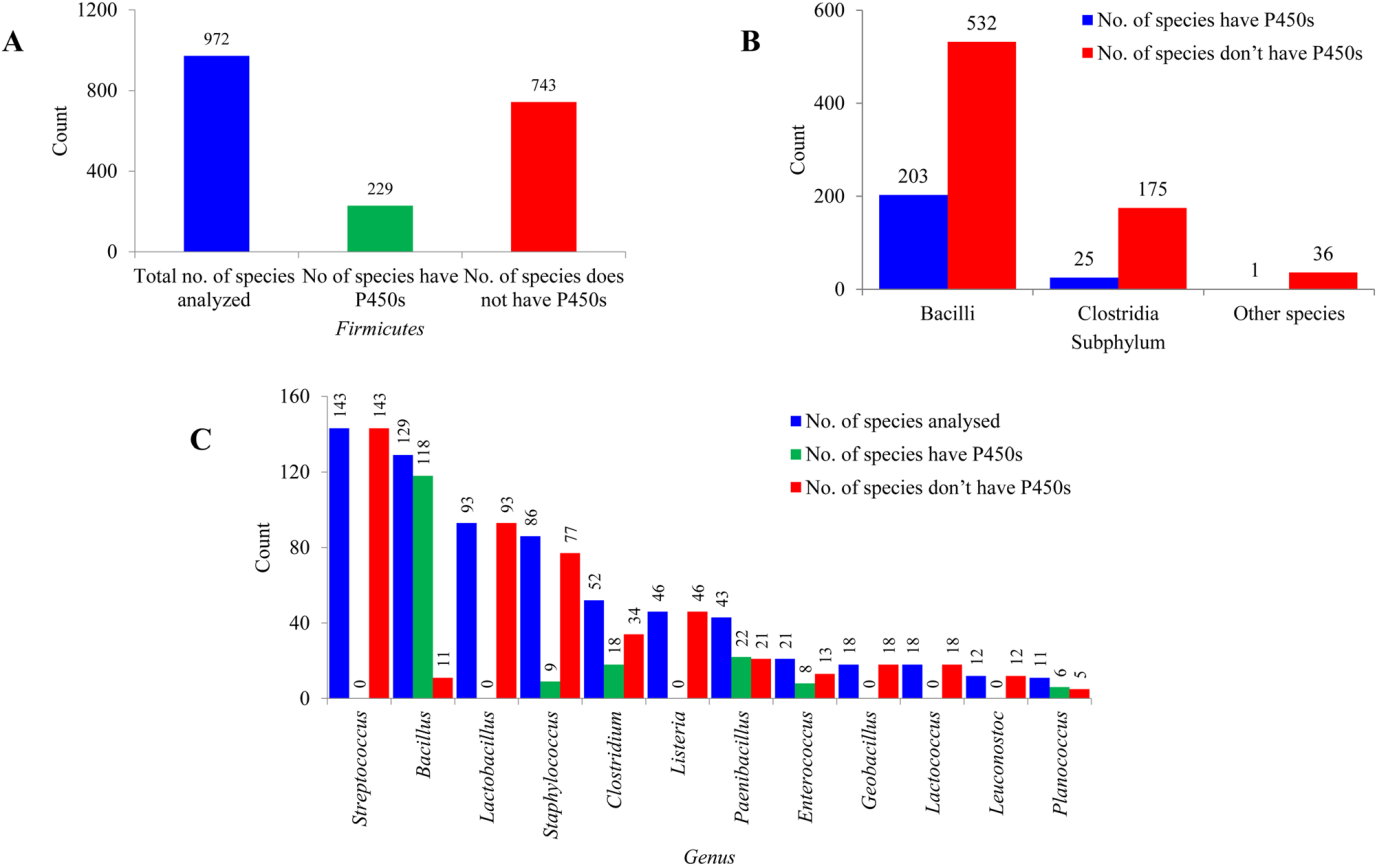
Analysis of P450s in *Firmicutes* species. Comparative analysis of P450s at species level (**A**), subphylum level (**B**) and genera level (**C**) is presented in the figure. Owing to the availability of few species genomes belonging to the other five subphyla of *Firmicutes* (*Erysipelotrichia*, *Limnochordia*, *Negativicutes*, *Thermolithobacteria*, and *Tissierellia*), these species were kept under “Others” at KEGG^50^. Thus, we indicated the P450 profiles in these species under the name Other species. Numbers next to the bars indicate the number of species. Detailed information on *Firmicutes* species P450 profiles is presented in Table S1.

Comprehensive genome-wide data mining and annotation of P450s in 972 *Firmicutes* species (Table S1) revealed that only 229 species have P450s in their genomes (Fig. 1A). This indicates that most of the *Firmicutes* species do not have P450s in their genomes (Fig. 1A). Subphylum level analysis revealed that 38% of *Bacilli* species have P450s (Fig. 1B). In contrast to *Bacilli*, only 14% and 2.7% of *Clostridia* and other *Firmicutes* species have P450s (Fig. 1B). Owing to the availability of few species genomes that belong to the other five *Firmicutes* subphyla (*Erysipelotrichia*, *Limnochordia*, *Negativicutes*, *Thermolithobacteria*, and *Tissierellia*), these species were kept under “Others” at KEGG^50^. Thus, we indicated the P450 profiles in these species under the name “other species”. Only one species (*Limnochorda pilosa*) among 37 species in this category had P450s. Analysis of the *Firmicutes* genera disclosed that of the 158 genera, species belonging to 37 genera have P450s in their genomes (Fig. 1C). Most of the species belonging to the genus *Bacillius* have P450s, followed by *Paenibacillus* and *Clostridium* (Fig. 1C). Based on the number of species used in the study, we can safely conclude that species belonging to the genera *Streptococcus*, *Lactobacillus*, *Listeria*, *Geobacillus*, *Lactococcus* and *Leuconostoc* do not have P450s (Fig. 1C). Furthermore, among 86 *Staphylococcus* species only nine species have P450s (Fig. 1C). Information on genera, species and the number of P450s in a species is presented in Table S1. On average three P450s were found in 229 species, whereas 92 species had a single P450 in their genome (Table S1). Among the *Firmicutes*, species belonging to the genus *Paenibacillus* had the highest number of P450s in their genomes. *P. mucilaginosus* 3016 had the highest number of P450s (11 P450s) in its genome, followed by *P. mucilaginosus* K02 and *P. mucilaginosus* KNP414 (10 P450s each) (Table S1). Comparative analysis of different bacterial species revealed that *Firmicutes* species had the lowest average number of P450s in their genomes compared to the bacterial species belonging to the genera *Streptomyces* and *Mycobacterium* and the phylum *Cyanobacteria* (Table 2). The absence of P450s in species belonging to the genera *Streptococcus*, *Lactobacillus*, *Listeria*, *Lactococcus*, *Leuconostoc* or the presence of a single P450 in a handful of *Staphylococcus* species strongly suggests that the impact of lifestyle profoundly influenced the P450 repertoire in the species, as also observed in mycobacterial species^47^ and in *Saccharomyces* species^37^. The pathogenic lifestyle of species belonging to the genera *Streptococcus*, *Listeria* and *Staphylococcus* led them to adapt readily available carbon sources in the host and thus possibly led to the loss of P450s, similarly to mycobacterial species ^47^. Species belonging to the genera *Lactobacillus*, *Lactococcus* and *Leuconostoc* are involved in fermentation processing (industrial scale or inside the human gut), indicating that adaption to thrive on simple carbon sources led to the loss of P450s, just as observed in *Saccharomyces* species^37^.

**Table 2:** Comparative analysis of key features of P450s and their association with secondary metabolism between *Firmicutes* species and different bacterial species.

	*Firmicutes* Species	*Streptomyces* Species	Mycobacterial Species	Cyanobacterial Species
Total no. of species analyzed	972	203	60	114
No. of P450s	712	5460	1784	341
No. of families	14	253	77	36
No. of subfamilies	53	698	132	79
Dominant P450 family	CYP107	CYP107	CYP125	CYP110
Average no. of P450s	1	27	30	3
No. of P450s part of BGCs	126	1231	204	27
Percentage of P450s part of BGCs	18	23	11	8
Reference(s)	This work	^45,46^	^45,47^	^49^

*BGC*, biosynthetic gene cluster.

### *Firmicutes* species have the lowest P450 diversity

**Figure 2:**
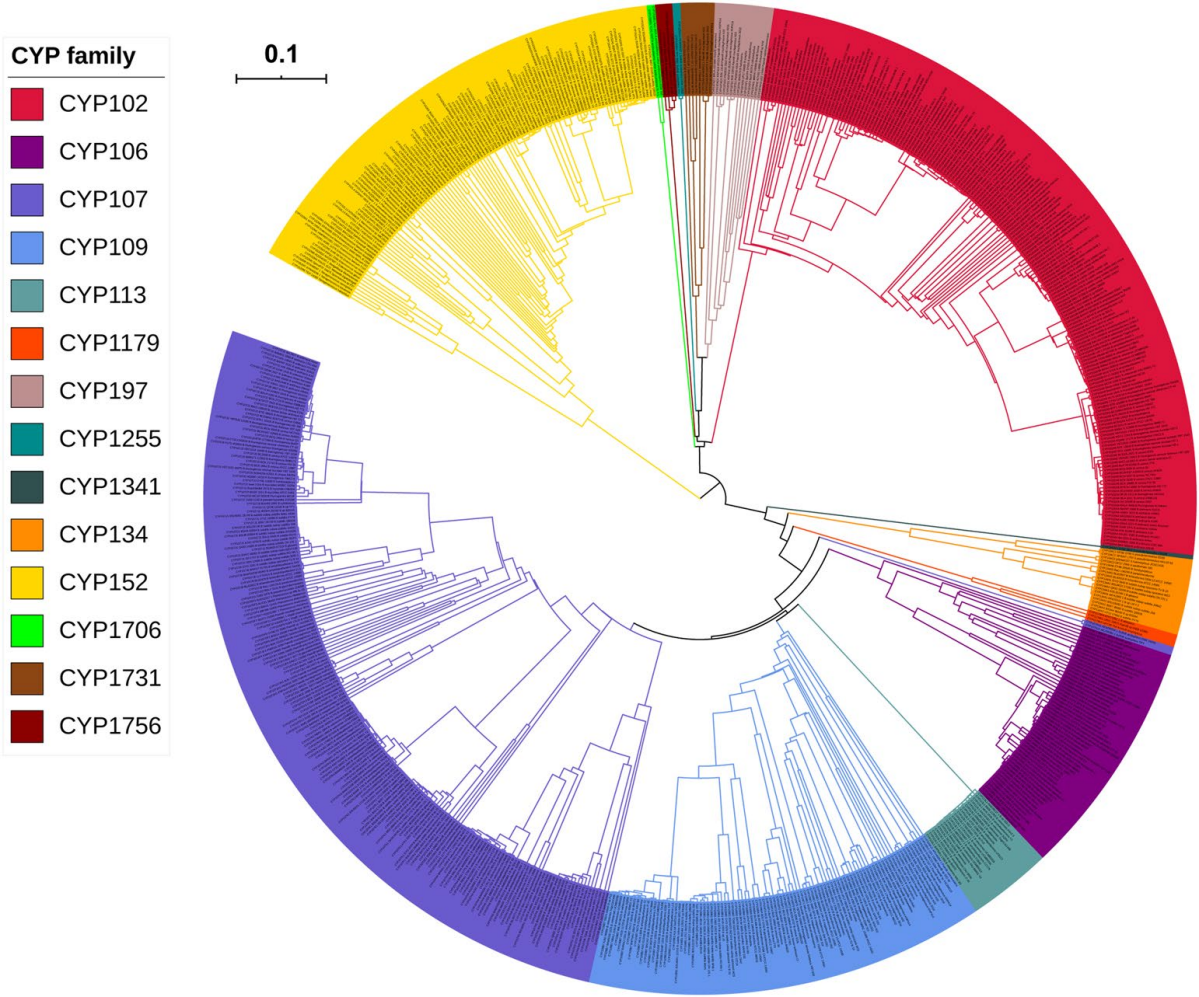
Phylogenetic tree of *Firmicutes* species P450s. Different P450 families are indicated with different colours. A high-resolution phylogenetic tree is provided in Supplementary Dataset 2.

*Firmicutes* species P450s were grouped into different P450 families and P450 subfamilies following the International P450 Nomenclature Committee rules that include phylogenetic analysis of P450s (Fig. 2)^51–53^. Based on the percentage identity of > 40% for a family and > 55% for a subfamily and following the evolutionary analysis where P450s belonging to the same family grouped together (Fig. 2), all 712 P450s found in 229 *Firmicutes* species were grouped into 14 P450 families and 53 P450 subfamilies (Table 3). *Firmicutes* species P450s identified in this study, along with their protein sequences and species, are presented in Supplementary Dataset 1. The number of P450 families found in *Firmicutes* species is very low compared to other bacterial species (Table 2), indicating the lowest P450 diversity. As predicted, P450 families such as CYP107, CYP102, CYP152, CYP109 and CYP106 were expanded in *Firmicutes* species, as the percentage contribution of these families to the total number of P450s was higher compared to the rest of the P450 families (Table 3). Among P450 families, CYP107 had the highest number of P450s (199 P450s), contributing 28% of 712 P450s, followed by CYP102 (179 P450s), CYP152 (110 P450s), CYP109 (95 P450s) and CYP106 (57 P450s) (Table 3). Blooming of certain P450 families in species is a common phenomenon and is observed in species belonging to different biological kingdoms as a potential indication of adaptation to an ecological niche^34,36,38–42,44^. Comparative analysis of dominant P450 families across different bacterial species revealed that the CYP107 family is dominantly present in *Firmicutes* species and *Streptomyces* species and CYP125 and CYP110 are dominantly present in mycobacterial and cyanobacterial species, respectively (Table 2).

**Table 3:** Comparative analysis of P450 families and subfamilies in *Firmicutes* species.

Family	P450 count	Percentage count	Subfamily	P450 count	Percentage contribution
CYP102	179	25.14	A	179	25.14
CYP106	57	8.01	A	15	2.11
			B	41	5.76
			C	1	0.14
CYP107	199	27.95	CB	3	0.42
			DE	3	0.42
			DF	12	1.69
			DY	8	1.12
			H	47	6.60
			J	66	9.27
			JF	7	0.98
			JG	2	0.28
			JH	3	0.42
			JF	1	0.14
			K	45	6.32
			NJ	2	0.28
CYP109	95	13.34	A	23	3.23
			AJ	1	0.14
			B	44	6.18
			E	4	0.56
			J	3	0.42
			T	12	1.69
			U	1	0.14
			V	3	0.42
			W	3	0.42
			X	1	0.14
CYP113	20	2.81	L	20	2.81
CYP1179	3	0.42	A	3	0.42
CYP1255	2	0.28	A	2	0.28
CYP1341	1	0.14	C	1	0.14
CYP134	18	2.53	A	14	1.97
			C	4	0.56
CYP152	110	15.45	A	61	8.57
			AC	1	0.14
			AK	1	0.14
			AL	1	0.14
			AM	1	0.14
			AN	5	0.70
			J	1	0.14
			K	12	1.69
			L	11	1.54
			M	14	1.97
			N	2	0.28
CYP1706	2	0.28	B	2	0.28
CYP1731	8	1.12	A	5	0.70
			B	3	0.42
CYP1756	4	0.56	A	4	0.56
CYP197	14	1.97	A	1	0.14
			AD	3	0.42
			AE	1	0.14
			AF	3	0.42
			AH	3	0.42
			S	3	0.42

The percentage contribution of a particular family and its subfamilies to the total number of P450s is also presented in the table.

**Figure 3:**
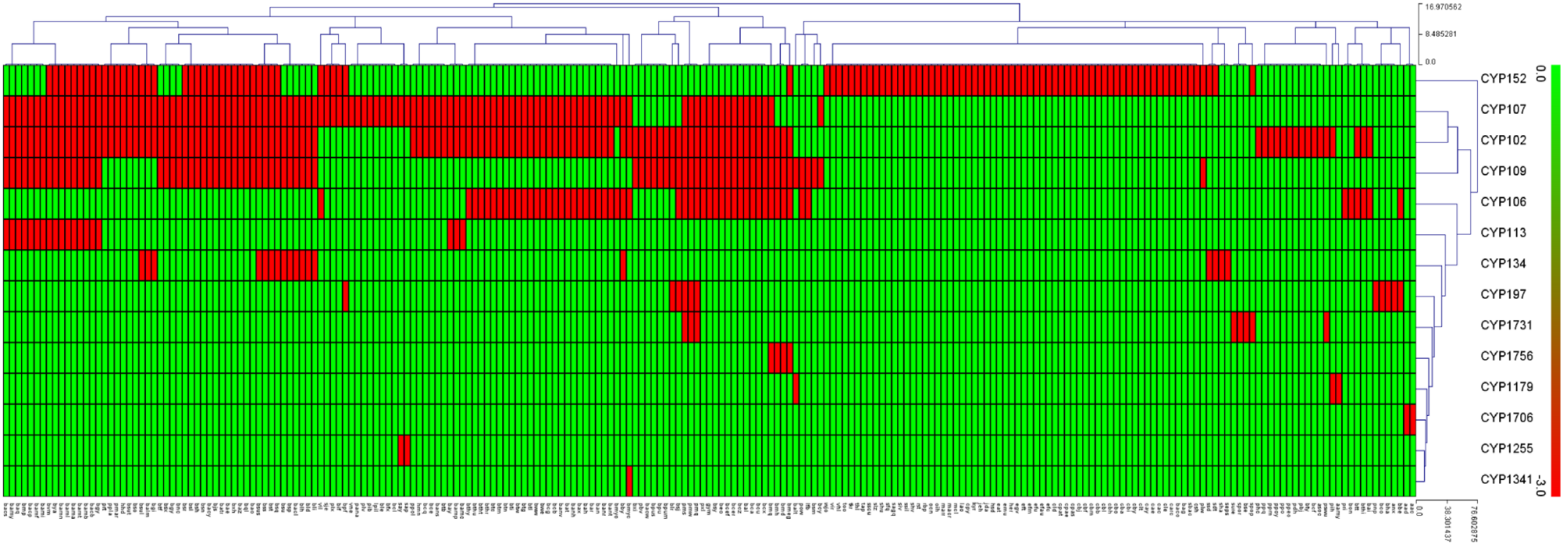
Analysis of presence of P450 family (red) or its absence (green) in 229 *Firmicutes* species. Two hundred and twenty-nine *Firmicutes* species form the horizontal axis and P450 family numbers form the vertical axis. The data used in the generation of this figure are presented in Supplementary Dataset 3.

Analysis of P450 subfamilies revealed subfamily-level blooming in *Firmicutes* species as some subfamilies were expanded in a family (Table 3). CYP107 had the highest number of P450 subfamilies (12 subfamilies), followed by CYP152 (11 subfamilies), CYP109 (10 subfamilies), CYP197 (6 subfamilies), CYP106 (3 subfamilies) and CYP134 and CYP1731 and (Table 3). Seven of 14 P450 families had a single subfamily (Table 3). The dominant subfamilies in expanded P450 families were the following: CYP107 family had the subfamily “J” as the dominant subfamily; subfamily “B” was dominant in P450 families CYP106 and CYP109 and subfamily “A” was dominant in the CYP152 family. It is interesting to note that the CYP102 P450 family, despite contributing the second largest number of P450s, had a single subfamily “A” (Table 3). Blooming of certain P450 subfamilies in a family was also observed in species of different biological kingdoms and it is assumed that the P450 blooms possibly bestow certain advantages on organisms in adapting to particular ecological niches^34,36,38–42,44^. Analysis of conservation of P450 families in 229 *Firmicutes* species revealed that none of the 14 P450 families was conserved in these species (Fig. 3). However, based on the heat-map profile of P450 families, the P450 families CYP152, CYP107, CYP012 and CYP109 were found to be a co-presence in most *Firmicutes* species (Fig. 3). Cyanobacterial species also had no P450 family conserved, but P450 families CYP110 and CYP120 were found to be a co-presence in most of these species^49^. the large CYP107 family was found to be conserved in *Streptomyces* species^46^ and quite a number of P450 families were found to be conserved in mycobacterial species^47^.

### A small proportion of P450s are involved in secondary metabolism in *Firmicutes* species

**Figure 4:**
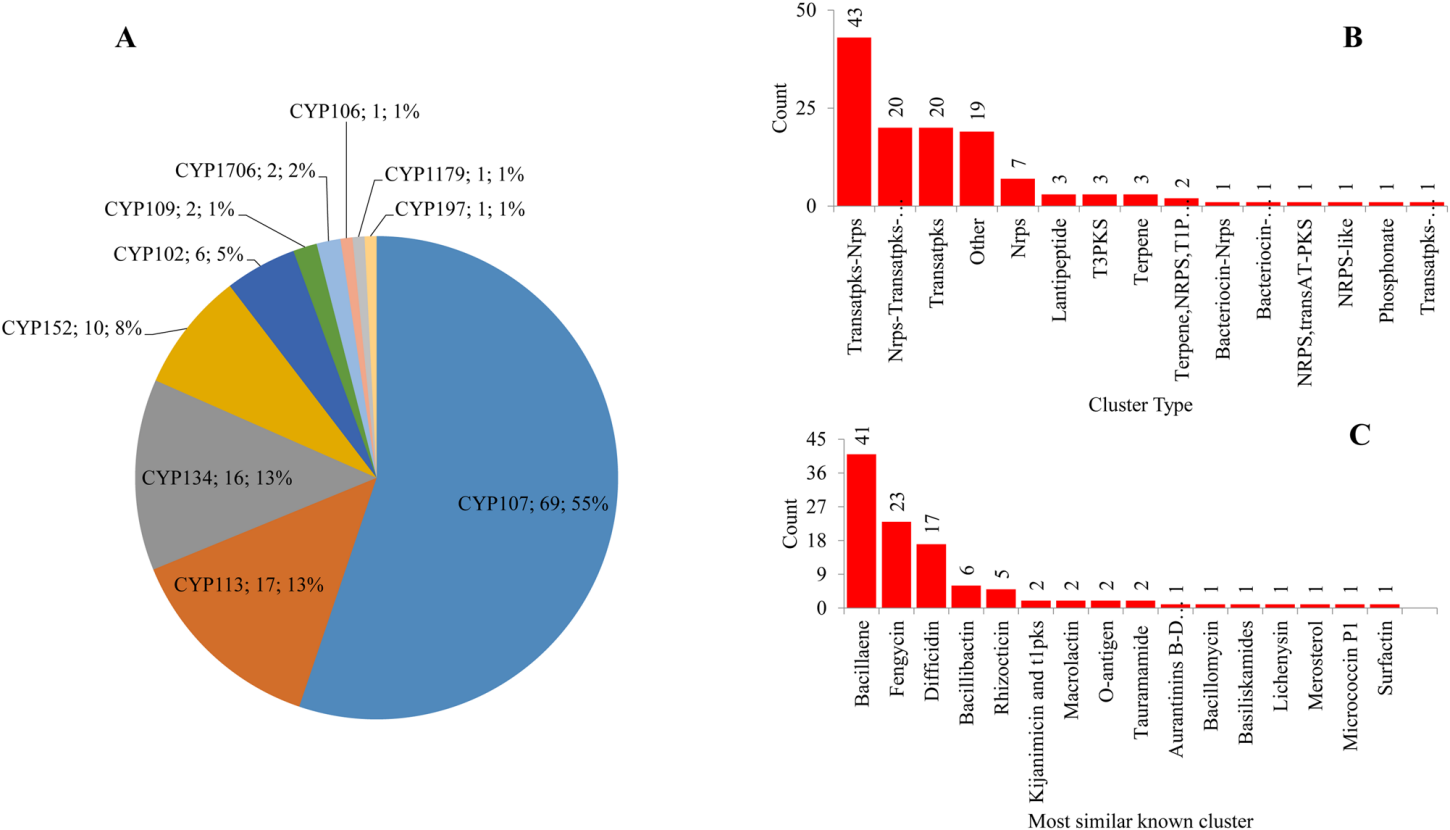
Comparative analysis of P450s associated with secondary metabolism in *Firmicutes* species. (**A**) Comparative analysis of P450 families that are part of secondary metabolite biosynthetic gene clusters (BGCs). The P450 family name, number of P450s and their percentage of the total number of P450s part of BGCs are presented in the figure. (**B**) Comparative analysis of types of BGCs. The number at the top of each bar represents the number of P450s in the type of BGC. (**C**) Comparative analysis of most similar known clusters that have P450s. The number at the top of each bar represents the total number of similar clusters. Detailed information is presented in Table S2.

Among 712 P450s identified in 229 *Firmicutes* species, 125 P450s (18%) of 62 *Firmicutes* species were found to be part of secondary metabolite BGCs (Fig. 4 and Table S2). P450s that were part of BGCs were from species belonging to the subphylum *Bacilli* and most of these species belonged to the genera *Bacillus* (49 species) (Table S2). Most of the *Firmicutes* species P450 families were found to be part of BGCs (Fig. 4A). Among 14 P450 families, ten families, namely CYP107, CYP113, CYP134, CYP152, CYP102, CYP109, CYP1706, CYP106, CYP1179 and CYP197, were found to be part of different secondary metabolite BGCs (Fig. 4A). Among these families, P450s belonging to the CYP107 family were dominantly present in BGCs with more than half of the P450s (55%) were part of BGCs (Fig. 4A). A point to be noted is that P450 families such as CYP107, CYP152, CYP102 and CYP109 are expanded in *Firmicutes* and part of the BGCs also clearly support a previous hypothesis that “species populate specific P450s if they are useful in their adaptation to certain ecological niches or useful in their physiology”^34,36,38–42,44^. Interestingly, some P450 families such as CYP113, CYP134, CYP197, CYP1706 and CYP1179 that are scarcely present in *Firmicutes* species but they are also part of BGCs, indicating their important role in producing these metabolites.

An interesting phenomenon is observed when comparing at the subfamily level where P450 subfamilies “H” and “K” are part of BGCs and the subfamily “J” P450s are not part of BGCs despite this subfamily’s dominance in the CYP107 family (Table 3), indicating subfamily level selectivity by species. P450s were found to be part of 15 BGC types (Fig. 4B). Most of the P450s are part of BGC types such as Transatpks-Nrps (Trans-AT polyketide synthase-Non-ribosomal peptide synthetase cluster) (43 P450s), followed by Nrps-Transatpks-Otherks (Nrps-Transatpks-Other types of PKS cluster) and Transatpks (each 20 P450s) and Other (19 P450s) (Fig. 4B). Analysis of most similar known clusters revealed that the majority of P450 clusters were bacillaene (41 clusters), followed by fengycin (23 clusters) and difficidin (17 clusters) (Fig. 4C). Analysis of the association between P450 families and BGCs showed that CYP107 family P450s are mostly associated with BGCs Nrps-Transatpks-Otherks, Transatpks, Transatpks-Nrps and Other, a putative gene cluster; CYP113 family P450s are associated with Transatpks-Nrps and Transatpks BGC, and CYP134 family P450s are associated with Transatpks-Nrps and Other (Table S2). The percentage identity of *Firmicutes* BGCs containing P450s with similar known clusters revealed that most of the clusters have 100% sequence identity, indicating that these clusters indeed produce the expected metabolites (Table S2).

Comparative analysis of P450s involved in secondary metabolism among different bacterial species revealed that more P450s from *Firmicutes* species (18%) are involved in secondary metabolism compared to the P450s from mycobacterial (11%) and cyanobacterial (8%) species (Table 2). However, P450s from *Firmicutes* species are only second to *Streptomyces* species P450s (23%) in terms of their involvement in secondary metabolism (Table 2). These results strongly support the recent observation that more P450s are involved in secondary metabolism in *Streptomyces* species compared to other bacterial species^46^ and it is no surprise that two thirds of all known antibiotics in the world come from these species^54^.

### Most *Firmicutes* species P450s are orphans of unknown function

Among the 712 *Firmicutes* species P450s, only a handful of P450s are characterized for their physiological functions. The well-known and well-studied P450 CYP102A1 (P450-BM3) from *B. megaterium* is found to be a fatty acid hydroxylase^55–57^. CYP152A1 from *B. subtilis* and CYP152K6 from *B. methanolicus* were found to be peroxygenases that use hydrogen peroxide to drive hydroxylation and decarboxylation of fatty acids^58–60^. CYP107H1 from *B. subtilis* is a pimelic acid hydroxylase involved in biotin synthesis^61,62^. CYP134A1 from *B. subtilis* is involved in synthesis of pulcherriminic acid, a natural product, by three-step oxidative transformation of the diketopiperazine cyclo-l-leucyl-l-leucyl^63^. Based on the P450s’ location in different BGCs and their percentage identity with known similar clusters (most of them have 100% sequence identity) (Table S2), we predict 89 P450 functions in the synthesis of different secondary metabolites such as polyketides (macrolactin, bacillaene, and difficidin), lipopeptides (surfactin, lichenysin and fengycin), phosphono-oligopeptides (rhizocticin) and siderophores (Bacillibactin) (Table 4). These secondary metabolites are well known for their potential biotechnological applications such as antibacterial, antiviral and cytotoxic properties as listed in Table 1.

**Table 4:** Functional prediction of *Firmicutes* species P450s’ involvement in synthesis of secondary metabolites.

P450	Species name	Predicted biological role
CYP134A1	*Bacillus subtilis* subsp. *subtilis* 168	Bacillaene biosynthesis
CYP107K1	*Bacillus subtilis* subsp. *subtilis* RO-NN-1	Bacillaene biosynthesis
CYP107K1	*Bacillus subtilis* subsp. *subtilis* 6051-HGW	Bacillaene biosynthesis
CYP107K1	*Bacillus subtilis* subsp. *subtilis* BAB-1	Bacillaene biosynthesis
CYP107K1	*Bacillus subtilis* subsp. *subtilis* AG1839	Bacillaene biosynthesis
CYP107K1	*Bacillus subtilis* subsp. *subtilis* JH642	Bacillaene biosynthesis
CYP107K1	*Bacillus subtilis* subsp. *subtilis* OH 131.1	Bacillaene biosynthesis
CYP107K1	*Bacillus subtilis* subsp. *spizizenii* W23	Bacillaene biosynthesis
CYP107K1	*Bacillus subtilis* subsp. *spizizenii* TU-B-10	Bacillaene biosynthesis
CYP107K1	*Bacillus subtilis* BSn5	Bacillaene biosynthesis
CYP107K1	*Bacillus subtilis* QB928	Bacillaene biosynthesis
CYP107K1	*Bacillus subtilis* XF-1	Bacillaene biosynthesis
CYP107K1	*Bacillus subtilis* PY79	Bacillaene biosynthesis
CYP107K3	*Bacillus velezensis* FZB42	Bacillaene biosynthesis
CYP107K3	*Bacillus velezensis* CAU B946	Bacillaene biosynthesis
CYP107K3	*Bacillus velezensis* YAU B9601-Y2	Bacillaene biosynthesis
CYP107K3	*Bacillus velezensis* UCMB5036	Bacillaene biosynthesis
CYP107K3	*Bacillus velezensis* UCMB5033	Bacillaene biosynthesis
CYP107K3	*Bacillus velezensis* UCMB5113	Bacillaene biosynthesis
CYP107K3	*Bacillus velezensis* NAU-B3	Bacillaene biosynthesis
CYP107K3	*Bacillus velezensis* TrigoCor1448	Bacillaene biosynthesis
CYP107K3	*Bacillus velezensis* SQR9	Bacillaene biosynthesis
CYP107K3	*Bacillus velezensis*	Bacillaene biosynthesis
CYP107K3	*Bacillus amyloliquefaciens* DSM 7	Bacillaene biosynthesis
CYP107K3	*Bacillus amyloliquefaciens* TA208	Bacillaene biosynthesis
CYP107K3	*Bacillus amyloliquefaciens* LL3	Bacillaene biosynthesis
CYP107K3	*Bacillus amyloliquefaciens* XH7	Bacillaene biosynthesis
CYP107K3	*Bacillus amyloliquefaciens* Y2	Bacillaene biosynthesis
CYP107K3	*Bacillus amyloliquefaciens* IT-45	Bacillaene biosynthesis
CYP107K3	*Bacillus amyloliquefaciens* LFB112	Bacillaene biosynthesis
CYP107K2	*Bacillus atrophaeus* NRS 1221A	Bacillaene biosynthesis
CYP107K3	*Bacillus vallismortis*	Bacillaene biosynthesis
CYP107K1	*Bacillus* sp. JS	Bacillaene biosynthesis
CYP107K3	*Bacillus* sp. Pc3	Bacillaene biosynthesis
CYP107K3	*Bacillus* sp. BH072	Bacillaene biosynthesis
CYP107K1	*Bacillus* sp. YP1	Bacillaene biosynthesis
CYP107K1	*Bacillus* sp. BS34A	Bacillaene biosynthesis
CYP107K1	*Bacillus* sp. LM 4-2	Bacillaene biosynthesis
CYP107K1	*Bacillus gibsonii*	Bacillaene biosynthesis
CYP107K3	*Bacillus* sp. SDLI1	Bacillaene biosynthesis
CYP107K1	*Bacillus subtilis* subsp. *subtilis* BSP1	Bacillaene biosynthesis
CYP152K2	*Solibacillus silvestris* DSM 12223	Bacillibactin biosynthesis
CYP113L1	*Bacillus* sp. Pc3	Bacillibactin biosynthesis
CYP113L1	*Bacillus velezensis* FZB42	Difficidin biosynthesis
CYP113L1	*Bacillus velezensis* CAU B946	Difficidin biosynthesis
CYP113L1	*Bacillus velezensis* YAU B9601-Y2	Difficidin biosynthesis
CYP113L1	*Bacillus velezensis* AS43.3	Difficidin biosynthesis
CYP113L1	*Bacillus velezensis* UCMB5036	Difficidin biosynthesis
CYP113L1	*Bacillus velezensis* UCMB5033	Difficidin biosynthesis
CYP113L1	*Bacillus velezensis* UCMB5113	Difficidin biosynthesis
CYP113L1	*Bacillus velezensis* NAU-B3	Difficidin biosynthesis
CYP113L1	*Bacillus velezensis* SQR9	Difficidin biosynthesis
CYP113L1	*Bacillus velezensis*	Difficidin biosynthesis
CYP113L1	*Bacillus amyloliquefaciens* Y2	Difficidin biosynthesis
CYP113L1	*Bacillus amyloliquefaciens* IT-45	Difficidin biosynthesis
CYP113L1	*Bacillus amyloliquefaciens* CC178	Difficidin biosynthesis
CYP113L1	*Bacillus amyloliquefaciens* LFB112	Difficidin biosynthesis
CYP113L1	*Bacillus vallismortis*	Difficidin biosynthesis
CYP107H4	*Bacillus* sp. BH072	Difficidin biosynthesis
CYP113L1	*Bacillus* sp. SDLI1	Difficidin biosynthesis
CYP107H4	*Bacillus velezensis* FZB42	Fengycin biosynthesis
CYP107H4	*Bacillus velezensis* CAU B946	Fengycin biosynthesis
CYP107H4	*Bacillus velezensis* YAU B9601-Y2	Fengycin biosynthesis
CYP113L1	*Bacillus velezensis* AS43.3	Fengycin biosynthesis
CYP107H4	*Bacillus velezensis* UCMB5036	Fengycin biosynthesis
CYP107H4	*Bacillus velezensis* UCMB5033	Fengycin biosynthesis
CYP107H4	*Bacillus velezensis* UCMB5113	Fengycin biosynthesis
CYP107H4	*Bacillus velezensis* NAU-B3	Fengycin biosynthesis
CYP107H4	*Bacillus velezensis* TrigoCor1448	Fengycin biosynthesis
CYP107H4	*Bacillus velezensis* SQR9	Fengycin biosynthesis
CYP107H4	*Bacillus velezensis*	Fengycin biosynthesis
CYP107H2	*Bacillus amyloliquefaciens* DSM 7	Fengycin biosynthesis
CYP107H2	*Bacillus amyloliquefaciens* TA208	Fengycin biosynthesis
CYP107H2	*Bacillus amyloliquefaciens* LL3	Fengycin biosynthesis
CYP107H2	*Bacillus amyloliquefaciens* XH7	Fengycin biosynthesis
CYP107H4	*Bacillus amyloliquefaciens* Y2	Fengycin biosynthesis
CYP107H4	*Bacillus amyloliquefaciens* IT-45	Fengycin biosynthesis
CYP107H4	*Bacillus amyloliquefaciens* CC178	Fengycin biosynthesis
CYP107H4	*Bacillus amyloliquefaciens* LFB112	Fengycin biosynthesis
CYP107H4	*Bacillus vallismortis*	Fengycin biosynthesis
CYP107H4	*Bacillus* sp. Pc3	Fengycin biosynthesis
CYP107H4	*Bacillus* sp. BH072	Fengycin biosynthesis
CYP107H4	*Bacillus* sp. SDLI1	Fengycin biosynthesis
CYP1179A4	*Bacillus xiamenensis*	Lichenysin biosynthesis
CYP1179A4	*Bacillus altitudinis*	Lichenysin biosynthesis
CYP107K3	*Bacillus velezensis* YAU B9601-Y2	Macrolactin biosynthesis
CYP107K3	*Bacillus velezensis* AS43.3	Macrolactin biosynthesis
CYP152A1	*Bacillus subtilis* subsp. *spizizenii* W23	Rhizocticin biosynthesis
CYP152A9	*Bacillus atrophaeus* 1942	Surfactin biosynthesis

Analysis of association of P450s with BGCs revealed that specific P450 orthologues are involved in the production of the same secondary metabolite (Table 4), indicating horizontal gene transfer of these BGCs among *Bacillus* species, a well-known phenomenon of gene-cluster transfer between bacterial species^33,64,65^. Prominent observations include CYP107K P450s’ involvement in biosynthesis of bacillaene, CYP113L1 P450s’ involvement in the biosynthesis of difficidin and CYP107H4 P450s’ involvement in the biosynthesis of fengycin (Table 4). Considering the high conservation of ortholog P450s, it can be assumed that BGCs such as bacillaene, difficidin and fengycin populated among *Bacillus* species via horizontal gene-cluster transfer.

An interesting comparison can be drawn between the role of secondary metabolites produced by *Firmicutes* where P450s are involved (Table 4) and the role of secondary metabolites produced by *Streptomyces* species^45,46^. In both cases, these secondary metabolites seem to be helping organisms gain the upper hand in their ecological niches, as the secondary metabolites produced by these organisms have anti-bacterial, anti-fungal and anti-viral properties, suggesting that these compounds help both the *Firmicutes*^8^ and the *Streptomyces*^45,46^ species to thrive in their ecological niches by eliminating other organisms. The structure and biological functions of bacillaene, difficidin, fengycin, lichenysin, macrolactin, rhizocticin and surfactin produced by *Firmicutes* were recently reviewed^8^.

## Methods

### Species and database

Nine hundred and seventy-two *Firmicutes* species genomes publicly available at Kyoto Encyclopedia of Genes and Genomes (KEGG)^50^ were used in this study. The species names, species codes, genbank accession numbers and references are presented in Table S1.

### Genome data mining and annotation of P450s

Genome data mining of P450s in *Firmicutes* species was carried out following the standard method described elsewhere^30,45,47,49^. Briefly, using the information and databases presented in Table S1, the whole proteome of each *Firmicutes* species was downloaded and submitted to the NCBI Batch Web CD-Search Tool^66^. Proteins that were assigned as P450 superfamily were chosen and assessed for the presence of characteristics EXXR and CXG motifs^67,68^. Proteins having both motifs were selected for annotation. Annotation of P450s was accomplished following the International P450 Nomenclature Committee rules^51–53^. Proteins with an identity > 40% were classified under the same family and proteins with an identity > 55% were classified under the same subfamily. Proteins with less than 40% identity were assigned to a new P450 family. P450s and gene cluster data for *Bacillus* species were retrieved from a published article and used in the study^30^.

### Phylogenetic analysis of P450s

The phylogenetic tree of *Firmicutes* species P450s was constructed following the method described elsewhere^30,45,47,49^. Briefly, the *Firmicutes* species P450 protein sequences were aligned using the online database, MAFFT^69^. Then the alignments were automatically subjected to tree inferring and optimization by the Trex web server^70^. Finally, the best-inferred trees were visualized, colored, and generated by iTOL^71^.

### Generation of P450 profile heat-maps

Heat-map profiles for P450s were constructed following the method described elsewhere^45,47^. The heat-map profile shows the presence or absence of P450s in *Firmicutes* species. The data were represented as − 3 for family absence (green) and 3 for family presence (red). A tab-delimited file was loaded into MeV (Multi-experiment viewer) using a two-color array^72^. Hierarchical clustering using a Euclidean distance metric was used to cluster the data. Fourteen CYP families formed the vertical axis and 229 *Firmicutes* species formed the horizontal axis.

### Secondary metabolite BGCs analysis and P450s identification

Secondary metabolite BGCs analysis and P450 identification in *Firmicutes* species were carried out following the method described elsewhere^45,47^. Briefly, each *Firmicutes* species genome ID presented in Table S1 was submitted to anti-SMASH^73^ for the identification of secondary metabolite BGCs. The results were downloaded both in the form of gene cluster sequences and Excel spreadsheets. All gene cluster sequences for each species was downloaded and captured in a separate Word file. Using these data, P450s that were part of a specific gene cluster were identified. Standard gene cluster abbreviation terminology available at the anti-SMASH database^73^ was maintained in this study.

### Bacterial P450s and gene cluster data

P450s and their BGCs data for *Streptomyces* species^45,46^, mycobacterial species^47^ and cyanobacterial species^49^ were retrieved from published articles and used for comparative analysis.

### Functional prediction of P450s

Functional prediction of P450s was carried out based on their location in a particular BGC and the percentage sequence identity of BGCs with characterized gene clusters where more than > 70% identity was set as a cut-off value. However, in the case of *Firmicutes* species most BGCs have 100% sequence identity with the characterized gene cluster, indicating P450s’ definite role in the production of a specific secondary metabolite.

## Conclusions

Cytochrome P450 monooxygenases (CYPs/P450s) have been well known to biologists for more than five decades. These enzymes perform enzymatic reactions in stereo- and regio-specific manner and have thus gained attention in the production of chemicals valuable to human beings. Studies indicated that P450s performing such enzymatic reactions help organisms to adapt to specific ecological niches. This includes loss of P450s if an organism adapts to a lifestyle where abundant simple carbon sources are present. In this study, we examined such a phenomenon where *Firmicutes* species that are pathogens and adapted to simple lifestyles lost P450s in their genomes. This strongly supports the earlier hypothesis put forward by our laboratory that the impact of lifestyle shapes P450 content in an organism. Analysis of P450s in *Firmicutes* species revealed the presence of the lowest number of P450s and the lowest P450 diversity in these species compared to bacterial species belonging to the genera *Streptomyces* and *Mycobacterium* and from the phylum *Cyanobacterium*. The lowest P450 diversity is due to the P450 family blooming/expansion especially in P450 families such as CYP107, CYP102, CYP152, CYP109 and CYP106. *Firmicutes* species were found to have the second highest number of BGCs in their genomes after *Streptomyces*. Most of the P450s belonging to the expanded families were found to be part of BGCs indicating that these P450s were favored by these species, possibly to gain some advantage, as observed in other organisms. Interestingly, the preference of certain P450 subfamilies for being part of BGCs were found in *Firmicutes* species despite these P450 subfamilies not being expanded. Based on the presence of ortholog P450s in *Firmicutes* species and the highest percentage of sequence identity with characterized BGCs, in this study we successfully predicted 89 P450s’ function in the synthesis of different secondary metabolites. Interestingly, most of these P450s are involved in the synthesis of bacillaene and difficidin (polyketides) and fengycin (lipopeptide). Based on the P450 profiles among species belonging to the same phylum and their lifestyles, one can connect P450 profiles to their lifestyles and this concept has been successfully demonstrated in eukaryotes and in some prokaryotes. It is worth mentioning that the phylogenetic age of bacteria from old to young is *Firmicutes*, *Streptomyces*, *Cyanobacteria* and *Mycobacterium*^74^, indicating that there is no link with the number and diversity of P450s and the age of the bacteria and thus P450 profiles may be more strongly related to the lifestyle. Studies are in progress to determine the impact of lifestyle in other bacterial species.

## Supplementary information

Supplementary file1

Supplementary file2

Supplementary file3

Supplementary file4
