# Supplementary material for: Impact of lifestyle on cytochrome P450 monooxygenase repertoire is clearly evident in the bacterial phylum Firmicutes
Source: Sci Rep. 2020 Aug 19;10:13982. doi: 10.1038/s41598-020-70686-8 (PMC7438502; doi:10.1038/s41598-020-70686-8)
Supplement: Supplementary file 1 — Supplementary file1 [file 41598_2020_70686_MOESM1_ESM.docx]

**Impact of lifestyle on cytochrome P450 monooxygenase repertoire is clearly evident in the bacterial phylum *Firmicutes***

Tiara Padayachee^1^, Nomfundo Nzuza^1^, Wanping Chen^2^, David R Nelson^3^*, Khajamohiddin Syed^1*^

^1^ Department of Biochemistry and Microbiology, Faculty of Science and Agriculture, University of Zululand, KwaDlangezwa 3886, South Africa.

^2^ Department of Molecular Microbiology and Genetics, University of Göttingen, 37077 Göttingen, Germany

^3^ Department of Microbiology, Immunology and Biochemistry, University of Tennessee Health Science Center, Memphis, TN, 38163; drnelson1@gmail.com

* Corresponding authors’ email:

drnelson1@gmail.com and khajamohiddinsyed@gmail.com

**Supplementary Dataset 1.** List of P450s identified in different *Firmicutes* species. Each P450 is presented with its given name followed by protein ID in parenthesis and species name.

>CYP102A3_ortholog(BSNT_09106)Bacillus subtilis subsp. natto BEST195

MLMKQASAIPQPKTYGPLKNLPHLEKEQLSQSLWRIADELGPIFRFDFPGVSSVFVSGHNFVAEVCDESRFDKNLGKGLQKVREFGGDGLFTSWTHEPNWQKAHRILLPSFSQKAMKGYHSMMLDIATQLIQKWSRLNPNEEIDVADDMTRLTLDTIGLCGFNYRFNSFYRDSQHPFITSMLRALKEAMNQSKRLGLQDKMMVKTKLQFQKDIEVMNSLVDRMIAERKANPDDNIKDLLSLMLYAKDPVTGETLDDENIRYQIITFLIAGHETTSGLLSFAIYCLLTHPEKLKKA

>CYP102A4_ortholog(BAMEG_1392)Bacillus anthracis CDC 684

MDKKVSAIPQPKTYGPLGNLPLIDKDKPTLSFIKLAEEYGPIFRMQTLSDTIIVVSGHELVAEVCDETRFDKSIEGALAKVRAFAGDGLFTSETQEPNWQKAHNILMPTFSQRAMKDYHAMMVDIAVQLVQKWARLNPNENVDVPEDMTRLTLDTIGLCGFNYRFNSFYRETPHPFITSMTRALDEAMHQLQRLDIEDKLMWRTKRQFQHDIQSMFSLVDNIIAERKSSENQEENDLLSRMLNVQDPETGEKLDDENIRFQIITFLIAGHETTSGLLSFAIYFLLKNPDKLKKAYEEVDRVLTDSTPTYQQVMKLKYIRMILNESLRLWPTAPAFSLYAKEDTVIGGKYPIKKGEDRISVLIPQLHRDKDAWGDNVEEFQPERFEELDKVPHHAYKPFGNGQRACIGMQFALHEATLVMGMLLQHFEFIDYEEYQLDVKQTLTLKPGDFKIRIVPRNQTISHTTVLAPTEEKLKNHEIKQQVQKTPSIIGADNLSLLVLYGSDTGVAEGIARELADTASLEGVQTEVAALNDRIGSLPKEGAVLIVTSSYNGKPPSNAGQFVQWLEELKGDELKGVQYAVFGCGDHNWASTYQRIPRYIDEQMAQKGATRFSTRGEADASGDFEEQLEQWKQRMWSDAMKVFGLELNKNMEKERSTLSLQFVSRLGGSPLARTYEAVYASILENRELQSSSSERSTRHIEISLPEGATYKEGDHLGVLPINSEKNVNRILKRFGLNGKDQVILSASGRSVNHIPLDSPVRLYDLLSYSVEVQEAATRAQIREMVTFTACPPHKKELESLLEDGVYQEQILKKRISMLDLLEKYEACEIRFEPFLELLPALKPRYYSISSSPLVAQDRLSITVGVVNAPAWSGEGTYEGVASNYLAQRHNKDEIICFIRTPQSNFQLPENPETPIIMVGPGTGIAPFRGFLQARRVQKQKGMNVGEAHLYFGCRHPEKDYLYRTELENDERDGLISLHTAFSRLEGQAKTYVQHVIKEDRIHLISLLDNGAHLYICGDGSKMAPDVEDTLCQAYQEIHEVSEQEARNWLDRLQEEGRYGKDVWAGI

>CYP102A4_ortholog(BAA_3269)Bacillus anthracis A0248

MDKKVSAIPQPKTYGPLGNLPLIDKDKPTLSFIKLAEEYGPIFRMQTLSDTIIVVSGHELVAEVCDETRFDKSIEGALAKVRAFAGDGLFTSETQEPNWQKAHNILMPTFSQRAMKDYHAMMVDIAVQLVQKWARLNPNENVDVPEDMTRLTLDTIGLCGFNYRFNSFYRETPHPFITSMTRALDEAMHQLQRLDIEDKLMWRTKRQFQHDIQSMFSLVDNIIAERKSSENQEENDLLSRMLNVQDPETGEKLDDENIRFQIITFLIAGHETTSGLLSFAIYFLLKNPDKLKKAYEEVDRVLTDSTPTYQQVMKLKYIRMILNESLRLWPTAPAFSLYAKEDTVIGGKYPIKKGEDRISVLIPQLHRDKDAWGDNVEEFQPERFEELDKVPHHAYKPFGNGQRACIGMQFALHEATLVMGMLLQHFEFIDYEEYQLDVKQTLTLKPGDFKIRIVPRNQTISHTTVLAPTEEKLKNHEIKQQVQKTPSIIGADNLSLLVLYGSDTGVAEGIARELADTASLEGVQTEVAALNDRIGSLPKEGAVLIVTSSYNGKPPSNAGQFVQWLEELKGDELKGVQYAVFGCGDHNWASTYQRIPRYIDEQMAQKGATRFSTRGEADASGDFEEQLEQWKQRMWSDAMKVFGLELNKNMEKERSTLSLQFVSRLGGSPLARTYEAVYASILENRELQSSSSERSTRHIEISLPEGATYKEGDHLGVLPINSEKNVNRILKRFGLNGKDQVILSASGRSVNHIPLDSPVRLYDLLSYSVEVQEAATRAQIREMVTFTACPPHKKELESLLEDGVYQEQILKKRISMLDLLEKYEACEIRFEPFLELLPALKPRYYSISSSPLVAQDRLSITVGVVNAPAWSGEGTYEGVASNYLAQRHNKDEIICFIRTPQSNFQLPENPETPIIMVGPGTGIAPFRGFLQARRVQKQKGMNVGEAHLYFGCRHPEKDYLYRTELENDERDGLISLHTAFSRLEGQAKTYVQHVIKEDRIHLISLLDNGAHLYICGDGSKMAPDVEDTLCQAYQEIHEVSEQEARNWLDRLQEEGRYGKDVWAGI

>CYP102A4_ortholog(BA_3221)Bacillus anthracis Ames

MDKKVSAIPQPKTYGPLGNLPLIDKDKPTLSFIKLAEEYGPIFRMQTLSDTIIVVSGHELVAEVCDETRFDKSIEGALAKVRAFAGDGLFTSETQEPNWQKAHNILMPTFSQRAMKDYHAMMVDIAVQLVQKWARLNPNENVDVPEDMTRLTLDTIGLCGFNYRFNSFYRETPHPFITSMTRALDEAMHQLQRLDIEDKLMWRTKRQFQHDIQSMFSLVDNIIAERKSSENQEENDLLSRMLNVQDPETGEKLDDENIRFQIITFLIAGHETTSGLLSFAIYFLLKNPDKLKKAYEEVDRVLTDSTPTYQQVMKLKYIRMILNESLRLWPTAPAFSLYAKEDTVIGGKYPIKKGEDRISVLIPQLHRDKDAWGDNVEEFQPERFEELDKVPHHAYKPFGNGQRACIGMQFALHEATLVMGMLLQHFEFIDYEEYQLDVKQTLTLKPGDFKIRIVPRNQTISHTTVLAPTEEKLKNHEIKQQVQKTPSIIGADNLSLLVLYGSDTGVAEGIARELADTASLEGVQTEVAALNDRIGSLPKEGAVLIVTSSYNGKPPSNAGQFVQWLEELKGDELKGVQYAVFGCGDHNWASTYQRIPRYIDEQMAQKGATRFSTRGEADASGDFEEQLEQWKQRMWSDAMKVFGLELNKNMEKERSTLSLQFVSRLGGSPLARTYEAVYASILENRELQSSSSERSTRHIEISLPEGATYKEGDHLGVLPINSEKNVNRILKRFGLNGKDQVILSASGRSVNHIPLDSPVRLYDLLSYSVEVQEAATRAQIREMVTFTACPPHKKELESLLEDGVYQEQILKKRISMLDLLEKYEACEIRFEPFLELLPALKPRYYSISSSPLVAQDRLSITVGVVNAPAWSGEGTYEGVASNYLAQRHNKDEIICFIRTPQSNFQLPENPETPIIMVGPGTGIAPFRGFLQARRVQKQKGMNVGEAHLYFGCRHPEKDYLYRTELENDERDGLISLHTAFSRLEGQAKTYVQHVIKEDRIHLISLLDNGAHLYICGDGSKMAPDVEDTLCQAYQEIHEVSEQEARNWLDRLQEEGRYGKDVWAGI

>CYP102A4_ortholog(HYU01_1585)Bacillus anthracis HYU01

MDKKVSAIPQPKTYGPLGNLPLIDKDKPTLSFIKLAEEYGPIFRMQTLSDTIIVVSGHELVAEVCDETRFDKSIEGALAKVRAFAGDGLFTSETQEPNWQKAHNILMPTFSQRAMKDYHAMMVDIAVQLVQKWARLNPNENVDVPEDMTRLTLDTIGLCGFNYRFNSFYRETPHPFITSMTRALDEAMHQLQRLDIEDKLMWRTKRQFQHDIQSMFSLVDNIIAERKSSENQEENDLLSRMLNVQDPETGEKLDDENIRFQIITFLIAGHETTSGLLSFAIYFLLKNPDKLKKAYEEVDRVLTDSTPTYQQVMKLKYIRMILNESLRLWPTAPAFSLYAKEDTVIGGKYPIKKGEDRISVLIPQLHRDKDAWGDNVEEFQPERFEELDKVPHHAYKPFGNGQRACIGMQFALHEATLVMGMLLQHFEFIDYEEYQLDVKQTLTLKPGDFKIRIVPRNQTISHTTVLAPTEEKLKNHEIKQQVQKTPSIIGADNLSLLVLYGSDTGVAEGIARELADTASLEGVQTEVAALNDRIGSLPKEGAVLIVTSSYNGKPPSNAGQFVQWLEELKGDELKGVQYAVFGCGDHNWASTYQRIPRYIDEQMAQKGATRFSTRGEADASGDFEEQLEQWKQRMWSDAMKVFGLELNKNMEKERSTLSLQFVSRLGGSPLARTYEAVYASILENRELQSSSSERSTRHIEISLPEGATYKEGDHLGVLPINSEKNVNRILKRFGLNGKDQVILSASGRSVNHIPLDSPVRLYDLLSYSVEVQEAATRAQIREMVTFTACPPHKKELESLLEDGVYQEQILKKRISMLDLLEKYEACEIRFEPFLELLPALKPRYYSISSSPLVAQDRLSITVGVVNAPAWSGEGTYEGVASNYLAQRHNKDEIICFIRTPQSNFQLPENPETPIIMVGPGTGIAPFRGFLQARRVQKQKGMNVGEAHLYFGCRHPEKDYLYRTELENDERDGLISLHTAFSRLEGQAKTYVQHVIKEDRIHLISLLDNGAHLYICGDGSKMAPDVEDTLCQAYQEIHEVSEQEARNWLDRLQEEGRYGKDVWAGI

>CYP102A4_ortholog(BAPAT_3089)Bacillus anthracis SVA11

MLFERVFVMDKKVSAIPQPKTYGPLGNLPLIDKDKPTLSFIKLAEEYGPIFRMQTLSDTIIVVSGHELVAEVCDETRFDKSIEGALAKVRAFAGDGLFTSETQEPNWQKAHNILMPTFSQRAMKDYHAMMVDIAVQLVQKWARLNPNENVDVPEDMTRLTLDTIGLCGFNYRFNSFYRETPHPFITSMTRALDEAMHQLQRLDIEDKLMWRTKRQFQHDIQSMFSLVDNIIAERKSSENQEENDLLSRMLNVQDPETGEKLDDENIRFQIITFLIAGHETTSGLLSFAIYFLLKNPDKLKKAYEEVDRVLTDSTPTYQQVMKLKYIRMILNESLRLWPTAPAFSLYAKEDTVIGGKYPIKKGEDRISVLIPQLHRDKDAWGDNVEEFQPERFEELDKVPHHAYKPFGNGQRACIGMQFALHEATLVMGMLLQHFEFIDYEEYQLDVKQTLTLKPGDFKIRIVPRNQTISHTTVLAPTEEKLKNHEIKQQVQKTPSIIGADNLSLLVLYGSDTGVAEGIARELADTASLEGVQTEVAALNDRIGSLPKEGAVLIVTSSYNGKPPSNAGQFVQWLEELKGDELKGVQYAVFGCGDHNWASTYQRIPRYIDEQMAQKGATRFSTRGEADASGDFEEQLEQWKQRMWSDAMKVFGLELNKNMEKERSTLSLQFVSRLGGSPLARTYEAVYASILENRELQSSSSERSTRHIEISLPEGATYKEGDHLGVLPINSEKNVNRILKRFGLNGKDQVILSASGRSVNHIPLDSPVRLYDLLSYSVEVQEAATRAQIREMVTFTACPPHKKELESLLEDGVYQEQILKKRISMLDLLEKYEACEIRFEPFLELLPALKPRYYSISSSPLVAQDRLSITVGVVNAPAWSGEGTYEGVASNYLAQRHNKDEIICFIRTPQSNFQLPENPETPIIMVGPGTGIAPFRGFLQARRVQKQKGMNVGEAHLYFGCRHPEKDYLYRTELENDERDGLISLHTAFSRLEGQAKTYVQHVIKEDRIHLISLLDNGAHLYICGDGSKMAPDVEDTLCQAYQEIHEVSEQEARNWLDRLQEEGRYGKDVWAGI

>CYP102A4_ortholog(A16_32460)Bacillus anthracis A16

MDKKVSAIPQPKTYGPLGNLPLIDKDKPTLSFIKLAEEYGPIFRMQTLSDTIIVVSGHELVAEVCDETRFDKSIEGALAKVRAFAGDGLFTSETQEPNWQKAHNILMPTFSQRAMKDYHAMMVDIAVQLVQKWARLNPNENVDVPEDMTRLTLDTIGLCGFNYRFNSFYRETPHPFITSMTRALDEAMHQLQRLDIEDKLMWRTKRQFQHDIQSMFSLVDNIIAERKSSENQEENDLLSRMLNVQDPETGEKLDDENIRFQIITFLIAGHETTSGLLSFAIYFLLKNPDKLKKAYEEVDRVLTDSTPTYQQVMKLKYIRMILNESLRLWPTAPAFSLYAKEDTVIGGKYPIKKGEDRISVLIPQLHRDKDAWGDNVEEFQPERFEELDKVPHHAYKPFGNGQRACIGMQFALHEATLVMGMLLQHFEFIDYEEYQLDVKQTLTLKPGDFKIRIVPRNQTISHTTVLAPTEEKLKNHEIKQQVQKTPSIIGADNLSLLVLYGSDTGVAEGIARELADTASLEGVQTEVAALNDRIGSLPKEGAVLIVTSSYNGKPPSNAGQFVQWLEELKGDELKGVQYAVFGCGDHNWASTYQRIPRYIDEQMAQKGATRFSTRGEADASGDFEEQLEQWKQRMWSDAMKVFGLELNKNMEKERSTLSLQFVSRLGGSPLARTYEAVYASILENRELQSSSSERSTRHIEISLPEGATYKEGDHLGVLPINSEKNVNRILKRFGLNGKDQVILSASGRSVNHIPLDSPVRLYDLLSYSVEVQEAATRAQIREMVTFTACPPHKKELESLLEDGVYQEQILKKRISMLDLLEKYEACEIRFEPFLELLPALKPRYYSISSSPLVAQDRLSITVGVVNAPAWSGEGTYEGVASNYLAQRHNKDEIICFIRTPQSNFQLPENPETPIIMVGPGTGIAPFRGFLQARRVQKQKGMNVGEAHLYFGCRHPEKDYLYRTELENDERDGLISLHTAFSRLEGQAKTYVQHVIKEDRIHLISLLDNGAHLYICGDGSKMAPDVEDTLCQAYQEIHEVSEQEARNWLDRLQEEGRYGKDVWAGI

>CYP102A4_ortholog(DJ46_1971)Bacillus anthracis Vollum

MDKKVSAIPQPKTYGPLGNLPLIDKDKPTLSFIKLAEEYGPIFRMQTLSDTIIVVSGHELVAEVCDETRFDKSIEGALAKVRAFAGDGLFTSETQEPNWQKAHNILMPTFSQRAMKDYHAMMVDIAVQLVQKWARLNPNENVDVPEDMTRLTLDTIGLCGFNYRFNSFYRETPHPFITSMTRALDEAMHQLQRLDIEDKLMWRTKRQFQHDIQSMFSLVDNIIAERKSSENQEENDLLSRMLNVQDPETGEKLDDENIRFQIITFLIAGHETTSGLLSFAIYFLLKNPDKLKKAYEEVDRVLTDSTPTYQQVMKLKYIRMILNESLRLWPTAPAFSLYAKEDTVIGGKYPIKKGEDRISVLIPQLHRDKDAWGDNVEEFQPERFEELDKVPHHAYKPFGNGQRACIGMQFALHEATLVMGMLLQHFEFIDYEEYQLDVKQTLTLKPGDFKIRIVPRNQTISHTTVLAPTEEKLKNHEIKQQVQKTPSIIGADNLSLLVLYGSDTGVAEGIARELADTASLEGVQTEVAALNDRIGSLPKEGAVLIVTSSYNGKPPSNAGQFVQWLEELKGDELKGVQYAVFGCGDHNWASTYQRIPRYIDEQMAQKGATRFSTRGEADASGDFEEQLEQWKQRMWSDAMKVFGLELNKNMEKERSTLSLQFVSRLGGSPLARTYEAVYASILENRELQSSSSERSTRHIEISLPEGATYKEGDHLGVLPINSEKNVNRILKRFGLNGKDQVILSASGRSVNHIPLDSPVRLYDLLSYSVEVQEAATRAQIREMVTFTACPPHKKELESLLEDGVYQEQILKKRISMLDLLEKYEACEIRFEPFLELLPALKPRYYSISSSPLVAQDRLSITVGVVNAPAWSGEGTYEGVASNYLAQRHNKDEIICFIRTPQSNFQLPENPETPIIMVGPGTGIAPFRGFLQARRVQKQKGMNVGEAHLYFGCRHPEKDYLYRTELENDERDGLISLHTAFSRLEGQAKTYVQHVIKEDRIHLISLLDNGAHLYICGDGSKMAPDVEDTLCQAYQEIHEVSEQEARNWLDRLQEEGRYGKDVWAGI

>CYP102A4_ortholog(GBAA_3221)Bacillus anthracis Ames Ancestor

MDKKVSAIPQPKTYGPLGNLPLIDKDKPTLSFIKLAEEYGPIFRMQTLSDTIIVVSGHELVAEVCDETRFDKSIEGALAKVRAFAGDGLFTSETQEPNWQKAHNILMPTFSQRAMKDYHAMMVDIAVQLVQKWARLNPNENVDVPEDMTRLTLDTIGLCGFNYRFNSFYRETPHPFITSMTRALDEAMHQLQRLDIEDKLMWRTKRQFQHDIQSMFSLVDNIIAERKSSENQEENDLLSRMLNVQDPETGEKLDDENIRFQIITFLIAGHETTSGLLSFAIYFLLKNPDKLKKAYEEVDRVLTDSTPTYQQVMKLKYIRMILNESLRLWPTAPAFSLYAKEDTVIGGKYPIKKGEDRISVLIPQLHRDKDAWGDNVEEFQPERFEELDKVPHHAYKPFGNGQRACIGMQFALHEATLVMGMLLQHFEFIDYEEYQLDVKQTLTLKPGDFKIRIVPRNQTISHTTVLAPTEEKLKNHEIKQQVQKTPSIIGADNLSLLVLYGSDTGVAEGIARELADTASLEGVQTEVAALNDRIGSLPKEGAVLIVTSSYNGKPPSNAGQFVQWLEELKGDELKGVQYAVFGCGDHNWASTYQRIPRYIDEQMAQKGATRFSTRGEADASGDFEEQLEQWKQRMWSDAMKVFGLELNKNMEKERSTLSLQFVSRLGGSPLARTYEAVYASILENRELQSSSSERSTRHIEISLPEGATYKEGDHLGVLPINSEKNVNRILKRFGLNGKDQVILSASGRSVNHIPLDSPVRLYDLLSYSVEVQEAATRAQIREMVTFTACPPHKKELESLLEDGVYQEQILKKRISMLDLLEKYEACEIRFEPFLELLPALKPRYYSISSSPLVAQDRLSITVGVVNAPAWSGEGTYEGVASNYLAQRHNKDEIICFIRTPQSNFQLPENPETPIIMVGPGTGIAPFRGFLQARRVQKQKGMNVGEAHLYFGCRHPEKDYLYRTELENDERDGLISLHTAFSRLEGQAKTYVQHVIKEDRIHLISLLDNGAHLYICGDGSKMAPDVEDTLCQAYQEIHEVSEQEARNWLDRLQEEGRYGKDVWAGI

>CYP102A4_ortholog(A16R_32890)Bacillus anthracis A16R

MDKKVSAIPQPKTYGPLGNLPLIDKDKPTLSFIKLAEEYGPIFRMQTLSDTIIVVSGHELVAEVCDETRFDKSIEGALAKVRAFAGDGLFTSETQEPNWQKAHNILMPTFSQRAMKDYHAMMVDIAVQLVQKWARLNPNENVDVPEDMTRLTLDTIGLCGFNYRFNSFYRETPHPFITSMTRALDEAMHQLQRLDIEDKLMWRTKRQFQHDIQSMFSLVDNIIAERKSSENQEENDLLSRMLNVQDPETGEKLDDENIRFQIITFLIAGHETTSGLLSFAIYFLLKNPDKLKKAYEEVDRVLTDSTPTYQQVMKLKYIRMILNESLRLWPTAPAFSLYAKEDTVIGGKYPIKKGEDRISVLIPQLHRDKDAWGDNVEEFQPERFEELDKVPHHAYKPFGNGQRACIGMQFALHEATLVMGMLLQHFEFIDYEEYQLDVKQTLTLKPGDFKIRIVPRNQTISHTTVLAPTEEKLKNHEIKQQVQKTPSIIGADNLSLLVLYGSDTGVAEGIARELADTASLEGVQTEVAALNDRIGSLPKEGAVLIVTSSYNGKPPSNAGQFVQWLEELKGDELKGVQYAVFGCGDHNWASTYQRIPRYIDEQMAQKGATRFSTRGEADASGDFEEQLEQWKQRMWSDAMKVFGLELNKNMEKERSTLSLQFVSRLGGSPLARTYEAVYASILENRELQSSSSERSTRHIEISLPEGATYKEGDHLGVLPINSEKNVNRILKRFGLNGKDQVILSASGRSVNHIPLDSPVRLYDLLSYSVEVQEAATRAQIREMVTFTACPPHKKELESLLEDGVYQEQILKKRISMLDLLEKYEACEIRFEPFLELLPALKPRYYSISSSPLVAQDRLSITVGVVNAPAWSGEGTYEGVASNYLAQRHNKDEIICFIRTPQSNFQLPENPETPIIMVGPGTGIAPFRGFLQARRVQKQKGMNVGEAHLYFGCRHPEKDYLYRTELENDERDGLISLHTAFSRLEGQAKTYVQHVIKEDRIHLISLLDNGAHLYICGDGSKMAPDVEDTLCQAYQEIHEVSEQEARNWLDRLQEEGRYGKDVWAGI

>CYP102A4_ortholog(BAS2993)Bacillus anthracis Sterne

MDKKVSAIPQPKTYGPLGNLPLIDKDKPTLSFIKLAEEYGPIFRMQTLSDTIIVVSGHELVAEVCDETRFDKSIEGALAKVRAFAGDGLFTSETQEPNWQKAHNILMPTFSQRAMKDYHAMMVDIAVQLVQKWARLNPNENVDVPEDMTRLTLDTIGLCGFNYRFNSFYRETPHPFITSMTRALDEAMHQLQRLDIEDKLMWRTKRQFQHDIQSMFSLVDNIIAERKSSENQEENDLLSRMLNVQDPETGEKLDDENIRFQIITFLIAGHETTSGLLSFAIYFLLKNPDKLKKAYEEVDRVLTDSTPTYQQVMKLKYIRMILNESLRLWPTAPAFSLYAKEDTVIGGKYPIKKGEDRISVLIPQLHRDKDAWGDNVEEFQPERFEELDKVPHHAYKPFGNGQRACIGMQFALHEATLVMGMLLQHFEFIDYEEYQLDVKQTLTLKPGDFKIRIVPRNQTISHTTVLAPTEEKLKNHEIKQQVQKTPSIIGADNLSLLVLYGSDTGVAEGIARELADTASLEGVQTEVAALNDRIGSLPKEGAVLIVTSSYNGKPPSNAGQFVQWLEELKGDELKGVQYAVFGCGDHNWASTYQRIPRYIDEQMAQKGATRFSTRGEADASGDFEEQLEQWKQRMWSDAMKVFGLELNKNMEKERSTLSLQFVSRLGGSPLARTYEAVYASILENRELQSSSSERSTRHIEISLPEGATYKEGDHLGVLPINSEKNVNRILKRFGLNGKDQVILSASGRSVNHIPLDSPVRLYDLLSYSVEVQEAATRAQIREMVTFTACPPHKKELESLLEDGVYQEQILKKRISMLDLLEKYEACEIRFEPFLELLPALKPRYYSISSSPLVAQDRLSITVGVVNAPAWSGEGTYEGVASNYLAQRHNKDEIICFIRTPQSNFQLPENPETPIIMVGPGTGIAPFRGFLQARRVQKQKGMNVGEAHLYFGCRHPEKDYLYRTELENDERDGLISLHTAFSRLEGQAKTYVQHVIKEDRIHLISLLDNGAHLYICGDGSKMAPDVEDTLCQAYQEIHEVSEQEARNWLDRLQEEGRYGKDVWAGI

>CYP102A4_ortholog(H9401_3072)Bacillus anthracis H9401

MLFERVFVMDKKVSAIPQPKTYGPLGNLPLIDKDKPTLSFIKLAEEYGPIFRMQTLSDTIIVVSGHELVAEVCDETRFDKSIEGALAKVRAFAGDGLFTSETQEPNWQKAHNILMPTFSQRAMKDYHAMMVDIAVQLVQKWARLNPNENVDVPEDMTRLTLDTIGLCGFNYRFNSFYRETPHPFITSMTRALDEAMHQLQRLDIEDKLMWRTKRQFQHDIQSMFSLVDNIIAERKSSENQEENDLLSRMLNVQDPETGEKLDDENIRFQIITFLIAGHETTSGLLSFAIYFLLKNPDKLKKAYEEVDRVLTDSTPTYQQVMKLKYIRMILNESLRLWPTAPAFSLYAKEDTVIGGKYPIKKGEDRISVLIPQLHRDKDAWGDNVEEFQPERFEELDKVPHHAYKPFGNGQRACIGMQFALHEATLVMGMLLQHFEFIDYEEYQLDVKQTLTLKPGDFKIRIVPRNQTISHTTVLAPTEEKLKNHEIKQQVQKTPSIIGADNLSLLVLYGSDTGVAEGIARELADTASLEGVQTEVAALNDRIGSLPKEGAVLIVTSSYNGKPPSNAGQFVQWLEELKGDELKGVQYAVFGCGDHNWASTYQRIPRYIDEQMAQKGATRFSTRGEADASGDFEEQLEQWKQRMWSDAMKVFGLELNKNMEKERSTLSLQFVSRLGGSPLARTYEAVYASILENRELQSSSSERSTRHIEISLPEGATYKEGDHLGVLPINSEKNVNRILKRFGLNGKDQVILSASGRSVNHIPLDSPVRLYDLLSYSVEVQEAATRAQIREMVTFTACPPHKKELESLLEDGVYQEQILKKRISMLDLLEKYEACEIRFEPFLELLPALKPRYYSISSSPLVAQDRLSITVGVVNAPAWSGEGTYEGVASNYLAQRHNKDEIICFIRTPQSNFQLPENPETPIIMVGPGTGIAPFRGFLQARRVQKQKGMNVGEAHLYFGCRHPEKDYLYRTELENDERDGLISLHTAFSRLEGQAKTYVQHVIKEDRIHLISLLDNGAHLYICGDGSKMAPDVEDTLCQAYQEIHEVSEQEARNWLDRLQEEGRYGKDVWAGI

>CYP102A4_ortholog(BCAH820_3229)Bacillus cereus AH820

MDKKVSAIPQPKTYGPLGNLPLIDKDKPTLSFIKLAEEYGPIFQIQTLSDTIIVVSGHELVAEVCDETRFDKSIEGALAKVRAFAGDGLFTSETQEPNWQKAHNILMPTFSQRAMKDYHAMMVDIAVQLVQKWARLNPNENVDVPEDMTRLTLDTIGLCGFNYRFNSFYRETPHPFITSMTRALDEAMHQLQRLDIEDKLMWRTKRQFQHDIQSMFSLVDNIIAERKSSEKQEENDLLSRMLNVQDPETGEKLDDENIRFQIITFLIAGHETTSGLLSFAIYFLLKNPDKLKKAYEEVDRVLTDPTPTYQQVMKLKYIRMILNESLRLWPTAPAFSLYAKEDTVIGGKYPIKKGKDRISVLIPQLHRDKDAWGDNVEEFQPERFEDLDKVPHHAYKPFGNGQRACIGMQFALHEATLVMGMLLQHFEFIDYEDYQLDVKQTLTLKPGDFKIRIVPRNQTISHTTVLAPTEEKLKKHEIKQQVQKTPSIIGADNLSLLVLYGSDTGVAEGIARELADTASLEGVQTEVVALNDRIGSLPKEGAVLIVTSSYNGKPPSNAGQFVQWLEELKPDELKGVQYAVFGCGDHNWASTYQRIPRYIDEQMAQKGATRFSTRGEADASGDFEEQLEQWKQRMWSDAMKAFGLELNKNMEKERSTLSLQFVSRLGGSPLARTYEAVYASILENRELQSSSSERSTRHIEISLPEGATYKEGDHLGVLPINSEKNVNRILKRFGLNGKDQVILSASGRSVNHIPLDSPVRLYDLLSYSVEVQEAATRAQIREMVTFTACPPHKKELESLLEDGVYQEQILKKRISMLDLLEKYEACEIRFERFLELLPALKPRYYSISSSPLVAQDRLSITVGVVNAPAWSGEGTYEGVASNYLAQRHNKDEIICFIRTPQSNFQLPENPETPIIMVGPGTGIAPFRGFLQARRVQKQKGMNVGEAHLYFGCRHPEKDYLYRTELENDERDGLISLHTAFSRLEGQAKTYVQHVIKEDRIHLISLLDNGAHLYICGDGSKMAPDVEDTLCQAYQEIHEVSEQEARNWLDRLQEEGRYGKDVWAGI

>CYP102A5_ortholog(BC3211)Bacillus cereus ATCC 14579

MEKKVSAIPQPKTYGPLGNLPLIDKDKPTLSFIKIAEEYGPIFQIQTLSDTIIVVSGHELVAEVCDETRFDKSIEGALAKVRAFAGDGLFTSETHEPNWKKAHNILMPTFSQRAMKDYHAMMVDIAVQLVQKWARLNPNENVDVPEDMTRLTLDTIGLCGFNYRFNSFYRETPHPFITSMTRALDEAMHQLQRLDIEDKLMWRTKRQFQHDIQSMFSLVDNIIAERKSSGDQEENDLLSRMLNVPDPETGEKLDDENIRFQIITFLIAGHETTSGLLSFAIYFLLKNPDKLKKAYEEVDRVLTDPTPTYQQVMKLKYMRMILNESLRLWPTAPAFSLYAKEDTVIGGKYPIKKGEDRISVLIPQLHRDKDAWGDNVEEFQPERFEELDKVPHHAYKPFGNGQRACIGMQFALHEATLVMGMLLQHFELIDYQNYQLDVKQTLTLKPGDFKIRILPRKQTISHPTVLAPTEDKLKNDEIKQHVQKTPSIIGADNLSLLVLYGSDTGVAEGIARELADTASLEGVQTEVVALNDRIGSLPKEGAVLIVTSSYNGKPPSNAGQFVQWLEELKPDELKGVQYAVFGCGDHNWASTYQRIPRYIDEQMAQKGATRFSKRGEADASGDFEEQLEQWKQNMWSDAMKAFGLELNKNMEKERSTLSLQFVSRLGGSPLARTYEAVYASILENRELQSSSSDRSTRHIEVSLPEGATYKEGDHLGVLPVNSEKNINRILKRFGLNGKDQVILSASGRSINHIPLDSPVSLLALLSYSVEVQEAATRAQIREMVTFTACPPHKKELEALLEEGVYHEQILKKRISMLDLLEKYEACEIRFERFLELLPALKPRYYSISSSPLVAHNRLSITVGVVNAPAWSGEGTYEGVASNYLAQRHNKDEIICFIRTPQSNFELPKDPETPIIMVGPGTGIAPFRGFLQARRVQKQKGMNLGQAHLYFGCRHPEKDYLYRTELENDERDGLISLHTAFSRLEGHPKTYVQHLIKQDRINLISLLDNGAHLYICGDGSKMAPDVEDTLCQAYQEIHEVSEQEARNWLDRVQDEGRYGKDVWAGI

>CYP102A5_ortholog(BMB171_C2898)Bacillus thuringiensis BMB171

MEKKVSAIPQPKTYGPLGNLPLIDKDKPTLSFIKIAEEYGPIFQIQTLSDTIIVVSGHELVAEVCDETRFDKSIEGALAKVRAFAGDGLFTSETHEPNWKKAHNILMPTFSQRAMKDYHAMMVDIAVQLVQKWARLNPNENVDVPEDMTRLTLDTIGLCGFNYRFNSFYRETPHPFITSMTRALDEAMHQLQRLDIEDKLMWRTKRQFQHDIQSMFSLVDNIIAERKSSGDQEENDLLSRMLNVKDPETGEKLDDENIRFQIITFLIAGHETTSGLLSFAIYFLLKNPDKLKKAYEEVDRVLTDPTPTYQQVMKLKYIRMILNESLRLWPTAPAFSLYAKEDTVIGGKYPIKKGEDRISVLIPQLHRDKDAWGDNVEEFQPERFEELDKVPHHAYKPFGNGQRACIGMQFALHEATLVMGMLLQHFEIIDYQNYQLDVKQTLTLKPGDFKIRILPRKQTISHPTVLAPTEDKLKNDEIKQHVQKTPSIIGADNLSLLVLYGSDTGVAEGIARELADTASLEGVQTEVVALNDRIGSLPKEGAVLIVTSSYNGKPPSNAGQFVQWLEELKPDELKGVQYAVFGCGDHNWASTYQRIPRYIDEQMAQKGATRFSKRGEADASGDFEEQLEQWKQNMWSDAMKAFGLELNKNMEKERSTLSLQFVSRLGGSPLARTYEAVYASILENRELQSSSSDRSTRHIEVSLPEGATYKEGDHLGVLPVNSEKNINRILKRFGLNGKDQVILSASGRSINHIPLDSPVSLLDLLSYSVEVQEAATRAQIREMVTFTACPPHKKELEALLEEGVYHEQILKKRISMLDLLEKYEACEIRFERFLELLPALKPRYYSISSSPLVAHNRLSITVGVVNAPAWSGEGTYEGVASNYLAQRHNKDEIICFIRTPQSNFELPKNPETPIIMVGPGTGIAPFRGFLQARRVQKQKGMNLGQAHLYFGCRHPEKDYLYRTELENDERDGLISLHTAFSRLEGHPKTYVQHLIKQDRINLISLLDNGAHLYICGDGSKMAPDVEDTLCQAYQEIHEVSEQEARNWLDRVQDEGRYGKDVWAGI

>CYP102A5_ortholog(BCB4264_A3231)Bacillus cereus B4264

MVAEVCDETRFDKSIEGALAKVRAFAGDGLFTSETHEPNWKKAHNILMPTFSQRAMKDYHAMMVDIAVQLVQKWARLNPNENVDVPEDMTRLTLDTIGLCGFNYRFNSFYRETPHPFITSMTRALDEAMHQLQRLDIEDKLMWRTKRQFQHDIQSMFSLVDNIIAERKSSGDQEENDLLSRMLNVKDPETGEKLDDENIRFQIITFLIAGHETTSGLLSFAIYFLLKNPDKLKKAYEEVDRVLTDPTPTYEQVMKLKYIRMILNESLRLWPTAPAFSLYAKEDTVIGGKYPIKKGEDRISVLIPQLHRDKDAWGDNVEEFQPERFEELDKVPHHAYKPFGNGQRACIGMQFALHEATLVMGMLLQHFELIDYQNYQLDVKQTLTLKPGDFKIRILPRKQTISHPTVLAPTEDKLKNDEIKQHVQKTPSIIGADNLSLLVLYGSDTGVAEGIARELADTASLEGVRTEVVALNDQIGSLPKEGAVLIVTSSYNGKPPSNAGQFVQWLEELKPDELKGVQYAVFGCGDHNWASTYQRIPRYIDEQMAQKGATRFSKRGEADASGDFEEQLEQWKQSMWSDAMKAFGLELNKNMEKERSTLSLQFVSRLGGSPLARTYEAVYASILENRELQSSSSDRSTRHIEVSLPEGATYKEGDHLGVLPVNSEKNINRILKRFGLNGKDQVILSASGRSINHIPLDSPVSLLDLLSYSVEVQEAATRAQIREMVTFTACPPHKKELEALLEEGVYHEQILKKRISMLDLLEKYEACEIRFERFLELLPALKPRYYSISSSPLVAHNRLSITVGVVNAPAWSGEGTYEGVASNYLAQRHNKDEIICFIRTPQSNFELPKDPETPIIMVGPGTGIAPFRGFLQARRVQKQKGMNLGQAHLYFGCRHPEKDYLYRTELENDERDGLISLHTAFSRLEGHPKTYVQHLIKQDRINLISLLDNGAHLYICGDGSKMAPDVEDTLCQAYQEIHEVSEQEARNWLDRVQDEGRYGKDVWAGI

>CYP102A5_ortholog(CY96_14915)Bacillus bombysepticus

MEKKVSAIPQPKTYGPLGNLPLIDKDKPTLSFIKIAEEYGPIFQIQTLSDTIIVVSGHELVAEVCDETRFDKSIEGALAKVRAFAGDGLFTSETHEPNWKKAHNILMPTFSQRAMKDYHAMMVDIAVQLVQKWARLNPNENVDVPEDMTRLTLDTIGLCGFNYRFNSFYRETPHPFITSMTRALDEAMHQLQRLDIEDKLIWRTKRQFQHDIQSMFSLVDNIIAERKSSGNQEENDLLSRMLNVPDPETGEKLDDENIRFQIITFLIAGHETTSGLLSFAIYFLLKNPDKLKKAYEEVDRVLTDSTPTYQQVMKLKYIRMILNESLRLWPTAPAFSLYAKEDTVIGGKYPIKKGEDRISVLIPQLHRDKDAWGDNVEEFQPERFEELDKVPNHAYKPFGNGQRACIGMQFALHEATLVMGMLLQHFELIDYQNYQLEVKQTLTLKPGDFKIRILPRKQTISHPTVLAPTEDKLKNDEIKQHVQKTPSIIGADNLSLLVLYGSDTGVAEGIARELADTASLEGVQTEVVALNDRIGSLPKEGAVLIVTSSYNGKPPSNAGQFVQWLEELKPDELKGVQYAVFGCGDHNWASTYQRIPRYIDEQMAQKGATRFSKRGEADASGDFEEQLEQWKQSMWSDAMKAFGLELNKNMEKERSTLSLQFVSRLGGSPLARTYEAVYASILENRELQSSSSDRSTRHIEVSLPEGATYKEGDHLGVLPVNSEKNINRILKRFGLNGKDQVILSASGRSINHIPLDSPVSLLDLLSYSVEVQEAATRAQIREMVTFTACPPHKKELEALLEEGVYHEQILKKRISMLDLLEKYEACEIRFERFLELLPALKPRYYSISSSPLVAQNRLSITVGVVNAPAWSGEGTYEGVASNYLAQRHNKDEIICFIRTPQSNFELPKDPETPIIMVGPGTGIAPFRGFLQARRVQKQKGINLGEAHLYFGCRHPEKDYLYRTELENDERDGLISLHTAFSRLEGHPKTYVQHLIKQDRINLISLLDNGAHLYICGDGSKMAPDVEDTLCQAYQEIHEVSEQEARNWLDRVQDEGRYGKDVWAGI

>CYP102A5_ortholog(CT43_CH3164)Bacillus thuringiensis serovar chinensis CT-43

MEKKVSAIPQPKTYGPLGNLPLIDKDKPTLSFIKIAEEYGPIFQIQTLSDTIIVVSGHELVAEVCDETRFDKSIEGALAKVRAFAGDGLFTSETHEPNWKKAHNILMPTFSQRAMKDYHAMMVDIAVQLVQKWARLNPNENVDVPGDMTRLTLDTIGLCGFNYRFNSFYRETPHPFITSMTRALDEAMHQLQRLDIEDKLMWRTKRQFQHDIQSMFSLVDNIIAERKSNGNQEENDLLSRMLNVPDPETGEKLDDENIRFQIITFLIAGHETTSGLLSFAIYFLLKNPDKLKKAYEEVDRVLTDPTPTYQQVMKLKYIRMILNESLRLWPTAPAFSLYAKEDTVIGGKYPIKKGEDRISVLIPQLHRDKDAWGDNVEEFQPERFEELDKVPHHAYKPFGNGQRACIGMQFALHEATLVMGMLLQHFELIDYQNYQLDVKQTLTLKPGDFKIRILPRKQTISHPTVLTPTEDKLKNDEIKQHVQKPPSIIGADNLSLLVLYGSDTGVAEGIARELADTASLEGVRTEVVALNDRIGSLPKEGAVLIVTSSYNGKPPSNAGQFVQWLEELKPDELKGVQYAVFGCGDHNWASTYQRIPRYIDEQMAQKGATRFSKRGEADASGDFEEQLEQWKQNMWSDAMKAFGLELNKNIEKERSTLSLQFVSRLGGSPLARTYEAVYASILENRELQSPSSDRSTRHIEVSLPEGATYKEGDHLGVLPVNSEKNINRILKRFGLNGKDQVILSASGRSINHIPLDSPVSLLDLLSYSVEVQEAATRAQIREMVTFTACPPHKKELEALLEEGVYHEQILKKRISMLDLLEKYEACEIRFERFLELLPALKPRYYSISSSPLVAHNRLSITVGVVNAPAWSGEGTYEGVASNYLAQRHNKDEIICFIRTPQSNFELPKDPETPIIMVGPGTGIAPFRGFLQARRVQKQKGINLGQAHLYFGCRHPEKDYLYRTELENDERDGLISLHTAFSRLEGHPKTYVQHLIKQDRINLISLLDNGAHLYICGDGSRMAPDVEDTLCQAYQEIHEVSEQEARNWLDRVQDEGRYGKDVWAGI

>CYP102A5_ortholog(BTB_c32980)Bacillus thuringiensis Bt407

MEKKVSAIPQPKTYGPLGNLPLIDKDKPTLSFIKIAEEYGPIFQIQTLSDTIIVVSGHELVAEVCDETRFDKSIEGALAKVRAFAGDGLFTSETHEPNWKKAHNILMPTFSQRAMKDYHAMMVDIAVQLVQKWARLNPNENVDVPGDMTRLTLDTIGLCGFNYRFNSFYRETPHPFITSMTRALDEAMHQLQRLDIEDKLMWRTKRQFQHDIQSMFSLVDNIIAERKSNGNQEENDLLSRMLNVPDPETGEKLDDENIRFQIITFLIAGHETTSGLLSFAIYFLLKNPDKLKKAYEEVDRVLTDPTPTYQQVMKLKYIRMILNESLRLWPTAPAFSLYAKEDTVIGGKYPIKKGEDRISVLIPQLHRDKDAWGDNVEEFQPERFEELDKVPHHAYKPFGNGQRACIGMQFALHEATLVMGMLLQHFELIDYQNYQLDVKQTLTLKPGDFKIRILPRKQTISHPTVLTPTEDKLKNDEIKQHVQKPPSIIGADNLSLLVLYGSDTGVAEGIARELADTASLEGVRTEVVALNDRIGSLPKEGAVLIVTSSYNGKPPSNAGQFVQWLEELKPDELKGVQYAVFGCGDHNWASTYQRIPRYIDEQMAQKGATRFSKRGEADASGDFEEQLEQWKQNMWSDAMKAFGLELNKNIEKERSTLSLQFVSRLGGSPLARTYEAVYASILENRELQSPSSDRSTRHIEVSLPEGATYKEGDHLGVLPVNSEKNINRILKRFGLNGKDQVILSASGRSINHIPLDSPVSLLDLLSYSVEVQEAATRAQIREMVTFTACPPHKKELEALLEEGVYHEQILKKRISMLDLLEKYEACEIRFERFLELLPALKPRYYSISSSPLVAHNRLSITVGVVNAPAWSGEGTYEGVASNYLAQRHNKDEIICFIRTPQSNFELPKDPETPIIMVGPGTGIAPFRGFLQARRVQKQKGINLGQAHLYFGCRHPEKDYLYRTELENDERDGLISLHTAFSRLEGHPKTYVQHLIKQDRINLISLLDNGAHLYICGDGSRMAPDVEDTLCQAYQEIHEVSEQEARNWLDRVQDEGRYGKDVWAGI

>CYP102A5_ortholog(BTK_13800)Bacillus thuringiensis serovar kurstaki HD-1

MEKKVSAIPQPKTYGPLGNLPLIDKDKPTLSFIKIAEEYGPIFQIQTLSDTIIVVSGHELVAEVCDETRFDKSIEGALAKVRAFAGDGLFTSETHEPNWKKAHNILMPTFSQRAMKDYHAMMVDIAVQLVQKWARLNPNENVDVPEDMTRLTLDTIGLCGFNYRFNSFYRETPHPFITSMTRALDEAMHQLQRLDIEDKLMWRTKRQFQHDIQSMFSLVDNIIAERKSSGDQEENDLLSRMLNVQDPETGEKLDDENIRFQIITFLIAGHETTSGLLSFAIYFLLKNPDKLKKAYEEVDRVLTDPTPTYQQVMKLKYIRMILNESLRLWPTAPAFSLYAKEDTVIGGKYPIKKGEDRISVLIPQLHRDKDAWGDNVEEFQPERFEELDKVPHHAYKPFGNGQRACIGMQFALHEATLVMGMLLQHFELIDYQNYQLDVKQTLTLKPGDFKIRILPRKQTISHPTALAPTEDKLKNDEIKQHVQKTPSIIGADNLSLLVLYGSDTGVAEGIARELADTASLEGVRTEVVALNDRIGSLPKEGAVLIVTSSYNGKPPSNAGQFVQWLEELKTDELKGVQYAVFGCGDHNWASTYQRIPRYIDEQMAQKGATRFSKRGEADASGDFEEQLEQWKQSMWSDAMKAFGLELNKNMEKERSTLSLQFVSRLGGSPLARTYEAVYASILENRELQSSSSDRSTRHIEVSLPEGATYKEGDHLGVLPVNSEKNINRILRRFGLNGKDQVILSASGRSINHIPLDSPVSLLDLLSYSVEVQEAATRAQIREMVTFTACPPHKKELEALLEEGVYHEQILKKRISMLDLLEKYEACEIRFERFLELLPALKPRYYSISSSPLVVQNRLSITVGVVNAPAWSGEGTYEGVASNYLAQRHNKDEIICFIRTPQSNFELPKDPETPIIMVGPGTGIAPFRGFLQARRVQKQKGINLGEAHLYFGCRHPEKDYLYRTELENDERDGLISLHTAFSRLEGHPKTYVQHLIKQDRINLISLLDNGAHLYICGDGSKMAPDVEDTLCQAYQEIHEVSEQEARNWLDRVQDEGRYGKDVWAGI

>CYP102A5_ortholog(BTK_16685)Bacillus thuringiensis serovar kurstaki HD-1

MEKKVSAIPQPKTYGPLGNLPLIDKDKPTLSFIKIAEEYGPIFQIQTLSDTIIVVSGHELVAEVCDETRFDKSIEGALAKVRAFAGDGLFTSETHEPNWKKAHNILMPTFSQRAMKDYHAMMVDIAVQLVQKWARLNPNENVDVPEDMTRLTLDTIGLCGFNYRFNSFYRETPHPFITSMTRALDEAMHQLQRLDIEDKLMWRTKRQFQHDIQSMFSLVDNIIAERKSSGDQEENDLLSRMLNVQDPETGEKLDDENIRFQIITFLIAGHETTSGLLSFAIYFLLKNPDKLKKAYEEVDRVLTDPTPTYQQVMKLKYIRMILNESLRLWPTAPAFSLYAKEDTVIGGKYPIKKGEDRISVLIPQLHRDKDAWGDNVEEFQPERFEELDKVPHHAYKPFGNGQRACIGMQFALHEATLVMGMLLQHFELIDYQNYQLDVKQTLTLKPGDFKIRILPRKQTISHPTALAPTEDKLKNDEIKQHVQKTPSIIGADNLSLLVLYGSDTGVAEGIARELADTASLEGVRTEVVALNDRIGSLPKEGAVLIVTSSYNGKPPSNAGQFVQWLEELKTDELKGVQYAVFGCGDHNWASTYQRIPRYIDEQMAQKGATRFSKRGEADASGDFEEQLEQWKQSMWSDAMKAFGLELNKNMEKERSTLSLQFVSRLGGSPLARTYEAVYASILENRELQSSSSDRSTRHIEVSLPEGATYKEGDHLGVLPVNSEKNINRILRRFGLNGKDQVILSASGRSINHIPLDSPVSLLDLLSYSVEVQEAATRAQIREMVTFTACPPHKKELEALLEEGVYHEQILKKRISMLDLLEKYEACEIRFERFLELLPALKPRYYSISSSPLVVQNRLSITVGVVNAPAWSGEGTYEGVASNYLAQRHNKDEIICFIRTPQSNFELPKDPETPIIMVGPGTGIAPFRGFLQARRVQKQKGINLGEAHLYFGCRHPEKDYLYRTELENDERDGLISLHTAFSRLEGHPKTYVQHLIKQDRINLISLLDNGAHLYICGDGSKMAPDVEDTLCQAYQEIHEVSEQEARNWLDRVQDEGRYGKDVWAGI

>CYP102A5_ortholog(YBT1520_13665)Bacillus thuringiensis serovar kurstaki YBT-1520

MEKKVSAIPQPKTYGPLGNLPLIDKDKPTLSFIKIAEEYGPIFQIQTLSDTIIVVSGHELVAEVCDETRFDKSIEGALAKVRAFAGDGLFTSETHEPNWKKAHNILMPTFSQRAMKDYHAMMVDIAVQLVQKWARLNPNENVDVPEDMTRLTLDTIGLCGFNYRFNSFYRETPHPFITSMTRALDEAMHQLQRLDIEDKLMWRTKRQFQHDIQSMFSLVDNIIAERKSSGDQEENDLLSRMLNVQDPETGEKLDDENIRFQIITFLIAGHETTSGLLSFAIYFLLKNPDKLKKAYEEVDRVLTDPTPTYQQVMKLKYIRMILNESLRLWPTAPAFSLYAKEDTVIGGKYPIKKGEDRISVLIPQLHRDKDAWGDNVEEFQPERFEELDKVPHHAYKPFGNGQRACIGMQFALHEATLVMGMLLQHFELIDYQNYQLDVKQTLTLKPGDFKIRILPRKQTISHPTALAPTEDKLKNDEIKQHVQKTPSIIGADNLSLLVLYGSDTGVAEGIARELADTASLEGVRTEVVALNDRIGSLPKEGAVLIVTSSYNGKPPSNAGQFVQWLEELKTDELKGVQYAVFGCGDHNWASTYQRIPRYIDEQMAQKGATRFSKRGEADASGDFEEQLEQWKQSMWSDAMKAFGLELNKNMEKERSTLSLQFVSRLGGSPLARTYEAVYASILENRELQSSSSDRSTRHIEVSLPEGATYKEGDHLGVLPVNSEKNINRILRRFGLNGKDQVILSASGRSINHIPLDSPVSLLDLLSYSVEVQEAATRAQIREMVTFTACPPHKKELEALLEEGVYHEQILKKRISMLDLLEKYEACEIRFERFLELLPALKPRYYSISSSPLVVQNRLSITVGVVNAPAWSGEGTYEGVASNYLAQRHNKDEIICFIRTPQSNFELPKDPETPIIMVGPGTGIAPFRGFLQARRVQKQKGINLGEAHLYFGCRHPEKDYLYRTELENDERDGLISLHTAFSRLEGHPKTYVQHLIKQDRINLISLLDNGAHLYICGDGSKMAPDVEDTLCQAYQEIHEVSEQEARNWLDRVQDEGRYGKDVWAGI

>CYP102A5_ortholog(H175_ch3219)Bacillus thuringiensis serovar thuringiensis IS5056

MEKKVSAIPQPKTYGPLGNLPLIDKDKPTLSFIKIAEEYGPIFQIQTLSDTIIVVSGHELVAEVCDETRFDKSIEGALAKVRAFAGDGLFTSETHEPNWKKAHNILMPTFSQRAMKDYHAMMVDIAVQLVQKWARLNPNENVDVPGDMTRLTLDTIGLCGFNYRFNSFYRETPHPFITSMTRALDEAMHQLQRLDIEDKLMWRTKRQFQHDIQSMFSLVDNIIAERKSNGNQEENDLLSRMLNVPDPETGEKLDDENIRFQIITFLIAGHETTSGLLSFAIYFLLKNPDKLKKAYEEVDRVLTDPTPTYQQVMKLKYIRMILNESLRLWPTAPAFSLYAKEDTVIGGKYPIKKGEDRISVLIPQLHRDKDAWGDNVEEFQPERFEELDKVPHHAYKPFGNGQRACIGMQFALHEATLVMGMLLQHFELIDYQNYQLDVKQTLTLKPGDFKIRILPRKQTISHPTVLTPTEDKLKNDEIKQHVQKPPSIIGADNLSLLVLYGSDTGVAEGIARELADTASLEGVRTEVVALNDRIGSLPKEGAVLIVTSSYNGKPPSNAGQFVQWLEELKPDELKGVQYAVFGCGDHNWASTYQRIPRYIDEQMAQKGATRFSKRGEADASGDFEEQLEQWKQNMWSDAMKAFGLELNKNIEKERSTLSLQFVSRLGGSPLARTYEAVYASILENRELQSPSSDRSTRHIEVSLPEGATYKEGDHLGVLPVNSEKNINRILKRFGLNGKDQVILSASGRSINHIPLDSPVSLLDLLSYSVEVQEAATRAQIREMVTFTACPPHKKELEALLEEGVYHEQILKKRISMLDLLEKYEACEIRFERFLELLPALKPRYYSISSSPLVAHNRLSITVGVVNAPAWSGEGTYEGVASNYLAQRHNKDEIICFIRTPQSNFELPKDPETPIIMVGPGTGIAPFRGFLQARRVQKQKGINLGQAHLYFGCRHPEKDYLYRTELENDERDGLISLHTAFSRLEGHPKTYVQHLIKQDRINLISLLDNGAHLYICGDGSRMAPDVEDTLCQAYQEIHEVSEQEARNWLDRVQDEGRYGKDVWAGI

>CYP102A5_ortholog(AQ980_17175)Bacillus thuringiensis YWC2-8

MEKKVSAIPQPKTYGPLGNLPLIDKDKPTLSFIKIAEEYGPIFQIQTLSDTIIVVSGHELVAEVCDETRFDKSIEGALAKVRAFAGDGLFTSETHEPNWKKAHNILMPTFSQRAMKDYHAMMVDIAVQLVQKWARLNPNENVDVPEDMTRLTLDTIGLCGFNYRFNSFYRETPHPFITSMTRALDEAMHQLQRLDIEDKLMWRTKRQFQHDIQSMFSLVDNIIAERKSSGDQEENDLLSRMLNVQDPETGEKLDDENIRFQIITFLIAGHETTSGLLSFAIYFLLKNPDKLKKAYEEVDRVLTDPTPTYQQVMKLKYIRMILNESLRLWPTAPAFSLYAKEDTVIGGKYPIKKGEDRISVLIPQLHRDKDAWGDNVEEFQPERFEELDKVPHHAYKPFGNGQRACIGMQFALHEATLVMGMLLQHFELIDYQNYQLDVKQTLTLKPGDFKIRILPRKQTISHPTALAPTEDKLKNDEIKQHVQKTPSIIGADNLSLLVLYGSDTGVAEGIARELADTASLEGVRTEVVALNDRIGSLPKEGAVLIVTSSYNGKPPSNAGQFVQWLEELKTDELKGVQYAVFGCGDHNWASTYQRIPRYIDEQMAQKGATRFSKRGEADASGDFEEQLEQWKQSMWSDAMKAFGLELNKNMEKERSTLSLQFVSRLGGSPLARTYEAVYASILENRELQSSSSDRSTRHIEVSLPEGATYKEGDHLGVLPVNSEKNINRILRRFGLNGKDQVILSASGRSINHIPLDSPVSLLDLLSYSVEVQEAATRAQIREMVTFTACPPHKKELEALLEEGVYHEQILKKRISMLDLLEKYEACEIRFERFLELLPALKPRYYSISSSPLVVQNRLSITVGVVNAPAWSGEGTYEGVASNYLAQRHNKDEIICFIRTPQSNFELPKDPETPIIMVGPGTGIAPFRGFLQARRVQKQKGINLGEAHLYFGCRHPEKDYLYRTELENDERDGLISLHTAFSRLEGHPKTYVQHLIKQDRINLISLLDNGAHLYICGDGSKMAPDVEDTLCQAYQEIHEVSEQEARNWLDRVQDEGRYGKDVWAGI

>CYP102A5_ortholog(HD73_2783)Bacillus thuringiensis serovar kurstaki HD73

MEKKVSAIPQPKTYGPLGNLPLIDKDKPTLSFIKIAEEYGPIFQIQTLSDTIIVVSGHELVAEVCDETRFDKSIEGALAKVRAFAGDGLFTSETHEPNWKKAHNILMPTFSQRAMKDYHAMMVDIAVQLVQKWARLNPNENVDVPEDMTRLTLDTIGLCGFNYRFNSFYRETPHPFITSMTRALDEAMHQLQRLDIEDKLMWRTKRQFQHDIQSMFSLVDNIIAERKSSGDQEENDLLSRMLNVQDPETGEKLDDENIRFQIITFLIAGHETTSGLLSFAIYFLLKNPDKLKKAYEEVDRVLTDPTPTYQQVMKLKYIRMILNESLRLWPTAPAFSLYAKEDTVIGGKYPIKKGEDRISVLIPQLHRDKDAWGDNVEEFQPERFEELDKVPHHAYKPFGNGQRACIGMQFALHEATLVMGMLLQHFELIDYQNYQLDVKQTLTLKPGDFKIRILPRKQTISHPTALAPTEDKLKNDEIKQHVQKTPSIIGADNLSLLVLYGSDTGVAEGIARELADTASLEGVRTEVVALNDRIGSLPKEGAVLIVTSSYNGKPPSNAGQFVQWLEELKTDELKGVQYAVFGCGDHNWASTYQRIPRYIDEQMAQKGATRFSKRGEADASGDFEEQLEQWKQSMWSDAMKAFGLELNKNMEKERSTLSLQFVSRLGGSPLARTYEAVYASILENRELQSSSSDRSTRHIEVSLPEGATYKEGDHLGVLPVNSEKNINRILRRFGLNGKDQVILSASGRSINHIPLDSPVSLLDLLSYSVEVQEAATRAQIREMVTFTACPPHKKELEALLEEGVYHEQILKKRISMLDLLEKYEACEIRFERFLELLPALKPRYYSISSSPLVVQNRLSITVGVVNAPAWSGEGTYEGVASNYLAQRHNKDEIICFIRTPQSNFELPKDPETPIIMVGPGTGIAPFRGFLQARRVQKQKGINLGEAHLYFGCRHPEKDYLYRTELENDERDGLISLHTAFSRLEGHPKTYVQHLIKQDRINLISLLDNGAHLYICGDGSKMAPDVEDTLCQAYQEIHEVSEQEARNWLDRVQDEGRYGKDVWAGI

>CYP102A5_ortholog(YBT1518_17610)Bacillus thuringiensis YBT-1518

MEKKVSAIPQPKTYGPLGNLPLIDKDKPTLSFIKIAEEYGPIFQIQTLSDTIIVVSGHELVAEVCDETRFDKSIEGALAKVRAFAGDGLFTSETHEPNWKKAHNILMPTFSQRAMKDYHAMMVDLAVQLVQKWARLNPNENVDVPEDMTRLTLDTIGLCGFNYRFNSFYRETPHPFITSMSRALDEAMHQLQRLDIEDKLMWRTKRQFQHDIQSMFSLVDNIIAERKSSGNQEENDLLSRMLNVQDPETGEKLDDENIRFQIITFLIAGHETTSGLLSFSIYFLLKNPDKLKKAYEEVDRVLTDPTPTYQQVMKLKYIRMILNESLRLWPTAPAFSLYAKEDTVIGGKYPIKKGEDRISVLIPQLHRDKDAWGDNVEEFQPERFEELDKVPHHAYKPFGNGQRACIGMQFALHEATLVMGMLLQHFELIDYQNYQLDVKQTLTLKPGDFKIRILPRKQTISHPTVLAPTEDKLKNDEIKQHVQKTPSIIGADNLSLLVLYGSDTGVAEGIARELADTASLEGVRTEVVALNDRIGSLPKEGAVLIVTSSYNGKPPSNAGQFVQWLEELKPDELKGVQYAVFGCGDHNWASTYQRIPRYIDEQMAQKGATRFSKRGEADASGDFEEQLEQWKQSMWSDAMKAFGLELNKNMEKERSTLSLQFVSRLGGSPLARTYEAVYASILENRELQSSSSDRSTRHIEVSLPEGATYQEGDHLGVLPINSEKNVNRILKRFGLNGKDQVILSASGRSINHIPLDSPVSLLDLLSYSVEVQEAATRAQIREMVTFTACPPHKKELEALLEEGVYHEQILKKRISMLDLLEKYEACEIRFERFLELLPALKPRYYSISSSPLVAQNRLSITVGVVNAPAWSGEGTYEGVASNYLAQRHNKDEIICFIRTPQSNFELPKDPETPIIMVGPGTGIAPFRGFLQARRVQKQKGINLGQAHLYFGCRHPEKDYLYRTELENDERDGLISLHTAFSRLEGHPKTYVQHLIKQDRINLISLLDNGAHLYICGDGSKMAPDVEDTLCQAYQEIHEVSEQEARNWLDRVQDEGRYGKDVWAGI

>CYP102A5_ortholog(BCG9842_B2016)Bacillus cereus G9842

MEKKVSAIPQPKTYGPLGNLPLIDKDKPTLSFIKLAEEYGPIFRIQTLSDAIIVVSGHELVAEVCDETRFDKSIEGALAKVRAFAGDGLFTSETHEPNWKKAHNILMPTFSQRAMKDYHAMMVDLAVQLVQKWARLNPNEDVDVPEDMTRLTLDTIGLCGFNYRFNSFYRETPHPFITSMSRALDEAMHQLQRLDIEDKLMWRTKRQFQHDIQSMFSLVDNIIAERKSSGDQEENDLLSRMLNVQDPETGEKLDDENIRFQIITFLIAGHETTSGLLSFAIYFLLKNPDKLKKAYEEVDRILTDPTPTYQQVMKLKYIRMILNESLRLWPTAPAFSLYAKEDTVIGGKYPIKKGEDRISVLIPQLHRDKDAWGDNVEEFQPERFEELDKVPHHAYKPFGNGQRACIGMQFALHEATLVMGMLLQHFELIDYQNYQLDVKQTLTLKPGDFKIRILPRKQTISHPTVLAPTEDKLKNHEIKQHVQKTPSIIGADNLSLLVLYGSDTGVAEGIARELADTASLEGVQTEVVALNDRIGSLPKEGAVLIVTSSYNGKPPSNAGQFVQWLEELKPDELKGVQYAVFGCGDHNWASTYQRIPRYIDEQMAQKGATRFSKRGEADASGDFEEQLEQWKQGMWSDAMKAFGLEFNKNMEKERSTLSLQFVSRLGGSPLARTYEAVYATILENRELQSSSSDRSTRHIEVSLPEGATYQEGDHLGVLPINSEKNVNRILKRFGLNGKDQVILSASGRSINHIPLDSPVSLLDLLSYSVEVQEAATRAQIREMVTFTACPPHKKELEALLEEGVYHEQILKKRISMLDLLEKYEACEIRFERFLELLPALKPRYYSISSSPLVAQNRLSITVGVVNAPAWSGEGTYEGVASNYLAQRHNKDEIICFIRTPQSNFELPKDPETPIIMVGPGTGVAPFRGFLQARRVQKQKGINLGQAHLYFGCRHPEKDYLYRTELENDERDGLISLHTAFSRLEGHPKTYVQHLIKQDSINLISLLDNGAHLYICGDGSKMAPDVEDTLCQAYQEIHEVSEQEARNWLDRVQDEGRYGKDVWAGI

>CYP102A5_ortholog(BTG_03315)Bacillus thuringiensis HD-771

MEKKVSAIPQPKTYGPLGNLPLIDKDKPTLSFIKLAEEYGPIFRIQTLSDAIIVVSGHELVAEVCDETRFDKSIEGALAKVRAFAGDGLFTSETHEPNWKKAHNILMPTFSQRAMKDYHAMMVDLAVQLVQKWARLNPNEDVDVPEDMTRLTLDTIGLCGFNYRFNSFYRETPHPFITSMSRALDEAMHQLQRLDIEDKLMWRTKRQFQHDIQSMFSLVDNIIAERKSSGDQEENDLLSRMLNVQDPETGEKLDDENIRFQIITFLIAGHETTSGLLSFAIYFLLKNPDKLKKAYEEVDRILTDPTPTYQQVMKLKYIRMILNESLRLWPTAPAFSLYAKEDTVIGGKYPIKKGEDRISVLIPQLHRDKDAWGDNVEEFQPERFEELDKVPHHAYKPFGNGQRACIGMQFALHEATLVMGMLLQHFELIDYQNYQLDVKQTLTLKPGDFKIRILPRKQTISHPTVLAPTEDKLKNHEIKQHVQKTPSIIGADNLSLLVLYGSDTGVAEGIARELADTASLEGVQTEVVALNDRIGSLPKEGAVLIVTSSYNGKPPSNAGQFVQWLEELKPDELKGVQYAVFGCGDHNWASTYQRIPRYIDEQMAQKGATRFSKRGEADAGGDFEEQLEQWKQGMWSDAMKAFGLEFNKNMEKERSTLSLQFVSRLGGSPLARTYEAVYATILENRELQSSSSDRSTRHIEVSLPEGATYQEGDHLGVLPINSEKNVNRILKRFGLNGKDQVILSASGRSINHIPLDSPVSLLDLLSYSVEVQEAATRAQIREMVTFTACPPHKKELEALLEEGVYHEQILKKRISMLDLLEKYEACEIRFERFLELLPALKPRYYSISSSPLVAQNRLSITVGVVNAPAWSGEGTYEGVASNYLAQRHNKDEIICFIRTPQSNFELPKDPETPIIMVGPGTGVAPFRGFLQARRVQKQKGINLGQAHLYFGCRHPEKDYLYRTELENDERDGLISLHTAFSRLEGHPKTYVQHLIKQDSINLISLLDNGAHLYICGDGSKMAPDVEDTLCQAYQEIHEVSEQEARNWLDRVQDEGRYGKDVWAGI

>CYP102A5_ortholog(BTF1_13525)Bacillus thuringiensis HD-789

MEKKVSAIPQPKTYGPLGNLPLIDKDKPTLSFIKLAEEYGPIFRIQTLSDAIIVVSGHELVAEVCDETRFDKSIEGALAKVRAFAGDGLFTSETHEPNWKKAHNILMPTFSQRAMKDYHAMMVDLAVQLVQKWARLNPNEDVDVPEDMTRLTLDTIGLCGFNYRFNSFYRETPHPFITSMSRALDEAMHQLQRLDIEDKLMWRTKRQFQHDIQSMFSLVDNIIAERKSSGDQEENDLLSRMLNVQDPETGEKLDDENIRFQIITFLIAGHETTSGLLSFAIYFLLKNPDKLKKAYEEVDRILTDPTPTYQQVMKLKYIRMILNESLRLWPTAPAFSLYAKEDTVIGGKYPIKKGEDRISVLIPQLHRDKDAWGDNVEEFQPERFEELDKVPHHAYKPFGNGQRACIGMQFALHEATLVMGMLLQHFELIDYQNYQLDVKQTLTLKPGDFKIRILPRKQTISHPTVLAPTEDKLKNHEIKQHVQKTPSIIGADNLSLLVLYGSDTGVAEGIARELADTASLEGVQTEVVALNDRIGSLPKEGAVLIVTSSYNGKPPSNAGQFVQWLEELKPDELKGVQYAVFGCGDHNWASTYQRIPRYIDEQMAQKGATRFSKRGEADASGDFEEQLEQWKQGMWSDAMKAFGLEFNKNMEKERSTLSLQFVSRLGGSPLARTYEAVYATILENRELQSSSSDRSTRHIEVSLPEGATYQEGDHLGVLPINSEKNVNRILKRFGLNGKDQVILSASGRSINHIPLDSPVSLLDLLSYSVEVQEAATRAQIREMVTFTACPPHKKELEALLEEGVYHEQILKKRISMLDLLEKYKACEIRFERFLELLPALKPRYYSISSSPLVAQNRLSITVGVVNAPAWSGEGTYEGVASNYLAQRHNKDEIICFIRTPQSNFELPKDPETPIIMVGPGTGVAPFRGFLQARRVQKQKGINLGQAHLYFGCRHPEKDYLYRTELENDERDGLISLHTAFSRLEGHPKTYVQHLIKQDSINLISLLDNGAHLYICGDGSKMAPDVEDTLCQAYQEIHEVSEQEARNWLDRVQDEGRYGKDVWAGI

>CYP102A5_ortholog(MC28_2359)Bacillus thuringiensis MC28

MLFERVFLMDKKVSAIPQPKTYGPLGNLPLIDKDKPTLSFIKIAEEYGPIFQIQTLSDTIIVVSGHELVAEVCDETRFDKSIDGALAKVRAFAGDGLFTSETDEPNWKKAHNILMPTFSQRAMKDYHGMMVDIAVQLVQKWARLNPNENVDVPEDMTRLTLDTIGLCGFNYRFNSFYRETSHPFITSMSRALDEAMHQLQRLDIEDKLMWRTKRQFQHDIQSMFSLVDNIIAERKSNGNQEENDLLARMLNVQDPETGEKLDDENIRFQIITFLIAGHETTSGLLSFAIYFLLKNPDKLKKAYEEVDRVLTDPTPTYQQVMKLKYIRMILNESLRLWPTAPAFSLYAKEDTVIGGKYPIKKGEDRISVLIPQLHRDKDAWGDNVEEFQPERFEEPDKVPHHAYKPFGNGQRACIGMQFALHEATLVMGMLLQHFELIDYQNYQLDVKQTLTLKPGDFKIRILARNQTISHPTVLAPIEEKQKDHEIKQQVQKTPSIIGADNLSLLVLYGSDTGVAEGIARELADTASLEGVQTEVVALNERIGSLPKEGAVLIVTSSYNGKPPSNAGQFVQWLEELKSDELKGVQYAVFGCGDHNWASTYQRIPRYIDEQMAQKGATRFSKRGEADASGDFEEQLEQWKQSMWSDAMRAFGLELNKNMEKERSTLSLQFVSRLGGSPLARTYEAVYASILENRELQSSSSDRSTRHIEVSLPEGATYQEGDHLGVLPINSEKNVNRILKRFGLNGKDQVILSASGRSVNHIPLDSPVSLFDLISYSVEVQEAATRAQIREMVTFTACPPHKKELESLLEEGVYHERILKKRISMLDLLEKYEACEIRFERFLELLPALKPRYYSISSSPLVAQDRLSITVGVVNAPAWSGEGTYEGVASNYLAQRHNKDEIICFIRTPQSNFQLPENPETPIIMVGPGTGIAPFRGFLQARRVQKQKGINLGQAHLYFGCRHPEKDYLYRTELENDERDGLISLHTAFSRLEGHPKTYVQHLIKQDRINLISLLDNGAHLYICGDGSKMAPDVEDTLCQAYEEIHEVSEQEARNWLDHLQHEGRYGKDVWTGI

>CYP102A7_ortholog(BLi02848)Bacillus licheniformis DSM 13 = ATCC 14580

MNKLDGIPIPKTYGPLGNLPLLDKNRVSQSLWKIADEMGPIFQFKFADAIGVFVSSHELVKEVSEESRFDKNMGKGLLKVREFSGDGLFTSWTEEPNWRKAHNILLPSFSQKAMKGYHPMMQDIAVQLIQKWSRLNQDESIDVPDDMTRLTLDTIGLCGFNYRFNSFYREGQHPFIESMVRGLSEAMRQTKRFPLQDKLMIQTKRRFNSDVESMFSLVDRIIADRKQAESESGNDLLSLMLHAKDPETGEKLDDENIRYQIITFLIAGHETTSGLLSFAIYLLLKHPDKLKKAYEEADRVLTDPVPSYKQVQQLKYIRMILNESIRLWPTAPAFSLYAKEETVIGGKYLIPKGQSVTVLIPKLHRDQSVWGEDAEAFRPERFEQMDSIPAHAYKPFGNGQRACIGMQFALHEATLVLGMILQYFDLEDHANYQLKIKESLTLKPDGFTIRVRPRKKEAMTAMPGAQPEENGRQEERPSAPAAENTHGTPLLVLYGSNLGTAEEIAKELAEEAREQGFHSRTAELDQYAGAIPAEGAVIIVTASYNGNPPDCAKEFVNWLEHDQTDDLRGVKYAVFGCGNRSWASTYQRIPRLIDSVLEKKGAQRLHKLGEGDAGDDFEGQFESWKYDLWPLLRTEFSLAEPEPNQTETDRQALSVEFVNAPAASPLAKAYQVFTAKISANRELQCEKSGRSTRHIEISLPEGAAYQEGDHLGVLPQNSEVLIGRVFQRFGLNGNEQILISGRNQASHLPLERPVHVKDLFQHCVELQEPATRAQIRELAAHTVCPPHQRELEDLLKDDVYKDQVLNKRLTMLDLLEQYPACELPFARFLALLPPLKPRYYSISSSPQLNPRQTSITVSVVSGPALSGRGHYKGVASNYLAGLEPGDAISCFIREPQSGFRLPEDPETPVIMVGPGTGIAPYRGFLQARRIQRDAGVKLGEAHLYFGCRRPNEDFLYRDELEQAEKDGIVHLHTAFSRLEGRPKTYVQDLLREDAALLIHLLNEGGRLYVCGDGSRMAPAVEQALCEAYRIVQGASREESQSWLSALLEEGRYAKDVWDGGVSQHNVKADCIART

>CYP102A7_ortholog(BL02398)Bacillus licheniformis ATCC 14580

MNKLDGIPIPKTYGPLGNLPLLDKNRVSQSLWKIADEMGPIFQFKFADAIGVFVSSHELVKEVSEESRFDKNMGKGLLKVREFSGDGLFTSWTEEPNWRKAHNILLPSFSQKAMKGYHPMMQDIAVQLIQKWSRLNQDESIDVPDDMTRLTLDTIGLCGFNYRFNSFYREGQHPFIESMVRGLSEAMRQTKRFPLQDKLMIQTKRRFNSDVESMFSLVDRIIADRKQAESESGNDLLSLMLHAKDPETGEKLDDENIRYQIITFLIAGHETTSGLLSFAIYLLLKHPDKLKKAYEEADRVLTDPVPSYKQVQQLKYIRMILNESIRLWPTAPAFSLYAKEETVIGGKYLIPKGQSVTVLIPKLHRDQSVWGEDAEAFRPERFEQMDSIPAHAYKPFGNGQRACIGMQFALHEATLVLGMILQYFDLEDHANYQLKIKESLTLKPDGFTIRVRPRKKEAMTAMPGAQPEENGRQEERPSAPAAENTHGTPLLVLYGSNLGTAEEIAKELAEEAREQGFHSRTAELDQYAGAIPAEGAVIIVTASYNGNPPDCAKEFVNWLEHDQTDDLRGVKYAVFGCGNRSWASTYQRIPRLIDSVLEKKGAQRLHKLGEGDAGDDFEGQFESWKYDLWPLLRTEFSLAEPEPNQTETDRQALSVEFVNAPAASPLAKAYQVFTAKISANRELQCEKSGRSTRHIEISLPEGAAYQEGDHLGVLPQNSEVLIGRVFQRFGLNGNEQILISGRNQASHLPLERPVHVKDLFQHCVELQEPATRAQIRELAAHTVCPPHQRELEDLLKDDVYKDQVLNKRLTMLDLLEQYPACELPFARFLALLPPLKPRYYSISSSPQLNPRQTSITVSVVSGPALSGRGHYKGVASNYLAGLEPGDAISCFIREPQSGFRLPEDPETPVIMVGPGTGIAPYRGFLQARRIQRDAGVKLGEAHLYFGCRRPNEDFLYRDELEQAEKDGIVHLHTAFSRLEGRPKTYVQDLLREDAALLIHLLNEGGRLYVCGDGSRMAPAVEQALCEAYRIVQGASREESQSWLSALLEEGRYAKDVWDGGVSQHNVKADCIART

>CYP102A7_ortholog(BaLi_c29470)Bacillus paralicheniformis

MNMLNGIPIPKTYGPLGNLPLLDKNKVSQSLWKIADEMGPIFQFKFADAIGIFVSSHELVKEVSDESRFDKNMGKGLLKVREFSGDGLFTSWTKEPNWRKAHNILLPSFSQKAMKGYHPMMQDIAVQLIQKWSRLNQDESIDVPDDMTRLTLDTIGLCGFNYRFNSFYREGQHPFIESMVRGLSEAMRQTKRFPLQDKLMVQTKRQFDSDVESMFSLVDRIIADRKQAGGESGNDLLSLMLHAKDPETGEKLDDENIRYQIITFLIAGHETTSGLLSFAIYLLLKHPDKLKKAYEEADRVLTDPVPSYKQVQQLKYIRMILNESIRLWPTAPAFSLYAKDETVIGGKYLIPKGQSVTVLIPKLHRDQSVWGEDAEGFRPERFEQMDSIPAHAYKPFGNGQRACIGMQFALHEATLVLGMILQYFDLEDHANYQLKIKESLTLKPDGFTIRVRPRKKEAMMVTPGAQPEENMRQEEKPSAPAAENTHGTPLLVLYGSNLGTAEEVAKELAEEAREQGYRSRTAELDQYPGALPAEGAVIIVTASYNGNPPDCAREFVNWLEHDQTGDLHGVKYAVFGCGNRSWASTYQRIPRLIDSALENRGAQRLHKLGEGDAGDDFEGQFESWKNDLWPLLRTEFSLSEPDPNQTETDRQAISVEFVSAPAAAPLAKAYQVFTAKISANRELQCEESGRSTRHIEISLPEGTAYQEGDHLGVLPQNSGVLIERVFQRFGLNGDEQILISGRNQASHLPLERPVHVKDLFQHCVELQEPAARAQIRELAAHTVCPPHQRELEDLLKDDVYKNQVLKKRLTMLDLLEQYPACELPFARFLALLPPLKPRYYSISSSPQLNPRQTSITVSVVSGPALSGRGQYKGVASNYLAGLAPKDAISCFIREPQSGFRLPEDPETPVIMVGPGTGIAPYRGFLQARRIQRDAGIKLGEAHLYFGCRRPDEDFLYRDELEQAEKDGIVHLHTAFSRLEGRPKTYVQDLLREDADMLIHLLNEGGRLYVCGDGSRMAPAVEQALCEAYRIVQGASQEESESWLSGLLEEGRYAKDVWDGGVSQHDVNADSIART

>CYP102A8_ortholog(BT9727_2981)Bacillus thuringiensis HD-771

MDKKVSAIPQPKTYGPLGNLPLIDKDKPTLSFIKLAEEYGPIFQIQTLSDTIIVVSGHELVAEVCDETRFDKSIEGALAKVRAFAGDGLFTSETDEPNWKKAHNILMPTFSQRAMKDYHAMMVDIAVQLVQKWARLNPNENVDVPEDMTRLTLDTIGLCGFNYRFNSFYRETPHPFITSMTRALDEAMHQLQRLDIEDKLMWRTKRQFQHDIQSMFSLVDNIIAERKSSENQEENDLLSRMLNVQDPETGEKLDDENIRFQIITFLIAGHETTSGLLSFAIYFLLKNPDKLKKAYEEVDRVLTDSTPTYQQVMKLKYIRMILNESLRLWPTAPAFSLYAKEDTVIGGKYPIKKGEDRISVLIPQLHRDKDAWGDDVEEFQPERFEELDKVPHHAYKPFGNGQRACIGMQFALHEATLVMGMLLQHFEFIDYEDYQLDVKQTLTLKPGDFKIRIVPRNQTISHTTVLAPTEEKLKKHEIKKQVQKTPSIIGADNLSLLVLYGSDTGVAEGIARELADTASLEGVQTEVVALNDRIGSLPKEGAVLIVTSSYNGKPPSNAGQFVQWLEELKPDELKGVQYAVFGCGDHNWASTYQRIPRYIDEQMAQKGATRFSTRGEADASGDFEEQLEQWKQSMWSDAMKAFGLELNKNMEKERSTLSLQFVSRLGGSPLARTYEAVYASILENRELQSSSSERSTRHIEISLPEGATYKEGDHLGVLPINNEKNVNRILKRFGLNGKDQVILSASGRSVNHIPLDSPVRLYDLLSYSVEVQEAATRAQIREMVTFTACPPHKKELESLLEDGVYQEQILKKRISMLDLLEKYEACEIRFERFLELLPALKPRYYSISSSPLVAQDRLSITVGVVNAPAWSGEGTYEGVASNYLAQRHNKDEIICFIRTPQSNFQLPENPETPIIMVGPGTGIAPFRGFLQARRVQKQKGMKVGEAHLYFGCRHPEKDYLYRTELENDERDGLISLHTAFSRLEGHPKTYVQHVIKEDRIHLISLLDNGAHLYICGDGSKMAPDVEDTLCQAYQEIHEVSEQEARNWLDRLQEEGRYGKDVWAGI

>CYP102A8_ortholog(BF38_4372)Bacillus thuringiensis HD1011

MDKKVSAIPQPKTYGPLGNLPLIDKDKPTLSFIKLAEEYGPIFRMQTLSDTIIVVSGHELVAEVCDETRFDKSIEGALAKVRAFAGDGLFTSETQEPNWQKAHNILMPTFSQRAMKDYHAMMVDIAVQLVQKWARLNPNENVDVPEDMTRLTLDTIGLCGFNYRFNSFYRETPHPFITSMTRALDEAMHQLQRLDIEDKLMWRTKRQFQHDIQSMFSLVDNIIAERKSSENQEENDLLSRMLNVQDPETGEKLDDENIRFQIITFLIAGHETTSGLLSFAIYFLLKNPDKLKKAYEEVDRVLTDPTPTYQQVMKLKYIRMILNESLRLWPTAPAFSLYAKEDTVIGGKYPIKKGEDRISVLIPQLHRDKDAWGDNVEEFQPERFEELDRVPHHAYKPFGNGQRACIGMQFALHEATLVMGMLLQHFEFIDYEDYQLDVKQTLTLKPGDFKIRIVPRNQTISHTTVLAPTEEKLKKHEIKQQVQKTPSIIGADNLSLLVLYGSDTGVAEGIARELADTASLEGVQTEVVALNDRIGSLPKEGAVLIVTSSYNGKPPSNAGQFVQWLEELKPDELKGVQYAVFGCGDHNWASTYQRIPRYIDEQMAQKGATRFSTRGEADASGDFEEQLEQWKQRMWSDAMKAFGLELNKNMEKERSTLSLQFVSRLGGSPLARTYEAVYASILENRELQSSSSERSTRHIEISLPEGATYKEGDHLGVLPINSEKNVNRILKRFGLNGKDQVILSASGRSVNHIPLDSPVRLYDLLSYSVEVQEAATRAQIREMVTFTACPPHKKELESLLEDGVYQEQILKKRISMLDLLEKYEACEIRFERFLELLPALKPRYYSISSSPLVAQDRLSITVGVVNAPAWSGEGTYEGVASNYLAQRHNKDEIICFIRTPQSNFQLPENPETPIIMVGPGTGIAPFRGFLQARRVQKQKGMNVGEAHLYFGCRHPEKDYLYRTELENDERDGLISLHTAFSRLEGHPKTYVQHVIKEDRIHLISLLDNGAHLYICGDGSKMAPDVEDTLCQAYQEIHEVSEQEARNWLDRVQEEGRYGKDVWAGI

>CYP102A8_ortholog(bcf_15685)Bacillus cereus F837/76

MDKKVSAIPQPKTYGPLGNLPLIDKDKPTLSFIKLAEEYGPIFRIQTLSDTIIVVSGHELVAEVCDETRFDKSIEGALAKVRAFAGDGLFTSETQEPNWQKAHNILMPTFSQRAMKDYHAMMVDIAVQLVQKWARLNPNENVDVPEDMTRLTLDTIGLCGFNYRFNSFYRETPHPFITSMTRALDEAMHQLQRLDIEDKLMWRTKRQFQHDIQSMFSLVDNIIAERKSSENQEENDLLSRMLNVQDPETGEKLDDENIRFQIITFLIAGHETTSGLLSFAIYFLLKNPDKLKKAYEEVDRVLTDPTPTYQQVMKLKYIRMILNESLRLWPTAPAFSLYAKEDTVIGGKYPIKKGEDRISVLIPQLHRDKDAWGDNVEEFQPERFEELDKVPHHAYKPFGNGQRACIGMQFALHEATLVMGMLLQHFEFIDYEDYQLDVKQTLTLKPGDFKIRIVPRNQTISHTTVLAPTEEKLKNHEIKQQVQKTPSIIGADNLSLLVLYGSDTGVAEGIARELADTASLEGVQTEVVALNDRIGSLPKEGAVLIVTSSYNGKPPSNAGQFVQWLEELKPDELKGVQYAVFGCGDHNWASTYQRIPRYIDEQMAQKGATRFSTRGEADASGDFEEQLEQWKQRMWSDAMKAFGLELNKNMEKERSTLSLQFVSRLGGSPLARTYEAVYASILENRELQSSSSERSTRHIEISLPEGATYKEGDHLGVLPINSEKNVNRILKRFGLNGKDQVILSASGRSVNHIPLDSPVRLYDLLSYSVEVQEAATRAQIREMVAFTACPPHKKELESLLEDGIYHEQILKKRISMLDLLEKYEACEIRFERFLELLPALKPRYYSISSSPLVAQDRLSITVGVVNAPAWSGEGTYEGVASNYLAQRHNKDEIICFIRTPQSNFQLPENPETPIIMVGPGTGIAPFRGFLQARRVQKQKGMKVGEAHLYFGCRHPEKDYLYRTELENDEREGLISLHTAFSRLEGHPKTYVQHVIKEDRIHLISLLDNGAHLYICGDGSKMAPDVEDTLCQAYQEIHQASEQEARNWLDRLQDEGRYGKDVWAGI

>CYP102A8_ortholog(BALH_2868)Bacillus thuringiensis Al Hakam

MLFERVFLMDKKVSAIPQPKTYGPLGNLPLIDKDKPTLSFIKLAEEYGPIFRIQTLSDTIIVVSGHELVAEVCDETRFDKSIEGALAKVRAFAGDGLFTSETQEPNWQKAHNILMPTFSQRAMKDYHAMMVDIAVQLVQKWARLNPNENVDVPEDMTRLTLDTIGLCGFNYRFNSFYRETPHPFITSMTRALDEAMHQLQRLDIEDKLMWRTKRQFQHDIQSMFSLVDNIIAERKSSENQEENDLLSRMLNVQDPETGEKLDDENIRFQIITFLIAGHETTSGLLSFAIYFLLKNPDKLKKAYEEVDRVLTDPTPTYQQVMKLKYIRMILNESLRLWPTAPAFSLYAKEDTVIGGKYPIKKGEDRISVLIPQLHRDKDAWGDNVEEFQPERFEELDKVPHHAYKPFGNGQRACIGMQFALHEATLVMGMLLQHFEFIDYEDYQLDVKQTLTLKPGDFKIRIVPRNQTISHTTVLAPTEEKLKNHEIKQQVQKTPSIIGADNLSLLVLYGSDTGVAEGIARELADTASLEGVQTEVVALNDRIGSLPKEGAVLIVTSSYNGKPPSNAGQFVQWLEELKPDELKGVQYAVFGCGDHNWASTYQRIPRYIDEQMAQKGATRFSTRGEADASGDFEEQLEQWKQRMWSDAMKAFGLELNKNMEKERSTLSLQFVSRLGGSPLARTYEAVYASILENRELQSSSSERSTRHIEISLPEGATYKEGDHLGVLPINSEKNVNRILKRFGLNGKDQVILSASGRSVNHIPLDSPVRLYDLLSYSVEVQEAATRAQIREMVAFTACPPHKKELESLLEDGIYHEQILKKRISMLDLLEKYEACEIRFERFLELLPALKPRYYSISSSPLVAQDRLSITVGVVNAPAWSGEGTYEGVASNYLAQRHNKDEIICFIRTPQSNFQLPENPETPIIMVGPGTGIAPFRGFLQARRVQKQKGMKVGEAHLYFGCRHPEKDYLYRTELENDEREGLISLHTAFSRLEGHPKTYVQHVIKEDRIHLISLLDNGAHLYICGDGSKMAPDVEDTLCQAYQEIHQASEQEARNWLDRLQDEGRYGKDVWAGI

>CYP102A8_ortholog(BCQ_3034)Bacillus cereus Q1

MDKKVSAIPQPKTYGPLGNLPLIDKDKPTLSFIKLAEEYGPIFQIQTLSDTIIVVSGHELVVEVCDETRFDKSIEGALAKVRAFAGDGLFTSETDEPNWKKAHNILMPTFSQRAMKDYHAMMVDIAVQLVQKWARLNPNENVDVPGDMTRLTLDTIGLCGFNYRFNSFYRETSHPFITSMTRALDEAMHQLQRLDIEDKLMWRTKRQFQHDIQSMFSLVDNIIAERKSSGNQEENDLLSRMLHVQDPETGEKLDDENIRFQIITFLIAGHETTSGLLSFAIYFLLKNPDKLKKAYEEVDRVLTDPTPTYQQVMKLKYIRMILNESLRLWPTAPAFSLYAKEDTVIGGKYPIKKGEDRISVLIPQLHRDKDAWGDNVEEFQPERFEELDKVPHHAYKPFGNGQRACIGMQFALHEATLVMGMLLQHFEFIDYEDYQLDVKQTLTLKPGDFKIRIVPRNQTISHTTVLAPTEEKLKNHETKQQVQKTPSIIGADNLSLLVLYGSDTGVAEGIARELADTASLEGVQTEVAALNDRIGSLPKEGAVLIVTSSYNGKPPSNAGQFVQWLEELKPDELKGVQYAVFGCGDHNWASTYQRIPRYIDEQMAQKGATRFSKRGEADASGDFEEQLEQWKQSMWSDAMKSFGLELNKNMEKERSTLSLQFVSRLGGSPLARTYEAVYASILENRELQSSSSERSTRHIEISLPEGATYKEGDHLGVLPINSEKNVNRILKRFGLNGKDQVILSASGRSVNHIPLDSPVRLYDLLSYSVEVQEAATRAQIREMVTFTVCPPHKKELESLLEEGVYQEQILKKRISMLDLLEKYEACEIRFERFLELLPALKPRYYSISSSPLVAQDRLSITVGVVNAPAWSGEGTYEGVASNYLAQRHNKDEIICFIRTPQSNFQLPENPETPIIMVGPGTGIAPFRGFLQARRVQKQKGMKVGEAHLYFGCRHPEKDYLYRTELENDEREGLISLHTAFSRLEGHPKTYVQHVIKEDRIHLISLLDNGAHLYICGDGSKMAPDVEDTLCQAYQEIHEVSEQEARNWLDRLQDEGRYGKDVWAGI

>CYP102A8_ortholog(BcrFT9_02490)Bacillus cereus FT9

MKDYHAMMVDIAVQLVQKWARLNPNENVDVPGDMTRLTLDTIGLCGFNYRFNSFYRETSHPFITSMSRALDEAMHQLQRLDIEDKLMWRTKRQFQHDIQSMFSLVDNIIAERKSSGNQEENDLLSRMLNVQDPETGEKLDDENIRFQIITFLIAGHETTSGLLSFAIYFLLKNPDKLKKAYEEVDRVLTDPTPTYQQVMKLKYIRMILNESLRLWPTAPAFSLYAKEDTVIGGKYPIKKGEDRISVLIPQLHRDKDAWGDNVEEFQPERFEELDNVPHHAYKPFGNGQRACIGMQFALHEATLVMGMLLQHFEFIDYEDYQLDVKQTLTLKPGDFKIRIVPRNQTISHTTVLAPTEEKLKNHETKQQVQKTPSIIGADNLSLLVLYGSDTGVAEGIARELADTASLEGVQTEVAALNDRIGSLPKEGAVLIVTSSYNGKPPSNAGQFVQWLEELKPDELKGVQYAVFGCGDHNWASTYQRIPRYIDEQMAQKGATRFSKRGEADASGDFEEQLEQWKQSMWSDAMKAFGLELNKNMEKERSTLSLQFVSRLGGSPLARTYEAVYASILENRELQSSSSERSTRHIEISLPEGATYKEGDHLGVLPINSEKNVNRILKRFGLNGKDQVILSASGRSVNHIPLDSPVRLYDLLSYSVEVQEAATRAQIREMVTFTVCPPHKKELESLLEEGVYQEQILKKRISMLDLLEKYEACEIRFERFLELLPALKPRYYSISSSPLVAQDRLSITVGVVNAPAWSGEGTYEGVASNYLAQRHNKDEIICFIRTPQSNFQLPENPETPIIMVGPGTGIAPFRGFLQARRVQKQKGMKVGEAHLYFGCRHPEKDYLYRTELENDEREGLISLHTAFSRLEGHPKTYVQHVIKEDRIHLISLLDNGAHLYICGDGSKMAPDVEDTLCQAYQEIHEVSEQEARNWLDRVQEEGRYGKDVWAGI

>CYP102A8_ortholog(BACI_c31660)Bacillus cereus biovar anthracis CI

MDKKVSAIPQPKTYGPLGNLPLIDKDKPTLSFIKLAEEYGPIFQIQTLSDTIIVVSGHELVAEVCDETRFDKSIEGALAKVRAFAGDGLFTSETDEPNWKKAHNILMPTFSQRAMKDYHAMMVDIAVQLVQKWARLNPKENVDVPGDMTRLTLDTIGLCGFNYRFNSFYRETSHPFITSMTRALDEAMHQLQRLDIEDKLMWRTKRQFQHDIQSMFSLVDNIIAERKSSGNQEENDLLSRMLNVKDPETGEKLDDENIRFQIITFLIAGHETTSGLLSFAIYFLLKNPDKLKKAYEEVDRVLTDPTPTYQQVMKLKYIRMILNESLRLWPTAPAFSLYAKEDTVIGGKYPIKKGEDRISVLIPQLHRDKDAWGDNVEEFQPERFEELDKVPHHAYKPFGNGQRACIGMQFALHEATLVMGMLLQHFEFIDYEDYQLDVKQTLTLKPGDFKIRIVPRNETISHTTVLAPTEEKLKNHETKQQVQKTPSIIGADNLSLLVLYGSDTGVAEGIARELADTASLEGVQTEVAALNDRIGSLPKEGAVLIVTSSYNGKPPSNAGQFVQWLEELKPDELKGVQYAVFGCGDHNWASTYQRIPRYIDEQMAQKGATRFSKRGEADASGDFEEQLEQWKQSMWSDAMKAFGLELNKNMEKERSTLSLQFVSRLGGSPLARTYEAVYASILENRELQSSSSERSTRHIEISLPEGATYKEGDHLGVLPINSEKNVNRILKRFGLNGKDQVILSASGRSVNHIPLDSPVRLYDLLSYSVEVQEAATRAQIREMVTFTVCPPHKKELESLLEEGVYQEQILKKRISMLDLLEKYEACEIRFERFLELLPALKPRYYSISSSPLVAQDRLSITVGVVNAPAWSGEGTYEGVASNYLAQRHNKDEIICFIRTPQSNFQLSENPETPIIMVGPGTGIAPFRGFLQARRVQKQKGMKVGEAHLYFGCRHPEKDYLYRTELENDEREGLISLHTAFSRLEGHPKTYVQHVIKEDRIHLISLLDNGAHLYICGDGSKMAPDVEDTLCQAYQEIHQASEQEARNWLDRLQEEGRYGKDVWAGI

>CYP102A8_ortholog(BCA_3251)Bacillus cereus 03BB102

MDKKVSAIPQPKTYGPLGNLPLIDKDKPTLSFIKLAEEYGPIFRIQTLSDTIIVVSGHELVAEVCDETRFDKSIEGALAKVRAFAGDGLFTSETQEPNWQKAHNILMPTFSQRAMKDYHAMMVDIAVQLVQKWARLNPNENVDVPEDMTRLTLDTIGLCGFNYRFNSFYRETPHPFITSMTRALDEAMHQLQRLDIEDKLMWRTKRQFQHDIQSMFSLVDNIIAERKSSENQEENDLLSRMLNVQDPETGEKLDDENIRFQIITFLIAGHETTSGLLSFAIYFLLKNPDKLKKAYEEVDRVLTDPTPTYQQVMKLKYIRMILNESLRLWPTAPAFSLYAKEDTVIGGKYPIKKGEDRISVLIPQLHRDKDAWGDNVEEFQPERFEELDRVPHHAYKPFGNGQRACIGMQFALHEATLVMGMLLQHFEFIDYEDYQLDVKQTLTLKPGDFKIRIVPRNQTISHTTVLAPTEEKLKNHEIKQQVQKTPSIIGADNLSLLVLYGSDTGVAEGIARELADTASLEGVQTEVVALNDRIGSLPKEGAVLIVTSSYNGKPPSNAGQFVQWLEELKPDELKGVQYAVFGCGDHNWASTYQRIPRYIDEQMAQKGATRFSKRGEADASGDFEEQLEQWKQRMWSDAMKAFGLELNKNMEKERSTLSLQFVSRLGGSPLARTYEAVYASILENRELQSSSSERSTRHIEISLPEGATYKEGDHLGVLPINSEKNVNRILKRFGLNGKDQVILSASGRSVNHIPLDSPVRLYDLLSYSVEVQEAATRAQIREMVTFTVCPPHKKELESLLEEGVYQEQILKKRISMLDLLEKYEACEIRFERFLELLPALKPRYYSISSSPLVAQDRLSITVGVVNAPAWSGEGTYEGVASNYLAQRHNKEEIICFIRTPQSNFQLPENPETPIIMVGPGTGIAPFRGFLQARRVQKQKGMNLGEAHLYFGCRHPEKDYLYRTELENDEREGLISLHTAFSRLEGHPKTYVQHVIKEDRIHLISLLDNGAHLYICGDGSKMAPDVEDTLCQAYQEIHQASEQEARNWLDRLQDEGRYGKDVWAGI

>CYP102A8_ortholog(BCE_3239)Bacillus cereus ATCC 10987

MDKKVSAIPQPKTYGPLGNLPLIDKDKPTLSFIKIAEEYGPIFQIQTLSDTIIVISGHELVAEVCDETRFDKSIEGALAKVRAFAGDGLFTSETQEPNWKKAHNILMPTFSQRAMKDYHAMMVDIAVQLVQKWARLNPNENVDVPEDMTRLTLDTIGLCGFNYRFNSFYRETPHPFITSMTRALDEAMHQLQRLDIEDKLMWRTKRQFQHDIQSMFSLVDNIIAERKSSGNQEENDLLSRMLHVQDPETGEKLDDENIRFQIITFLIAGHETTSGLLSFAIYFLLKNPDKLKKAYEEVDRVLTDPTPTYQQVMKLKYIRMILNESLRLWPTAPAFSLYAKEDTVIGGKYPIKKGEDRISVLIPQLHRDKDAWGDNVEEFQPERFEDLDKVPHHAYKPFGNGQRACIGMQFALHEATLVMGMLLQHFEFIDYEDYQLDVKQTLTLKPGDFKIRIVPRNQNISHTTVLAPTEEKLKNHEIKQQVQKTPSIIGADNLSLLVLYGSDTGVAEGIARELADTASLEGVQTEVAALNDRIGSLPKEGAVLIVTSSYNGKPPSNAGQFVQWLEELKPDELKGVQYAVFGCGDHNWASTYQRIPRYIDEQMAQKGATRFSTRGEADASGDFEEQLEQWKESMWSDAMKAFGLELNKNMEKERSTLSLQFVSRLGGSPLARTYEAVYASILENRELQSSSSERSTRHIEISLPEGATYKEGDHLGVLPINSEKNVNRILKRFGLNGKDQVILSASGRSVNHIPLDSPVRLYDLLSYSVEVQEAATRAQIREMVTFTACPPHKKELESLLEDGVYHEQILKKRISMLDLLEKYEACEIRFERFLELLPALKPRYYSISSSPLIAQDRLSITVGVVNAPAWSGEGTYEGVASNYLAQRHNKDEIICFIRTPQSNFQLPENPETPIIMVGPGTGIAPFRGFLQARRVQKQKGMNLGEAHLYFGCRHPEKDYLYRTELENDERDGLISLHTAFSRLEGHPKTYVQHVIKEDRMNLISLLDNGAHLYICGDGSKMAPDVEDTLCQAYQEIHEVSEQEARNWLDRLQDEGRYGKDVWAGI

>CYP102A8_ortholog(BCK_18985)Bacillus cereus FRI-35

MDKKVSAIPQPKTYGPLGNLPLIDKDKPTLSFIKIAEEYGPIFRMQTLSDTIIVVSGHELVAEVCDETRFDKSIEGALAKVRAFAGDGLFTSETQEPNWKKAHNILMPTFSQRAMKDYHAMMVDIAVQLVQKWARLNPNENVDVPEDMTRLTLDTIGLCGFNYRFNSFYRETPHPFITSMTRALDEAMHQLQRLDIEDKLMWRTKRQFQHDIQSMFSLVDNIIAERKNSGNQEENDLLSRMLHVQDPETGEKLDDENIRFQIITFLIAGHETTSGLLSFAIYFLLKNPDKLKKAYEEVDRVLTDPTPTYQQVMKLKYIRMILNESLRLWPTAPAFSLYAKEDTVIGGKYPIKKGEDRISVLIPQLHRDKDAWGDNVEEFQPERFEDLDKVPHHAYKPFGNGQRACIGMQFALHEATLVMGMLLQHFEFIDYEDYQLDVKQTLTLKPGDFKIRIVPRNQTISHTTVLAPVEEKLKNHEIKQQVQKTPSIIGADNLSLLVLYGSDTGVAEGIARELADTASLEGVQTEVAALNDRIGSLPKEGAVLIVTSSYNGKPPSNAGQFVQWLEELKPDELKGVQYAVFGCGDHNWASTYQRIPRYIDEQMAQKGATRFSTRGEADASGDFEEQLEQWKESMWSDAMKAFGLELNKNMEKERSTLSLQFVSRLGGSPLARTYEAVYASILENRELQSSSSERSTRHIEISLPEGATYKEGDHLGVLPINSEKNVNRILKRFGLNGKDQVILSASGRSVNHIPLDSPVRLYDLLSYSVEVQEAATRAQIREMVTFTACPPHKKELESLLEDGVYHEQILKKRISMLDLLEKYEACEIRFERFLELLPALKPRYYSISSSPLIAQDRLSITVGVVNAPAWSGEGTYEGVASNYLAQRHNQDEIICFIRTPQSNFQLPENPETPIIMVGPGTGIAPFRGFLQARRVQKQKGMNLGEAHLYFGCRHPEKDYLYRTELENDERDGLISLHTAFSRLEGHPKTYVQHVIKEDRIHLISLLDNGAHLYICGDGSKMAPDVEDTLCQAYQEIHEVSEQEARNWLDRLQDEGRYGKDVWAGI

>CYP102A8_ortholog(BCAH187_A3250)Bacillus cereus AH820

MDKKVSAIPQPKTYGPLGNLPLIDKDKPTLSFIKLAEEYGPIFQIQTLSDTIIVVSGHELVAEVCDETRFDKSIEGALAKVRAFAGDGLFTSETDEPNWKKAHNILMPTFSQRAMKDYHAMMVDIAVQLVQKWARLNPNENVDVPGDMTRLTLDTIGLCGFNYRFNSFYRETSHPFITSMTRALDEAMHQLQRLDIEDKLMWRTKRQFQHDIQSMFSLVDNIIAERKSSGNQEENDLLSRMLHVQDPETGEKLDDENIRFQIITFLIAGHETTSGLLSFAIYFLLKNPDKLEKAYEEVDRVLTDPTPTYQQVMKLKYIRMILNESLRLWPTAPAFSLYAKEDTVIGGKYPIKKGEDRISVLIPQLHRDKDAWGDNVEEFQPERFEELDKVPHHAYKPFGNGQRACIGMQFALHEATLVMGMLLQHFEFIDYEDYQLDVKQTLTLKPGDFKIRIVPRNETISHNTVLAPTEEKLKNDETKQQVQKTPSIIGADNLSLLVLYGSDTGVAEGIARELADTASLEGVQTEVAALNDRIGSLPKEGAVLIVTSSYNGKPPSNAGQFVQWLEELKPDELKGVQYAVFGCGDHNWASTYQRIPRYIDEQMAQKGATRFSKRGEADASGDFEEQLEQWKQSMWSDAMKSFGLELNKNMEKERSTLSLQFVSRLGGSPLARTYEAVYASILENRELQSSSSERSTRHIEISLPEGATYKEGDHLGVLPINSEKNVNRILKRFGLNGKDQVILSASGRSVNHIPLDSPVRLYDLLSYSVEVQEAATRAQIREMATFTVCPPHKKELESLLEEGVYQEQILKKRISMLDLLEKYEACEIRFERFLELLPALKPRYYSISSSPLVAQDRLSITVGVVNAPAWSGEGTYEGVASNYLAQRHNKDEIICFIRTPQSNFQLPENLETPIIMVGPGTGIAPFRGFLQARRVQKQKGMKVGEAHLYFGCRHPEKDYLYRTELENDEREGLISLHTAFSRLEGHPKTYVQHVIKEDRIHLISLLDNGAHLYICGDGSKMAPDVEDTLCQAYQEIHEVSEQEARNWLDRLQDEGRYGKDVWAGI

>CYP102A8_ortholog(BCN_3047)Bacillus cereus NC7401

MDKKVSAIPQPKTYGPLGNLPLIDKDKPTLSFIKLAEEYGPIFQIQTLSDTIIVVSGHELVAEVCDETRFDKSIEGALAKVRAFAGDGLFTSETDEPNWKKAHNILMPTFSQRAMKDYHAMMVDIAVQLVQKWARLNPNENVDVPGDMTRLTLDTIGLCGFNYRFNSFYRETSHPFITSMTRALDEAMHQLQRLDIEDKLMWRTKRQFQHDIQSMFSLVDNIIAERKSSGNQEENDLLSRMLHVQDPETGEKLDDENIRFQIITFLIAGHETTSGLLSFAIYFLLKNPDKLEKAYEEVDRVLTDPTPTYQQVMKLKYIRMILNESLRLWPTAPAFSLYAKEDTVIGGKYPIKKGEDRISVLIPQLHRDKDAWGDNVEEFQPERFEELDKVPHHAYKPFGNGQRACIGMQFALHEATLVMGMLLQHFEFIDYEDYQLDVKQTLTLKPGDFKIRIVPRNETISHNTVLAPTEEKLKNDETKQQVQKTPSIIGADNLSLLVLYGSDTGVAEGIARELADTASLEGVQTEVAALNDRIGSLPKEGAVLIVTSSYNGKPPSNAGQFVQWLEELKPDELKGVQYAVFGCGDHNWASTYQRIPRYIDEQMAQKGATRFSKRGEADASGDFEEQLEQWKQSMWSDAMKSFGLELNKNMEKERSTLSLQFVSRLGGSPLARTYEAVYASILENRELQSSSSERSTRHIEISLPEGATYKEGDHLGVLPINSEKNVNRILRRFGLNGKDQVILSASGRSVNHIPLDSPVRLYDLLSYSVEVQEAATRAQIREMATFTVCPPHKKELESLLEEGVYQEQILKKRISMLDLLEKYEACEIRFERFLELLPALKPRYYSISSSPLVAQDRLSITVGVVNAPAWSGEGTYEGVASNYLAQRHNKDEIICFIRTPQSNFQLPENLETPIIMVGPGTGIAPFRGFLQARRVQKQKGMKVGEAHLYFGCRHPEKDYLYRTELENDEREGLISLHTAFSRLEGHPKTYVQHVIKEDRIHLISLLDNGAHLYICGDGSKMAPDVEDTLCQAYQEIHEVSEQEARNWLDRLQDEGRYGKDVWAGI

>CYP102A8_ortholog(BCE33L2921)Bacillus cereus E33L

MDKKVSAIPQPKTYGPLGNLPLIDKDKPTLSFIKIAEEYGPIFRMQTLSDAIIVVSGHELVAEVCDETRFDKSIEGALAKVRAFAGDGLFTSETDEPNWKKAHNILMPTFSQRAMKDYHAMMVDIAVQLVQKWARLNPNENVDVPGDMTRLTLDTIGLCGFNYRFNSFYRETPHPFITSMTRALDEAMHQLQRLDIEDKLMWRTKRQFQHDIQSMFSLVDNIIAERKSSGNQEENDLLSRMLNVQDPETGEKLDDENIRFQIITFLIAGHETTSGLLSFTIYFLLKNPDKLKKAYEEVDRVLTDPTPTYQQVMKLKYIRMILNESLRLWPTAPAFSLYAKEDTVIGGKYPIKKGEDRISVLIPQLHRDKDAWGDNVEEFQPERFEELDKVPHHAYKPFGNGQRACIGMQFALHEATLVMGMLLQHFEFIDYEDYQLGVKQTLTLKPGDFKIRIVPRNQTISHNTVLAPTEEKLKNHEIKQKVQKIPSIIGADNLSLLVLYGSDTGVAEGIARELADTASLEGVQTEVAALNDRIGSLPKEGAVLIVTSSYNGKPPSNAGQFVQWLEELKGDELKGVQYAVFGCGDHNWASTYQRIPRYIDEQMAQKGATRFSTRGEADASGDFEEQLEQWKESMWSDAMKAFGLELNKNIEKERSTLSLQFVSRLGGSPLARTYEAVYASILENRELQSSSSERSTRHIEISLPEGATYKEGDHLGVLPINSEKNVNRILKRFGLNGKDQVILSASGRSVNHIPLDSPVRLYDLLSYSVEVQEAATRAQIREMVTFTACPPHKKELESLLEDGVYHEQILKKRISMLDLLEKYEACEIRFERFLELLPALKPRYYSISSSPLVAQNRLSITVGVVNAPAWSGEGTYEGVASNYLAQLHNKDEIICFIRTPQSNFQLPENPETPIIMVGPGTGIAPFRGFLQARRVQKQKGMKVGEAHLYFGCRHPEKDYLYRTELENDERDGLISLHTAFSRLEGHPKTYVQHVIKQDRIHLISLLDNGAHFYICGDGSKMAPDVEDTLCQAYQEIHEVSEQEARNWLDRLQEEGRYGKDVWAGI

>CYP102A8_ortholog(YBT020_15915)Bacillus thuringiensis serovar finitimus YBT-020

MDKKVSAIPQPKTYGPLGNLPLIDKDKPTLSFIKLAEEYGPIFQIQTLSDTIIVVSGHELVAEVCDETRFDKSIEGALAKVRAFAGDGLFTSETHEPNWKKAHNILMPTFSQRAMKDYHAMMVDIAVQLVQKWARLNPNENVDVPGDMTRLTLDTIGLCGFNYRFNSFYRETSHPFITSMTRALDEAMHQLQRLDIEDKLMWRTKRQFQHDIQSMFSLVDNIIAERKSSGNQEENDLLSRMLHVQDPETGEKLDDENIRFQIITFLIAGHETTSGLLSFAIYFLLKNPDKLKKAYEEVDRVLTDPTPTYQQVMKLKYIRMILNESLRLWPTAPAFSLYAKEDTVIGGKYPIKKGEDRISVLIPQLHRDKDAWGDNVEEFQPERFEELDKVPHHAYKPFGNGQRACIGMQFALHEATLVMGMLLQHFEFIDYEDYKLDVKQTLTLKPGDFKIRIVPRNQTISHTTVLAPTEEKLKNHETKQQVQKTPSIIGADNLSLLVLYGSDTGVAEGIARELADTASLEGVQTEVAALNDRIGSLPKEGAVLIVTSSYNGKPPSNAGQFVQWLEELKPDELKGVQYAVFGCGDHNWASTYQRIPRYIDEQMAQKGATRFSKRGEADASGDFEEQLEQWKQSMWSDAMKAFGLELNKNMEKERSTLSLQFVSRLGGSPLARTYEAVYASILENRELQSSSSERSTRHIEISLPEGATYKEGDHLGVLPINSEKNVNRILKRFGLNGKDQVILSASGRSVNHIPLDSPVRLFDLLSYSVEVQEAATRAQIREMVTFTVCPPHKKELESLLEDGVYHEQILKKRISMLDLLEKYEACEIRFERFLELLPALKPRYYSISSSPLVAQDRLSITVGVVNAPAWSGEGTYEGVASNYLAQRHNKDEIICFIRTPQSNFQLPENPETPIIMVGPGTGIAPFRGFLQARRVQKQKGMNLGEAHLYFGCRHPEKDYLYRTELENDEREGLISLHTAFSRLEGHPKTYVQHVIKEDRIHLIALLDNGAHLYICGDGSKMAPDVEDTLCEAYQEIHEVSEQEARNWLERVQDEGRYGKDVWAGI

>CYP102A8_ortholog(Btoyo_0469)Bacillus toyonensis

MDKKVSAIPQPKTYGPLGNLPLIDKDKPTLSFIKIAEEYGPIFQIQTLSDTIIVVSGHELVAEVCDETRFDKSIDGALAKVRAFAGDGLFTSETDEPNWKKAHNILMPTFSQRAMKDYHGMMVDIAVQLVQKWARLNPNENVDVPEDMTRLTLDTIGLCGFNYRFNSFYRETSHPFITSMSRALDEAMHQLQRLDIEDKLMWRTKRQFQHDIQSMFSLVDNIIAERKSNGNQEENDLLARMLNVQDPETGEKLDDENIRFQIITFLIAGHETTSGLLSFAIYFLLKNPDKLKKAYEEVDRVLTDPTPTYQQVMKLKYIRMILNESLRLWPTAPAFSLYAKEDTVIGGKYPIKKGEDRISVLIPQLHRDKDAWGDNVEEFQPERFEEPDKVPHHAYKPFGNGQRACIGMQFALHEATLVMGMLLQHFELIDYQNYQLDVKQTLTLKPGDFKIRILARNQTISHPTVLAPIEEKQKDHEIKQQVQKTPSIIGADNLSLLVLYGSDTGVAEGIARELADTASLEGVQTEVVALNERIGSLPKEGAVLIVTSSYNGKPPSNAGQFVQWLEELKPDELKGVQYAVFGCGDHNWASTYQRIPRYIDEQMAQKGATRFSTRGEADASGDFEEQLEQWKQSMWSDAMRAFGLELNKNMEKERSTLSLQFVSRLGGSPLARTYEAVYASILENRELQSSSSDRNTRHIEVSLPEGATYQEGDHLGVLPINSEKNVNRILKRFGLNGKDQVILSASGRSVNHIPLDSPVSLFDLISYSVEVQEAATRAQIREMVTFTACPPHKKELESLLEEGVYHERILKKRISMLDLLEKYEACEIRFERFLELLPALKPRYYSISSSPLVAQDRLSITVGVVNAPAWSGEGTYEGVASNYLAQRHNKDEIICFIRTPQSNFQLPENPETPIIMVGPGTGIAPFRGFLQARRVQKQKGINLGQAHLYFGCRHPEKDYLYRTELENDERDGLISLHTAFSRLEGHPKTYVQHLIKQDRINLISLLDNGAHLYICGDGSKMAPDVEDTLCQAYEEIHEVSEQEARNWLDYLQHEGRYGKDVWTGI

>CYP102A9_ortholog(BcerKBAB4_2936)Bacillus mycoides KBAB4

MDKKVSAIPQPKTYGLLGNLPLIDKDKPTLSFIKIAEEYGPIFRIQTLSDTIIVVSGHELVAEVCDETRFDKSIEGALAKVRAFAGDGLFTSETHEPNWKKAHNILMPTFSQRAMKDYHAMMVDIAVQLVQKWARLNPNENVDVPEDMTRLTLDTIGLCGFNYRFNSYYRETPHPFITSMSRALDEAMHQLQRLDIEDKLMWRTKRQFQHDIQSMFSLVDNIIAERKSSGNQEENDLLSRMLNVQDPETGEKLDDENIRFQIITFLIAGHETTSGLLSFAIYFLLKNPDKLKKAYEEVDRVLTDPTPTYQQVMKLKYIRMILNESLRLWPTAPAFSLYAKEDTVIGGKYPIKKGEDRISVLIPQLHRDKDAWGDNVEEFQPERFEELDKIPHHAYKPFGNGQRACIGMQFALHEATLVMGMLLQHFEFIDYQDYQLDVKQTLTLKPGDFKIRILPRNQTISHTTVLAPIEEKLKNDEIEQQVQKTPSIIGADNLSLLVLYGSDTGVAEGIARELADTASLEGVQTEVVALNDRIGSLPKEGAVLIVTSSYNGKPPSNAGQFVQWLEELKSDELKGVQYAVFGCGDHNWASTYQRIPRYIDEQMAQKGATRFSTRGEADASGDFEEQLEQWKQSMWSDAMKAFGLELNKNIEKERSTLSLQFVSRLGGSPLARTYEAVYASILENRELQSSSSERSTRHIEISLPEGATYQEGDHLGVLPINSEKNVNRILKRFGLNGKDQVILSASGRSVNHIPLDSPVSLFDLLSYSVEVQEAATRAQIREMVTFTACPPHKKELELLLEEGVYHEQILKKRMSMLDLLEKYEACEIRFERFLELLPALKPRYYSISSSPLVAQDRLSITVGVVNAPAWSGEGTYEGVASNYLAQRHNKDEIICFIRTPQSNFQLPENPETPIIMVGPGTGVAPFRGFLQARRVQKQKGINLGQAHLYFGCRHPEKDYLYRTELENDERDGLISLHIAFSRLEGYPKTYVQHLIKQDRINLISLLDNGAHLYICGDGSKMAPDVEDTLCQAYQEIHEVSEQEARNWLDRVQEEGRYGKDVWAGI

>CYP102A9_ortholog(bwei_1841)Bacillus mycoides WSBC 10204

MDKKVSAIPQPKTYGLLGNLPLIDKDKPTLSFIKIAEEYGPIFRIQTLSDTIIVVSGHELVAEVCDETRFDKSIEGALAKVRAFAGDGLFTSETHEPNWKKAHNILMPTFSQRAMKDYHAMMVDIAVQLVQKWARLNPNENVDVPEDMTRLTLDTIGLCGFNYRFNSYYRETPHPFITSMSRALDEAMHQLQRLDIEDKLMWRTKRQFQHDIQSMFSLVDNIIAERKSSGNQEENDLLSRMLNVQDPETGEKLDDENIRFQIITFLIAGHETTSGLLSFAIYFLLKNPDKLKKAYEEVDRVLTDPTPTYQQVMKLKYIRMILNESLRLWPTAPAFSLYAKEDTVIGGKYPIKKGEDRISVLIPQLHRDKDAWGDNVEEFQPERFEELDKIPHHAYKPFGNGQRACIGMQFALHEATLVMGMLLQHFEFIDYQDYQLDVKQTLTLKPGDFKIRILPRNQTISHTTVLAPIEEKLKNDEIEQQVQKTPSIIGADNLSLLVLYGSDTGVAEGIARELADTASLEGVQTEVVALNDRIGSLPKEGAVLIVTSSYNGKPPSNAGQFVQWLEELKPDELKGVQYAVFGCGDHNWASTYQRIPRYIDEQMAQKGATRFSTRGEADASGDFEEQLEQWKQSMWSDAMKAFGLELNKNIEKERSTLSLQFVSRLGGSPLARTYEAVYASILENRELQSSSSERSTRHIEISLPEGATYQEGDHLGVLPINSEKNVNRILKRFGLNGKDQVILSASGRSVNHIPLDSPVSLFDLLSYSVEVQEAATRAQIREMVTFTACPPHKKELELLLEEGVYHEQILKKRMSMLDLLEKYEACEIRFERFLELLPALKPRYYSISSSPLVAQDRLSITVGVVNAPAWSGVGTYEGVASNYLAQRHNKDEIICFIRTPQSNFQLPENPETPIIMVGPGTGIAPFRGFLQARRVQKQKGINLGQAHLYFGCRHPEKDYLYRTELENDERDGLISLHTAFSRLEGYPKTYVQHLIKQDRINLISLLDNGAHLYICGDGSKMAPDVEDTLCQAYQEIHEVSEQEARNWLDRVQEEGRYGKDVWAGI

>CYP102A15_ortholog(BPUM_1680)Bacillus pumilus SAFR-032

MQQTSIIPKPKTYGPFKNIPHIKKGELSQTFWRLADELGPIFQFEFSKATSIFVSSHELAKEVFDESRFDKFIGSSLNKVRTFSGDGLFTSWTEEPNWRKAHHILMPAFSQQAMKGYHEMMLDIATQLVQKWQRTGRDEEIEVAEDMTKLTLDTIGLCGFDFRFNSFYKENQHPFIESMLNGLNEAMEQASRLPVADKLMIKRRKKFEENVDFMKTLVDDIIQERRKQDKTGNDLLSLMLHAKDPETGERLSDENIRYQIITFLIAGHETTSGLLSFAIYFLLKNPEKLKKAVQEADDVLQGELPTFKQVQKLTYTRMVLNEALRLWPTAPTFSLYAKEDTIIGGKYSIKKNQSVSVLLPKLHRDQAVWGGDAEEFKPERFLHPEKIPQHAYKPFGNGQRACIGMQFALHEATMVLAMVLHNMELIDHTSYELNLKESLTIKPNDFKIKVRPRKQQLFMAPPKEETKKSTTIEESKVKSHGTPLLVLYGSNLGTAQQIANELAEDGKAKGFDMTTAPLDDYARQLPDKGAVLIVTASYNGHPPDHAKTFVDWVTQDKEKDLTNVTFAVFGCGDRNWASTYQRIPRLIDEALESKGAKRVADLGEGDAGGDMDEDKETFQKIVFEQLAKEFQLTFQEKGKETPKLSVAYTNELVERPVAKTYGAFSAVVLKNEELQSQKSERKTRHIELRLPEGKKYKEGDHIGIVPKNRDVLVQRVIDRFNLDPKQHIKLSSEKEANHLPLGQPIQIRELLASHVELQEPATRTQLRELASYTVCPPHRVELEQMAGEAYQEAILKKRVTMLDLLDQYEACEMPFAHFLALLPGLKPRYYSISSSPKIDEKRVSITVAVVKGKAWSGRGEYAGVASNYLCDLQKGEEVACFLHEAQAGFQLPPSSETPMIMIGPGTGIAPFRGFVQAREVWQKEGKRLGEAHLYFGCRHPHEDDLYFEEMQLAAQKGVVHIRRAYSRHKDQKVYVQHLLKEDGGMLIKLLDEGAYLYVCGDGKVMAPDVESTLIDLYQHEKQCSKEDAENWLTTLANNNRYVKDVWS

>CYP102A16_ortholog(PPSC2_17420)Paenibacillus polymyxa SC2

MTSTNSIPQPKTYGPLGNLPLIDTHAPVQSLVKLANEHGPIFRMDLPEGTNIYISGHKLVADACDESRFDKQVWAPLQKVRAFAGDGLFTSWTEEPNWRKAHQILLPSFSQRAMKGYHNMMLDLAVQLVQKWSRLNPDESVEVPEDMTRLTLDTIGLCGFNYRFNSFYRDQPHPFVTSMTRALDEAMGQLQRLNLQNKLMLSKKKQFKHDIETMFSLVDSIIQERKTVGNQGEEDLLARMLEGKDPETGETLDDENIRYQIITFLIAGHETTSGLLSFAIYYLLKNPRTLTKAYEEVDRVLTDSLPSYTQVRELKYIRMILNEALRLWPTAPAFSLFAKEDTLLDGTYPLKKGDSVNVLIPKLHRDTEAWGEDVEEFRPERFEDPSAIPQDAYKPFGNGQRACIGQQFALQEATLVLGMVLKHFELIDHTHYELKVKETLTLKPGGFTIQVRPRSTQTTIMLPGAAQELHEKEEQKVAAPHAEKHDTSLLSLYGSNLGTAEGLAGELADLGRNWGFKSSIATLNDHVDHLPKEGVVLITTATYNGHPPDNADAFVEWLKEVDEGQLAGIRYAVFGCGDRNWASTYQRIPRMIDELMTAKGAKRLYDRGEGDASGDFEKDWEVWNHGLWPELLNAFGIEHSDTEPQDTSSLSIEFVSDVLSAPLAANYEAATAVVTVNRELHAAESERSTRHLEIQLPTGLSYREGDHLGVLPRNPALLVNRVMQRFKLQDQNYIVLRGSDRDAAHLPLDRPVSVGDLLTLSVELQEPATRAQLRQLASFTVCPPHKKEIEALLEDTTFDQEIRKKRVTMLDILEKYPACELPFENFISLLPPLKPRYYSISSSPLESENSASITVSVVRGPARSGQGEYLGIASNYLAQLQPDDPVVIFVRKPQSGFRLPEDPTVPVIMVGPGTGVAPFRGFLQTRHVLKERGEQLGEAHLYYGCRDPKLDYLYKQELQTWEQEGIVTVHTAFSRLPGQPKRYVQHVMNEGADTLIHLLDEGAHLYVCGDGSRMAPDVENTLCAAYADIHHTSKEEAQQWLDHLQQEKRYAKDVWTGI

>CYP102A16_ortholog(PPM_3514)Paenibacillus polymyxa M1

MTSTNSIPQPKTYGPLGNLPLIDTHAPVQSLVKLANEHGPIFRMDLPEGTNIYISGHKLVADACDESRFDKQVWAPLQKVRAFAGDGLFTSWTEEPNWRKAHQILLPSFSQRAMKGYHNMMLDLAVQLVQKWSRLNPDESVEVPEDMTRLTLDTIGLCGFNYRFNSFYRDQPHPFVTSMTRALDEAMGQLQRLNLQNKLMLSKKKQFKHDIETMFSLVDSIIQERKTVGNQGEEDLLARMLEGKDPETGETLDDENIRYQIITFLIAGHETTSGLLSFAIYYLLKNPRTLTKAYEEVDRVLTDSLPSYTQVRELKYIRMILNEALRLWPTAPAFSLFAKEDTLLDGTYPLKKGDSVNVLIPKLHRDTEAWGEDVEEFRPERFEDPSAIPQDAYKPFGNGQRACIGQQFALQEATLVLGMVLKHFELIDHTHYELKVKETLTLKPGGFTIQVRPRSTQTTIMLPGAAQELHEKEEQKVAAPHAEKHDTSLLSLYGSNLGTAEGLAGELADLGRNWGFKSSIATLNDHVDHLPKEGVVLITTATYNGHPPDNADAFVEWLKEVDEGQLAGIRYAVFGCGDRNWASTYQRIPRMIDELMTAKGAKRLYDRGEGDASGDFEKDWEVWNHGLWPELLNAFGIEHSDTEPQDTSSLSIEFVSDVLSAPLAANYEAATAVVTVNRELHAAESERSTRHLEIQLPTGLSYREGDHLGVLPRNPALLVNRVMQRFKLQDQNYIVLRGSDRDAAHLPLDRPVSVGDLLTLSVELQEPATRAQLRQLASFTVCPPHKKEIEALLEDTTFDQEIRKKRVTMLDILEKYPACELPFENFISLLPPLKPRYYSISSSPLESENSASITVSVVRGPARSGQGEYLGIASNYLAQLQPDDPVVIFVRKPQSGFRLPEDPTVPVIMVGPGTGVAPFRGFLQTRHVLKERGEQLGEAHLYYGCRDPKLDYLYKQELQTWEQEGIVTVHTAFSRLPGQPKRYVQHVMNEGADTLIHLLDEGAHLYVCGDGSRMAPDVENTLCAAYADIHHTSKEEAQQWLDHLQQEKRYAKDVWTGI

>CYP102A16_ortholog(RE92_19680)Paenibacillus polymyxa Sb3-1

MTSTNSIPQPKTYGPLGNLPLIDTHAPVQSLVKLANEHGPIFRMDLPEGTNIYISGHKLVADACDESRFDKQVWAPLQKVRAFAGDGLFTSWTEEPNWRKAHQILLPSFSQRAMKGYHNMMLDLAVQLVQKWSRLNPDESVEVPEDMTRLTLDTIGLCGFNYRFNSFYRDQPHPFVTSMTRALDEAMGQLQRLNLQNKLMLSKKKQFKHDIETMFSLVDSIIQERKTVGNQGEEDLLARMLEGKDPETGETLDDENIRYQIITFLIAGHETTSGLLSFAIYYLLKNPRTLTKAYEEVDRVLTDSLPSYTQVRELKYIRMILNEALRLWPTAPAFSLFAKEDTLLDGTYPLKKGDSVNVLIPKLHRDTEAWGEDVEEFRPERFEDPSVIPQDAYKPFGNGQRACIGQQFALQEATLVLGMVLKHFELIDHTHYELKVKETLTLKPEGFTIQVRPRSTQTTIMLPGAAQELHEKEEQKVAPPHAEKHDTSLLSLYGSNLGTAEGLAGELADLGRNWGFNSSIATLNDHVDHLPKEGVVLITTASYNGHPPDNADAFVEWLKEVDEGQLAGIRYAVFGCGDRNWASTYQRIPRMIDELLAAKGAKRLYDRGEGDASGDFEKDWEAWNHGLWPELLNTFGIEHSGTEPQDTSSLSIEFVSDVLSAPLAANYEAATAIVTVNRELHAAESERSTRHLEIQLPTGLSYREGDHLGVLPRNPASLVNRVMQRFKLQDQDYIVLRGSDRDAAHLPLDRPVSVGDLLTLSVELQESATRAQLRQLASFTVCPPHKKEIEALLEDTTFDQEIRKKRVTMLDILEKYPACELPFENFISLLPPLKPRYYSISSSPLESENSASITVSVVRGPARSGQGEYLGIASNYLAQLQPGDPVVIFVRKPQSGFRLPEDPTVPVIMVGPGTGVAPFRGFLQTRHVLKERGEQLGEAHLYYGCRDPKLDYLYKQELQAWEQEGIVTVHTAFSRLPGQPKRYVQHVMNEDADTLIHLLDEGAHLYVCGDGSRMAPDVEDTLCAAYADIHHTSKEEAQQWLDHLQQEKRYAKDVWTGI

>CYP102A16_ortholog(PPSQR21_035110)Paenibacillus polymyxa SQR-21

MTSTNSIPQPKTYGPLGNLPLIDTHAPVQSLVKLANEYGPIFRMDLPEGTNIYISGHKLVADACDETRFDKQVWAPLQKVRAFAGDGLFTSWTEEPNWRKAHQILLPSFSQRAMKGYHNMMLDLAVQLVQKWSRLNPDESVEVPEDMTRLTLDTIGLCGFNYRFNSFYRDQPHPFVTSMTRALDEAMGQLQRLNLQNKLMLSKKKQFKHDIETMFSLVDSIIQERKTTGNQGEEDLLARMLEGKDPETGETLDDENIRYQIITFLIAGHETTSGLLSFAIYYLLKNPRTLTKAYEEVDRVLTDSLPSYTQVRELKYIRMILNEALRLWPTAPAFSLFAKEDTLLNGTYPLKKGDSVNVLIPKLHRDTEAWGEDVEEFRPERFEDPSAIPQDAYKPFGNGQRACIGQQFALQEATLVLGMVLKHFELIDHTHYELKVKETLTLKPEGFTIQVRPRSTQTAVMLPGAAQELHEKEEQKVAAPHAEKHDTSLLSLYGSNLGTAEGLAGELADLGRNWGFNSSIATLNDRVDHLPKEGVVLITTASYNGHPPDNADAFVEWLKQVDEGQLAGIRYAVFGCGDRNWASTYQRIPRMIDELMAAKGAERLYDRGEGDASGDFEKDWEVWNHGLWPELLNAFGIEHSDTEPQDTSSLSIEFVSDVLSAPLAANYEAATAIVTANRELHAAESERSTRHLEIQLPTGLSYREGDHLGVLPRNPASLVNRVMQRFKLQDQDYIVLKGSDRDAAHLPLDRPVSVGDLLTLSVELQESATRAQLRQLASFTVCPPHKKEIEALLEDTTFDQEIRKKRVTMLDILEKYPACELPFENFISLLPPLKPRYYSISSSPLESENSASITVSVVRGPARSGKGEYLGIASNYLAQLQPDDPVVIFVRKPQSGFRLPEDPTVPVIMVGPGTGIAPFRGFLQTRHVLKERGEQLGEAHLYYGCRDPKLDYLYNQELEAWEQEGIVTVHTAFSRLPGQPKRYVQLVMNEDADTLIHLLDEGAHLYVCGDGSRMAPDVEDTLCAAYADVHHTSKEEAQQWLEQLQQDKRYAKDVWTGI

>CYP102A16_ortholog(ABE82_17520)Paenibacillus peoriae

MTSTDLIPQPKTYGPLGNLPLIDTHAPVQSLVKLSYEYGPIFRMDLPEGTNIYISGHKLVADACDESRFDKQVWAPLQKVRAFAGDGLFTSWTEEPNWRKAHQILLPSFSQRAMKGYYNMMLDLAVQLIQKWSRLNPDESVEVPEDMTRLTLDTIGLCGFNYRFNSFYRDQPHPFVTSMTRALDEAMGQLQRLNLQNKLMLSKKKQFKHDIETMFSLVDSIIQERKTLGNQGEEDLLARMLEGKDPETGETLDDENIRYQIITFLIAGHETTSGLLSFAIYYLLKNPRTLTKAYEEVDRVLTDSLPSYTQVRELKYIRMILNEALRLWPTAPAFSLFAKEDTLLDGTYPLKKGDSVNVLIPKLHRDTEAWGEDVEEFRPERFEDPSAIPQDAYKPFGNGQRACIGQQFALQEATLVLGMVLKHFELIDHTHYELKVKETLTLKPEGFTIQVRPRSTQTAVMLPGAVQELHEQEEHKVAPPHAEKHDTSLLSLYGSNLGTAEGLAGELADLGRNWGFNSSIATLNDRVDNLPKEGVVLITTASYNGHPPDNADAFVQWLEQEGEGQLAGVRYAVFGCGDRNWASTYQRIPRLIDELMSAKGAKRLYDRGEGDASGDFEKDWEAWNHGLWPDLLNAFGVEHNDTEPQDSNSLSIEFVSAILSTPLADNYGAVTAVVTANRELLAAESERSTRHLELKLPTGLSYREGDHLGVLPRNPASLVKRVMQRFKLQEQDYIVLRGSDRDAAHLPLDRPVSIGDLLTLSVELQEPATRAQLRQLASFTVCPPHKKEIEALLEDTTFDREIRQKRVTMLDILEKYPACELPFENFISLLPPLKPRYYSISSSPLESDNSASITVGVVRGPARSGQGEYLGIASNYLAQLQPDDSIVIFVRKPQSGFRLPEDPTVPVIMVGPGTGVAPFRGFLQTRHVLKERGEQLGEAHLYYGCRNPKLDYLYKQELQAWEQEGIVTLHTAFSRLPDQPKRYVQHVMKENADTLIHLLDEGAHLYVCGDGSRMAPGVEDTLCAAYAEAHHTSKEEAQQWLDRLQQEKRYAKDVWTGI

>CYP102A16_ortholog(X809_33460)Paenibacillus polymyxa CR1

MTSTDLIPQPKTYGPLGNLPLIDTHAPVQSLVKLSYEYGPIFRMDLPEGTNIYISGHKLVADACDESRFDKQVWAPLQKVRAFAGDGLFTSWTEEPNWRKAHQILLPSFSQRAMKGYHNMMLDLAVQLIQKWSRLNPDESVEVPEDMTRLTLDTIGLCGFNYRFNSFYRDQPHPFVTSMTRALDEAMGQLQRLNLQNKLMLSKRKQFKHDIETMFSLVDSIIQERKTLGNQGEEDLLARMLEGKDPETGETLDDENIRYQIITFLIAGHETTSGLLSFAIYYLLKNPRTLTKAYEEVDRVLTDSLPSYTQVRELKYIRMILNEALRLWPTAPAFSLFAKEDTLLDGTYPLKKGDSVNVLIPKLHRDTEAWGEDIEEFRPERFEDPSAIPQDAYKPFGNGQRACIGQQFALQEATLVLGMVLKHFELIDHTHYELKVKETLTLKPEGFTIQVRPRSTQTAVMLPGAVQELHEQEEQKVAPPHAEKHDTSLLSLYGSNLGTAEGLAGELADLGRNWGFNSSIATLNDRVDNLPKEGVVLITTASYNGHPPDNADAFVQWLEQADEGQLAGVRYAVFGCGDRNWASTYQRIPRLIDELMSAKGAKRLYDRGEGDASGDFEKDWEAWNHGLWPDLLNAFGVEHNDTEPQDSNSLSIEFVSAILSTPLADNYGAVTAVVTANRELLAAESERSTRHLELKLPTGLSYREGDHLGVLPRNPASLVKRVMQRFKLQEQDYIVLRGSDRDAAHLPLDRPVSIEDLFTLSVELQEPATRAQLRQLASFTVCPPHKKEIEALLEDTTFDREIRQKRVTMLDILEKYPASELPFENFIALLPPLKPRYYSISSSPLELANSVSITVGVVRGPARSGQGEYLGIASNYLAQLQPDDPIVIFVRKPQSGFRLPEDPTVPVIMVGPGTGVAPFRGFLQTRHVLKERGEQLGEAHLYYGCRNPKLDYLYKQELQAWEQEGIVTLHTAFSRLPDQPKRYVQHVMKENVDALIHLLDEGAHLYVCGDGSRMAPDVEDTLCAAYAEAHHTSKEEAQQWLDRLQQEKRYAKDVWTGI

>CYP102A19_ortholog(BS614_16355)Paenibacillus xylanexedens

MPPISVPQPKTFGPLGNLPQLNFEEPVQSLVKLAEEYGPIFRMEYPGRSELYISGHELVAEVTDESKFDKRVWAPLAKVRAFAGDGLFTSWTEEPNWKKAHNVLLPSFSQRAMQGYHNKMIDLAVQLVQKWSRLNPDETVNVPDDMTRLTLDTIGLCGFNYRFNSFYREEPHPFITSMVRALDESMSSLQRLRLQDKLMITKKKQFEQDIRSMFSLVDHIIAERKEKPQEGADDLLSHMLSGKDPETGETLDDENIRYQIITFLIAGHETTSGLLSFAVYYLMKNPDTLAKAQAEVDQILKDPVPTYNQVRNLKYVRMVLNEALRLWPTAPAFSLYAKEDTVLAGQYPLQKGDSVSVLIPKLHRDREAWGDDVEEFRPERFEDPSKVPHDAYKPFGNGQRACIGQQFALQEATLVLGMVLKHFDFIDHSDYQLKVKETLTLKPDNFTIRVRARGGQPVMAVPGVAVEEPKPVAKRTEPDAANAHHTPMLVLYGSNLGTAEGIAREIADTARYQGFRSEVAALDDRVGKLPKEGAVIIVSASYNGQPPSNAKMFVEWIEHADANEFKGVRFVVLGCGDHNWASTYQRIPRLIDEQLSSRGAERLSPLGESDASGDFEKQVEDWTEQLWPDLARTMGLKLNTSSNSERSSLSVQFVSGLAVTPLADTYDAHVAEVLENRELHDAGSERSTRHLEIKLPEGITYKEGDHLGILPQNPPELVERVLRRYGFTGTEHLVLDASGRSAAHLPLHQPVNLYDLLSHSVELQEATTRAQLREMAAYTVCPPHKKELEALLDESVYMDEVRNKRISMLDYLVKYEACELPFERFLELLPSLKARYYSISSSPRVQPDQASITVSVVRAPAWSGQGEYKGIASNYLANLKPGDEIVMFTRTPESGFQLPEDAQVPVIMVGPGTGVAPFRGFLQARHVLKEQGREVGEAHLYFGCRNPEHDYLYKNELEAAQQEGLVKLHTAFSRVDGEEKCYVQHLMRDDARHLIPLFEEGAHLYICGDGSKMAPDVEATLQQAYADIHGKSAKQAEDWLNQLQQEGRYAKDVWTGI

>CYP102A21_ortholog(BS614_17515)Paenibacillus xylanexedens

MSDATSIPQPKMYGPLGNLPLIDKDKPTLSLGVLAEQHGPIYRLTVPGYSGLIISGPDLVAELCDVSRFDKFVYNELENVRAFGGDGLFTSRTSEPNWKKAHNILLPTFSKQAMKGYHSMMIDIADQLINKWARLNSNDTIDVADDMTRLTLDTIGLCGFNYRFNSFYRQDHSPFIESMVRALNEAMQKSSRLKIQNLLMVKTKRQFHEDIQTMFSLVDQIIEERKASSAPEEVDLLARMLNGKDPETGEMLDDENIRYQIITFLIAGHETTSGLLSFALYFLLKNPESLQKAYDEVDQILVSDSPQYEEILQLSYIRMILSESLRLWPTAPGFDVYAKEDTVIGGKYPLKKGESCSILLPQLHRDREAWGEDAELFRPERFEDTAKVPHHAYKPFGNGERACIGMQFALYEATLVLGMVLKHFELIDYSNYELDVKQTLTLKPGDFRIQVKAREVSQKMNKSTVSAKEKPAPVVHKNIASESQREEILAVEADGKPSLLVLYGSNLGTAEGIARELVEKGRSYGIPSEAATLNEWVGRIPRQGVVLIVTASYNGKPPQNATAFVDWLKGTESEEAKDVNYAVLGCGDRSWSGTYQSIPRWIDERLEELGGKRLLSRGEADAGGDMEKQVEAWQHMLWPQVLSVLGISEEAIKSSKPSASRLQMEFVREKTDMPLARTYDASYVTVVVNKELQALGSGRSTRHIEVLLPEGMSYREGDHLGVLPSNQKQNVDRILSRFGLRGDTPIQLTSDVSHLTHLPLNRPVKIHELLAYCVELQTPVSRTQLQELANHTVCPPHKRELEGMLDENSYKDDILANRITMLDLLEKYEACELPFERFLELLPPLKPRYYSISSSPSLNSEQASITVSVVREPAWSGKGEYLGVASSYLAGCSSGEDILMFVRTPESEFHLSEEVDVPMIMVGPGTGVAPFRGFLQARAVMKQRGLQLSEAHLFFGCRNDSDFIYRQELEQYEREGIVKLHTAFSRADGKPKTYVQHLMKENSKQLIHMLMSKGKLYVCGDGSQMAPAVEETLRQAYQEVQGATMQQAKDWLIQLQAGGRYVQDVWAGNKRTGETPKTVHEEVH

>CYP102A29(UP12_08650)Bacillus pumilus SH-B9

MHQSSIIPKPKTYGPFKNIPHIKKGELSQTFWRLADELGPIFQFEFSNATSIFVSSHELVKEVCDESRFDKFIGTSLNKVRAFAGDGLFTSWTEEPNWRKAHQILMPAFSQQAMKGYHEMMLDIATQLVQKWQRTGRDEEIEVAEDMTKLTLDTIGLCGFDFRFNSFYKENQHPFIESMLKGLNEAMEQSSRLPIADKLMIKRRKEFDQHVDFMSQLVDDIIQERKKQDKTGDDLLSLMLHAEDPETGERLSDENIRYQIITFLIAGHETTSGLLSFAIYFLLKNPEKLKKAIQEADEVLQGGLPTFKQVQKLSYTRMVLNEALRLWPTAPTFSLYAKEDTVIGGKYPIQKNQSVSVLLPKLHRDPAVWGEDSEDFKPERFMHPEKIPQHAYKPFGNGQRACIGMQFALHEATMVLAMVLHNLELIDHTSYELDLKESLTIKPNDFKIKVLPRKQQFFMAPPKEEPKKSTKSSDSKVASHGTSLLVLYGSNLGTAKKIANELVEDGKAKGFDVTTASLDDFTRRLPDTGAVLIVTASYNGHPPDNAKQFVDWLTQDEEQDLSNVTFAVFGCGDRNWASTYQRIPRLIDEALERKGAKRVTDIGEGDAGGDMDEDKETFQKTVFEQLAKEFKLTFQEKGKEKPNLSVAYTNELVERPVAKIYDAFSAVVLKNEELQSEKSTRQTRHIELKLPAGKVYKEGDHIGIVPKNSDALVQRVTHRFKLDPEQHIKLSSEKEASHLPLDQPIPIKELLASHVELQEPATRTQLRELAAHTVCPPHRVELEQMAGEAYQDAILKKRVTMLDLLDQYEACELPFVHFLALLPGLKPRYYSISSSPKVEEEKVSITVAVVKGKAWNGRGEYAGVASTYLCGLKEGEEVACFLHEAQAGFQLPPSSELPMIMIGPGTGIAPFRGFVQAREVWQKEGKPLGEAHLYFGCRHPHEDDLYFEEMQLAAQKGVVHIHRAYSRYNEQKIYVQHLLKEDGGTLIKLLDQGAYLYVCGDGKVMAPDVEATLIDLYQQEKQCSKEVAENWLTTLANNNRYVKDVWS

>CYP102A29_ortholog(QR42_08590)Bacillus sp. WP8

MKQTSIIPKPKTYGPFKNIPHIKKGELSQSFWRLADELGPIFQFEFSNATSIFVSSHELVKEVFDESRFDKFIGTSLSKVRAFAGDGLFTSWTEEPNWRKAHQILMPAFSQQAMKGYHEMMLDIATQLVQKWQRTGRDEEIEVAEDMTKLTLDTIGLCGFDFRFNSFYKENQHPFIESMLKGLNEAMEQSSRLPIADKLMIKRRKEFDQHVDFMKQLVDDIIQERRKQDKTGDDLLSLMLHAEDPETGERLSDENIRYQIITFLIAGHETTSGLLSFAIYFLLKNPEKLKKAVQEADEVLQGGLPTFKQVQKLSYTRMVLNEALRLWPTAPTFSLYAKEDTVIGGKYPIHKNQSVSVLLPKLHRDPAVWGEDAEEFKPERFTHPEKIPQHAYKPFGNGQRACIGMQFALHEATMVLAMVLHNLELIDHTSYELDLKESLTIKPNDFKIKVRPRKQQFFMAPPKEEPKKSTKSGESKVPSHGTPLLVLYGSNLGTAKQIANELAEDGKAKGFDVTTAPLDEYTRQLPDTGAVLIVTASYNGYPPDNAKQFVDWLAQDEEQDLSNVTFVVFGCGDRNWASTYQRIPRLIDEALERKGAKRVADLGEGDAGGDMDEDKEIFQKTVFEQLAKEFKLTFQEKGKEKPNLSVAYTNELVERPVAKIYDAFSAVVLKNEELQSEKSTRQTRHIELKLPAGKAYKEGDHIGIVPKNSDALVERVTRRFKLNPEQHIKLSSEKEASHLPLEQPIPIKELLASHVELQEPATRTQLRELAAHTVCPPHRVELEQMAGEAYQEAILKKRVTMLDLLDQYEACELPFVHFLALLPGLKPRYYSISSSPKVDEEKVSITVAVVKDKAWSGRGEYAGVASTYLCGLKEGEEVACFLHEAQDGFQLPPSSEVPMIMIGPGTGIAPFRGFVQAREVWQKEGKQLGEAHLYFGCRHPEEDDLYFDEMQLAAQNGVIHIHRAYSRYDEQKVYVQHLLKEEGGMLIELLDQGAYLYVCGDGKVMAPDVEATLIDLYQNEKQCSKETAENWLTALANDNRYVKDVWS

>CYP102A30(BW16_09260)Bacillus pumilus MTCC B6033

MQQTSIIPKPKTYGPLKNIPHIKKGELSQTFWRLADELGPIFQFEFSKATSIFVSSHELAKEVFDERRFDKFIGSSLNKVRTFSGDGLFTSWTEEPNWRKAHHILMPAFSQQAMKGYHEMMLDIATQLVQKWQRTGRDEEIEVAEDMTKLTLDTIGLCGFDFRFNSFYKENQHPFIESMLNGLNEAMDQASRLPVADKLMIKRRKEFEQDVDFMKQLVDDIIQERKKQDKTGNDLLSLMLHAKDPETGERLSDENIRYQIITFLIAGHETTSGLLSFAIYFLLKNPDKLKKAVQEADNVLQGGLPTFKQVQKLSYTRMVLNEALRLWPTAPTFSLYAKEDTVIGGKYPIKKNQSVSVLLPKIHRDQAVWGEDAEEFKPERFMHPEKIPQHAYKPFGNGQRACIGMQFALHEATMVLAMVLHNLELIDHTSYELDLKESLTIKPNDFKIKVRPRKQQFFMAPPKEEPTKSTASAEAKIASHGTPLLVLYGSNLGTAKQMANEFAEDGKAKGFDVTTAPLDDYTRKLPESGAVLIVTASYNGHPPDHAKQFVDWVTQDEEQDLSNVTFAVFGCGDRNWASTYQRIPRLIDEALERKGAKRVTDIGEGDAGGDMDEDKETFQKTVFDELAKEFNLTLQEKRQEKPNLSIAYTNELVERPVAKTYGAFSAVVLKNQELQSEKSTRQTRHIELQLPEGKHYKEGDHIGIVPKNSATLVQRVTDRFKLDPKQHMILSSEKEASHLPLNQPIQVGELLASHVELQEPVTRTQLRDLAKYTVCPPHRIELEQMAGEIYQEAILKKRVTMLDLLEQYEACELPFAHFLALLPGLKPRYYSISSSPKVDEKRLSITVAVVKGKSWSGRGEYAGVASNYLCGLQEGDEVACFLHEAQAGFGLPPSPEIPMIMIGPGTGIAPFRGFIQARETWQNEGKPLGEADLYFGCRHPHEDDLYYDEMQLAEQKGVVTIHRAYSRYEEQKVYVQHFIKNDGAKLIELLDKGAYLYICGDGKVMAPDVEATLIELYQTEKQCAKETAEQWLTSLANDNRYVKDVWS

>CYP102A31(GYMC10_2763)Paenibacillus sp. Y412MC10

MTQTKEVPQPKTYGPLGNLPLLDSAAPVQSLVKVASELGPIFQFQYPGGRKELYVSGHEYVKDACDEKRFDKRIWAPLQNVRPFAGDGLFTSATQEPNWKKAHNILLSSFSQRAMQGYHTKMVDIAMQLIQKWARLNPDETVDVPADMTRLTLDTIGLCGFNYRFNSFYREDNHPFIDSMVRALDEGMNQLHRLGIQDMFMIKKKRQFQEDIQFMFSLVDELIQDRRKHGGEEGDLLAHMLEGVDPDTGESLDHENIRYQIITFLIAGHETTSGLLSFAIYYLMKNPEALFKAVSEVDRVLKDPVPTYNQVRELKYVRMVLNESLRLWPTAPAFSLYAKEDTAIGGTYPMKKGDSVTVLIPALHRDSRVWGDDAETFRPERFEDPSRVPHDAYKPFGNGQRACIGQQFALQEATLVLGLVLKYFELIDNQPYELQVKETLTLKPEGFRIQVRSRQGGTALLVPGGGQVASLESKKRDDAKSDPSAAFAHHTPLLALYGSNLGTAEGIAREVADAAKHQGFDSQVGTLDEYAGKLPKEGVVVIVTASYNGQPPSNAKAFMEWLENANESEIEGVRYAVFGCGDHNWASTYQRIPRLIDEQLSLKGGQRLIHRGESDASGDFEREVDEWTEHLWPDLMQSLGLPVTAVHGQKRSALSVQFVQGTAAVPLTETYRAVTGRIVFSRNLQHEGSGRITQHIEISLPPGTEYREGDHLGVLPVNPKTLVQRVLRRFKLNAGSHLILSAEGRSGAHLPTGIPVRLDDLLSRSVELQEPATMAQIREMAASTVCPPHAKELQELIEEASYQAEVRAKRITMLDLLERYEACELPFERFLELLPPLKPRYYSISSSPKVSRNTASVTVSVVRDRAWSGKGEYRGIASNYLAELETGAEVLMFIRSPESGFALPEDPATPMIMIGPGTGVAPFRGFIQARQALREAGQELGAAHLYFGCRNPEQDFLYREEFERAEQEGLVTLHTAFSRVSGAEKCYVQHLMKQDGMLLLSLLEGGAQMYICGDGSRMAPEVEQTLIQSYQERHGVNAEDAAAWLSGLESTGQYVKDVWAG

>CYP102A32(BBI08_16145)Planococcus halocryophilus

MIEMKNLPQPKTYGPLGNLPLIDKEKPVQSFMKQARELGPIYQFHFPGRASTFVSSARLAAEICDEIRFDKKIGPSLQKVRPFGGDGLFTSGTEEPNWKKAHNILLPSFSQQAMKGYHEKMIDLSSQLVQKWARLNPNEEIDVPDDMTRLTLDTIGLCGFDYRFNSFYREDSHPFIEKMVRALDESMSQTQRLGIQDKLMIRSKQQFKEDIDYMFNLVDQLIVERKQTGDQGEDDLLAHMLKGKDPETGESLDDENIRFQIITFLIAGHETTSGLLSFAIYYLLKNPEKLQKAYQEVDNVLGDDTPSFKQVKQLKYVRMILNEALRLWPTAPAFSVYAKEDTTLAGEYKVEKGDSFTLLIPELHRDPSVWGEDAEAFIPERFEDISSIPHHAYKPFGNGQRACIGQQFALHEATLVLGMVLQHFELEDYMDYELNVKETLTFKPDGLKMKVKSRRKVQMFQAPAVADKAESIAQSKPDAAIESHGTPLLVLYGSNLGTAEGVARELAETARYQGFEVEAAPLDDYAGDLPTAGAVLIISASYNGNPPDNAIRFMDWLTTADKADLTGVTYAVFGCGDHNWATTYQRVPSIMDEQMAAIGASRLLARGEGDASEDFDGDLEKWQERLWPTLADHFDLDLEKREHSSSQVSMEFVSGISYTPIARTYDAFTAVVAENIELLKAADRSTRHIEVQLPSGASYQEGDHLGVLPENSPTLVNRVLNRFAIKGDEYVVLGESSGRASHLPTNQPVQLKQLVATYVEIQEPATRAQIRELASSNPCPPHKMELEQLLEDEIYKREVLGKRQTMLELLEYYPSCELEFESFISLLPALKARYYSISSSPRVAKQQASITVSLVRGEAWSGKGEYAGVTSNYLSSRQPGDKIACFIRTPQTDFQLPENPETPVIFVGPGTGIAPFRGFIQARRVLQVEGKTLGKAHLYFGCRHPEQDFLYEEELKEAEQLGLIELYSAFSRQHDEKIYVQHLMKNNAQAILSLLEQGGHLYICGDGSKMAPEVTDTLTQCYQELHQVSNQEAIAWLQGLEQSGRFAKDVWAAT

>CYP102A33(BGLY_3163)Bacillus glycinifermentans

MNKLSTIPIPKTYGPLGNLPLLDKTKVSQSLWKIADEMGPIFQFQFGETVGVFVSSHELVSEVCDESRFDKNMGKGLLKVREFSGDGLFTSWTHEPNWRKAHNILLPSFSQKAMKGYHSMMQDIALQLIQKWSRLNQNESIDVPDDMTRLTLDTIGLCGFNYRFNSFYREGQHPFIESLGRGLNEAMRQTKRLEWQDKLMVKTRRQFNRDVESMFSLVDRIIAERRQTGGGSGNDLLSLMLHAKDPETGERLDDENIRYQIITFLIAGHETTSGLLSFALYLLLQHPDKLKKAYEEADRVLTDPVPSYKQVQQLKYIRMILNESLRLFPTAPSFSLYAKEETVIGGKYLIPKGQSVSVLIPKLHRDQSVWGKDAEEFRPERFERMNSIPKNAYKPFGNGQRACIGMQFALHEATLVLGMILQYFELEDHTNYRLTIKESLTIKPDGFTMRVKPRREMALMTAAAHQEKQAASRTDIHSQAAADAHGTPLLVLYGSNLGSAEDAARELADEARLQGWESRTAELDQYKGRIPKEGAVLVVTASYNGHPPDNAKEFVNWLEQDPEKDLQGVKYAVFGCGNTSWASTYQRIPRLIDDAFEKKGAIRLYTRGEGDANDDFEGSFEAWKNGLWPNLRKAFSITEHKQQADRQELSVEFISAPAGSPLVKTYRAFTAQVLTNRELQSPDSGRSTRHIEISLPEGTAYVEGDHLGVLPQNSNALVDRVLKRFGLSGDEHVLISGERRVSHLPLDRPVNVTALFQNSVELQEPATRAQIRELAQHTVCPPHQRELAALLEDEAYKAQVLQKRLTMLDLLEQYPACELPLARFLTLLPSLKPRYYSISSSPREHARNTSITVAVVSGPALSGRGQYRGVASNYLAELAPGDSISCFVREPSSGFRLPDDPETPLIMVGPGTGIAPFRGFLQARRIVKEAGATLGEAHLYFGCRHPEHDFLYRDELEKAEQHGIVQLHTAFSRFEDGPKTYVQDLIKEDAERLIHLLNNGGRLYVCGDGANMAPAVEDALCEAYEHILQASKEEAQSWISKLQEEGRYAKDVWSGAGISGGTASVSAQPQV

>CYP102A34(BBI15_15485)Planococcus plakortidis

MIQMKNLPQPKSYGPLGNLPLIDKEKPVQSFMKQARELGPIYQFHFPGRASTFVSSAELAAEICDETRFDKKIGPALQKVRAFGGDGLFTSGTEEPNWKKAHNILLPSFSQQAMKGYHEKMIDLASQLVQKWARLNPNEEIDVPDDMTRLTLDTIGLCGFNYRFNSFYREDSHPFIEKMVRALDESMSQTQRLGIQDKLMVRSKQQFKEDIDYMFNLVDELIAERKQAGDQGEDDLLSHMLKGKDPETGESLDDENIRFQIITFLIAGHETTSGLLSFAIQYLLKHPEKLEKAYIEVDEVLGDATPSFKQVKQLKYVRMILNEALRLWPTAPAFSVYAKEDTTLAGKYEVQKGEAFTLLIPELHRDKSVWGQDAESFRPERFEDISKIPHHAYKPFGNGQRACIGQQFALHEAVLVLGMVLQHFELVDHTNYQLEVKETLTFKPDGLTMKVKPRRKVQMFQAPVAEDPVEAVVGQQAIESHGTPLLVLYGSNLGTAQGVARELSETARFKGFETQVAALDDYAGNLPTDGAVVIVSASYNGNPPDNAVRFMEWLEAAGSTEGVTYSVFGCGDRNWATTYQRVPSIIDEQLSANGASQLIERGEGDASEDFDGELEKWQGALWPALAERFGLDLETNTPASNQLSMEFISGVSHTPVARAYDAFTAVVSGNDELLKTADRSTRHIEIDLPEGAMYQEGDHLGVLPENSGELVGRVLNRFGLKGDEHVVLEGSSGRANHLPTGQPVQLAQLLASYVELQEPATRAQLRELAKSNPCPPHKAELEGLVEDETYKREVLAKRLTMLELLERFMSCEMEFENFLSLLPPLKARYYSISSSPRVSSSKASITVSVVKGDAWSGTGEYEGVASNYLAKREAGDKIACFIRTPQTAFQLPDNPETPLVMVGPGTGIAPFRGFIQARKVLKEQGSTLGAAQLYFGCRHAQEDFLYEEQLKQAEQLGLIELHSAFSRQQQEKVYVQHLMEQNAQDILGLLEQGGHLYICGDGGKMAPAVEESLIHSYQELKNATHEQALVWLGELEQSGRFAKDVWSAS

>CYP102A34_ortholog(AUC31_06375)Planococcus rifietoensis

MIQMKNLPQPKSYGPLGNLPLIDKEKPVQSFMKQARELGPIYQFHFPGRASTFVSSAELAAEICDETRFDKKIGPALQKVRAFGGDGLFTSGTEEPNWKKAHNILLPSFSQQAMKGYHEKMIDLASQLVQKWARLNPNEEIDVPDDMTRLTLDTIGLCGFNYRFNSFYREDSHPFIEKMVRALDESMSQTQRLGIQDKLMVRSKQQFKEDIDYMFNLVDQLIAERKEAGDQGEDDLLAHMLKGKDPETGESLDDENIRFQIITFLIAGHETTSGLLSFAIQYLLKHPDKLAKAYAEVDEVLGDATPSFKQVKQLKYVRMILNEALRLWPTAPAFSVYAKEDTTLAGKYEVGKGEAFTLLIPELHRDKSVWGQDAESFRPERFEDISKIPHHAYKPFGNGQRACIGQQFALHEAVLVLGMVLQHFELIDHSDYQLEVKETLTFKPDGLTMKVKPRRKVQMFQAPVAEEPEQAPEAEQAIDSHGTPLLVLYGSNLGTAQGVARELSETARFKGFESQVAALDDYAGNLPTQGAVVIVSASYNGNPPDNAVRFMEWLETADSTEGVTYSVFGCGDRNWATTYQRVPSIIDEQLSATGAAQLIGRGEGDASEDFDGELEKWQQALWPALAERFGLDLETNAQATNQLTMEFISGVSHTPSARAYDAFTAVVAGNDELLKTAERSTRHIEIQLPEGAAYQEGDHLGVLPENSKELVERVLTRFGLKGEEHVVLEGSSGRANHLPTGQPVQLEQLLASYVELQEPATRAQLRELAKSNPCPPHKQELEQLVEDETYKREVLAKRLTMLELLERFMSCEMEFEHFLALLPALKARYYSISSSPRVSSSKASITVSVVKGAAWSGTGEYEGIASNYLAKREAGDKIACFIRTPQTAFQLPENPETPLVMVGPGTGIAPFRGFIQARKVLKEQGVQLGAAQLYFGCRHAQEDFLYEEELKQAEQLGLIELHSAFSRQQQDKVYVQHLMEQNAQEILGLLEQGGHLYICGDGGKMAPAVEQSLIRSYQELRNTTHEQALAWLAELEQSGRFAKDVWSAS

>CYP102A35(BATR1942_01195)Bacillus atrophaeus 1942

MKKTSPIPQPKTFGPLGNLPLIDKDRPTLSLSKLADEYGPIFQLNTPAGTTIIVSGHELVEEICDESRFDKSIEGALEKVRAFSGDGLFTSWTHEPNWRKAHNILMPTFSQRAMKEYHSMMVDIAVQLIQKWARLNPNEAVDVPGDMTRLTLDTIGLCGFNYRFNSYYRETPHPFINSMVRALDEAMHQMQRLDFQDKLMVRTKRQFHHDIQTMFSLVDSIIAERKADGDQDEKDLLARMLNVEDPETGEKLDDENIRFQIITFLIAGHETTSGLLSFAIYFLLKNPDKMKKAYEEVDQVLTGPTPTYKQVLQLSYIRMILNESLRLWPTAPAFSLYAKEDTVIGGKYPITPKDRISVLIPQLHRDKEAWGENAEEFHPERFENPDQVPHHAYKPFGNGQRACIGMQFALHEATLVLGMILQYFKLIDHTNYELDIKQTLTLKPGDFKIRVQSRNQEAMNSAVLTSDEKAPDDQKEKPDTKSASIVGVNNRPLLVLYGSDTGTAEGVARELADTASMHGVRTEVAALNDQIGKLPKEGAVLIVTSSYNGKPPSNAGQFVQWLEEIKPGELTGVQYAVFGCGDHNWASTYQDVPRYIDEQLAQKGAARFSARGEGDVSGDFEGQLDQWKQTMWSDAMNAFGLKLNENAEKERSTLSLEFVRGLGGSPLARSYEAVHASVTENRELQSADSDRSTRHIEIALPPGVTYQEGDHLGVLPSNSQEKVNRILRRFGLKGNDQVTLTASGRSAAHLPLDRPVSLHDLLSYSVDVQEAATRAQIRELAAFTVCPPHKRELEGLAEEGVYQEKILQKRISMLDLLEEYEACEMPFERFLELLRPLKPRYYSISSSPRVNPEQAAITVGVVRSPAWSGHGEYRGVASNYLADRTPGDDIDMFVRTPESRFQLPEDPEKPIIMVGPGTGVAPFRGFLQARAALKQEGKTLGEAHLYFGCRNDNDFIYRGELEAYEKEGIVTLHTAFSRKEGIPKTYVQHLMAENAEELISILDQGGHLYVCGDGSKMAPDVEATLQKAYQSVHGVGEQEAQKWLGNLQTNGMYAKDVWAGI

>CYP102A35_ortholog(TD68_00815)Bacillus atrophaeus NRS 1221A

MKKTSPIPQPKTFGPLGNLPLIDKDRPTLSLSKLADEYGPIFQLNTPAGTTIIVSGHELVEEICDESRFDKSIEGALEKVRAFSGDGLFTSWTHEPNWRKAHNILMPTFSQRAMKEYHSMMVDIAVQLIQKWARLNPNEAVDVPGDMTRLTLDTIGLCGFNYRFNSYYRETPHPFINSMVRALDEAMHQMQRLDFQDKLMVRTKRQFHHDIQTMFSLVDSIIAERKADGDQDEKDLLARMLNVEDPETGEKLDDENIRFQIITFLIAGHETTSGLLSFAIYFLLKNPDKMKKAYEEVDQVLTGPTPTYKQVLQLSYIRMILNESLRLWPTAPAFSLYAKEDTVIGGKYPITPKDRISVLIPQLHRDKEAWGENAEEFHPERFENPDQVPHHAYKPFGNGQRACIGMQFALHEATLVLGMILQYFKLIDHTNYELDIKQTLTLKPGDFKIRVQSRNQEAMNSAVLTSDEKAPDDQKEKPDTKSASIVGVNNRPLLVLYGSDTGTAEGVARELADTASMHGVRTEVAALNDQIGKLPKEGAVLIVTSSYNGKPPSNAGQFVQWLEEIKPGELTGVQYAVFGCGDHNWASTYQDVPRYIDEQLAQKGAARFSARGEGDVSGDFEGQLDQWKQTMWSDAMNAFGLKLNENAEKERSTLSLEFVRGLGGSPLARSYEAVHASVTENRELQSADSDRSTRHIEIALPPGVTYQEGDHLGVLPSNSQEKVNRILRRFGLKGNDQVTLTASGRSAAHLPLDRPVSLHDLLSYSVDVQEAATRAQIRELAAFTVCPPHKRELEGLAEEGVYQEKILQKRISMLDLLEEYEACEMPFERFLELLRPLKPRYYSISSSPRVNPEQAAITVGVVRSPAWSGHGEYRGVASNYLADRTPGDDIDMFVRTPESRFQLPEDPEKPIIMVGPGTGVAPFRGFLQARAALKQEGKTLGEAHLYFGCRNDNDFIYRGELEAYEKEGIVTLHTAFSRKEGIPKTYVQHLMAENAEELISILDQGGHLYVCGDGSKMAPDVEATLQKAYQSVHGVGEQEAQKWLGNLQTNGMYAKDVWAGI

>CYP102A36(BSn5_15535)Bacillus subtilis BSn5

MKETSPIPQPKTFGPLGNLPLIDKDKPTLSLIKLAEEQGPIFQIHTPAGTTIVVSGHELVKEVCDEERFDKSIEGALEKVRAFSGDGLFTSWTHEPNWRKAHNILMPTFSQRAMKDYHEKMVDIAVQLIQKWARLNPNEAVDVPGDMTRLTLDTIGLCGFNYRFNSYYRETPHPFINSMVRALDEAMHQMQRLDVQDKLMVRTKRQFRHDIQTMFSLVDSIIAERRANGDQDEKDLLARMLNVEDPETGEKLDDENIRFQIITFLIAGHETTSGLLSFATYFLLKHPDKLKKAYEEVDRVLTDAAPTYKQVLELTYIRMILNESLRLWPTAPAFSLYPKEDTVIGGKFPITTNDRISVLIPQLHRDRDAWGKDAEEFRPERFEHQDQVPHHAYKPFGNGQRACIGMQFALHEATLVLGMILKYFTLIDHENYELDIKQTLTLKPGDFHIRVQSRNQDAIHADVQAVEKAASDEQKEKTEAKGTSVIGLNNRPLLVLYGSDTGTAEGVARELADTASLHGVRTETAPLNDRIGKLPKEGAVVIVTSSYNGKPPSNAGQFVQWLQEIKPGELEGVHYAVFGCGDHNWASTYQYVPRFIDEQLAEKGATRFSARGEGDVSGDFEGQLDEWKKSMWADAIKAFGLELNENADKERSTLSLQFVRGLGESPLARSYEASHASIAENRELQSADSDRSTRHIEIALPPDVEYQEGDHLGVLPKNSQTNVSRILHRFGLKGTDQVTLSASGRSAGHLPLGRPVSLHDLLSYSVEVQEAATRAQIRELAAFTVCPPHRRELEELSAEGVYQEQILKKRISMLDLLEKYEACDMPFERFLELLRPLKPRYYSISSSPRVNPRQASITVGVVRGPAWSGRGEYRGVASNDLAERQAGDDVVMFIRTPESRFQLPKDPETPIIMVGPGTGVAPFRGFLQARDVLKREGKTLGEAHLYFGCRNDRDFIYRDELERFEKDGIVTVHTAFSRKEGMPKTYVQHLMADQADTLISILDRGGRLYVCGDGSKMAPDVEAALQKAYQAVHGTGEQEAQNWLRHLQDTGMYAKDVWAGI

>CYP102A36_ortholog(BS34A_08250)Bacillus sp. BS34A

MKETSPIPQPKTFGPLGNLPLIDKDKPTLSLIKLAEEQGPIFQIHTPAGTTIVVSGHELVKEVCDEERFDKSIEGALEKVRAFSGDGLFTSWTHEPNWRKAHNILMPTFSQRAMKDYHEKMVDIAVQLIQKWARLNPNEAVDVPGDMTRLTLDTIGLCGFNYRFNSYYRETPHPFINSMVRALDEAMHQMQRLDVQDKLMVRTKRQFRYDIQTMFSLVDSIIAERRANGDQDEKDLLARMLNVEDPETGEKLDDENIRFQIITFLIAGHETTSGLLSFATYFLLKHPDKLKKAYEEVDRVLTDAAPTYKQVLELTYIRMILNESLRLWPTAPAFSLYPKEDTVIGGKFPITTNDRISVLIPQLHRDRDAWGKDAEEFRPERFEHQDQVPHHAYKPFGNGQRACIGMQFALHEATLVLGMILKYFTLIDHENYELDIKQTLTLKPGDFHISVQSRHQEAIHADVQAAEKAAPDEQKEKTEAKGASVIGLNNRPLLVLYGSDTGTAEGVARELADTASLHGVRTKTAPLNDRIGKLPKEGAVVIVTSSYNGKPPSNAGQFVQWLQEIKPGELEGVHYAVFGCGDHNWASTYQYVPRFIDEQLAEKGATRFSARGEGDVSGDFEGQLDEWKKSMWADAIKAFGLELNENADKERSTLSLQFVRGLGESPLARSYEASHASIAENRELQSADSDRSTRHIEIALPPDVEYQEGDHLGVLPKNSQTNVSRILHRFGLKGTDQVTLSASGRSAGHLPLGRPVSLHDLLSYSVEVQEAATRAQIRELASFTVCPPHRRELEELSAEGVYQEQILKKRISMLDLLEKYEACDMPFERFLELLRPLKPRYYSISSSPRVNPRQASITVGVVRGPAWSGRGEYRGVASNDLAERQAGDDVVMFIRTPESRFQLPKDPETPIIMVGPGTGVAPFRGFLQARDVLKREGKTLGEAHLYFGCRNDRDFIYRDELERFEKDGIVTVHTAFSRKEGMPKTYVQHLMADQADTLISILDRGGRLYVCGDGSKMAPDVEAALQKAYQAVHGTGEQEAQNWLRHLQDTGMYAKDVWAGI

>CYP102A36_ortholog(U712_03660)Bacillus subtilis PY79

MKETSPIPQPKTFGPLGNLPLIDKDKPTLSLIKLAEEQGPIFQIHTPAGTTIVVSGHELVKEVCDEERFDKSIEGALEKVRAFSGDGLFTSWTHEPNWRKAHNILMPTFSQRAMKDYHEKMVDIAVQLIQKWARLNPNEAVDVPGDMTRLTLDTIGLCGFNYRFNSYYRETPHPFINSMVRALDEAMHQMQRLDVQDKLMVRTKRQFRYDIQTMFSLVDSIIAERRANGDQDEKDLLARMLNVEDPETGEKLDDENIRFQIITFLIAGHETTSGLLSFATYFLLKHPDKLKKAYEEVDRVLTDAAPTYKQVLELTYIRMILNESLRLWPTAPAFSLYPKEDTVIGGKFPITTNDRISVLIPQLHRDRDAWGKDAEEFRPERFEHQDQVPHHAYKPFGNGQRACIGMQFALHEATLVLGMILKYFTLIDHENYELDIKQTLTLKPGDFHISVQSRHQEAIHADVQAAEKAAPDEQKEKTEAKGASVIGLNNRPLLVLYGSDTGTAEGVARELADTASLHGVRTKTAPLNDRIGKLPKEGAVVIVTSSYNGKPPSNAGQFVQWLQEIKPGELEGVHYAVFGCGDHNWASTYQYVPRFIDEQLAEKGATRFSARGEGDVSGDFEGQLDEWKKSMWADAIKAFGLELNENADKERSTLSLQFVRGLGESPLARSYEASHASIAENRELQSADSDRSTRHIEIALPPDVEYQEGDHLGVLPKNSQTNVSRILHRFGLKGTDQVTLSASGRSAGHLPLGRPVSLHDLLSYSVEVQEAATRAQIRELASFTVCPPHRRELEELSAEGVYQEQILKKRISMLDLLEKYEACDMPFERFLELLRPLKPRYYSISSSPRVNPRQASITVGVVRGPAWSGRGEYRGVASNDLAERQAGDDVVMFIRTPESRFQLPKDPETPIIMVGPGTGVAPFRGFLQARDVLKREGKTLGEAHLYFGCRNDRDFIYRDELERFEKDGIVTVHTAFSRKEGMPKTYVQHLMADQADTLISILDRGGRLYVCGDGSKMAPDVEAALQKAYQAVHGTGEQEAQNWLRHLQDTGMYAKDVWAGI

>CYP102A36_ortholog(B657_07250)Bacillus subtilis QB928

MKETSPIPQPKTFGPLGNLPLIDKDKPTLSLIKLAEEQGPIFQIHTPAGTTIVVSGHELVKEVCDEERFDKSIEGALEKVRAFSGDGLFTSWTHEPNWRKAHNILMPTFSQRAMKDYHEKMVDIAVQLIQKWARLNPNEAVDVPGDMTRLTLDTIGLCGFNYRFNSYYRETPHPFINSMVRALDEAMHQMQRLDVQDKLMVRTKRQFRYDIQTMFSLVDSIIAERRANGDQDEKDLLARMLNVEDPETGEKLDDENIRFQIITFLIAGHETTSGLLSFATYFLLKHPDKLKKAYEEVDRVLTDAAPTYKQVLELTYIRMILNESLRLWPTAPAFSLYPKEDTVIGGKFPITTNDRISVLIPQLHRDRDAWGKDAEEFRPERFEHQDQVPHHAYKPFGNGQRACIGMQFALHEATLVLGMILKYFTLIDHENYELDIKQTLTLKPGDFHISVQSRHQEAIHADVQAAEKAAPDEQKEKTEAKGASVIGLNNRPLLVLYGSDTGTAEGVARELADTASLHGVRTKTAPLNDRIGKLPKEGAVVIVTSSYNGKPPSNAGQFVQWLQEIKPGELEGVHYAVFGCGDHNWASTYQYVPRFIDEQLAEKGATRFSARGEGDVSGDFEGQLDEWKKSMWADAIKAFGLELNENADKERSTLSLQFVRGLGESPLARSYEASHASIAENRELQSADSDRSTRHIEIALPPDVEYQEGDHLGVLPKNSQTNVSRILHRFGLKGTDQVTLSASGRSAGHLPLGRPVSLHDLLSYSVEVQEAATRAQIRELASFTVCPPHRRELEELSAEGVYQEQILKKRISMLDLLEKYEACDMPFERFLELLRPLKPRYYSISSSPRVNPRQASITVGVVRGPAWSGRGEYRGVASNDLAERQAGDDVVMFIRTPESRFQLPKDPETPIIMVGPGTGVAPFRGFLQARDVLKREGKTLGEAHLYFGCRNDRDFIYRDELERFEKDGIVTVHTAFSRKEGMPKTYVQHLMADQADTLISILDRGGRLYVCGDGSKMAPDVEAALQKAYQAVHGTGEQEAQNWLRHLQDTGMYAKDVWAGI

>CYP102A36_ortholog(BSUA_00799)Bacillus subtilis subsp. subtilis JH642

MKETSPIPQPKTFGPLGNLPLIDKDKPTLSLIKLAEEQGPIFQIHTPAGTTIVVSGHELVKEVCDEERFDKSIEGALEKVRAFSGDGLFTSWTHEPNWRKAHNILMPTFSQRAMKDYHEKMVDIAVQLIQKWARLNPNEAVDVPGDMTRLTLDTIGLCGFNYRFNSYYRETPHPFINSMVRALDEAMHQMQRLDVQDKLMVRTKRQFRYDIQTMFSLVDSIIAERRANGDQDEKDLLARMLNVEDPETGEKLDDENIRFQIITFLIAGHETTSGLLSFATYFLLKHPDKLKKAYEEVDRVLTDAAPTYKQVLELTYIRMILNESLRLWPTAPAFSLYPKEDTVIGGKFPITTNDRISVLIPQLHRDRDAWGKDAEEFRPERFEHQDQVPHHAYKPFGNGQRACIGMQFALHEATLVLGMILKYFTLIDHENYELDIKQTLTLKPGDFHISVQSRHQEAIHADVQAAEKAAPDEQKEKTEAKGASVIGLNNRPLLVLYGSDTGTAEGVARELADTASLHGVRTKTAPLNDRIGKLPKEGAVVIVTSSYNGKPPSNAGQFVQWLQEIKPGELEGVHYAVFGCGDHNWASTYQYVPRFIDEQLAEKGATRFSARGEGDVSGDFEGQLDEWKKSMWADAIKAFGLELNENADKERSTLSLQFVRGLGESPLARSYEASHASIAENRELQSADSDRSTRHIEIALPPDVEYQEGDHLGVLPKNSQTNVSRILHRFGLKGTDQVTLSASGRSAGHLPLGRPVSLHDLLSYSVEVQEAATRAQIRELASFTVCPPHRRELEELSAEGVYQEQILKKRISMLDLLEKYEACDMPFERFLELLRPLKPRYYSISSSPRVNPRQASITVGVVRGPAWSGRGEYRGVASNDLAERQAGDDVVMFIRTPESRFQLPKDPETPIIMVGPGTGVAPFRGFLQARDVLKREGKTLGEAHLYFGCRNDRDFIYRDELERFEKDGIVTVHTAFSRKEGMPKTYVQHLMADQADTLISILDRGGRLYVCGDGSKMAPDVEAALQKAYQAVHGTGEQEAQNWLRHLQDTGMYAKDVWAGI

>CYP102A36_ortholog(BSUB_00799)Bacillus subtilis subsp. subtilis AG1839

MKETSPIPQPKTFGPLGNLPLIDKDKPTLSLIKLAEEQGPIFQIHTPAGTTIVVSGHELVKEVCDEERFDKSIEGALEKVRAFSGDGLFTSWTHEPNWRKAHNILMPTFSQRAMKDYHEKMVDIAVQLIQKWARLNPNEAVDVPGDMTRLTLDTIGLCGFNYRFNSYYRETPHPFINSMVRALDEAMHQMQRLDVQDKLMVRTKRQFRYDIQTMFSLVDSIIAERRANGDQDEKDLLARMLNVEDPETGEKLDDENIRFQIITFLIAGHETTSGLLSFATYFLLKHPDKLKKAYEEVDRVLTDAAPTYKQVLELTYIRMILNESLRLWPTAPAFSLYPKEDTVIGGKFPITTNDRISVLIPQLHRDRDAWGKDAEEFRPERFEHQDQVPHHAYKPFGNGQRACIGMQFALHEATLVLGMILKYFTLIDHENYELDIKQTLTLKPGDFHISVQSRHQEAIHADVQAAEKAAPDEQKEKTEAKGASVIGLNNRPLLVLYGSDTGTAEGVARELADTASLHGVRTKTAPLNDRIGKLPKEGAVVIVTSSYNGKPPSNAGQFVQWLQEIKPGELEGVHYAVFGCGDHNWASTYQYVPRFIDEQLAEKGATRFSARGEGDVSGDFEGQLDEWKKSMWADAIKAFGLELNENADKERSTLSLQFVRGLGESPLARSYEASHASIAENRELQSADSDRSTRHIEIALPPDVEYQEGDHLGVLPKNSQTNVSRILHRFGLKGTDQVTLSASGRSAGHLPLGRPVSLHDLLSYSVEVQEAATRAQIRELASFTVCPPHRRELEELSAEGVYQEQILKKRISMLDLLEKYEACDMPFERFLELLRPLKPRYYSISSSPRVNPRQASITVGVVRGPAWSGRGEYRGVASNDLAERQAGDDVVMFIRTPESRFQLPKDPETPIIMVGPGTGVAPFRGFLQARDVLKREGKTLGEAHLYFGCRNDRDFIYRDELERFEKDGIVTVHTAFSRKEGMPKTYVQHLMADQADTLISILDRGGRLYVCGDGSKMAPDVEAALQKAYQAVHGTGEQEAQNWLRHLQDTGMYAKDVWAGI

>CYP102A36_ortholog(BSU6051_07250)Bacillus subtilis subsp. subtilis 6051-HGW

MKETSPIPQPKTFGPLGNLPLIDKDKPTLSLIKLAEEQGPIFQIHTPAGTTIVVSGHELVKEVCDEERFDKSIEGALEKVRAFSGDGLFTSWTHEPNWRKAHNILMPTFSQRAMKDYHEKMVDIAVQLIQKWARLNPNEAVDVPGDMTRLTLDTIGLCGFNYRFNSYYRETPHPFINSMVRALDEAMHQMQRLDVQDKLMVRTKRQFRYDIQTMFSLVDSIIAERRANGDQDEKDLLARMLNVEDPETGEKLDDENIRFQIITFLIAGHETTSGLLSFATYFLLKHPDKLKKAYEEVDRVLTDAAPTYKQVLELTYIRMILNESLRLWPTAPAFSLYPKEDTVIGGKFPITTNDRISVLIPQLHRDRDAWGKDAEEFRPERFEHQDQVPHHAYKPFGNGQRACIGMQFALHEATLVLGMILKYFTLIDHENYELDIKQTLTLKPGDFHISVQSRHQEAIHADVQAAEKAAPDEQKEKTEAKGASVIGLNNRPLLVLYGSDTGTAEGVARELADTASLHGVRTKTAPLNDRIGKLPKEGAVVIVTSSYNGKPPSNAGQFVQWLQEIKPGELEGVHYAVFGCGDHNWASTYQYVPRFIDEQLAEKGATRFSARGEGDVSGDFEGQLDEWKKSMWADAIKAFGLELNENADKERSTLSLQFVRGLGESPLARSYEASHASIAENRELQSADSDRSTRHIEIALPPDVEYQEGDHLGVLPKNSQTNVSRILHRFGLKGTDQVTLSASGRSAGHLPLGRPVSLHDLLSYSVEVQEAATRAQIRELASFTVCPPHRRELEELSAEGVYQEQILKKRISMLDLLEKYEACDMPFERFLELLRPLKPRYYSISSSPRVNPRQASITVGVVRGPAWSGRGEYRGVASNDLAERQAGDDVVMFIRTPESRFQLPKDPETPIIMVGPGTGVAPFRGFLQARDVLKREGKTLGEAHLYFGCRNDRDFIYRDELERFEKDGIVTVHTAFSRKEGMPKTYVQHLMADQADTLISILDRGGRLYVCGDGSKMAPDVEAALQKAYQAVHGTGEQEAQNWLRHLQDTGMYAKDVWAGI

>CYP102A36_ortholog(BSU07250) Bacillus subtilis subsp. subtilis 168

MKETSPIPQPKTFGPLGNLPLIDKDKPTLSLIKLAEEQGPIFQIHTPAGTTIVVSGHELVKEVCDEERFDKSIEGALEKVRAFSGDGLFTSWTHEPNWRKAHNILMPTFSQRAMKDYHEKMVDIAVQLIQKWARLNPNEAVDVPGDMTRLTLDTIGLCGFNYRFNSYYRETPHPFINSMVRALDEAMHQMQRLDVQDKLMVRTKRQFRYDIQTMFSLVDSIIAERRANGDQDEKDLLARMLNVEDPETGEKLDDENIRFQIITFLIAGHETTSGLLSFATYFLLKHPDKLKKAYEEVDRVLTDAAPTYKQVLELTYIRMILNESLRLWPTAPAFSLYPKEDTVIGGKFPITTNDRISVLIPQLHRDRDAWGKDAEEFRPERFEHQDQVPHHAYKPFGNGQRACIGMQFALHEATLVLGMILKYFTLIDHENYELDIKQTLTLKPGDFHISVQSRHQEAIHADVQAAEKAAPDEQKEKTEAKGASVIGLNNRPLLVLYGSDTGTAEGVARELADTASLHGVRTKTAPLNDRIGKLPKEGAVVIVTSSYNGKPPSNAGQFVQWLQEIKPGELEGVHYAVFGCGDHNWASTYQYVPRFIDEQLAEKGATRFSARGEGDVSGDFEGQLDEWKKSMWADAIKAFGLELNENADKERSTLSLQFVRGLGESPLARSYEASHASIAENRELQSADSDRSTRHIEIALPPDVEYQEGDHLGVLPKNSQTNVSRILHRFGLKGTDQVTLSASGRSAGHLPLGRPVSLHDLLSYSVEVQEAATRAQIRELASFTVCPPHRRELEELSAEGVYQEQILKKRISMLDLLEKYEACDMPFERFLELLRPLKPRYYSISSSPRVNPRQASITVGVVRGPAWSGRGEYRGVASNDLAERQAGDDVVMFIRTPESRFQLPKDPETPIIMVGPGTGVAPFRGFLQARDVLKREGKTLGEAHLYFGCRNDRDFIYRDELERFEKDGIVTVHTAFSRKEGMPKTYVQHLMADQADTLISILDRGGRLYVCGDGSKMAPDVEAALQKAYQAVHGTGEQEAQNWLRHLQDTGMYAKDVWAGI

>CYP102A36_ortholog(C663_0751)Bacillus subtilis XF-1

MKETSPIPQPKTFGPLGNLPLIDKDKPTLSLIKLAEEQGPIFQIHTPAGTTIVVSGHELVKEVCDEERFDKSIEGALEKVRAFSGDGLFTSWTHEPNWRKAHNILMPTFSQRAMKDYHEKMVDIAVQLIQKWARLNPNEAVDVPGDMTRLTLDTIGLCGFNYRFNSYYRETPHPFINSMVRALDEAMHQMQRLDVQDKLMVRTKRQFRHDIQTMFSLVDSIIAERRSNGDQDEKDLLARMLNVEDPETGEKLDDENIRFQIITFLIAGHETTSGLLSFATYFLLKHPDKLKKAYEEVDRVLTDAAPTYKQVLELTYIRMILNESLRLWPTAPAFSLYPKEDTVIGGKFPITTKDRISVLIPQLHRDRDAWGKDAEEFRPERFEHQDQVPHHAYKPFGNGQRACIGMQFALHEATLVLGMILKYFTLIDHENYELDIKQTLTLKPGDFHIRVQSRNQEAIHADVQAAEKAVSDEQKEKTEAKGASVIGLNNRPLLLLYGSDTGTAEGVARELADTASLHGVRTETAPLNDRIGKLPKEGAVVIVTSSYNGKPPSNAGQFVQWLQEIKPGELEGVHYAVFGCGDHNWASTYQYVPRFIDEQLAEKGATRFSARGEGDVSGDFEGQLDEWKKSMWADAIKAFGLELNENADKERSTLSLQFVRGLGESPLARSYEASHASIAENRELQSADSDRSTRHIEIALPPDVEYQEGDHLGVLPKNSQTNVSRILHRFGLKGTDQVTLSASGRSAGHLPLGRPVSLHDLLSYSVEVQEAATRAQIRELAAFTVCPPHRRELEELSAEGVYQEQILKKRISMLDLLEKYEACDMPFERFLELLRPLKPRYYSISSSPRVNPRQASITVGVVRGPAWSGRGEYRGVASNDLAERKAGDDVVMFIRTPESRFQLPEDPETPIIMVGPGTGVAPFRGFLQAREVLKREGKTLGEAHLYFGCRNDRDFIYRDELEQFEKDGIVTVHTAFSRKEGMPKTYVQHLMADHAETLISILDRGGRLYVCGDGSKMAPDVEAALQKAYQSVHGTGEQEVQNWLRHLQDTGMYAKDVWAGI

>CYP102A36_ortholog(I653_03650)Bacillus subtilis subsp. subtilis BAB-1

MKETSPIPQPKTFGPLGNLPLIDKDKPTLSLIKLAEEQGPIFQIHTPAGTTIVVSGHELVKEVCDEERFDKSIEGALEKVRAFSGDGLFTSWTHEPNWRKAHNILMPTFSQRAMKDYHEKMVDIAVQLIQKWARLNPNEAVDVPGDMTRLTLDTIGLCGFNYRFNSYYRETPHPFINSMVRALDEAMHQMQRLDVQDKLMVRTKRQFRHDIQTMFSLVDSIIAERRSNGDQDEKDLLARMLNVEDPETGEKLDDENIRFQIITFLIAGHETTSGLLSFATYFLLKHPDKLKKAYEEVDRVLTDAAPTYKQVLELTYIRMILNESLRLWPTAPAFSLYPKEDTVIGGKFPITTKDRISVLIPQLHRDRDAWGKDAEEFRPERFEHQDQVPHHAHKPFGNGQRACIGMQFALHEATLVLGMILKYFTLIDHENYELDIKQTLTLKPGDFHIRVQSRNQEAIHADVQAAEKAVSDEQKEKTEAKGASVIGLNNRPLLLLYGSDTGTAEGVARELADTASLHGVRTETAPLNDRIGKLPKEGAVVIVTSSYNGKPPSNAGQFVQWLQEIKPGELEGVHYAVFGCGDHNWASTYQYVPRFIDEQLAEKGATRFSARGEGDVSGDFEGQLDEWKKSMWADAIKAFGLELNENADKERSTLSLQFVRGLGESPLARSYEASHASIAENRELQSADSDRSTRHIEIALPPDVEYQEGDHLGVLPKNSQTNVSRILHRFGLKGTDQVTLSASGRSAGHLPLGRPVSLHDLLSYSVEVQEAATRAQIRELAAFTVCPPHRRELEELSAEGVYQEQILKKRISMLDLLEKYEACDMPFERFLELLRPLKPRYYSISSSPRVNPRQASITVGVVRGPAWSGRGEYRGVASNDLAERKAGDDVVMFIRTPESRFQLPEDPETPIIMVGPGTGVAPFRGFLQAREVLKREGKTLGEAHLYFGCRNDRDFIYRDELEQFEKDGIVTVHTAFSRKEGMPKTYVQHLMADHAETLISILDRGGRLYVCGDGSKMAPDVEAALQKAYQSVHGTGEQEVQNWLRHLQDTGMYAKDVWAGI

>CYP102A36_ortholog(BSNT_07111)Bacillus subtilis subsp. natto BEST195

MKETSPIPQPKTFGPLGNLPLIDKDKPTLSLIKLAEEQGPIFQIHTPAGTTIVVSGHELVKEVCDEERFDKSIEGALEKVRAFSGDGLFTSWTHEPNWRKAHNILMPTFSQQAMKDYHEKMVDIAVQLIQKWARLNPNEVVDVPGDMTRLTLDTIGLCGFNYRFNSYYRETPHPFINSMVRALDEAMHQMQRLDVQDKLMVRTKRQFRHDIQTMFSLVDSIIAERRANGDQDEKDLLARMLNVEDPETGEKLDDENIRFQIITFLIAGHETTSGLLSFATYFLLKHPDKLKKAYEEVDRVLTDAAPTYKQVLELTYIRMILNESLRLWPTAPAFSLYPKEDTVIGGKFPITTKDRISVLIPQLHRDRDAWGKDAEEFRPERFEHQDQVPHHAYKPFGNGQRACIGMQFALHEATLVLGMILKYFTLIDHENYELDIKQTLTLKPGDFHIRVQSRHQEAIHADVQTAEKAAPDEQKEKTEAKGASVIGLNNRPLLVLYGSDTGTAEGVARELADTASLHDVRTETAPLNDRIGKLPKEGAVVIVTSSYNGKPPSNAGQFVQWLQEIKPGELEGVHYAVFGCGDHNWASTYQYVPRFIDEQLAEKGATRFSGRGEGDVSGDFEGQLDEWKKSMWADAIKAFGLELNENADKERSTLSLQFVRGLGESPLARSYEASHASIAENRELQSADSDRSTRHIEIALPPDVEYQEGDHLGVLPKNSQTNVSRILHRFGLKGTDQVTLSASGRSAGHLPLGRPVSLYDLLSYSVEVQEAATRAQIRELAAFTVCPPHRRELEELSAEGVYQEQILKKRISMLDLLEKYEACDMPFERFLELLRPLKPRYYSISSSPRVNPRLASITVGVVRGPAWSGRGEYRGVASNDLAERQAGDDVVMFIRTPESRFQLPEDPETPIIMVGPGTGVAPFRGFLQAREVLKREGKTLGEAHLYFGCRNDRDFIYRDELEQFEKDGIVTVHTAFSRKEGMPKTYVQHVMADHAETLISILDRGGRLYVCGDGSKMAPDVEAALQKAYQSVHGTGEQEAQNWLRHLQDTGMYAKDVWAGI

>CYP102A36_ortholog(BGM20_18795)Bacillus gibsonii

MKETSPIPQPKTFGPLGNLPLIDKDKPTLSLIKLAEEQGPIFQIHTPAGTTIVVSGHELVKEVCDEERFDKSIEGALEKVRAFSGDGLFTSWTHEPNWRKAHNILMPTFSQRAMKDYHEKMVDIAVQLIQKWARLNPNEAVDVPGDMTRLTLDTIGLCGFNYRFNSYYRETPHPFINSMVRALDEAMHQMQRLDVQDKLMVRTKRQFHHDIQTMFSLVDSIIAERRSNGDQDEKDLLARMLNVEDPETGEKLDDENIRFQIITFLIAGHETTSGLLSFATYFLLKHPDKLKKAYEEVDRVLTDAAPTYKQVLELTYIRMILNESLRLWPTAPAFSLYPKEDTVIGGKFPITTNDRISVLIPQLHRDRDAWGKDAEEFRPERFEHQDQVPHHAYKPFGNGQRACIGMQFALHEATLVLGMILKYFTLIDHENYELDIKQTLTLKPGDFHIRVQSRNQDAIHADVQAAEKAASDEQKEKTEAKGASVIGLNNRPLLVLYGSDTGTAEGVARELADTASLHGVRTETAPLNDRIGKLPKEGAVVIVTSSYNGKPPSNAGQFVQWLQEIKPGELEGVHYAVFGCGDHNWASTYQYVPRFIDEQLAEKGATRFSARGEGDVSGDFEGQLDEWKKSMWADAIKAFGLELNENADKERSTLSLQFVRGLGESPLARSYEASHASIAENRELQSADSDRSTRHIEIALPPDVEYQEGDHLGVLPKNSQTNVSRILHRFGLKETDQVTLSASGRSAGHLPLGRPVSLHDLLSYSVEVQEAATRAQIRELAAFTVCPPHRRELEELSAEGVYQEQILKKRISMLDLLEKYEACDMPFERFLELLRPLKPRYYSISSSPRVNPRLASITVGVVRGPAWSGRGEYRGVASNDLAERQAGDDLVMFVRTPESRFQLPEDPETPIIMVGPGTGVAPFRGFLQAREVLKREGKTLGEAHLYFGCRNDRDFIYRDELEQFEKDGIVTVHTAFSRKEGMPKTYVQHVMADHAETLISILDRGGRLYVCGDGSKMAPDVEAALQKAYQSVHGTGEQEAQNWLRHLQDTGMYAKDVWAGI

>CYP102A36_ortholog(Q433_04075)Bacillus subtilis subsp. subtilis OH 131.1

MKETSPIPQPKTFGPLGNLPLIDKDKPTLSLIKLAEEQGPIFQIHTPAGTTIVVSGHELVKEVCDEERFDKSIEGALEKVRAFSGDGLFTSWTHEPNWRKAHNILMPTFSQRAMKDYHEKMVDIAVQLIQKWARLNPNEAVDVPGDMTRLTLDTIGLCGFNYRFNSYYRETPHPFINSMVRALDEAMHQMQRLDVQDKLMVRTKRQFHHDIQTMFSLVDSIIAERRSNGDQDEKDLLARMLNVEDPETGEKLDDENIRFQIITFLIAGHETTSGLLSFATYFLLKHPDKLKKAYEEVDRVLTDAAPTYKQVLELTYIRMILNESLRLWPTAPAFSLYPKEDTVIGGKFPITTNDRISVLIPQLHRDRDAWGKDAEEFRPERFEHQDQVPHHAYKPFGNGQRACIGMQFALHEATLVLGMILKYFTLIDHENYELDIKQTLTLKPGDFHIRVQSRNQDAIHADVQAAEKAASDEQKEKTEAKGTSVIGLNNRPLLVLYGSDTGTAEGVARELADTASLHGVRTETAPLNDRIGKLPKEGAVVIVTSSYNGKPPSNAGQFVQWLQEIKPGELEGVHYAVFGCGDHNWASTYQYVPRFIDEQLAEKGATRFSARGEGDVSGDFEGQLDEWKKSMWADAIKAFGLELNENADKERSTLSLQFVRGLGESPLARSYEASHASIAENRELQSADSDRSTRHIEIALPPDVEYQEGDHLGVLPKNSQTNVSRILHRFGLKGTDQVTLSASGHSAGHLPLGRPVSLHDLLSYSVEVQEAATRAQIRELAAFTVCPPHRRELEELSAEGVYQEQILKKRISMLDLLEKYEACDMPFERFLELLRPLKPRYYSISSSPRVNPRLASITVGVVRGPAWSGRGEYRGVASNDLAERQAGDDLVMFVRTPESRFQLPEDPETPIIMVGPGTGVAPFRGFLQAREVLKREGKTLGEAHLYFGCRNDRDFIYRDELEQFEKDGIVTVHTAFSRKEGMPKTYVQHVMADHAETLISILDRGGRLYVCGDGSKMAPDVEAALQKAYQSVHGTGEQEAQNWLRHLQDTGMYAKDVWAGI

>CYP102A36_ortholog(BsLM_0764)Bacillus sp. LM 4-2

MKETSPIPQPKTFGPLGNLPLIDKDKPTLSLIKLAEEQGPIFQIHTPAGTTIVVSGHELVKEVCDEERFDKSIEGALEKVRAFSGDGLFTSWTHEPNWRKAHNILMPTFSQRAMKDYHEKMVDIAVQLIQKWARLNPNEAVDVPGDMTRLTLDTIGLCGFNYRFNSYYRETPHPFINSMVRALDEAMHQMQRLDVQDKLMVRTKRQFRHDIQTMFSLVDSIIAERRANGDQDEKDLLARMLNVEDPETGEKLDDENIRFQIITFLIAGHETTSGLLSFATYFLLKHPDKLKKAYEEVDRVLTDAAPTYKQVLELTYIRMILNESLRLWPTAPAFSLYPKADTVIGGKFPITTNDRISVLIPQLHRDRDAWGKDAEEFRPERFEHQDQVPHHAYKPFGNGQRACIGMQFALHEATLVLGMILKYFTLIDHENYELDIKQTLTLKPGDFHIRVQSRHLEAIHADVRAAEKAASDEQKEKTEAKGTSVIGLNNRPLLVLYGSDTGTAEGVARELADTASLHGVRTETAPLNDRIGKLPKEGAVVIVTSSYNGKPPSNAGQFVQWLQEIKPGELEGVHYAVFGCGDHNWASTYQYVPRFIDEQLAEKGATRFSARGEGDVSGDFEGQLDEWKKSMWADAIKAFGLELNENADKERSTLSLQFVRGLGESPLARSYEASHASIAENRELQSADSDRSTRHIEIALPPDVEYQEGDHLGVLPKNSQTNVNRILHRFGLKGTDQVTLSASGRSAGHLPLGRPVSLHDLLSYSVEVQEAATRAQIRELAAFTVCPPHKRELEELTAEGVYQEQILKKRISMLDLLEKYEACDMPFERFLELLRPLKPRYYSISSSPRVNPGQASITVGVVRGPAWSGRGEYRGVASNDLAERQAGDDVVMFIRTPESRFQLPKDPETPIIMVGPGTGVAPFRGFLQAREVLKREGKTLGEAHLYFGCRNDRDFIYRDELEQFEKDGIVTVHTAFSRKEGMPKTYVQHVMAAHAETLISIFDRGGRLYVCGDGSKMAPDVEAALQKAYQSVHGTGEQEAQNWLRHLQDTGMYAKDVWAGI

>CYP102A36_ortholog(A7A1_1768)Bacillus subtilis subsp. subtilis BSP1

MKETSPIPQPKTFGPLGNLPLIDKDKPTLSLIKLAEEQGPIFQIHTPAGTTIVVSGHELVKEVCDEERFDKSIEGALEKVRAFSGDGLFTSWTHEPNWRKAHNILMPTFSQRAMKDYHEKMVDIAVQLIQKWARLNPNEAVDVPGDMTRLTLDTIGLCGFNYRFNSYYRETPHPFINSMVRALDEAMHQMQRLDVQDKLMVRTKRQFHHDIQTMFSLVDSIIAERRSNGDQDEKDLLARMLNVEDPETGEKLDDENIRFQIITFLIAGHETTSGLLSFATYFLLKHPDKLKKAYEEVDRVLTDAAPTYKQVLELTYIRMILNESLRLWPTAPAFSLYPKEDTVIGGKFPITTNDRISVLIPQLHRDRDAWGKDAEEFRPERFEHQDQVPHHAYKPFGNGQRACIGMQFALHEATLVLGMILKYFTLIDHENYELDIKQTLTLKPGDFHIRVQSRNQDAIHADVQAAEKAASDEQKEKTEAKGTSVIGLNNRPLLVLYGSDTGTAEGVARELADTASLHGVRTETAPLNDRIGKLPKEGAVVIVTSSYNGKPPSNAGQFVQWLQEIKPGELEGVHYAVFGCGDHNWASTYQYVPRFIDEQLAEKGATRFSARGEGDVSGDFEGQLDEWKKSMWADAIKAFGLELNENADKERSTLSLQFVRGLGESPLARSYEASHASIAENRELQSADSDRSTRHIEIALPPDVEYQEGDHLGVLPKNSQTNVSRILHRFGLKGTDQVTLSASGHSAGHLPLGRPVSLHDLLSYSVEVQEAATRAQIRELAAFTVCPPHRRELEELSAEGVYQEQILKKRISMLDLLEKYEACDMPFERFLELLRPLKPRYYSISSSPRVNPRLASITVGVVRGPAWSGRGEYRGVASNDLAERQAGDDLVMFVRTPESRFQLPEDPETPIIMVGPGTGVAPFRGFLQAREVLKREGKTLGEAHLYFGCRNDRDFIYRDELEQFEKDGIVTVHTAFSRKEGIPKTYVQHVMADHAETLISILDRGGRLYVCGDGSKMAPDVEAALQKAYQSVHGTGEQEAQNWLRHLQDTGMYAKDVWAGI

>CYP102A36_ortholog(QF06_02530)Bacillus sp. YP1

MKETSPIPQPKTFGPLGNLPLIDKDKPTLSLIKLAEEQGPIFQIHTPAGTTIVVSGHELVKEVCDEERFDKSIEGALEKVRAFSGDGLFTSWTHEPNWRKAHNILMPTFSQRAMKDYHEKMVDIAVQLIQKWARLNPNEAVDVPGDMTRLTLDTIGLCGFNYRFNSYYRETPHPFINSMVRALDEAMHQMQRLDVQDKLMVRTKRQFRHDIQTMFSLVDSIIAERRANGDQDEKDLLARMLNVEDPETGEKLDDENIRFQIITFLIAGHETTSGLLSFATYFLLKHPDKLKKAYEEVDRVLTDAAPTYKQVLELKYIRMILNESLRLWPTAPAFSLYPKEDTVIGGKFPITTKDRISVLIPQLHRDRDAWGKDAEEFRPERFEHQDQVPHHAYKPFGNGQRACIGMQFALHEATLVLGMILKYFTLIDHENYELDIKQTLTLKPGDFHIRVQSRHQEAIHADAQADEKAAPYEQKEKTEAKGASVIGLNNRPLLVLYGSDTGTAEGVARELADTASLHGVRTETAPLNDRIGKLPKEGAVVIVTSSYNGKMPSNAGQFVQWLQEIKPGELEGVHYAVFGCGDHNWASTYQYVPRFIDEQLAEKGATRFSGRGEGDVSGDFEGQLDEWKKSMWADAIKAFGLELNENADKERSTLSLQFVRGLGESPLARSYEASHASIAENRELQSADSDRSTRHIEITLPPDVEYQEGDHLGVLPKNSQTNVSRILHRFGLKGTDQVTLSASGRSAGHLPLGRPVSLHDLLSYSVEVQEAATRAQIRELAAFTVCPPHRRELEELSAEGVYQEQILKKRISMLDLLEKYEACDMPFERFLELLRPLKPRYYSISSSPRVNPRLASITVGVVRGPAWSGRGEYRGVASNDLAERQAGDDLVMFVRTPESRFQLPEDPETPIIMVGPGTGVAPFRGFLQAREVLKREGKTLGEAHLYFGCRNDRDFIYRDELEQFEKDGIVTVHTAFSRKEGMPKTYVQHVMADHAETLISILDRGGRLYVCGDGSKMAPDVEAALQKAYQSVHGTGEQEAQNWLRHLQDTGMYAKDVWAGI

>CYP102A36_ortholog(I33_0816)Bacillus subtilis subsp. subtilis RO-NN-1

MKETSPIPQPKTFGPLGNLPLIDKDKPTLSLIKLAEEQGPIFQIHTPAGTTIVVSGHELVKEVCDEERFDKSIEGALEKVRAFSGDGLFTSWTHEPNWRKAHNILMPTFSQRAMKDYHEKMVDIAVQLIQKWARLNPNEAVDVPGDMTRLTLDTIGLCGFNYRFNSYYRETPHPFINSMVRALDEAMHQMQRLDFQDKLMVRTKRQFHHDIQTMFSLVDSIIAERRSNGDQDEKDLLARMLNVEDPETGEKLDDENIRFQIITFLIAGHETTSGLLSFATYFLLKHPDKLKKAYEEVDRVLTDAAPTYKQVLELKYIRMILNESLRLWPTAPAFSLYPKEDTVIGGKFPITTKDRISVLIPQLHRDRDAWGKDAEEFRPERFEHQDQVPHHAYKPFGNGQRACIGMQFALHEATLVLGMILKYFTLIDHENYELDIKQTLTLKPGDFHIRVQSRHQEAIHADVQAAEKAAPDEQKEKTEAKDASVIGLNNRLLLVLYGSDTGTAEGVARELADTASLHGVRTETAPLNDRIGKLPKEGAVVIVTSSYNGKPPSNAGQFVQWLQEIKLGELEGVHYAVFGCGDHNWASTYQDVPRFIDEQLAEKGATRFSARGEGDVSGDFEGQLDEWKKSMWADAIKAFGLELNENADKERSTLSLQFVRGLGESPLARSYEASHASIVENRELQSADSDRSTRHIEIALPPDVEYQEGDHLGILPKNSQTNVSRILHRFGLKGTDQVTLSASGRSAGHLPLGRPVSLHDLLSYSVEVQEAATRAQIRELAAFTVCPPHRRELEELTAEGVYQEQILKKRISMLDLLEKYEACDMPFERFLELLRPLKPRYYSISSSPRVNPRQASITVGVVRGPAWSGRGEYRGVASNDLAERQAGDDVVMFIRTPESRFQLPEDPETPIIMVGPGTGVAPFRGFLQAREVLKREGKKLGEAHLYFGCRNDRDFIYRDELEQFKKDGIVTVHTAFSRKEGMPKTYVQHLMADHAETLISILDRGGRLYVCGDGSKMAPDVEAALQKAYQSVHGTGEQEAQNWLRHLQDTGMYAKDVWAGI

>CYP102A36_ortholog(MY9_0816)Bacillus sp. JS

MKETSPIPQPKTFGPLGNLPLIDKDKPTLSLIKLAEEQGPIFQIHTPAGTTIVVSGHELVKEVCDEGRFDKSIEGALEKVRAFSGDGLFTSWTHEPNWRKAHNILMPTFSQRAMKDYHEKMVDIAVQLIQKWARLNPNEAVDVPGDMTRLTLDTIGLCGFNYRFNSYYRETPHPFINSMVRALDEAMHQMQRLDFQDKLMVRTKRQFHHDIQTMFSLVDSIIAERRANGDQDEKDLLARMLNVEDPETGEKLDDENIRFQIITFLIAGHETTSGLLSFAIYFLLKHPDKLKKAYEEVERVLTDAAPTYKQVLELKYIRMILNESLRLWPTAPAFSLYPKEDTVIGGKFPITTKDRISVLIPQLHRDRDAWGKDAEEFRPERFEHQDQVPHHAYKPFGNGQRACIGMQFALHEATLVLGMILKYFTLIDHENYELDIKQTLTLKPGDFRIKVQTRHQEAIHADIQATEKPGPDKQKEETETKGASVIGLNNRPLLVLYGSDTGTAEGVARELADTASLHGVRTEVSPLNDQIGKLPKEGAVVIVTSSYNGKPPSNARQFVQWLQEIKPGELEGVHYAVFGCGDHNWASTYQDVPRFIDEQLAEKGATRFSARGEGDVSGDFEGQLDEWKKSMWADAIKAFGLELNENADKERSTLSLQFVRGLGESPLARSYEAAHASIAENRELQSADSDRSTRHIEIALPPDVEYQEGDHLGVLPKNSPTNVSRILHRFGLKGTDQVTLLASGRSAGHLPLGRPVSLNDLLSYSVEVQEAATRAQIRELAAFTVCPPHKRELEELTAEGVYQEQILKKRISMLDLLEKYEACDMPFERFLELLRPLKPRYYSISSSPRVNPGQASITVGVVRGPAWSGRGEYRGVASNDLAERQAGDDVVMFIRTPESRFQLPKDPETPIIMVGPGTGVAPFRGFLQARDVLKREGKTLGEAHLYFGCRNDRDFIYRDELEQFEKDGVVTVHTAFSRKEGMPKTYVQHLMAEHAETLISILDRGGRLYVCGDGSKMAPDVEAALQKAYQSVHGTGEQEAQNWLRHLQDTGMYAKDVWAGI

>CYP102A36_ortholog(GYO_0986)Bacillus subtilis subsp. spizizenii TU-B-10

MKETSPIPQPKTYGPLGNLPLIDKDKPTLSLIKLAEEQGPIFQIHTPAGTTIVVSGHELVKEVCDEERFDKSIEGALEKVRAFSGDGLFTSWTHEPNWRKAHNILMPTFSQRAMKDYHEKMVDIAVQLIQKWARLNPNEAVDVPGDMTRLTLDTIGLCGFDYRFNSYYRETPHPFINSMVRALDEAMHQMQRLDFQDKLMVRTKRQFHHDIQTMFSLVDSIIAERRSNGDQDEKDLLARMLNVEDPETGEKLDDENIRFQIITFLIAGHETTSGLLSFAIYFLLKHPDKLEKAYEEVDRVLTGAAPTYKQVLELKYIRMILNESLRLWPTAPAFSLYPKEDTVISGKYPITTKDRISVLIPQLHRDQDAWGEDAEEFRPERFEHQDQVPHHAYKPFGNGQRACIGMQFALHEATLVLGMVLKYFTLIDHENYELDIKQTLTLKPGDFRIRVQTRHQEAIHADVPAAEKAAPDEQKEKTETKGASVIGLNNRPLLVLYGSDTGTAEGVARELADTASLHGVRTEVAPLNDRIGKLPKEGAVVIVTSSYNGKPPSNAGQFVQWLQEIKPGELEGVHYAVFGCGDHNWASTYQYVPRFIDEQLAEKGATRFSARGEGDVSGDFEGQLDEWKKSMWTDAIKAFGLELNENADKERSTLSLQFVRGLGESPLARSYEASHASISENRELQSADSDRSTRHIEITLPPDVEYREGDHLGVLPRNSQTNVSRILHRFGLKGTDQVTLSASGRSAGHLPLGRPVSLHDLLSYSVEVQEAASRAQIRELAAFTVCPPHKRELEDLATEGIYQEQILKKRISMLDLLEQYEACDMPFERFLELLRPLKPRYYSISSSPRVNPEQASITVGVVHGPAWSGRGEYRGVSSNYLAERQAGDDVVMFVRTPESRFQLPEDPETPIIMVGPGTGVAPFRGFLQARAALKREGKALGEAHLYFGCRNDHDFIYRDELEQFEKDGIVTVHTAFSRKEGMPKTYVQHLMADHAETLISILDRGGRLYVCGDGSKMAPDVEAGLQKAYQSVHGTGEQEAQNWLKHLQDTGIYAKDVWSGI

>CYP102A36_ortholog(BSUW23_03690)Bacillus subtilis subsp. spizizenii W23

MKETSPIPQPKTYGPLGNLPLIDKDKPTLSLIKLAEEQGPIFQIHTPAGTTIVVSGHELVKEVCDEERFDKSIEGALEKVRAFSGDGLFTSWTHEPNWRKAHNILMPTFSQRAMKDYHEKMVDIAVQLIQKWARLNPNEAVDVPGDMTRLTLDTIGLCGFNYRFNSYYRETPHPFINSMVRALDEAMHQMQRLDFQDKLMVRTKRQFHHDIQTMFSLVDSIIAERRSNGDQDEKDLLARMLNVEDPETGEKLDDENIRFQIITFLIAGHETTSGLLSFAIYFLLKHPDKLKKAYEEVDRVLTGAAPTYKQVLELKYIRMILNESLRLWPTAPAFSLYPKEDTVIGGKYMITTQDRISVLIPQLHRDQDAWGEDAEEFRPERFEHQDQVPHHAYKPFGNGQRACIGMQFALHEATLVLGMVLKYFTLIDHENYELDIKQTLTLKPGDFRIRVQTRHQEAIHTDVPAAEKEAPVEQKEETETKGASVIGLNNRPLLVLYGSDTGTAEGVARELADTASLHGVRTEVAPLNDRIGKLPKEGAVVIVTASYNGKPPSNAGQFVQWLQEIKPGELEGVHYAVFGCGDHNWASTYQYVPRFIDEQLAEKGATRFSERGEGDVSGDFEGQLDEWKKSMWTDAIKAFGLELNENADKERSTLSLQFVRGLGESPLARSYEAAHASIAENRELQSADSDRSTRHIEIALPPDVEYREGDHLGVLPRNSQTNVSRILHRFGLKGTDQVTLSASGRSAGHLPLGRPVSLHDLLSYSVEVQEAATRAQIRELAAFTVCPPHKRELEDMTEEGVYQEQILKKRISMLDLLEQYESCEMPFERFLELLRPLKPRYYSISSSPRVNPEQASITVGVVRGPAWSGRGEYRGVSSSYLAERQAGDDVVMFVRTPESRFQLPEDPETPIIMVGPGTGVAPFRGFLQARAALKREGKALGEAHLYFGCRNDHDFIYRDELEQFEKDGIVTVHTAFSRKEGMPKTYVQHLMADHAETLISILDRGGRLYVCGDGSKMAPDVEAGLQKAYQSVHGTGEEEAQNWLKHLQDTGIYAKDVWSGV

>CYP102A37(B0X71_13980)Paenibacillus mucilaginosus 3016

MKDTAVIPQPKTYGPLGNIPLIDRDKPILSLMELAEEFGPIFRIQTPVGRSIVVSGHELVKEVCDESRFDKSVEGALAKVRAFTGDGLFTSWTHEPNWHKAHNILMPTFSQRAMKDYHGMMVDIADQLVQKWARLNPNEPVDVPEDMTRLTLDTIGLCGFNYRFNSYYRETPHPFITSMTRALDEAMHQLQRLDVQDKLMVITKRQFNHDIKAMFSLVDSLIAERKERGDQGENDLLARMLNVDDPETGDKLDDENIRFQIITFLIAGHETTSGLLSFALYFLLNNPDKLQKAYEEVDQVFSGSTPSYKEVTQLKYIRMVLNESLRLWPTAPAFSLYAKEDTVIGGEYPIQKEADRITVLIPQLHRDKEAWGEDAEAFRPERFEDPKQVPHHAYKPFGNGQRACIGMQFALHEAALVLGMLLKHFEFVDHSDYELDVKQTLTLKPGDFTIQVKPRTSAPGTPSVAVAEEKDNTELAAEDPAVQNVSVQGLNNRPLLVLYGSDTGTAEGVARELADTATLRGIRTEVASLDDRIGKLPKEGALLIITSSYNGKPPSNADQFVQWLEESKPGELAGVQYAVFGCGDHNWASTYQDVPRFIDEQLAEKGAVRFSARGEGDVSSDFEEQLDQWKQTMWMEAIDAFDLKVSENLEKEPNTLSLQFVKGIGGSPLAHSYEAVHATIAVNQELQAAESERSTRHIEVTLPEGITYQEGDHLGVLPANSRENVGRILRRFSLNGNDHVLLSASGRSAAHLPLNQPVSLHDVLTFSVEVQEAATRAQIRELVSFTTCPPHKRELEALLEDGVYQENILKKRVSMLELLEKYEACEMPFERFLDSLPALKPRYYSISSSPLAAQSRLSITVGVVEGPAWSGRAEYKGVASNYLAHRQLGDDILCFVRTPQTNFQLPTDLETPIIMVGPGTGIAPFRGFLQARRIQQQQDKRLGEAHLYFGCRHPEKDFLYRTELEQGEKDGILSLHTAFSRLEGQPRTYVQDLIEQDGAKLISLLDSGARLYVCGDGSRMAPDVEGALQQAYEQQHSATEQQAKEWLEELQRTGQYAKDVWAGV

>CYP102A38(RBAU_0722)Bacillus velezensis UCMB5033

MLERVLFMKETGPIPQPKTFGPLGNLPLLDKDKPTMSLIKLANEQGPIFQLHTPAGAIIVVSGHELVKEVCDEERFDKSIEGALEKVRAFSGDGLFTSWTHEPNWRKAHNILMPTFSQRAMKDYHSMMTDIAVQLIQKWARLNPDEAVDVPADMTRLTLDTIGLCGFNYRFNSYYRETPHPFINSMVRALDEAMHQMQRLDVQDKLMIRTKRQFHHDIQAMFSLVDSIIAERRSGGRDEKDLLARMLNVEDPETGEKLDDENIRFQIITFLIAGHETTSGLLSFAIYFLLKHPRVLEKAYEEADRVLTDPVPSYKQVLDLTYIRMILQESLRLWPTAPAFSLYAKEDTVIGGKYPITPKDRISVLIPQLHRDKDAWGDNAEEFYPERFEHPDQVPHHAYKPFGNGQRACIGMQFALHEATLVLGMILQHFTFIDHTDYELDIKQTLTIKPGDFHIRVRPRNKEAVAAALPAAEKAAEDVEKEKRETKGASIIGLDNRPLLILYGSDTGTAEGVARELADTAGMHGVRTETAPLNDRIGKLPKEGALLIITSSYNGKPPSNAGQFVQWLEEVKPGELEGVRYAVFGCGDHNWAATYQAVPRLIDEKLAEKGAERFSSRGEGDVSGDFEGKLDEWKKSMWTDAMKAFGLKLNENAEKERSALGLQFVSGLGGSPLAQTYEAVYASVAENRELQAPESGRSTRHIEITLPKEAAYHEGDHLGVLPVNSKEQVSRVLRRFNLNGNDQVLLTASGQSAAHLPLDRPVRLHDLLSSCVELQEAASRAQIREMAAYTVCPPHKRELEDFLEEGVYQEQILTSRVSMLDLLEKYEACELPFERFLELLRPLKPRYYSISSSPRKHPGQASITVGVVRGPARSGLGEYRGVASNYLADRGPEDGIVMFVRTPETRFRLPEDPEKPIIMVGPGTGVAPFRGFLQARAALKKEGKELGEAHLYFGCRNDHDFIYRDELEAYEKDGIVTLHTAFSRKEGVPKTYVQHLMAKDAGALISILGRGGHLYVCGDGSKMAPDVEATLQKAYQSVHETDERQAQEWLLDLQTKGIYAKDVWAGI

>CYP102A38_ortholog(BASU_0699)Bacillus velezensis UCMB5113

MLERVLFMKETGPIPQPKTFGPLGNLPLLDKDKPTMSLIKLANEQGPIFQLHTPAGAIIVVSGHELVKEVCDEERFDKSIEGALEKVRAFSGDGLFTSWTHEPNWRKAHNILMPTFSQRAMKDYHSMMTDIAVQLIQKWARLNPDEAVDVPADMTRLTLDTIGLCGFNYRFNSYYRETPHPFINSMVRALDEAMHQMQRLDVQDKLMIRTKRQFHHDIQAMFSLVDSIIAERRSGGRDEKDLLARMLNVEDPETGEKLDDENIRFQIITFLIAGHETTSGLLSFAIYFLLKHPRVLEKAYEEADRVLTDPVPSYKQVLDLTYIRMILQESLRLWPTAPAFSLYAKEDTVIGGKYPITPKDRISVLIPQLHRDKDAWGDNAEEFYPERFEHPDQVPHHAYKPFGNGQRACIGMQFALHEATLVLGMILQHFTFIDHTDYELDIKQTLTIKPGDFHIRVRPRNKEAVAAALPAAEKAAEDVEKEKRETKGASIIGLDNRPLLILYGSDTGTAEGVARELADTAGMHGVRTETAPLNDRIGKLPKEGALLIITSSYNGKPPSNAGQFVQWLEEVKPGELEGVRYAVFGCGDHNWAATYQAVPRLIDEKLAEKGAERFSSRGEGDVSGDFEGKLDEWKKSMWTDAMKAFGLKLNENAEKERSALGLQFVSGLGGSPLAQTYEAVYASVAENRELQAPESGRSTRHIEITLPKEAAYHEGDHLGVLPVNSKEQVSRVLRRFNLNGNDQVLLTASGQSAAHLPLDRPVRLHDLLSSCVELQEAASRAQIREMAAYTVCPPHKRELEDFLEEGVYQEQILTSRVSMLDLLEKYEACELPFERFLELLRPLKPRYYSISSSPRKHPGQASITVGVVRGPARSGLGEYRGVASNYLADRGPEDGIVMFVRTPETRFRLPEDPEKPIIMVGPGTGVAPFRGFLQARAALKKEGKELGEAHLYFGCRNDHDFIYRDELEAYEKDGIVTLHTAFSRKEGVPKTYVQHLMAKDAGALISILGRGGHLYVCGDGSKMAPDVEATLQKAYQSVHETDERQAQEWLLDLQTKGIYAKDVWAGI

>CYP102A38_ortholog(MUS_0726)Bacillus amyloliquefaciens Y2

MLERVLFMKETGPIPQPKTFGPLGNLPLLDKDKPTMSLIKLANEQGPIFQLHTPAGAIIVVSGHELVKEVCDEERFDKSIEGALEKVRAFSGDGLFTSWTHEPNWRKAHNILMPTFSQRAMKDYHSMMTDIAVQLIQKWARLNPDEAVDVPADMTRLTLDTIGLCGFNYRFNSYYRETPHPFINSMVRALDEAMHQMQRLDVQDKLMIRTKRQFHHDIQAMFSLVDSIIAERRSGGRDEKDLLARMLNVEDPETGEKLDDENIRFQIITFLIAGHETTSGLLSFAIYFLLKHPRVLEKAYEEADRVLTDPVPSYKQVLDLTYIRMILQESLRLWPTAPAFSLYAKEDTVIGGKYPITPKDRISVLIPQLHRDKDAWGDNAEEFYPERFEHPDQVPHHAYKPFGNGQRACIGMQFALHEATLVLGMILQHFTFIDHTDYELDIKQTLTIKPGDFHIRVRPRNKGAVAAALPAAEKAAEDVEKEKRETKGASIIGLDNRPLLILYGSDTGTAEGVARELADTAGMHGVRTETAPLNDRIGKLPKEGALLIITSSYNGKPPSNAGQFVQWLEEVKPGELEGVRYAVFGCGDHNWAATYQAVPRLIDEKLAEKGAERFSSRGEGDVSGDFEGKLDEWKKSMWTDAMKAFGLKLNENAEKERSSLGLQFVSGLGGSPLAQTYEAVYASVAENRELQAPESGRSTRHIEITLPKEAAYHEGDHLGVLPVNSKEQVSRVLRRFNLNGNDQVLLTASGQSAAHLPLDRPVRLHDLLSSCVELQEAASRAQIREMAAYTVCPPHKRELEDFLEEGVYQEKILTLRVSMLDLLEKYEACELPFERFLELLRPLKPRYYSISSSPRKHPGQASITVGVVRGPARSGLGEYRGVASNYLADRGPEDGIVMFVRTPETRFRLPEDPEKPIIMVGPGTGVAPFRGFLQARAALKKEGKELGEAHLYFGCRNDHDFIYRDELEAYEKDGIVTLHTAFSRKEGVPKTYVQHLMAKDAGALISILGRGGHLYVCGDGSKMAPDVEATLQKAYQSVHETDERQAQEWLLDLQTKGIYAKDVWAGI

>CYP102A38_ortholog(BAM5036_0659)Bacillus velezensis UCMB5036

MLERVLFMKETGPIPQPKTFGPLGNLPLLDKDKPTMSLIKLANEQGPIFQLHTPAGAIIVVSGHELVKEVCDEERFDKSIEGALEKVRAFSGDGLFTSWTHEPNWRKAHHILMPTFSQRAMKDYHSMMTDIAVQLIQKWARLNPDEAVDVPADMTRLTLDTIGLCGFNYRFNSYYRETPHPFINSMVRALDEAMHQMQRLDVQDKLMIRTKRQFHHDIQAMFSLVDSIIAERRSGGRDEKDLLARMLNVEDPETGEKLDDENIRFQIITFLIAGHETTSGLLSFAIYFLLKHPRVLEKAYEEADRVLTDPVPSYKQVLDLTYIRMILQESLRLWPTAPAFSLYAKEDTVIGGKYPITPKDRISVLIPQLHRDKDAWGDNAEEFYPERFEHPDQVPHHAYKPFGNGQRACIGMQFALHEATLVLGMILQHFTFIDHTDYELDIKQTLTIKPGDFHIRVRPRNKEAVAAALPAAEKAAEDVEKEKRETKGASIIGLDNRPLLILYGSDTGTAEGVARELADTAGMHGVRTETAPLNDRIGKLPKEGALLIITSSYNGKPPSNAGQFVQWLEEVKPGELEGVRYAVFGCGDHNWAATYQAVPRLIDEKLAEKGAERFSSRGEGDVSGDFEGKLDEWKKSMWTDAIKAFGLKLNENAEKERSALGLQFVSGLGGSPLAQTYEAVYASVAENRELQAPESGRSTRHIEITLPKEAAYHEGDHLGVLPVNSKEQVSRVLRRYNLNGNDQVLLTASGQSAAHLPLDRPVRLHDLLSSCVELQEAASRAQIREMAAYTVCPPHKRELEDFLEEGFYQEQILTSRVSMLDLLEKYEACELPFERFLELLRPLKPRYYSISSSPRKHPGQASITVGVVRGPARSGLGEYRGVASNYLADRGPEDGIVMFVRTPETRFRLPEDPEKPIIMVGPGTGVAPFRGFLQARAALKKEGKELGEAHLYFGCRNDHDFIYRDELEAYEKDGIVTLHTAFSRKEGVPKTYVQHLMAKDAGALISILGRGGHLYVCGDGSKMAPDVEATLQKAYQSVHETDERQAQEWLLNLQTKGIYAKDVWAGI

>CYP102A38_ortholog(NG74_00734)Bacillus velezensis

MLERVLFMKETGPIPQPKTFGPLGNLPLLDKDKPTMSLIKLANEQGPIFQLHTPAGAIIVVSGHELVKEVCDEERFDKSIEGALEKVRAFSGDGLFTSWTHEPNWRKAHNILMPTFSQRAMKDYHSMMTDIAVQLIQKWARLNPDEAVDVPADMTRLTLDTIGLCGFNYRFNSYYRETPHPFINSMVRALDEAMHQMQRLDVQDKLMIRTKRQFHHDIQAMFSLVDSIIAERRSGGRDEKDLLARMLNVEDPETGEKLDDKNIRFQIITFLIAGHETTSGLLSFAIYFLLKHPRVLDKAYEEADRVLTDPVPSYKQVLDLTYIRMILQESLRLWPTAPAFSLYAKEDTVIGGKYPITPKDRISVLIPQLHRDKDAWGDNAEEFYPERFEHPDQVPQHAYKPFGNGQRACIGMQFALHEATLVLGMILQHFTFIDHTDYELDIKQTLTIKPGDFHIRVRPRNKGAVAAALPAAEKAAGDVEKEKRETKGASIIGLDNRPLLILYGSDTGTAEGVARELADTAGMHGVRTETAPLNDRIGKLPKEGALLIITSSYNGKPPSNAGQFVQWLEEVKPGELEGVRYAVFGCGDHNWAATYQAVPRLIDEKLAEKGAERFSSRGEGDVSGDFEGKLDEWKKSMWTDAMKAFGLKLNENAEKERSSLGLQFVSGLGGSPLAKTYEAVYASVAENRELQAPESGRSTRHIEITLPKEAAYHEGDHLGVLPVNSKEQVSRVLRRFNLNGNDQVLLTASGQSAAHLPLDRPVRLHDLLSSCVELQEAASRAQIREMAAYTVCPPHKRELEDFLEEGVYQEQILTLRVSMLDLLEKYEACELPFERFLELLRPLKPRYYSISSSPRKHPGQASITVGVVCGPARSGLGEYRGVASNYLADRGPEDGIVMFVRTPETRFRLPEDPEKPIIMVGPGTGVAPFRGFLQARAALKKEGKELGEAHLYFGCRNDHDFIYRDELEAYEKDGIVTLHTAFSRKEGVPKTYVQHLMAKDAGALISILGRGGHLYVCGDGSKMAPDVEATLQKAYQSVHETDERQAQEWLLDLQTKGIYAKDVWAGI

>CYP102A38_ortholog(OY17_06355)Bacillus sp. BH072

MKETGPIPQPKTFGPLGNLPLLDKDKPTMSLIKLANEQGPIFQLHTPAGAIIVVSGHELVKEVCDEERFDKSIEGALEKVRAFSGDGLFTSWTHEPNWRKAHNILMPTFSQRAMKDYHSMMTDIAVQLIQKWARLNPDEAVDVPADMTRLTLDTIGLCGFNYRFNSYYRETPHPFINSMVRALDEAMHQMQRLDVQDKLMIRTKRQFHHDIQAMFSLVDSIIAERRSGGRDEKDLLARMLNVEDPETGEKLDDENIRFQIITFLIAGHETTSGLLSFAIYFLLKHPRVLEKAYEEADRVLTDPVPSYKQVLDLTYIRMILQESLRLWPTAPAFSLYAKEDTVIGGKYPITPKDRISVLIPQLHRDKDAWGDNAEEFYPERFEHPDQVPHHAYKPFGNGQRACIGMQFALHEATLVLGMILQHFTFIDHTDYELDIKQTLTIKPGDFHIRVRPRNKGAVAAALPAAEKAAEDVEKEKRETKGASIIGLDNRPLLILYGSDTGTAEGVARELADTAGMHGVRTETAPLNDRIGKLPKEGALLIITSSYNGKPPSNAGQFVQWLEEVKPGELEGVRYAVFGCGDHNWAATYQAVPRLIDEKLAEKGAERFSSRGEGDVSGDFEGKLDEWKKSMWTDAMKAFGLKLNENAEKERSSLGLQFVSGLGGSPLAQTYEAVYASVAENRELQAPESGRSTRHIEITLPKEAAYHEGDHLGVLPVNSKEQVSRVLRRFNLNGNDQVLLTASGQSAAHLPLDRPVRLHDLLSSCVELQEAASRAQIREMAAYTVCPPHKRELEDFLEEGVYQEKILTLRVSMLDLLEKYEACELPFERFLELLRPLKPRYYSISSSPRKHPGQASITVGVVRGPARSGLGEYRGVASNYLADRGPEDGIVMFVRTPETRFRLPEDPEKPIIMVGPGTGVAPFRGFLQARAALKKEGKELGEAHLYFGCRNDHDFIYRDELEAYEKDGIVTLHTAFSRKEGVPKTYVQHLMAKDAGALISILGRGGHLYVCGDGSKMAPDVEATLQKAYQSVHETDERQAQEWLLDLQTKGIYAKDVWAGI

>CYP102A38_ortholog(V529_06850)Bacillus velezensis SQR9

MKETGPIPQPKTFGPLGNLPLLDKDKPTMSLIKLANEQGPIFQLHTPAGAIIVVSGHELVKEVCDEERFDKSIEGALEKVRAFSGDGLFTSWTHEPNWRKAHNILMPTFSQRAMKDYHSMMTDIAVQLIQKWARLNPDEAVDVPADMTRLTLDTIGLCGFNYRFNSYYRETPHPFINSMVRALDEAMHQMQRLDVQDKLMIRTKRQFHHDIQAMFSLVDSIIAERRSGGRDEKDLLARMLNVEDPETGEKLDDENIRFQIITFLIAGHETTSGLLSFAIYFLLKHPRVLEKAYEEADRVLTDPVPSYKQVLDLTYIRMILQESLRLWPTAPAFSLYAKEDTVIGGKYPITPKDRISVLIPQLHRDKDAWGDNAEEFYPERFEHPDQVPHHAYKPFGNGQRACIGMQFALHEATLVLGMILQHFTFIDHTDYELDIKQTLTIKPGDFHIRVRPRNKEAVAAALPAAEKAAEDVEKEKRETKGASIIGLDNRPLLILYGSDTGTAEGVARELADTAGMHGVRTETAPLNDRIGKLPKEGALLIITSSYNGKPPSNAAQFVQWLEEVKPGELEGVRYAVFGCGDHNWAATYQAVPRLIDEKLAEKGAERFSSRGEGDVSGDFEGKLDEWKKSMWTDAMKVFGLKLNENAEKERSALGLQFVSGLGGSPLAQTYEAVYASVAENRELQAPESGRSTRHIEITLPKEAAYHEGDHLGVLPVNSKEQVRRVLRRFNLNGNDQVLLTASGQSAAHLPLDRPVRLHDLLSSCVELQEAASRAQIREMAAYTVCPPHKRELEDFLEEGVYQEQILTSRVSMLDLLEKYEACELPFERFLELLRPLKPRYYSISSSPRKNPGQASITVGVVRGPARSGLGEYRGVASNYLADRGPEDGIVMFVRTPETRFRLPEDPEKPIIMVGPGTGVAPFRGFLQARAALKKEGKELGEAHLYFGCRNDHDFIYRDELEAYEKDGIVTLHTAFSRKEGVPKTYVQHLMAKDAGALISILGRGGHLYVCGDGSKMAPDVEATLQKAYQSVHETDERQAQEWLLDLQTKGIYAKDVWAGI

>CYP102A38_ortholog(BANAU_0661)Bacillus velezensis YAU B9601-Y2

MKETGPIPQPKTFGPLGNLPLLDKDKPTMSLIKLANEQGPIFQLHTPAGAIIVVSGHELVKEVCDEERFDKSIEGALEKVRAFSGDGLFTSWTHEPNWRKAHNILMPTFSQRAMKDYHSMMTDIAVQLIQKWARLNPDEAVDVPADMTRLTLDTIGLCGFNYRFNSYYRETPHPFINSMVRALDEAMHQMQRLDVQDKLMIRTKRQFHHDIQAMFSLVDSIIAERRSGGRDEKDLLARMLNVEDPETGEKLDDENIRFQIITFLIAGHETTSGLLSFAIYFLLKHPRVLEKAYEEADRVLTDPVPSYKQVLDLTYIRMILQESLRLWPTAPAFSLYAKEDTVIGGKYPITPKDRISVLIPQLHRDKDAWGDNAEEFYPERFEHPDQVPHHAYKPFGNGQRACIGMQFALHEATLVLGMILQHFTFIDHTDYELDIKQTLTIKPGDFHIRVRPRNKGAVAAALPAAEKAAEDVEKEKRETKGASIIGLDNRPLLILYGSDTGTAEGVARELADTAGMHGVRTETAPLNDRIGKLPKEGALLIITSSYNGKPPSNAGQFVQWLEEVKPGELEGVRYAVFGCGDHNWAATYQAVPRLIDEKLAEKGAERFSSRGEGDVSGDFEGKLDEWKKSMWTDAMKAFGLKLNENAEKERSSLGLQFVSGLGGSPLAQTYEAVYASVAENRELQAPESGRSTRHIEITLPKEAAYHEGDHLGVLPVNSKEQVSRVLRRFNLNGNDQVLLTASGQSAAHLPLDRPVRLHDLLSSCVELQEAASRAQIREMAAYTVCPPHKRELEDFLEEGVYQEKILTLRVSMLDLLEKYEACELPFERFLELLRPLKPRYYSISSSPRKHPGQASITVGVVRGPARSGLGEYRGVASNYLADRGPEDGIVMFVRTPETRFRLPEDPEKPIIMVGPGTGVAPFRGFLQARAALKKEGKELGEAHLYFGCRNDHDFIYRDELEAYEKDGIVTLHTAFSRKEGVPKTYVQHLMAKDAGALISILGRGGHLYVCGDGSKMAPDVEATLQKAYQSVHETDERQAQEWLLDLQTKGIYAKDVWAGI

>CYP102A38_ortholog(AJ82_04120)Bacillus velezensis TrigoCor1448

MKETGPIPQPKTFGPLGNLPLLDKDKPTMSLIKLANEQGPIFQLHTPAGAIIVVSGHELVKEVCDEERFDKSIEGALEKVRAFSGDGLFTSWTHEPNWRKAHHILMPTFSQRAMKDYHSMMTDIAVQLIQKWARLNPDEAVDVPADMTRLTLDTIGLCGFNYRFNSYYRETPHPFINSMVRALDEAMHQMQRLDVQDKLMIRTKRQFHHDIQAMFSLVDSIIAERRSGGRDEKDLLARMLNAEDPETGEKLDDENIRFQIITFLIAGHETTSGLLSFAIYFLLKHPRVLEKAYEEADRVLTDPVPSYKQVLDLTYIRMILQESLRLWPTAPAFSLYAKEDTVIGGKYPITPKDRISVLIPQLHRDKDAWGDNAEEFYPERFEHPDQVPHHAYKPFGNGQRACIGMQFALHEATLVLGMILQHFTFIDHTDYELDIKQTLTIKPGDFHIRVRPRNKEDVAAALPAAEKAAEDVEKEKRETKGASIIGLDNRPLLILYGSDTGTAEGVARELADTAGMHGVRTETAPLNDRIGKLPKEGALLIITSSYNGKPPSNAGQFVQWLEEVKPGELEGVRYAVFGCGDHNWAATYQAVPRLIDEKLAEKGAERFSSRGEGDVSGDFEGKLDEWKKSMWTDAMKAFGLKLNENAEKERSALGLQFVSGLGGSPLAQTYEAVYASVAENRELQAPESGRSTRHIEITLPKEAAYHEGDHLGVLPVNSKEQVSRVLRRFNLNGNDQVLLTASGQSAAHLPLDRPVRLHDLLSSCVELQEAASRAQIREMAAYTVCPPHKRELEDFLEEGVYQEQILTSRVSMLDLLEKYEACELPFERFLELLRPLKPRYYSISSSPRKHPGQASITVGVVRGPARSGLGEYRGVASNYLADRGPEDGIVMFVRTPETRFRLPEDPEKPIIMVGPGTGVAPFRGFLQARAALKKEGKELGEAHLYFGCRNDHDFIYRDELEAYEKDGIVTLHTAFSRKEGVPKTYVQHLMAKDAGALISILGRGGHLYVCGDGSKMAPDVEATLQKAYQSVRETDERQAQEWLLDLQTKGIYAKDVWAGI

>CYP102A38_ortholog(SB24_06155)Bacillus sp. Pc3

MKETGPIPQPKTFGPLGNLPLLDKDKPTMSLIKLANEQGPIFQLHTPAGAIIVVSGHELVKEVCDEERFDKSIEGALEKVRAFSGDGLFTSWTHEPNWRKAHHILMPTFSQRAMKDYHSMMTDIAVQLIQKWARLNPDEAVDVPADMTRLTLDTIGLCGFNYRFNSYYRETPHPFINSMVRALDEAMHQMQRLDVQDKLMIRTKRQFHHDIQAMFSLVDSIIAERRSGGRDEKDLLARMLNVEDPETGEKLDDENIRFQIITFLIAGHETTSGLLSFAIYFLLKHPRVLEKAYEEADRVLTDPVPSYKQVLELSYIRMILQESLRLWPTAPAFSLYAKEDTVIGGKYPITPKDRISVLIPQLHRDKDAWGDNAEEFYPERFEHPDQVPHHAYKPFGNGQRACIGMQFALHEATLVLGMILQHFTFIDHTDYELDIKQTLTIKPGDFHIRVRPRNKEAVAAALPAAEKAAEDVEKEKRETKGASIIGLDNRPLLILYGSDTGTAEGVARELADTAGMHGVRTETAPLNDRIGKLPKEGALLIITSSYNGKPPSNAGQFVQWLEEVKPGELEGVRYAVFGCGDHNWAATYQAVPRLIDEKLAEKGAERFSSRGEGDVSGDFEGKLDEWKKSMWTDAMKAFGLKLNENAEKERSALGLQFVSGLGGSPLAQTYEAVYASVAENRELQAPESGRSTRHIEITLPKEAAYHEGDHLGVLPVNSKEQVSRVLRRYNLNGNDQVLLTASGQSAAHLPLDRPVRLHDLLSSCVELQEAASRAQIREMAAYTVCPPHKRELEDFLEEGVYHEQILTSRVSMLDLLEKYEACELPFERFLELLRPLKPRYYSISSSPRKHPGQASITVGVVRGPARSGLGEYRGVASNYLADRGPEDGIVMFVRTPETRFRLPEDSEKPIIMVGPGTGVAPFRGFLQARAALKKEGKELGEAHLYFGCRNDHDFIYRDELEAYEKDGIVTLHTAFSRKEGVPKTYVQHLMAKDAGALISILGRGGHLYVCGDGSKMAPDVEATLQKAYKSVHETDERQAQEWLLDLQTKGIYAKDVWAGI

>CYP102A38_ortholog(U471_07160)Bacillus amyloliquefaciens CC178

MKETGPIPQPKTFGPLGNLPLLDKDKPTMSLIKLANEQGPIFQLHTPAGAIIVVSGHELVKEVCDEERFDKSIEGALEKVRAFSGDGLFTSWTHEPNWRKAHHILMPTFSQRAMKDYHSMMTDIAVQLIQKWARLNPDEAVDVPADMTRLTLDTIGLCGFNYRFNSYYRETPHPFINSMVRALDEAMHQMQRLDVQDKLMIRTKRQFHHDIQAMFSLVDSIIAERRSGGRDEKDLLARMLNVEDPETGEKLDDENIRFQIITFLIAGHETTSGLLSFAIYFLLKHPRVLEKAYEEADRVLTDPVPSYKQVLDLTYIRMILQESLRLWPTAPAFSLYAKEDTVIGGKYPITPKDRISVLIPQLHRDKDAWGDNAEEFYPERFEHPDRVPHHAYKPFGNGQRACIGMQFALHEATLVLGMILQHFTFIDHTDYELDIKQTLTIKPGDFHIRVRPRNKEDVAAALPAAEKAAEDVGKEKRETKGASIIGLDNRPLLILYGSDTGTAEGVARELADTAGMHGVRTETAPLNDRIGKLPKEGALLIITSSYNGKPPSNAGQFVQWLEEVKPGELEGVRYAVFGCGDHNWAATYQAVPRLIDEKLAEKGAERFSSRGEGDVSGDFEGKLDEWKKSMWTDAMKAFGLKLNENAEKERSALGLQFVSGLGGSPLAQTYEAVYASVAENRELQAPESGRSTRHIEITLPKEAAYHEGDHLGVLPVNSKEQVSRVLRRFNLNGNDQVLLTASGQSAAHLPLDRPVRLHDLLSSCVELQEAASRAQIREMAAYTVCPPHKRELEDFLEEGVYQEQILTSRVSMLDLLEKYEACELPFERFLELLRPLKPRYYSISSSPRKHPGQASITVGVVRGPARSGLGEYRGVASNYLADRGPEDGIVMFVRTPETRFRLPEDPEKPIIMVGPGTGVAPFRGFLQARAALKKEGKELGEAHLYFGCRNDHDFIYRDELEAYEKDGIVTLHTAFSRKEGVPKTYVQHLMAKDAGALISILGRGGHLYVCGDGSKMAPDVEATLQKAYQSVRETDERQAQEWLLDLQTKGIYAKDVWAGI

>CYP102A38_ortholog(RBAM_007390)Bacillus velezensis FZB42

MKETGPIPQPKTFGPLGNLPLLDKDKPTMSLIKLANEQGPIFQLHTPAGAIIVVSGHELVKEVCDEERFDKSIEGALEKVRAFSGDGLFTSWTHEPNWRKAHHILMPTFSQRAMKDYHSMMTDIAVQLIQKWARLNPDEAVDVPADMTRLTLDTIGLCGFNYRFNSYYRETPHPFINSMVRALDEAMHQMQRLDVQDKLMIRTKRQFHHDIQAMFSLVDSIIAERRSGGRDEKDLLARMLNVEDPETGEKLDDENIRFQIITFLIAGHETTSGLLSFAIYFLLKHPRVLEKAYEEADRVLTDPVPSYKQVLDLTYIRMILQESLRLWPTAPAFSLYAKEDTVIGGKYPITPKDRISVLIPQLHRDKDAWGDNAEEFYPERFEHPDRVPHHAYKPFGNGQRACIGMQFALHEATLVLGMILQHFTFIDHTDYELDIKQTLTIKPGDFHIRVRPRNKEDVAAALPAAEKAAEDVGKEKRETKGASIIGLDNRPLLILYGSDTGTAEGVARELADTAGMHGVRTETAPLNDRIGKLPKEGALLIITSSYNGKPPSNAGQFVQWLEEVKPGELEGVRYAVFGCGDHNWAATYQAVPRLIDEKLAEKGAERFSSRGEGDVSGDFEGKLDEWKKSMWTDAMKAFGLKLNENAEKERSALGLQFVSGLGGSPLAQTYEAVYASVAENRELQAPESGRSTRHIEITLPKEAAYHEGDHLGVLPVNSKEQVSRVLRRFNLNGNDQVLLTASGQSAAHLPLDRPVRLHDLLSSCVELQEAASRAQIREMAAYTVCPPHKRELEDFLEEGVYQEQILTSRVSMLDLLEKYEACELPFERFLELLRPLKPRYYSISSSPRKHPGQASITVGVVRGPARSGLGEYRGVASNYLADRGPEDGIVMFVRTPETRFRLPEDPEKPIIMVGPGTGVAPFRGFLQARAALKKEGKELGEAHLYFGCRNDHDFIYRDELEAYEKDGIVTLHTAFSRKEGVPKTYVQHLMAKDAGALISILGRGGHLYVCGDGSKMAPDVEATLQKAYQSVRETDERQAQEWLLDLQTKGIYAKDVWAGI

>CYP102A38_ortholog(B938_03495)Bacillus velezensis AS43.3

MKETGPIPQPKTFGPLGNLPLLDKDKPTMSLIKLANEQGPIFQLHTPAGAIIVVSGHELVKEVCDEERFDKSIEGALEKVRAFSGDGLFTSWTHEPNWRKAHHILMPTFSQRAMKDYHSMMTDIAVQLIQKWARLNPDEAVDVPADMTRLTLDTIGLCGFNYRFNSYYRETPHPFINSMVRALDEAMHQMQRLDVQDKLMIRTKRQFHHDIQAMFSLVDSIIAERRSGGRDEKDLLARMLNVEDPETGEKLDDENIRYQIITFLIAGHETTSGLLSFAIYFLLKHPRVLEKAYEEADRVLTDPVPSYKQVLDLTYIRMILQESLRLWPTAPAFSLYAKEDTVIGGKYPITPKDRISVLIPQLHRDKDAWGDNAEEFYPERFEHPDRVPHHAYKPFGNGQRACIGMQFALHEATLVLGMILQHFTFIDHTDYELDIKQTLTIKPGDFHIRVRPRNKEDVAAALPAAEKAAEDVGKEKRETKGASIIGLDNRPLLILYGSDTGTAEGVARELADTAGMHGVRTETAPLNDRIGKLPKEGALLIITSSYNGKPPSNAGQFVQWLEEVKPGELEGVRYAVFGCGDHNWAATYQAVPRLIDEKLAEKGAERFSSRGEGDVSGDFEGKLDEWKKSMWTDAMKAFGLKLNENAEKERSALGLQFVSGLGGSPLAQTYEAVYASVAENRELQAPESGRSTRHIEITLPKEAAYHEGDHLGVLPVNSKEQVSRVLRRFNLNGNDQVLLTASGQSAAHLPLDRPVRLHDLLSSCVELQEAASRAQIREMAAYTVCPPHKRELEDFLEEGVYQEQILTSRVSMLDLLEKYEACELPFERFLELLRPLKPRYYSISSSPRKHPGQASITVGVVRGPARSGLGEYRGVASNYLADRGPEDGIVMFVRTPETRFRLPEDPEKPIIMVGPGTGVAPFRGFLQARAALKKEGKELGEAHLYFGCRNDHDFIYRDELEAYEKDGIVTLHTAFSRKEGVPKTYVQHLMAKDAGALISILGRGGHLYVCGDGSKMAPDVEATLQKAYQSVRETDERQAQEWLLDLQTKGIYAKDVWAGI

>CYP102A38_ortholog(U722_03680)Bacillus amyloliquefaciens LFB112

MKETGPIPQPKTFGPLGNLPLLDKDKPTMSLIKLANEQGPIFQLHTPAGAIIVVSGHELVKEVCDEERFDKSIEGALEKVRAFAGDGLFTSWTHEPNWRKAHNILMPTFSQRAMKDYHSMMTDIAVQLIQKWARLNPDEAVDVPADMTRLTLDTIGLCGFNYRFNSYYRETPHPFINSMVRALDEAMHQMQRLDVQDKLMIRTKRQFHHDIQAMFSLVDSIIAERRSGGRDEKDLLARMLNVEDPETGEKLDDENIRFQIITFLIAGHETTSGLLSFAIYFLLKHPRVLEKAYEEADRVLTDPVPSYKQVLELSYIRMILQESLRLWPTAPAFSLYAKEDTVIGGKYPITPKDRISVLIPQLHRDKDAWGDNAEEFYPERFEHPDQVPHHAYKPFGNGQRACIGMQFALHEATLVLGMILQHFTFIDHTDYELDIKQTLTIKPGDFHIRVRPRNKGAVAAALPAAEKAAEDVEKEKRETKGASIIGLDNRPLLILYGSDTGTAEGVARELADTAGMHGVRTETAPLNDRIGKLPKEGALLIITSSYNGKPPSNAGQFVQWLEEVKPGELEGVRYAVFGCGDHNWAATYQAVPRLIDEKLAEKGAERFSARGEGDVSGDFEGKLDEWKKSMWTDAMKAFGLKLNENAEKERSALGLQFVSGLGGSPLAQTYEAVYASVAENRELQAPESGRSTRHIEITLPKEAAYHEGDHLGVLPVNSKEQVSRVLRRFNLNGNDQVLLTASGQSAAHLPLDRPVRLHDLLSSCVELQEAASRAQIREMAAYTVCPPHKRELEDFLEEGVYQEQILTSRVSMLDLLEKYEACELPFERFLELLRPLKPRYYSISSSPRKHPGQASITVGVVRGPARSGLGEYRGVASNYLADRRPEDGIVMFVRTPETRFRLPEDPEKPIIMVGPGTGVAPFRGFLQARAALKKEGKELGEAHLYFGCRNDHDFIYRDELEAYEKDGIVTLHTAFSRKEGVPKTYVQHLMAKDAGALISILGRGGHLYVCGDGSKMAPDVEATLQKAYQSVRETDERQAQEWLLDLQTKGIYAKDVWAGI

>CYP102A38_ortholog(KSO_016080)Bacillus amyloliquefaciens IT-45

MKETGPIPQPKTFGPLGNLPLLDKDKPTMSLIKLANEQGPIFQLHTPAGAIIVVSGHELVKEVCDEERFDKSIEGALEKVRAFAGDGLFTSWTHEPNWRKAHNILMPTFSQRAMKDYHSMMTDIAVQLIQKWARLNPDEAVDVPADMTRLTLDTIGLCGFNYRFNSYYRETPHPFINSMVRALDEAMHQMQRLDVQDKLMIRTKRQFHHDIQAMFSLVDSIIAERRSGGRDEKDLLARMLNVEDPETGEKLDDENIRFQIITFLIAGHETTSGLLSFAIYFLLKHPRVLEKAYEEADRVLTDPVPSYKQVLELSYIRMILQESLRLWPTAPAFSLYAKEDTVIGGKYPITPKDRISVLIPQLHREKDAWGDNAEEFYPERFEHPDQVPHHAYKPFGNGQRACIGMQFALHEATLVLGMILQHFTFIDHTDYELDIKQTLTIKPGDFHIRVRPRNKEAVAAALPAAEKAAEDVEKEKRETKGASIIGLDNRPLLILYGSDTGTAEGVARELADTAGMHGVRTETAPLNDRIGKLPKEGALLIITSSYNGKPPSNAGQFVQWLEEVKPGELEGVRYAVFGCGDHNWAATYQAVPRLIDEKLAEKGAERFSSRGEGDVSGDFEGKLDEWKKSMWTDAMKAFGLKLNENAEKERSALGLQFVSGLGGSPLAQTYEAVYASVAENRELQAPESGRSTRHIEITLPKEAAYHEGDHLGVLPVNSKEQVSRVLRRFHLNGNDQVLLTASGQSAAHLPLDRPVRLHDLLSSCVELQEAASRAQIREMAAYTVCPPHKRELEDFLEEGVYQEQILTSRVSMLDLLEKYEACELPFERFLELLRPLKPRYYSISSSPRKHPGQASITVGVVRGPARSGLGEYRGVSSNYLADRRPEDGIVMFVRTPETRFRLPEDPEKPIIMVGPGTGVAPFRGFLQARAALKKEGKELGEAHLYFGCRNDHDFIYRDELEAYEKDGIVTLHTAFSRKEGVPKTYVQHLMAKDAGALISILGRGGRLYVCGDGSKMAPDVEATLQKAYQSVRETDERQAQEWLLDLQTKGIYAKDVWAGI

>CYP102A38_ortholog(BACAU_0708)Bacillus velezensis CAU B946

MKETGPIPQPKTFGPLGNLPLLDKDKPTMSLIKLANEQGPIFQLHTPAGAIIVVSGHELVKEVCDEERFDKSIEGALEKVRAFAGDGLFTSWTHEPNWRKAHNILMPTFSQRAMKDYHSMMTDIAVQLIQKWARLNPDEAVDVPADMTRLTLDTIGLCGFNYRFNSYYRETPHPFINSMVRALDEAMHQMQRLDVQDKLMIRTKRQFHHDIQAMFSLVDSIIAERRSGGRDEKDLLARMLNVEDPETGEKLDDENIRFQIITFLIAGHETTSGLLSFAIYFLLKHPRVLEKAYEEADRVLTDPVPSYKQVLELSYIRMILQESLRLWPTAPAFSLYAKEDTVIGGKYPITPKDRISVLIPQLHREKDAWGDNAEEFYPERFEHPDQVPHHAYKPFGNGQRACIGMQFALHEATLVLGMILQHFTFIDHTDYELDIKQTLTIKPGDFHIRVRPRNKGAVAAALPAAEKAAEDVEKEKRETKGASIIGLDNRPLLILYGSDTGTAEGVARELADTAGMHGVRTETAPLNDRIGKLPKEGALLIITSSYNGKPPSNAGQFVQWLEEVKPGELEGVRYAVFGCGDHNWAATYQAVPRLIDEKLAEKGAERFSARGEGDVSGDFEGKLDEWKKSMWTDAMKAFGLKLNENAEKERSALGLQFVSGLGGSPLAQTYEAVYASVAENRELQAPESGRSTRHIEITLPKEAAYHEGDHLGVLPVNSKKQVSRVLRRFNLNGNDQVLLTASGQSAAHLPLDRPVRLHDLLSSCVELQEAASRAQIREMAAYTVCPPHKRELEDFLEEGVYQEQILTSRVSMLDLLEKYEACELPFERFLELLRPLKPRYYSISSSPRKHPGQASITVGVVRGPARSGLGEYRGVASNYLADRRPEDGIVMFVRTPETRFRLPEDPEKPIIMVGPGTGVAPFRGFLQARAALKKEGKELGEAHLYFGCRNDHDFIYRDELEAYEKDGIVTLHTAFSRKEGVPKTYVQHLMAKDAGALISILGRGGRLYVCGDGSKMAPDVEATLQKAYQSVRETDERQAQEWLLDLQTKGIYAKDVWAGI

>CYP102A38_ortholog(BAPNAU_0670)Bacillus velezensis NAU-B3

MKETGPIPQPKTFGPLGNLPLLDKDKPTMSLIKLANEQGPIFQLHTPAGAIIVVSGHELVKEVCDEERFDKSIEGALEKVRAFSGDGLFTSWTHEPNWRKAHNILMPTFSQRAMKDYHSMMTDIAVQLIQKWARLNPDEAVDVPADMTRLTLDTIGLCGFNYRFNSYYRETPHPFINSMVRALDEAMHQMQRLDVQDKLMIRTKRQFHHDIQAMFSLVDSIIAERRSGGRDEKDLLARMLNVEDPETGEKLDDKNIRFQIITFLIAGHETTSGLLSFAIYFLLKHPRVLDKAYEEADRVLTDPVPSYKQVLDLTYIRMILQESLRLWPTAPAFSLYAKEDTVIGGKYPITPKDRISVLIPQLHRDKDAWGDNAEEFYPERFEHPDQVPQHAYKPFGNGQRACIGMQFALHEATLVLGMILQHFTFIDHTDYELDIKQTLTIKPGDFHIRVRPRNKGAVAAALPAAEKAAGDVEKEKRETKGASIIGLDNRPLLILYGSDTGTAEGVARELADTAGMHGVRTETAPLNDRIGKLPKEGALLIITSSYNGKPPSNAGQFVQWLEEVKPGELEGVRYAVFGCGDHNWAATYQAVPRLIDEKLAEKGAERFSSRGEGDVSGDFEGKLDEWKKSMWTDAMKAFGLKLNENAEKERSSLGLQFVSGLGGSPLAKTYEAVYASVAENRELQAPESGRSTRHIEITLPKEAAYHEGDHLGVLPVNSKEQVSRVLRRFNLNGNDQVLLTASGQSAAHLPLDRPVRLHDLLSSCVELQEAASRAQIREMAAYTVCPPHKRELEDFLEEGVYQEQILTLRVSMLDLLEKYEACELPFERFLELLRPLKPRYYSISSSPRKHPGQASITVGVVCGPARSGLGEYRGVASNYLADRGPEDGIVMFVRTPETRFRLPEDPEKPIIMVGPGTGVAPFRGFLQARAALKKEGKELGEAHLYFGCRNDHDFIYRDELEAYEKDGIVTLHTAFSRKEGVPKTYVQHLMAKDAGALISILGRGGHLYVCGDGSKMAPDVEATLQKAYQSVHETDERQAQEWLLDLQTKGIYAKDVWAGI

>CYP102A38_ortholog(BAXH7_00690)Bacillus amyloliquefaciens XH7

MKETSPIPKPKTFGPLGNLPLLDKDKPTMSLIKLAKEQGPIFQLHTPAGTIIVVSGHELVKEVCDEERFDKSIEGALEKVRAFSGDGLFTSWTHEPNWRKAHNILMPTFSKRAMKDYHSMMTDIAVQLIQKWARLNPDEAVDVPADMTRLTLDTIGLCGFNYRFNSYYRETPHPFINSMVRALDEAMHQMQRLDVQDKLMIRTKRQFHHDIQAMFSLVDSIIAERRSDGGRDEKDLLARMLNVEDPETGEKLDDENIRFQIITFLIAGHETTSGLLSFAIYFLLKHPRVLEKAYEEADRVLTDPVPSYKQVLELTYIRMILQESLRLWPTAPAFSLYAKEDTVIGGKYPITTKDRISVLIPQLHRDKDAWGDNAEEFYPERFEHPDQVPHHAYKPFGNGQRACIGMQFALHEATLVLGMILQHFTFTDHTDYELDIKQTLTLKPNDFHIRVRPRSKEAVPAAFTAAEKAAEDVTKEKQETKGASIIGLDNRPLLILYGSDTGTAEGVAWELADTAGMHGVRTETAPLNDRIGKLPKEGALLIITSSYNGKPPSNAGQFVQWLEEVKQGELEGVRYAVFGCGDHNWAATYQAVPRLIDEKLAEKGAERFSSRGEGDVSGDFEGKLDEWKKSMWKDAMKAFGLKLNENAEKERSTLGLQFVSGLGGSPLAQTYEAVYASVAENRELQAPESDRSTRHIEIILPKEAAYNEGDHLGVLPVNSKEQVSRVLRRYNLNGNDQVLLTASGQSAAHLPLDRPVRLHDLLSSCVELQEAASRAQIREMAAYTVCPPHKHELEGLLEEGVYQEQILTLRVSMLDLLEKYEACELPFERFLELLRPLKPRYYSISSSPLKHPGQASITVGVVRGPARSGLGEYRGVASNYLADRSPDDGIVMFVRTPETKFRLPEDPEKPIIMVGPGTGVAPFRGFLQARAALKGEGKELGEAHLYFGCRNDYDFIYRDELEAYEKDGIVTLHTAFSRKEGVPKTYVQHLMAEDAETLISILDRGGHLYVCGDGSKMAPDVEETLQKAYQSAHGTDERQAQEWLLDLQTKGIYAKDVWAGI

>CYP102A38_ortholog(LL3_00745)Bacillus amyloliquefaciens LL3

MKETSPIPKPKTFGPLGNLPLLDKDKPTMSLIKLAKEQGPIFQLHTPAGTIIVVSGHELVKEVCDEERFDKSIEGALEKVRAFSGDGLFTSWTHEPNWRKAHNILMPTFSKRAMKDYHSMMTDIAVQLIQKWARLNPDEAVDVPADMTRLTLDTIGLCGFNYRFNSYYRETPHPFINSMVRALDEAMHQMQRLDVQDKLMIRTKRQFHHDIQAMFSLVDSIIAERRSDGGRDEKDLLARMLNVEDPETGEKLDDENIRFQIITFLIAGHETTSGLLSFAIYFLLKHPRVLEKAYEEADRVLTDPVPSYKQVLELTYIRMILQESLRLWPTAPAFSLYAKEDTVIGGKYPITTKDRISVLIPQLHRDKDAWGDNAEEFYPERFEHPDQVPHHAYKPFGNGQRACIGMQFALHEATLVLGMILQHFTFTDHTDYELDIKQTLTLKPNDFHIRVRPRSKEAVPAAFTAAEKAAEDVTKEKQETKGASIIGLDNRPLLILYGSDTGTAEGVAWELADTAGMHGVRTETAPLNDRIGKLPKEGALLIITSSYNGKPPSNAGQFVQWLEEVKQGELEGVRYAVFGCGDHNWAATYQAVPRLIDEKLAEKGAERFSSRGEGDVSGDFEGKLDEWKKSMWKDAMKAFGLKLNENAEKERSTLGLQFVSGLGGSPLAQTYEAVYASVAENRELQAPESDRSTRHIEIILPKEAAYNEGDHLGVLPVNSKEQVSRVLRRYNLNGNDQVLLTASGQSAAHLPLDRPVRLHDLLSSCVELQEAASRAQIREMAAYTVCPPHKHELEGLLEEGVYQEQILTLRVSMLDLLEKYEACELPFERFLELLRPLKPRYYSISSSPLKHPGQASITVGVVRGPARSGLGEYRGVASNYLADRSPDDGIVMFVRTPETKFRLPEDPEKPIIMVGPGTGVAPFRGFLQARAALKGEGKELGEAHLYFGCRNDYDFIYRDELEAYEKDGIVTLHTAFSRKEGVPKTYVQHLMAEDAETLISILDRGGHLYVCGDGSKMAPDVEETLQKAYQSAHGTDERQAQEWLLDLQTKGIYAKDVWAGI

>CYP102A38_ortholog(BAMTA208_03280)Bacillus amyloliquefaciens TA208

MKETSPIPKPKTFGPLGNLPLLDKDKPTMSLIKLAKEQGPIFQLHTPAGTIIVVSGHELVKEVCDEERFDKSIEGALEKVRAFSGDGLFTSWTHEPNWRKAHNILMPTFSKRAMKDYHSMMTDIAVQLIQKWARLNPDEAVDVPADMTRLTLDTIGLCGFNYRFNSYYRETPHPFINSMVRALDEAMHQMQRLDVQDKLMIRTKRQFHHDIQAMFSLVDSIIAERRSDGGRDEKDLLARMLNVEDPETGEKLDDENIRFQIITFLIAGHETTSGLLSFAIYFLLKHPRVLEKAYEEADRVLTDPVPSYKQVLELTYIRMILQESLRLWPTAPAFSLYAKEDTVIGGKYPITTKDRISVLIPQLHRDKDAWGDNAEEFYPERFEHPDQVPHHAYKPFGNGQRACIGMQFALHEATLVLGMILQHFTFTDHTDYELDIKQTLTLKPNDFHIRVRPRSKEAVPAAFTAAEKAAEDVTKEKQETKGASIIGLDNRPLLILYGSDTGTAEGVAWELADTAGMHGVRTETAPLNDRIGKLPKEGALLIITSSYNGKPPSNAGQFVQWLEEVKQGELEGVRYAVFGCGDHNWAATYQAVPRLIDEKLAEKGAERFSSRGEGDVSGDFEGKLDEWKKSMWKDAMKAFGLKLNENAEKERSTLGLQFVSGLGGSPLAQTYEAVYASVAENRELQAPESDRSTRHIEIILPKEAAYNEGDHLGVLPVNSKEQVSRVLRRYNLNGNDQVLLTASGQSAAHLPLDRPVRLHDLLSSCVELQEAASRAQIREMAAYTVCPPHKHELEGLLEEGVYQEQILTLRVSMLDLLEKYEACELPFERFLELLRPLKPRYYSISSSPLKHPGQASITVGVVRGPARSGLGEYRGVASNYLADRSPDDGIVMFVRTPETKFRLPEDPEKPIIMVGPGTGVAPFRGFLQARAALKGEGKELGEAHLYFGCRNDYDFIYRDELEAYEKDGIVTLHTAFSRKEGVPKTYVQHLMAEDAETLISILDRGGHLYVCGDGSKMAPDVEETLQKAYQSAHGTDERQAQEWLLDLQTKGIYAKDVWAGI

>CYP102A38_ortholog(BAMF_0695)Bacillus amyloliquefaciens DSM 7

MKETSPIPKPKTFGPLGNLPLLDKDKPTMSLIKLAKEQGPIFQLHTPAGTIIVVSGHELVKEVCDEERFDKSIEGALEKVRAFSGDGLFTSWTHEPNWRKAHNILMPTFSKRAMKDYHSMMTDIAVQLIQKWARLNPDEAVDVPADMTRLTLDTIGLCGFNYRFNSYYRETPHPFINSMVRALDEAMHQMQRLDVQDKLMIRTKRQFHHDIQAMFSLVDSIIAERRSDGGRDEKDLLARMLNVEDPETGEKLDDENIRFQIITFLIAGHETTSGLLSFAIYFLLKHPRVLEKAYEEADRVLTDPVPSYKQVLELTYIRMILQESLRLWPTAPAFSLYAKEDTVIGGKYPITTKDRISVLIPQLHRDKDAWGDNAEEFYPERFEHPDQVPHHAYKPFGNGQRACIGMQFALHEATLVLGMILQHFTFTDHTDYELDIKQTLTLKPNDFHIRVRPRSKEAVPAAFTAAEKAAEDVTKEKQETKGASIIGLDNRPLLILYGSDTGTAEGVAWELADTAGMHGVRTETAPLNDRIGKLPKEGALLIITSSYNGKPPSNAGQFVQWLEEVKQGELEGVRYAVFGCGDHNWAATYQAVPRLIDEKLAEKGAERFSSRGEGDVSGDFEGKLDEWKKSMWKDAMKAFGLKLNENAEKERSTLGLQFVSGLGGSPLAQTYEAVYASVAENRELQAPESDRSTRHIEIILPKEAAYNEGDHLGVLPVNSKEQVSRVLRRYNLNGNDQVLLTASGQSAAHLPLDRPVRLHDLLSSCVELQEAASRAQIREMAAYTVCPPHKHELEGLLEEGVYQEQILTLRVSMLDLLEKYEACELPFERFLELLRPLKPRYYSISSSPLKHPGQASITVGVVRGPARSGLGEYRGVASNYLADRSPDDGIVMFVRTPETKFRLPEDPEKPIIMVGPGTGVAPFRGFLQARAALKGEGKELGEAHLYFGCRNDYDFIYRDELEAYEKDGIVTLHTAFSRKEGVPKTYVQHLMAEDAETLISILDRGGHLYVCGDGSKMAPDVEETLQKAYQSAHGTDERQAQEWLLDLQTKGIYAKDVWAGI

>CYP102A39(HM131_13145)Halobacillus mangrovi

MNIHSGFINLLLRGDFKINTQTLPQPKSYGPLGNLPLIDKEKPIQSFMKLARELGPLYQFHFPGRTSNFVSSARFAAEICDETRFDKKLGPALQKVRSFGGDGLFTSGTEEPNWKKAHNILLPSFSQQAMKGYHDKMVDLATQLIQKWARLNPKEEVDVPEDMTRLTLDTIGLCGFNYRFNSFYRETSHPFVQKMVRALDESMNQTQRLGIQDKLMVKSKRQYKEDIDYMFDLVDELIKERKETGDQGEDDLLAHMLKGKDPETGEALGDENIRFQIITFLIAGHETTSGLLSFALYYLLKNPEKLEKAYKEVDEVLGEETPTYKQVKKLKYVRMILNEALRLWPTAPAFSVYAKDNTSLDGVDEVEEGEAFTLLIPELHRDKSVWGEDVETFKPERFEDPSSIPHHAYKPFGNGQRACIGQQFALHEATLVLGMVLQHFELEDHSNYELDVKETLTLKPDGLTMRVKSRKNADFIQMPVHSEKKAVEDNNPKTTMVDAHNTPMLVLYGSNLGTSEGVSRELAEKGRHQGFQVETAPLDDYTGQLPVDGATIIVSASYNGHPPDNAVSFVEWVASTPQEDLKGVQYAVFGCGDRNWANTYQRIPKLIDEGLESRGATRIVTRGEGDANEDFEGDLEKWEEILWDRLAKTFNLKLEESNQQSNHISMEFISGSTHTPVAREHHAFTAVVTKNRELLSMEERSTRHIELQLPAGVMYKEGDHLGVLPQNSDKLVTRVLERFGLNGEDYVVLGEDAGKAAHLPTQQPIELKELLMSYVELQEPATRRQLRELAASNPCPPHKKELEQWLEDNKYKQEVLEKRKTMLEILEEYASCELDFERFLALLPALKPRYYSISSSPRVQQDKVSVTVSVVRGPAWSGKGEFLGIASNYLACRQVASKVACFIRTPQSNFQLPEDSAAPLVLVGPGTGIAPFRGFIQTRRVLKEQGQTLGDAHLYFGCRHPEQDFLYEKELKKAEREGLITLHTAFSRHESCKKTYVQHLMEQDSSRLVDLLNNGGFLYICGDGSKMAPQVTETLIKGYQDIQQTSYEDAVEWLDNLEKEGRFAKDVWAGV

>CYP102A40(HBHAL_1310)Halobacillus halophilus

MEKTNHLPQPKSYGPLGNLPLINKEKPIQSFMKLADEFGPLYQFQFPGRTSRFVSSAQFAAEICDESRFDKKVGPALQKVRAFGGDGLFTSETEEINWKKAHNILLPSFSQQAMKGYHNKMVDLASQLIQKWARLNPNEEVEVPEDMTRLTLDTIGLCGFNYRFNSFYRERSHPFVEKMVHALDESMSQTQRFELQDKLMIRTKKQYKEDIEYMFNLVDQLIAERKETGDQGEDDLLAHMLKGKDPETGEALDDENIRFQIITFLIAGHETTSGLLSFAIHYLMKNPDKLKKAQEEVDEVIGEDIPSYKQVKKLKYVRMILNEALRLWPTAPAFSVYAKENTTLAGQYEVEKGETFTLLLPQLHRDTSIWGEDAEAFKPERFEDPSQIPRHAYKPFGNGQRACIGQQFALHEATLVLGMVLQYFDFEDHTNYQLDVKEALTMKPEGLTMRVKSRRDAALMQPAPAQKEAEKSSKTEAQSVPDAHLTPLLVLYGSNMGTAEGVARELAQTGKLQGFDVKTAPLNQYTSALPAEGAVLIVSASYNGNPPDNADAFVRWLKEADGEEAKGVSYAVFGCGDRNWASTYQRVPTLIDEELQALGAERILAREGGDASEDFEGDLEKWEAALWPALADTFNLELEEADDPSSHVSMEFVSGVTQTPLARTHHAFTAMVNENRELQVSSERSTRHLELKLPEGVNYQEGDHLGVLPQNGTELVERVLRRFQLKGEEYVVLGEDTGKATHLPTSQPISLRELLTSYVELQEPATRSQIRDLAAHNPCPPHKMELEKLLEDETYKNEILAHRLTMLDLLEDYLSCEVPFERFLALLPPLKARYYSISSSPRKHSEEASVTVSVVKDTAWSGRGEFKGTASNYMANRDIGDKVACFINTPQSNFTLPENPETPMVWIGPGTGIAPFRGFIQARENLLEEGHRLGEAHLYFGCRHPEKDFLYKNELEKAAD

>CYP102A41(B2K_06870)Paenibacillus mucilaginosus K02

MTQTSQIPQPKTFGPLGNLPNLNTKEPVQSIVKIADEFGPIFQLDLPRGKSVYISSHELVADACDESRFDKYVWAPLQKVRAFAGDGLFTAETDEPNWQKAHAILLPSFSQRAMQGYHAMMVDLAVQLVQKWARLNPDESVEVGEDMTRLTLDTIGLCGFNYRFNSFYRDQPHPFITSMVRALDEAMSQLQRLGLQDKLMVISKRQYKHDIQTMFGLVDKIIAERKEHGSKEGEDLLAHMLNGRDPHTGEALDDENIRYQIITFLIAGHETTSGLLTFALYFLLKNPEKLAKAYEEVDRVLTSPLPTYSQVRELKYIRMILNESLRLWPTAPAFALTAKEDTMLGGRYPMQKGDSVSVLIPKLHRDPSVWGDDVESFIPERFEDPSSIPPHAYKPFGNGQRACIGQQFALHEAVLVLGMVLRHFEIIDHTNYQLKIKETLTLKPEGFTMRVRSRIEPLAVVMPGREAAPLEEKREQAAEAAPVPKHNTPLLVLYGSNLGTAEGIARELADAARYQGFSSETAPLDEHVGSLPKEGAVFIVTASYNGSPPDNARAFVQWLEQAEPGSLDGVRYAVFGCGDHNWASTYQQVPRKIDALLADKGAKRLAGLGEGDVSDDFEQQLESWRGGLWPQVMEAFGLSLNENAGQERSSLTVQFVSGLIGTPLAESYDALQTTVAENRELQGEGSDRSTRHIEVVLPEEVSYREGDHLGVLPLNPKDLVGRVLQRFGLQGGDHLVLSATGRSAAHLPLGRPVRLSDLLGSSVELQEPATRAQLRELAAYTVCPPHKRELEALLQEDAYKTEVLRKRVTMLELLEKYPACELPFERFLELLPPLKARYYSISSSPRVLGDRVSITVSVVRGPAWSGRGEYRGIASNYLAERKPGDPVVVFVRSPESGFELPEDPSTPVIMVGPGTGVAPFRGFLQARRVLKAQGAELGEAHLYFGCRNPQHDYLYREELEQAERDGLVVLHTACSRVDGQEKTYVQDLMKGDASLLIGLLDRGGKLYICGDGSRMAPDVEATLRRAYSEIHGVSGQEAADWLDGLARENRYAKDVWAGTAG

>CYP102A41_ortholog(KNP414_03453)Paenibacillus mucilaginosus KNP414

MTQTSQIPQPKTFGPLGNLPNLNTKEPVQSIVKIADEFGPIFQLDLPRGKSVYISSHELVADACDESRFDKYVWAPLQKVRAFAGDGLFTAETDEPNWQKAHAILLPSFSQRAMQGYHAMMVDLAVQLVQKWARLNPDESVEVGEDMTRLTLDTIGLCGFNYRFNSFYRDQPHPFITSMVRALDEAMSQLQRLGLQDKLMVISKRQYKHDIQTMFGLVDKIIAERKEHGPKDGEDLLAHMLNGRDPHTGEALDDENIRYQIITFLIAGHETTSGLLTFALYFLLKNPEKLAKAYEEVDRVLTSPLPTYSQVRELKYIRMILNESLRLWPTAPAFALTAKEDTMLGGRYPMKKGDSVSVLIPKLHRDPSVWGDDVESFIPERFEDPSSIPPHAYKPFGNGQRACIGQQFALHEAVLVLGMVLRHFEIIDHTNYQLKIKETLTLKPEGFTMRVRSRIEPQAVVMPGREAAPLEEKREQAAEAAPVPKHNTPLLVLYGSNLGTAEGIARELADAARYQGFSSETAPLDEHVGSLPKEGAVFIVTASYNGSPPDNARAFVQWLEQAEPGSLDGVRYAVFGCGDHNWASTYQQVPRKIDALLADKGAKRLAGLGEGDVSDDFEQQLESWRGGLWPQVMEAFGLSLNENAGQERSSLTVQFVSGLIGTPLAESYDALQTTVAENRELQGEGSDRSTRHIEVVLPEEVSYREGDHLGVLPLNPKDLVGRVLQRFGLQGGDHLVLSATGRSAAHLPLDRPVRLSDLLGSSVELQEPATRAQLRELAAYTVCPPHKRELEALLQEDAYKTEVLRKRVTMLELLEKYPACELPFERFLELLPPLKARYYSISSSPRVLGDRVSITVSVVRGPAWSGRGEYRGIASNYLAERKPGDPVVVFVRSPESGFELPEDPSTPVIMVGPGTGVAPFRGFLQARRVLQAQGAELGEAHLYFGCRNPQHDYLYREELEQAEKDGLVVLHTACSRVDGQEKTYVQDLMKGDASLLIGLLDRGGKLYICGDGSRMAPDVEATLRRAYSEIHGVSGQEAADWLDGLARENRYAKDVWAGTAG

>CYP102A42(UP17_20335)Bacillus simplex

MENTTQFPQPKSYGPLGSLPIIDKDKPLQSYMKLARELGPVFQFQFPGRISTFVSSASLAKEICDETRFDKKVGPSLQKVRAFGGDGLFTSETAEPNWKKAHNILLPSFSQQAMKGYHAKMVDLATQLIQKWTRVNPADVIDVPEDMTRLTLDTIGLCGFNYRFNSFYRETTHPFVTSMVRALDEAMSQTQRLGIQDKLMVKSKKQFREDIQYMFSLVDEMIAERKQNGDQGEDDLLSHMLKGVDPETGVSLDDENIRFQIITFLIAGHETTSGLLSFAIYFLMNNRDKLKKAQQEVDEVLGDDVPDYKQVKKLKYVRMVLNEVLRLWPTAPAFSVYAKEDTILDEKYTVKKGDVFTLLIPELHRDPSVWGDDVESFIPERFENLDSIPYHAYKPFGNGQRACIGQQFALHEATLVLGMVLQHFDLIDHEDYQLDVKETLTLKPDGLTMRVSPRKPAMSFTVASPEPKSADKGATASSIESAHGTPLLVLFGSNMGTAEGIARDLAETGKLQGFNARVAPLNDYTNRLPQEGAVLIVSASYNGNPTDNADDFVSWLKESHDATLDGVHYAVFGCGDRNWANTYQRIPIFIDERLEQKGAVRLSETGYGDASDDFEGDYEKWTEALWPNLAETLNIEVNMNERLVSSITMNFVSNVSGTPLARTHHAFTSIVKRNLELQHVESGRSTRHIELTVPEGIYYKEGDHLGVLPQNPSELVERVLSRFSLNGQDYVNLTGDSGKAAHLPTGKPIKLEELLSNHVEFQEPATRSQIRALATHTVCPPHVKELENLLEDSTYKREILNKRMTMLDLTETYLACEIPFERFLALLPPLKARYYSISSSPLHKEGEASITVSVVRGKALSGNGEYKGIASNYLAERSEGDKVACFINTPQSNFQLPEQTEKPIIMIGPGTGIAPFRGFIQARRALKKKGETLGSAHLYFGCRNPEHDFLYQEELVQAEHEGLVTLHTAFSRCPGQEKTYVQNRLAENAQDILPLLKEGGHLYICGDGSKMAPDVERTLIDSYMHFYQTTKEEATEWLQSLEENGRYAKDVWAGA

>CYP102A43(DJ92_5480) Bacillus pseudomycoides 219298

MREEQTSNIPQPKTYGPLGNYPLFDMSQPTISFCEMAKEYGPIFRLTAPGFKTICISEHKLVTEVCDINRFDKHVANDMVHVRKFTGDGLFTSKTSEPNWRKAHNILLPTFSQQAMKGYHTMMLDIASQLIQKWARLNHDSDHIDVPGDMTRLTLDTIGLCGFNYRFNSFYKEKHDPFIDSMVRALDEAMHKIARSKGLDTLMIRKNRQFQDDIQSMFSLVDKIIKERKANGNNGEIDLLARMLNSKDPETGEVLDDENIRYQIITFLIAGHETTSGLLSFTTYFLLKHPEVLKKAYEEVDQVLGDSTPSYKQVLNLKYIRMILNEAIRLWPTAPSFDVYPKEDTIIGGQYHVKKGEGLTVLLPALHRDKEVWGEDAEEFRPERFADPSKVPNHAYKPFGNGQRACIGMQFALYEATLVLGMILQKLELIDSENYQLKIKQSLTIKPENFRIRVKLREGKEVAPLLNLPLDDEKENEEKKKVTSQYSKQEVLEGAENVPLLVLYGSNLGRAEEFARKLADHARLLGFQSDVRTLNDYIGKLPKEGAVLIVTSSYNGQPPENAAQFVKWLEQASAEECTGIRYAVFGCGDRNWANTYQSVPKFIDKELELKGAIRYTERGEADAGGDFENDFAQWQKNMWKDVSNVFCLKMKEGTETVSHQSSLSVQLISGPHANPIVQKNEAVYATVIASRELQSSESERSTRHIEIALPEGVTYQVGDHLGVLPSNSRKNVNRILKRFRLNEKDQVILSTSGSSTAHLPLDRPVSLFDLLSHSVEIQEAATRAQIRELARFTVCPPHKYELEALLEDGVYQDQILKKHISMLDLLEKYEACEIPFERFLELLPALKPRYYSISSSPLVAQDRLSITVGVVSGPAWSGQGEYKGVASNYLAQRHNEDEITCFIRTPESRFQLPEDPEKPIIMVGPGTGVAPFRGFLQTRRVQKQKGVNLGEAHLYFGCRHPEKDYLYRTELENDEKDGLISLHTAFSRSEGHPKTYVQHLMKQDGANLISLLDNGAHLYICGDGSRMAPDVEDTLCHAYQEMHGVSEQEARNWLDQLQHEGRYGKDVWAGI

>CYP102A44(VN24_23120)Paenibacillus beijingensis

MDNNKNPQIPQPKTFGPLGNLPQLNIEEPVQSMVRLADEFGPIFRMDYPGGRSEIYISDQDLVADACDESRFDKKVWAPLQKVSAFTGDGLFTSATEEPNWQKAHNILLPSFSQRAMQGYHNMMVDIAVQLVQKWARLNPDESVNVPEDMTRLTLDTIGLCGFDYRFNSFYRDQPHPFIVSMVRALDEAMSQLTRLGIQDKLMPMTKRQFKRDIEAINSLVDNIIAERRLSGNQDAEDLLSRMLNNKDPETGEGIDDENIRYQIITFLIAGHETTSGLLSFAIYYLLNNLDKLHKAYEEVDRVLTDSTPTYKQVLDLKYIRMILSETLRLWPTAPAFSLFAKEDTILAGRYPLKKGESVNVLIPKLHRDTNAWGDDVEDFRPERFEDQSQVPQDAYKPFGNGQRACIGQQFALHEAVLVLGMVLKHFELIDHTHYQLKVKETLTLKPDNFTIHVRSRQPMELNMSVADKSSSTGKDDNRTNQEPQSVSIIGASHLSLLVLYGSNLGTAEGIARELADTARFYGVRSEVDTLNNRIGKLPKEGAAVFIVTASYNGKPPSNASEFVQWLERVEPGELEGVNYVVFGCGDRNWASTYQDVPKFIDKQLADKGARRLSLRGEGDASGDFEKELEDWRDRLWPDVMEAFELKFNEKMDKERSSLTVQFVSGLAGAPLAQSYEAVHATVTENNELQLVNTGRSTRHIEITLPEGVTYQEGDHLGVLPSNSREIVDRVLRRFGLNGNDQLVVKASGRSSAHLPLDSPVSLYDLLSHSVELQEAATRAQLRELMVVTVCPPHKHEIEALLEEEAYKEQVLKKRISMLDILEKYEACEMSFERFLELLPPLKARYYSISSSPRLTPERSSITVAVVQGPAWSGHGEYRGVTSNYLADRKPGDDIVMFVRTPESGFQLPEDTETPIIMVGPGTGVAPFRGFLQARAALKQEGKTLGAAHLYFGCRTEDDFIYREELEQYERDGVVTLHTAFSRKGQDEKTYVQHLMNKNKEELIDILDHNGRLYICGDGSKMAPDVEKTLQEAYQEVHTVDEQEAKAWLDGLQNEGRYAKDVWAGI

>CYP102A44_ortholog(UB51_10470)Paenibacillus sp. IHBB 10380

MDNNKNPQIPQPKTFGPLGNLPQLNIEEPVQSMVRLADEFGPIFRMDYPGGRSEIYISDQDLVADACDESRFDKKVWAPLQKVSAFTGDGLFTSATEEPNWQKAHNILLPSFSQRAMQGYHNMMVDIAVQLVQKWARLNPDESVNVPEDMTRLTLDTIGLCGFDYRFNSFYRDQPHPFIVSMVRALDEAMSQLTRLGIQDKLMPMTKRQFKRDIEAINSLVDNIIAERRLSGNQDAEDLLSRMLNNKDPETGEGIDDENIRYQIITFLIAGHETTSGLLSFAIYYLLNNLDKLHKAYEEVDRVLTDSTPTYKQVLDLKYIRMILSETLRLWPTAPAFSLFAKEDTILAGRYPLKKGESVNVLIPKLHRDTNAWGDDVEDFRPERFEDQSQVPQDAYKPFGNGQRACIGQQFALHEAVLVLGMVLKHFELIDHTHYQLKVKETLTLKPDNFTIHVRSRQPMELNMSVADKSSSTGKDDNRTNQEPQSVSIIGASHLSLLVLYGSNLGTAEGIARELADTARFYGVRSEVDTLNNRIGKLPKEGAAVFIVTASYNGKPPSNASEFVQWLERVEPGELEGVNYVVFGCGDRNWASTYQDVPKFIDKQLADKGARRLSLRGEGDASGDFEKELEDWRDRLWPDVMEAFELKFNEKMDKERSSLTVQFVSGLAGAPLAQSYEAVHATVTENNELQLVNTGRSTRHIEITLPEGVTYQEGDHLGVLPSNSREIVDRVLRRFGLNGNDQLVVKASGRSSAHLPLDSPVSLYDLLSHSVELQEAATRAQLRELMVVTVCPPHKHEIEALLEEEAYKEQVLKKRISMLDILEKYEACEMSFERFLELLPPLKARYYSISSSPRLTPERSSITVAVVQGPAWSGHGEYRGVTSNYLADRKPGDDIVMFVRTPESGFQLPEDTETPIIMVGPGTGVAPFRGFLQARAALKQEGKTLGAAHLYFGCRTEDDFIYREELEQYERDGVVTLHTAFSRKGQDEKTYVQHLMNKNKEELIDILDHNGRLYICGDGSKMAPDVEKTLQEAYQEVHTVDEQEAKAWLDGLQNEGRYAKDVWAGI

>CYP102A45(BRLA_c016970)Brevibacillus laterosporus

MITISHLQNIPQPKTFGPLGNLPLINPEMPIQSIMKLAHELGPIFRLEYPGGRVSIYISNHKLVAEACDESRFDKNVWPPLQKVRTFAGDGLFTSSTDEPNWQKAHNILLPSFSQRAMQGYHSMMVDIAVQLIQKWARLNPDESIDVPEDMTRLTLDTIGLCGFNYRFNSFYREQPHPFITSMVRALDEAMSQLQRLEIKDKLMVITKRQFKHDIQAMFTLVDKIIAERKEHEDHDANDLLSHMLKGKDPQTGEGLDDENIRYQIITFLIAGHETTSGLLSFALYFLLKNPDKLQKAYEEVDRVLTGSTPTYTEVRNLTYISMILNESLRLWPTAPAFSLYAKEDTLLDGKYPLKKGESVNVLIPTLHRDTSVWGDDVEEFRPERFEDPSQIPYDAYKPFGNGQRACIGQQFALQEATLVLGMILKYFDIIDHNHYQLKIKETLTLKPEGFSMRVRLRTNELSFQITGKEKEREKTQAQPTASTIVDAHNTPLLVLYGSNLGTAEGIARELAEDARFQGFKSEVAPLNDYLGKLPKEGAVFIITSSYNGKPPSNAKDFVQWLKEAEPEEFTGVNYTVFGCGDHNWASTYQQVPHLIDKQLEAKGATRLMKQGEGDASGDFEKQLEDWRHQLWPGVMNALGLKINENRERERSTLSVQYVNGIAGVPLAESYDALRSYVTENRELQGETSERSTRHIEIAIPAGVHYHEGDHLGVLPKNCPQLVERIVHRFGLNGNDYLILSGSGRSAAHLPLDRPVSVYDLLSHSVELQEAATRAQLRELAAYTTCPPHKRELEALLEEDTYKTNIMNIRISMFNLMEQYPACELPFERFLELLPPLKARYYSISSSPHLLPDRTSITVSVVRGPAWSGHGEYRGVASNYLADLKPNDPVVIFVRTPESHFTLPTNTETPLIMIGPGTGVAPFRGFLQARKHYQQQGKQLGEAHLYFGARHPQQDYLYQEEFAQYEKEGIVTIHTAFSRVEGQPKTYVQHLMKQNEQELIRLLDQGARLYVCGDGSRMAPDVEATLISSYQTIHSVSKEKAQAWLDKLQRDEQYVKDVWTGA

>CYP102A46(VN24_14405)Paenibacillus beijingensis

MSETITVPLPKMFGPLGNLPLLDKETPMQSIAKIADELGPIFRLELPGRSDLWIWGHELVKDACDESRFDKYLPVPLQKLRPIGGDGLFTSWTDEPNWKKAHNILLPSFSQRAMQGYHAMMADIALQLIQKWSRLNPDESIDVPEDMTRLTLDTIGLCGFGYRFNSYYRDNPHPFIVNMVRALTEGMEQSQRLSLQDKLMVMTKRQFKLDIQSMFSLVDKIIAERKESGSQDAQDLLSRMLRSKDPETGETLSEENIRYQIITFLIAGHETTSGLLSFSLYFLLKNPDKLRKAYEEVDRVLTEPVPTYNQVRSLKYIRMILDESLRLWPTAPIFSLYAKEDTMLGGVYSMKKGEGVNVLLPKLHRDTAVWGDDAEAFRPERFENPSLIPQHAYKPFGNGMRACIGMQFSLHEATMVLGMILRQFEIIDHTNYQLKVKETLTLKPQGLTMRVRPRAGQPIAAVALKEEAPDKKMTQPVAASSAVQSHNTPLLVLYGSNLGTAEGIARELADAARYQGFRTEVAPLDERIGKLPTEGAVLIVAASYNGKPADNARGFVQWLEQAEPDAVKGVRYAVFGCGDRNWAVSYQQVPRLIDRQLDAKGASRLTGIGEGDASDDFEKQLDDWRNRMWPDMMGAFDLTVNENAKQEPSTLTVRLVSGPVDTPLAESYDAFFASVLDNRELQSAESGRSTRHLEFALPEGAAYQEGDHLGVFPHNHPDLVERVLRRYELNGSDYFILSASTSSAAHLPLDRPVNVRVLLSRSVELQEAATRAQLRELASYTVCPPHKRELEALLQEDAYKTNVLEKRLTMLDLLEQYPACEMPFERFLELLPPLKARYYSISSSSRVQPDRASITVGVVSGPARSGRGEYRGVASNYLAGRLTGDSVALFVRRPESGFQLPDDPMTPIIMVGPGTGIAPFRGFLQARRALVQEGREVGEAHLYFGCRNPHHDYLYQEELASFEREGLVTLHTAFSRVEGKEKRYVQHLIRQDAGHLIRLLDQGGRLYICGDGSRMAPDVEAALRSAYGSVHGGSERDAQLWLDQIRDEGRYAKDVWAGVMENKKYANMLIMPELDSAEKRLFPFDILSVLRKEAPVRYDRTRNCWDVFAYGDVHRVLKDSRTFSSVRGAAAGQNLLFMDPPKHTQLRDLVNKAFTPKAIQELAPRIQSIAEQLLDQVTGEEMDIVSDFATPLPVIVISELLGAPKEDRAHFKRWSDILVESAEDVSDEAFQKITEKRMGAMQELTLYFKSILELRAKQPQDDLISALLKAEINGEKLTERDLIGFCILLLAAGNETTTNLITNGVRIVTEQPSIHSELNLSPETIPSFIEEVLRYYPPIVAIGRIATENTEIGSQTVQAGDQIIIWVGAANWDESKFTDPDKFDLHRKPNPHLSFGFGIHFCLGAPLARLEGKIALQALLDRYNEIKLQTDQPLIPIPSSFVFGVKNYPITFVQ

>CYP102A47(BATR1942_11285)Bacillus atrophaeus 1942

MKQASLIPQPKTYGPLKNLPQLDKEKVSQSLWRIADEYGPIFRFEFPGAVGVFVSGHELVAEVCDESRFDKNLSNSLQKVREFSGDGLFTSWTHEHNWQKAHRILLPSFSQKAMKGYHSMMLDIAEQLIQKWSRLNPNEEIDVAEDMTRLTLDTIGLCGFNYRFNSFYRDTQHPFITSMLRALKEAMRQTQRLSLQDKLMVKAKQQFQHDIEVMNALVDRIIAERKENPDENVKDLLSLMLHAEDPVTGERLDDENIRHQIITFLIAGHETTSGLLSFAIYCLLKNRDKLEKACQEAEQVLTGDTPTYKQIQHLKYIRMVLNEALRLYPTAPAFSVYAKEDTVLGGQYQISKGQPVSVLVPKLHRDQSVWGEDAEDFRPERFENPSDIPNHAYKPFGNGQRACIGMQFALQEATMVLGLVLKHFELIDHTDYELKIKEALTIKPDHFKIRVKPKKSASKSFSKQTAEQPEQTNKRAETAGKPSHGTPLLVLYGSNLGTAEGLAEELADIGRYQGFQTETAPLDDYIGNLPAKGAVVIVTASYNGAPPDNAAGFVKWIETLEAGELKGVNYAVFGCGNRNWAGTYQRIPRLIDETLAAKGANRLIPIGEGDAADDFESSQEEWEQRFWEDTLKAFHLQASSAKEERPALSIEFMSETIGTPLAKTYDAFEAIVEENRKLQTNTSPRDTRHIELQVPVAEDYKEGDHIGILPKNSKELVGRVIKRFGLAPHSLVKISGGRNVSHLPLEQPINVADLLSSNVELQEPATRAQLRELAAYTVCPPHKKELEMLLSDQTYKDQVLKKRITMIDLLEDYPACEMPFERFLELLPSLKARYYSISSSPRVYQHKVSITVGVVASPAWSGSGEYRGVASNYLAGLKAGDRVVCFIRTPQSGFRLPESFETPLIMVGPGTGIAPYRGFIQARGVWKEKGNKLGEAHLYFGCRHPEQDDLYREELDQAEDAGLVNVHRGYSRRETEPKVYVQHLLKQDAEQVIALLDQGAYFYVCGDGSRMAPEVEETLREAFEAVKGESRKASAEWISKLQEEGRYVKDVWTGV

>CYP102A47_ortholog(TD68_10360)Bacillus atrophaeus NRS 1221A

MKQASLIPQPKTYGPLKNLPQLDKEKVSQSLWRIADEYGPIFRFEFPGAVGVFVSGHELVAEVCDESRFDKNLSNSLQKVREFSGDGLFTSWTHEHNWQKAHRILLPSFSQKAMKGYHSMMLDIAEQLIQKWSRLNPNEEIDVAEDMTRLTLDTIGLCGFNYRFNSFYRDTQHPFITSMLRALKEAMRQTQRLSLQDKLMVKAKQQFQHDIEVMNALVDRIIAERKENPDENVKDLLSLMLHAEDPVTGERLDDENIRHQIITFLIAGHETTSGLLSFAIYCLLKNRDKLEKACQEAEQVLTGDTPTYKQIQHLKYIRMVLNEALRLYPTAPAFSVYAKEDTVLGGQYQISKGQPVSVLVPKLHRDQSVWGEDAEDFRPERFENPSDIPNHAYKPFGNGQRACIGMQFALQEATMVLGLVLKHFELIDHTDYELKIKEALTIKPDHFKIRVKPKKSASKSFSKQTAEQPEQTNKRAETAGKPSHGTPLLVLYGSNLGTAEGLAEELADIGRYQGFQTETAPLDDYIGNLPAKGAVVIVTASYNGAPPDNAAGFVKWIETLEAGELKGVNYAVFGCGNRNWAGTYQRIPRLIDETLAAKGANRLIPIGEGDAADDFESSQEEWEQRFWEDTLKAFHLQASSAKEERPALSIEFMSETIGTPLAKTYDAFEAIVEENRKLQTNTSPRDTRHIELQVPVAEDYKEGDHIGILPKNSKELVGRVIKRFGLAPHSLVKISGGRNVSHLPLEQPINVADLLSSNVELQEPATRAQLRELAAYTVCPPHKKELEMLLSDQTYKDQVLKKRITMIDLLEDYPACEMPFERFLELLPSLKARYYSISSSPRVYQHKVSITVGVVASPAWSGSGEYRGVASNYLAGLKAGDRVVCFIRTPQSGFRLPESFETPLIMVGPGTGIAPYRGFIQARGVWKEKGNKLGEAHLYFGCRHPEQDDLYREELDQAEDAGLVNVHRGYSRRETEPKVYVQHLLKQDAEQVIALLDQGAYFYVCGDGSRMAPEVEETLREAFEAVKGESRKASAEWISKLQEEGRYVKDVWTGV

>CYP102A48(MY9_2696)Bacillus sp. JS

MKQASAIPQPKTYGPLKNLPHLEKEQLSQSLWRLADELGPIFRFDFPGVSSVFVSGHNLVTEVCDESRFDKNLGKGLLKVREFGGDGLFTSWTHEPNWQKAHRILLPSFSQKAMKGYHSMMLDIATQLIQKWSRLNPNEEIDVADDMTRLTLDTIGLCGFNYRFNSFYRDSQHPFITSMLRALKEAMNQSKRLGLQDKMMVKTKLQFQKDIEVMNSLVDRMIAERKANPDENIKDLLSLMLYAKDPVTGETLDDENIRYQIITFLIAGHETTSGLLSFAIYCLLTHPEKLKKAQEEADRVLTDDTPEYKQIQQLKYIRMVLNETLRLYPTAPAFSLYAKEDTVLGGEYPISKGQPVTILIPKLHRDQNAWGADAEDFRPERFEDPSSIPHHAYKPFGNGQRACIGMQFALQEATMVLGLVLKHFDMLNHTGYKLKIKEALTIKPDDFKITVKPRKTAAINVQRREQADIKTETKPKETQPKHGTPLLVLYGSNLGTAEGIAGELAAQGRQMGFTAETAPLDDYIGKLPEEGAVVIVTASYNGAPPDNAAGFVEWLKELEEGRLKGVSYAVFGCGNRSWASTYQRIPRLIDDMMKAKGATRLTAIGEGDAADDFESHRESWENRFWKETLDAFDINEIAQKEDRPSLSITFLSEATETPVAKAYGAFEGIVLENRELQSADSTRSTRHIELEIPEGKTYKEGDHIGILPKNSRELVQRVLSRFGLQSNHVIQISGSAHMAHLPMDRPIKVADLLSSYVELQEPASRLQLRELASYTVCPPHQKELEQLVSDDGTYKEQVLAKRLTMLDLLEDYPACEMPFERFLALLPSLKPRYYSISSSPKAHANIVSMTVGVVKASAWSGRGEYRGVASTYLAELNTGDAAACFIRTPQSGFQMPDDPDTPMIMVGPGTGIAPFRGFIQARSVLNKEGSTLGEALLYFGCRRPDHDDLYREELDQAEQEGLVTIRRCYSRVENESKGYVQHLLKQDSQKLMTLIEKGAHIYVCGDGSQMAPDVEKTLRWAYEIEKGASQEESADWLQKLQDQKRYVKDVWTGN

>CYP102A48_ortholog(BSn5_04360)Bacillus subtilis BSn5

MKQASAIPQPKTYGPLKNLPHLEKEQLSQSLWRIADELGPIFRFDFPGVSSVFVSGHNLVAEVCDESRFDKNLGKGLQKVREFGGDGLFTSWTHEPNWQKAHRILLPSFSQKAMKGYHSMMLDIATQLIQKWSRLNPNEEIDVADDMTRLTLDTIGLCGFNYRFNSFYRDSQHPFITSMLRALKEAMNQSKRLGLQDKMMVKTKLQFQKDIEVMNSLVDRMIAERKANPDDNIKDLLSLMLYAKDPVTGETLDDENIRYQIITFLIAGHETTSGLLSFAIYCLLTHPEKLKKAQEEADRVLTDDTPEYKQIQQLKYTRMVLNETLRLYPTAPAFSLYAKEDTVLGGEYPISKGQPVTVLIPKLHRDQNAWGPDAEDFRPERFEDPSSIPHHAYKPFGNGQRACIGMQFALQEATMVLGLVLKHFELINHTGYELKIKEALTIKPDDFKITVKPRKTAAINVQRKEQADIKAETKPKETKPKHGTPLLVLYGSNLGTAEGIAGELAAQGRQMGFTAETAPLDDYIGKLPEEGAVVIVTASYNGSPPDNAAGFVEWLKELEEGQLKGVSYAVFGCGNRSWASTYQRIPRLIDDMMKAKGASRLTEIGEGDAADDFESHRESWENRFWKETMDAFDINEIAQKEDRPSLSIAFLSEATETPVAKAYGAFEGVVLENRELQTADSTRSTRHIELEIPAGKTYKEGDHIGIMPKNSRELVQRVLSRFGLQSNHVIKVSGSAHMSHLPMDRPIKVADLLSSYVELQEPASRLQLRELASYTVCPPHQKELEQLVLDDGIYKEQVLAKRLTMLDFLEDYPACEMPFERFLALLPSLKPRYYSISSSPKVHANIVSMTVGVVKASAWSGRGEYRGVASNYLAELNTGDAAACFIRTPQSGFQMPDEPETPMIMVGPGTGIAPFRGFIQARSVLKKEGSTLGEALLYFGCRRPDHDDLYREELDQAEQEGLVTIRRCYSRVENESKGYVQHLLKQDSQKLMTLIEKGAHIYVCGDGSQMAPDVEKTLRWAYETEKGASQEESADWLQKLQDQKRYIKDVWTGN

>CYP102A48_ortholog(BGM20_07470)Bacillus gibsonii

MKQASAIPQPKTYGPLKNLPHLEKEQLSQSLWRIADELGPIFRFDFPGVSSVFVSGHNLVAEVCDEKRFDKNLGKGLQKVREFGGDGLFTSWTHEPNWQKAHRILLPSFSQKAMKGYHSMMLDIATQLIQKWSRLNPNEEIDVADDMTRLTLDTIGLCGFNYRFNSFYRDSQHPFITSMLRALKEAMNQSKRLGLQDKMMVKTKLQFQKDIEVMNSLVDRMIAERKANPDENIKDLLSLMLYAKDPVTGETLDDENIRYQIITFLIAGHETTSGLLSFAIYCLLTHPEKLKKAQEEADRVLTDDTPEYKQIQQLKYIRMVLNETLRLYPTAPAFSLYAKEDTVLGGEYPISKGQPVTVLIPKLHRDQNAWGPDAEDFRPERFEDPSSIPHHAYKPFGNGQRACIGMQFALQEATMVLGLVLKHFELINHTGYELKIKEALTIKPDDFKITVKPRKTAAINVQRKEQADIKAETKPKETKPKHGTPLLVLFGSNLGTAEGIAGELAAQGRQMGFTAETAPLDDYIGKLPEEGAVVIVTASYNGAPPDNAAGFVEWLKELEEGQLKGVSYAVFGCGNRSWASTYQRIPRLIDDMMKAKGASRLTAIGEGDAADDFESHRESWENRFWKETMDAFDINEIAQKEDRPSLSITFLSEATETPVAKAYGAFEGIVLENRELQTAASTRSTRHIELEIPAGKTYKEGDHIGILPKNSRELVQRVLSRFGLQSNHVIKVSGSAHMAHLPMDRPIKVADLLSSYVELQEPASRLQLRELASYTVCPPHQKELEQLVSDDGIYKEQVLAKRLTMLDFLEDYPACEMPFERFLALLPSLKPRYYSISSSPKVHANIVSMTVGVVKASAWSGRGEYRGVASNYLAELNTGDAAACFIRTPQSGFQMPDEPETPMIMVGPGTGIAPFRGFIQARSVLKKEGSTLGEALLYFGCRRPDHDDLYREELDQAEQDGLVTIRRCYSRVENEPKEYVQHLLKQDTQKLMTLIEKGAYIYVCGDGSQMAPDVEKTLRLAYEAEKGASQEESAEWLQKLQDQKRYVKDVWTGM

>CYP102A48_ortholog(BsLM_2683)Bacillus sp. LM 4-2

MKQASAIPQPKTYGPLKNLPHLEKEQLSQSLWRIADELGPIFRFDFPGVSSVFVSGHNLVAEVCDEKRFDKNLGKGLQKVREFGGDGLFTSWTHEPNWQKAHRILLPSFSQKAMKGYHSMMLDIATQLIQKWSRLNPNEEIDVADDMTRLTLDTIGLCGFNYRFNSFYRDSQHPFITSMLRALKEAMNQSKRLGLQDKMMVKTKLQFQKDIEVMNSLVDRMIAERKANPDENIKDLLSLMLYAKDPVTGETLDDENIRYQIITFLIAGHETTSGLLSFAIYCLLTHPEKLKKAQEEADRVLTDDTPEYKQIQQLKYIRMVLNETLRLYPTAPAFSLYAKEDTVLGGEYPISKGQPVTVLIPKLHRDQNAWGPDAEDFRPERFEDPSSIPHHAYKPFGNGQRACIGMQFALQEATMVLGLVLKHFELINHTGYELKIKEALTIKPDDFKITVKPRKTAAINVQRKEQADIKAETKPKETKPKHGTPLLVLFGSNLGTAEGIAGELAAQGRQMGFTAETAPLDDYIGKLPEEGAVVIVTASYNGAPPDNAAGFVEWLKELEEGQLKGVSYAVFGCGNRSWASTYQRIPRLIDDMMKAKGASRLTAIGEGDAADDFESHRESWENRFWKETMDAFDINEIAQKEDRPSLSITFLSEATETPVAKAYGAFEGIVLENRELQTAASTRSTRHIELEIPAGKTYKEGDHIGILPKNSRELVQRVLSRFGLQSNHVIKVSGSAHMAHLPMDRPIKVVDLLSSYVELQEPASRLQLRELASYTVCPPHQKELEQLVSDDGIYKEQVLAKRLTMLDFLEDYPACEMPFERFLALLPSLKPRYYSISSSPKVHANIVSMTVGVVKASAWSGRGEYRGVASNYLAELNTGDAAACFIRTPQSGFQMPNDPETPMIMVGPGTGIAPFRGFIQARSVLKKEGSTLGEALLYFGCRRPDHDDLYREELDQAEQDGLVTIRRCYSRVENEPKGYVQHLLKQDTQKLMTLIEKGAHIYVCGDGSQMAPDVERTLRLAYEAEKAASQEESAVWLQKLQDQRRYVKDVWTGM

>CYP102A48_ortholog(BS34A_29630)Bacillus sp. BS34A

MKQASAIPQPKTYGPLKNLPHLEKEQLSQSLWRIADELGPIFRFDFPGVSSVFVSGHNLVAEVCDEKRFDKNLGKGLQKVREFGGDGLFTSWTHEPNWQKAHRILLPSFSQKAMKGYHSMMLDIATQLIQKWSRLNPNEEIDVADDMTRLTLDTIGLCGFNYRFNSFYRDSQHPFITSMLRALKEAMNQSKRLGLQDKMMVKTKLQFQKDIEVMNSLVDRMIAERKANPDENIKDLLSLMLYAKDPVTGETLDDENIRYQIITFLIAGHETTSGLLSFAIYCLLTHPEKLKKAQEEADRVLTDDTPEYKQIQQLKYIRMVLNETLRLYPTAPAFSLYAKEDTVLGGEYPISKGQPVTVLIPKLHRDQNAWGPDAEDFRPERFEDPSSIPHHAYKPFGNGQRACIGMQFALQEATMVLGLVLKHFELINHTGYELKIKEALTIKPDDFKITVKPRKTAAINVQRKEQADIKAETKPKETKPKHGTPLLVLFGSNLGTAEGIAGELAAQGRQMGFTAETAPLDDYIGKLPEEGAVVIVTASYNGAPPDNAAGFVEWLKELEEGQLKGVSYAVFGCGNRSWASTYQRIPRLIDDMMKAKGASRLTAIGEGDAADDFESHRESWENRFWKETMDAFDINEIAQKEDRPSLSITFLSEATETPVAKAYGAFEGIVLENRELQTAASTRSTRHIELEIPAGKTYKEGDHIGILPKNSRELVQRVLSRFGLQSNHVIKVSGSAHMAHLPMDRPIKVVDLLSSYVELQEPASRLQLRELASYTVCPPHQKELEQLVSDDGIYKEQVLAKRLTMLDFLEDYPACEMPFERFLALLPSLKPRYYSISSSPKVHANIVSMTVGVVKASAWSGRGEYRGVASNYLAELNTGDAAACFIRTPQSGFQMPNDPETPMIMVGPGTGIAPFRGFIQARSVLKKEGSTLGEALLYFGCRRPDHDDLYREELDQAEQDGLVTIRRCYSRVENEPKGYVQHLLKQDTQKLMTLIEKGAHIYVCGDGSQMAPDVERTLRLAYEAEKAASQEESAVWLQKLQDQRRYVKDVWTGM

>CYP102A48_ortholog(QF06_11770)Bacillus sp. YP1

MKQASAIPQPKTYGPLKNLPHLEKEQLSQSLWRIADELGPIFRFDFPGVSSVFVSGHNLVAEVCDESRFDKNLGKGLQKVREFGGDGLFTSWTHEPNWQKAHRILLPSFSQKAMKGYHSMMLDIATQLIQKWSRLNPNEEIDVADDMTRLTLDTIGLCGFNYRFNSFYRDSQHPFITSMLRALKEAMNQSKRLGLQDKMMVKTKLQFQKDIEVMNSLVDRMIAERKANPDDNIKDLLSLMLYAKDPVTGETLDDENIRYQIITFLIAGHETTSGLLSFAIYCLLTHPEKLKKAQEEADRVLTDDTPEYKQIQQLKYTRMVLNETLRLYPTAPAFSLYAKEDTVLGGEYPISKGQPVTVLIPKLHRDQNAWGPDAEDFRPERFEDPSSIPHHAYKPFGNGQRACIGMQFALQEATMVLGLVLKHFELINHTGYELKIKEALTIKPDDFKITVKPRKTAAINVQRKEQADIKAETKPKETKPKHGTPLLVLYGSNLGTAEGIAGELAAQGRQMGFTAETAPLDDYIGKLPEEGAVVIVTASYNGSPPDNAAGFVEWLKELEEGQLKGVSYAVFGCGNRSWASTYQRIPRLIDDMMKAKGASRLTEIGEGDAADDFESHRESWENRFWKETMDAFDINEIAQKEDRPSLSIAFLSEATETPVAKAYGAFEGVVLENRELQTADSTRSTRHIELEIPAGKTYKEGDHIGILPKNSRELVQRVLSRFGLQSNHVIKVSGSAHMAHLPMDRPIKVVDLLSSYVELQEPASRLQLRELASYTVCPPHQKELEQLVSDDGIYKEQVLAKRLTMLDFLEDYPACEMPFERFLALLPSLKPRYYSISSSPKVHANIVSMTVGVVKASAWSGRGEYRGVASNYLAELNTGDAAACFIRTPQSGFQMPDEPETPMIMVGPGTGIAPFRGFIQARSVLKKEGSTLGEALLYFGCRRPDHDDLYREELDQAEQDGLVTIRRCYSRVENEPKEYVQHLLKQDTQKLMTLIEKGAHIYVCGDGSQMAPDVEKTLRLAYEAEKGASQEESAEWLQKLQDQKRYVKDVWTGM

>CYP102A48_ortholog(U712_13335)Bacillus subtilis PY79

MKQASAIPQPKTYGPLKNLPHLEKEQLSQSLWRIADELGPIFRFDFPGVSSVFVSGHNLVAEVCDEKRFDKNLGKGLQKVREFGGDGLFTSWTHEPNWQKAHRILLPSFSQKAMKGYHSMMLDIATQLIQKWSRLNPNEEIDVADDMTRLTLDTIGLCGFNYRFNSFYRDSQHPFITSMLRALKEAMNQSKRLGLQDKMMVKTKLQFQKDIEVMNSLVDRMIAERKANPDENIKDLLSLMLYAKDPVTGETLDDENIRYQIITFLIAGHETTSGLLSFAIYCLLTHPEKLKKAQEEADRVLTDDTPEYKQIQQLKYIRMVLNETLRLYPTAPAFSLYAKEDTVLGGEYPISKGQPVTVLIPKLHRDQNAWGPDAEDFRPERFEDPSSIPHHAYKPFGNGQRACIGMQFALQEATMVLGLVLKHFELINHTGYELKIKEALTIKPDDFKITVKPRKTAAINVQRKEQADIKAETKPKETKPKHGTPLLVLFGSNLGTAEGIAGELAAQGRQMGFTAETAPLDDYIGKLPEEGAVVIVTASYNGAPPDNAAGFVEWLKELEEGQLKGVSYAVFGCGNRSWASTYQRIPRLIDDMMKAKGASRLTAIGEGDAADDFESHRESWENRFWKETMDAFDINEIAQKEDRPSLSITFLSEATETPVAKAYGAFEGIVLENRELQTAASTRSTRHIELEIPAGKTYKEGDHIGILPKNSRELVQRVLSRFGLQSNHVIKVSGSAHMAHLPMDRPIKVVDLLSSYVELQEPASRLQLRELASYTVCPPHQKELEQLVSDDGIYKEQVLAKRLTMLDFLEDYPACEMPFERFLALLPSLKPRYYSISSSPKVHANIVSMTVGVVKASAWSGRGEYRGVASNYLAELNTGDAAACFIRTPQSGFQMPNDPETPMIMVGPGTGIAPFRGFIQARSVLKKEGSTLGEALLYFGCRRPDHDDLYREELDQAEQDGLVTIRRCYSRVENEPKGYVQHLLKQDTQKLMTLIEKGAHIYVCGDGSQMAPDVERTLRLAYEAEKAASQEESAVWLQKLQDQRRYVKDVWTGM

>CYP102A48_ortholog(BSU6051_27160)Bacillus subtilis subsp. subtilis 6051-HGW

MKQASAIPQPKTYGPLKNLPHLEKEQLSQSLWRIADELGPIFRFDFPGVSSVFVSGHNLVAEVCDEKRFDKNLGKGLQKVREFGGDGLFTSWTHEPNWQKAHRILLPSFSQKAMKGYHSMMLDIATQLIQKWSRLNPNEEIDVADDMTRLTLDTIGLCGFNYRFNSFYRDSQHPFITSMLRALKEAMNQSKRLGLQDKMMVKTKLQFQKDIEVMNSLVDRMIAERKANPDENIKDLLSLMLYAKDPVTGETLDDENIRYQIITFLIAGHETTSGLLSFAIYCLLTHPEKLKKAQEEADRVLTDDTPEYKQIQQLKYIRMVLNETLRLYPTAPAFSLYAKEDTVLGGEYPISKGQPVTVLIPKLHRDQNAWGPDAEDFRPERFEDPSSIPHHAYKPFGNGQRACIGMQFALQEATMVLGLVLKHFELINHTGYELKIKEALTIKPDDFKITVKPRKTAAINVQRKEQADIKAETKPKETKPKHGTPLLVLFGSNLGTAEGIAGELAAQGRQMGFTAETAPLDDYIGKLPEEGAVVIVTASYNGAPPDNAAGFVEWLKELEEGQLKGVSYAVFGCGNRSWASTYQRIPRLIDDMMKAKGASRLTAIGEGDAADDFESHRESWENRFWKETMDAFDINEIAQKEDRPSLSITFLSEATETPVAKAYGAFEGIVLENRELQTAASTRSTRHIELEIPAGKTYKEGDHIGILPKNSRELVQRVLSRFGLQSNHVIKVSGSAHMAHLPMDRPIKVVDLLSSYVELQEPASRLQLRELASYTVCPPHQKELEQLVSDDGIYKEQVLAKRLTMLDFLEDYPACEMPFERFLALLPSLKPRYYSISSSPKVHANIVSMTVGVVKASAWSGRGEYRGVASNYLAELNTGDAAACFIRTPQSGFQMPNDPETPMIMVGPGTGIAPFRGFIQARSVLKKEGSTLGEALLYFGCRRPDHDDLYREELDQAEQDGLVTIRRCYSRVENEPKGYVQHLLKQDTQKLMTLIEKGAHIYVCGDGSQMAPDVERTLRLAYEAEKAASQEESAVWLQKLQDQRRYVKDVWTGM

>CYP102A48_ortholog(BSUA_02898)Bacillus subtilis subsp. subtilis JH642

MKQASAIPQPKTYGPLKNLPHLEKEQLSQSLWRIADELGPIFRFDFPGVSSVFVSGHNLVAEVCDEKRFDKNLGKGLQKVREFGGDGLFTSWTHEPNWQKAHRILLPSFSQKAMKGYHSMMLDIATQLIQKWSRLNPNEEIDVADDMTRLTLDTIGLCGFNYRFNSFYRDSQHPFITSMLRALKEAMNQSKRLGLQDKMMVKTKLQFQKDIEVMNSLVDRMIAERKANPDENIKDLLSLMLYAKDPVTGETLDDENIRYQIITFLIAGHETTSGLLSFAIYCLLTHPEKLKKAQEEADRVLTDDTPEYKQIQQLKYIRMVLNETLRLYPTAPAFSLYAKEDTVLGGEYPISKGQPVTVLIPKLHRDQNAWGPDAEDFRPERFEDPSSIPHHAYKPFGNGQRACIGMQFALQEATMVLGLVLKHFELINHTGYELKIKEALTIKPDDFKITVKPRKTAAINVQRKEQADIKAETKPKETKPKHGTPLLVLFGSNLGTAEGIAGELAAQGRQMGFTAETAPLDDYIGKLPEEGAVVIVTASYNGAPPDNAAGFVEWLKELEEGQLKGVSYAVFGCGNRSWASTYQRIPRLIDDMMKAKGASRLTAIGEGDAADDFESHRESWENRFWKETMDAFDINEIAQKEDRPSLSITFLSEATETPVAKAYGAFEGIVLENRELQTAASTRSTRHIELEIPAGKTYKEGDHIGILPKNSRELVQRVLSRFGLQSNHVIKVSGSAHMAHLPMDRPIKVVDLLSSYVELQEPASRLQLRELASYTVCPPHQKELEQLVSDDGIYKEQVLAKRLTMLDFLEDYPACEMPFERFLALLPSLKPRYYSISSSPKVHANIVSMTVGVVKASAWSGRGEYRGVASNYLAELNTGDAAACFIRTPQSGFQMPNDPETPMIMVGPGTGIAPFRGFIQARSVLKKEGSTLGEALLYFGCRRPDHDDLYREELDQAEQDGLVTIRRCYSRVENEPKGYVQHLLKQDTQKLMTLIEKGAHIYVCGDGSQMAPDVERTLRLAYEAEKAASQEESAVWLQKLQDQRRYVKDVWTGM

>CYP102A48_ortholog(BSUB_02898)Bacillus subtilis subsp. subtilis AG1839

MKQASAIPQPKTYGPLKNLPHLEKEQLSQSLWRIADELGPIFRFDFPGVSSVFVSGHNLVAEVCDEKRFDKNLGKGLQKVREFGGDGLFTSWTHEPNWQKAHRILLPSFSQKAMKGYHSMMLDIATQLIQKWSRLNPNEEIDVADDMTRLTLDTIGLCGFNYRFNSFYRDSQHPFITSMLRALKEAMNQSKRLGLQDKMMVKTKLQFQKDIEVMNSLVDRMIAERKANPDENIKDLLSLMLYAKDPVTGETLDDENIRYQIITFLIAGHETTSGLLSFAIYCLLTHPEKLKKAQEEADRVLTDDTPEYKQIQQLKYIRMVLNETLRLYPTAPAFSLYAKEDTVLGGEYPISKGQPVTVLIPKLHRDQNAWGPDAEDFRPERFEDPSSIPHHAYKPFGNGQRACIGMQFALQEATMVLGLVLKHFELINHTGYELKIKEALTIKPDDFKITVKPRKTAAINVQRKEQADIKAETKPKETKPKHGTPLLVLFGSNLGTAEGIAGELAAQGRQMGFTAETAPLDDYIGKLPEEGAVVIVTASYNGAPPDNAAGFVEWLKELEEGQLKGVSYAVFGCGNRSWASTYQRIPRLIDDMMKAKGASRLTAIGEGDAADDFESHRESWENRFWKETMDAFDINEIAQKEDRPSLSITFLSEATETPVAKAYGAFEGIVLENRELQTAASTRSTRHIELEIPAGKTYKEGDHIGILPKNSRELVQRVLSRFGLQSNHVIKVSGSAHMAHLPMDRPIKVVDLLSSYVELQEPASRLQLRELASYTVCPPHQKELEQLVSDDGIYKEQVLAKRLTMLDFLEDYPACEMPFERFLALLPSLKPRYYSISSSPKVHANIVSMTVGVVKASAWSGRGEYRGVASNYLAELNTGDAAACFIRTPQSGFQMPNDPETPMIMVGPGTGIAPFRGFIQARSVLKKEGSTLGEALLYFGCRRPDHDDLYREELDQAEQDGLVTIRRCYSRVENEPKGYVQHLLKQDTQKLMTLIEKGAHIYVCGDGSQMAPDVERTLRLAYEAEKAASQEESAVWLQKLQDQRRYVKDVWTGM

>CYP102A48_ortholog(B657_27160)Bacillus subtilis QB928

MKQASAIPQPKTYGPLKNLPHLEKEQLSQSLWRIADELGPIFRFDFPGVSSVFVSGHNLVAEVCDEKRFDKNLGKGLQKVREFGGDGLFTSWTHEPNWQKAHRILLPSFSQKAMKGYHSMMLDIATQLIQKWSRLNPNEEIDVADDMTRLTLDTIGLCGFNYRFNSFYRDSQHPFITSMLRALKEAMNQSKRLGLQDKMMVKTKLQFQKDIEVMNSLVDRMIAERKANPDENIKDLLSLMLYAKDPVTGETLDDENIRYQIITFLIAGHETTSGLLSFAIYCLLTHPEKLKKAQEEADRVLTDDTPEYKQIQQLKYIRMVLNETLRLYPTAPAFSLYAKEDTVLGGEYPISKGQPVTVLIPKLHRDQNAWGPDAEDFRPERFEDPSSIPHHAYKPFGNGQRACIGMQFALQEATMVLGLVLKHFELINHTGYELKIKEALTIKPDDFKITVKPRKTAAINVQRKEQADIKAETKPKETKPKHGTPLLVLFGSNLGTAEGIAGELAAQGRQMGFTAETAPLDDYIGKLPEEGAVVIVTASYNGAPPDNAAGFVEWLKELEEGQLKGVSYAVFGCGNRSWASTYQRIPRLIDDMMKAKGASRLTAIGEGDAADDFESHRESWENRFWKETMDAFDINEIAQKEDRPSLSITFLSEATETPVAKAYGAFEGIVLENRELQTAASTRSTRHIELEIPAGKTYKEGDHIGILPKNSRELVQRVLSRFGLQSNHVIKVSGSAHMAHLPMDRPIKVVDLLSSYVELQEPASRLQLRELASYTVCPPHQKELEQLVSDDGIYKEQVLAKRLTMLDFLEDYPACEMPFERFLALLPSLKPRYYSISSSPKVHANIVSMTVGVVKASAWSGRGEYRGVASNYLAELNTGDAAACFIRTPQSGFQMPNDPETPMIMVGPGTGIAPFRGFIQARSVLKKEGSTLGEALLYFGCRRPDHDDLYREELDQAEQDGLVTIRRCYSRVENEPKGYVQHLLKQDTQKLMTLIEKGAHIYVCGDGSQMAPDVERTLRLAYEAEKAASQEESAVWLQKLQDQRRYVKDVWTGM

>CYP102A48_ortholog(BSU27160) Bacillus subtilis subsp. subtilis 168

MKQASAIPQPKTYGPLKNLPHLEKEQLSQSLWRIADELGPIFRFDFPGVSSVFVSGHNLVAEVCDEKRFDKNLGKGLQKVREFGGDGLFTSWTHEPNWQKAHRILLPSFSQKAMKGYHSMMLDIATQLIQKWSRLNPNEEIDVADDMTRLTLDTIGLCGFNYRFNSFYRDSQHPFITSMLRALKEAMNQSKRLGLQDKMMVKTKLQFQKDIEVMNSLVDRMIAERKANPDENIKDLLSLMLYAKDPVTGETLDDENIRYQIITFLIAGHETTSGLLSFAIYCLLTHPEKLKKAQEEADRVLTDDTPEYKQIQQLKYIRMVLNETLRLYPTAPAFSLYAKEDTVLGGEYPISKGQPVTVLIPKLHRDQNAWGPDAEDFRPERFEDPSSIPHHAYKPFGNGQRACIGMQFALQEATMVLGLVLKHFELINHTGYELKIKEALTIKPDDFKITVKPRKTAAINVQRKEQADIKAETKPKETKPKHGTPLLVLFGSNLGTAEGIAGELAAQGRQMGFTAETAPLDDYIGKLPEEGAVVIVTASYNGAPPDNAAGFVEWLKELEEGQLKGVSYAVFGCGNRSWASTYQRIPRLIDDMMKAKGASRLTAIGEGDAADDFESHRESWENRFWKETMDAFDINEIAQKEDRPSLSITFLSEATETPVAKAYGAFEGIVLENRELQTAASTRSTRHIELEIPAGKTYKEGDHIGILPKNSRELVQRVLSRFGLQSNHVIKVSGSAHMAHLPMDRPIKVVDLLSSYVELQEPASRLQLRELASYTVCPPHQKELEQLVSDDGIYKEQVLAKRLTMLDFLEDYPACEMPFERFLALLPSLKPRYYSISSSPKVHANIVSMTVGVVKASAWSGRGEYRGVASNYLAELNTGDAAACFIRTPQSGFQMPNDPETPMIMVGPGTGIAPFRGFIQARSVLKKEGSTLGEALLYFGCRRPDHDDLYREELDQAEQDGLVTIRRCYSRVENEPKGYVQHLLKQDTQKLMTLIEKGAHIYVCGDGSQMAPDVERTLRLAYEAEKAASQEESAVWLQKLQDQRRYVKDVWTGM

>CYP102A48_ortholog(I33_2757)Bacillus subtilis subsp. subtilis RO-NN-1

MKQASAIPQPKTYGPLKNLPHLEKEQLSQSLWRIADELGPIFRFDFPGVSSVFVSGHNLVAEVCDESRFDKNLGKGLQKVREFGGDGLFTSWTHEPNWQKAHRILLPSFSQKAMKGYHSMMLDIATQLIQKWSRLNPNEEIDVADDMTRLTLDTIGLCGFNYRFNSFYRDSQHPFITSMLRALKEAMNQSKRLGLQDKMMVKTKLQFQKDIEVMNSLVDRMIAERKANPDDNIKDLLSLMLYAKDPVTGETLDDENIRYQIITFLIAGHETTSGLLSFAIYCLLTHPEKLKKAQEEADRVLTDDTPEYKQIQQLKYIRMVLNETLRLYPTAPAFSLYAKEDTVLGGEYPISKGQPVTVLIPKLHRDQNAWGPDAEDFRPERFEDPSSIPHHAYKPFGNGQRACIGMQFALQEATMVLGLVLKHFELINHTGYELKIKEALTIKPDDFKITVKPRKTAAINVQRKEQADIKAETKPKETKPKHGTPLLVLYGSNLGTAEGIAGELAAQGRQMGFTAETAPLDDYIGKLPEEGAVVIVTASYNGAPPDNAAGFVEWLKELEEGQLKGVSYAVFGCGNRSWASTYQRIPRLIDDMMKAKGASRLTAIGEGDAADDFESHRESWENRFWKETMDAFDINEIAQKEDRPSLSITFLSEATETPVAKAYGAFEGIVLENRELQTADSTRSTRHIELEIPDGKTYKEGDHIGILPKNSRELVQRVLSRFGLQSNHVIKVSGSAHMAHLPMDRPIRVVDLLSSYVELQEPASRLQLRELASYTVCPPHQKELEQLVSDDGIYKEHVLAKRLTMLDFLEDYPACEMPFERFLALLPSLKPRYYSISSSPKVHANIVSMTVGVVKASAWSGRGEYRGVASNYLAELNTGDAAACFIRTPQSGFQMPDELETPMIMVGPGTGIAPFRGFIQARSVLKKEGSTLGEALLYFGCRRPDHDDLYREELDQAEQDGLVTIRRCYSRVENEPKEYVQHLLKQDTQKLMTLIEKGAHIYVCGDGSQMAPDVEKTLRLAYEAEKGASQEESAEWLQKLQDQRRYVKDVWTGM

>CYP102A48_ortholog(Q433_14610)Bacillus subtilis subsp. subtilis OH 131.1

MKQASAIPQPKTYGPLKNLPHLEKEQLSQSLWRIADELGPIFRFDFPGVSSVFVSGHNFVAEVCDESRFDKNLGKGLQKVREFGGDGLFTSWTHEPNWQKAHRILLPSFSQKAMKGYHSMMLDIATQLIQKWSRLNPNEEIDVADDMTRLTLDTIGLCGFNYRFNSFYRDSQHPFITSMLRALKEAMNQSKRLGLQDKMMVKTKLQFQKDIEVMNSLVDRMIAERKANPDENIKDLLSLMLYAKDPVTGETLDDENIRYQIITFLIAGHETTSGLLSFAIYCLLTHPEKLKKAQEEADCVLTDDTPEYKQIQQLKYIRMVLNETLRLYPTAPAFSLYAKEDTVLGGEYPISKGQPVTVLIPKLHRDQNAWGPDAEDFRPERFEDPSSIPHHAYKPFGNGQRACIGMQFALQEATMVLGLVLKHFELINHTGYELKIKEALTIKPDDFKITVKPRKTAAINVQRKEQADIKAETKPKETKPKHGTPLLVLYGSNLGTAEGIAGELVAQGCQMGFTAETAPLDDYIGKLPEEGAVVIVTASYNGAPPDNAAGFVEWLKELEEGQLKGVSYAVFGCGNRSWASTYQRIPRLIDDMMKAKGASRLTAIGEGDAADDFESHRESWENRFWKETMDAFDINELAQKEDRPSLSITFLSEATETPVAKAYGAFEGIVLENRELQTAASTRSTRHIELEIPAGKTYKEGDHIGILPKNSRELVQRVLSRFGLQSNHVIKVSGSAHMAHLPMDRPIKVVDLLSSYVELQEPASRLQLRELASYTVCPPHQKELEQLVSDDGIYKEQVLAKRLTMLDFLEDYPACEMPFERFLALLPSLKPRYYSISSSPKVHANIVSMTVGVVKASAWSGRGEYRGVASNYLAELNTGDAAACFIRTPQSGFQMPDEPETPMIMIGPGTGIAPFRGFIQARSVLKKEGSTLGEALLYFGCRRPDHDDLYREELDQAEQDGLVTIRRCYSRVENEPKGYVQHLLKQDTQKLMTLIEKGAHIYVCGDGSQMAPDVEKTLRLAYEAEKGASQEESAEWLQKLQDQRRYVKDVWTGM

>CYP102A48_ortholog(C663_2550)Bacillus subtilis XF-1

MLMKQASAIPQPKTYGPLKNLPHLEKEQLSQSLWRIADELGPIFRFDFPGVSSVFVSGHNLVAEVCDESRFDKNLGKGLQKVREFGGDGLFTSWTHEPNWQKAHRILLPSFSQKAMKGYHSMMLDIATQLIQKWSRLNPNEEIDVADDMTRLTLDTIGLCGFNYRFNSFYRDSQHPFITSMLRALKEAMNQSKRLGLQDKMMVKTKLQFQKDIEVMNSLVDRMIAERKANPDEDIKDLLSLMLYAKDPVTGETLDDENIRYQIITFLIAGHETTSGLLSFAIYCLLTHPEKLKKAQEEADRVLTDDTPEYKQIQQLKYIRMVLNETLRLYPTAPAFSLYAKEDTVLGGEYPISKGQPVTVLIPKLHRDQNAWGPDAEDFRPERFEDPSSIPHHAYKPFGNGQRACIGMQFALQEATMVLGLVLKHFELINHTGYELKIKEALTIKPDDFKITVKPRKTAAINVQRKEQADIKAETKPKETKPIHGTPLLVLYGSNLGTAEGIAGELAAQGRQMGFAAETAPLDDYIGKLPEEGAVVIVTASYNGAPPDNAAGFVEWLKELKEGQLKGVSYAVFGCGNRSWASTYQRIPRLIDDMMKAKGASRLTEIGEGDAADDFESHRESWENRFWKETMDAFDINEIAQKEDRPSLSITFLSETTETPVAKAYGAFEGVVLENRELQTADSTRSTRHIELEIPAGKTYKEGDHIGIMPKNSRELVQRVLSRFGLQSNHVIKVSGSAHMAHLPMDRPIKVVDLLSSYVELQEPASRLQLRELASYTVCPPHQKELEQLVSDDGIYKEQVLAKRLTMLDFLEDYPACEMPFERFLALLPSLKPRYYSISSSPKVHANIVSMTVGVVKASAWSGRGEYRGVASNYLAELNTGDAAACFIRTPQSGFQMPDESETPMIMVGPGTGIAPFRGFIQARSVLKKEGSTLGEALLYFGCRRPDHDDLYREELDQAEQDGLVTIRRCYSRVENEPKEYVQHLLKQDTQKLMTLIEKGAHIYVCGDGSQMAPDVEKTLRLAYEAEKGASQEESAEWLQKLQDQKRYVKDVWTGM

>CYP102A48_ortholog(I653_12890)Bacillus subtilis subsp. subtilis BAB-1

MKQASAIPQPKTYGPLKNLPHLEKEQLSQSLWRIADELGPIFRFDFPGVSSVFVSGHNLVAEVCDESRFDKNLGKGLQKVREFGGDGLFTSWTHEPNWQKAHRILLPSFSQKAMKGYHSMMLDIATQLIQKWSRLNPNEEIDVADDMTRLTLDTIGLCGFNYRFNSFYRDSQHPFITSMLRALKEAMNQSKRLGLQDKMMVKTKLQFQKDIEVMNSLVDRMIAERKANPDEDIKDLLSLMLYAKDPVTGETLDDENIRYQIITFLIAGHETTSGLLSFAIYCLLTHPEKLKKAQEEADRVLTDDTPEYKQIQQLKYIRMVLNETLRLYPTAPAFSLYAKEDTVLGGEYPISKGQPVTVLIPKLHRDQNAWGPDAEDFRPERFEDPSSIPHHAYKPFGNGQRACIGMQFALQEATMVLGLVLKHFELINHTGYELKIKEALTIKPDDFKITVKPRKTAAINVQRKEQADIKAETKPKETKPIHGTPLLVLYGSNLGTAEGIAGELAAQGRQMGFAAETAPLDDYIGKLPEEGAVVIVTASYNGAPPDNAAGFVEWLKELKEGQLKGVSYAVFGCGNRSWASTYQRIPRLIDDMMKAKGASRLTEIGEGDAADDFESHRESWENRFWKETMDAFDINEIAQKEDRPSLSITFLSETTETPVAKAYGAFEGVVLENRELQTADSTRSTRHIELEIPAGKTYKEGDHIGIMPKNSRELVQRVLSRFGLQSNHVIKVSGSAHMAHLPMDRPIKVVDLLSSYVELQEPASRLQLRELASYTVCPPHQKELEQLVSDDGIYKEQVLAKRLTMLDFLEDYPACEMPFERFLALLPSLKPRYYSISSSPKVHANIVSMTVGVVKASAWSGRGEYRGVASNYLAELNTGDAAACFIRTPQSGFQMPDESETPMIMVGPGTGIAPFRGFIQARSVLKKEGSTLGEALLYFGCRRPDHDDLYREELDQAEQDGLVTIRRCYSRVENEPKEYVQHLLKQDTQKLMTLIEKGAHIYVCGDGSQMAPDVEKTLRLAYEAEKGASQEESAEWLQKLQDQKRYVKDVWTGM

>CYP102A48_ortholog(A7A1_0357)Bacillus subtilis subsp. subtilis BSP1

KQASAIPQPKTYGPLKNLPHLEKEQLSQSLWRIADELGPIFRFDFPGVSSVFVSGHNFVAEVCDESRFDKNLGKGLQKVREFGGDGLFTSWTHEPNWQKAHRILLPSFSQKAMKGYHSMMLDIATQLIQKWSRLNPNEEIDVADDMTRLTLDTIGLCGFNYRFNSFYRDSQHPFITSMLRALKEAMNQSKRLGLQDKMMVKTKLQFQKDIEVMNSLVDRMIAERKANPDENIKDLLSLMLYAKDPVTGETLDDENIRYQIITFLIAGHETTSGLLSFAIYCLLTHPEKLKKAQEEADCVLTDDTPEYKQIQQLKYIRMVLNETLRLYPTAPAFSLYAKEDTVLGGEYPISKGQPVTVLIPKLHRDQNAWGPDAEDFRPERFEDPSSIPHHAYKPFGNGQRACIGMQFALQEATMVLGLVLKHFELINHTGYELKIKEALTIKPDDFKITVKPRKTAAINVQRKEQADIKAETKPKETKPKHGTPLLVLYGSNLGTAEGIAGELVAQGCQMGFTAETAPLDDYIGKLPEEGAVVIVTASYNGAPPDNAAGFVEWLKELEEGQLKGVSYAVFGCGNRSWASTYQRIPRLIDDMMKAKGASRLTAIGEGDAADDFESHRESWENRFWKETMDAFDINELAQKEDRPSLSITFLSEATETPVAKAYGAFEGIVLENRELQTAASTRSTRHIELEIPAGKTYKEGDHIGILPKNSRELVQRVLSRFGLQSNHVIKVSGSAHMAHLPMDRPIKVVDLLSSYVELQEPASRLQLRELASYTVCPPHQKELEQLVSDDGIYKEQVLAKRLTMLDFLEDYPACEMPFERFLALLPSLKPRYYSISSSPKVHANIVSMTVGVVKASAWSGRGEYRGVASNYLAELNTGDAAACFIRTPQSGFQMPDEPETPMIMIGPGTGIAPFRGFIQARSVLKKEGSTLGEALLYFGCRRPDHDDLYREELDQAEQDGLVTIRRCYSRVENEPKGYVQHLLKQDTQKLMTLIEKGAHIYVCGDGSQMAPDVEKTLRLAYEAEKGASQEESAEWLQKLQDQRRYVKDVWTGM

>CYP102A48_ortholog(BSUW23_13125)Bacillus subtilis subsp. spizizenii W23

MKQASAIPQPKTYGPLKNLPHLEKEQLSQSLWRIADELGPIFRFDFPGVSSVFVSGHNLVAEVCDESRFDKNLGKGLLKVREFGGDGLFTSWTNEPNWQKAHRILLPSFSQKAMKGYHSMMLDIATQLIQKWSRLNPNEEIDVADDMTRLTLDTIGLCGFNYRFNSFYRDSQHPFITSMLRALKEAMNQSKRLGLQDKMMVKTKLQFQKDIEVMNSLVDRMIAERKANPDENIKDLLSLMLYAKDPVTGETLDDENIRYQIITFLIAGHETTSGLLSFAIYCLLTHPEKLKKAQEEADRVLTDDTPEYRQIQQLKYIRMVLNETLRLYPTAPAFSLYAKEDTVLGGEYPISKGQPVTVLIPKLHRDQNAWGEDAEDFRPERFEDPSRIPHHAYKPFGNGQRACIGMQFALQEATMVLGLVLKHFDVMNHTGYELKIKEALTIKPDDFKITVKPRKTAAINVQRKEQADIKTETKPKETKPKHGTPLLVLYGSNLGTAEGIAGELAAYGRQMGFTAETAPLDDYIGKLLEQGAVVIVTASYNGAPPDNAAGFVEWLEGLEEGRLNGVSYAVFGCGNRSWASTYQRIPRLIDDMMKAKGASRLTAIGEGDAADDFESHRESWENRFWKETMEAFAINEISQKEDRPSLSIAFLSEATETPLAKAYGAFEGNVLENRELQTADSPRSTRHIELQIPDAKTYKEGDHIGILPKNSQKLVQRVLSRFDLQSNHVIKITGGPHMAHLPMDRPIKVTDLLASYVELQDPASRLQLRELASYTVCPPHKKELEQLVSDDGIYKEQVLAKRLTMLDLLEDYPACEMPFERFLALLPSLKPRYYSISSSPKVHANIVSMTVGVVKASAWSGRGEYRGVASNYLAELNTGDAAACFIRTPQSGFQMPDDPETPIIMVGPGTGIAPFRGFIQARSVLKKEGSALGEALLYFGCRHPDHDDLYREELDQAEQDGLVTIRRCYSRVENESKGYVQHLLKQDTQKLMSLIEKGASIYVCGDGSQMAPDVENSLRQAYETEKGASEEESADWLQKLQDQKRYVKDVWTGM

>CYP102A48_ortholog(GYO_2953)Bacillus subtilis subsp. spizizenii TU-B-10

MKQASAIPQPKTYGPLKNLPHLEKEQLSQSLWRIADELGPIFRFDFPGVSSVFVSGHNLVAEVCDESRFDKNLGKGLLKVREFGGDGLFTSWTHEPNWQKAHRILLPSFSQKAMKGYHSMMLDIATQLIQKWSRLNPNEEIDVADDMTRLTLDTIGLCGFNYRFNSFYRDSQHPFITSMLRALKEAMNQSKRLGLQDKMMVKTKLQFQKDIEVMNSLVDRLIAERKANPDENIKDLLSLMLYAKDPVTGETLDDENIRYQIITFLIAGHETTSGLLSFAIYCLLTHPEKLKKAQEEADRVLTDDTPEYRQIQQLKYIRMILNETLRLYPTAPAFSLYAKEDTVLGGEYPISKGQPVTVLIPKLHRDQNAWGEDAEAFRPERFEDPSRIPHHAYKPFGNGQRACIGMQFALQEATMVLGLVLKHFDVMNHTGYELKIKEALTIKPDEFKITVKPRKTAAINVQRKEQADIKTETKPKETKPKHGTPLLVLYGSNLGTAEGIAGELAAYGRQMGFTAETAPLDDYIGKLPEQGAVVIVTASYNGAPPDNAAGFVEWLEGLEEGQLKGVSYAVFGCGNRSWASTYQRIPRLIDDMMKAKGASRLTAIGEGDAADDFESHRESWENRFWKETMEAFHINEIAQKEDRPSLSITFLSEATETPLAKAYGAFEGNVLENRELQTADSPRSTRHIELQIPDAKTYKEGDHIGILPKNSQKLVQRVLSRFDLQSNHVMKMSGGPHMAHLLMDRPIKVMDLLASYVELQDPASRLQLRELASYTVCPPHKKELEQLVSDDGIYKEQVLAKRLTMLDFLEDYPACEMPFERFLALLPSLKPRYYSISSSPKVHANIVSMTVGVVKDSAWSGRGEYRGVASNYLAELNTGDAAACFIRTPQSGFQMPDDPETPMIMVGPGTGIAPFRGFIQARSVLKKEGSALGEALLYFGCRRPDHDDLYREELDQAEQDGLVTIRRCYSRVENESKEYVQHLLKQDTQKLMSLIEKGAHIYVCGDGSQMAPDVENTLRQAYEAEKGASQEESADWLEKLQDQKRYVKDVWTGM

>CYP102A48_ortholog(BSNT_09105)Bacillus subtilis subsp. natto BEST195

MLTDDTPEYKQIQQLKYTRMVLNETLRLYPTAPAFSLYAKEDTVLGGEYPISKGQPVTVLIPKLHRDQNAWGPDAEDFRPERFEDPSSIPHHAYKPFGNGQRACIGMQFALQEATMVLGLVLKHFELINHTGYELKIKEALTIKPDDFKITVKPRKTAAINVQRKEQAEIKAETKPKETKPKHGTPLLVLYGSNLGTAEGIADELASQGRQMGFTAETAPLDDYIGKLPEEGAVVIVTASYNGSPPDNAAGFVEWLKELEEGQLKGVSYAVFGCGNRSWASTYQRIPRLIDDMMKAKGASRLTEIGEGDAADDFESHRESWENRFWKETMDAFDINEIAQKEDRPSLSIAFLSEATETPVAKAYGAFEGVVLENRELQTADSTRSTCHIELEIPAGKTYKEGDHIGILPKNSRELVQRVLSRFGLQSNHVIKVSGSAHMAHLPMDRPIKVADLLSSYVELQEPASRLQLRELASYTVCPPHQKELEQLVSDDGIYKEQVLAKRLTMLDFLEDYPACEMPFERFLALLPSLKPRYYSISSSPKVHANIVSMTVGVVKASAWSGRGEYRGVASNYLAELNTGDAAACFIRTPQSGFQMPDEPETPMIMVGPGTGIAPFRGFIQARSVLKKEGSTLGEALLYFGCRRPDHDDLYREELDQAEQEGLVTIRRCYSRVKNEPKEYVQHLLKQDTQKLMTLIEKGAHIYVCGDGSQMAPDVEKTLRLAYEAEKGASQEESAEWLQKLQDQRRYVKDVWTGM

>CYP102A49(SB24_16560)Bacillus sp. Pc3

MKQLSAIPQPKTYGPLKNLPHLEKEKLSQSLWKIAEEYGPIFRFEFPSSVGVFVSGRELAAEVCDEKRFDKNLSKALLKVREFGGDGLFTSWTHEKNWQKAHRILLPSFSQKAMKGYHSMMLDIAMQLVQKWSRLNPNEEIDVAEDMTRLTLDTIGLCGFHYRFNSFYRDTQHPFITSMLRALQEAMRQSQRHSLQDKLMIKTRHQFQQDIEEMNSLVDRIIAERRENPDENLSDLLALMLEAKDPVTGERLDDENIRYQIITFLIAGHETTSGLLSFAIYCLLKNKDKLKKAVQEAERVLTGETPEYKQIQQLTYIRMVLNETLRLYPTAPAFSLYAKEDTVLGGKYPIAKGQPVTILTPQLHRDKSAWGEDAELFRPERFSDPAAIPADAYKPFGNGQRACIGMQFALQEATMVLGLVLKHFELIDHTDYELTIKEALTIKPGDFKIRVKNKDVSNHQPVQNHKAEGSGHKEETKEIPSHGTPLLILYGSNLGTAEGIAEELADIGRSKGFSTETGPLDDYAGKLPVKGAVVIVTASYNGAPPDNAAGFVKWMETLEDQELKGVSYAVFGCGDRNWAATYQRIPRLIDKVLEEKGAKRLTSIGEGDNADDFEYSQEVWEDSFWKDIMKAFHIEAAPKQTNKSQLSIEYVSEAAETPIAKTYKAFEAEVITNKELHTESSKRSVRHIELRLPETETYQEGDHLGVLPQNSGELISRVIRRFGLDPNQHFKIKGRQLPHLPMDRPVNAPELLASYVELQEPATRAQLRELAAHTVCPPHQKELEHLYSDDAAYKENVLKKRMTMLDLLEDYPACELPFERFLELLPSLKARYYSISSSPKAASGELSITVGVVTAPAWSGRGEYRGVASNYLAGLQKGDSAVCFIRSPQSGFALPENPKTPLIMVGAGTGIAPFRGFIQARAAEKMSGNSLGEAHLYFGCRHPEEDDLYKDEFDHAEKNGLVTVHRAYSRLNQDCKVYVQDVLLREAAQIIALLDQGGHLYICGDGSKMAPAVENVLLQAYEKVHNTDSKVSLEWLEQLQAEGRYAKDVWAGM

>CYP102A49_ortholog(BAM5036_2365)Bacillus velezensis UCMB5036

MKQLSAIPQPKTYGPLKNLPHLEKEKLSQSLWKIAEEYGPIFRFEFPSSVGVFVSGRELAAEVCDEKRFDKNLSKALLKVREFGGDGLFTSWTHEKNWQKAHRILLPSFSQKAMKGYHSMMLDIAMQLVQKWSRLNPNEEIDVAEDMTRLTLDTIGLCGFHYRFNSFYRDTQHPFITSMLRALQEAMRQSQRHSLQDKLMIKTRHQFQQDIEEMNSLVDRIIAERRENPDENLSDLLALMLEAKDPVTGERLDDENIRYQIITFLIAGHETTSGLLSFAIYCLLKNKDKLKKAVQEAERVLTGETPEYKQIQQLTYIRMVLNETLRLYPTAPAFSLYAKEDTVLGGKYPIAKGQPVTILTPQLHRDKSAWGEDAELFRPERFSDPAAIPADAYKPFGNGQRACIGMQFALQEATMVLGLVLKHFELIDHTDYELTIKEALTIKPGDFKIRVKNKDVSNHQPVQNHKAEGSGHKEETKEIPSHGTPLLILYGSNLGTAEGIAEELADIGRSKGFSTETGPLDDYAGKLPVKGAVVIVTASYNGAPPDNAAGFVKWMETLEDQELKGVSYAVFGCGDRNWAATYQRIPRLIDKVLEEKGAKRLTSIGEGDNADDFEYSQEVWEDSFWKDIMKAFHIEAAPKQTNKSQLSIEYVSEAAETPIAKTYKAFEAEVITNKELHTESSKRSVRHIELRLPETETYQEGDHLGVLPQNSGELISRVIRRFGLDPNQHFKIKGRQLPHLPMDRPVNAPELLASYVELQEPATRAQLRELAAHTVCPPHQKELEHLYSDDAAYKENVLKKRMTMLDLLEDYPACELPFERFLELLPSLKARYYSISSSPKAASGELSITVGVVTAPAWSGRGEYRGVASNYLAGLQKGDSAVCFIRSPQSGFALPENPKTPLIMVGAGTGIAPFRGFIQARAAEKMSGNSLGEAHLYFGCRHPEEDDLYKDEFDHAEKNGLVTVHRAYSRLNQDCKVYVQDVLLREAAQIIALLDQGGHLYICGDGSKMAPAVENVLLQAYEKVHNTDSKVSLEWLEQLQAEGRYAKDVWAGM

>CYP102A49_ortholog(RBAU_2563)Bacillus velezensis UCMB5033

MKQLSAIPQPKTYGPLKNLPHLEKEKLSQSLWKIAEEYGPIFRFEFPSSVGVFVSGRELAAEVCDEKRFDKNLSKALLKVREFGGDGLFTSWTHEKNWQKAHRILLPSFSQKAMKGYHSMMLDIAMQLVQKWSRLNPNEEIDVAEDMTRLTLDTIGLCGFHYRFNSFYRDTQHPFITSMLRALQEAMRQSQRHSLQDKLMIKTRHQFQQDIEEMNSLVDRIIAERRENPDENLSDLLALMLEAKDPVTGERLDDENIRYQIITFLIAGHETTSGLLSFAIYCLLKNKDKLKKAVQEAERVLTGETPEYKQIQQLTYIRMVLNETLRLYPTAPAFSLYAKEDTVLGGKYPIAKGQPVTILTPQLHRDKSAWGEDAESFRPERFSDPAAIPADAYKPFGNGQRACIGMQFALQEATMVLGLVLKHFELIDHTDYELTIKEALTIKPGDFKIRVKNKDVSNHQPVQNHKAEGSGHKEETKEIPSHGTPLLILYGSNLGTAEGIAEELADIGRSKGFSTETGPLDDYAGKLPVKGAVVIVTASYNGAPPDNAAGFVKWMETLEDQELKGVSYAVFGCGDRNWAATYQRIPRLIDKVLEEKGAKRLTSIGEGDNADDFEYSQEAWEDSFWKDIMKAFHIEAAPKQTNKSQLSIEYVSEAAETPIAKTYKAFEAEVITNKELHTESSKRSVRHIELRLPETETYQEGDHLGVLPQNSGELISRVIRRFGLDPNQHFKIKGRQLPHLPMDRPVNAPELLASYVELQEPATRAQLRELAAHTVCPPHQKELEHLYSDDAAYKENVLKKRMTMLDLLEDYPACELPFERFLELLPSLKARYYSISSSPKATSGELSITVGVVTAPAWSGRGEYRGVASNYLAGLQKGDSAVCFIRSPQSGFALPENPKTPLIMVGAGTGIAPFRGFIQARAAEKMSGNSLGEAHLYFGCRHPEEDDLYKDEFDHAEKNGLVTVHRAYSRLDQDCKVYVQDVLLREAAQIIALLDQGGHLYICGDGSKMAPAVENVLLQAYEKVHNTDSKVSLEWLEQLQAEGRYAKDVWAGM

>CYP102A49_ortholog(AJ82_13700)Bacillus velezensis TrigoCor1448

MKQLSAIPQPKTYGPLKNLPHLEKEKLSQSLWKIAEEYGPIFRFEFPSSVGVFVSGRELAAEVCDEKRFDKNLSKALLKVREFGGDGLFTSWTHEKNWQKAHRILLPSFSQKAMKGYHSMMLDIAMQLVQKWSRLNPNEEIDVAEDMTRLTLDTIGLCGFHYRFNSFYRDTQHPFITSMLRALQEAMRQSQRHSLQDKLMIKTRHQFQQDIEEMNSLVDRIIAERRENPDENLSDLLALMLEAKDPVTGERLDDENIRYQIITFLIAGHETTSGLLSFAIYCLLKNKDKLKKAVQEAERVLTGETPEYKQIQQLTYIRMVLNETLRLYPTAPAFSLYAKEDTVLGGKYPIAKGQPVTILTPQLHRDKSAWGEDAELFRPERFSDPAAIPADAYKPFGNGQRACIGMQFALQEATMVLGLVLKHFELIDHTDYELTIKEALTIKPGDFKIRVKNKDVSNHQPVQNHKAEGSGHKEETKEIPSHGTPLLILYGSNLGTAEGIAEELADIGRSKGFSTETGPLDDYAGKLPVKGAVVIVTASYNGAPPDNAAGFVKWMETLEDQELKGVSYAVFGCGDRNWAATYQRIPRLIDKVLEEKGAKRLTSIGEGDNADDFEYSQEAWEDSFWKDIMKAFHIEAAPKQTNKSQLSIEYVSEAAETPIAKTYKAFEAEVITNKELHTESSKRSVRHIELRLPETETYQEGDHLGVLPQNSGELISRVIRRFGLDPNQHFKIKGRQLPHLPMDRPVNAPELLASYVELQEPATRAQLRELAAHTVCPPHQKELEHLYSDDAAYKENVLKKRMTMLDLLEDYPACELPFERFLELLPSLKARYYSISSSPKATSGELSITVGVVTAPAWSGRGEYRGVASNYLAGLQKGDSAVCFIRSPQSGFALPENPKTPLIMVGAGTGIAPFRGFIQARAAEKMSGNSLGEAHLYFGCRHPEEDDLYKDEFDHAEKNGLVTVYRAYSRLDQDCKVYVQDVLLREAAQIIALLDQGGHLYICGDGSKMAPAVENVLLQAYEKVHNTDSKVSLEWLEQLQAEGRYAKDVWAGM

>CYP102A49_ortholog(B938_12535)Bacillus velezensis AS43.3

MKQLSAIPQPKTYGPLKNLPHLEKEKLSQSLWKIAEEYGPIFRFEFPSSVGVFVSGRELAAEVCDEKRFDKNLSKALLKVREFGGDGLFTSWTHEKNWQKAHRILLPSFSQKAMKGYHSMMLDIAMQLVQKWSRLNPNEEIDVAEDMTRLTLDTIGLCGFHYRFNSFYRDTQHPFITSMLRALQEAMRQSQRHSLQDKLMIKTRHQFQQDIEEMNSLVDRIIAERRENPDENLSDLLALMLEAKDPVTGERLDDENIRYQIITFLIAGHETTSGLLSFAIYCLLKNKDKLKKAVQEAERVLTGETPEYKKIQQLTYIRMVLNETLRLYPTAPAFSLYAKEDTVLGGKYPIAKGQPVTILTPQLHRDKSAWGEDAESFRPERFSDPAAIPADAYKPFGNGQRACIGMQFALQEATMVLGLVLKHFELIDHTDYELTIKEALTIKPGDFKIRVKNKDVSNHQPVQNHKAEGSGHKEETKEIPSHGTPLLILYGSNLGTAEGIAEELADIGRSKGFSTETGPLDDYAGKLPVKGAVVIVTASYNGAPPDNAAGFVKWMETLEDQELKGVSYAVFGCGDRNWAATYQRIPRLIDKVLEEKGAKRLTSIGEGDNADDFEYSQEAWEDSFWKDIMKAFHIEAAPKQTNKSQLSIEYVSEAAETPIAKTYKAFEAEVITNKELHTESSKRSVRHIELRLPETETYQEGDHLGVLPQNSGELISRVIRRFGLDPNQHFKIKGRQLPHLPMDRPVNAPELLASYVELQEPATRAQLRELAAHTVCPPHQKELEHLYSDDAAYKENVLKKRMTMLDLLEDYPACELPFERFLELLPSLKARYYSISSSPKATSGELSITVGVVTAPAWSGRGEYRGVASNYLAGLQKGDSAVCFIRSPQSGFALPENPKTPLIMVGAGTGIAPFRGFIQARAAEKMSGNSLGEAHLYFGCRHPEEDDLYKDEFDHAEKNGLVTVHRAYSRLDQDCKVYVQDVLLREAAQIIALLDQGGHLYICGDGSKMAPAVENVLLQAYEKVHNTDSKVSLEWLEQLQAEGRYAKDVWAGM

>CYP102A49_ortholog(U471_25150)Bacillus amyloliquefaciens CC178

MKQLSAIPQPKTYGPLKNLPHLEKEKLSQSLWKIAEEYGPIFRFEFPSSVGVFVSGRELAAEVCDEKRFDKNLSKALLKVREFGGDGLFTSWTHEKNWQKAHRILLPSFSQKAMKGYHSMMLDIAMQLVQKWSRLNPNEEIDVAEDMTRLTLDTIGLCGFHYRFNSFYRDTQHPFITSMLRALQEAMRQSQRHSLQDKLMIKTRHQFQQDIEEMNSLVDRIIAERRENPDENLSDLLALMLEAKDPVTGERLDDENIRYQIITFLIAGHETTSGLLSFAIYCLLKNKDKLKKAVQEAERVLTGETPEYKQIQQLTYIRMVLNETLRLYPTAPAFSLYAKEDTVLGGKYPIAKGQPVTILTPQLHRDKSAWGEDAELFRPERFSDPAAIPADAYKPFGNGQRACIGMQFALQEATMVLGLVLKHFELIDHTDYELTIKEALTIKPGDFKIRVKNKDVSNHQPVQNHKAEGSGHKEETKEIPSHGTPLLILYGSNLGTAEGIAEELADIGRSKGFSTESGPLDDYAGKLPVKGAVVIVTASYNGAPPDNAAGFVKWMETLEDQELKGVSYAVFGCGDRNWAATYQRIPRLIDKVLEEKGAKRLTSIGEGDNADDFEYSQEAWEDSFWKDIMKAFHIEAAPKQTNKSQLSIEYVSEAAETPIAKTYKAFEAEVITNKELHTESSKRSVRHIELRLPETETYQEGDHLGVLPQNSGELISRVIRRFGLDPNQHFKIKGRQLPHLPMDRPVNAPELLASYVELQEPATRAQLRELAAHTVCPPHQKELEHLYSDDAAYKENVLKNRMTMLDLLEDYPACELPFERFLELLPSLKARYYSISSSPKATSGELSITVGVVTAPAWSGRGEYRGVASNYLAGLQKGDSAVCFIRSPQSGFALPENPKTPLIMVGAGTGIAPFRGFIQARAAEKMSGNSLGEAHLYFGCRHPEQDDLYKDEFDHAEKNGLVTVHRAYSRLDQDCKVYVQDVLLREAAQIIALLDQGGHLYICGDGSKMAPAVENVLLQAYEKVHNTDSKVSLNWLEQLQAEGRYAKDVWAGM

>CYP102A49_ortholog(RBAM_024260)Bacillus velezensis FZB42

MKQLSAIPQPKTYGPLKNLPHLEKEKLSQSLWKIAEEYGPIFRFEFPSSVGVFVSGRELAAEVCDEKRFDKNLSKALLKVREFGGDGLFTSWTHEKNWQKAHRILLPSFSQKAMKGYHSMMLDIAMQLVQKWSRLNPNEEIDVAEDMTRLTLDTIGLCGFHYRFNSFYRDTQHPFITSMLRALQEAMRQSQRHSLQDKLMIKTRHQFQQDIEEMNSLVDRIIAERRENPDENLSDLLALMLEAKDPVTGERLDDENIRYQIITFLIAGHETTSGLLSFAIYCLLKNKDKLKKAVQEAERVLTGETPEYKQIQQLTYIRMVLNETLRLYPTAPAFSLYAKEDTVLGGKYPIAKGQPVTILTPQLHRDKSAWGEDAELFRPERFSDPAAIPADAYKPFGNGQRACIGMQFALQEATMVLGLVLKHFELIDHTDYELTIKEALTIKPGDFKIRVKNKDVSNHQPVQNHKAEGSGHKEETKEIPSHGTPLLILYGSNLGTAEGIAEELADIGRSKGFSTESGPLDDYAGKLPVKGAVVIVTASYNGAPPDNAAGFVKWMETLEDQELKGVSYAVFGCGDRNWAATYQRIPRLIDKVLEEKGAKRLTSIGEGDNADDFEYSQEAWEDSFWKDIMKAFHIEAAPKQTNKSQLSIEYVSEAAETPIAKTYKAFEAEVITNKELHTESSKRSVRHIELRLPETETYQEGDHLGVLPQNSGELISRVIRRFGLDPNQHFKIKGRQLPHLPMDRPVNAPELLASYVELQEPATRAQLRELAAHTVCPPHQKELEHLYSDDAAYKENVLKNRMTMLDLLEDYPACELPFERFLELLPSLKARYYSISSSPKATSGELSITVGVVTAPAWSGRGEYRGVASNYLAGLQKGDSAVCFIRSPQSGFALPENPKTPLIMVGAGTGIAPFRGFIQARAAEKMSGNSLGEAHLYFGCRHPEQDDLYKDEFDHAEKNGLVTVHRAYSRLDQDCKVYVQDVLLREAAQIIALLDQGGHLYICGDGSKMAPAVENVLLQAYEKVHNTDSKVSLNWLEQLQAEGRYAKDVWAGM

>CYP102A49_ortholog(NG74_02548)Bacillus velezensis

MKQLSAIPQPKTYGPLKNLPHLEKEKLSQSLWKIAEEYGPIFRFEFPSSVGVFVSGRELAAEVCDEKRFDKNLSKALLKVREFGGDGLFTSWTHEKNWQKAHRILLPSFSQKAMKGYHSMMLDIAMQLVQKWSRLNPNEEIDVAEDMTRLTLDTIGLCGFHYRFNSFYRDTQHPFITSMLRALQEAMRQSQRHSLQDKLMIKTRHQFQQDIEEMNSLVDRIIAERRENPDENLSDLLALMLEAKDPVTGERLDDENIRYQIITFLIAGHETTSGLLSFAIYCLLKNKDKLKKAVQEAERVLTGETPEYKQIQQLTYIRMVLNETLRLYPTAPAFSLYAKEDTVLGGKYPIEKGQPVTILTPQLHRDKSAWGEDAESFRPERFSDPAAIPADAYKPFGNGQRACIGMQFALQEATMVLGLVLKHFELIDHTDYELTIKEALTIKPGDFKIRVKNKDVSNHQPVQNHKAEGSGHKEETKEIPSHGTPLLILYGSNLGTAEGIAEELADIGRSKGFSTETGPLDDYAGKLPVKGAVVIVTASYNGAPPDNAAGFVKWMETLEDQELKGVSYAVFGCGDRNWAATYQRIPRLIDKVLEEKGAKRLTSIGEGDNADDFEYSQEAWEDSFWKDIMKTFHIEAAPKQTNKSQLSIEYVSEAAETPIAKTYKAFEAEVITNKELHTESSKRSVRHIELRLPETETYQEGDHLGVLPQNSGELISRVIRRFGLDPNQHFKIKGRQLPHLPMDRPVNAPELLASYVELQEPATRAQLRELAAHTVCPPHQKELEHLYSDDAAYKENVLKKRMTMLDLLEDYPACELPFERFLELLPSLKARYYSISSSPKAASGELSITVGVVTAPAWSGRGEYRGVASNYLAGLQNGDPAVCFIRSPQSGFALPENPKTPLIMVGAGTGIAPFRGFIQARAAEKMSGNSLGEAHLYFGCRHPEEDDLYKDEFDDAEKNGLVTVHRAYSRLDQDCKVYVQDVLLREAAQIIALLDQGGHLYICGDGSKMAPAVENVLLQAYEKVHNTDSKVSLNWLEQLQAEGRYAKDVWAGM

>CYP102A49_ortholog(BAPNAU_1137)Bacillus velezensis NAU-B3

MKQLSAIPQPKTYGPLKNLPHLEKEKLSQSLWKIAEEYGPIFRFEFPSSVGVFVSGRELAAEVCDEKRFDKNLSKALLKVREFGGDGLFTSWTHEKNWQKAHRILLPSFSQKAMKGYHSMMLDIAMQLVQKWSRLNPNEEIDVAEDMTRLTLDTIGLCGFHYRFNSFYRDTQHPFITSMLRALQEAMRQSQRHSLQDKLMIKTRHQFQQDIEEMNSLVDRIIAERRENPDENLSDLLALMLEAKDPVTGERLDDENIRYQIITFLIAGHETTSGLLSFAIYCLLKNKDKLKKAVQEAERVLTGETPEYKQIQQLTYIRMVLNETLRLYPTAPAFSLYAKEDTVLGGKYPIEKGQPVTILTPQLHRDKSAWGEDAESFRPERFSDPAAIPADAYKPFGNGQRACIGMQFALQEATMVLGLVLKHFELIDHTDYELTIKEALTIKPGDFKIRVKNKDVSNHQPVQNHKAEGSGHKEETKEIPSHGTPLLILYGSNLGTAEGIAEELADIGRSKGFSTETGPLDDYAGKLPVKGAVVIVTASYNGAPPDNAAGFVKWMETLEDQELKGVSYAVFGCGDRNWAATYQRIPRLIDKVLEEKGAKRLTSIGEGDNADDFEYSQEAWEDSFWKDIMKTFHIEAAPKQTNKSQLSIEYVSEAAETPIAKTYKAFEAEVITNKELHTESSKRSVRHIELRLPETETYQEGDHLGVLPQNSGELISRVIRRFGLDPNQHFKIKGRQLPHLPMDRPVNAPELLASYVELQEPATRAQLRELAAHTVCPPHQKELEHLYSDDAAYKENVLKKRMTMLDLLEDYPACELPFERFLELLPSLKARYYSISSSPKAASGELSITVGVVTAPAWSGRGEYRGVASNYLAGLQNGDPAVCFIRSPQSGFALPENPKTPLIMVGAGTGIAPFRGFIQARAAEKMSGNSLGEAHLYFGCRHPEEDDLYKDEFDDAEKNGLVTVHRAYSRLDQDCKVYVQDVLLREAAQIIALLDQGGHLYICGDGSKMAPAVENVLLQAYEKVHNTDSKVSLNWLEQLQAEGRYAKDVWAGM

>CYP102A49_ortholog(V529_27090)Bacillus velezensis SQR9

MKQLSAIPQPKTYGPLKNLPHLEKEKLSQSLWKIAEEYGPIFRFEFPSSVGVFVSGRELAAEVCDEKRFDKNLSKALLKVREFGGDGLFTSWTHEKNWQKAHRILLPSFSQKAMKGYHSMMLDIAMQLVQKWSRLNPNEEIDVAEDMTRLTLDTIGLCGFHYRFNSFYRDTQHPFITSMLRALQEAMRQSQRHSLQDKLMIKTRHQFQQDIEEMNSLVDRIIAERRENPDENLSDLLALMLEAKDPVTGERLDDENIRYQIITFLIAGHETTSGLLSFAIYCLLKNKDKLKKAVQEAERVLTGETPEYKQIQQLTYIRMVLNETLRLYPTAPAFSLYAKEDTVLGGKYPIAKGQPVTILTPQLHRDKSAWGEDAESFRPERFSDPAAIPADAYKPFGNGQRACIGMQFALQEATMVLGLVLKHFELIDHTDYELTIKEALTIKPGDFKIRVKNKDVSNHQPVQNHKAEGSGHKEETKEIPSHGTPLLILYGSNLGTAEGIAEELAGIGRSKGFSTETGPLDDYAGKLPVKGAVVIVTASYNGAPPDNAAGFVKWMETLEDQELKGVSYAVFGCGDRNWAATYQRVPRLIDKVLEEKGAKRLTSIGEGDNADDFEYSQEAWEDSFWKDIMKAFHIEAAPKQTNKSQLSIEYVSEAAETPIAKTYKAFEAEVITNKELHTESSKRSVRHIELRLPETETYQEGDHLGVLPQNSGELISRVIRRFGLDPNQHFKIKGRQLPHLPMDRPVNAPELLASYVELQEPATRVQLRELAAHTVCPPHQKELEHLYSDDTAYKENVLKKRMTMLDLLENYPACELPFERFLELLPSLKARYYSISSSPKAASGELSITVGVVTAPAWSGRGEYRGVASNYLAGLQNGDSAVCFIRSPQSGFALPENPKTPLIMVGAGTGIAPFRGFIQARAAEKMSGNILGAAHLYFGCRHPEEDDLYKDEFDHAEKNGLVTVHRAYSRLDQDCKVYVQDVLLREAEQIIALLDQGGHLYICGDGSKMAPAVENVLLQAYEKVHNTDSKVSLEWLEQLQAEGRYAKDVWAGM

>CYP102A49_ortholog(OY17_15415)Bacillus sp. BH072

MKQLSAIPQPKTYGPLKNLPHLEKEKLSQSLWKIAEEYGPIFRFEFPSSVGVFVSGRELAAEVCDEKRFDKNLSKALLKVREFGGDGLFTSWTHEKNWQKAHRILLPSFSQKAMKGYHSMMLDIAMQLVQKWSRLNPNEEIDVAEDMTRLTLDTIGLCGFHYRFNSFYRDTQHSFITSMLRALQEAMRQAQRHSLQDKLMIKTRHQFQQDIEEMNSLVDRIIAERRENPDENLSDLLALMLEAKDPVTGERLDDENIRYQIITFLIAGHETTSGLLSFAIYCLLKNKDKLKKAVQEAERVLTGETPEYKQIQQLTYIRMVLNETLRLYPTAPAFSLYAKEDTVLGGKYPIAKGQPVTILTPQLHRDKSAWGEDAESFRPERFSDPAAIPADAYKPFGNGQRACIGMQFALQEATMVLGLVLKHFELIDHTDYELTIKEALTIKPGDFKIRVKNKDVSNQQSAQNHKAEGSGHKEEMKEIPSHGTPLLILYGSNLGTAEGIAEELADIGRSKGFSTETGPLDDYAGKLPVKGAVVIVTASYNGAPPDNAAGFVKWMETLEDQELKGVSYAVFGCGDRNWAATYQRIPRLIDKVLEEKGAKRLTSIGEGDNADDFEYSQEAWEDSFWKDIMKAFHIEAAPKQTNKSQLSIEYVSEAAETPIAKTYKAFEAEVITNKELHTESSKRSVRHIELRLPETETYQEGDHLGVLPQNSGELISRVIRRFGLDPNQHFKIKGRQLPHLPMDRPVNAPELLASYVELQEPATRAQLRELAAHTVCPPHQKELEHLYSDDAAYKENVLKKRMTMLDLLEDYPACELPFERFLELLPSLKARYYSISSSPKAASGELSITVGVVTAPAWSGRGEYRGVASNYLAGLQNGDPAVCFIRSPQSGFALPENPKTPLIMVGAGTGIAPFRGFIQARAAEKMSGNSLGEAHLYFGCRHPEEDDLYKDEFDDAEKNGLVTVHRAYSRLDQDCKVYVQDVLLREAAQIIALLDQGGHLYICGDGSKMAPAVENVLLQAYEKVHNTDSKVSLNWLEQLQAEGRYAKDVWAGM

>CYP102A49_ortholog(MUS_2901)Bacillus amyloliquefaciens Y2

MKQLSAIPQPKTYGPLKNLPHLEKEKLSQSLWKIAEEYGPIFRFEFPSSVGVFVSGRELAAEVCDEKRFDKNLSKALLKVREFGGDGLFTSWTHEKNWQKAHRILLPSFSQKAMKGYHSMMLDIAMQLVQKWSRLNPNEEIDVAEDMTRLTLDTIGLCGFHYRFNSFYRDTQHSFITSMLRALQEAMRQAQRHSLQDKLMIKTRHQFQQDIEEMNSLVDRIIAERRENPDENLSDLLALMLEAKDPVTGERLDDENIRYQIITFLIAGHETTSGLLSFAIYCLLKNKDKLKKAVQEAERVLTGETPEYKQIQQLTYIRMVLNETLRLYPTAPAFSLYAKEDTVLGGKYPIAKGQPVTILTPQLHRDKSAWGEDAESFRPERFSDPAAIPADAYKPFGNGQRACIGMQFALQEATMVLGLVLKHFELIDHTDYELTIKEALTIKPGDFKIRVKNKDVSNQQSAQNHKAEGSGHKEEMKEIPSHGTPLLILYGSNLGTAEGIAEELADIGRSKGFSTETGPLDDYAGKLPVKGAVVIVTASYNGAPPDNAAGFVKWMETLEDQELKGVSYAVFGCGDRNWAATYQRIPRLIDKVLEEKGAKRLTSIGEGDNADDFEYSQEAWEDSFWKDIMKAFHIEAAPKQTNKSQLSIEYVSEAAETPIAKTYKAFEAEVITNKELHTESSKRSVRHIELRLPETETYQEGDHLGVLPQNSGELISRVIRRFGLDPNQHFKIKGRQLPHLPMDRPVNAPELLASYVELQEPATRAQLRELAAHTVCPPHQKELEHLYSDDAAYKENVLKKRMTMLDLLEDYPACELPFERFLELLPSLKARYYSISSSPKAASGELSITVGVVTAPAWSGRGEYRGVASNYLAGLQNGDPAVCFIRSPQSGFALPENPKTPLIMVGAGTGIAPFRGFIQARAAEKMSGNSLGEAHLYFGCRHPEEDDLYKDEFDDAEKNGLVTVHRAYSRLDQDCKVYVQDVLLREAAQIIALLDQGGHLYICGDGSKMAPAVENVLLQAYEKVHNTDSKVSLNWLEQLQAEGRYAKDVWAGM

>CYP102A49_ortholog(BANAU_2583)Bacillus velezensis YAU B9601-Y2

MKQLSAIPQPKTYGPLKNLPHLEKEKLSQSLWKIAEEYGPIFRFEFPSSVGVFVSGRELAAEVCDEKRFDKNLSKALLKVREFGGDGLFTSWTHEKNWQKAHRILLPSFSQKAMKGYHSMMLDIAMQLVQKWSRLNPNEEIDVAEDMTRLTLDTIGLCGFHYRFNSFYRDTQHSFITSMLRALQEAMRQAQRHSLQDKLMIKTRHQFQQDIEEMNSLVDRIIAERRENPDENLSDLLALMLEAKDPVTGERLDDENIRYQIITFLIAGHETTSGLLSFAIYCLLKNKDKLKKAVQEAERVLTGETPEYKQIQQLTYIRMVLNETLRLYPTAPAFSLYAKEDTVLGGKYPIAKGQPVTILTPQLHRDKSAWGEDAESFRPERFSDPAAIPADAYKPFGNGQRACIGMQFALQEATMVLGLVLKHFELIDHTDYELTIKEALTIKPGDFKIRVKNKDVSNQQSAQNHKAEGSGHKEEMKEIPSHGTPLLILYGSNLGTAEGIAEELADIGRSKGFSTETGPLDDYAGKLPVKGAVVIVTASYNGAPPDNAAGFVKWMETLEDQELKGVSYAVFGCGDRNWAATYQRIPRLIDKVLEEKGAKRLTSIGEGDNADDFEYSQEAWEDSFWKDIMKAFHIEAAPKQTNKSQLSIEYVSEAAETPIAKTYKAFEAEVITNKELHTESSKRSVRHIELRLPETETYQEGDHLGVLPQNSGELISRVIRRFGLDPNQHFKIKGRQLPHLPMDRPVNAPELLASYVELQEPATRAQLRELAAHTVCPPHQKELEHLYSDDAAYKENVLKKRMTMLDLLEDYPACELPFERFLELLPSLKARYYSISSSPKAASGELSITVGVVTAPAWSGRGEYRGVASNYLAGLQNGDPAVCFIRSPQSGFALPENPKTPLIMVGAGTGIAPFRGFIQARAAEKMSGNSLGEAHLYFGCRHPEEDDLYKDEFDDAEKNGLVTVHRAYSRLDQDCKVYVQDVLLREAAQIIALLDQGGHLYICGDGSKMAPAVENVLLQAYEKVHNTDSKVSLNWLEQLQAEGRYAKDVWAGM

>CYP102A49_ortholog(KSO_007250)Bacillus amyloliquefaciens IT-45

MKQLSAIPQPKTYGPLKNLPHLEKEKLSQSLWKIAEEYGPIFRFEFPSSVGVFVSGRELAAEVCDEKRFDKNLSKALLKVREFGGDGLFTSWTHEKNWQKAHRILLPSFSQKAMKGYHSMMLDIAMQLVQKWSRLNPNEEIDVAEDMTRLTLDTIGLCGFHYRFNSFYRDTQHPFITSMLRALQEAMRQSQRHSLQDKLMIKTRHQFQQDIEVMNELVDRIIAERRENPDENLSDLLALMLEAKDPVTGERLDDENIRYQIITFLIAGHETTSGLLSFAIYCLLKNKDKLKKAVQEAERVLTGETPEYKQIQQLIYIRMVLNETLRLYPTAPAFSLYAKEDTVLGGKYPIAKGQPVTILTPQLHRDKSAWGEDAELFRPERFSDPAAIPADAYKPFGNGQRACIGMQFALQEATMVLGLVLKHFELIDHTDYELTIKEALTIKPGDFKIRVKNKDVSNHQPVQNHKAEGGGHKEETKEIPSHGTPLLILYGSNLGTAEGIAEELADIGRSKGFSTETGPLDDYAGKLPVKGAVVIVTASYNGAPPDNAAGFVKWMETLEDQELKGVSYAVFGCGDRNWAATYQRIPRLIDKVLEEKGAKRLTSIGEGDNADDFEYSQEAWEDSFWKDIMKAFHIEAAPKQANKSQLSIEYVSEAAETPIAKTYKAFEAEVITNKELHTESSKRSVRHIELRLPKTETYQEGDHLGVLPQNSGELISRVIRRFGLDPNQHFKIKGRQLPHLPMDRPVNAPELLASYVELQEPATRAQLRELAAHTVCPPHQKELEHLYSDDAAYKENVLKKRMTMLDLLEDYPACELPFERFLELLPSLKARYYSISSSPKANKRELSITVGVVTAPAWSGRGEYRGVASNYLAGLQNGDSAVCFIRSPQSGFALPENTKTPLIMIGAGTGIAPFRGFIQARAAEKMSGNSLGEAHLYFGCRHPEEDDLYKDEFDHAEKNGLVTVHRAYSRLDQDCKVYVQDVLLREAAQIIALLDQGGHLYICGDGSKMAPAVENVLLQAYEKVHNTDSKVSSNWLEQLQAEGRYAKDVWAGV

>CYP102A49_ortholog(BASU_2369)Bacillus velezensis UCMB5113

MKQLSAIPQPKTYGPLKNLPHLEKEKLSQSLWKIAEEYGPIFRFEFPSSVGVFVSGRELAAEVCDEKRFDKNLSKALLKVREFGGDGLFTSWTHEKNWQKAHRILLPSFSQKAMKGYHSMMLDIAMQLVQKWSRLNPNEEIDVAEDMTRLTLDTIGLCGFHYRFNSFYRDTQHPFITSMLRALQEAMRQSQRHSLQDKLMIKTRHQFQQDIEEMNSLVDRIIAERRENPDENLSDLLALMLEAKDPVTGERLDDENIRYQIITFLIAGHETTSGLLSFAIYCLLKNKDKLKKAVQEAERVLTGETPEYKQIQQLTYIRMVLNETLRLYPTAPAFSLYAKEDTVLGGKYPIAKGQPVTILTPQLHRDKSAWGEDAELFRPERFSDPAAIPADAYKPFGNGQRACIGMQFALQEATMVLGLVLKHFELIDHTDYELTIKEALTIKPGDFKIRVKNKDVSNHQPVQNHKAEGSGHKEETKEIPSHGTPLLILYGSNLGTAEGIAEELADIGRSKGFSTETGPLDDYAGKLPVKGAVVIVTASYNGAPPDNAAGFVKWMETLEDQELKGVSYAVFGCGDRNWAATYQRIPRLIDKVLEEKGAKRLTSIGEGDNADDFEYSQEAWEDSFWKDIMKAFHIDIEAAPKQANKSQLSIEYVSEAAETPIAKTYKAFEAEVITNKELHTESSKRSVRHIELRLPETETYQEGDHLGVLPQNSGELISRVIRRFGLDPNQHFKIKGRQLPHLPMDRPVNAPELLASYVELQEPATRAQLRELAAHTVCPPHQKELEHLYSDDAAYKENVLKKRMTMLDLLEDYPACELPFERFLELLPSLKARYYSISSSPKATSGELSITVGVVTAPAWSGRGEYRGVASNYLAGLQKGDSAVCFIRSPQSGFALPENPKTPLIMVGAGTGIAPFRGFIQARAAEKMSGNSLGEAHLYFGCRHPEEDDLYKDEFDHAEKNGLVTVHRAYSRLDQDCKVYVQDVLLREAAQIIALLDQGGHLYICGDGSKMAPAVENVLLQAYEKVHNTDSKVSLEWLEQLQAEGRYAKDVWAGM

>CYP102A49_ortholog(BACAU_2438)Bacillus velezensis CAU B946

MKQLSAIPQPKTYGPLKNLPHLEKEKLSQSLWKIAEEYGPIFRFEFPSSVGVFVSGRELAAEVCDEKRFDKNLSKALLKVREFGGDGLFTSWTHEKNWQKAHRILLPSFSQKAMKGYHSMMLDIAMQLVQKWSRLNPNEEIDVAEDMTRLTLDTIGLCGFHYRFNSFYRDTQHPFITSMLRALQEAMRQSQRHSLQDKLMIKTRHQFQQDIEVMNELVDRIIAERRENPDENLSDLLALMLEAKDPVTGERLDDENIRYQIITFLIAGHETTSGLLSFAIYCLLKNKDKLKKAVQEAERVLTGETPEYKQIQQLTYIRMVLNETLRLYPTAPAFSLYAKEDTVLGGKYPIAKGQPVTILTPQLHRDKSAWGEDAELFRPERFSDPAAIPADAYKPFGNGQRACIGMQFALQEATMVLGLVLKHFELIDHTDYELTIKEALTIKPGDFKIRVKNKDVSNHQPVQNHKAEGGGHKEETKEIPSHGTPLLILYGSNLGTAEGIAEELADIGRSKGFSTETGPLNDYAGKLPVKGAVVIVTASYNGAPPDNAAGFVKWMETLEDQELKGVSYAVFGCGDRNWAATYQRIPRLIDKVLEEKGAKRLTSIGEGDNADDFEYSQEAWEDSFWKDIMKAFHIEAAPKQANKSQLSIEYVSEAAETPIAKTYKAFEAEVITNKELHTESSKRSVRHIELRLPKTETYREGDHLGVLPQNSGELISRVIRRFGLDPNQHFKIKGRQLPHLPMDRPVNAPELLASYVELQEPATRAQLRELAAHTVCPPHQKELEHLYSDDAAYKENVLKKRMTMLDLLEDYPACELPFERFLELLPSLKARYYSISSSPKANKRELSITVGVVTAPAWSGRGEYKGVASNYLAGLQNGDSAVCFIRSPQSGFALPENTKTPLIMIGAGTGIAPFRGFIQARAAEKMSGNSLGEAHLYFGCRHPEEDDLYKDEFDHAEKNGLVTVHRAYSRLDQDCKVYVQDVLLREAAQIIALLDQGGHLYICGDGSKMAPAVENVLLQAYEKVHNTDSKVSSNWLEQLQAEGRYAKDVWAGV

>CYP102A49_ortholog(U722_13165)Bacillus amyloliquefaciens LFB112

MKQLSAIPQPKTYGPLKNLPHLEKEKLSQSLWKIAEEYGPIFRFEFPSSVGVFVSGRELAAEVCDEKRFDKNLSKALLKVREFGGDGLFTSWTHEKNWQKAHRILLPSFSQKAMKGYHSMMLDIAMQLVQKWSRLNPNEEIDVAEDMTRLTLDTIGLCGFHYRFNSFYRDTQHPFITSMLRALQEAMRQSQRHSLQDKLMIKTRHQFQQDIEVMNELVDRIIAERRENPDENLSDLLALMLEAKDPVTGERLDDENIRYQIITFLIAGHETTSGLLSFAIYCLLKNKDKLKKAVQEAERVLTGETPEYKQIQQLTYIRMVLNETLRLYPTAPAFSLYAKEDTVLGGKYPIAKGQPVTILTPQLHRDKSAWGEDAELFRPERFSDPAAIPADAYKPFGNGQRACIGMQFALQEATMVLGLVLKHFELIDHTDYELTIKEALTIKPGDFKIRVKNKDVSNHQPVQNHKAEGGGHKEETKEIPSHGTPLLILYGSNLGTAEGIAEELADIGRSKGFSTETGPLDDYAGKLPVKGAVVIVTASYNGAPPDNAVGFVKWTETLEDQELKGVSYAVFGCGDRNWAATYQRIPRLIDKVLEEKGAKRLTSIGEGDNADDFEYSQEAWEDSFWKDIMKAFHIEAAPKQANKSQLSIEYVSEAAETPIAKTYKAFEAEVITNKELHTESSKRSVRHIELRLPKTETYREGDHLGVLPQNSGELISRVIRRFGLDPNQHFKIKGRQLPHLPMDRPVNAPELLASYVELQEPATRAQLRELAAHTVCPPHQKELEHLYSDDAAYKENVLKKRMTMLDLLEDYPACELPFERFLELLPSLKARYYSISSSPKANKRELSITVGVVTAPAWSGRGEYKGVASNYLAGLQNGDSAVCFIRSPQSGFALPENTKTPLIMIGAGTGIAPFRGFIQARAAEKMSGNSLGEAHLYFGCRHPEEDDLYKDEFDHAEKNGLVTVHRAYSRLDQDCKVYVQDVLLREAAQIIALLDQGGHLYICGDGSKMAPAVENVLLQAYEKVHNTDSKVSSNWLEQLQAEGRYAKDVWAGV

>CYP102A49_ortholog(BAMF_2522)Bacillus amyloliquefaciens DSM 7

MKQLSAIPQPKTYGPLKNLPHLEKEKLSQSLWKIAEEYGPIFRFEFPSSVGVFVSGRELAAEVCDEKRFDKNLSKALLKVREFGGDGLFTSWTHEKNWQKAHRILLPSFSQKAMKGYHSMMLDIAMQLVQKWSRLNPNEEIDVAEDMTRLTLDTIGLCGFHYRFNSFYRDTQHPFITSMLRALQEAMRQSQRHSLQDKLMIKTRHQFQQDIEVMNELVDRIIAERRENPDENLSDLLALMLEAKDPVTGERLDDENIRYQIITFLIAGHETTSGLLSFAIYCLLKNKDKLKKAIQEAERVLTGETPEYKQIQQLTYIRMVLNETLRLYPTAPAFSLYAKEDTVLGGKYPIAKGQPVTILTPQLHRDKSAWGEDAELFRPERFSDPAAIPADAYKPFGNGQRACIGMQFALQEATMVLGLVLKHFELIDHTDYELTIKEALTIKPGDFKIRVKNKDVSNQQSAQVHKAEGGGHKEETKEIPSHGTPLLILYGSNLGTAEGIAEELADIGRSKGFSSETGPLDDYAGKLPVKGAVVIVTASYNGAPPDNAAGFVKWMETLEDQELKGVSYAVFGCGDRNWAATYQRIPRLIDKVLEEKGAKRLTSIGEGDNADDFEYSQEAWEDSFWKDIMKAFHIEAAPKQTNKSQLSIEYVSEAAETPIAKTYKAFEAEVTTNKELHTESSKRSVRHIELRLPETETYQEGDHLGVLPQNSGELISRVIRRFGLDPNQHFKIKGRQLPHLPMDRPVNAPELLASYVELQEPATRAQLRELAAHTVCPPHQKELEHLYSDYAAYKENVLKKRMTMLDLLEDYPACELPFERFLELLPSLKARYYSISSSPKANKRELSITVGVVTAPAWSGRGEYRGVASNYLAGLQNGDSAVCFIRSPQSGFALPENTKTPLIMIGAGTGIAPFRGFIQARAAEKMSGNSLGEAHLYFGCRHPEEDDLYKDEFDHAEKNGLVTVHRAYSRLDQDCKVYVQDVLLREAAQIIALLDQGGHLYICGDGSKMAPAVENVLLQAYEKVHNTDSKVSLNWLEQLQAEGRYAKDVWAGV

>CYP102A49_ortholog(BAXH7_02724)Bacillus amyloliquefaciens XH7

MKQLSAIPQPKTYGPLKNLPHLEKEKLSQSLWKIAEEYGPIFRFEFPSSVGVFVSGRELAAEVCDEKRFDKNLGKALLKVREFGGDGLFTSWTNEKNWQKAHRILLPSFSQKAMKGYHSMMLDIAMQLVQKWSRLNPNEEIDVAEDMTRLTLDTIGLCGFHYRFNSFYRDTQHPFITSMLRALQEAMRQSQRHSLQDKLMIKTRHQFQQDIEEMNALVDRIIAERRENPDENLSDLLALMLEAKDPVTGERLDDENIRYQIITFLIAGHETTSGLLSFAIYCLLKNKDKLEKAVQEAERVLTGETPEYKQIQQLTYIRMVLNETLRLYPTAPAFSLYAKEDTVLGGKYPIEKGQPVTILTPQLHRDKSAWGEDAELFRPERFSDPAAIPADAYKPFGNGQRACIGMQFALQEATMVLGLVLKHFELIDHSDYELTIKEALTIKPGDFKIRVKNKDVSNRQTAQNHKAEGSDHKEETKEIPSHGTPLLILYGSNLGTAEGIAEELADIGRSKGFSTETGPLDDYAGKLPVKGAVVIVTASYNGAPPDNAAGFVKWMETLEDQELKGVSYAVFGCGDRNWAATYQKIPRLIDKVLEEKGATRLTSIGEGDNADDFEYSQEAWEDSFWQDIIKAFHIEAAPEQQNKSQLSIEYVSEAAETPIAKTYKAFEAEVITNKELHTESSTRSVRHIELRLPETETYQEGDHLGVLPQNSGELISRVIHRFALDPNQHFKISGRHLPHLPMDRPVNALELLASYVELQEPATRAQLRELAAHTVCPPHQKELEHLYSDETAYKENVLKKRMTMLDLLEDYPACELPFERFLELLPSLKARYYSISSSPKANSGELSITVGVVTAPAWSGRGEYRGVASNYLAGLQNGDHAVCFIRSPQSGFALPENTKTPLIMVGAGTGIAPFRGFIQARAAEKMSGNSLGEAHLYFGCRHPEEDDLYKDEFDQAEKTGLVTVHRAYSRLDQDCKVYVQDVLLREAAQIIALLDQGGHLYICGDGSKMAPAVENVLLQAYEKVHNTDSKVSLSWLEQLQAEGRYAKDVWAGV

>CYP102A49_ortholog(LL3_02800)Bacillus amyloliquefaciens LL3

MKQLSAIPQPKTYGPLKNLPHLEKEKLSQSLWKIAEEYGPIFRFEFPSSVGVFVSGRELAAEVCDEKRFDKNLGKALLKVREFGGDGLFTSWTNEKNWQKAHRILLPSFSQKAMKGYHSMMLDIAMQLVQKWSRLNPNEEIDVAEDMTRLTLDTIGLCGFHYRFNSFYRDTQHPFITSMLRALQEAMRQSQRHSLQDKLMIKTRHQFQQDIEEMNALVDRIIAERRENPDENLSDLLALMLEAKDPVTGERLDDENIRYQIITFLIAGHETTSGLLSFAIYCLLKNKDKLEKAVQEAERVLTGETPEYKQIQQLTYIRMVLNETLRLYPTAPAFSLYAKEDTVLGGKYPIEKGQPVTILTPQLHRDKSAWGEDAELFRPERFSDPAAIPADAYKPFGNGQRACIGMQFALQEATMVLGLVLKHFELIDHSDYELTIKEALTIKPGDFKIRVKNKDVSNRQTAQNHKAEGSDHKEETKEIPSHGTPLLILYGSNLGTAEGIAEELADIGRSKGFSTETGPLDDYAGKLPVKGAVVIVTASYNGAPPDNAAGFVKWMETLEDQELKGVSYAVFGCGDRNWAATYQKIPRLIDKVLEEKGATRLTSIGEGDNADDFEYSQEAWEDSFWQDIIKAFHIEAAPEQQNKSQLSIEYVSEAAETPIAKTYKAFEAEVITNKELHTESSTRSVRHIELRLPETETYQEGDHLGVLPQNSGELISRVIHRFALDPNQHFKISGRHLPHLPMDRPVNALELLASYVELQEPATRAQLRELAAHTVCPPHQKELEHLYSDETAYKENVLKKRMTMLDLLEDYPACELPFERFLELLPSLKARYYSISSSPKANSGELSITVGVVTAPAWSGRGEYRGVASNYLAGLQNGDHAVCFIRSPQSGFALPENTKTPLIMVGAGTGIAPFRGFIQARAAEKMSGNSLGEAHLYFGCRHPEEDDLYKDEFDQAEKTGLVTVHRAYSRLDQDCKVYVQDVLLREAAQIIALLDQGGHLYICGDGSKMAPAVENVLLQAYEKVHNTDSKVSLSWLEQLQAEGRYAKDVWAGV

>CYP102A49_ortholog(BAMTA208_13325)Bacillus amyloliquefaciens TA208

MKQLSAIPQPKTYGPLKNLPHLEKEKLSQSLWKIAEEYGPIFRFEFPSSVGVFVSGRELAAEVCDEKRFDKNLGKALLKVREFGGDGLFTSWTNEKNWQKAHRILLPSFSQKAMKGYHSMMLDIAMQLVQKWSRLNPNEEIDVAEDMTRLTLDTIGLCGFHYRFNSFYRDTQHPFITSMLRALQEAMRQSQRHSLQDKLMIKTRHQFQQDIEEMNALVDRIIAERRENPDENLSDLLALMLEAKDPVTGERLDDENIRYQIITFLIAGHETTSGLLSFAIYCLLKNKDKLEKAVQEAERVLTGETPEYKQIQQLTYIRMVLNETLRLYPTAPAFSLYAKEDTVLGGKYPIEKGQPVTILTPQLHRDKSAWGEDAELFRPERFSDPAAIPADAYKPFGNGQRACIGMQFALQEATMVLGLVLKHFELIDHSDYELTIKEALTIKPGDFKIRVKNKDVSNRQTAQNHKAEGSDHKEETKEIPSHGTPLLILYGSNLGTAEGIAEELADIGRSKGFSTETGPLDDYAGKLPVKGAVVIVTASYNGAPPDNAAGFVKWMETLEDQELKGVSYAVFGCGDRNWAATYQKIPRLIDKVLEEKGATRLTSIGEGDNADDFEYSQEAWEDSFWQDIIKAFHIEAAPEQQNKSQLSIEYVSEAAETPIAKTYKAFEAEVITNKELHTESSTRSVRHIELRLPETETYQEGDHLGVLPQNSGELISRVIHRFALDPNQHFKISGRHLPHLPMDRPVNALELLASYVELQEPATRAQLRELAAHTVCPPHQKELEHLYSDETAYKENVLKKRMTMLDLLEDYPACELPFERFLELLPSLKARYYSISSSPKANSGELSITVGVVTAPAWSGRGEYRGVASNYLAGLQNGDHAVCFIRSPQSGFALPENTKTPLIMVGAGTGIAPFRGFIQARAAEKMSGNSLGEAHLYFGCRHPEEDDLYKDEFDQAEKTGLVTVHRAYSRLDQDCKVYVQDVLLREAAQIIALLDQGGHLYICGDGSKMAPAVENVLLQAYEKVHNTDSKVSLSWLEQLQAEGRYAKDVWAGV

>CYP102A50(BRLA_c023790)Brevibacillus laterosporus

MKEVGYIPQPKTYGPLGNYPLIEKDTPTLSLCKLAEEFGPIYRLTLSGSSSLVISGHKLAAEVCDESRFFKNITGDLENVRAFSGDGLFTSRIEEPNWQKAHNILLPTFSQQAMKGYHSMMVDIALQLIQKWARLNPNESIDVPGDMTRLTLDTIGLCGFNYRFNSFYRETHSPFINSMVRALQEAMHQSGRLKIQNTLMVGTRRQFTQDIETMFSLVDKIIEERKVNGDQGEIDLLTRMLNGKDPQTGERLDDENIRYQIITFLIAGHETTSGLLSFALYFLLKNPEVREKAYQEVDRVLTGVSPSYQQVLQLTYVRMILQESLRLWPTAPGFELTARADTIIGGKYAIKKGEVVALLLPQLHRDKDAWGDDADEFCPERFEDPDKVPHHAYKPFGNGQRACIGMQFALHEATLVLGMILQNFELIDHTNYQLSIQQTLTIKPEEFYIRVKQRKKQKLDQSTQEPEVGAVNIRGTDSRWEQIKTSIARVNHVSLLILYGSNLGTAERTARELSTNAGLLGIRSEMAPLDDWLGKLPKEGVVLIVTASYNGKPPNNARKFVQWLEGVEAGALQGVTYAVFGCGDRNWANTYQDVPRFIDEQLTLKGAKRLSLRGEADTSGDFEKQLEDWQKRMWLDVMETFILQENLEEKDHGTLTIQFMNDMSDVPLALKYNAVYAYITENRELESANCGRSIRQVEITLPDGLSYQEGDQIGVLPKNRKEHVERILQRFGLNGNDPLILRADGRNIAHLPLDRPVRLNDLLSQNVDLQATATHAQLHELAAYTVCPPHKRELEDLLDEVVYEQQVEKTGVSMLELLEKYEACEMPFERVIELLPPLQPKYYTITSSPENSPHQASIMIGADSLESECGVHLGMLDHYLADCRPEEKIIMFISTPES

>CYP102A51(BMWSH_1945)Bacillus megaterium WSH-002

MTIKEMPQPKTFGELKNLPLLNTDKPIQTLMKIADELGEIFKFEAPGRVTRYLSSQRLIKEACDESRFDKNLSQALKFVRDFAGDGLFTSWTHEKNWKKAHNILLPSFSQQAMKGYHAMMVDIAVQLIQKWERLNADEHIEVPEDMTRLTLDTIGLCGFNYRFNSFYRDQPHPFITSMVRALDEAMNKLQRANPDDPAYDENKRQFQEDIKVMNDLVDKIIADRKASGEQSDDLLTHMLNGKDPETGEPLDDENIRYQIITFLIAGHETTSGLLSFALYFLVKNPHVLQKAAEEAARVLVDPVPSYKQVKQLKYVGMVLNEALRLWPTAPAFSLYAKEDTVLGGEYPLEKGDELMVLIPQLHRDKTIWGDDVEEFRPERFENPSAIPQHAFKPFGNGQRACIGQQFALHEATLVLGMMLKHFDFEDHTNYELDIKETLTLKPEGFVVKAKSKKIPLGGIPSLSTEQSAKKVRKKVENAHNTPLLVLYGSNMGTAEGTARDLADIAMSKGFAPQVATLDSHAGNLPREGAVLIVTASYNGHPPDNAKEFVDWLDQASADEVKGVRYSVFGCGDKNWATTYQKVPAFIDETLAAKGAENIAERGEADASDDFEGTYEEWREHMWSDVAAYFNLDIENSEDNKSTLSLQFVDSAADMPLAKMHGAFSANVVASKELQQPGSERSTRHLEIELPKEASYQEGDHLGVIPRNYEGIVNRVATRFGLDASQQIRLEAEEEKLAHLPLGKTVSVEELLQYVELQDPVTRTQLRAMAAKTVCPPHKVELEALLEKQTYKEQVLAKRLTMLELLEKYPACEMEFSEFIALLPSMRPRYYSISSSPRVDEKQASITVSVVSGEAWSGYGEYKGIASNYLAELQEGDTITCFISTPQSGFALPKDSQTPIIMVGPGTGVAPFRGFVQARKQLKEQGQSLGEAHLYFGCRSPHEDYLYQEELENAQNEGIITLHTAFSRVPNQPKTYVQHVMEQDGTKLIELLDQGAHFYICGDGSQMAPDVEATLIKSYADVHEVSEADARLWLQQLEEKGRYAKDVWAG

>CYP102A51_ortholog(BG04_163)Bacillus megaterium NBRC 15308 = ATCC 14581

MTIKEMPQPKTFGELKNLPLLNTDKPVQALMKIADELGEIFKFEAPGRVTRYLSSQRLIKEACDESRFDKNLSQALKFVRDFAGDGLFTSWTHEKNWKKAHNILLPSFSQQAMKGYHAMMVDIAVQLVQKWERLNADEHIEVPEDMTRLTLDTIGLCGFNYRFNSFYRDQPHPFITSMVRALDEAMNKLQRANPDDPAYDENKRQFQEDIKVMNDLVDKIIADRKASGEQSDDLLTHMLNGKDPETGEPLDDENIRYQIITFLIAGHETTSGLLSFALYFLVKNPHVLQKAAEEAARVLVDPVPSYKQVKQLKYVGMVLNEALRLWPTAPAFSLYAKEDTVLGGEYPLEKGDELMVLIPQLHRDKTIWGDDVEEFRPERFENPSAIPQHAFKPFGNGQRACIGQQFALHEATLVLGMMLKHFDFEDHTNYELDIKETLTLKPEGFVVKAKSKKIPLGGIPSPSTEQSAKKVRKKAENAHNTPLLVLYGSNMGTAEGTARDLADIAMSKGFAPQVATLDSHAGNLPREGAVLIVTASYNGHPPDNAKQFVDWLDQASADEVKGVRYSVFGCGDKNWATTYQKVPAFIDETLAAKGAENIADRGEADASDDFEGTYEEWREHMWSDVAAYFNLDIENSEDNKSTLSLQFVDSAADMPLAKMHGAFSTNVVASKELQQPGSARSTRHLEIELPKEASYQEGDHLGVIPRNYEGIVNRVTARFGLDASQQIRLEAEEEKLAHLPLAKTVSVEELLQYVELQDPVTRTQLRAMAAKTVCPPHKVELEALLEKQAYKEQVLAKRLTMLELLEKYPACEMKFSEFIALLPSIRPRYYSISSSPRVDEKQASITVSVVSGEAWSGYGEYKGIASNYLAELQEGDTITCFISTPQSEFTLPKDPETPLIMVGPGTGVAPFRGFVQARKQLKEQGQSLGEAHLYFGCRSPHEDYLYQEELENAQSEGIITLHTAFSRMPNQPKTYVQHVMEQDGKKLIELLDQGAHFYICGDGSQMAPAVEATLMKSYADVHQVSEADARLWLQQLEEKGRYAKDVWAG

>CYP102A51_ortholog(BMD_3248)Bacillus megaterium DSM 319

MTIKEMPQPKTFGELKNLPLLNTDKPVQALMKIADELGEIFKFEAPGRVTRYLSSQRLIKEACDESRFDKNLSQALKFVRDFTGDGLFTSWTHEKNWKKAHNILLPSFSQQAMKGYHAMMVDIAVQLVQKWERLNADEHIEVPEDMTRLTLDTIGLCGFNYRFNSFYRDQPHPFITSMVRALDEAMNKLQRANPDDPAYDENKRQFQEDIKVMNDLVDKIIADRKASGEQSDDLLTHMLNGKDPETGEPLDDENIRYQIITFLIAGHETTSGLLSFALYFLVKNPHVLQKAAEEAARVLVDPVPSYKQVKQLKYVGMVLNEALRLWPTAPAFSLYAKEDTVLGGEYPLEKGDELMVLIPQLHRDKTIWGDDVEEFRPERFENPSAIPQHAFKPFGNGQRACIGQQFALHEATLVLGMVLKHFDFEDHTNYELDIKETLTLKPEGFVVKAKSKKIPLGGIPSPSPEQSAKKVRKKAENAHNTPLLVLYGSNMGTAEGTARDLADIAMSKGFAPQVATLDSHAGNLPREGAVLIVTASYNGHPPDNAKQFVDWLDQASADEVKGVRYSVFGCGDKNWATTYQKVPAFIDETLAAKGAENIADRGEADASDDFEGTYEEWREHMWSDVAAYFNLDIENSEDNKSTLSLQFVDSAADMPLAKMHGAFSTNVVASKELQQPGSARSTRHLEIELPKEASYQEGDHLGIIPRNYEGIVNRVTARFGLDASQQIRLEAEEEKLAHLPLAKTVSVEELLQYVELQDPVTRTQLRAMAAKTVCPPHKVELEALLEKQAYKEQVLAKRLTMLELLEKYPACEMEFSEFIALLPSIRPRYYSISSSPRVDEKQASITVSVVSGEAWSGYGEYKGIASNYLAELQEGDTITCFISTPQSEFTLPKDPETPLIMVGPGTGVAPFRGFVQARKQLKEKGQSLGEAHLYFGCRSPHEDYLYQEELENAQNEGIITLHTAFSRVPNQPKTYVQHVMEQDGKKLIELLDQGAHFYICGDGSQMAPDVEATLMKSYAGVHQVSEADARLWLQQLEEKGRYAKDVWAG

>CYP102A51_ortholog(BMQ_3237)Bacillus megaterium QM B1551

MPIKEMPQPKTFGELKNLPLLNTDKPIQTLMKIADELGEIFKFEAPGRVTRYLSSQRLIKEACDESRFDKNLSQALKFVRDFAGDGLFTSWTHEKNWKKAHNILLPSFSQQAMKGYHAMMVDIAVQLIQKWERLNTDEHIEVPEDMTRLTLDTIGLCGFNYRFNSFYRDQPHPFITSMVRALDEAMNKLQRANPDDPAYDENKRQFQEDIKVMNDLVDKIITDRKASGEQSDDLLTHMLNGKDPETGEPLDDENIRYQIITFLIAGHETTSGLLSFALYFLVKNPHVLQKAAEEAARVLVDPVPSYKQVKQLKYVGMVLNEALRLWPTAPAFSLYAKEDTVLGGEYPLEKGDELMVLIPQLHRDKTIWGDDVEEFRPERFENPSAIPQHAFKPFGNGQRACIGQQFALHEATLVLGMMLKHFDFEDHTNYELDIKETLTLKPEGFVVKAKSKQIPLGGIPSPSREQSAKKERKTVENAHNTPLLVLYGSNMGTAEGTARDLADIAMSKGFAPQVATLDSHAGNLPREGAVLIVTASYNGHPPDNAKEFVDWLDQASADEVKGVRYSVFGCGDKNWATTYQKVPAFIDETLAAKGAENIAERGEADASDDFEGTYEEWREHMWSDLAAYFNLDIENSEENASTLSLQFVDSASDMPLAKMHRAFSANVVASKELQKPGSARSTRHLEIELPKEASYQEGDHLGVIPRNYEGIVNRVATRFGLDASQQIRLEAEEEKLAHLPLGKTVSVEELLQYVELQDPVTRTQLRAMAAKTVCPPHKVELEVLLEKQAYKEQVLAKRLTMLELLEKYPACEMEFSEFIALLPSMRPRYYSISSSPRVDEKQASITVSVVSGEAWSGYGEYKGIASNYLANLQEGDTITCFVSTPQSGFTLPKGPETPLIMVGPGTGVAPFRGFVQARKQLKEQGQSLGEAHLYFGCRSPHEDYLYQKELENAQNEGIITLHTAFSRVPNQPKTYVQHVMEQDGKKLIELLDQGAHFYICGDGSQMAPDVEATLMKSYAEVHQVSEADARLWLQQLEEKGRYAKDVWAG

>CYP102A52(BEH_16435)Bacillus endophyticus

MEKERTIPQPKTYGPLGSLPLMDKDKPMQTFMKIGQELGPIFQFQFPHALGTFVSGHDLVAEVCDEEKFQKQLGVSLTSAREFAGDGLFTSYTEEPNWKKAHNILLPSFSQRAMKGYHHMMLDIATQLVEKWERLNKEEEIDVPEDMTRLTLDTIGLCGFGYRFNSFYREGAHPFVDSMVRALDESMNKMNRLPIQDKLMVRKKRQFQEDKMFMFELADEIIKERKKQGEVDGDDLLAHMLNGKDPETGEKLDDENIRYQMITFLIAGHETTSGLLSFAIYYLLKNPRVLKKAYKEVDEVLTGMPTYEDVRKLKYIRMILNEALRLWPTAPAFSLYAKEDIMLGGKYPIEKNQSVSVLLGNLHRDKRVWGENVEEFRPERFQDEEKIPHHAYKPFGNGQRACIGQQFALHEATLVLGMILQRFNIVDHRNYELKVKETLTLKPDNFTIKVSTREREYAALNEHKSENKAAEKVEQDVIIEEHNTPLLVLHGSNLGTAEGIANELAELGERYGFSTTIASLNSYSGKLPISGAVLIVSASYNGKAPDNATEFVNWLETSKNDLSGVHYAVFGCGDRNWASTYQRIPRVIDEKMTKLGAERIVDRGEGDASGDFELHLDEWKNQLTKKVKETFNVEETKEVAEKKGLNVTFVKENVQHPLVKTYKAQEAEILLNKELQREESGRSTRHIEISLPNGVSYKEGDHLGILPQNSAELVNRVLKRYGLDGSESIILEGDVYGLQHLPLNKPVRVRDVLTFNVELQEPVSRKALQELAEATVCPPHKYELEALLGDHYSENVTNKRLTMLSLLEKYEACELSFERFLTLCPPLKPRYYSISSSPLKSNQKASITVSVVEGKAWSGDGMYHGVASSYLAQCQAGEKVMMFINTPQSRFELPADESRPLIMVGPGTGVAPFRGFLQARSLLKEKGKTLGEAHLYFGCRNEAHDFIYKDELEKAESDGIVTLHTAFSRMESCDKTYVQHLIEQDAAEVISILVEKEGALYICGDGKSMAPAVESTLKKAYQDIKHKSEEEAERFLLDLEEQGRFAKDVWAG

>CYP102A53(AR543_14735)Paenibacillus bovis

MMSTIPQPKTFGPLGNLPLIDAEHPLESVMKLLDEHGPLIKLEFPDREEIYTSDPHLVKELSDESKFDKNVWSPLQRVRPFAGDGLFTSWTNEPNWGKAHRLLMPSFSQRAMQDYHDKMVDIAVQLVQKWSRLNRDEPVEVPDDMTRLTLDTIGLCAFNYRFNSFYSQEPHPFVESMVEALDEAMNQGHRLEIQNKLALHKKREFQKNIDYMFEVADGLIEQRKENGDHGENDLLSRMLNGVDPETGDKLPDENIRYQMITFLIAGHETTSGLLSFALYFLLNNPDKLEKARAEVDRVLADPIPGYKQVRDLKYIRMILREALRLWPTAPAFSRMAKEDTILAGQYPLQKGQSVNIISPSLHRNREVWGDNAEAFEPERFEDESQIPEYAYKPFGHGMRACIGQQFAMYEATLVLGMLVKHFEFEDAEQYQLKIREALTLKPDGFRMKVHPRTARVAVAIPGTEGTEQQNEPEVSQAVQKHDTPLLALYGSNLGTSEAVARELEEGGKTYGFETSAAPLDDYAGNLPQEGVLMIVASSYNGQPTSNAEKFVNWLQNADSSSCKGVKYVVFGCGDHNWASTYQRIPKLIDELLEQKGAERIFRRGEGDASGDFEMQLDEWRSELWPELFKIFNVEINEQELKKENAIQVQFVNSPVGSPLVRNYNAVECEIAEAYELQQPDSGRSTLHLEIALPPGETYHEGDHMGIIPRNPQSLVQRVLRRFRMDGGERIRITGQGSSAAYLPLDRPVSIMELLTLSVELQDVATRAQIRAMADNNQCPPHRKELEALLDNETYHREILQKRVTLLDLMDEYMSCEMSFEQFLGLLPALKPRYYSISSSPAVQPDRASITVSVIAGPARSGKGEYYGIATSYLSELKKGDTVLMFIRSPRSGFDLPEDPQSPMIMVGPGTGVAPFRGFLQARHHMQQQGTALGEARLYFGCRTPDKDYLYRDELQRYANEGIVKLYTAFSRQEPNHKQYVQDMMREHAEELIGLLDESAKLYICGDGNKMAPDVEKALQEMYSDVHQASAEQAEQWLGELQQNGRYVKDVWTGI

>CYP106A1_ortholog(BG04_4190)Bacillus megaterium NBRC 15308 = ATCC 14581

MNKEVIPVTEIPKFQSRAEEFFPIQWYKEMLNNSPVYFHEETNTWNVFQYEHVKQVLSNYDFFSSDGQRTTIFVGDNSKKKSTSPITNLTNLDPPDHRKARSLLAAAFTPRSLKNWEPRIKQIAADLVEAIQKNSTINIVDDLSSPFPSLVIADLFGVPVKDRYQFKKWVDILFQPYDQERLEEIEQEKQRAGAEYFQYLYPIVIEKRSNLSDDIISDLIQAEVDGETFTDEEIVHATMLLLGAGVETTSHAIANMFYSFLYDDKSLYSELRNNRELAPKAVEEMLRYRFHISRRDRTVKQDNELLGVKLKKGDVVIAWMSACNMDETMFENPFSVDIHRPTNKKHLTFGNGPHFCLGAPLARLEMKIILEAFLEAFSHIEPFEDFELEPHLTASATGQSLTYLPMTVYR

>CYP106A1_ortholog(BMD_1855)Bacillus megaterium DSM 319

MNKEVIPVTEIPKFQSRAEEFFPIQWYKEMLNNSPVYFHEETNTWNVFQYEHVKQVLSDYEFFSSDGQRTTIFVGDNSKKKSTSPITNLTNLDPPDHRKARSLLAAAFTHRSLKNWEPRIKQIAADLVEAIQKNPTINIVDDLSSPFPSLVIADLFGVPVKDRYQFKKWVDILFQPYDQERLEEIEQEKQRAGAEYFQYLYPIVIEKRSNLSDDIISDLIQAEFDGETFTDEEIVHATMLLLGAGVETTSHAIANMFYSFLYDDKSLYSELRNNRELAPKAVEEMLRYRFHISRRDRTVKQDNELLGVKLKKGDVVIAWMSACNMDETMFENPFSVDIHRPTNKKHLTFGNGPHFCLGAPLARLEMKIILEAFLEAFSHIEPFEDFELEPHLTASATGQSLTYLPMTVYR

>CYP106A1_ortholog(BMWSH_3391)Bacillus megaterium WSH-002

MNKEVIPVTEIPKFQSRTEEFFPIQWYKEMLNNSPVYFHEETNTWNVFQYDHVKQVLSNYEFFSSDGQRTTIFVGDNSKKKSTSPITNLTNLDPPDHRKARSLLAAAFTPRSLKNWEPRIKQIAADLVEAIQKNSTINIVEDLSSPFPSLVIADLFGVPVKDRYQFKKWVDILFQPYDQERLEEIQQEKQCAGAEYFQYLYPIVVEKRSNLSDDIISDLIQAEVDGETFTDEEIVHATMLLLGAGVETTSHAIANMFYSFLYDDKSLYSKLRNNRELAPKAVEEMLRYRFHISRRDRTVKQDNELLGVKLKKGDVVIAWMSACNMDKNMFENPFSVDIHRPTNKKHLTFGNGPHFCLGAPLARLEMKIILEAFLETFSHIEPFEDFELESHLTASATGQSLTYLPMTVYR

>CYP106A1_ortholog(BMQ_1861)Bacillus megaterium QM B1551

MNKEVIPVTEIPKFQSRAEEFFPIQWYKEMLNNHPVYFHEETNTWNVFQYDHVKQVLSNYEFFSSDGQRTTIFVGDNSKKKSTSPITNLTNLDPPDHRKVRSLLAAAFTPRSLKNWEPRIKQIAAELVEAIQKNLTIDIVDDLSSPFPSLVIADLFGVPVKDRYQFKKWVDILFQPYDQERLEKIEQEKQRAGAEYFQYLYPIVIEKRSNLSDDIISDLIQAEVDGETFTDEEIVHATMLLLGAGVETTSHAIANMFYSFLYDDESLYSELRNNRELAPKAVEEMLRYRFHISRRDRTVKQDNDLLGVKLKKGDVVIAWMSACNMDENMFKTPFSVDIHRPTNKKHLTFGNGPHFCLGAPLARLEMTIILEAFLEAFSHIEPFEDFELESHLTASATGQSLTYLPMTVYR

>CYP106A2_ortholog(UP17_19080)Bacillus simplex

MMKEVIAIKEFTRFKTRTEEFSPYAWCKRMLENDPVSYHEGTDTWNVFKYEDVKRVLSDYKHFSSVRKRTTISVGTDSGEGAVPEKIKITESDPPEHRKRRSLLAAAFTPRSLQNWEPRIQEIADELIGQMDEETEIDIVQSLASPLPIIVMSDLMGVPSKDRLLFKKWVDILFLPFDKEKQEEVNELKQVAAKEYYQYLYPIVVQKRLNPADDIISDLLKSEVDGEMFTDDEVVRTTMLILGAGVETTSHLLANSFYSLLYDDVEVYQELHENLDLVPQAVEEMLRFRFNLIKLDRTVQEDNDLLGVELKKGENVVVWMSAANLDEAMFEDAFTLNIHRPNNKKHLTFGNGPHFCLGAPLARLEAKIALTTFLKKFKHIEAVPTFQVEENLTDSATGQTLTSLPLKARRTH

>CYP106A6_ortholog(A361_03810)Bacillus oceanisediminis

MLKEVIPVNEITNFKSRAEEFFPIEWYKDMLHHHPVYYHEQTNTWNVFTYEGVKQVLGNYEFFSSAGPRTTIFVGANDKHEKASPLTNLTLVDPPDHRKGRSLLAAAFTPRSLKNWEPRIKQIAEELVENIQDNNEINIVEALAAPLPSMVIADLFGVPIQDRAQFKEWVDILFQPYDKERLEDIELQKQNAAKEYFQYLYPIVVQKRSNLSDDIISDLIQAEVDGEKFTDNEIVQVTMLLLGAGVETTSHAIANTFYSLLYDDESLYGELRNDLELVPNAVEEMLRYRFHMSRRDRTVKKDNNLLGVELKEGDVVIAWMSACNMDHRMFEDPFSINIHRPNNKKHLTFGNGPHFCLGAPLARLEMKIALETFVKKFSSIEPVEGFELEKNLTASATGQSLTNLPMNVYK

>CYP106A7(BS614_28810)Paenibacillus xylanexedens

MRPTKEAKRVTKSSIISLSEITNFQSKEEEFSPYRWYRRMLDQEPVIYNEETDSWHVFKYDLVKTVLNDHEHFSSVRKRSISSVGYSSSEEEGTDSEHHIPDKLDIHNVDPPEHRKRRSLLASAFTPRSLKLWEPRIEAVAEELIREFEHQSEVDIIKAYTSTFPIIIMSDLLGIPSNDHHLFKGWVDTMFMPSTDATFDQINQLKKIAGEEYFKYLYPFVVSKRSNLGEDIISDLIQVEHDGEHFTDEEIVRTTMFILGAGIETTSNLLANSFYALLYDQPELYVELRDNLELVPAAVEEMLRYRFHISKMDRMVKEDNDLLGVKMKKGDAIVAWMSAANMDENMFEDPFKLNIHRSNNNKQLAFGFGTHFCLGAPLARLEGKIVLTTFLKTFNKIEPVDGFILEDNLTPSAAGQSLIRLPLKLYK

>CYP106A8(BS614_17870)Paenibacillus xylanexedens

MYKEVIRVEDITGFQSRSEEFFPLHWFRKMLSEHPVYYHEDTDTWNVFRYDDVKQVLSNHEYFSAEGTRTTIAVGAKNNEGTPPDKLNISSIDPPRHQKSRSLLSAAFTPRSLKNWEPRIRNIAEQLVADIEPNTTIDIVQALAAPLPSMVMADLLGIPLTDSHRFKNWVDILFQPTNPKTAEESELKKQTAAKEYYQFLYPIVVYKRTHPGEDIITDLLNVDVEGEKFTEDEVVRTTMLLLGAGIETTSHMVSNTFYSFLYDDPSLYGQLRQNPEWVPLAVEEMLRYRFHNAKRHRTVKQDNQLLGVDLKKGDVVISWMSAANMDEQMFENPFELNIHRRNNKRHLTFGNGPHFCLGAPLARMELSIALTAFVEKIARIEPVESFDLENNLATSAPGQSLTHLPVKIVG

>CYP106A9(KNP414_04629)Paenibacillus mucilaginosus KNP414

MEVEFLMNQQLIPVQDISRFQSRSEEFFPLDWYKRMLTDNPVYYHEETDTWNVFRYDHVKLVLSNHEFFSAEGTRTTFQIGAKNKEGTVPEKLSLTNMDPPRHQKYRSLLSAAFTPRSLKNWEPRIRQVVAKLIDEMEGGTTVDIVQSLAGPLPSIAMADLLGVPSKDNLLFKNWVDILFQPARGGDARELEERKGTAAREYFQYLYPIVLQKRAEPADDIISDLLQVELEGERFEDDEVVKTTMLLLGAGIETTSHMLSSTFYSFLYDDPALYGQLRENPELIPLAVEEMLRYRFHCSKRHRTVKQDNDLLGVELKKGDVVISWMSAANMDERMFENPFTLDIHRANNKKHLTFGSGPHFCLGAPLARLELNLALTEFLPRFSLIEPVAGFDLESHLADSAPGQTLTSLPVTIYK

>CYP106A9_ortholog(PM3016_4040)Paenibacillus mucilaginosus 3016

MNQQLIPVQDISRFRSRSEEFFPLDWYKRMLTDNPVYYHEETDTWNVFRYDHVKLVLSNHEFFSAEGTRTTFQIGAKNKEGTVPEKLSLTNMDPPRHQKYRSLLSAAFTPRSLKNWEPRIRQVVAKLIDEMEGGTTVDIVQSLAGPLPSIAMADLLGVPSKDNLLFKNWVDILFQPARGGDARELEERKGTAAREYFQYLYPIVLQKRAEPADDIISDLLQVELEGERFEDDEVVKTTMLLLGAGIETTSHMLSSTFYSFLYDDPALYGQLRENPELIPLAVEEMLRYRFHCSKRHRTVKQDNDLLGVELKKGDVVISWMSAANMDERMFENPFTLDIHRANNKKHLTFGSGPHFCLGAPLARLELNLALTEFLPRFSRIEPVAGFDLESHLADSAPGQTLTSLPVTIYK

>CYP106A9_ortholog(B2K_20985)Paenibacillus mucilaginosus K02

MNQQLIPVQDISRFRSRSEEFFPLDWYKRMLTDNPVYYHEETDTWNVFRYDHVKLVLSNHEFFSAEGTRTTFQIGAKNKEGTVPEKLSLTNMDPPRHQKYRSLLSAAFTPRSLKNWEPRIRQVVAKLIDEMEGGTTVDIVQSLAGPLPSIAMADLLGVPSKDNLLFKNWVDILFQPARGGDARELEERKGTAAREYFQYLYPIVLQKRAEPADDIISDLLQVELEGERFEDDEVVKTTMLLLGAGIETTSHMLSSTFYSFLYDDPALYGQLRENPELIPLAVEEMLRYRFHCSKRHRTVKQDNDLLGVELKKGDVVISWMSAANMDERMFENPFTLDIHRANNKKHLTFGSGPHFCLGAPLARLELNLALTEFLPRFSRIEPVAGFDLESHLADSAPGQTLTSLPVTIYK

>CYP106A10(BEH_10270)Bacillus endophyticus

MEKEVIALKEIMKAKKRTDEFFPINWYKEKLESDPVYFHEETNTWNVFKYKHVKEVLSNYKFFSSEGERTTISVGAKSKEGQTPDKVQLTSVDPPAHRKSRSLLSAAFTPRSLKEWEPRIRQIVRNLLDNIEENSENDIVQVLTGPLPAVVMADLFGLSGTDQMLFKKWVDILFQPFDKNKQEDIEAQKQKAAKEYYEYLYPYVAERRFNLSDDIISDLIRAEVDDEKFTDDEIVRITMMILGAGIETTSHMLANTFYALLYDDQSIYKELRSNIELVPNTVEEMLRYRFHMSRRDRTVKQDNDLLGVPLKKGDVVIAWMSAANVDEEVFENAFSLDIHRSNNKKHLTFGSGAHFCLGAPLARLEGQIALEEFVQRFSKIEPVEGFELEENLTDSATGQSLTHLPLKVYK

>CYP106A11(IJ21_43710)Paenibacillus sp. 32O-W

MQNEIISLAEITQFGSPSEEFNPYDWYRRKLENEPISYHEATDTWNVFRYEDVKRVLSDHEYFSSVRTRTTISVGADNDEGRHMSKINLSSDPPEHQKSRSLIAAAFTPRSLKLWEPRIQEIASQLLKEMEDAPVIDVVKDFTGALPTIVIADLLGVPSEDRLLFKQWVDHLFLPLPKDNAEDVNELKRRAAKEYYDYLFPHIVRKRSNLTDDIISDLIRVEVDGERLSDDEIVRTTMFLLGAGIETTSHLLANTFYSFLYDDTSIYSEVRASRELLPNTVEEMLRYRFHIAKMDRTVKQDNRVLGVHLKKGDVVVAWMSAANMDKEVFEDPFTLNIHRPNNKKHLTFGIGPHFCLGAPLARLEANIGIGMFMDQFQQIEPVPGFQLEENLTPSAAGQMLTQLPLRVHRA

>CYP106A12(GYMC10_2785)Paenibacillus sp. Y412MC10

MNHEIISLEEITQFRSPIEEFNPYEWYKHKLQHEPIVYNQATQTWNVFRYDDVKRVLKDHEYFSSVRTRTMISVGADNDEGQKMSKINLQSDPPEHQKSRALISAAFTPRSLKLWEPRIQEIADQLLQEMGDAHVIDIVQHYASALPTIVIADLLGVPSEDRLLFKEWVNHLFLPLPKNHSEDVQELKRHAAKEYYNYLYPHIVQKRANLADDIISDLIQAEVDGDRLSDDEIVRTTMFILGAGIETTTHLLANTFYSFLYDNNQIYGEIQANRELLPNAIEEMLRYRFNIAKMDRTVKQDNHVLGVDMKRGDLVVAWMSAANMDEEVFEDPFTLNIHRTNNKKHLTFGNGAHFCLGAPLARLEANIAISRFMDFFPRIEPVPEFKLEDNLSPSAAGQMLTQLPIRVHR

>CYP106B1_ortholog(BAMEG_1960)Bacillus anthracis CDC 684

MASPENVILVHEISKLKTKEELWNPYEWYQFMRDNHPVHYDDEQDVWNVFLYDDVNRVLSDYSLFSSRRERRQFAIPPLETRININSTDPPEHRNVRSIVSKAFTPRSLEQWKPRIQSIANELVKDIENCSEVDIVEQFAAPLPVTVISDLLGVPTTDRKKIKAWSDILFMPYSKEKFNDLDAEKGIALNEFKAYLLPIVQEKRYHLTDDIISDLIRAEYEGERLTDEEIVTFSLGLLAAGNETTTNLIINSFYCFLVDSPATYKEVREKPKLISKAVEEVLRYRFPVTLARRITEDTNIFGPLMKKDQMVVAWVSAANLDEKKFSQASKFNIHRIGNEKHLTFGKGPHFCLGAPLARLEAEIALTTFINAFEKIALSPSFNIEQCILENEQTLKFLPIRLKPQ

>CYP106B1_ortholog(BAA_2698)Bacillus anthracis A0248

MASPENVILVHEISKLKTKEELWNPYEWYQFMRDNHPVHYDDEQDVWNVFLYDDVNRVLSDYSLFSSRRERRQFAIPPLETRININSTDPPEHRNVRSIVSKAFTPRSLEQWKPRIQSIANELVKDIENCSEVDIVEQFAAPLPVTVISDLLGVPTTDRKKIKAWSDILFMPYSKEKFNDLDAEKGIALNEFKAYLLPIVQEKRYHLTDDIISDLIRAEYEGERLTDEEIVTFSLGLLAAGNETTTNLIINSFYCFLVDSPATYKEVREKPKLISKAVEEVLRYRFPVTLARRITEDTNIFGPLMKKDQMVVAWVSAANLDEKKFSQASKFNIHRIGNEKHLTFGKGPHFCLGAPLARLEAEIALTTFINAFEKIALSPSFNIEQCILENEQTLKFLPIRLKPQ

>CYP106B1_ortholog(BA_2632)Bacillus anthracis Ames

MASPENVILVHEISKLKTKEELWNPYEWYQFMRDNHPVHYDDEQDVWNVFLYDDVNRVLSDYSLFSSRRERRQFAIPPLETRININSTDPPEHRNVRSIVSKAFTPRSLEQWKPRIQSIANELVKDIENCSEVDIVEQFAAPLPVTVISDLLGVPTTDRKKIKAWSDILFMPYSKEKFNDLDAEKGIALNEFKAYLLPIVQEKRYHLTDDIISDLIRAEYEGERLTDEEIVTFSLGLLAAGNETTTNLIINSFYCFLVDSPATYKEVREKPKLISKAVEEVLRYRFPVTLARRITEDTNIFGPLMKKDQMVVAWVSAANLDEKKFSQASKFNIHRIGNEKHLTFGKGPHFCLGAPLARLEAEIALTTFINAFEKIALSPSFNIEQCILENEQTLKFLPIRLKPQ

>CYP106B1_ortholog(HYU01_13075)Bacillus anthracis HYU01

MASPENVILVHEISKLKTKEELWNPYEWYQFMRDNHPVHYDDEQDVWNVFLYDDVNRVLSDYSLFSSRRERRQFAIPPLETRININSTDPPEHRNVRSIVSKAFTPRSLEQWKPRIQSIANELVKDIENCSEVDIVEQFAAPLPVTVISDLLGVPTTDRKKIKAWSDILFMPYSKEKFNDLDAEKGIALNEFKAYLLPIVQEKRYHLTDDIISDLIRAEYEGERLTDEEIVTFSLGLLAAGNETTTNLIINSFYCFLVDSPATYKEVREKPKLISKAVEEVLRYRFPVTLARRITEDTNIFGPLMKKDQMVVAWVSAANLDEKKFSQASKFNIHRIGNEKHLTFGKGPHFCLGAPLARLEAEIALTTFINAFEKIALSPSFNIEQCILENEQTLKFLPIRLKPQ

>CYP106B1_ortholog(A16_26690)Bacillus anthracis A16

MASPENVILVHEISKLKTKEELWNPYEWYQFMRDNHPVHYDDEQDVWNVFLYDDVNRVLSDYSLFSSRRERRQFAIPPLETRININSTDPPEHRNVRSIVSKAFTPRSLEQWKPRIQSIANELVKDIENCSEVDIVEQFAAPLPVTVISDLLGVPTTDRKKIKAWSDILFMPYSKEKFNDLDAEKGIALNEFKAYLLPIVQEKRYHLTDDIISDLIRAEYEGERLTDEEIVTFSLGLLAAGNETTTNLIINSFYCFLVDSPATYKEVREKPKLISKAVEEVLRYRFPVTLARRITEDTNIFGPLMKKDQMVVAWVSAANLDEKKFSQASKFNIHRIGNEKHLTFGKGPHFCLGAPLARLEAEIALTTFINAFEKIALSPSFNIEQCILENEQTLKFLPIRLKPQ

>CYP106B1_ortholog(DJ46_1415)Bacillus anthracis Vollum

MASPENVILVHEISKLKTKEELWNPYEWYQFMRDNHPVHYDDEQDVWNVFLYDDVNRVLSDYSLFSSRRERRQFAIPPLETRININSTDPPEHRNVRSIVSKAFTPRSLEQWKPRIQSIANELVKDIENCSEVDIVEQFAAPLPVTVISDLLGVPTTDRKKIKAWSDILFMPYSKEKFNDLDAEKGIALNEFKAYLLPIVQEKRYHLTDDIISDLIRAEYEGERLTDEEIVTFSLGLLAAGNETTTNLIINSFYCFLVDSPATYKEVREKPKLISKAVEEVLRYRFPVTLARRITEDTNIFGPLMKKDQMVVAWVSAANLDEKKFSQASKFNIHRIGNEKHLTFGKGPHFCLGAPLARLEAEIALTTFINAFEKIALSPSFNIEQCILENEQTLKFLPIRLKPQ

>CYP106B1_ortholog(GBAA_2632)Bacillus anthracis Ames Ancestor

MASPENVILVHEISKLKTKEELWNPYEWYQFMRDNHPVHYDDEQDVWNVFLYDDVNRVLSDYSLFSSRRERRQFAIPPLETRININSTDPPEHRNVRSIVSKAFTPRSLEQWKPRIQSIANELVKDIENCSEVDIVEQFAAPLPVTVISDLLGVPTTDRKKIKAWSDILFMPYSKEKFNDLDAEKGIALNEFKAYLLPIVQEKRYHLTDDIISDLIRAEYEGERLTDEEIVTFSLGLLAAGNETTTNLIINSFYCFLVDSPATYKEVREKPKLISKAVEEVLRYRFPVTLARRITEDTNIFGPLMKKDQMVVAWVSAANLDEKKFSQASKFNIHRIGNEKHLTFGKGPHFCLGAPLARLEAEIALTTFINAFEKIALSPSFNIEQCILENEQTLKFLPIRLKPQ

>CYP106B1_ortholog(A16R_27040)Bacillus anthracis A16R

MASPENVILVHEISKLKTKEELWNPYEWYQFMRDNHPVHYDDEQDVWNVFLYDDVNRVLSDYSLFSSRRERRQFAIPPLETRININSTDPPEHRNVRSIVSKAFTPRSLEQWKPRIQSIANELVKDIENCSEVDIVEQFAAPLPVTVISDLLGVPTTDRKKIKAWSDILFMPYSKEKFNDLDAEKGIALNEFKAYLLPIVQEKRYHLTDDIISDLIRAEYEGERLTDEEIVTFSLGLLAAGNETTTNLIINSFYCFLVDSPATYKEVREKPKLISKAVEEVLRYRFPVTLARRITEDTNIFGPLMKKDQMVVAWVSAANLDEKKFSQASKFNIHRIGNEKHLTFGKGPHFCLGAPLARLEAEIALTTFINAFEKIALSPSFNIEQCILENEQTLKFLPIRLKPQ

>CYP106B1_ortholog(BAS2452)Bacillus anthracis Sterne

MASPENVILVHEISKLKTKEELWNPYEWYQFMRDNHPVHYDDEQDVWNVFLYDDVNRVLSDYSLFSSRRERRQFAIPPLETRININSTDPPEHRNVRSIVSKAFTPRSLEQWKPRIQSIANELVKDIENCSEVDIVEQFAAPLPVTVISDLLGVPTTDRKKIKAWSDILFMPYSKEKFNDLDAEKGIALNEFKAYLLPIVQEKRYHLTDDIISDLIRAEYEGERLTDEEIVTFSLGLLAAGNETTTNLIINSFYCFLVDSPATYKEVREKPKLISKAVEEVLRYRFPVTLARRITEDTNIFGPLMKKDQMVVAWVSAANLDEKKFSQASKFNIHRIGNEKHLTFGKGPHFCLGAPLARLEAEIALTTFINAFEKIALSPSFNIEQCILENEQTLKFLPIRLKPQ

>CYP106B1_ortholog(H9401_2507)Bacillus anthracis H9401

MASPENVILVHEISKLKTKEELWNPYEWYQFMRDNHPVHYDDEQDVWNVFLYDDVNRVLSDYSLFSSRRERRQFAIPPLETRININSTDPPEHRNVRSIVSKAFTPRSLEQWKPRIQSIANELVKDIENCSEVDIVEQFAAPLPVTVISDLLGVPTTDRKKIKAWSDILFMPYSKEKFNDLDAEKGIALNEFKAYLLPIVQEKRYHLTDDIISDLIRAEYEGERLTDEEIVTFSLGLLAAGNETTTNLIINSFYCFLVDSPATYKEVREKPKLISKAVEEVLRYRFPVTLARRITEDTNIFGPLMKKDQMVVAWVSAANLDEKKFSQASKFNIHRIGNEKHLTFGKGPHFCLGAPLARLEAEIALTTFINAFEKIALSPSFNIEQCILENEQTLKFLPIRLKPQ

>CYP106B1_ortholog(BCAH820_2650)Bacillus cereus AH820

MASPENVILVHEISKLKTKEELWNPYEWYQFMRDNHPVHYDDEQDVWNVFLYDDVNRVLSDYSLFSSRRERRQFAIPPLETRININSTDPPEHRNVRSIVSKAFTPRSLEQWKPRIQSIANELVKDIENCSEVDIVEQFAAPLPVTVISDLLGVPTTDRKKIKAWSDILFMPYSKEKFNDLDAEKGIALNEFKAYLLPIVQEKRYHLTDDIISDLIRAEYEGERLTDEEIVTFSLGLLAAGNETTTNLIINSFYCFLVDSPATYKEVREKPKLISKAVEEVLRYRFPVTLARRITEDTNIFGPLMKKDQMVVAWVSAANLDEKKFSQASKFNIHRIGNEKHLTFGKGPHFCLGAPLARLEAEIALTTFINAFEKIALSPSFNIEQCILENEQTLKFLPIRLKPQ

>CYP106B1_ortholog(BF38_3812)Bacillus thuringiensis HD1011

MASPENVILVHEISKLKTKEELWNPYEWYQFMRDNHPVHYDDEQDVWNVFLYDDVNRVLSDYSLFSSRRERRQFAIPPLETRININSTDPPEHRNVRSIVSKAFTPRSLEQWKPRIQSIANELVKDIENCSEVDIVEQFAAPLPVTVISDLLGVPTTDRKKIKAWSDILFMPYSKEKFNDLDAEKGIALNEFKAYLLPIVQEKRYHLTDDIISDLIRAEYEGERLTDEEIVTFSLGLLAAGNETTTNLIINSFYCFLVDSPATYKEVREKPKLISKAVEEVLRYRFPVTLARRITEDTNIFGPLMKKDQMVVAWVSAANLDEKKFSQASKFNIHRIGNEKHLTFGKGPHFCLGAPLARLEAEIALTTFINAFEKIALSPSFNIEQCILENQQTLKFLPIRLKPQ

>CYP106B1_ortholog(BACI_c26070)Bacillus cereus biovar anthracis CI

MASPENVILVHEISKLKTKEELWNPYEWYQFMRDNHPVHYDDEQDVWNVFLYDDVNRVLSNYSLFSSRRERRQFAIPPLETRININSTDPPEHRNVRSIVSKAFTPRSLEQWKPRIQSIANELVKDIENCSEVDIVEQFAAPLPVTVISDLLGVPTTDRKKIKAWSDILFMPYSKEKFNDLDAEKGIALNEFKAYLLPIVQEKRYHLTDDIISDLIRAEYEGERLTDEEIVTFSLGLLAAGNETTTNLIINSFYCFLVDSPATYKEVREKPKLISKAVEEVLRYRFPVTLARRITEDTNIFGPLMKKDQMVVAWVSAANLDEKKFSQASKFNIHRIGNEKHLTFGKGPHFCLGAPLARLEAEIALTTFINAFEKIALSPSFNIEQCILENEQTLKFLPIRLKAQ

>CYP106B1_ortholog(BT9727_2414)Bacillus thuringiensis HD-771

MASPENVILVHEISKLKTKEELWNPYEWYQFMRDNHPVHYDDEQDVWNVFLYDDVNRVLSDYRLFSSRRERRQFAIPPLETRININSTDPPEHRNVRSIVSKAFTPRSLEQWKPRIQSIANELVKDIENCSEVDIVEQFAAPLPVTVISDLLGVPTTDRKKIKAWSDILFMPYSKEKFNDLDAEKGIALNEFKAYLLPIVQEKRYHLTDDIISDLIRAEYEGERLTDEEIVTFSLGLLAAGNETTTNLIINSFYCFLVDSPATYKEVREKPKLISKAVEEVLRYRFPVTLARRITEDTNIFGPLMKKDQMVVAWVSAANLDEKKFSQASKFNIHRIGNEKHLTFGKGPHFCLGAPLARLEAEIALTTFINAFEKIALSPSFNIEQCILENEQTLKFLPIRLKAQ

>CYP106B1_ortholog(BCA_2723)Bacillus cereus 03BB102

MDSPENVILVHEISKLKTKEELWNPYEWYQFMRDNHPVHYDDEQDVWNVFLYDDVNRVLSDYRLFSSRRERRQFAIPPLETRININSTDPPEHRNVRSIVSKAFTPRSLEQWKPRIQSIANELVKDIENCSEVDIVEQFAAPLPVTVISDLLGVPTTDRKKIKTWSDILFMPYSKEKFNDLDAKKGIALNEFKAYLLPIVQEKRYYLTDDIISDLIRAEYEGERLTDEEIVTFSLGLLAAGNETTTNLIINSFYCFLVDSPATYKELREKPKLISKAIEEVLRYRFPVTLARRITEDTNIFGPLMKKDQMVVAWVSAANLDEKKFSQASKFNIHRIGNEKHLTFGKGPHFCLGAPLARLEAEIALTTFINAFEKIALSPSFNIEQCILENEQTLKFLPIRLKPQ

>CYP106B1_ortholog(BALH_2368)Bacillus thuringiensis Al Hakam

MDSPENVILVHEISKLKTKEELWNPYEWYQFMRDNHPVHYDDEQDVWNVFLYDDVNRVLSDYRLFSSRRERRQFAIPPLETRININSTDPPEHRNVRSIVSKAFTPRSLEQWKPRIQSIANELVKDIENCSEVDIVEQFAAPLPVTVISDLLGVPTTDRKKIKTWSDILFMPYSKEKFNDLDAKKGIALNEFKAYLLPIVQEKRYYLTDDIISDLIRAEYEGERLTDEEIVTFSLGLLAAGNETTTNLIINSFYCFLVDSPATYKELREKPKLISKAIEEVLRYRFPVTLARRITEDTNIFGPLMKKDQMVVAWVSAANLDEKKFSQASKFNIHRIGNEKHLTFGKGPHFCLGAPLARLEAEIALTTFINAFEKIALSPSFNIEQCILENEQTLKFLPIRLKPQ

>CYP106B1_ortholog(BCE_2659)Bacillus cereus ATCC 10987

MASPENVILVHEISKLKTKEELWNPYEWYQFMRDNHPVHYDEEQDVWNVFLYEDVNRVLSDYRLFSSRRERRQFSIPPLETRININSTDPPEHRNVRSIVSKAFTPRSLEQWKPRIQAIANELVQHIGKYSEVNIVEEFAAPLPVTVISDLLGVPTTDRKKIKAWSDILFMPYSKEKFNDLDVEKGIALNEFKAYLLPIVQEKRYHLTDDIISDLIRAEYEGERLTDEEIVTFSLGLLAAGNETTTNLIINSFYCFLVDSPGTYKELREEPTLISKAIEEVLRYRFPITLARRITEDTNIFGPLMKKDQMVVAWVSAANLDEKKFSQASKFNIHRIGNEKHLTFGKGPHFCLGAPLARLEAEIALTTFINAFEKIALSPSFNLEQCILENEQTLKFLPICLKTQ

>CYP106B1_ortholog(BCK_21685)Bacillus cereus FRI-35

MASPENVILVHEISKLKTKEELWNPYEWYQFMRDNHPVHYDEEQDVWNVFLYEDVNRVLSDYRLFSSRRERRQFSIPPLETRININSTDPPEHRNVRSIVSKAFTPRSLEQWKPRIQAIANELVQHIGKYSEVNIVEEFAAPLPVTVISDLLGVPTTDRKKIKAWSDILFMPYSKEKFNDLDVEKGIALNEFKAYLLPIVQEKRYHLTDDIISDLIRAEYEGERLTDEEIVTFSLGLLAAGNETTTNLIINSFYCFLVDSPGTYNELREEPTLISKAIEEVLRYRFPITLARRITEDTNIFGPLMKKDQMVVAWVSAANLDEKKFSQASKFNIHRIGNEKHLTFGKGPHFCLGAPLARLEAEIALTTFINAFEKIALSPSFNLEQCILENEQTLKFLPICLKTQ

>CYP106B1(BCE33L2377)Bacillus cereus E33L

MASPENVILVHEISKLKTKEELWNPYEWYQFMRDNHPVYYDEEQDVWNVFLYEDVNRVLSDYRLFSSRRERRQFSIPPLETRININSTDPPEHRNVRSIVSKAFTPRSLEQWKPRIQAIADELVKDIEKFSEVDIVEQFAASLPVTVISDLLGVPTTDRKKIKAWSDILFMPYSKEKFNDLDAEKGIALNEFKAYLLPIVQEKRYLLTDDIISDLIRAEYEGERLTDEEIVIFSLGLLAAGNETTTNLIINSFYCFLMDSPGTYKELRKEPKLISKAIEEVLRYRFPVTLARRITEDTNIFGPLMKKDQMVVAWVSAANLDEKKFAQASKFNIHRIGNEKHLTFGKGAHFCLGAPLARLEAEIALSTFINAFEKIELSSSFDLEKCILKNEQTLKFLPICLKTQ

>CYP106B1_ortholog(BcrFT9_02073)Bacillus cereus FT9

MATPENVILVHEISKLKTKEELWNPYEWYQFMRDNHPVYYDEEQDVWNVFLYEDVNRVLSDYRLFSSRRERRQFSIPPLETRININSTDPPEHRNVRSIVSKAFTPRSLEQWKPRIQAIANELVQHIGKYSEVNIVEEFAAPLPVTVISDLLGVPTTDRKKIKAWSDILFMPYSKEKFNDLDAEKGIALNEFKAYLLPIVQEKRYLLTDDIISDLIRAEYEGERLTDEEIVTFSLGVLAAGNETTTNLIINSLYCFLVDSPGTYKELRKEPKLISKAIEEVLRYRFPVTLARRITEDTNIFGPLMKKDQMVVAWVSAANLDEKKFAQASKFNIHRIGNEKHLTFGKGAHFCLGAPLARLEAEIALSTFINAFEKIELSSSFDLEKCILENEQTLKFLPICLKTQ

>CYP106B1_ortholog(BcerKBAB4_2508)Bacillus mycoides KBAB4

MASPENVILVHEISKLKTKEELWNPYEWYEFMRDNHPVHYDEEQDVWNVFLYDDVNRVLSDYRLFSSRRERRQFAIPPLETRININSTDPPEHRNVRSIVSKAFTPRSLEQWKPRIQAIANELVQHIRKYSEVNIVEEFAVALPVTVISDLLGVPTTDRKKIKEWSDILFMPYSKEKFNDLDVEKGIALNEFKTYLLPIVQEKRYHLTDDIISDLIRAEYEGERLTDEEIVTFSLGLLAAGNETTTNLIINSFYCFLVDSPGIYEELRKKPTLISKAIEEVLRYRFPITLARRITADTNIFGPFMKKDQMIVAWVSAANSDEKKFLQASKFNLHRIGNEKHLTFGKGPHFCLGAPLARLEAEIALTTFINAFEKIELSPSFNLEKCILENEQTLKYLPILLKTR

>CYP106B1_ortholog(bwei_2323)Bacillus mycoides WSBC 10204

MPYSKEKFNDLDVEKGIALNEFKTYLLPIVQEKRYHLTDDIISDLIRAEYEGERLTDEEIVTFSLGLLAAGNETTTNLIINSFYCFLVDSPGIYEELRKEPTLISKAIEEVLRYRFPITLARRITADTNIFGPFMKKDQMIVAWVSAANLDEKKFLQASKFNLHRIGNEKHLTFGKGPHFCLGAPLARLEAEIELTTFINAFEKIELSPSFNLEKCILENEQTLKYLPILLKTR

>CYP106B1_ortholog(BG05_3318) Bacillus mycoides ATCC 6462

MPTTDRKKIKEWSDILFMPYSKEKFNDLDVEKGIALNEFKTYLLPIVQEKRYHLTDDIISDLIRAEYEGERLTDEEIVTFSLGLLAAGNETTTNLIINSFYCFLVDSPGIYKELRKEPTLISKAIEEVLRYRFPITLARRITADTNIFGPFMKKDQMIVTWVSAANSDEKKFLQASKFNLHRIGNEKHLTFGKGPHFCLGAPLARLEAEIALTTFINAFEKIELSPSFNLEKCILENEQTLKYLPILLKTR

>CYP106B4(CY96_11910)Bacillus bombysepticus

MASPENVILVHEISKLKTKEELWNPYEWYRLMRDNHPVHYDEEQDVWNVFLYDDVNRVLSDYRLFSSRRERRQFSIPPLETRININSTDPPEHRNVRSIVSKAFTPRSLEQWKPRIQAIADELVQHIEKCSEVNIVEQFAAPLPVTVISDLLGVPTTDRKKIKEWSDILFMPYSKEKFNDLDAEKGIALNEFKAYLLPIVQEKRYHLTDDIISDLIRAEYEGERLTDEEIVTFSLGLLAAGNETTTNLIINSFYCFLVDSPGIYEELRKEPNLISKAIEEVLRYRFPVTLARRITEDTNIFGSLMKKDQMIVAWVSAANLDEKKFSQASQFNVHRTGNEKHLTFGKGPHFCLGAPLARLEAEIALTTFINAFEKIELSPSFCLEKCILENEQTLKYLPIRLKAK

>CYP106B4_ortholog(AQ980_14355)Bacillus thuringiensis YWC2-8

MASPENVILVHEISKLKTKEELWNPYEWYRLMRDNHPVHYDEEQDVWNVFLYDDVNRVLSDYRLFSSRRERRQFSIPPLETRININSTDPPEHRNVRSIVSKAFTPRSLEQWKPRIQAIADELVQHIEKCSEVNIVEQFAAPLPVTVISDLLGVPTTDRKKIKEWSDILFMPYSKEKFNDLDAEKGIALNEFKAYLLPIVQEKRYHLTDDIISDLIRAEYEDERLTDEEIVTFSLGLLAAGNETTTNLIINSFYCFLVDSPGIYEELRKEPNLISKAIEEVLRYRFPVTLARRITEDTNIFGSLMKKDQMIVAWVSAANLDEKKFSQASQFNVHRTGNEKHLTFGKGPHFCLGAPLARLEAEIALTTFINAFEKIELSPSFCLEKCILENEQTLKYLPIRLKAK

>CYP106B4_ortholog(BTK_16660)Bacillus thuringiensis serovar kurstaki HD-1

MASPENVILVHEISKLKTKEELWNPYEWYRLMRDNHPVHYDEEQDVWNVFLYDDVNRVLSDYRLFSSRRERRQFSIPPLETRININSTDPPEHRNVRSIVSKAFTPRSLEQWKPRIQAIADELVQHIEKCSEVNIVEQFAAPLPVTVISDLLGVPTTDRKKIKEWSDILFMPYSKEKFNDLDAEKGIALNEFKAYLLPIVQEKRYHLTDDIISDLIRAEYEDERLTDEEIVTFSLGLLAAGNETTTNLIINSFYCFLVDSPGIYEELRKEPNLISKAIEEVLRYRFPVTLARRITEDTNIFGSLMKKDQMIVAWVSAANLDEKKFSQASQFNVHRTGNEKHLTFGKGPHFCLGAPLARLEAEIALTTFINAFEKIELSPSFCLEKCILENEQTLKYLPIRLKAK

>CYP106B4_ortholog(YBT1520_16645)Bacillus thuringiensis serovar kurstaki YBT-1520

MASPENVILVHEISKLKTKEELWNPYEWYRLMRDNHPVHYDEEQDVWNVFLYDDVNRVLSDYRLFSSRRERRQFSIPPLETRININSTDPPEHRNVRSIVSKAFTPRSLEQWKPRIQAIADELVQHIEKCSEVNIVEQFAAPLPVTVISDLLGVPTTDRKKIKEWSDILFMPYSKEKFNDLDAEKGIALNEFKAYLLPIVQEKRYHLTDDIISDLIRAEYEDERLTDEEIVTFSLGLLAAGNETTTNLIINSFYCFLVDSPGIYEELRKEPNLISKAIEEVLRYRFPVTLARRITEDTNIFGSLMKKDQMIVAWVSAANLDEKKFSQASQFNVHRTGNEKHLTFGKGPHFCLGAPLARLEAEIALTTFINAFEKIELSPSFCLEKCILENEQTLKYLPIRLKAK

>CYP106B4_ortholog(BCB4264_A2656)Bacillus cereus B4264

MASPENVILVHEISKLKTKEELWNPYEWYRLMRDNHPVHYDEEQDVWNVFLYDDVNRVLSDYRLFSSRRERRQFSIPPLETRININSTDPPEHRNVRSIVSKAFTPRSLEQWKPRIQAIADELVQHIEKCSEVNIVEQFAAPLPVTVISDLLGVPTTDRKKIKEWSDILFMPYSKEKFNDLNAEKGIALNEFKAYLLPIVQEKRYHLTDDIISDLIRAEYEGERLTDEEIVTFSLGLLAAGNETTTNLIINSFYCFLVDSPGIYEELRKEPNLISKAIEEVLRYRFPVTLARRITEDTNIFGPLMKKDQMIVAWVSAANLDEKKFSQASQFNVHRTGNEKHLTFGKGPHFCLGAPLARLEAEIALTTFINTFEKIELSPSFCLEKCILENEQTLKYLPIRLKAK

>CYP106B4_ortholog(H175_ch2660)Bacillus thuringiensis serovar thuringiensis IS5056

MASPENVILVHEISKLKTKEELWNPYEWYRLMRDNHPVHYDEEQDVWNVFLYDDVNRVLSDYRLFSSRRERRQFSIPPLETRININSTDPPEHRNVRSIVSKAFTPRSLEQWKPRIQAIADELVQHIEKCSEVNIVEQFAAPLPVTVISDLLGVPTTDRKKIKEWSDILFMPYSKEKFNDLDAEKGIALHEFKAYLLPIVQEKRYHLTDDIISDLIRAEYEGERLTDEEIVTFSLGLLAAGNETTTNLIINSFYCFLVDSPGIYEELRKEPNLILKAIEEVLRYRFPVTLARRITEDTNIFGPFMKKNQMIVAWVSAANLDEKKFSQASQFNVHRTGNEKHLTFGKGPHFCLGAPLARLEAEIALTTFINAFEKIELSPSFCLEKCILENEQTLKYLPIRLKAK

>CYP106B4_ortholog(BTB_c27420)Bacillus thuringiensis Bt407

MASPENVILVHEISKLKTKEELWNPYEWYRLMRDNHPVHYDEEQDVWNVFLYDDVNRVLSDYRLFSSRRERRQFSIPPLETRININSTDPPEHRNVRSIVSKAFTPRSLEQWKPRIQAIADELVQHIEKCSEVNIVEQFAAPLPVTVISDLLGVPTTDRKKIKEWSDILFMPYSKEKFNDLDAEKGIALHEFKAYLLPIVQEKRYHLTDDIISDLIRAEYEGERLTDEEIVTFSLGLLAAGNETTTNLIINSFYCFLVDSPGIYEELRKEPNLILKAIEEVLRYRFPVTLARRITEDTNIFGPFMKKNQMIVAWVSAANLDEKKFSQASQFNVHRTGNEKHLTFGKGPHFCLGAPLARLEAEIALTTFINAFEKIELSPSFCLEKCILENEQTLKYLPIRLKAK

>CYP106B4_ortholog(CT43_CH2615)Bacillus thuringiensis serovar chinensis CT-43

MASPENVILVHEISKLKTKEELWNPYEWYRLMRDNHPVHYDEEQDVWNVFLYDDVNRVLSDYRLFSSRRERRQFSIPPLETRININSTDPPEHRNVRSIVSKAFTPRSLEQWKPRIQAIADELVQHIEKCSEVNIVEQFAAPLPVTVISDLLGVPTTDRKKIKEWSDILFMPYSKEKFNDLDAEKGIALHEFKAYLLPIVQEKRYHLTDDIISDLIRAEYEGERLTDEEIVTFSLGLLAAGNETTTNLIINSFYCFLVDSPGIYEELRKEPNLILKAIEEVLRYRFPVTLARRITEDTNIFGPFMKKNQMIVAWVSAANLDEKKFSQASQFNVHRTGNEKHLTFGKGPHFCLGAPLARLEAEIALTTFINAFEKIELSPSFCLEKCILENEQTLKYLPIRLKAK

>CYP106B4_ortholog(BMB171_C2370)Bacillus subtilis subsp. subtilis BAB-1

MASPENVILVHEISKLKTKEELWNPYEWYRLMRDNHPVHYDEEQDVWNVFLYDDVNRVLSDYRLFSSRRERRQFSIPPLETRININSTDPPEHRNVRSIVSKAFTPRSLEQWKPRIQAIADELVQHIEKCSEVNIVEQFAAPLPVTVISDLLGVPTTDRKKMKEWSDILFMPYSKEKFNDLDAEKGIALHEFKAYLLPIVQEKRYHLTDDIISDLIRAEYEGERLTDEEIVTFSLGLLAAGNETTTNLIINSFYCFLVDSPGIYEELRKEPNLILKAIEEVLRYRFPVTLARRITEDTNIFGPFMKKNQMIVAWVSAANLDEKKFSQASQFNVHRTGNEKHLTFGKGPHFCLGAPLARLEAEIALTTFINAFEKIELSPSFCLEKCILENEQTLKYLPIRLKAK

>CYP106B4_ortholog(BTF1_10360)Bacillus thuringiensis HD-789

MASPENVILVHEISKLKTKEELWNPYEWYRLMRDNHPVHYDEEQDVWNVFLYDDVNRVLSDYRLFSSRRERRQFSIPPLETRININSTDPPEHRNARSIVSKAFTPRSLEQWKPRIQAIADELVQHIEKYSEVNIVEQFAAPLPVTVISDLLGVPTTDRKKIKEWSDILFMPYSKEKFNDLDAEKGIALNEFKAYLLPIVQEKRYHLTDDIISDLIRAEYEGERLTDEEIVTFSLGLLAAGNETTTNLIINSFYCFLVDSPGIYEDLRKKPSLISKAIEEVLRYRFPVTLARRITEDTNIFGPLMKKDQMIVAWVSAANLDEKKFSHPSQFNVHRIGNEKHLTFGKGPHFCLGAPLARLEAEIALTTFINAFEKIELSPSFCLEKCILENEQTLKYLPIRLKAK

>CYP106B4_ortholog(BCG9842_B2668)Bacillus cereus G9842

MASPENVILVHEISKLKTKEELWNPYEWYRLMRDNHPVHYDEEQDVWNVFLYDDVNRVLSDYRLFSSRRERRQFSIPPLETRININSTDPPEHRNVRSIVSKAFTPRSLEQWKPRIQAIADELVQHIEKYSEVNIVEQFAALLPVTVISDLLGVPTTDRKKIKEWSDILFMPYSKEKFNDLDAEKEIALNEFKAYLLPIVQEKRYHLTDDIISDLIRAEYEGERLTDEEIVTFSLGLLAAGNETTTNLIINSFYCFLVDSPGIYEDLRKTPSLISKAIEEVLRYRFPVTLARRITEDTNIFGPLMKKDQMIVAWVSAANLDEKKFSHPSQFNVHRIGNEKHLTFGKGPHFCLGAPLARLEAEIALTTFINAFEKIELSPSFCLEKCILENEQTLKYLPIRLKAK

>CYP106B4_ortholog(BTG_06600)Bacillus thuringiensis HD-771

MASPENVILVHEISKLKTKEELWNPYEWYRLMRDNHPVRYDEEQDVWNVFLYDDVNRVLSDYRLFSSRRERRQFSIPPLETRININSTDPPEHRNVRSIVSKAFTPRSLEQWKPRIQAIADELVQHIEKYSEVNIVEQFAAPLPVTVISDLLGVPTTDRKKIKAWSDILFMPYSKEKFNDLDAEKEIALNEFKAYLLPIVQEKRYHLTDDIISDLIRAEYEGERLTDEEIVTFSLGLLAAGNETTTNLIINSFYCFLVDSPGIYEDLRKNPSLISKAIEEVLRYRFPVTLARRITEDTNIFGPLMKKDQMIVAWVSAANLDEKKFSHPSQFNVHRIGNEKHLTFGKGPHFCLGAPLARLEAEIALTTFINAFEKIELSPSFCLEKCILENEQTLKYLPIRLKAK

>CYP106B4_ortholog(YBT1518_14485)Bacillus thuringiensis YBT-1518

MASPENVILVHEISKLKTKEELWNPYEWYRLMRDNHPVHYDEEQDVWNVFLYDDVNRVLSDYRLFSSRRERRQFSIPPLETRININSTDPPEHRNVRSIVSKAFTPRSLGQWKTRIQAIADELVQHIEKCSEVNIVEQFAAPLPVTVISDLLGVPTTDRKKIKEWSDILFMPYSKEKFNDLDAEKGIALHEFKAYLLPIVQEKRYHLTDDIISDLIRAEYEGERLTDEEIVTFSLGLLAAGNETTTNLIINSFYCFLVDSPGIYEELRKEPNLILKAIEEVLRYRFPVTLARRITEDTNIFGPFMKKNQMIVAWVSAANLDEKKFSQASQFNVHRTGNEKHLTFGKGPHFCLGAALARLEAEIALTTFINAFEKIELSPSFCLEKCILENEQTLKYLPIRLKAKEKS

>CYP106B4_ortholog(HD73_3376)Bacillus thuringiensis serovar kurstaki HD73

MASPENVILVHEISKLKTKEELWNPYEWYRLMRDNHPVHYDEEQDVWNVFLYDDVNRVLSDYRLFSSRRERRQFSIPPLETRININSTDPPEHRNVRSIVSKAFTPRSLEQWKPRIQAIADELVQHIEKCSEVNIVEQFAAPLPVTVISDLLGVPTTDRKKIKEWSDILFMPYSKEKFNDLDAEKGIALNEFKAYLLPIVQEKRYHLTDDIISDLIRAEYEDERLTDEEIVTFSLGLLAAGNETTTNLIINSFYCFLVDSPGIYEELRKEPNLISKAIEEVLRYRFPVTLARRITEDTNIFGSLMKKDQMIVAWVSAANLDEKNFHKPLNLMYIEQEMKSI

>CYP106B5(MC28_1835)Bacillus thuringiensis MC28

MDSPENVILVHEISKLKTKEELWNPYGWYQFMRDNHPVHYDEEQDVWNVFLYEDVNRVLSDYRLFSSRRERRQFSIPPLETRININSTDPPEHRNVRSIVSKAFTPRSLEQWKPRIKAIADELVKNIKKYDEVNIVNQFAAPLPVTVISDLLGVPTTDRKQIKEWSDILFMPYSKEKFNDLDAEKGIALNEFNAYLLPIVQEKRYHLTDDIISDLIRAEYEGERLTDEEIVTFSLGLLAAGNETTTNLIINSFYCFLVDSPGIYQELREEPKLVAKAIEEVLRYRFPVTLARRITEDTNIFGPLMKKDQMIVAWVSAANLDENKFSHASKYNLHRIGNEKHLTFGKGPHFCLGAPLARLEAEIALSTFINAFEKIELSPSFNLERCILKNKQTLKYLPIRLKTQ

>CYP106B6(DJ92_5186) Bacillus pseudomycoides 219298

MNSPENVILVHEISKLKTKEELWNPYEWYKHMRENHPVYYDAEQDVWNVFLYNDVNRVLSDYRLFSSRRDRRQFSVPPLDTRVNLNSSDPPEHRNVRSIVSKAFTPRSLQQWKPQIQAIANELVKDMNNYDEIDIVEQFAAILPVTVISDLLGVPTTDRKKIKMWSDILFMPYSKEKFSDLDKQKEVALHEFKSYLLPIVQEKRYHLKEDIISDLIRAEYEGERLTDEEIVTFSLGLLAAGNETTTNLIINSFYCFLVDAPKVYKELSESPELIPKAIEEVLRYRFPVTLARTITEDTTIFGPKMKKGQMIVTWISAANLDENKFSQADCFDLHRPGNEKHLTFGKGPHFCLGAPLARLEAEVALTTFIRNFKKLELSPSFHLENCILENEQTLKRFPILLKK

>CYP106B7(PRIO_5418)Paenibacillus riograndensis

MNAYRQVILLNEISKLATKQAQWEPYAWYKDMRDRNPVYYDAEQDVWNVFSYDHVKRVLFDHELFSNKKERSLIPTPIQLDNRSNVNLVDPPEHRKRRALLTQAFTPRSLKEWEPRIQEIVDELITNMEGAPVIDIVQKLAIPLPVTVIADLLGVPSRARGQIKAWSDILFLPYNKKAYTDIEQQKGRAMKEFAEFLYPIVLEKRKHPADDIISDLTQAELEGEKLTDEEVVRSAIGLLGAGNETTTTLISNIFYCMLFDQPGVYQELRADLSLVPKLIEEVLRFRFGATIDRKVAQDTNVFGPEMKAGQVVVAWIGAANRDESHFTRADVFDIHRPGNQQHMTFGAGPHFCLGAPLARLEANMALASFVRRFPDIQQTDGFNLDEHLTASATGQSLKSLTIGTA

>CYP106C1(BBR47_39810)Brevibacillus brevis

MNKIVFPNEISGAENLQQQFEPYGWYAEMRKNSPVHYDEKQQVWNVFLYPDVERVLTDYHLFSSDTGKRIAGSLALKEKGITEMDPPDHGKRRALYTKAFSTRTLQEWEPRIQEISRHLLEEVKEKQSIRILGDLATPMPVIVIADLLGVPSSDWMLFKQWSDVLITSGTRETYEDITLKKEEIMKEMAVYLSPIIQEKRENPAKDFLSDLTQTEYKGQKLSDREIIDIAISLLLAGNVTTSTLLFSVFYCFLLDRPGVYRELRDNPELVDQAIEEVLRFRPPAQVLMRKVQEDTDMFGSLMKKGEIVMAWLGSANRDENTFAQGDQFDIHRPNSNKHLSFGKGCHLCLGAPLSRLEAKVMLTEMLKCYSDISIGGFENDLILHVTRD

>CYP107H1_ortholog(BS34A_32830)Bacillus sp. BS34A

MTIASSTASSEFLKNPYSFYDTLRAVHPIYKGSFLKYPGWYVTGYEETAAILKDARFKVRTPLPESSTKYQDLSHVQNQMMLFQNQPDHRRLRTLASGAFTPRTTESYQPYIIETVHHLLDQVQGKKKMEVISDFAFPLASFVIANIIGVPEEDREQLKEWAASLIQTIDFTRSRKALTEGNIMAVQAMAYFKELIQKRKRHPQQDMISMLLKGREKDKLTEEEAASTCILLAIAGHETTVNLISNSVLCLLQHPEQLLKLRENPDLIGTAVEECLRYESPTQMTARVASEDIDICGVTIRQGEQVYLLLGAANRDPSIFTNPDVFDITRSPNPHLSFGHGHHVCLGSSLARLEAQIAINTLLQRMPSLNLADFEWRYRPLFGFRALEELPVTFE

>CYP107H1_ortholog(BSU6051_30190)Bacillus subtilis subsp. subtilis 6051-HGW

MTIASSTASSEFLKNPYSFYDTLRAVHPIYKGSFLKYPGWYVTGYEETAAILKDARFKVRTPLPESSTKYQDLSHVQNQMMLFQNQPDHRRLRTLASGAFTPRTTESYQPYIIETVHHLLDQVQGKKKMEVISDFAFPLASFVIANIIGVPEEDREQLKEWAASLIQTIDFTRSRKALTEGNIMAVQAMAYFKELIQKRKRHPQQDMISMLLKGREKDKLTEEEAASTCILLAIAGHETTVNLISNSVLCLLQHPEQLLKLRENPDLIGTAVEECLRYESPTQMTARVASEDIDICGVTIRQGEQVYLLLGAANRDPSIFTNPDVFDITRSPNPHLSFGHGHHVCLGSSLARLEAQIAINTLLQRMPSLNLADFEWRYRPLFGFRALEELPVTFE

>CYP107H1_ortholog(B657_30190)Bacillus subtilis QB928

MTIASSTASSEFLKNPYSFYDTLRAVHPIYKGSFLKYPGWYVTGYEETAAILKDARFKVRTPLPESSTKYQDLSHVQNQMMLFQNQPDHRRLRTLASGAFTPRTTESYQPYIIETVHHLLDQVQGKKKMEVISDFAFPLASFVIANIIGVPEEDREQLKEWAASLIQTIDFTRSRKALTEGNIMAVQAMAYFKELIQKRKRHPQQDMISMLLKGREKDKLTEEEAASTCILLAIAGHETTVNLISNSVLCLLQHPEQLLKLRENPDLIGTAVEECLRYESPTQMTARVASEDIDICGVTIRQGEQVYLLLGAANRDPSIFTNPDVFDITRSPNPHLSFGHGHHVCLGSSLARLEAQIAINTLLQRMPSLNLADFEWRYRPLFGFRALEELPVTFE

>CYP107H1_ortholog(BSU30190)Bacillus subtilis subsp. subtilis 168

MTIASSTASSEFLKNPYSFYDTLRAVHPIYKGSFLKYPGWYVTGYEETAAILKDARFKVRTPLPESSTKYQDLSHVQNQMMLFQNQPDHRRLRTLASGAFTPRTTESYQPYIIETVHHLLDQVQGKKKMEVISDFAFPLASFVIANIIGVPEEDREQLKEWAASLIQTIDFTRSRKALTEGNIMAVQAMAYFKELIQKRKRHPQQDMISMLLKGREKDKLTEEEAASTCILLAIAGHETTVNLISNSVLCLLQHPEQLLKLRENPDLIGTAVEECLRYESPTQMTARVASEDIDICGVTIRQGEQVYLLLGAANRDPSIFTNPDVFDITRSPNPHLSFGHGHHVCLGSSLARLEAQIAINTLLQRMPSLNLADFEWRYRPLFGFRALEELPVTFE

>CYP107H1_ortholog(BSUA_03208)Bacillus subtilis subsp. subtilis JH642

MTIASSTASSEFLKNPYSFYDTLRAVHPIYKGSFLKYPGWYVTGYEETAAILKDARFKVRTPLPESSTKYQDLSHVQNQMMLFQNQPDHRRLRTLASGAFTPRTTESYQPYIIETVHHLLDQVQGKKKMEVISDFAFPLASFVIANIIGVPEEDREQLKEWAASLIQTIDFTRSRKALTEGNIMAVQAMAYFKELIQKRKRHPQQDMISMLLKGREKDKLTEEEAASTCILLAIAGHETTVNLISNSVLCLLQHPEQLLKLRENPDLIGTAVEECLRYESPTQMTARVASEDIDICGVTIRQGEQVYLLLGAANRDPSIFTNPDVFDITRSPNPHLSFGHGHHVCLGSSLARLEAQIAINTLLQRMPSLNLADFEWRYRPLFGFRALEELPVTFE

>CYP107H1_ortholog(BSUB_03208)Bacillus subtilis subsp. subtilis AG1839

MTIASSTASSEFLKNPYSFYDTLRAVHPIYKGSFLKYPGWYVTGYEETAAILKDARFKVRTPLPESSTKYQDLSHVQNQMMLFQNQPDHRRLRTLASGAFTPRTTESYQPYIIETVHHLLDQVQGKKKMEVISDFAFPLASFVIANIIGVPEEDREQLKEWAASLIQTIDFTRSRKALTEGNIMAVQAMAYFKELIQKRKRHPQQDMISMLLKGREKDKLTEEEAASTCILLAIAGHETTVNLISNSVLCLLQHPEQLLKLRENPDLIGTAVEECLRYESPTQMTARVASEDIDICGVTIRQGEQVYLLLGAANRDPSIFTNPDVFDITRSPNPHLSFGHGHHVCLGSSLARLEAQIAINTLLQRMPSLNLADFEWRYRPLFGFRALEELPVTFE

>CYP107H1_ortholog(U712_14985)Bacillus subtilis PY79

MMSIVFIFEQKGENHVTIASSTASSEFLKNPYSFYDTLRAVHPIYKGSFLKYPGWYVTGYEETAAILKDARFKVRTPLPESSTKYQDLSHVQNQMMLFQNQPDHRRLRTLASGAFTPRTTESYQPYIIETVHHLLDQVQGKKKMEVISDFAFPLASFVIANIIGVPEEDREQLKEWAASLIQTIDFTRSRKALTEGNIMAVQAMAYFKELIQKRKRHPQQDMISMLLKGREKDKLTEEEAASTCILLAIAGHETTVNLISNSVLCLLQHPEQLLKLRENPDLIGTAVEECLRYESPTQMTARVASEDIDICGVTIRQGEQVYLLLGAANRDPSIFTNPDVFDITRSPNPHLSFGHGHHVCLGSSLARLEAQIAINTLLQRMPSLNLADFEWRYRPLFGFRALEELPVTFE

>CYP107H1_ortholog(BSn5_05935)Bacillus subtilis BSn5

MMSIVFIFEQKGENHVTIASSTASSEFLKNPYSFYDTLRAVHPIYKGSFLKYPGWYVTGYEETAAILKDARFKVRTPLPESSTKYQDLSHVQNQMMLFQNPPDHRRLRTLASGAFTPRTTESYQPYIIETVHHLLDQVQGKKKMEVISDFAFPLASFVIANIIGVPEEDREQLKEWAASLIQTIDFTRSRKALTEGNIMAVQAMAYFKELIQKRKRHPQQDMISMLLKGREKDKLTEEEAASTCILLAIAGHETTVNLISNSVLCLLQHPEQLLKLRENPDLIGTAVEECLRYESPTQMTARVASEDIDICGVTIRQGEQVYLLLGAANRDPSIFTNPDVFDITRSPNPHLSFGHGHHVCLGSSLARLEAQIAINTLLQRMPSLNLADFEWRYRPLFGFRALEELPVTFE

>CYP107H1_ortholog(Q433_16375)Bacillus subtilis subsp. subtilis OH 131.1

MTIASSTASYEFLKNPYSFYDTLRAVHPIYKGSFLKYPGWYVTGYEETAAILKDARFKVRTPLPESSTKYQDLSHVQNQMMLFQNRPDHRRLRTLASGAFTPRTTESYQPYIIETVHHLLDQVQGKKKMEVISDFAFPLASFVIANIIGVPEEDREQLKEWAASLIQTIDFTRSRKALTEGNIMAVQAMAYFKELIQKRKRHPQQDMISMLLKGREKDKLTEEEAASTCILLAIAGHETTVNLISNSVLCLLQHPEQLLKLRENPDLIGTAVEECLRYESPTQMTARVASEDIDICGVTIRQGEQVYLLLGAANRDPSIFTNPDVFDITRSPNPHLSFGHGHHVCLGSSLARLEAQIAINTLLQRMPSLKLADFEWRYRPLFGFRALEELPVTFE

>CYP107H1_ortholog(A7A1_1657)Bacillus subtilis subsp. subtilis BSP1

MTIASSTASSEFLKNPYSFYDTLRAVHPIYKGSFLKYPGWYVTGYEETAAILKDARFKVRTPLPESSTKYQDLSHVQNQMMLFQNQPDHRRLRTLASGAFTPRATESYQPYIIETVHHLLDQVQGKKKMEVISDFAFPLASFVIANIIGVPEEDREQLKEWAGSLIQTIDFTRSRKALTEGNIMAVQAMAYFKELIQKRKRHPQQDMISMLLKGREKDKLTEEEAASTCILLAIAGHETTVNLISNSVLCLLQHPEQILKLRENPDLIGTAVEECLRYESPTQMTARVASEDIDICGVTIRQGEQIYLLLGAANRDPSIFTNPDVFDITRSPNPHLSFGHGHHVCLGSSLARLEAQIAINTLLQRMPSLKLADFEWRYRPLFGFRALEELPVTFE

>CYP107H1_ortholog(BsLM_3003)Bacillus sp. LM 4-2

MMSIAFIFEQKGENHVTIASSTASSEFLKNPYSFYDTLRAVHPIYKGSFLKYPGWYVTGYEETAAILKDARFKVRTPLPESSTKYQDLSHVQNQMMLFQNQPDHRRLRTLASGAFTPRTTESYQPYIIETVHQLLDQVQGEKKMEVISDFAFPLASFVIANIIGVPEEDREQLKEWAASLIQTIDFTRSRKALTEGNHMAVQAMAYFKELIQKRKRHPQQDMISMLLKGREKDKLTEEEAASTCILLAIAGHETTVNLISNSVLCLLQHPEQILKLRENPDLIGTAVEECLRYESPTQMTARVASEDIDICGVTIRQGEQVYLLLGAANRDPSIFTNPDVFDITRSPNPHLSFGHGYHVCLGSSLARLEAQIAINTLLQRMPSLKLADFEWRYRPLFGFRALEELPVTFE

>CYP107H1_ortholog(QF06_13285)Bacillus sp. YP1

MTIASSTASSEFLKNPYSFYDTLRAVHPIYKGSFLKYPGWYVTGYEETATILKDARFKVRTPLPESSTKYQDLSHVQNQMMLFQNQPDHRRLRTLASGAFTPRATESYQPYIIETVHHLLDQVQGEKKMEVISDFAFPLASFVIANIVGVPEEDREQLKEWAASLIQTIDFTRSRKALTEGNHMAVQAMAYFKELIQKRKRHPQQDMISMLLKGKENDKLTEEEAASTCILLAIAGHETTVNLISNSVLCLLQHPEQLLKLRENPDLIGTAVEECLRYESPTQMTARVASEDIDISGVTIRQGEQVYLLLGAANRDPSIFTNPDVFDITRSPNQHLSFGHGHHVCLGSSLARLEAQIAINTLLQRMPSLKLADFEWRYRPLFGFRALEELPVTFE

>CYP107H1_ortholog(BSNT_09439)Bacillus subtilis subsp. natto BEST195

MMSIAFIFEQKGENHVTIASSTASSEFLKNPYSFYDTLRAVHPIYKGSFLKYPGWYVTGYEETAAILKDARFKVRTPLPESSTKYQDLSHVQNQMMLFQNQPDHRRLRTLASGAFTPRATESYQPYIIETVHQLLDQVQGEKKMEVISDFAFPLASFVIANIIGVPEEDREQLKEWAASLIQTIDFTRSRKALTEGNHMAVQAMAYFKELIQKRKRHPQQDMISMLLKGKENDKLTEEEAASTCILLAIAGHETTVNLISNSVLCLLQHPEQLLKLRENPDLIGTAVEECLRYESPTQMTARVASEDIDISGVTIRQGEQVYLLLGAANRDPSIFTNPDVFDITRSPNPHLSFGHGHHVCLGSSLARLEAQIAINTFLQRMPSLKLADFEWRYRPLFGFRALEELPVTFE

>CYP107H1_ortholog(I33_3073)Bacillus subtilis subsp. subtilis RO-NN-1

MTIASSAASSEFLKNPYSFYDTLRAVHPIYKGSFLKYPGWYVTGYEETTAILKDARFKVRTPLPDSSTKYQDLSNVQNQMMLFQNQPDHRRLRTLASGALTPRATESYQPYIIETVHHLLDQVQGKKKMEVISDFAFPLASFVIANIIGVPEEDREQLKEWAASLIQTIDFTRSRKALTEGNHMAVQAMAYFKELIQKRKRHPQQDMISMLLKGKEKDKLTEEEAASTCILLAIAGHETTVNLISNSVLCLLQHPEQLLKLRENPDLIGTAVEECLRYESPTQMTARVASEDIDISGVTIRQGEQVYLLLGAANRDPSTFTNPDVFDITRSPNPHLSFGNGHHVCLGSSLARLEAQIAINTLLQRMPSLKLADFEWRYRPLFGFRALEELPVTFE

>CYP107H2_ortholog(BAMF_1919)Bacillus amyloliquefaciens DSM 7

MTAGLSIAHPSLHADSDFWNNPYPFYDKLRAVDPVYQGTVLKYPGRYITGYQEAEAVLKDTRFKNRIPMPEASAKYKHLKNLQKDMLLFTNHSDHKRLRMLIGKAFTLKKAERLKPFITATVHDVLDQIDHSKTADLVSDFAFPVASLVIADILGVPKEDRASFREWTADVIQAIDLTRSKKSLLKAGGTAGKLTAYFKDLIQKRKTEPQKDVITTLISEEQLTEEEVLASCILLIIAGHETTVNLICNGVFSLLKHPAELSKLLENPQLIASATEEFLRFESPAQLTARTASEDCVINQHLIKKGEQVYILLGAANRDPEVFHRPHQLDITRNPNPHLAFGKGAHVCIGSSLARIEAQTAILTLLERAPDIRLVKTDVTYRKLFGFRSLSALPVVLS

>CYP107H2_ortholog(BAMTA208_07910)Bacillus amyloliquefaciens TA208

MTAGLSIAHPSLHADSDFWNNPYPFYDKLRAVDPVYQGTVLKYPGRYITGYQEAEAVLKDTRFKNRIPMPEASAKYKHLKNLQKDMLLFTNHSDHKRLRMLIGKAFTLKKAERLKPFITATVHDVLDQIDHSKTADLVSDFAFPVASLVIADILGVPKEDRASFREWTADVIQAIDLTRSKKSLLKAGGTAGKLTAYFKDLIQKRKTEPQKDVITTLISEEQLTEEEVLASCILLIIAGHETTVNLICNGVFSLLKHPAELSKLLENPQLIASATEEFLRFESPAQLTARTASEDCVINQHLIKKGEQVYILLGAANRDPEVFHRPHQLDITRNPNPHLAFGKGAHVCIGSSLARIEAQTAILTLLERAPDIRLVKTDVTYRKLFGFRSLSALPVVLS

>CYP107H2_ortholog(LL3_02009)Bacillus amyloliquefaciens LL3

MTAGLSIAHPSLHADSDFWNNPYPFYDKLRAVDPVYQGTVLKYPGRYITGYQEAEAVLKDTRFKNRIPMPEASAKYKHLKNLQKDMLLFTNHSDHKRLRMLIGKAFTLKKAERLKPFITATVHDVLDQIDHSKTADLVSDFAFPVASLVIADILGVPKEDRASFREWTADVIQAIDLTRSKKSLLKAGGTAGKLTAYFKDLIQKRKTEPQKDVITTLISEEQLTEEEVLASCILLIIAGHETTVNLICNGVFSLLKHPAELSKLLENPQLIASATEEFLRFESPAQLTARTASEDCVINQHLIKKGEQVYILLGAANRDPEVFHRPHQLDITRNPNPHLAFGKGAHVCIGSSLARIEAQTAILTLLERAPDIRLVKTDVTYRKLFGFRSLSALPVVLS

>CYP107H2_ortholog(BAXH7_01611)Bacillus amyloliquefaciens XH7

MTAGLSIAHPSLHADSDFWNNPYPFYDKLRAVDPVYQGTVLKYPGRYITGYQEAEAVLKDTRFKNRIPMPEASAKYKHLKNLQKDMLLFTNHSDHKRLRMLIGKAFTLKKAERLKPFITATVHDVLDQIDHSKTADLVSDFAFPVASLVIADILGVPKEDRASFREWTADVIQAIDLTRSKKSLLKAGGTAGKLTAYFKDLIQKRKTEPQKDVITTLISEEQLTEEEVLASCILLIIAGHETTVNLICNGVFSLLKHPAELSKLLENPQLIASATEEFLRFESPAQLTARTASEDCVINQHLIKKGEQVYILLGAANRDPEVFHRPHQLDITRNPNPHLAFGKGAHVCIGSSLARIEAQTAILTLLERAPDIRLVKTDVTYRKLFGFRSLSALPVVLS

>CYP107H3(BSUW23_14625)Bacillus subtilis subsp. spizizenii W23

MTIASSTASSEFLKNPYPFYETLRAVHPIYKGSFLKYPGWYVTGYEETAAILKDARFKVRTPLPETSIKYQDLTHVQNQMMLFQNQPDHRRLRMPASGAFTPRMAERYRPYIDETVHHLLDQVQGEKKMEVISDFAFPLASYVIANIIGVPAEDREQLKDWAASLIQTIDFTRSRKALIEGNHSAVQAMAYFRELIQKRKRHPQQDMISMLLKGKEKDKMTEEEVASTCILLAIAGHETTVNLISNSILCLLQHPEQLLELKENPDLIATAVEECLRFESPTQMTARVASEDIDISGVTIRKGEQVYLLLGAANRDPNIFTRADVFDITRSPNPHLSFGHGHHVCLGSSLARLEAQMAIHTLLQRMPGLKLAESKWRYRPLFGFRALEELPVTFE

>CYP107H3_ortholog(GYO_3267)Bacillus subtilis subsp. spizizenii TU-B-10

MTIAFSTASSAFLKDPYPFYETLRAVHPIYKGSFLKYPGWYVTGYEETAAILKDARFKVRTPLPETSIKYQDLKHVQNQMMLFQNQPDHRRLRTLASGAFTPRMAESYRLYIDETVHHLLDQVQGEKKMEVISDFAFPLASYVIANIIGVPAEDREQLKEWASILIQTIDFTRSRKALTEGNHISVQAMAYFRELIQRRKRHPQQDMVSMLLKGKEKDKLTEEEVASTCVLLAIAGHETTVNLISNSVLCLLQHPQQLLKLKENPDLIDTAVEECLRYESPTQMTARVASEDIDISGVTIRKGEQVYLLLGAANRDPNIFMQADVFDITRSPNPHLSFGQGHHVCLGSSLARLEAQIAIHTLLQRMPGLKLADSEWRYRPLFGFRALEELPVTFE

>CYP107H4(BAPNAU_1920)Bacillus velezensis NAU-B3

MTAGLSIAHPSLHADSDFWNDPYPFYDKLRSIDPVYKGTVLKYPGWYVTGYKEAAAILKDTRFKNRIPLPEASTKYQNLSHIQHDMLLFKNQSDHKRMRMLIGKEFTAKTAESLRPCIKETVHDLLDQVQIKKNADLVSEFAFPLASLIIAEILGVPKEERYQFRQWTADVIQAIDLTRSRKTLVRASDTAGRLTSYFRDLIHKRKAHPQQDLISRFIMEEQLSKEEVLATCILLVIAGHETTVNLMCNGVFTLLKHPEQLSALRENPSLIETAVEECLRYDSPAQLTARTASEDCEINGKTIKKGEQVYILLGAANRDPSIFDQPHKMDIQRKPNPHLAFGKNAHFCIGSSLARIEAQIAILTLFERMPKLRLAAHRLEYRKLIGFRSLKELPVVIG

>CYP107H4_ortholog(NG74_01900)Bacillus velezensis

MTAGLSIAHPSLHADSDFWNDPYPFYDKLRSIDPVYKGTVLKYPGWYVTGYKEAAAILKDTRFKNRIPLPEASTKYQNLSHIQHDMLLFKNQSDHKRMRMLIGKEFTAKTAESLRPCIKETVHDLLDQVQIKKNADLVSEFAFPLASLIIAEILGVPKEERYQFRQWTADVIQAIDLTRSRKTLVRASDTAGRLTSYFRDLIHKRKAHPQQDLISRFIMEEQLSKEEVLATCILLVIAGHETTVNLMCNGVFTLLKHPEQLSALRENPSLIETAVEECLRYDSPAQLTARTASEDCEINGKTIKKGEQVYILLGAANRDPSIFDQPHKMDIQRKPNPHLAFGKNAHFCIGSSLARIEAQIAILTLFERMPKLRLAAHRLEYRKLIGFRSLKELPVVIG

>CYP107H4_ortholog(OY17_12255)Bacillus sp. BH072

MTAGLSIAHPSLHADSDFWNDPYSFYDKLRSIDPVYKGTVLKYPGWYVTGYKEAAAILKDTRFKNRIPLPEASTKYQNLSHIQHDMLLFKNQSDHKRMRMLIGKEFTAKTAESLRPCIKETVHDLLDQVQIKKNADLVSEFAFPLASLIIAEILGVPKEERYQFRQWTADVIQAIDLTRSRKTLVRASDTAGRLTSYFRDLIHKRKAHPQQDLISRFIMEEQLSKEEVLATCILLVIAGHETTVNLMCNGVFTLLKHPEQLSALRENPSLIETAVEECLRYDSPAQLTARTASEDCEINGKTIKKGEQVYILLGAANRDPSIFDQPHKMDIQRKPNPHLAFGKNAHFCIGSSLARIEAQIAILTLFERMPKLRLAAHRLEYRKLIGFRSLKELPVVIG

>CYP107H4_ortholog(MUS_2177)Bacillus amyloliquefaciens Y2

MTAGLSIAHPSLHADSDFWNDPYSFYDKLRSIDPVYKGTVLKYPGWYVTGYKEAAAILKDTRFKNRIPLPEASTKYQNLSHIQHDMLLFKNQSDHKRMRMLIGKEFTAKTAESLRPCIKETVHDLLDQVQIKKNADLVSEFAFPLASLIIAEILGVPKEERYQFRQWTADVIQAIDLTRSRKTLVRASDTAGRLTSYFRDLIHKRKAHPQQDLISRFIMEEQLSKEEVLATCILLVIAGHETTVNLMCNGVFTLLKHPEQLSALRENPSLIETAVEECLRYDSPAQLTARTASEDCEINGKTIKKGEQVYILLGAANRDPSIFDQPHKMDIQRKPNPHLAFGKNAHFCIGSSLARIEAQIAILTLFERMPKLRLAAHRLEYRKLIGFRSLKELPVVIG

>CYP107H4_ortholog(BANAU_1949)Bacillus velezensis YAU B9601-Y2

MTAGLSIAHPSLHADSDFWNDPYSFYDKLRSIDPVYKGTVLKYPGWYVTGYKEAAAILKDTRFKNRIPLPEASTKYQNLSHIQHDMLLFKNQSDHKRMRMLIGKEFTAKTAESLRPCIKETVHDLLDQVQIKKNADLVSEFAFPLASLIIAEILGVPKEERYQFRQWTADVIQAIDLTRSRKTLVRASDTAGRLTSYFRDLIHKRKAHPQQDLISRFIMEEQLSKEEVLATCILLVIAGHETTVNLMCNGVFTLLKHPEQLSALRENPSLIETAVEECLRYDSPAQLTARTASEDCEINGKTIKKGEQVYILLGAANRDPSIFDQPHKMDIQRKPNPHLAFGKNAHFCIGSSLARIEAQIAILTLFERMPKLRLAAHRLEYRKLIGFRSLKELPVVIG

>CYP107H4_ortholog(AJ82_10365)Bacillus velezensis TrigoCor1448

MTADLSIAHPSLHADSDFWNDPYPFYDKLRSIDPVYKGTVLKYPGWYVTGYKEAAAILKDTRFKNRIPLPEASTKYQNLSHIQHDMLLFKNQSDHKRMRMLIGKEFTAKTAESLRPCIKETVHDLLDQVQIKKTADLVSEFAFPLASLIIAEILGVPKEERYQFRQWTADVIQAIDLTRSRKTLVRASDTAGRLTSYFRDLIHKREAHPQQDLISRFIMEEQLSKEEVLATCILLVIAGHETTVNLMSNGVFTLLKHPEQLSALRENPSLIETAVEECLRYDSPAQLTARTASEDCEINGKTIKKGEQVYILLGAANRDPSIFDQPHKMDIQRKPNPHLAFGRNAHFCIGSSLARIEAQIAILTLFERMPKLRLAAHRLEYRKLIGFRSLKELPVVIG

>CYP107H4_ortholog(RBAU_1799)Bacillus velezensis UCMB5033

MTADLSIVHPSLHADSDFWNDPYPFYDKLRSIDPVYKGTVLKYPGWYVTGYKEAAAILKDIRFKNRIPLPEASTKYQNLSHIQHDMLLFKNQSDHKRMRMLIGKEFTAKTAESLRPCIKETVHDLLDQVQIKKTADLVSEFAFPLASLIIAEILGVPKEERYQFRQWTADVIQAIDLTRSRKTLVRASDTAGRLTSYFKDLIHKRKAHPQQDLISRFIMEEQLSKEEVLATCILLVIAGHETTVNLISNGVFTLLKHPEQLSALRENPSLIETAVEECLRYDSPAQLTARTASEDCEINGKTIKKGEQVYILLGAANRDPSIFDQPHKMDIQRKPNPHLAFGKNAHFCIGSSLARIEAQIAILTLFERMPKLRLAAHRLEYRKLIGFRSLKELPVVIG

>CYP107H4_ortholog(BASU_1779)Bacillus velezensis UCMB5113

MTADLSIAHPSLHADSDFWNDPYPFYDKLRSIDPVYKGTVLKYPGWYVTGYKEAAAILKDIRFKNRIPLPEASTKYQNLSHIQHDMLLFKNQSDHKRMRMLIGKEFTAKTAESLRPCIKETVHDLLDQVQIKKTADLVSEFAFPLASLIIAEILGVPKEERYQFRQWTADVIQAIDLTRSRKTLVRASDTAGRLTSYFKDLIHKRKAHPQQDLISRFIMEEQLSKEEVLATCILLVIAGHETTVNLISNGVFTLLKHPEQLSALRENPSLIETAVEECLRYDSPAQLTARTASEDCEINGKTIKKGEQVYILLGAANRDPSIFDQPHKMDIQRKPNPHLAFGKNAHFCIGSSLARIEAQIAILTLFERMPKLRLAAHRLEYRKLIGFRSLKELPVVIG

>CYP107H4_ortholog(BAM5036_1761)Bacillus velezensis UCMB5036

MTADLSIAHPSLHADSDFWNDPYPFYDKLRSSDPVYKGTVLKYPGWYVTGYKEAAAILKDIRFKNRIPLPEASTKYQNLSHIQHDMLLFKNQSDHKRMRMLIGKEFTAKTAESLRPCIKETVHDLLDQVQIKKNADLVSEFAFPLASLIIAEILGVPKEERYQFRQWTADVIHAIDLTRSRKTLVRASDTAGRLTSYFRDLIHKRKAHPQQDLISRFIMEEQLSKEEVLATCILLVIAGHETTVNLISNGVFTLLKHPEQLSALRENPSLIETAVEECLRYDSPTQLTARTASEDCEINGKTIKKGEQVYILLGAANRDPSIFDQSHKMDIQRKPNPHLAFGKNAHFCIGSSLARIEAQIAILTLFERMPKLRLAAHRLEYRKLIGFRSLKELPVVIG

>CYP107H4_ortholog(SB24_00535)Bacillus sp. Pc3

MTADLSIAHPSLHADSDFWNDPYPFYDKLRSSDPVYKGTVLKYPGWYVTGYKEAAAILKDIRFKNRIPLPEASTKYQNLSHIQHDMLLFKNQSDHKRMRMLIGKEFTAKTAESLRPCIKETVHDLLDQVQIKKTADLVSEFAFPLASLIIAEILGVPKEERYQFRQWTADVIHAIDLTRSRKTLVRASDTAGRLTSYFRDLIHKRKAHPQQDLISRFIMEEQLSKEEVLATCILLVIAGHETTVNLISNGVFTLLKHPEQLSALRENPSLIETAVEECLRYDSPTQLTARTASEDCEINGKTIKKGEQVYILLGAANRDPSIFDQSHKMDIQRKPNPHLAFGKNAHFCIGSSLARIEAQIAILTLFERMPKLRLAAHRLEYRKLIGFRSLKELPVVIG

>CYP107H4_ortholog(U722_09650)Bacillus amyloliquefaciens LFB112

MTAGLSTAHPSLHADSDFWNDPYPFYEKLRAIDPVYKGTVLKHPGWYVTGYKEAAAILKDTRFKNRVPFPEASTKYQNLSHIQHDMLLFKNQSDHKRMRMLIGKEFTAKTAESLRPCIKETVHDLLDQIQNKKNADLVSEFAFPLASLIIAEILGVPKEERYQFRQWTADVIQAIDLTRSRKMLVRASDTAGRLTAYFRDLIHERKAHPQEDLISRFIMGEQLSKDEVLATCILLVIAGHETTVNLMSNGVFTLLKHPEQLSALRENPSLIETAVEECLRYDSPAQLTARTASEDCEINGKTIKKGEQVYILLGAANRDPSIFDQPHKMDIQRKPNPHLAFGKNAHFCIGSSLARIEAQIAILTLFERMPKLRLAAHRLEYRKLIGFRSLKELPVVLG

>CYP107H4_ortholog(BACAU_1782)Bacillus velezensis CAU B946

MTAGLSTAHPSLHADSDFWNDPYPFYEKLRAIDPVYKGTVLKHPGWYVTGYKEAAAILKDTRFKNRVPFPEASTKYQNLSHIQHDMLLFKNQSDHKRMRMLIGKEFTAKTAESLRPCIKETVHDLLDQIQNKKNADLVSEFAFPLASLIIAEILGVPKEERYQFRQWTADVIQAIDLTRSRKMLVRASDTAGRLTAYFRDLIHERKAHPQEDLISRFIMGEQLSKDEVLATCILLVIAGHETTVNLMSNGVFTLLKHPEQLSALRENPSLIETAVEECLRYDSPAQLTARTASEDCEINGKTIKKGEQVYILLGAANRDPSIFDQPHKMDIQRKPNPHLAFGKNAHFCIGSSLARIEAQIAILTLFERMPKLRLAAHRLEYRKLIGFRSLKELPVVLG

>CYP107H4_ortholog(KSO_010245)Bacillus amyloliquefaciens IT-45

MTAGLSTAHPSLHADSDFWNDPYPFYEKLRAIDPVYKGTVLKHPGWYVTGYKEAAAILKDTRFKNRVPFPEASTKYQNLSHIQHDMLLFKNQSDHKRMRMLIGKEFTAKTAESLRPCIKETVHDLLDQIQNKKNADLVSEFAFPLASLIIAEILGVPKDERYQFRQWTADVIQAIDLTRSRKMLVRASDTAGRLTAYFRDLIHERKAHPQEDLISRFIMGEQLSKDEVLATCILLVIAGHETTVNLMSNGVFTLLKHPEQLSALRENPSLIETAVEECLRYDSPAQLTARTASEDCEINGKTIKKGEQVYILLGAANRDPSIFDQPHKMDIQRKPNPHLAFGKNAHFCIGSSLARIEAQIAILTLFERMPKLRLAAHRLEYRKLIGFRSLKELPVVLG

>CYP107H5(BGLY_0785)Bacillus glycinifermentans

MTTELDSVLGIDRDFWREPYPFYDKLRSIDPVYKGTVLKQPGWYVTGHNEAMTIFKDARFKNRIPLPETSTKYEALKNVQQNMLLFKNDSDHKRMRTLVGKEFTPKAADVLRPFIKEAADELLDQLQTGTTADIVSQFAFPLASLVIARILGVPKEERHQFRQWTANLIETIDFTRSRQTLVKGSDTASRLTAYVRDLISKRKAVPQDDLITTFISAGKLTEEEVLATCVLLVIAGHETTVNLISNGLLSLMKHPAQLGALKENPELITSAVEEFLRYESPTQLTARTASEDCGINGHVIRKGEQVYILLGAANRDPNVFDHPHVLDIERHPNPHLAFGYGAHVCPGSSLARLEAQIAIQALLERAPGLRLAGSAVQYRRLLGFRALASLPVILN

>CYP107H6(BATR1942_12885)Bacillus atrophaeus 1942

MTIKNLTIPVSLSSSEFFENPYPFYDKLRSIHPIYKGNFLKYPGWYVTGYAEAAAILKDTRFKNRIPLPENSQKYSELKNMQHHMMLFKNPPDHKRIRMMVSRAFTPRFAESYRPYINETVNVLLNEVQYKRTMDVVSDFAFPLASLIIAKMLGVPAGESYQFREWAASLIQTIDFTRSRKELTKGNDKAIKLIAYFKDLIQQKKHNPQNDLISMLMKEQQDERLSDEELLATCILLVIAGHETTVNLISNSILSLLNHPEKLMELKEKPSLIESAVEEFLRYESPTQMTARVASEDIEMNGMMIKKEEHVYILLGAANRDPKKFTHPHVLDITRNPNPHLAFGQGIHFCLGSSLARLEAQIAICTLLQRIPNLQLSTPNVQYRKLIGFRSLTELPVTF

>CYP107H6_ortholog(TD68_11905)Bacillus atrophaeus NRS 1221A

MTIKNLTIPVSLSSSEFFENPYPFYDKLRSIHPIYKGNFLKYPGWYVTGYAEAAAILKDTRFKNRIPLPENSQKYSELKNMQHHMMLFKNPPDHKRIRMMVSRAFTPRFAESYRPYINETVNVLLNEVQYKRTMDVVSDFAFPLASLIIAKMLGVPAGESYQFREWAASLIQTIDFTRSRKELTKGNDKAIKLIAYFKDLIQQKKHNPQNDLISMLMKEQQDERLSDEELLATCILLVIAGHETTVNLISNSILSLLNHPEKLMELKEKPSLIESAVEEFLRYESPTQMTARVASEDIEMNGMMIKKEEHVYILLGAANRDPKKFTHPHVLDITRNPNPHLAFGQGIHFCLGSSLARLEAQIAICTLLQRIPNLQLSTPNVQYRKLIGFRSLTELPVTF

>CYP107H7(BaLi_c08690)Bacillus paralicheniformis

MTTELHEAGPRLKSDSDFWRDPYLFYDKLRSVHPVYKGTVLKHLGWYVTGYEEAAAILRDAVFKNRVPLPEASTKYEQLHELQRNMMLFQNESGHKRMRTIVGKEFMPQKTASFRPVIEEIVHELLDQLENKKMADMVSEFAFPLASLIIADMLGVPAEERYQFRQWTADLIQTIDLTRTRKVLAKGGDTVAKLTAYFKDLIEKRKAHPSQDLISTFAGHEQLAEDEVLATCILLVIAGHETTVNLLTNGLFLLMNHPGQLSALKENPLLIESAVEECLRYESPTQLTARTASEDCEINGKIIKKGEHVYILLGAANRDPKIFQNPHVFDITRKPNPHLAFGAGAHVCLGSALARLEAQIAIPALLERLPKLKLASTDIPFRRLIGFRSLAELPVILN

>CYP107H8(BLi00771)Bacillus licheniformis DSM 13 = ATCC 14580

MTTELHEAGPSLKSDSDFWRDPYLFYDQLRSIHPVYKGTVLKHPGWYVTGYEEAAAILKEPAFINRVPLPEASTKYEQLQRLQRNMMLFQNESGHKRMRTIAGKEFMPQKTATFRPAIEETVHELLDQLENKKTADMVSEFAFPLASLIIAGMLGVPEEEWYQFRQWTADLIQTIDLTRTRKVMAKGGDTVAKLTAYFKNLIEKRKAHPSEDLISTFAGHEQLADEEVLATCILLVIAGHETTVNLLTNGLFLLMTHPDQLSELKENPLLIESAVEECLRYESPTQLTARTASEDCEINGKIIKKGEHLYILLGAANRDPKIFQNPHLFDITRKPNPHLAFGAGAHVCLGSALARLEAQIAIPALLARLPNLKLASTDVPFRRLIGFRSLAELPVILN

>CYP107H8_ortholog(BL00957)Bacillus licheniformis ATCC 14580

MTTELHEAGPSLKSDSDFWRDPYLFYDQLRSIHPVYKGTVLKHPGWYVTGYEEAAAILKEPAFINRVPLPEASTKYEQLQRLQRNMMLFQNESGHKRMRTIAGKEFMPQKTATFRPAIEETVHELLDQLENKKTADMVSEFAFPLASLIIAGMLGVPEEEWYQFRQWTADLIQTIDLTRTRKVMAKGGDTVAKLTAYFKNLIEKRKAHPSEDLISTFAGHEQLADEEVLATCILLVIAGHETTVNLLTNGLFLLMTHPDQLSELKENPLLIESAVEECLRYESPTQLTARTASEDCEINGKIIKKGEHLYILLGAANRDPKIFQNPHLFDITRKPNPHLAFGAGAHVCLGSALARLEAQIAIPALLARLPNLKLASTDVPFRRLIGFRSLAELPVILN

>CYP107H9(BpOF4_16075)Bacillus pseudofirmus

MNTVSQTKAKQIRLKRDDLFKDPYGLYKRLRERGPIYKGSILKQPGWFVTGYKETAYILKDSSFFTRIPLPEATEKYSHLKQIQSKMMLYMNKEDHRKLRLLVNKGFTPKQIAGFRPLIEEVVDSLLDELIVKEEFDVVSEFAFPLTSLVIATILGVPEEDRDRFRAWAALLLQSIDFSRSHKTLEASDQLAEELSEYFSSLIAQKRAEPGDDLISMLIKENDCTENELISTFILLVIAGHETTVNLISNTIYTFLKHPEQWKMLNENPTLAGSAIEEVLRFESPTQLTARVAMKDTEVAGKHITCGEQLYLMLGAANRDPDVFDEPDEFLITRKKVPHLSFGLGAHFCLGSTLARLEAQIALSRFSKRVHTYNQTKTEIKWRPLTGFRALEKLRITVQTSN

>CYP107H10(BEH_11250)Bacillus endophyticus

MNTTNIESFSFNTSEFFQNPYPFYEEMRSINPIYKSTLLKYQGWYITGYEETLKVLKDSRFHSRPPIPAFSRNYENIRRIQENMMLFTTPQHHRRLRSLVTKFFTPNIVNECRPFIEETVDELIDQVSCRGTMEVVSDYAFPLASSVIAKILGVPVKDYSLFKEWTATFIQSIDFTRSPKTLGNSRKATEEIIAYFKKIIREREQEPKEDLISKLIQEKQREEKLSYDELIATCVLLLIAGHETTVNLISSSIFLLIQNPRQLEKLQDDPELITTAIEEFLRFESPAQLIARTASEDIIINEAVIRKGEQVYLFLGAANRDSRKFRNSHQLEITRNPNPHLAFGHSRHFCLGSSLARLEAKIAIERLLQNVQNLQAVDGNGHYRKIIGFKSLVELHVTFDSYPDENFI

>CYP107J1_ortholog(BS34A_29190)Bacillus sp. BS34A

MSSKEKKSVTILTESQLSSRAFKDEAYEFYKELRKSQALYPLSLGALGKGWLISRYDDAIHLLKNEKLKKNYENVFTAKEKRPALLKNEETLTKHMLNSDPPDHNRLRTLVQKAFTHRMILQLEDKIQHIADSLLDKVQPNKFMNLVDDYAFPLPIIVISEMLGIPLEDRQKFRVWSQAIIDFSDAPERLQENDHLLGEFVEYLESLVRKKRREPAGDLISALIQAESEGTQLSTEELYSMIMLLIVAGHETTVNLITNMTYALMCHHDQLEKLRQQPDLMNSAIEEALRFHSPVELTTIRWTAEPFILHGQEIKRKDVIIISLASANRDEKIFPNADIFDIERKNNRHIAFGHGNHFCLGAQLARLEAKIAISTLLRRCPNIQLKGEKKQMKWKGNFLMRALEELPISF

>CYP107J1_ortholog(QF06_11525)Bacillus sp. YP1

MSSKEKKSVTILTESQLSSRAFKDEAYEFYKELRKSQALYPLSLGALGKGWLISRYDDAIHLLKNEKLKKNYENVFTAKEKRPALLKNEETLTKHMLNSDPPDHNRLRTLVQKAFTHRMILQLEDKIQHIADSLLDKVQPNKFMNLVDDYAFPLPIIVISEMLGIPLEDRQKFRVWSQAIIDFSDAPERLQENDHLLGEFVEYLESLVRKKRREPAGDLISALIQAESEGTQLSTEELYSMIMLLIVAGHETTVNLITNMTYALMCHHDQLEKLRQQPDLMNSAIEEALRFHSPVELTTIRWTAEPFILHGQEIKRKDVIIISLASANRDEKIFPNADIFDIERKNNRHIAFGHGNHFCLGAQLARLEAKIAISTLLRRCPNIQLKGEKKQMKWKGNFLMRALEELPISF

>CYP107J1_ortholog(BSU6051_26740)Bacillus subtilis subsp. subtilis 6051-HGW

MSSKEKKSVTILTESQLSSRAFKDEAYEFYKELRKSQALYPLSLGALGKGWLISRYDDAIHLLKNEKLKKNYENVFTAKEKRPALLKNEETLTKHMLNSDPPDHNRLRTLVQKAFTHRMILQLEDKIQHIADSLLDKVQPNKFMNLVDDYAFPLPIIVISEMLGIPLEDRQKFRVWSQAIIDFSDAPERLQENDHLLGEFVEYLESLVRKKRREPAGDLISALIQAESEGTQLSTEELYSMIMLLIVAGHETTVNLITNMTYALMCHHDQLEKLRQQPDLMNSAIEEALRFHSPVELTTIRWTAEPFILHGQEIKRKDVIIISLASANRDEKIFPNADIFDIERKNNRHIAFGHGNHFCLGAQLARLEAKIAISTLLRRCPNIQLKGEKKQMKWKGNFLMRALEELPISF

>CYP107J1_ortholog(U712_13095)Bacillus subtilis PY79

MSSKEKKSVTILTESQLSSRAFKDEAYEFYKELRKSQALYPLSLGALGKGWLISRYDDAIHLLKNEKLKKNYENVFTAKEKRPALLKNEETLTKHMLNSDPPDHNRLRTLVQKAFTHRMILQLEDKIQHIADSLLDKVQPNKFMNLVDDYAFPLPIIVISEMLGIPLEDRQKFRVWSQAIIDFSDAPERLQENDHLLGEFVEYLESLVRKKRREPAGDLISALIQAESEGTQLSTEELYSMIMLLIVAGHETTVNLITNMTYALMCHHDQLEKLRQQPDLMNSAIEEALRFHSPVELTTIRWTAEPFILHGQEIKRKDVIIISLASANRDEKIFPNADIFDIERKNNRHIAFGHGNHFCLGAQLARLEAKIAISTLLRRCPNIQLKGEKKQMKWKGNFLMRALEELPISF

>CYP107J1_ortholog(B657_26740)Bacillus subtilis QB928

MSSKEKKSVTILTESQLSSRAFKDEAYEFYKELRKSQALYPLSLGALGKGWLISRYDDAIHLLKNEKLKKNYENVFTAKEKRPALLKNEETLTKHMLNSDPPDHNRLRTLVQKAFTHRMILQLEDKIQHIADSLLDKVQPNKFMNLVDDYAFPLPIIVISEMLGIPLEDRQKFRVWSQAIIDFSDAPERLQENDHLLGEFVEYLESLVRKKRREPAGDLISALIQAESEGTQLSTEELYSMIMLLIVAGHETTVNLITNMTYALMCHHDQLEKLRQQPDLMNSAIEEALRFHSPVELTTIRWTAEPFILHGQEIKRKDVIIISLASANRDEKIFPNADIFDIERKNNRHIAFGHGNHFCLGAQLARLEAKIAISTLLRRCPNIQLKGEKKQMKWKGNFLMRALEELPISF

>CYP107J1_ortholog(BSU26740)Bacillus subtilis subsp. subtilis 168

MSSKEKKSVTILTESQLSSRAFKDEAYEFYKELRKSQALYPLSLGALGKGWLISRYDDAIHLLKNEKLKKNYENVFTAKEKRPALLKNEETLTKHMLNSDPPDHNRLRTLVQKAFTHRMILQLEDKIQHIADSLLDKVQPNKFMNLVDDYAFPLPIIVISEMLGIPLEDRQKFRVWSQAIIDFSDAPERLQENDHLLGEFVEYLESLVRKKRREPAGDLISALIQAESEGTQLSTEELYSMIMLLIVAGHETTVNLITNMTYALMCHHDQLEKLRQQPDLMNSAIEEALRFHSPVELTTIRWTAEPFILHGQEIKRKDVIIISLASANRDEKIFPNADIFDIERKNNRHIAFGHGNHFCLGAQLARLEAKIAISTLLRRCPNIQLKGEKKQMKWKGNFLMRALEELPISF

>CYP107J1_ortholog(BSUA_02854)Bacillus subtilis subsp. subtilis JH642

MSSKEKKSVTILTESQLSSRAFKDEAYEFYKELRKSQALYPLSLGALGKGWLISRYDDAIHLLKNEKLKKNYENVFTAKEKRPALLKNEETLTKHMLNSDPPDHNRLRTLVQKAFTHRMILQLEDKIQHIADSLLDKVQPNKFMNLVDDYAFPLPIIVISEMLGIPLEDRQKFRVWSQAIIDFSDAPERLQENDHLLGEFVEYLESLVRKKRREPAGDLISALIQAESEGTQLSTEELYSMIMLLIVAGHETTVNLITNMTYALMCHHDQLEKLRQQPDLMNSAIEEALRFHSPVELTTIRWTAEPFILHGQEIKRKDVIIISLASANRDEKIFPNADIFDIERKNNRHIAFGHGNHFCLGAQLARLEAKIAISTLLRRCPNIQLKGEKKQMKWKGNFLMRALEELPISF

>CYP107J1_ortholog(BSUB_02854)Bacillus subtilis subsp. subtilis AG1839

MSSKEKKSVTILTESQLSSRAFKDEAYEFYKELRKSQALYPLSLGALGKGWLISRYDDAIHLLKNEKLKKNYENVFTAKEKRPALLKNEETLTKHMLNSDPPDHNRLRTLVQKAFTHRMILQLEDKIQHIADSLLDKVQPNKFMNLVDDYAFPLPIIVISEMLGIPLEDRQKFRVWSQAIIDFSDAPERLQENDHLLGEFVEYLESLVRKKRREPAGDLISALIQAESEGTQLSTEELYSMIMLLIVAGHETTVNLITNMTYALMCHHDQLEKLRQQPDLMNSAIEEALRFHSPVELTTIRWTAEPFILHGQEIKRKDVIIISLASANRDEKIFPNADIFDIERKNNRHIAFGHGNHFCLGAQLARLEAKIAISTLLRRCPNIQLKGEKKQMKWKGNFLMRALEELPISF

>CYP107J1_ortholog(BSn5_0400)Bacillus subtilis BSn5

MSSKEKKSVTILTESQLSSRAFKDEAYEFYKELRKSQPLYPLSLGALGKGWLISRYDDAIHLLKNEKLKKNYENVFTAKEKRPALLKNEETLTKHMLNSDPPDHNRLRTLVQKAFTHRMILQLEDKIQHIADSLLDKVQPNKFMNLVDDYAFPLPIIVISEMLGIPLEDRQKFRVWSQAIIDFSDAPERLQENDHLLGEFVEYLESLVRKKRREPAGDLISALIQAESEGTQLSTEELYSMIMLLIVAGHETTVNLITNMTYALMCHHDQLEKLRQQPDLMNSAIEEALRFHSPVELTTIRWTAEPFILHGQEIKRKDVIIISLASANRDEKIFPNADIFDIERKNNRHIAFGHGNHFCLGAQLARLEAKIAISTLLRRCPNIQLKGEKKQMKWKGNFLMRALEELPISF

>CYP107J1_ortholog(A7A1_0793)Bacillus subtilis subsp. subtilis BSP1

MSSKEKKSVTILTESQLSSRAFKDEAYEFYKELRKSQPLYPLSLGALGKGWLISRYDDAIHLLKNEKLKKNYENVFTAKEKRPALLKNEETLTKHMLNSDPPDHNRLRTLVQKAFTHRMILQLEDKIQHIADSLLDKVQPNKFMNLVDDYAFPLPIIVISEMLGIPLEDRQKFRVWSQAIIDFSDAPERLQENDHLLGEFVEYLESLVRKKRREPAGDLISALIQAESEGTQLSTEELYSMIMLLIVAGHETTVNLITNMTYALMCHHDQLEKLRQQPDLMNSAIEEALRFHSPVELTTIRWTAEPFILHGQEIKRKDVIIISLASANRDEKIFPNADIFDIERKNNRHIAFGNGNHFCLGAQLARLEAKIAISTLLRRCPNIQLKGEKKQMKWKGNFLMRALEELPISF

>CYP107J1_ortholog(Q433_14300)Bacillus subtilis subsp. subtilis OH 131.1

MSSKEKKSVTILTESQLSSRAFKDEAYEFYKELRKSQPLYPLSLGALGKGWLISRYDDAIHLLKYEKLKKNYENVFTAKEKRPALLKNEETLTKHMLNSDPPDHNRLRTLVQKAFTHRMILQLEDKIQHIADSLLDKVQPNKFMNLVDDYAFPLPIIVISEMLGIPLEDRQKFRVWSQAIIDFSDAPERLQENDHLLGEFVEYLESLVRKKRREPAGDLISALIQAESEGTQLSTEELYSMIMLLIVAGHETTVNLITNMTYALMCHHDQLEKLRQQPDLMNSAIEEALRFHSPVELTTIRWTAEPFILHGQEIKRKDVIIISLASANRDEKIFPNADIFDIERKNNRHIAFGHGNHFCLGAQLARLEAKIAISTLLRRCPNIQLKGEKKQMKWKGNFLMRALEELPISF

>CYP107J1_ortholog(BGM20_07215)Bacillus gibsonii

MSSKEKKSVTILTESQLSSRAFKDEAYEFYKELRKSQPLYPLSLGSLGKGWLISRYDDAIHLLKNEKLKKNYENVFTAKEKRPALLKNEESLTKHMLNSDPPDHNRLRTLVQKAFTHRMILQLEDKIQHIADSLLDKVQPNKFMNLVDDYAFPLPIIVISEMLGIPLEDRQKFRVWSQAIIDFSDAPERLQENDHLLGEFVEYLESLVRKKRREPAGDLISALIQAESEGTQLSTEELYSMIMLLIVAGHETTVNLITNMTYALMCHGDQFEKLRQQPDLMNSAIEEALRFHSPVELTTIRWTAEPFILHGQEIKRKDVIIISLASANRDEKIFPNADIFDIERKNNRHIAFGHGNHFCLGAQLARLEAKIAMSTLLRRCPNIQLKGEKEQMKWKGNFLMRALEELPLSF

>CYP107J1_ortholog(BSNT_09062)Bacillus subtilis subsp. natto BEST195

MSSKEKNSVTILTESQLSSRAFKDEAYEFYKELRKSQPLYPLSLGALGKGWLISRYEDAIHLLKNEKLKKNYENVFTAKEKRPALLKNEESLTKHMLNSDPPDHNRLRTLVQKAFTHRMIFQLEDKIQHIADSLLDKVQPNKFMNLVDDYAFPLPIIVISEMLGIPLEDRQKFRVWSQAIIDFSDAPERLQENDHLLGEFVEYLESLVRKKRSEPSGDLISALIQAESEGTQLSTEELYSMIMLLIVIGHETTVNLITNMTYALMCHGDQLEKLRQQPDLMNSAIEEALWFHSPVELTTIRWTAEPFMLHGQEIKRKDVIIISLASANRDEKIFPNADIFDIERKNNRHIAFGHGNHFCLGAQLARLEAKIAISTLLRRCPNIQLKGEKEQIKWKGNFLMRALEELPLSF

>CYP107J1_ortholog(I653_12685)Bacillus subtilis subsp. subtilis BAB-1

MSSKEKNSVTILTESQLSSRAFKDEAYEFYKELRKSQPLYPLSLGALGKGWLISRYDDAIHLLKNEKLKKNYENVITAKEENRPVLLKNEEPLTKHMLNSDPPDHNRLRTLVQKAFTHRMILQLEDKIQHIADSLLDKVQPNKLMNLVDDYAFPLPIIVISEMLGIPLEDRQKFRVWSQVIIDFSDAPERLQENDHLLGEFVEYLESLVRKKRSEPSGDLISALIQAESEGAQLSTEELYSMIMLLIVAGHETTVNLITNMTYALMCHGDQFEKLRQQPDLMNSAIEEALRFHSPVELTTIRWTAEPFILHGQEIKRKDVIIISLASANRDEKIFPNADIFDIERKNNRHIAFGHGNHFCLGAQLARLEAKIAISTLLRRCPNIQLKGEKEQMKWKGNFLMRALEELPLSF

>CYP107J1_ortholog(I33_2707)Bacillus subtilis subsp. subtilis RO-NN-1

MSSKEKKSVTILTESHLSSRAFKDEAYKFYKELRKSQPLYPLSLGTLGKGWLITRYDDAIHLLKNEKLKKNYENVISAKEENRPVLLKNEEPLTKHMLNSDPPDHNRLRTLVQKAFTHRMILQLEDKIQHIADSLLDKVQPNKLMNLVDDYAFPLPIIVISEMLGIPLEDRQKFRVWSQAIIDFSDAPERLQENDHLLGEFVEYLESLVRKKRSEPSGDLISAFIQAESEGTQLSTEELYSMIMLLIVAGHETTVNLITNMTYALMCHGDQLEKLRHQPDLMNSAIEEALRFHSPVELTTIRWTAEPFILHGQEIKRKDVIIISLASANRDEKIFPNADLFDIERKSNRHIAFGHGNHFCLGAQLARLEAKIAISTLLRRCPNIQLKGEKEQMKWKGNFLMRALEELPLSF

>CYP107J2_ortholog(BAMEG_1965)Bacillus anthracis CDC 684

MAMKNKVGIRIEDGINLASAQFKEDAYEIYKESRKVQPVLFVNKTELGAEWLITRYEDALPLLKDNRLKKDPANVFSQDTLNVFLTVDNSDYLTTHMLNSDPPNHNRLRSLVQKVFTPKMIAQLEGRIQDIADDLLNEVERKGSLNLVDDYSFPLPIIVISEMLGIPKEDQAKFRIWSHAVIAYPETPEEIKETEKQLSEFITYLQYLVDMKRKEPKEDLVSALILAESEGHKLSARELYSMIMLLIVAGHETTVNLITNTVLALLENPNQLQLLKENPKLIDAAIEEGLRYYSPVEVTTSRWADEPFQIHDQTIEKGDMVVIALAAANRDETVFENPEVFDITRENNRHIAFGHGSHFCLGAPLARLEAKIAITTLFERMPELQIKGNREDIKWQGNYLMRSLEELPLTF

>CYP107J2_ortholog(BA_2627)Bacillus anthracis Ames

MAMKNKVGIRIEDGINLASAQFKEDAYEIYKESRKVQPVLFVNKTELGAEWLITRYEDALPLLKDNRLKKDPANVFSQDTLNVFLTVDNSDYLTTHMLNSDPPNHNRLRSLVQKVFTPKMIAQLEGRIQDIADDLLNEVERKGSLNLVDDYSFPLPIIVISEMLGIPKEDQAKFRIWSHAVIAYPETPEEIKETEKQLSEFITYLQYLVDMKRKEPKEDLVSALILAESEGHKLSARELYSMIMLLIVAGHETTVNLITNTVLALLENPNQLQLLKENPKLIDAAIEEGLRYYSPVEVTTSRWADEPFQIHDQTIEKGDMVVIALAAANRDETVFENPEVFDITRENNRHIAFGHGSHFCLGAPLARLEAKIAITTLFERMPELQIKGNREDIKWQGNYLMRSLEELPLTF

>CYP107J2_ortholog(HYU01_13045)Bacillus anthracis HYU01

MAMKNKVGIRIEDGINLASAQFKEDAYEIYKESRKVQPVLFVNKTELGAEWLITRYEDALPLLKDNRLKKDPANVFSQDTLNVFLTVDNSDYLTTHMLNSDPPNHNRLRSLVQKVFTPKMIAQLEGRIQDIADDLLNEVERKGSLNLVDDYSFPLPIIVISEMLGIPKEDQAKFRIWSHAVIAYPETPEEIKETEKQLSEFITYLQYLVDMKRKEPKEDLVSALILAESEGHKLSARELYSMIMLLIVAGHETTVNLITNTVLALLENPNQLQLLKENPKLIDAAIEEGLRYYSPVEVTTSRWADEPFQIHDQTIEKGDMVVIALAAANRDETVFENPEVFDITRENNRHIAFGHGSHFCLGAPLARLEAKIAITTLFERMPELQIKGNREDIKWQGNYLMRSLEELPLTF

>CYP107J2_ortholog(BAPAT_2522)Bacillus anthracis SVA11

MAMKNKVGIRIEDGINLASAQFKEDAYEIYKESRKVQPVLFVNKTELGAEWLITRYEDALPLLKDNRLKKDPANVFSQDTLNVFLTVDNSDYLTTHMLNSDPPNHNRLRSLVQKVFTPKMIAQLEGRIQDIADDLLNEVERKGSLNLVDDYSFPLPIIVISEMLGIPKEDQAKFRIWSHAVIAYPETPEEIKETEKQLSEFITYLQYLVDMKRKEPKEDLVSALILAESEGHKLSARELYSMIMLLIVAGHETTVNLITNTVLALLENPNQLQLLKENPKLIDAAIEEGLRYYSPVEVTTSRWADEPFQIHDQTIEKGDMVVIALAAANRDETVFENPEVFDITRENNRHIAFGHGSHFCLGAPLARLEAKIAITTLFERMPELQIKGNREDIKWQGNYLMRSLEELPLTF

>CYP107J2_ortholog(A16_26640)Bacillus anthracis A16

MAMKNKVGIRIEDGINLASAQFKEDAYEIYKESRKVQPVLFVNKTELGAEWLITRYEDALPLLKDNRLKKDPANVFSQDTLNVFLTVDNSDYLTTHMLNSDPPNHNRLRSLVQKVFTPKMIAQLEGRIQDIADDLLNEVERKGSLNLVDDYSFPLPIIVISEMLGIPKEDQAKFRIWSHAVIAYPETPEEIKETEKQLSEFITYLQYLVDMKRKEPKEDLVSALILAESEGHKLSARELYSMIMLLIVAGHETTVNLITNTVLALLENPNQLQLLKENPKLIDAAIEEGLRYYSPVEVTTSRWADEPFQIHDQTIEKGDMVVIALAAANRDETVFENPEVFDITRENNRHIAFGHGSHFCLGAPLARLEAKIAITTLFERMPELQIKGNREDIKWQGNYLMRSLEELPLTF

>CYP107J2_ortholog(DJ46_1410)Bacillus anthracis Vollum

MAMKNKVGIRIEDGINLASAQFKEDAYEIYKESRKVQPVLFVNKTELGAEWLITRYEDALPLLKDNRLKKDPANVFSQDTLNVFLTVDNSDYLTTHMLNSDPPNHNRLRSLVQKVFTPKMIAQLEGRIQDIADDLLNEVERKGSLNLVDDYSFPLPIIVISEMLGIPKEDQAKFRIWSHAVIAYPETPEEIKETEKQLSEFITYLQYLVDMKRKEPKEDLVSALILAESEGHKLSARELYSMIMLLIVAGHETTVNLITNTVLALLENPNQLQLLKENPKLIDAAIEEGLRYYSPVEVTTSRWADEPFQIHDQTIEKGDMVVIALAAANRDETVFENPEVFDITRENNRHIAFGHGSHFCLGAPLARLEAKIAITTLFERMPELQIKGNREDIKWQGNYLMRSLEELPLTF

>CYP107J2_ortholog(GBAA_2627)Bacillus anthracis Ames Ancestor

MAMKNKVGIRIEDGINLASAQFKEDAYEIYKESRKVQPVLFVNKTELGAEWLITRYEDALPLLKDNRLKKDPANVFSQDTLNVFLTVDNSDYLTTHMLNSDPPNHNRLRSLVQKVFTPKMIAQLEGRIQDIADDLLNEVERKGSLNLVDDYSFPLPIIVISEMLGIPKEDQAKFRIWSHAVIAYPETPEEIKETEKQLSEFITYLQYLVDMKRKEPKEDLVSALILAESEGHKLSARELYSMIMLLIVAGHETTVNLITNTVLALLENPNQLQLLKENPKLIDAAIEEGLRYYSPVEVTTSRWADEPFQIHDQTIEKGDMVVIALAAANRDETVFENPEVFDITRENNRHIAFGHGSHFCLGAPLARLEAKIAITTLFERMPELQIKGNREDIKWQGNYLMRSLEELPLTF

>CYP107J2_ortholog(A16R_26990)Bacillus anthracis A16R

MAMKNKVGIRIEDGINLASAQFKEDAYEIYKESRKVQPVLFVNKTELGAEWLITRYEDALPLLKDNRLKKDPANVFSQDTLNVFLTVDNSDYLTTHMLNSDPPNHNRLRSLVQKVFTPKMIAQLEGRIQDIADDLLNEVERKGSLNLVDDYSFPLPIIVISEMLGIPKEDQAKFRIWSHAVIAYPETPEEIKETEKQLSEFITYLQYLVDMKRKEPKEDLVSALILAESEGHKLSARELYSMIMLLIVAGHETTVNLITNTVLALLENPNQLQLLKENPKLIDAAIEEGLRYYSPVEVTTSRWADEPFQIHDQTIEKGDMVVIALAAANRDETVFENPEVFDITRENNRHIAFGHGSHFCLGAPLARLEAKIAITTLFERMPELQIKGNREDIKWQGNYLMRSLEELPLTF

>CYP107J2_ortholog(BAS2448)Bacillus anthracis Sterne

MAMKNKVGIRIEDGINLASAQFKEDAYEIYKESRKVQPVLFVNKTELGAEWLITRYEDALPLLKDNRLKKDPANVFSQDTLNVFLTVDNSDYLTTHMLNSDPPNHNRLRSLVQKVFTPKMIAQLEGRIQDIADDLLNEVERKGSLNLVDDYSFPLPIIVISEMLGIPKEDQAKFRIWSHAVIAYPETPEEIKETEKQLSEFITYLQYLVDMKRKEPKEDLVSALILAESEGHKLSARELYSMIMLLIVAGHETTVNLITNTVLALLENPNQLQLLKENPKLIDAAIEEGLRYYSPVEVTTSRWADEPFQIHDQTIEKGDMVVIALAAANRDETVFENPEVFDITRENNRHIAFGHGSHFCLGAPLARLEAKIAITTLFERMPELQIKGNREDIKWQGNYLMRSLEELPLTF

>CYP107J2_ortholog(H9401_2502)Bacillus anthracis H9401

MAMKNKVGIRIEDGINLASAQFKEDAYEIYKESRKVQPVLFVNKTELGAEWLITRYEDALPLLKDNRLKKDPANVFSQDTLNVFLTVDNSDYLTTHMLNSDPPNHNRLRSLVQKVFTPKMIAQLEGRIQDIADDLLNEVERKGSLNLVDDYSFPLPIIVISEMLGIPKEDQAKFRIWSHAVIAYPETPEEIKETEKQLSEFITYLQYLVDMKRKEPKEDLVSALILAESEGHKLSARELYSMIMLLIVAGHETTVNLITNTVLALLENPNQLQLLKENPKLIDAAIEEGLRYYSPVEVTTSRWADEPFQIHDQTIEKGDMVVIALAAANRDETVFENPEVFDITRENNRHIAFGHGSHFCLGAPLARLEAKIAITTLFERMPELQIKGNREDIKWQGNYLMRSLEELPLTF

>CYP107J2_ortholog(BCAH820_2645)Bacillus cereus AH820

MAMKNKVGLRIEDGINLASAQFKEDAYEIYKESRKVQPVLFVNKTELGAEWLITRYEDALPLLKDNRLKKDPANVFSQDTLNVFLTVDNSDYLTTHMLNSDPPNHNRLRSLVQKVFTPKMIAQLEGRIQDIADDLLNEVERKGSLNLVDDYSFPLPIIVISEMLGIPKEDQAKFRIWSHAVIAYPETPEEIKETEKQLSEFITYLQYLVDMKRKEPKEDLVSALILAESEGHKLSARELYSMIMLLIVAGHETTVNLITNTVLALLENPNQLQLLKENPKLIDAAIEEGLRYYSPVEVTTSRWADEPFQIHDQTIEKGDMVVIALASANRDETVFENPEVFDITRENNRHIAFGHGSHFCLGAPLARLEAKIAITTLFERMPELQIKGNREDIKWQGNYLMRSLEELPLTF

>CYP107J2_ortholog(BF38_3807)Bacillus thuringiensis HD1011

MAMKNKVGLRIEDGINLASAQFKEDAYEIYKESRKVQPVLFVNKTELGAEWLITRYEDALPLLKDNRLKKDPANVFSQDTLNVFLTVDNSDYLTTHMLNSDPPNHNRLRSLVQKVFTPKMIAQLEGRIQDIADDLLNEVERKGSLNLVDDYSFPLPIIVISEMLGIPKEDQAKFRIWSHAVIAYPETPEEIKETEKQLSEFITYLQYLVDMKRKEPKEDLVSALILAESEGHKLSARELYSMIMLLIVAGHETTVNLITNTVLALLENPNQLQLLKENPKLIDAAIEEGLRYYSPVEVTTSRWADEPFQIHDQTIEKGDMVVIALASANRDETVFENPEVFDITRENNRHIAFGHGSHFCLGAPLARLEAKIAITTLFERMPELQIKGNREDIKWQGNYLMRSLEELPLTF

>CYP107J2_ortholog(BCA_2718)Bacillus cereus 03BB102

MAMKNKVGLRIEDGINLASAQFKEDAYEIYKESRKVQPVLFVNKTELGAEWLITRYEDALPLLKDSRLKKDPANVFSQDTLNVFLTVDNGDHLTTHMLNSDPPNHNRLRSLVQKVFTPKMIAQLEGRIQDIADDLLNEVERKGSLNLVDDYSFPLPIIVISEMLGIPKEDQAKFRIWSHAVIAYPETPEEIKETEKQLSEFITYLQYLVDMKRKEPKEDLVSALILAESEGHKLSARELYSMIMLLIVAGHETTVNLITNTVLALLENPNQLQLLKENPKLIDAAIEEGLRYYSPVEVTTSRWADEPFQIHDQTIEKGDMVVIALASANRDETVFENPEVFDITRENNRHIAFGHGSHFCLGAPLARLEAKIAITTLFERMPELQIKGNREDIKWQGNYLMRSLEELPLTF

>CYP107J2_ortholog(BALH_2362)Bacillus thuringiensis Al Hakam

MAMKNKVGLRIEDGINLASAQFKEDAYEIYKESRKVQPVLFVNKTELGAEWLITRYEDALPLLKDSRLKKDPANVFSQDTLNVFLTVDNGDHLTTHMLNSDPPNHNRLRSLVQKVFTPKMIAQLEGRIQDIADDLLNEVERKGSLNLVDDYSFPLPIIVISEMLGIPKEDQAKFRIWSHAVIAYPETPEEIKETEKQLSEFITYLQYLVDMKRKEPKEDLVSALILAESEGHKLSARELYSMIMLLIVAGHETTVNLITNTVLALLENPNQLQLLKENPKLIDAAIEEGLRYYSPVEVTTSRWADEPFQIHDQTIEKGDMVVIALASANRDETVFENPEVFDITRENNRHIAFGHGSHFCLGAPLARLEAKIAITTLFERMPELQIKGNREDIKWQGNYLMRSLEELPLTF

>CYP107J2_ortholog(BT9727_2410)Bacillus thuringiensis HD-771

MAMKNKVGLRIEDGINLASAQFKEDAYEIYKESRKVQPVLFVNKTELGAEWLITRYEDALPLLKDSRLKKDPANVFSQDTLNVFLTVDNGDHLTTHMLNSDPPNHNRLRSLVQKVFTPKMIAQLEGRIQDITDDLLNEVERKGSLNLVDDYSFPLPIIVISEMLGIPKEDQAKFRIWSHAVIAYPETPEEIKETEKQLSEFITYLQYLVDMKRKEPKEDLVSALILAESEGHKLSARELYSMIMLLIVAGHETTVNLITNTVLALLENPNQLQLLKENPKLIDAAIEEGLRYYSPVEVTTSRWADEPFQIHDQTIEKGDMVVIALASANRDETVFENPEVFDITRENNRHIAFGHGSHFCLGAPLARLEAKIAITTLFERMPELQIKGNREDIKWQGNYLMRSLEELPLTF

>CYP107J2_ortholog(BCE33L2373)Bacillus cereus E33L

MAMKNKVGLRIEDGINLASAQFKEDAYEIYKESRKVQPVLFVNKTELGAEWLITRYEDALPLLKDSRLKKDPANVFSQDTLNVFLTVDNGDHLTTHMLNSDPPNHNRLRSLVQKVFTPKMIAQLEGRIQDITDDLLNEVERKGSLNLVDDYSFPLPIIVISEMLGIPKEDQAKFRIWSHAVIAYPETPEEIKETEKQLSEFITYLQYLVDMKRKEPTEDLVSALILAESEGHKLSARELYSMIMLLIVAGHETTVNLITNTVLALLENPNQLQLLKENPKLIDAAIEEGLRYYSPVEVTTSRWADEPFQIHDQTIEKGDMVVIALASANRDETVFENPEVFDITRENNRHIAFGHGSHFCLGAPLARLEAKIAITTLFERMPELQIKGNREDIKWQGNYLMRSLEELPLTF

>CYP107J2_ortholog(BACI_c26020)Bacillus cereus biovar anthracis CI

MSMKNKVGLRIEDGINLASAQFKEDAYEIYKESRKMQPILFVNKTELGAEWLITRYEDALPLLKDNRLKKDPANVFSQDTLNVFLTVDNSDYLTTHMLNSDPPNHNRLRSLVQKAFTPKMIAQLEGRIQHIADDLLNEVERKGSLNLVDDYSFPLPIIVISEMLGIPKEDQAKFRIWSHAVIAYPETPEEIKETEKQLSEFITYLQYLVDMKRKEPKEDLVSALILAESEGHKLSARELYSMIMLLIVAGHETTVNLITNTVLALLENPNQLRLLKENPKLIDAAIEEGLRYYSPVEVTTSRWADEPFQIHDQTIEKGDMVVIALASANRDETVFENPEVFDITRENNRHIAFGHGSHFCLGAPLARLEAKIAITTLFERMPELQIKGNREDIKWQGNYLMRSLEELPLTF

>CYP107J2_ortholog(BCG9842_B2673)Bacillus cereus G9842

MSMKNKVGLKIEEGINLASAQFKEDAYEIYKESRKMQPILFVNKTELGAEWLITRYEDALPLLKDSRLKKDPANVFSQDTLNVFLTVDNSDYLTTHMLNSDPPNHNRLRSLVQKAFTPKMITQLEGRIQDIADDLLNEVERKGSLNLVDDYSFPLPIIVISEMLGIPKEDQAKFRIWSHAVIAYPETPEEIKETEKQLSEFITYLQYLVDIKRKEPKEDLVSALILAESEGHKLSARELYSMIMLLIVAGHETTVNLITNTVLALLENPDQLQLLKENPKLIDAAIEEGLRYYSPVEVTTSRWADEPFQIHDQTIEKGDMVVIALASANRDETVFENPEVFDITRENNRHIAFGHGSHFCLGAPLARLEAKIAITTLFNRMPELQIKGNREEIKWQGNYLMRSLEELPLTF

>CYP107J2_ortholog(BTG_06625)Bacillus thuringiensis HD-771

MSMKNKVGLKIEEGINLASAQFKEDAYEIYKESRKMQPILFVNKTELGAEWLITRYEDALPLLKDSRLKKDPANVFSQDTLNVFLTVDNSDYLTTHMLNSDPPNHNRLRSLVQKAFTPKMITQLEGRIQNIADDLLNEVERKGSLNLVDDYSFPLPIIVISEMLGIPKEDQAKFRIWSHAVIAYPETPEEIKETEKQLSEFITYLQYLVDIKRKEPKEDLVSALILAESEGHKLSARELYSMIMLLIVAGHETTVNLITNTVLALLENPDQLQLLKENPKLIDAAIEEGLRYYSPVEVTTSRWADEPFQIHDQTIEKGDMVVIALASANRDETVFENPEVFDITRENNRHIAFGHGSHFCLGAPLARLEAKIAITTLFNRMPELQIKGNREEIKWQGNYLMRSLEELPLTF

>CYP107J2_ortholog(BTF1_10335)Bacillus thuringiensis HD-789

MSMKNKVGLKIEEGINLASAQFKEDAYEIYKESRKMQPILFVNKTELGAEWLITRYEDALPLLKDSRLKKDPANVFSQDTLNVFLTVDNSDYLTTHMLNSDPPNHNRLRSLVQKAFTPKMITQLEGRIQDIAGDLLNEVERKGSLNLVDDYSFPLPIIVISEMLGIPKEDQAKFRIWSHAVIAYPETPEEIKETEKQLSEFITYLQYLVDIKRKEPKEDLVSALILAESEGHKLSARELYSMIMLLIVAGHETTVNLITNTVLALLENPDQLQLLKENPKLIDAAIEEGLRYYSPVEVTTSRWADEPFQIHDQTIEKGDMVVIALASANRDETVFENPEVFDITRENNRHIAFGHGSHFCLGAPLARLEAKIAITTLFNRMPELQIKGNREEIKWQGNYLMRSLEELPLTF

>CYP107J2_ortholog(YBT020_13185)Bacillus thuringiensis serovar finitimus YBT-020

MSMKNKVGLRIEDGINLASAQFKEDAYEIYKESRKMQPILFVNKTELGAEWLITRYEDALPLLKDNRLKKDPANVFSQDTLNVFLTVDNSDYLTTHMLNSDPPNHNRLRSLVQKAFTPKMIAQLEGRIRDIADDLLNEVERKGSLNLVDDYSFPLPIIVISEMLGIPKEDQAKFRIWSHAVIAYPETPEEIKETEKQLSEFITYLQYLVDVKRQEPKEDLVSALILAESEGHKLSARELYSMIMLLIVAGHETTVNLITNTVLALLENPDQLQLLKENPKLIDVAIEEGLRYYSPVEVTTSRWADEPFQIHDRTIEKGDMVVIALASANRDETVFENPEVFDITRENNRHIAFGHGSHFCLGAPLARLEAKIAITTLFNRMPDLQIKGSREEIKWQGNYLMRSLEELPLTF

>CYP107J2_ortholog(BCQ_2494)Bacillus cereus Q1

MKNKVGLRIENGINLASAQFKEDAYEIYKESRKMQPILFVNKTELGAEWLITRYEDALPLLKDNRLKKDPANVFSQDTLNVFLTVDNSDYLTTHMLNSDPPNHNRLRSLVQKAFTPKMIAQLEGRIQHIADDLLNEVERKDSLNLVDDYSFPLPIIVISEMLGIPKEDQAKFRIWSHAVIAYPETPEEIKETEKQLSEFITYLQYLVDMKRKEPKEDLVSALILAESEGHKLNARELYSMIMLLIVAGHETTVNLITNTVLALLENPDQLQLLKENPKLIDAAIEEGLRYYSPVEVTTSRWADEPFQIHDQTIEKGDMVVIALASANRDETVFENPEVYDITRENNRHIAFGHGSHFCLGAPLAKLEAKIAITTLFNRMPKLQIKGDREEIKWQGNYLMRSLEELPLTF

>CYP107J2_ortholog(BCAH187_A2696)Bacillus cereus AH820

MSMKNKVGLRIENGINLASAQFKEDAYEIYKESRKMQPILFVNKTELGAEWLITRYEDALPLLKDNRLKKDPANVFSQDTLNVFLTVDNSDYLTTHMLNSDPPNHNRLRSLVQKAFTPKMIAQLEGRIQHIADDLLNEVERKDSLNLVDDYSFPLPIIVISEMLGIPKEDQAKFRIWSHAVIAYPETPEEIKETEKQLSEFITYLQYLVDMKRKEPKEDLVSALILAESEGHKLNARELYSMIMLLIVAGHETTVNLITNTVLALLENPDQLQLLKENPKLIDAAIEEGLRYYSPVEVTTSRWADEPFQIHDQTIEKGDMVVIALASANRDETVFENPEVYDITRENNRHIAFGHGSHFCLGAPLAKLEAKIAITTLFNRMPKLQIKGDREEIKWQGNYLMRSLEELPLTF

>CYP107J2_ortholog(BCN_2509)Bacillus cereus NC7401

MSMKNKVGLRIENGINLASAQFKEDAYEIYKESRKMQPILFVNKTELGAEWLITRYEDALPLLKDNRLKKDPANVFSQDTLNVFLTVDNSDYLTTHMLNSDPPNHNRLRSLVQKAFTPKMIAQLEGRIQHIADDLLNEVERKDSLNLVDDYSFPLPIIVISEMLGIPKEDQAKFRIWSHAVIAYPETPEEIKETEKQLSEFITYLQYLVDMKRKEPKEDLVSALILAESEGHKLNARELYSMIMLLIVAGHETTVNLITNTVLALLENPDQLQLLKENPKLIDAAIEEGLRYYSPVEVTTSRWADEPFQIHDQTIEKGDMVVIALASANRDETVFENPEVYDITRENNRHIAFGHGSHFCLGAPLAKLEAKIAITTLFNRMPKLQIKGDREEIKWQGNYLMRSLEELPLTF

>CYP107J3_ortholog(BC2609)Bacillus cereus ATCC 14579

MKNKVGLSIEDGINLASAQFKEDAYEIYKESRKKQPILFVNQVEIGKEWLITRYEDALPLLKDNRLKKDWTNVFSQDIKNMYLSVDNSDHLTTHMLNSDPPNHSRLRSLVQKAFTPKMIAQLDGRIQRIADDLISDIERKGTLNLVDDYSFPLPIIVISEMLGIPKEDQAKFRIWSHAVIASPETPEEIKETEKQLSEFITYLQYLVDIKRKEPKEDLVSALILAESEGHKLSARELYSMIMLLIVAGHETTVNLITNTVLALLENPNQLQLLKDNPKLIDSAIEEGLRYYSPVEVTTARWAAEPFQIHHQTIQKGDMVIIALASANRDETVFENPEIFDITRENNRHIAFGHGSHFCLGAPLARLEAKIAITTLFNRMPELQIKGNREEIKWQGNYLMRSLEELPLTF

>CYP107J3_ortholog(CT43_CH2610)Bacillus thuringiensis serovar chinensis CT-43

MSMKNKVGLSIEDGINLASAQFKEDAYEIYKESRKKQPILFVNQVEIGKEWLITRYEDALPLLKDNRLKKDWTNVFSQDIKNMYLSVDNSDHLTTHMLNSDPPNHSRLRSLVQKAFTPKMIAQLDGRIERIADDLISDIERKGTLNLVDDYSFPLPIIVISEMLGIPKEDQAKFRIWSHAVIASPETPEEIKETEKQLSEFITYLQYLVDIKRKEPKEDLVSALILAESEGHKLSARELYSMIMLLIVAGHETTVNLITNTVLALLENPNQLQLLKDNPKLIDSAIEEGLRYYSPVEVTTARWAAEPFQIHHQTIQKGDMVIIALASANRDETVFENPEIFDITRENNRHIAFGHGSHFCLGAPLARLEAKIAITTLFNRMPELKIKGNREEIKWQGNYLMRSLEELPLTF

>CYP107J3_ortholog(BTB_c27360)Bacillus thuringiensis Bt407

MSMKNKVGLSIEDGINLASAQFKEDAYEIYKESRKKQPILFVNQVEIGKEWLITRYEDALPLLKDNRLKKDWTNVFSQDIKNMYLSVDNSDHLTTHMLNSDPPNHSRLRSLVQKAFTPKMIAQLDGRIERIADDLISDIERKGTLNLVDDYSFPLPIIVISEMLGIPKEDQAKFRIWSHAVIASPETPEEIKETEKQLSEFITYLQYLVDIKRKEPKEDLVSALILAESEGHKLSARELYSMIMLLIVAGHETTVNLITNTVLALLENPNQLQLLKDNPKLIDSAIEEGLRYYSPVEVTTARWAAEPFQIHHQTIQKGDMVIIALASANRDETVFENPEIFDITRENNRHIAFGHGSHFCLGAPLARLEAKIAITTLFNRMPELKIKGNREEIKWQGNYLMRSLEELPLTF

>CYP107J3_ortholog(H175_ch2654)Bacillus thuringiensis serovar thuringiensis IS5056

MSMKNKVGLSIEDGINLASAQFKEDAYEIYKESRKKQPILFVNQVEIGKEWLITRYEDALPLLKDNRLKKDWTNVFSQDIKNMYLSVDNSDHLTTHMLNSDPPNHSRLRSLVQKAFTPKMIAQLDGRIERIADDLISDIERKGTLNLVDDYSFPLPIIVISEMLGIPKEDQAKFRIWSHAVIASPETPEEIKETEKQLSEFITYLQYLVDIKRKEPKEDLVSALILAESEGHKLSARELYSMIMLLIVAGHETTVNLITNTVLALLENPNQLQLLKDNPKLIDSAIEEGLRYYSPVEVTTARWAAEPFQIHHQTIQKGDMVIIALASANRDETVFENPEIFDITRENNRHIAFGHGSHFCLGAPLARLEAKIAITTLFNRMPELKIKGNREEIKWQGNYLMRSLEELPLTF

>CYP107J3_ortholog(YBT1518_14460)Bacillus thuringiensis YBT-1518

MSMKNKVGLSIEDGINLASAQFKEDAYEIYKESRKKQPILFVNQVEIGKEWLITRYEDALPLLKDNRLKKDWTNVFSQDIKNMYLSVDNSDHLTTHMLNSDPPNHSRLRSLVQKAFTPKMIAQLDGRIERIADDLISDIERKGTLNLVDDYSFPLPIIVISEMLGIPKEDQAKFRIWSHAVIASPETPEEIKETEKQLSEFITYLQYLVDIKRKEPKEDLVSALILAESEGHKLSARELYSMIMLLIVAGHETTVNLITNTVLALLENPNQLQLLKDNPKLIDSAIEEGLRYYSPVEVTTARWAAEPFQIHHQTIQKGDMVIIALASANRDETVFENPEIFDITRENNRHIAFGHGSHFCLGAPLARLEAKIAITTLFNRMPELKIKGNREEIKWQGNYLMRSLEELPLTF

>CYP107J3_ortholog(BMB171_C2365) Bacillus thuringiensis BMB171

MSMKNKVGLSIEDGINLASAQFKEDAYEIYKESRKKQPILFVNQVEIGKEWIITRYEDALPLLKDNRLKKDWTNVFSQDTKNMYLSVDNSDHLTTHMLNSDPPNHSRLRSLVQKAFTPKMIAQLDGRIQRIADDLISNIERKGTLNLVDDYSFPLPIIVISEMLGIPKEDQAKFRIWSHAVIASPETPEEIKETEKQLSEFITYLQYLVDIKRKEPKEDLVSALILAESEGHKLSARELYSMIMLLIVAGHETTVNLITNTVLALLENPNQLQLLKDNPKLIDSAIEEGLRYYSPVEVTTARWAAEPFQIHHQTIQKGDMVIIALASANRDETVFENPEIFDITRENNRHIAFGHGSHFCLGAPLARLEAKIAITTLFNRMPELQIKGNREEIKWQGNYLMRSLEELPLTF

>CYP107J3_ortholog(BCE_2654)Bacillus cereus ATCC 10987

MSMKNKVGLSIEDGINLASAQFKEDAYEIYKESRKKQPILFVNQVEIGKEWLITRYEDALPLLKDNRLKKDWTNVFSQDTKNMYLSVDNSDHLTTHMLNSDPPNHSRLRSLVQKAFTPKMIAQLDGRIQRIADDLISDIERKGTLNLVDDYSFPLPIIVISEMLGIPKEDQAKFRIWSHAVIASPETPEEIKETEKQLSEFITYLQYLVDVKRKEPKEDLVSALILAESEGHKLSARELYSMIMLLIVAGHETTVNLITNTVLALLENPNQLQLLKDNPKLIDSAIEEGLRYYSPVEVTTARWAAEPFQIHHQTIQKGDMVIIALASANRDETVFENPEIFDITRENNRHIAFGHGSHFCLGAPLARLEAKIAITTLFKRMPELQIKGDRKDIKWQGNYLMRSLEELPLTF

>CYP107J3_ortholog(CY96_11885)Bacillus bombysepticus

MSMKNKVGLSIEDGINLASAQFKEDAYEIYKESRKKQPILFVNQVEIGKEWLITRYEDALPLLKDNRLKKDWTNVFSQDIKNMYLSVDNSDHLTTHMLNSDPPNHSRLRSLVQKAFTPKMIAQLDKRIERIADELISDIERKGTLNLVDDYSFPLPIIVISEMLGIPKEDQAKFRIWSHAVIASPETPEEIKETEKQLSEFITYLQYLVDIKRKEPKEDLVSALILAESEGHKLSARELYSMIMLLIVAGHETTVNLITNTVLALLENPNQLQLLKDNPKLIDSAIEEGLRYYSPVEVTTARWAAEPFQIHDRTIEKGDMVVIALASANRDETVFENPEVFDITRENNRHIAFGHGSHFCLGAPLARLEAKIAITTLFNRMPELQIKGNREEIKWQGNYLMRSLEELPLTF

>CYP107J3_ortholog(BCB4264_A2651)Bacillus cereus B4264

MSMKNKVGLSIEDGINLASAQFKEDAYEIYKESRKKQPILFVNQVEIGKEWLITRYEDALPLLKDNRLKKDWTNVFSQDIKNMYLSVDNSDHLTTHMLNSDPPNHSRLRSLVQKAFTPKMIAQLDKRIEKIADDLISDIERKGTLNLVDDYSFPLPIIVISEMLGIPKEDQAKFRIWSHAVIASPETPEEIKETEKQLSEFITYLQYLVDIKRKEPKEDLVSALILAESEGHKLSARELYSMIMLLIVAGHETTVNLITNTVLALLENPNQLQLLKDNPKLIDSAIEEGLRYYSPVEVTTARWAAEPFQIHDRTIEKGDMVVIALASANRDETVFENPEVFDITRENNRHIAFGHGSHFCLGAPLARLEAKIAITTLFNRMPELQIKGNREEIKWQGNYLMRSLEELPLTF

>CYP107J3_ortholog(BCK_21710)Bacillus cereus FRI-35

MSMKNKVGLSIEDGINLASAQFKEDAYEIYKESRKKQPILFVNQVEIGKEWLITRYEDALPLLKDNRLKKDWKNVFSQDTKNMYLSVDNSDHLTTHMLNSDPPNHSRLRSLVQKAFTPKMIAQLDGRIQRIADDLISDIERKGTLNLVDDYSFPLPIIVISEMLGIPKEDQAKFRIWSHAVIATPETPEEIKETEKQLSEFITYLQYLVDVKRKEPKEELVSALILAESEGHKLSARELYSMIMLLIVAGHETTVNLITNTVLALLENPNQLQLLKDNPKLIDSAIEEGLRYYSPVEVTTARWAAEPFQIHHQTIQKGDMVIIALASANRDETVFENPEIFDITRENNRHIAFGHGSHFCLGAPLARLEAKIAITTLFNRMPELQIKGDRKDIKWQGNYLMRSLEELPLTF

>CYP107J3_ortholog(YBT1520_16670)Bacillus thuringiensis serovar kurstaki YBT-1520

MSMKNKVGLSIEDGINLASAQFKEDAYEIYKESRKKQPILFVNQVEIGKEWLITRYEDALPLLKDNRLKKDWTNVFSQDIKNMYLSVDNSDHLTTHMLNSDPPNHSRLRSLVQKAFTPKMIAQLDKRIEKIADDLISDIERKGTLNLVDDYSFPLPIIVISEMLGIPKEDQAKFRIWSHAVIASPETPEEIKETEKQLSEFITYLQYLVDIKRKEPKEDLVSALILAESEGHKLSARELYSMIMLLIVAGHETTVNLITNTVLALLENPNQLQLLKDNPKLIDSAIEEGLRYYSPVEVTTARWAAEPFQIHDRTIEKGDMVVIALASANRDETVFENPEVFDITRENNRHIAFGHGSHFCLGAPLARLEAKIAITTLFNRMPELQIKGNREEIKWQGNYLMRSLEELPLTF

>CYP107J3_ortholog(AQ980_14330)Bacillus thuringiensis YWC2-8

MSMKNKVGLSIEDGINLASAQFKEDAYEIYKESRKKQPILFVNQVEIGKEWLITRYEDALPLLKDNRLKKDWTNVFSQDIKNMYLSVDNSDHLTTHMLNSDPPNHSRLRSLVQKAFTPKMIAQLDKRIEKIADDLISDIERKGTLNLVDDYSFPLPIIVISEMLGIPKEDQAKFRIWSHAVIASPETPEEIKETEKQLSEFITYLQYLVDIKRKEPKEDLVSALILAESEGHKLSARELYSMIMLLIVAGHETTVNLITNTVLALLENPNQLQLLKDNPKLIDSAIEEGLRYYSPVEVTTARWAAEPFQIHDRTIEKGDMVVIALASANRDETVFENPEVFDITRENNRHIAFGHGSHFCLGAPLARLEAKIAITTLFNRMPELQIKGNREEIKWQGNYLMRSLEELPLTF

>CYP107J3_ortholog(MC28_1830)Bacillus thuringiensis MC28

MSMKNKVGLRIEDGINLASAQFKKDAYEIYKESRKVQPILFVNQIEIGKEWLITRYEDALPLLKDNRLKKDQANVFPQDTKNMYLSVDNSDHLTTHMLNSDPPNHSRLRSLVQKAFTPKMISQLDGRIQRIADDLISEIERKGTLNLVDDYSFPLPIIVISEMLGIPKEDQAKFRIWSHAVIASPETPEEIKETEKQLSEFITYLQYIVDVKRKNPKEDLVSALILAENEGQKLSARELYSMIMLLIVAGHETTVNLITNTVLALLENPKQLQLLKDNPKLIDSAIEEGLRYYSPVEVTTARWAAEPFQIHDQTIQKGDMVIIALASANRDETVFENPEVFDIMRENNRHIAFGHGSHFCLGAPLARLEAKIAITTLFKRMPELQIKGDREDIKWQGNYLMRSLEELPLTF

>CYP107J3_ortholog(BG05_3311) Bacillus mycoides ATCC 6462

MSIKNKVGRKIEDGINLASAQFKEDAYEIYKESRKMQPILFVNEVEIGKEWLITRYEDALPLLKDNRLKKDMANVFSQDTKNMYLSVDNSDHLTTHMLNSDPPNHSRLRSLVQKAFTPKMISQLDGRIQRVADDLINEIERKGTLNLVDDYSFPLPIIVISEMLGIPKEDQAKFRIWSHAVIASPETPEEIKETEKQLSEFITYLQYIVDVKRKNPKEDLVSALILAENEGHKLSARELYSMIMLLIVAGHETTVNLITNTVLALLENPKQLQLLKENPELIDSAIEEGLRYYSPVEVTTARWAAEPFQIHDQTIQKGDMVIIALASANRDETVFENPEVFDITRENNRHIAFGHGSHFCLGAPLARLEAKIAITTLFKRMPSLQIKGEREKINWQGNYLMRSLEELPLSF

>CYP107J3_ortholog(BcerKBAB4_2513)Bacillus mycoides KBAB4

MSIKNKVGRKIEDGINLASAQFKEDAYEIYKESRKVQPILFVNQIEIGKEWLITRYEDALPLLKDNRLKKDQANVFPQDTKNMHLSVDNSDHLTTHMLNSDPPNHSRLRSLVQKAFTPKMISQLDGRIQRIADDLISEIERKGTLNLVDDYSFPLPIIVISEMLGIPKEDQAKFRIWSHAVIASPETPEEVKETEKQLSEFITYLQYIVDVKRKNPKEDLVSALILAENEGHKLSARELYSMIMLLIVAGHETTVNLITNTVLALLENPKQLQLLKENPELIDSAIEEGLRYYSPVEVTTARWAAEPFQIHDQTIQKGDMVIIALASANRDETVFENPEVFDIMRENNRHIAFGHGSHFCLGAPLARLEAKIAITALFKRMPSLQIKGEREKINWQGNYLMRSLEELPLSF

>CYP107J3_ortholog(bwei_2316)Bacillus mycoides WSBC 10204

MSIKNKVGQKIEDGINLASAQFKEDAYEIYKESRKVQPILFVNQIEIGKEWLITRYEDALPLLKDNRLKKDQANVFPQDTKNMHLFVDNSDHLTTHMLNSDPPNHSRLRSLVQKAFTPKMISQLDGRIQRIADDLISEIERKGTLNLVDDYSFPLPIIVISEMLGIPKEDQAKFRIWSHAVIASPETPEEIKETEKQLSEFITYLQYIVDVKRKNPKEDLVSALILAENEGHKLSARELYSMIMLLIVAGHETTVNLITNTVLALLENPKQLQLLKENPELIDSAIEEGLRYYSPVEVTTARWAAEPFQIHDQTIQKGDMVIIALASANRDETVFENPEVFDIMRENNRHIAFGHGSHFCLGAPLARLEAKIAITALFKRMPSLQIKGERDKINWQGNYLMRSLEELPLSF

>CYP107J5(GYO_2281)Bacillus subtilis subsp. spizizenii TU-B-10

MSSKQKKSLTILTESQLCSSAFKDEAYDFYKESRKSHPLYPLSLGKLGEGWLISRYDDAIHLLKNEKLKKNYENVITAKEEKRPVLLKNEEPLTKHMLNSDPPDHNRLRTLVQKAFTHRMILQLEDKIQHIADSLLDKVQPNKFMNLVDDYAFPLPIIVISEMLGIPLEDRQRFRVWSQAIIDFSDAPERLQENDHLLGEFVEYLEYLVRIKRSEPAGDLISVLIQVESEGTQLSTEELYSMIMLLIVAGHETTVNLITNMTYALMCHHDQLEKLRQQPGLINSAIEEALRFHSPVELTTIRWTAEPFILHGQEIKRKDVIIISLASANRDEKIFSNADIFDIERKNNRHIAFGHGSHFCLGAQLARLESKIAISTLLRRCPNIQIKGEKEQIKWKGNFLMRALEELPLSF

>CYP107J6(BAMTA208_08360)Bacillus amyloliquefaciens TA208

MFSKQNESLTVLTESQLSSRAFKDEAYAFYKELRKSRPLCPLSLGSLGEGWLISRYDDAIQLLKNEKLKKNYENVFTAKEEKRPVLFKNEGPLRKHMLNSDPPDHNRLRTLVQKAFTHRMILQLEDKIQHIADSLLDKVQPSKFMNLVDDYAFPLPIIVISEMLGIPLEDRQNFKIWSQAIIDFSDAPERLQENDHLLGEFVEYLEYLVRKKRSEPAGDLISALIQAESEGTPLSTEELYSMIMLLIVAGHETTVNLITNMTYALMCHHDQLEKLRRQPDLMNSAIEEALRFHSPVELTTIRWTAEPFILHGQEIKRKDVIIISLASANRDEKIFPNADMFDIERKNNRHIAFGHGIHFCLGAQLARLESKIAISTLLRRCPNIQIKGGKEQIKWKGNFLMRALEELPLTF

>CYP107J6_ortholog(BAXH7_01705)Bacillus amyloliquefaciens XH7

MFSKQNESLTVLTESQLSSRAFKDEAYAFYKELRKSRPLCPLSLGSLGEGWLISRYDDAIQLLKNEKLKKNYENVFTAKEEKRPVLFKNEGPLRKHMLNSDPPDHNRLRTLVQKAFTHRMILQLEDKIQHIADSLLDKVQPSKFMNLVDDYAFPLPIIVISEMLGIPLEDRQNFKIWSQAIIDFSDAPERLQENDHLLGEFVEYLEYLVRKKRSEPAGDLISALIQAESEGTPLSTEELYSMIMLLIVAGHETTVNLITNMTYALMCHHDQLEKLRRQPDLMNSAIEEALRFHSPVELTTIRWTAEPFILHGQEIKRKDVIIISLASANRDEKIFPNADMFDIERKNNRHIAFGHGIHFCLGAQLARLESKIAISTLLRRCPNIQIKGGKEQIKWKGNFLMRALEELPLTF

>CYP107J6_ortholog(LL3_01914)Bacillus amyloliquefaciens LL3

MFSKQNESLTVLTESQLSSRAFKDEAYAFYKELRKSRPLCPLSLGSLGEGWLISRYDDAIQLLKNEKLKKNYENVFTAKEEKRPVLFKNEGPLRKHMLNSDPPDHNRLRTLVQKAFTHRMILQLEDKIQHIADSLLDKVQPSKFMNLVDDYAFPLPIIVISEMLGIPLEDRQNFKIWSQAIIDFSDAPERLQENDHLLGEFVEYLEYLVRKKRSEPAGDLISALIQAESEGTPLSTEELYSMIMLLIVAGHETTVNLITNMTYALMCHHDQLEKLLRQPDLMNSAIEEALRFHSPVELTTIRWTAEPFILHGQEIKRKDVIIISLASANRDEKIFPNADMFDIERKNNRHIAFGHGIHFCLGAQLARLESKIAISTLLRRCPNIQIKGGKEQIKWKGNFLMRALEELPLTF

>CYP107J7(DJ92_5192) Bacillus pseudomycoides 219298

MFFKNKVGLKIEDGIQLASAGFKEDAYEIYKESRASQPILFVYKSELWTEWLITRYEDALPLLKDSRLKKNPENVFSQERLKPFLSLENSDRLTKHMLNSDPPDHSRLRALVQKAFTPKMISQLDERIQNIADTLLDRVEYKHSLNLVNDYAFPLPINVISEMLGVPKDDQERFRIWSHAVIASPETPEEIKENEEKLSEFITYLQYLVDVKRKEPKEDLVSGLIQVESEGSKLSAPELYSMIMLLIVAGHETTVNLITNTVLALLENPDQLQLLKEKPQLIDSAIEEGLRYYSPVEITTARWATEPFMIHDQEIQKGDMVIISLASANRDENVFVNPDVFDITRENNRHIAFGHGSHFCLGAPLARLEAKIAIATLLKRIPKIQIKGEREKIKWQGNYLMRSLEELPLTF

>CYP107J8(Bcer98_1885)Bacillus cytotoxicus

MSVKNKVGRNIKDGIKLASPEFKEYAYEIYKESRKLQPILFVYKGELGTEWLITRYEDALSLLKDSRLKKNPENIFSQEKLKSLFSIENSDYLTKHMLNADPPDHNRLRALVQKAFTPKMISQLHNRIQHIADTLLDHVEQKRSFHFIRDFSFPLPIIVISEMLGIPKEDQAKFRIWSHAVIDSPEIPEEKIENEEKLSEFITYLQYLVDLKRKEPKEDLISRLIQAESEGGQLSAAELYSMIMLLIVAGHETTVNLMTNTVLALLENPDQLQLLKERPELIDSAIEEGLRYYSPVEVTTARWAAEPFHIHDKVIQKGDMVTISLASANRDETVFVNPDVFNITRENNRHIAFGHGGHFCLGAPLARLEAKIAIATLLKRMPRLRIKGKREDIKWEGNYLMRSLKELPLTF

>CYP107J9(BLi02822)Bacillus licheniformis DSM 13 = ATCC 14580

MSSKQEESFAILTERELSSAAFKDEAYEFYKRLRASRPVCPVSMGELGEGWLITRYDDAVHILKDARVKKNYENAFTEEELENFSALENEEPLSKHMLNADPPDHGRLRSLVQKAFTPRMVLQLENRIQKIADSLLDQVEPNHSMNLVDDFAFPLPIIVISEMLGIPLEDRQKFRVWSQAVIDFSDTPESLEEYKYKIGEFAEYLEYLVRKKRDEPAEDLVSALIQAESEGTKLSIEELYATIMLLIVAGHETTVNLITNMTLALLNHPEQLEKLRQNADLIDSAIEEALRFYSPVELTTLRWAAEPFTLHGQEIKRKDVVIISLASANRDYMVFSNADRFDIERKDNRHLAFGHGSHFCLGAPLARLEAKIAIQTLLRRFEHIEIKGEREQIKWKGNFLMRALEELPLSF

>CYP107J9_ortholog(BL02429)Bacillus licheniformis ATCC 14580

MSSKQEESFAILTERELSSAAFKDEAYEFYKRLRASRPVCPVSMGELGEGWLITRYDDAVHILKDARVKKNYENAFTEEELENFSALENEEPLSKHMLNADPPDHGRLRSLVQKAFTPRMVLQLENRIQKIADSLLDQVEPNHSMNLVDDFAFPLPIIVISEMLGIPLEDRQKFRVWSQAVIDFSDTPESLEEYKYKIGEFAEYLEYLVRKKRDEPAEDLVSALIQAESEGTKLSIEELYATIMLLIVAGHETTVNLITNMTLALLNHPEQLEKLRQNADLIDSAIEEALRFYSPVELTTLRWAAEPFTLHGQEIKRKDVVIISLASANRDYMVFSNADRFDIERKDNRHLAFGHGSHFCLGAPLARLEAKIAIQTLLRRFEHIEIKGEREQIKWKGNFLMRALEELPLSF

>CYP107J9_ortholog(BaLi_c29170)Bacillus paralicheniformis

MSSKQEESFAILTERELSSAAFKDEAYEFYKRLRASRPVYPVSMGDLGEGWLITRYEDAVHILKDARMKKNYENVFTEEELENFSALENEEPLSKHMLNSDPPDHGRLRSLVQKAFTPRMVLQLENRIQKIADSLLDQAEPNHSMNLVDDFAFPLPIIVISEMLGIPLEDRQKFRVWSQAVIDFSDTPESLEEYKHKIGEFAEYLEYLVCKKRDEPAEDLVSALIQAESEGTTLSIEELYSTIMLLIVAGHETTVNLITNMTFALLNHPEQLEKLHQNADLIDSAIEEALRFHSPVELTTLRWAAEPFTLHGQEMKRKDVIIISLASANRDDTVFPNADRFNIERKDNRHLAFGYGSHFCLGAPLARLEAKIAIQTLLRRFEHIEIKGEREQIKWKGNFLMRALDELPLSF

>CYP107J10(BGLY_3122 )Bacillus glycinifermentans

MSSKHEKSSAVLSEIDLSSAAFKHEAYDFYKELRASRPLYPVSLGELGQGWLITRYDDAVHLLKDARLKKDVENVFAENEKPVISKTKEPLTEHMLNSDPPDHTRLRSLVQKAFTPRMILQLEDRIQHIANSLLDNVQRNSMNLVADFSFPLPIIVISEMLGIPLEDRQKFKNWSQAIIDFTDTPESLDKIKQFAEYLQYLVHKKRNEPADDLISALIQAESEGTTLSTKELYSTIMLLIVAGHETTVNLITNMIFALLNHPDQLETLQQNPNLIDSAIEEALRFHSPVELTTLRWAAEPLTLHGQEVKRKDVIVISLASANRDEKVFPDADVFDIARKNNRHIAFGHGSHFCLGAPLARLEAKIAVPALLRRYPDVQIQGRREDIKWKGNFLMRSLEELPLCF

>CYP107J11(DJ92_5476) Bacillus pseudomycoides 219298

MISKNELNFSLLAETQLASPMFKQKAYDIYKELRAFQPVYPLSLCEQGQGWLITRYEDAMALLKDARLMKNFENVFSKKEDVKVPFSLENRELLRNHMLNSDPPDHHRLRSLVQKAFTPQMILQLECRIQHIADALLNKVEHTHSINLVNDYAFPLPIMVISEMLGIPLEDQHKFRIWSQAIIDTPLRLEDVQKNNQKLEEFAEYIQYLVYKKRKNPTDDLISALIQAESEGIKLNASELYSTITLLIVAGHETTVNLIANMTLALLEHPVQLQKLLQNPDLIDSAIEEALRFYSPVELTTIRWTAEPFTMHGQDIQSKDRIIISLASSNRDEKIFSNADVFDVTRKNNRHIAFGYGSHFCLGASLARLESKIAISTLLRRMPNLQIQGEREQVKWKESYLMRSLEELPLQL

>CYP107K1_ortholog(BS34A_18930)Bacillus sp. BS34A

MEKLMFHPHGKEFHHNPFSVLGRFREEEPIHRFELKRFGATYPAWLITRYDDCMAFLKDNRITRDVKNVMNQEQIKMLNVSEDIDFVSDHMLAKDTPDHTRLRSLVHQAFTPRTIENLRGSIEQIAEQLLDEMEKENKADIMKSFASPLPFIVISELMGIPKEDRSQFQIWTNAMVDTSEGNRELTNQALREFKDYIAKLIHDRRIKPKDDLISKLVHAEENGSKLSEKELYSMLFLLVVAGLETTVNLLGSGTLALLQHKKECEKLKQQPEMIATAVEELLRYTSPVVMMANRWAIEDFTYKGHSIKRGDMIFIGIGSANRDPNFFENPEILNINRSPNRHISFGFGIHFCLGAPLARLEGHIAFKALLKRFPDIELAVAPDDIQWRKNVFLRGLESLPVSLSK

>CYP107K1_ortholog(BSU6051_17230)Bacillus subtilis subsp. subtilis 6051-HGW

MEKLMFHPHGKEFHHNPFSVLGRFREEEPIHRFELKRFGATYPAWLITRYDDCMAFLKDNRITRDVKNVMNQEQIKMLNVSEDIDFVSDHMLAKDTPDHTRLRSLVHQAFTPRTIENLRGSIEQIAEQLLDEMEKENKADIMKSFASPLPFIVISELMGIPKEDRSQFQIWTNAMVDTSEGNRELTNQALREFKDYIAKLIHDRRIKPKDDLISKLVHAEENGSKLSEKELYSMLFLLVVAGLETTVNLLGSGTLALLQHKKECEKLKQQPEMIATAVEELLRYTSPVVMMANRWAIEDFTYKGHSIKRGDMIFIGIGSANRDPNFFENPEILNINRSPNRHISFGFGIHFCLGAPLARLEGHIAFKALLKRFPDIELAVAPDDIQWRKNVFLRGLESLPVSLSK

>CYP107K1_ortholog(U712_09035)Bacillus subtilis PY79

MEKLMFHPHGKEFHHNPFSVLGRFREEEPIHRFELKRFGATYPAWLITRYDDCMAFLKDNRITRDVKNVMNQEQIKMLNVSEDIDFVSDHMLAKDTPDHTRLRSLVHQAFTPRTIENLRGSIEQIAEQLLDEMEKENKADIMKSFASPLPFIVISELMGIPKEDRSQFQIWTNAMVDTSEGNRELTNQALREFKDYIAKLIHDRRIKPKDDLISKLVHAEENGSKLSEKELYSMLFLLVVAGLETTVNLLGSGTLALLQHKKECEKLKQQPEMIATAVEELLRYTSPVVMMANRWAIEDFTYKGHSIKRGDMIFIGIGSANRDPNFFENPEILNINRSPNRHISFGFGIHFCLGAPLARLEGHIAFKALLKRFPDIELAVAPDDIQWRKNVFLRGLESLPVSLSK

>CYP107K1_ortholog(B657_17230)Bacillus subtilis QB928

MEKLMFHPHGKEFHHNPFSVLGRFREEEPIHRFELKRFGATYPAWLITRYDDCMAFLKDNRITRDVKNVMNQEQIKMLNVSEDIDFVSDHMLAKDTPDHTRLRSLVHQAFTPRTIENLRGSIEQIAEQLLDEMEKENKADIMKSFASPLPFIVISELMGIPKEDRSQFQIWTNAMVDTSEGNRELTNQALREFKDYIAKLIHDRRIKPKDDLISKLVHAEENGSKLSEKELYSMLFLLVVAGLETTVNLLGSGTLALLQHKKECEKLKQQPEMIATAVEELLRYTSPVVMMANRWAIEDFTYKGHSIKRGDMIFIGIGSANRDPNFFENPEILNINRSPNRHISFGFGIHFCLGAPLARLEGHIAFKALLKRFPDIELAVAPDDIQWRKNVFLRGLESLPVSLSK

>CYP107K1_ortholog(BSU17230)Bacillus subtilis subsp. subtilis 168

MEKLMFHPHGKEFHHNPFSVLGRFREEEPIHRFELKRFGATYPAWLITRYDDCMAFLKDNRITRDVKNVMNQEQIKMLNVSEDIDFVSDHMLAKDTPDHTRLRSLVHQAFTPRTIENLRGSIEQIAEQLLDEMEKENKADIMKSFASPLPFIVISELMGIPKEDRSQFQIWTNAMVDTSEGNRELTNQALREFKDYIAKLIHDRRIKPKDDLISKLVHAEENGSKLSEKELYSMLFLLVVAGLETTVNLLGSGTLALLQHKKECEKLKQQPEMIATAVEELLRYTSPVVMMANRWAIEDFTYKGHSIKRGDMIFIGIGSANRDPNFFENPEILNINRSPNRHISFGFGIHFCLGAPLARLEGHIAFKALLKRFPDIELAVAPDDIQWRKNVFLRGLESLPVSLSK

>CYP107K1_ortholog(BSUA_01854)Bacillus subtilis subsp. subtilis JH642

MEKLMFHPHGKEFHHNPFSVLGRFREEEPIHRFELKRFGATYPAWLITRYDDCMAFLKDNRITRDVKNVMNQEQIKMLNVSEDIDFVSDHMLAKDTPDHTRLRSLVHQAFTPRTIENLRGSIEQIAEQLLDEMEKENKADIMKSFASPLPFIVISELMGIPKEDRSQFQIWTNAMVDTSEGNRELTNQALREFKDYIAKLIHDRRIKPKDDLISKLVHAEENGSKLSEKELYSMLFLLVVAGLETTVNLLGSGTLALLQHKKECEKLKQQPEMIATAVEELLRYTSPVVMMANRWAIEDFTYKGHSIKRGDMIFIGIGSANRDPNFFENPEILNINRSPNRHISFGFGIHFCLGAPLARLEGHIAFKALLKRFPDIELAVAPDDIQWRKNVFLRGLESLPVSLSK

>CYP107K1_ortholog(BSUB_01854)Bacillus subtilis subsp. subtilis AG1839

MEKLMFHPHGKEFHHNPFSVLGRFREEEPIHRFELKRFGATYPAWLITRYDDCMAFLKDNRITRDVKNVMNQEQIKMLNVSEDIDFVSDHMLAKDTPDHTRLRSLVHQAFTPRTIENLRGSIEQIAEQLLDEMEKENKADIMKSFASPLPFIVISELMGIPKEDRSQFQIWTNAMVDTSEGNRELTNQALREFKDYIAKLIHDRRIKPKDDLISKLVHAEENGSKLSEKELYSMLFLLVVAGLETTVNLLGSGTLALLQHKKECEKLKQQPEMIATAVEELLRYTSPVVMMANRWAIEDFTYKGHSIKRGDMIFIGIGSANRDPNFFENPEILNINRSPNRHISFGFGIHFCLGAPLARLEGHIAFKALLKRFPDIELAVAPDDIQWRKNVFLRGLESLPVSLSK

>CYP107K1_ortholog(A7A1_3357)Bacillus subtilis subsp. subtilis BSP1

MQMEKLMFHPHGKEFHHNPFSVLGRFREEEPIHRFELKRFGATYPAWLITRYDDCMAFLKDNRITRDVKNVMNQEQIKMLNVSEDIDFVSDHMLAKDTPDHTRLRSLVHQAFTPRTIENLRGSIEQIAEQLLDEMEKENKADIMKSFASPLPFIVISELMGIPKEDRSQFQIWTNAMVDTSEGNRELTNQALREFKDYISKLIHDRRIKPKDDLISKLVHAEENGSKLSEKELYSMLFLLVVAGLETTVNLLGSGTLALLQHKKECEKLKQQPEMIATAVEELLRYTSPVVMMANRWAIEDFTYKGHSIKRGDMIFIGIGSANRDPNFFENPEILNINRSPNRHISFGFGIHFCLGAPLARLEGHIAFNAASEEIS

>CYP107K1_ortholog(BsLM_1820)Bacillus sp. LM 4-2

MEKLMFHPHGKEFHHNPFSVLGRFREEEPIHRFELKRFGATYPAWLITRYDDCMAFLKDNRITRDVKNVMNQEQIKMLNVSEDIDFVSDHMLAKDTPDHTRLRSLVHQAFTPRTIENLRGSIEQIAEQLLDEMEKENKADIMKSFASPLPFIVISKLMGIPKEDRSQFQIWTNAMVDTSEGNRELTNQALREFKDYIAKLIHDRRIKPKDDLISKLVHAEENGSKLSEKELYSMLFLLVVAGLETTVNLLGSGTLALLQHKKECEKLKQHPEMIATAVEELLRYTSPVVMMANRWAIEDFTYKGHSIKRGDMIFIGIGSANRDPNFFENPEILNINRSPNRHISFGFGIHFCLGAPLARLEGHIAFNALLKRFPDIELAVAPDDIQWRKNVFLRGLESLPVSLSK

>CYP107K1_ortholog(BGM20_03015)Bacillus gibsonii

MEKLMFHPHGKEFHHNPFSVLGRFREEEPIHRFELKRFGATYPAWLITRYDDCMAFLKDNRITRDVKNVMNQEQIKMLNVSEDIDFVSDHMLAKDTPDHTRLRSLVHQAFTPRTIENLRGSIEQIAEQLLDEMEKENKADIMKSFASPLPFIVISELMGIPKEDRSQFQIWTNAMVDTSEGNRELTNQALREFKDYIAKLIHDRRIKPKDDLISKLVHAEENGSKLSEKELYSMLFLLVVAGLETTVNLLGSGTLALLQHKKECEKLKQYPEMIATAVEELLRYTSPVVMMANRWAIEDFTYKGHSIKRGDMIFIGIVSANRDPNFFENPEILNINRSPNRHISFGFGIHFCLGAPLARLEGHIAFNALLKRFPDIQLAVAPDDIQWRKNVFLRGLESLPVSLSK

>CYP107K1_ortholog(I33_1910)Bacillus subtilis subsp. subtilis RO-NN-1

MEKLMFHPHGKEFHHNPFSVLGRFREEEPIHRFELKRFGATYPAWLITRYDDCMAFLKDNRITRDVKNVMNQEQIKMLNVSEDIDFVSDHMLAKDTPDHTRLRSLVHQAFTPRTIENLRGSIEQIAEQLLDEMEKENKADIMKSFASPLPFIVISELMGIPKEDRSQFQIWTNAMVDTSEGNRELTNQALREFKDYIAKLIHDRRIKPKDDLISKLVHAEENGSKLSEKELYSMLFLLIVAGLETTVNLLGSGTLALLQHKKECEKLKQHPEMIATAVEELLRYTSPVVMMANRWAIEDFTYKGHSIKRGDMIFIGIGSANRDPNFFENPEILNINRSPNRHISFGFGIHFCLGAPLARLEGHIAFNALLKRFPDIELAAAPDDIQWRKNVFLRGLESLPVSLSK

>CYP107K1_ortholog(Q433_09795)Bacillus subtilis subsp. subtilis OH 131.1

MEKLMFHPHGKEFHHNPFSVLGRFREEEPIHRFELKRFGATYPAWLITRYDDCMAFLKDNRITRDVKNVMNQEQIKMLNVSEDIDFVSDHMLAKDTPDHTRLRSLVHQAFTPRTIENLRGSIEQIAEQLLDEMEKENKADIMKSFASPLPFIVISELMGIPKEDRSQFQIWTNAMVDTSEGNRELTNQALREFKDYIAKLIHDRRIKPKDDLISKLVHAEENGSKLSEKELYSMLFLLVVAGLETTVNLLGSGTLALLQHKKECEKLKQYPEMIATAVEELLRYTSPVVMMANRWAIEDFTYKGHSIKRGDMIFIGIVSANRDPNFFENPEILNINRSPNRHISFGFGIHFCLGAPLARLEGHIAFNALLKRFPDIQLAVAPDDIQWRKNVFLRGLESLPVSLSK

>CYP107K1_ortholog(C663_1772)Bacillus subtilis XF-1

MQMEKLMFHPHGKEFHHNPFSVLGRFREEEPIHRFELKRFGATYPAWLITRYDDCMAFLKDNRITRDVKNVMDQEQIKMLNVSEDIDFVSDHMLAKDTPDHTRLKSLVHQAFTPRTIENLRGSIEQIAEQLLDEMEKENKADIMKSFASPLPFIVISELMGIPKEDRSQFQIWTNAMVDTSEGNRELTNQALREFKDYIAKLIHDRRIKPKDDLISKLVHAEENGSKLSEKELYSMLFLLVVAGLETTVNLLGSGTLALLQHKKECEKLKQHPEMIATAVEELLRYTSPVVMMANRWAIEDFTYKGHSIKRGDMIFIGIGSANRDPNFFENPEILNINRSPNRHISFGFGIHFCLGAPLARLEGHIAFNALLKRFPDIELAVAPEDIQWRKNVFLRGLESLPVSLSK

>CYP107K1_ortholog(QF06_07660)Bacillus sp. YP1

MEKLMFHPHGKEFHHNPFSVLGRFREEEPIHRFELKRFGATYPAWLITRYDDCMAFLKDNRITRDVKNVMNQEQIKMLNVSEDIDFVSDHMLAKDTPDHTRLRSLVHQAFTPRTIENLRCSIEQIAEQLLDEMEKENKADIMKSFASPLPFIVISELMGIPKEDRSQFQIWTNAMVDTSEGNRELTNQALREFKDYIAKLIHDRRIKPKDDLISKLVHAEENGSKLSEKELYSMLFLLVVAGLETTVNLLGSGTLALLQHMKECEKLKQQPEMIATAVEELLRYTSPVVMMANRWAIEDFRYKGHSIKRGDMIFIGIGSANRDPNFFENPEILNINRSPNRHISFGFGIHFCLGAPLARLEGHIAFNALLKRFPDIELAVAPDDIQWRKNVFLRGLESLPVSLSK

>CYP107K1_ortholog(BSn5_20735)Bacillus subtilis BSn5

MEKLMFHPHGKEFHHNPFSVLGRFREEEPIHRFELKRFGATYPAWLITRYDDCMAFLKDNRITRDVKNVMNQEQIKMLNVSEDIDFVSDHMLAKDTPDHTRLRSLVHQAFTPRTIENLRGSIEQIAEQLLDEMEKENKADIMKSFASPLPFIVISELMGIPKEDRSQFQIWTNAMVDTSEGNRELTNQALREFKDYIAKLIHDRRIKPKDDLISKLVHAEENGSKLSEKELYSMLFLLIVAGLETTVNLLGSGTLALLQHMKECEKLKQHPEMIATAVEELLRYTSPVVMMANRWAIEDFTYKGHSIKRGDMIFIGIGSANRDPNFFENPEILNINRSPNRHISFGFGIHFCLGAPLARLEGHIAFNALLKRFPDIELAVAPDDIQWRKNVFLRGLESLPVSLSK

>CYP107K1_ortholog(I653_08845)Bacillus subtilis subsp. subtilis BAB-1

MEKLMFHPHGKEFHHNPFSVLGRFREEEPIHRFELKRFGATYPAWLITRYDDCMAFLKDNRITRDVKNVMDQEQIKMLNVSEDIDFVSDHMLAKDTPDHTRLKSLVHQAFTPRTIENLRGSIEQIAEQLLDEMEKENKADIMKSFASPLPFIVISELMGIPKEDRSQFQIWTNAMVDTSEGNRELTNQALREFKDYIAKLIHDRRIKPKDDLISKLVHAEENGSKLSEKELYSMLFLLVVAGLETTVNLLGSGTLALLQHKKECEKLKQHPEMIATAVEELLRYTSPVVMMANRWAIEDFTYKGHSIKRGDMIFIGIGSANRDPNFFENPEILNINRSPNRHISFGFGIHFCLGAPLARLEGHIAFNALLKRFPDIELAVAPEDIQWRKNVFLRGLESLPVSLSK

>CYP107K1_ortholog(MY9_1873)Bacillus sp. JS

MEKLMFHPHGKEFHDNPFSVLGRFREEDPIHRFELKRFGGTYPAWLITRYDDCMAFLKDTRITRDVKNVMSQEQIKMLNVSEDIDFVSDHMLAKDTPDHTRLRSLVHQAFTPRTIENLRGSIEQISEQLLDEMEKEDKADIMTSFASPLPFIVISELMGIPKEDRTQFQIWTNAMVDTSEGNRELTNQALREFKEYIAKLIHDRRIKPKDDLISKLVHAEENGSKLSEKELYSMLFLLVVAGLETTVNLLGSGTLALLQHKKEREKLKQHPEMIATAVEELLRYTSPVVMMANRWAIEDFTYKGHSIKRGDMIFIGIGSANRDPNFFENPEILNINRSPNRHISFGFGIHFCLGAPLARLEGHIAFNALLKRFPDIELAMPDDIQWRKNVFLRGLESLPVSLSK

>CYP107K1_ortholog(BSUW23_08860)Bacillus subtilis subsp. spizizenii W23

MEKLMFHPHGKEFHLNPFSVLGRFREEDPVHQFELKRFGGTYPAWLITRYDDCMAFLKDNRITRDVKNVMSQEQIKMLNVSEDIDFVSDHMLAKDTPDHTRLRSLVHQAFTPRTIENLRGSIEQITEQLLDEMEKENKADIMKSFASPLPFIVISELMGIPKEDRAQFQIWTNAMVDTSESNRELTNQALREFKDYIAKLIQDRRIQPKDDLISKLVHAEENGSKLSEKELYSMLFLLVVAGLETTVNLLGSGTLALLQHKEEFEKLKQYPEMIATAVEELLRYTSPVVMMANRWAIEDFTYKGHSIKRGDMIFIGIGSANRDPNFFENPETLNLNRSPNRHISFGFGIHFCLGAPLARLEGHIAFNALLKRFPDIELAAAPDDIHWRKNVFLRGLENLPVSLSK

>CYP107K1_ortholog(GYO_2078)Bacillus subtilis subsp. spizizenii TU-B-10

MEKLMFHPHGKEFHQNPFSVLGRFREEDPIHRFELKRFGGTYPAWLITRYDDCMAFLKDNRITRDVKNVMSQEQIKMLNVSEDIDFVSDHMLAKDTPDHTRLRSLVHQAFTPRTIENLRGSIEQITEQLLDEMEKENKADIMKSFASPLPFIVISELMGIPKEDRAQFQIWTNAIVDTSESNRELTNQALREFKDYIAKLIHDRRIQPRDDLISKLVHADENGSKLSEKELYSMLFLLVVAGLETTVNLLGSGTLALLQHKEEFEKLKQHPEMIATAVEELLRYTSPVVMMANRWAIEDFTYKGHSIKRGDMIFIGIGSANRDPNFFENPETLNLNRSPNRHISFGFGIHFCLGAPLARLEGHIAFNALLKRFPDIELAAAPNDIQWRKNVFLRGLECLPVSLSK

>CYP107K2(BATR1942_06420)Bacillus atrophaeus 1942

MEKLVFNAHSSEFHENPFTVLGHFREHDPIHHFELHRFGVTYRAWLITRYDDCMAFLKDNRITRDVKNVMSKEQIKKLNVSDDIDFVSDHMLAKDTPDHTRLRSLVHQAFTPRIIENLRTSIEQITERLLDDMEKENEPDIMKSFASPLPFIVISEMMGIPEEDRSQFQVWTNAMVDTSEGNQEKTNQSLREFKDYIGKLIQERRIHPKDDMISKLVHAEEDGHKLSEKELYSMLFLLVVAGLETTVNLLGSGTLALLQHREELEKLKQHPDMIDTAVEELLRYTAPVVMMANRWAIEDFTYQGHSIKRGDMIFIGIGSANRDPERFENPEKFDISRSPNRHISFGFGIHFCLGAPLARLEGQIAFNALLKRFPYIELAVASDEMKWRKNAFLRGLESLPVSLAKKNSLSRYS

>CYP107K2_ortholog(TD68_05870)Bacillus atrophaeus NRS 1221A

MEKLVFNAHSSEFHENPFTVLGHFREHDPIHHFELHRFGVTYRAWLITRYDDCMAFLKDNRITRDVKNVMSKEQIKKLNVSDDIDFVSDHMLAKDTPDHTRLRSLVHQAFTPRIIENLRTSIEQITERLLDDMEKENEPDIMKSFASPLPFIVISEMMGIPEEDRSQFQVWTNAMVDTSEGNQEKTNQSLREFKDYIGKLIQERRIHPKDDMISKLVHAEEDGHKLSEKELYSMLFLLVVAGLETTVNLLGSGTLALLQHREELEKLKQHPDMIDTAVEELLRYTAPVVMMANRWAIEDFTYQGHSIKRGDMIFIGIGSANRDPERFENPEKFDISRSPNRHISFGFGIHFCLGAPLARLEGQIAFNALLKRFPYIELAVASDEMKWRKNAFLRGLESLPVSLAKKNSLSRYS

>CYP107K3(SB24_01170)Bacillus sp. Pc3

MEKTMFHPHSPEFHENPFAVLSRFRAQDPIHKFELQRFGGTFPAWLITRYDDCMAFLKDGRITRDVKRVMPKELIAKLNVSEDIDFVSDHMLAKDPPDHSRLRSLVHQGFTPRMIEQLRTGIEQITEELLDEMETKADPDIMRDFAAPLPFIVISELLGIPKEDRAKFQVWTNAMVDTSESGQDATNQALKEFKQYMKTLIEGKRKHPGEDLTSKLIYAEEDGQKLSESELYSMLFLLVVAGLETTVNLLGSGTLALLLHKDQMEKIKRQPENIQTAVEELLRYTSPVIMMANRWAIEDFTYKDVSIKKGDMIFIGIGSANRDPEYFDDPDTLNIARTPNRHISFGFGIHFCLGAPLARMEASIAFTALLKRFPNIELKGAPEDVTWRKNVFLRGLETLPVRF

>CYP107K3_ortholog(BASU_1663)Bacillus velezensis UCMB5113

MEKTMFHPHSPEFHENPFAVLSRFREQDPIHKFELQRFGGTFPAWLITRYDDCMAFLKDGRITRDVKRVMPKELIAKLNVSEDIDFVSEHMLAKDPPDHSRLRSLVHQGFTPRMIEQLRTGIEQITEELLDEMETKADPDIMRDFAAPLPFIVISELLGIPKEDRAKFQVWTNAMVDTSESGQDATNQALKEFKQYMKTLIEEKRKHPGEDLTSKLIYAEEDGQKLSESELYSMLFLLVVAGLETTVNLLGSGTLALLLHKDQMEKIKRQPENIQTAVEELLRYTSPVIMMANRWAIEDFTYKDVSIKKGDMIFIGIGSANRDPEYFDDPDTLNIARTPNRHISFGFGIHFCLGAPLARMEASIAFTALLKRFPNIELKGAPEDVTWRKNVFLRGLETLPVRF

>CYP107K3_ortholog(BAM5036_1645)Bacillus velezensis UCMB5036

MEKTMFHPHSPEFHENPFAVLSRFRAQDPIHKFELQRFGGTFPAWLITRYDDCMAFLKDGRITRDVKRVMPKELIAKLNVSEDIDFVSDHMLAKDPPDHSRLRSLVHQGFTPRMIEQLRTGIEQITEELLDEMETKADPDIMRDFAAPLPFIVISELLGIPKEDRAKFQVWTNAMVDTSESGQDATNQALKEFKQYMKTLIEEKRKHPGEDLTSKLIYAEEDGQKLSESELYSMLFLLVVAGLETTVNLLGSGTLALLLHKDQMGKIKRQPENIQTAVEELLRYTSPVIMMANRWAIEDFTYKDVSIKKGDMIFIGIGSANRDPEYFDDPDTLNIARTPNRHISFGFGIHFCLGAPLARMEASIAFTALLKRFPNIELKGAPEDVTWRKNVFLRGLETLPVRF

>CYP107K3_ortholog(RBAU_1684)Bacillus velezensis UCMB5033

MEKTMFHPHSPEFHENPFAVLSRFREQDPIHKFELQRFGGTFPAWLITRYDDCMAFLKDGRITRDVKRVMPKELIAKLNVSEDIDFVSEHMLAKDPPDHSRLRSLVHQGFTPRMIEQLRTGIEQITEELLDEMETKADPDIMRDFAAPLPFIVISELLGIPKEDRAKFQVWTNAMVDTSESGQDATNQALKEFKQYMKTLIEEKRKHPGEDLTSKLIYAEEDGQKLSESELYSMLFLLVVAGLETTVNLLGSGTLALLLHKDQMEKIKRQPENIQTAVEELLRYTSPVIMMANRWAIEDFTYKDVSIKKGDMIFIGIGSANRDPEYFDDPDTLNIARTPNRHISFGFGIHFCLGAPLARMEASIAFTALLKRFPNIELKGAPEDVTWRKNVFLRGLETFPVRF

>CYP107K3_ortholog(V529_16630)Bacillus velezensis SQR9

MEKTMFHPHSPEFHENPFAVLSRFREQDPIHKFELQRFGGTFPAWLITRYDDCMAFLKDGRITRDVKRVMPKELIAKLNVSEDIDFVSEHMLAKDPPDHSRLRSLVHQGFTPRMIEQLRTGIEQITEELLDEMETKADPDIMRDFAAPLPFIVISELLGIPKEDRAKFQVWTNAMVDTSESGQDATNQALKEFKQYMKTLIEEKRKHPGEDLTSKLIYAEEDGQKLSESELYSMLFLLVVAGLETTVNLLGSGTLALLLHKDQMEKIKRQPEDIQTAVEELLRYTSPVIMMANRWAIEDFTYKDVSIKKGDMIFIGIGSANRDPEYFDDPDTLNIARTPNRHISFGFGIHFCLGAPLARMEASIAFTALLKRFPNIELKGAPEDVTWRKNVFLRGLETLPVRF

>CYP107K3_ortholog(U471_17460)Bacillus amyloliquefaciens CC178

MEKTMFHPHSPEFHENPFAVLSRFREQDPIHKFELQRFGGTFPAWLITRYDDCMAFLKDGRITRDVKRVMPKELIAKLNVSEDIDFVSEHMLAKDPPDHSRLRSLVHQGFTPRMIEQLRTGIEQITEELLDEMETKADPDIMRDFAAPLPFIVISELLGIPKEDRAKFQVWTNAMVDTSESGQDATNQALKEFKQYMKTLIEEKRKHPGEDLTSKLIYAEEDGQKLSESELYSMLFLLVVAGLETTVNLLGSGTLALLLHKDQMEKIKRQPENIQTAVEELLRYTSPVIMMANRWAIEDFTYKDVSIKKGDMIFIGIGSANRDPEYFDDPDTLNIARTPNRHISFGFGIHFCLGAPLARMEASIAFTALLKRFPNIELKGAAEDVTWRKNVFLRGLETLPVRF

>CYP107K3_ortholog(RBAM_017030)Bacillus velezensis FZB42

MEKTMFHPHSPEFHENPFAVLSRFREQDPIHKFELQRFGGTFPAWLITRYDDCMAFLKDGRITRDVKRVMPKELIAKLNVSEDIDFVSEHMLAKDPPDHSRLRSLVHQGFTPRMIEQLRTGIEQITEELLDEMETKADPDIMRDFAAPLPFIVISELLGIPKEDRAKFQVWTNAMVDTSESGQDATNQALKEFKQYMKTLIEEKRKHPGEDLTSKLIYAEEDGQKLSESELYSMLFLLVVAGLETTVNLLGSGTLALLLHKDQMEKIKRQPENIQTAVEELLRYTSPVIMMANRWAIEDFTYKDVSIKKGDMIFIGIGSANRDPEYFDDPDTLNIARTPNRHISFGFGIHFCLGAPLARMEASIAFTALLKRFPNIELKGAAEDVTWRKNVFLRGLETLPVRF

>CYP107K3_ortholog(NG74_01770)Bacillus velezensis

MEKTMFHPHSPEFHENPFAVLSRFREQDPIHKFELQRFGGTFPAWLITRYDDCMAFLKDGRITRDVKRVMPKELIAKLNVSEDIDFVSEHMLAKDPPDHSRLRSLVHQGFTPRMIEQLRTGIEQITEELLDEMETKADPDIMRDFAAPLPFIVISELLGIPKEDRAKFQVWTNAMVDTSESGQDATNQALKEFKQYMKTLIEEKRKHPAEDLTSKLIYAEEDGQKLSESELYSMLFLLVVAGLETTVNLLGSGTLALLLHKDQMEKIKRQPEDIQTAVEELLRYTSPVIMMANRWAIEDFTYKDVSIKKGDMIFIGIGSANRDPEYFDDPDTLNIARTPNRHISFGFGIHFCLGAPLARMEASIAFTALLKRFPNIELKGAPEDVTWRKNVFLRGLETLPVRF

>CYP107K3_ortholog(BAPNAU_2044)Bacillus velezensis NAU-B3

MEKTMFHPHSPEFHENPFAVLSRFREQDPIHKFELQRFGGTFPAWLITRYDDCMAFLKDGRITRDVKRVMPKELIAKLNVSEDIDFVSEHMLAKDPPDHSRLRSLVHQGFTPRMIEQLRTGIEQITEELLDEMETKADPDIMRDFAAPLPFIVISELLGIPKEDRAKFQVWTNAMVDTSESGQDATNQALKEFKQYMKTLIEEKRKHPAEDLTSKLIYAEEDGQKLSESELYSMLFLLVVAGLETTVNLLGSGTLALLLHKDQMEKIKRQPEDIQTAVEELLRYTSPVIMMANRWAIEDFTYKDVSIKKGDMIFIGIGSANRDPEYFDDPDTLNIARTPNRHISFGFGIHFCLGAPLARMEASIAFTALLKRFPNIELKGAPEDVTWRKNVFLRGLETLPVRF

>CYP107K3_ortholog(OY17_11620)Bacillus sp. BH072

MEKTMFHPHSPEFHENPFAVLSRFREQDPIHKFELQRFGGTFPAWLITRYDDCMAFLKDGRITRDVKRVMPKELIAKLNVSEDIDFVSEHMLAKDPPDHSRLRSLVHQGFTPRMIEQLRTGIEQITEELLDEMETKADPDIMRDFAAPLPFIVISELLGIPKEDRAKFQVWTNAMVDTSESGQDATNQALKEFKQYMKTLIEEKRKHPAEDLTSKLIYAEEDGQKLSESELYSMLFLLVVAGLETTVNLLGSGTLALLLHKDQMEKIKRQPENIQTAVEELLRYTSPVIMMANRWAIEDFTYKDVSIKKGDMIFIGIGSANRDPEYFDDSDTLNIARTPNRHISFGFGIHFCLGAPLARMEASIAFTALLKRFPNIELKGAPEDVTWRKNVFLRGLETLPVRF

>CYP107K3_ortholog(MUS_1888)Bacillus amyloliquefaciens Y2

MEKTMFHPHSPEFHENPFAVLSRFREQDPIHKFELQRFGGTFPAWLITRYDDCMAFLKDGRITRDVKRVMPKELIAKLNVSEDIDFVSEHMLAKDPPDHSRLRSLVHQGFTPRMIEQLRTGIEQITEELLDEMETKADPDIMRDFAAPLPFIVISELLGIPKEDRAKFQVWTNAMVDTSESGQDATNQALKEFKQYMKTLIEEKRKHPAEDLTSKLIYAEEDGQKLSESELYSMLFLLVVAGLETTVNLLGSGTLALLLHKDQMEKIKRQPENIQTAVEELLRYTSPVIMMANRWAIEDFTYKDVSIKKGDMIFIGIGSANRDPEYFDDSDTLNIARTPNRHISFGFGIHFCLGAPLARMEASIAFTALLKRFPNIELKGAPEDVTWRKNVFLRGLETLPVRF

>CYP107K3_ortholog(BANAU_1679)Bacillus velezensis YAU B9601-Y2

MEKTMFHPHSPEFHENPFAVLSRFREQDPIHKFELQRFGGTFPAWLITRYDDCMAFLKDGRITRDVKRVMPKELIAKLNVSEDIDFVSEHMLAKDPPDHSRLRSLVHQGFTPRMIEQLRTGIEQITEELLDEMETKADPDIMRDFAAPLPFIVISELLGIPKEDRAKFQVWTNAMVDTSESGQDATNQALKEFKQYMKTLIEEKRKHPAEDLTSKLIYAEEDGQKLSESELYSMLFLLVVAGLETTVNLLGSGTLALLLHKDQMEKIKRQPENIQTAVEELLRYTSPVIMMANRWAIEDFTYKDVSIKKGDMIFIGIGSANRDPEYFDDSDTLNIARTPNRHISFGFGIHFCLGAPLARMEASIAFTALLKRFPNIELKGAPEDVTWRKNVFLRGLETLPVRF

>CYP107K3_ortholog(AJ82_09675)Bacillus velezensis TrigoCor1448

MEKTMFHPHSPEFHENPFAVLSRFREQDPIHKFELQRFGGTFPAWLITRYDDCMAFLKDGRITRDVKRVMPKELIAKLNVSEDIDFVSEHMLAKDPPDHSRLRSLVHQGFTPRMIEQLRTGIEQITEELLDEMETKADPDIIRDFAAPLPFIVISELLGIPKEDRAKFQVWTNAMVDTSESGQDATNQALKEFKQYMKTLIEEKRKHPGEDLTSKLIYAEEDGQKLSESELYSMLFLLVVAGLETTVNLLGSGTLALLLHKDQMEKIKRQPENIQTAVEELLRYTSPVIMMANRWAIEDFTYKDVSIKKGDMIFIGIGSANRDPEYFDDPDTLNIARTPNRHISFGFGIHFCLGAPLARMEASIAFTALLKRFPNIELKGAAEDVTWRKNVFLRGLETLPVRF

>CYP107K3_ortholog(U722_09025)Bacillus amyloliquefaciens LFB112

MEKTMFHPHSPEFHEDPFAVLSRFREQDPIHKFELQRFGGTFPAWLITRYDDCMAFLKDGRITRDVKRVMPKELIAKLNVSEDIDFVSEHMLAKDPPDHSRLRSLVHQGFTPRMIEQLRTGIEQITEELLNEMETKADPDIMRDFAAPLPFIVISELLGIPKEDRAKFQVWTNAMVDTSESGQDATNQALKEFKQYMKTLIEEKRKHPAEDLTSKLIYAEEDGQKLSESELYSMLFLLVVAGLETTVNLLGSGTLALLLHKDQMEKIKRQPENIQTAVEELLRYTSPVIMMANRWAIEDFTYKDVSIKKGDMIFIGIGSANRDPEYFDDPDTLNIARTPNRHISFGFGIHFCLGAPLARMEASIAFTSLLKRFPNIELKGAPEDVTWRKNVFLRGLETLPVRF

>CYP107K3_ortholog(KSO_010830)Bacillus amyloliquefaciens IT-45

MEKTMFHPHSPEFHEDPFAVLSRFREQDPIHKFELQRFGGTFPAWLITRYDDCMAFLKDGRITRDVKRVMPKELIAKLNVSEDIDFVSEHMLAKDPPDHSRLRSLVHQGFTPRMIEQLRTGIEQITEELLNEMETKADPDIMRDFAAPLPFIVISELLGIPKEDRAKFQVWTNAMVDTSESGQDATNQALKEFKQYMKTLIEEKRKHPAEDLTSKLIYAEEDGQKLSESELYSMLFLLVVAGLETTVNLLGSGTLALLLHKDQMEKIKRQPENIQTAVEELLRYTSPVIMMANRWAIEDFTYKDVSIKKGDMIFIGIGSANRDPEYFDDPDTLNIARTPNRHISFGFGIHFCLGAPLARMEASIAFTSLLKRFPNIELKGAHEDVTWRKNVFLRGLETLPVRF

>CYP107K3_ortholog(BACAU_1675)Bacillus velezensis CAU B946

MEKTMFHPHSPEFHEDPFAVLSRFREQDPIHKFELQRFGGTFPAWLITRYDDCMAFLKDGRITRDVKRVMPKELIAKLNVSEDIDFVSEHMLAKDPPDHSRLRSLVHQGFTPRMIEQLRTGIEQITEELLNEMETKADPDIMRDFAAPLPFIVISELLGIPKEDRAKFQVWTNAMVDTSESGQDATNQALKEFKQYMKTLIEEKRKHPAEDLTSKLIYAEEDGQKLSESELYSMLFLLVVAGLETTVNLLGSGTLALLLHKDQMEKIKRQPENIQTAVEELLRYTSPVIMMANRWAIEDFTYKDVSIKKGDMIFIGIGSANRDPEYFDDPDTLNIARTPNRHISFGFGIHFCLGAPLARMEASIAFTSLLKRFPNIELKGAHEDVTWRKNVFLRGLETLPVRF

>CYP107K3_ortholog(B938_08830)Bacillus velezensis AS43.3

MSFQKHEGGEIQMEKTMFHPHSPEFHENPFAVLSRFREQDPIHKFELQRFGGTFPAWLITRYDDCMAFLKDGRITRDVKRVMPKELIAKLNVSEDIDFVSEHMLAKDPPDHSRLRSLVHQGFTPRMIEQLRTGIEQITEELLDEMETKADPDIMRDFAAPLPFIVISELLGIPKEDRAKFQVWTNAMVDTSESGQDATNQALKEFKQYMKTLIEEKRKHPGEDLTSKLIYAEEDGQKLSESELYSMLFLLVVAGLETTVNLLGSGTLALLLHKDQMEKIKRQPENIQTAVEELLRYTSPVIMMANRWAIEDFTYKDVSIKKGDMIFIGIGSANRDPEYFDDPDTLNIARTPNRHISFGFGIHFCLGAPLARMEASIAFTALLKRFPNIELKGAAEDVTWRKNVFLRGLKHCLSDFNIAPLFFGGFFNVL

>CYP107K3_ortholog(LL3_01878)Bacillus amyloliquefaciens LL3

MEKIMFHPHSSEFHENPFAVLSRFREQDPIHKFELQRFGGTFPAWLITRYDDCMAFLKDGRITRDVKRVMPKELIAKLNVSEDIDFVSEHMLAKDPPDHSRLRSLVHQGFTPRMIEQLRTGIERITEELLDEMETKADPDIMRDFAAPLPFIVISELLGIPKEDRAKFQVWTSAMVDTSESGQDATNQALREFKQYMQTLIEEKRNHPGEDLTSKLIYAEEDGQKLSESELYSMLFLLVVAGLETTVNLLGSGTLALLLHKDQLENIKRQPDTIQTAVEELLRYTSPVIMMANRWAIEDFTYKDVSIKKGDMIFIGIGSANRDPEYFDEPDTLNVARTPNRHISFGFGIHFCLGAPLARMEASIAFNALLKRFPNIELNGSSEDLTWRKNVFLRGLETLPVKF

>CYP107K3_ortholog(BAMTA208_08540)Bacillus amyloliquefaciens TA208

MEKIMFHPHSSEFHENPFAVLSRFREQDPIHKFELQRFGGTFPAWLITRYDDCMAFLKDGRITRDVKRVMPKELIAKLNVSEDIDFVSEHMLAKDPPDHSRLRSLVHQGFTPRMIEQLRTGIERITEELLDEMETKADPDIMRDFAAPLPFIVISELLGIPKEDRAKFQVWTSAMVDTSESGQDATNQALREFKQYMQTLIEEKRNHPGEDLTSKLIYAEEDGQKLSESELYSMLFLLVVAGLETTVNLLGSGTLALLLHKDQLENIKRQPDTIQTAVEELLRYTSPVIMMANRWAIEDFTYKDVSIKKGDMIFIGIGSANRDPEYFDEPDTLNVARTPNRHISFGFGIHFCLGAPLARMEASIAFNALLKRFPNIELNGSSEDLTWRKNVFLRGLETLPVKF

>CYP107K3_ortholog(BAMF_1791)Bacillus amyloliquefaciens DSM 7

MEKIMFHPHSSEFHENPFAVLSRFREQDPIHKFELQRFGGTFPAWLITRYDDCMAFLKDGRITRDVKRVMPKELIAKLNVSEDIDFVSEHMLAKDPPDHSRLRSLVHQGFTPRMIEQLRTGIERITEELLDEMETKADPDIMRDFAAPLPFIVISELLGIPKEDRAKFQVWTSAMVDTSESGQDATNQALREFKQYMQTLIEEKRNHPGEDLTSKLIYAEEDGQKLSESELYSMLFLLVVAGLETTVNLLGSGTLALLLHKDQLENIKRQPDTIQTAVEELLRYTSPVIMMANRWAIEDFTYKDVSIKKGDMIFIGIGSANRDPEYFDEPDTLNVARTPNRHISFGFGIHFCLGAPLARMEASIAFNALLKRFPNIELNGSSEDLTWRKNVFLRGLETLPVKF

>CYP107K3_ortholog(BAXH7_01741)Bacillus amyloliquefaciens XH7

MEKIMFHPHSSEFHENPFAVLSRFREQDPIHKFELQRFGGTFPAWLITRYDDCMAFLKDGRITRDVKRVMPKELIAKLNVSEDIDFVSEHMLAKDPPDHSRLRSLVHQGFTPRMIEQLRTGIERITEELLDEMETKADPDIMRDFAAPLPFIVISELLGIPKEDRAKFQVWTSAMVDTSESGQDATNQALREFKQYMQTLIEEKRNHPGEDLTSKLIYAEEDGQKLSESELYSMLFLLVVAGLETTVNLLGSGTLALLLHKDQLENIKRQPDTIQTAGEELLRYTSPVIMMANRWAIEDFTYKDVSIKKGDMIFIGIGSANRDPEYFDEPDTLNVARTPNRHISFGFGIHFCLGAPLARMEASIAFNALLKRFPNIELNGSSEDLTWRKNVFLRGLETLPVKF

>CYP107CB1(ABC0972)Bacillus clausii

MKPTSSEPIDLFSDQFHQQPYTYYKDIREQTGFAKVMLPYGIPAWMAFHYDVAEAVLKDERFIKDARTVFPDEVSDEQMLPISKSMLFVDPPDHKRLRGLIQKGFTPKRISRLKGRIDAIAMEQARRIKQKKRFDLVEEYAFPIPIIVICELLGIPDSDRDKFQYWSKLIVDLDNDGYGESSTVQEGMNDFLAYLQALIHARRQDPREDLLSDLIRAEEDGDRLTTNELYGVVMLLIVAGHETTVNLIANGMLALLMHPDQLALLKNDDQLIPQAVEELLRYNSPVEFSTDRWARESFSFMGKDIKKGDFVIVSLASANHDEALVEHPDKLDITREKSPHLSFGKGIHYCLGAPLARLEAESAIRVLLEECPDIRLGAEPAELAWRQSLIIRGLENLPVETG

>CYP107CB2(BleG1_0965)Bacillus lehensis

MSIVLNKKEFHQEPYEFYKEIRPHDAFAKVKLVSGIQDSWVAFTYEAAEAVLKDERFVKNPRAVFPDVSEHELMPITHSMLFADPPDHRRLRSLVQRGFTPKMIQRLQGRIEEIAKIQVEQMKGKETVDLIADYAFPIPIIVICELLGVPPEDRLDFQRWSNSMVEINDDPSFYEQVEAHMKEFQLYIEQLLAEKRIHPQDDLLSELIRAEEDGDKLSVQELYGAIMLMIVAGHETTVNLIANGMLALFTHPEQLKKLKESPSLIDGAIEEILRFNGPVEFSTDRYAKESFTFMGKQLQKGDHVLVSLASADHDPAVFSEPDKLVITREKSPHLAFGKGIHYCLGAPLARLEGKIAIQTLLNTFPEIQINTALANLEWRQSFVIRGLKEPAGEIELMFEKHVPSLKAQVF

>CYP107CB3(BC359_11355)Bacillus flexus

MNIKSLDLFSQKFHQNPYEYYEQIRLHEPFAKVKLYTEAPDSWMAFTYEAAEAVLKDERFIKDMRTVFPGEMTDENVPPISQTMLFADPPNHRRLRSLVQSGFTPKKIMSLSGRIEEIAREQAKRMKEHETVDFIETYAFPIPIIVICELLGVPQEDQLDFQRWSNVLVDINEASQYDEVLMEFMMYLEKLIHNRRQSPQNDLLSHLIQAEEDGDKLTATELYGVVMLLIVAGHETTVNLISNGLLALLTHPDQLALLKNDPSLISKAIDEFLRYNGPVEFSTDRWARESFTFMGQQVQKGDHVIVSLASTGRDPEVFSHPEKLDITREKNPHLAFGKGIHYCLGAPLARLEGEIAIRVLLEEFPNVGLVANLSDLEWRQSFIIRGLKELPIRLA

>CYP107DE1_ortholog(BS614_18400)Paenibacillus xylanexedens

MNNSNNQTSEKVEFNLFSEENSKDPFAMFAQMRSKGSVVPIPNPMGGAGQTWIVTRMDVAMEVLKDHSRFTVDMNSIDSGNDIRKNLSGELGSSEPQTFFTGKSMLFVDEPDHRRLRSLVSKAFTPRYMESLRPRVQEIADELIDQFEGKGEMDLVKDYAYPFPINVISEMLGIPQEDRPQIHVWSEAIAKGLGFGKQDPAVAQHLRSFAEYTSQLVANKRLEPSDDLISQLIAIEEEGDRLNEDELISMITLLIFAGHETTSNLIATGSYLLLTHPEQLEQLKQDLNLVPSAVEELLRYNGPATSSGPRYATQDTELDGQLIQKGDVVIPLLKSANRDEHQFTDPEDLDIERKIKRHLAFGHGIHMCLGAPLARVEGDVAFTTLLRRLPDIQLSIPQEEIQWHSALSSQGLAALPVKF

>CYP107DE3(BEH_08165)Bacillus endophyticus

MENSNQRLPEKEVLGLLSGKNGEDPFPLFAQLREIGPVISISSPLPGTNQQAWLVTRMEEAMQLLKDHTHFTVDQSTIEGQVDIRKGNSDASAPPTFLTGKSMLSVDNPDHKRLRRLVSKAFTPRYMESLRPRVQKIADELLDQVINKGEMDIVKDYAYPLPINVISDMLGVPQSDQGKIHKWSSAIAHGLGLGRKEPGVAEQIKAFGEYIAKLVEDKRHHPSDDLISQLIAIEEEGDKLSIEELLSMISLLIFAGHETTSNLIANGTLILLDRPEQLEKLKENADLVPSAVEELLRFNGPATIAGPRFATEDIEFAGQQMSKGDMVIPILKSANRDELKFADSEDLDITREIKQHLAFGHGIHSCLGAPLARIEGDVAFSTLLKRMPNLRLSVPRGNVNWEFTLSSQGLSSLPVAF

>CYP107DF1_ortholog(BS614_20390)Paenibacillus xylanexedens

MNPNESNESSKPAFFTKEFTHNPYPVYEMLRKEEPVFRLMFPHGEFGWIITRYEDAVQILKDPRFSKDMVRRYGADNQSIFSNNMLFSDPPDHKRLRGLVQKAFTPKLVADMRSHIQDIADELLDNLPSQEKMNLIDDFAFPLPIIVISEILGVPLEDRDKFRIWSNTIIDASIAKSAELFEQHAREFTDYLTAWFAKVRQDPGTDLISQLVIAEESGQQLTERELLGVVSLLIIAGHETTVNLIGNGILALLEHPEQRELLINQPELIHNAIEEMLRYNGPVEFSTSRWALEDIEFRGERIAQGDLVIVALDSANRDEQQFKDADIFDITREKSSHLAFGKGIHLCLGAPLARLEGEIAISTLLNRFPNIQLQADVNELEWRPGMNIRGVKEIPVQLK

>CYP107DF3(AUC31_06450)Planococcus rifietoensis

MDLHDKDKTQLFSPEFTRNPYPAYERLREEDPVHQVRFPDGQLGWLVTRYTDAEAVLKDPRFIKDFSKLFGGSMDEMSVFTQNMLFSDPPDHKRLRGLAQKAFTPKMIDSMKPRIQEITDELLDGFEGKSNVNLIDEFAFPLPIIVICEILGVPSQDRDKFRTWSNSLIEGTSGEAGVSVYEHMNEFVRYLGEWFQKVRQQPGDDLISRLIEAEEAGDRLTEKELYGLVSLLIIAGHETTVNLIGNTVLTLLKHPEQQHELCVQPELIGPAIEESLRLNGPVEFSTSRWAAEDMEFGGKTIHRGDLVIVALNAANHDPEQFDNPELFDIHREKSAHLAFGKGIHFCLGAPLARLEGHTAIDGLLKRFPGMALAVPENELEWRPGMIVRGVRELPVTLGN

>CYP107DF3_ortholog(BBI15_15435)Planococcus plakortidis

MNLHDNDETKLFSPEFTRNPCPAYKRLREEDPVHQVRFPDGQLGWLVTRYADAEAVLKDPRFIKDFSKLFGGSMDEMSVFTQNMLFSDPPDHKRLRGLAQKAFTPKMIDGMKPRIQEITDDLLDGFEGKSSVNLIDEFAFPLPIIVICEILGVPSQDRDKFRTWSNSLIEGTSGEAGVSVYEHMNEFVRYLGEWFQKVRQQPGDDLISRLIEAEEAGDRLTEKELYGLVSLLIIAGHETTVNLIGNTVLTLLNHPEQQQELCIQPELIGQAIEESLRLNGPVEFSTSRWASEDLEFGGKTIHRGDLVIVALNAANHDPEQFDNPELFDIHREKSAHLAFGKGIHFCLGAPLARLEGQIAIDGLLKRFPGMALAVPENELDWRPGMIVRGVRELPVTLGK

>CYP107DF4(BBH88_15935)Planococcus antarcticus

MTSNRAGDAGLFTEQFTQNPYPIYSKMREEDPIHAVRFPDGQNGWMVTRYEDAEKVLKDPRFTKDLSKIYGGSMDQMSVFTQNMLFSDPPDHKRLRGLAQKAFTPKMIAGMRDRIQEITDELLTAMEGRDQVDLIDEFAFPLPIIVICEILGVPSADRDKFRLWSNSMIEGTSGELGATVYQHMNDFVQYLGQWFATVREQPGDDLISSLIQAEEEGDYLSERELYGVITLLIIAGHETTVNLIGNTVLSLLENPKQRKILAQQPELVNQTIEESLRFNGPVEFSTSRWAGESFEFQGIELKKDDLVVVSLNSANHDRDQFAEPELFDIQREKSPHLAFGKGIHMCLGAPLARLEGEIAIASLLDRFPEMVLAVKSHELEWRPGMIVRGVKELPLKLK

>CYP107DF6(GYMC10_2940)Paenibacillus sp. Y412MC10

MNVNESLKNALFTKEFTHNPYPVYEKLRQSDPIMNLQFPDGRHGWLISSYAEAVEALKDGRFSKDVTKAMGGEQTSVFSTNMLFSDPPDHKRLRGLVQKGFTPQRIADMRSHIQEIADGLLDAVSSKDTMNLIDEYAFKLPIIVISEILGVPAEDQDKFRVWSNSIIGASNQEMNEQVVQHMNEFIAYLRDWFAKVREQPGDDMISQLVIAENQGDRLSEQELYGVVTLMIIAGHETTVNLIGNGVLALLEHPEQRKLLQEQPERIHGAIEEMLRYNGPVEFSTSRWAAKDLEFHGVHMKKGDLVVIALNSANRDSIQFEDPDLFDITREKSQHLAFGKGIHLCLGAPLARLEGEIAINTLLRRFPDFELQGNIDELEWRPGMIVRGVKEIPISLRS

>CYP107DF7(B0X71_01895)Paenibacillus mucilaginosus 3016

MNRPDAHSLLSENFTRNPYPVYTKLRETAAIARMRFPDGQQGWLVTRYADAVDVLTDRRFIKDYAKLIGSSMEQMPVFTQNMLFADPPDHRRLRNLAQKAFTPKMISSMRERIQEIADELLDQAGRTSEMNLIDDYAFPLPIIVICEILGIPAEDRDRFRRWSNSLIEGSSSEAGASIYQHMQEFIQYLSGRFALARQHPDSGLISQLIAVEEAGDRLTEKELYGLVTLLIIAGHETTVNLIGNCVLALLSHPEQKRLLQNNPELIGQTIEEALRFNGPVEFSTSRWAGEDVEFRGRKIRKGELVIVSLNSADHDPEKFNTPELFDITRDKSPHLAFGKGIHQCLGAPLARLEGEIAISSFMKRFPEAELKVNEEELEWRPGMIVRGVRELPLLL

>CYP107DF8(N288_13115)Bacillus infantis

MFNGNHQDNPEAYRIFSKEFIQNPYPGYRKLREEQPVYKTVMPDGQTGWVITKYEDAVAALKDKRFIKDFSKLYGGQMDHESIFTENMLFSDPPDHKRLRGLVQKAFTPRMIENMRGRIKEIADQLLDRMENKEEITLIDDYAFPLPIIVISEILGVPTEDQDKFRIWSNSLIEGSNGENWNEIQQHMNEFVKYLGEWFAFLRDNPRDDLISQLINAEEGGDKLTEKELYGVVSLLIIAGHETTVNLIGNGILSLLENPDQLKLLREKPELIHNAVEELLRYNGPVEYSTSRWAIEDVEFQGQTIRKGELVIAALNSANRDDEKFADPDVLDITREKSPHLAFGKGIHLCLGAPLARLEGEIAILALLERFPEIGLNASPDELEWRPGMIVRGVKELPLRVR

>CYP107DF9(AAV35_000625)Salimicrobium jeotgali

MTNHSNSQEADLFTHAFNQNPYPAFHSLQQHAPVHRTLMPDGHYAWIVTRYEDVVTVLKDQRFIKDFTVFLDEESRAQAHQSIFSRNMLFADPPDHKRLRGLVQQAFTPRMIEELRGRITEIANELIDDMQDRSQIDLIDDFAFPLPIIVICEMLGVPSEDRDKFRTWSNTLVEASNDPEKAEKVQQHMQEFTAYLQEWMTSRQENPQDDMISKLIQAEESGDRLSEQELYGVVSLLIIAGHETTVNLISNGMYALMTHPEQMRKLQEQPGLLRPAIEEMLRFEGPVEFSTDRWASETVELQGQTIHRGEHVLVALDAANRDPEAFEDPDVFDITRGRSKHLAFGKGMHFCLGAPLARVEAEVSLQALLERFPDLQLSTDASNLEWRPGILMRGLTELPVRLK

>CYP107DY1(BMQ_pBM50008)Bacillus megaterium QM B1551

MKKVTVDDFSSPENMHDVIGFYKKLTEHQEPLIRLDDYYGLGPAWVALRHDDVVTILKNPRFLKDVRKFTPLQDKKDSIDDSTSASKLFEWMMNMPNMLTVDPPDHTRLRRLASKAFTPRMIENLRPRIQQITNELLDSVEGKRNMDLVADFSFPLPIIVISEMLGIPPLDQKRFRDWTDKLIKAAMDPSQGAVVMETLKEFIDYIKKMLVEKRNHPDDDVMSALLQAHEQEDKLSENELLSTIWLLITAGHETTAHLISNGVLALLKHPEQMRLLRDNPSLLPSAVEELLRYAGPVMIGGRFAGEDIIMHGKMIPKGEMVLFSLVAANIDSQKFSYPEGLDITREENEHLTFGKGIHHCLGAPLARMEAHIAFGTLLQRFPDLRLAIESEQLVYNNSTLRSLKSLPVIF

>CYP107DY2(DJ92_399) Bacillus pseudomycoides 219298

MKKITLGDLNSPETMRDPIVYYKPFFEQQEPLFRLDDFYGMGGAWIALRYDDVVTILKDPRFLKDLRKFEPPQDKQEFIEENTSVSKLFEWMMNMPNMLTVDPPDHTRLRRLVSKAFTPRIIENLHPRIQQIADELLDAVKEKGKMDIIADFAYPLPIIVISEMLGIPVTDRNQFRDWTHKLMTAAMDPRQGAAVTATLEKFIHYIETLLAEKRVNPSDDLMSALVQAQEKEDKLSKNELLSTIWLLIIAGHETTVNLIGNGVLALLQHPEQMDLLRDNPSMLSSAVDELLRYAGPIMIGNRFAGEDITIHGKIIHKGEMVLFSLAAANIDPQKFSNPEALDITREENDHLAFGNGIHRCLGAPLARLEGQIAFGTLLQRLPNLRLATKPEQLVYNYSKFRSLATLPVVF

>CYP107DY3(CY96_28480)Bacillus bombysepticus

MKKLTFNDLNSPETMRNPIMFYKNLMEQQERFFRIDDFYGMGGAWVAFHYDDVVAILKDSRFIKDLRKFTPPRDKQNPIEENTAVSKLFEWLMNMPNMLTVDPPDHTRLRRLVSKSFTPRMIEDLRPRIQQIADELLDVVQEQGKMEIIADFAYPLPIIVISEMLGIPTNDRNQFRAWTQELMNASVDPGQGTAVTATLEKFINYIEVLFNEKRLNPSDDLISALVQAKEQEDKLSKNELLSTIWLLIIAGHETTVNLISNGVLALLQHPEQMNLLGQNPSLLPSAVDELLRYAGPIMFSSRFASEDVTIHGNRIRKGELVLLSLTAANIDPNIFPYPEELNISREENNHLAFGAGIHHCLGAPLARLEGQIALGTLLKRLPNLRLAIEADQLIYNHSKIRSLAKLPVIF

>CYP107DY3_ortholog(BCK_21505)Bacillus cereus FRI-35

MKKLTFNDLNSPETMRNPIMFYKNLMEQKERFFHIDDFYGMGGAWVVFHYDDVVAILKDSRFIKDLRKFTPPHYKQNPIEENTAVSKLFEWLMNMPNMLTVDPPDHTRLRRLVSKSFTPRMIEDLRPRIQQIADELLDVVQEQRKMEIIADFAYPLPIIVISEMLGIPVTDRNQFRAWTQELMKASVDPGQGTAVTATLEKFINYIEVLFNEKHLNPSDDLISALVQAKEQEDKLSKNELLSTIWLLIIAGHETTVNLISNGVLALLQHPEQMNLLRQDPSLLASAVDELLRYAGPIMFSSRFASEDVKIHGNRIRKGELVLLSLTAANIDPNIFPYPEELNISREENNHLAFGAGIHQCLGAPLARLEGQIALDTLLKRLPNLRLAIEADQLIYNHSKIRSLASLPVIF

>CYP107DY3_ortholog(BcrFT9_02103)Bacillus cereus FT9

MKKLTFNDLNSPETMRNPIMFYKNLMEQKERFFHIDDFYGMGGAWVVFHYDDVVAILKDSRFIKDLRKFTPPHYKQNPIEENTAVSKLFEWLMNMPNMLTVDPPDHTRLRRLVSKSFTPRMIEDLRPRIQQIADELLDVVQEQRKMEIIADFAYPLPIIVISEMLGIPATDRNQFRAWTQELMKASVDPGQGTTVTATLEKFINYIEILFNEKHLNPSDDLISALVQAKEQEDKLSKNELLSTIWLLIIAGHETTVNLISNGVLALLQHPEQMNLLRQDPSLLASAVDELLRYAGPIMFSSRFASEDVTIHGNRIRKGELVLLSLTAANIDPNIFPYPEELNISREENNHLAFGAGIHQCLGAPLARLEGQIALDTLLKRLPNLRLAIEADQLIYNHSKIRSLASLPVIF

>CYP107DY3_ortholog(BCE_2696)Bacillus cereus ATCC 10987

MKKLTFNDLNSPETMRNPIMFYKNLMEQKERFFHIDDFYGMGGAWVVFHYDDVVAILKDSRFIKDLRKFTPPHYKQNPIEENTAVSKLFEWLMNMPNMLTVDPPDHTRLRRLVSKSFTPRMIEDLRPRIQQIADELLDVVQEQRKMEIIADFAYPLPIIVISEMLGIPATDRNQFRAWTQELMKASVDPGQGTTVTATLEKFINYIEILFNEKHLNPSDDLISALVQAKEQEDKLSKNELLSTIWLLIIAGHETTVNLISNGVLALLQHPEQMNLLRQDPSLLASAVDELLRYAGPIMFSSRFASEDVTIHGNRIRKGELVLLSLTAANIDPNIFPYPEELNISREENNHLAFGAGIHQCLGAPLARLEGQIALDTLLKRLPNLRLAIEADQLIYNHSKIRSLASLPVIF

>CYP107DY4(YBT1518_14610)Bacillus thuringiensis YBT-1518

MKKLTFKDLNSPETMRNPIMFYKNLIKQQERFFQIDDFYGMGGTWVVLHYDDVITILKDARFIKDLRKFTSPHDKQHPNSENTAASKLFDWLMNMPNMLTVDPPDHTRLRRLVSKAFTPRMIEDLRPRIQQIADGLLVAVQEQGKMEIISDFAYPLPIIISEMLGIPVTDRNQFREWTQILMNASVNPSQGTAVTTTLEKFIQYIEVLLNEKRLNPDADLISKLVQTKDQEDKLSNNELLSTIWLLIIAGHETTVNLISNGVLALLQHPEQMNLLRENPSLISSTVDELLRYSGPVMFISRLASEDITIHGKRIRKGDLVLLSLTAANIDPQKFTYPETLNISREENNHLAFGAGIHHCLGAPLARLEGQIALGTLLQRLPNLRLAIKPDQLNYNHSKIRSLVNLPVVF

>CYP107DY4_ortholog(BCQ_PI030)Bacillus cereus Q1

MVKRHVISDHSIVIMIRRILVKKLTFKDLNSPETMRNPIMFYENLIKQQERFFQIDDFYGMGGTWVALHYDDVITILKDSRFIKDLRKFTSPHDKQHPNSGNTAASKMFDWLMNMPNMLTVDPPDHTRLRRLVSKAFTPRMIEDLRPRIQQIADELLVAVQEHGKMDIISDFAYPLPIIVISEMLGIPATDRNQFREWTQILMNVSVNPSQGNAVTTTLEKFIQYIEVLLNEKRLNPAADLISELVQTKEQEDRLSNNELLSTIWLLIIAGHETTVNLISNGVLSLLQHPEQMNLLRENPSLIPSAVDELLRYSGPVMFISRLASEDMTIHGKRIRKGDLVLLSLTAANIDPQKFSYPEELNISREENNHLAFGAGIHHCLGAPLARLEGQIALGTLLQRLPNLCLAIKPDQLNYNHSKIRSLVNLPVVF

>CYP107JF1(BS614_20220)Paenibacillus xylanexedens

MFTNGNKGHSKSGVDFLDKSFINTPFPVYEELRNEDPVHRFLLPSGHFAWMITRYEDAVEILQDKRFVTNPPFMGNVEGSAPPHQEIISRNLLSVNPEDHRRLRRLIQKAFTPRMIERLRGRIVEISDELLDKVLASGKRELDLIEDYAFPLPIMVICEMLGVPLEDQDKFQEWSNTIMEGFNNPQMNEKSDEVMKAFVDYLHVLITERRQHLQEDLISDLISVEEEGDVLSEQELYALVFVLIIAGHETTVNLIGNGMLALLDNPEQKQLLLNDPELIHPALEEILRYNGPAEVSNIRWATEDVEYGGKHIRQGEMLFVSFSSANRDQQQFPEPDTFDITRKVNNHIAFGKGIHFCLGAPLARLEGEIAINALLRKMPDIRLNTTEESLEWRPGMIIRGLKDFPVSF

>CYP107JF2(X809_28690)Paenibacillus polymyxa CR1

MFKNENSNQAGLKVDFLDQPFIQNPFPVYEKLRADEPIHRFFLPGGQYAWIVSRYEDAVDILQDKRFVTSHPHHGMAGEQAPHREIILQNLISVGPEDHRRLRRLIQKAFTPRMIERLRGRIEEISDDLLNKILASGKREIDLIEEYAFPLPLIVICEMLGVPIEDQDKFQLWSNTIMESVSNPQMGQESDQVMKEFVDYLHALITERRKEIKEDLISDLINVEEAGDKLTEKELYALVFVLIIAGHETTVNLIGNGILALLENPEQKQLLMNDPDLIQPAVEEILRFNGPAEVSNVRWATEDVELEGNQIRQGDMMFIALSSANRDANKFDEPNTFDITRKINDHIAFGKGVHYCLGVPLARLEGEIAIQDLLHKLPKIQLNTNIDLLEWRTGMIIRGLKTLPISF

>CYP107JF3(BGLY_3077)Bacillus glycinifermentans

MSKIDFLNKEFIRNPFPVYSEVREQEPVYRFLLPSGHYAWIVTRYDDAVKVLSDSKFIANPLHLNEADDPAEPPHKKIISRNLLSVNPADHRRLRRLVQKAFTPRMVERLRGRIEEIANELLDNVQDKGEMNLIEDYAFPLPIIVICEMLGIPHEDQDTFRKWSDVIMEGVNNPHFEQQSEEVMIAFVDYLRDLIARRRKELKEDLISDLISVEVEGDVLSEHEMYALVFVLIIAGHETTVNLIGNGILALLHHPDQKAKLQNQPELIHSAIEEMLRYDGPAEVSNIRWATEDAKLGGKTIRKGEMLFISFSSANRDPEKFADPDSFNITREANNHLAFGKGVHHCLGAPLARLEGEIAIGTLLRRMPDLRLKTNADFLEWRPGMIIRGLKEIPLLF

>CYP107JF4(PM3016_4289)Paenibacillus mucilaginosus 3016

MSIPDHEAPQLDLFSSEFKRNAYSLYAQLREQEPVYRFMLPNGQTAWLITRYEDAQAALKDSRFIKNPHTLLSQEQMDKWTFPMLERDLLIRNMVASDPPDHTRLRNLVQKAFTPKMIEELRGRIQEIADTLLDEIQDKGNMNVMDDFAFPLPIIVICEMLGIPAEDRDSFRDWSNAIVRAVHLPEKIQEALPQIRSFIKYMGQLIEERRNDPDEDLVSSLIQAETEGEQLTENELYSMIFLLIVAGHETTVNLIGNGVFALLQHPEQWEKLKSHPGLISSAVEEILRFYGPVELASSRWAGEDVPFHEQLIRKGDMIAVALASANRDEAQFIHPDRFDITRKNNRHLAFGTGIHHCLGAPLARLEGQIALLTLLRRMPNLRLSTDPGTLQWVPAYVMRGLVELPVQF

>CYP107JF4_ortholog(KNP414_04885)Paenibacillus mucilaginosus KNP414

MMRKGDQAMSIPDHEAPQLDLFSSEFKRNAYSLYAQLREQEPVYRFMLPNGQTAWLITRYEDAQAALKDSRFIKNPHTLLSQEQMDKWTFPMLERDLLIRNMVASDPPDHTRLRNLVQKAFTPKMIEELRGRIQEIADTLLDEIQDKGNMNVMDDFAFPLPIIVICEMLGIPAEDRDSFRDWSNAIVRAVHLPEKIQEALPQIRSFIKYMGQLIEERRNDPDEDLVSSLIQAETEGEQLTENELYSMIFLLIVAGHETTVNLIGNGVFALLQHPEQWEKLQSHPELISSAVEEILRFYGPVELASSRWAGEDVPFHEQLIRKGDMIAVALASANRDEAQFIHPDRFDITRKNNRHLAFGTGIHHCLGAPLARLEGQIALLTLLRRMPKLRLSTDPGTLQWVPAYVMRGLVELPVEF

>CYP107JF4_ortholog(B2K_22235)Paenibacillus mucilaginosus K02

MSIPDHEAPQLDLFSSEFKRNAYSLYAQLREQEPVYRFMLPNGQTAWLITQYEDAQAALKDSRFIKNPHTLLSQEQMDKWTFPMLERDLLIRNMVASDPPDHTRLRNLVQKAFTPKMIEELRGRIQEIADTLLDEIQDKGNMNVMDDFAFPLPIIVICEMLGIPAEDRDSFRDWSNAIVRAVHLPEKIQEALPQIRSFIKYMGQLIEERRNDPDEDLVSSLIQAETEGEQLTENELYSMIFLLIVAGHETTVNLIGNGVFALLQHPEQWEKLKSHPELISSAVEEILRFYGPVELASSRWAGEDVPFHEQLIRKGDMIAVALASANRDEAQFIHPDRFDITRKNNRHLAFGTGIHHCLGAPLARLEGQIALLTLLRRMPNLRLSTDPGTLQWVPAYVMRGLVELPVQF

>CYP107JF4_ortholog(ERIC2_c15090)Paenibacillus larvae

MATNSTVLPINPFSPDFKNQAYALYEKLRENDPIHKITLPNGKTGWVVTRYKDAAATLKKERLTKNLFQFMHSEDVGLPQKQMNLMFKHMLNTDQPDHTRLRSLVQKAFTPRMIEKLNGRVQEISDSLIDKVESREDMELIQDYAYPLPIIVICEMLGLPSEERDQFRKWSNALVSSMNVPKKYKQIVPDTIAFTNYIKSLIERRRQDPKEDLLSLLTQAESENGKLSEMELVSMIFLLIIAGHETTVNLIGNGTFTLLQHPEQLEELRRTPSLIGSAIEELLRFMGPVEFATNRWAGEGFEWEGKAISKGDIVLVGLASANRDPESFKHPERLDLTRENNNHLAFGMGIHHCLGAPLARMEGRIAINTLLRRLPNLKLAVSPDCLKWQPSYLMRGFDALPLRFR

>CYP107JG1(HBHAL_3372)Halobacillus halophilus

MENTETIYRSNFKIGAYTFYQDLRLNQPVFPMSSGGSNSSWIITKYDHVKELLKSPSFIKDQNKLFSQSSREEMNADELNIFQNMMLDVDPPDHTRLRKLVQPYFNPKTIKQLEPRITEIAQELLEEMKHKKGSVDLIDDYAFPLPIIVISELLGVPVEDRNKFRKWSNTIVAASDNMTADFQEDVEAFTAYLTSLFEKRKAEPEEDLISSLLQTAEEEEQLTKAELYSMVVLLIIAGHETTVNLIGNTMFALFEHPEQMSKIKFDQTLIPSAIEEGLRFYSPVDFSTARWAEEDMEFHGQEIRGGDLVLASISSANRDEEKFSHAEQFDVTRSKNAHVAFGFGIHFCLGAPLARLEGKVAIEELLSAFPNIDLNRTMNRPEWKPVFLLRGLSQLPVALN

>CYP107JG1_ortholog(HM131_11250)Halobacillus mangrovi

MENIKAIYQSNFKENVYPFYTHLRANDPIFAMSQEPNHTTWVITKHEHVKEMLKSKSFIKDQRKLFSNNQSHEKQDNGEINIFQNMMLDVDPPDHTRLRKLVQPYFNPKTIKELKPRIIEIADELLDQASEKQGPVDLIDEFAFPLPIIVISELLGIPSEDRNKFRKWSNTIVAASDEFEPDFMEDVQAFTNYLTDLFERRRKSPENDFISSLLQAEEDGEQLNRDELYSMVVLLIIAGHETTVNLIANTMYALFEHPDQLEKLKTDTKLTATAIEEGLRYYSPVDFTTARWSEDDMEFHGKTIKRGDLVLASLASANRDEEKFEAPDTFDITRKPNPHVAFGYGIHFCLGAPLARLEGEIALRKLMERFPNISMVENQTSSPWRPVFLLRGLEGLKVTIV

>CYP107JH1(PM3016_2832)Paenibacillus mucilaginosus 3016

MSLDVGLDMSSPEFKNRAYALYDRWRVEEPVHSMGTTGGQQAWLITRYDDAVEALKNEERFTKNNRSLQAPGEPGLLPDKVFGLINNNLLGSDPPDHTRLRSIVSKAFTPGVAESLRGQIEEITGRLIGELRQKGGGDLIDEFAFPLPIYVICQMLGIPPEDHALFRKWSDDFIAAATDIEKIRGVQSSLEAFASYVNGLIEERKAKPQADLITELIRVQENGDRLTREELSSTIFLLIVAGHETTVNLIGNGTLALLEHPDQLALWRGNPDLDSTAVEELLRFYGPVEISTNRWVREPFEWHGKQLQRGDLLFVSLASANRDPSQFEDPARLDLTRKKNRHIAFGSGIHFCLGAPLARLEGSIAISTLIRELPNLRFVVPREEIHWRPGRLMRGLQRLPVLV

>CYP107JH1_ortholog(B2K_14395)Paenibacillus mucilaginosus K02

MSLDVGLDMSSPEFKNRAYALYDRWRVEEPVHSMGTTGGQQAWLITRYDDAVEALKNEERFTKNNRSLQAPGEPGLLPDKVFGLINNNLLGSDPPDHTRLRSIVSKAFTPGVAESLRGQIEEITGRLIGELRQKGGGDLIDEFAFPLPIYVICQMLGIPPEDHALFRKWSDDFIAAATDIEKIRGVQSSLEAFASYVNGLIEERKAKPQADLITELIRVQENGDRLTREELSSTIFLLIVAGHETTVNLIGNGTLALLEHPDQLALWRGNPDLDSTAVEELLRFYGPVEISTNRWVREPFEWHGKQLQRGDLLFVSLASANRDPSQFEDPARLDLTRKKNRHIAFGSGIHFCLGAPLARLEGSIAISTLIRELPNLRFAVPREEIHWRPGRLMRGLQSLPVLV

>CYP107JH1_ortholog(KNP414_02585)Paenibacillus mucilaginosus KNP414

MSLDVGLDMSSPEFKNRAYALYDRWRVEEPVHSMGTTGGQQAWLITRYDDAVEALKNEERFTKNNRSLQAPGEPGLLPDKVFGLINNNLLGSDPPDHTRLRSIVSKAFTPGVAESLRGQIEEITGRLIGELRQKGGGDLIDEFAFPLPIYVICQMLGIPPEDHALFRKWSDDFIAAATDIEKIRGVQSSLEAFASYVNGLIEERKAKPQADLITELIRVQENGDRLTREELSSTIFLLIVAGHETTVNLIGNGTLALLEHPDQLALWRGIPDLDSTAVEELLRFYGPVEISTNRWVREPFEWHGKQLQRGDLLFVSLASANRDPSQFEDPARLDLTRKKNRHIAFGSGIHFCLGAPLARLEGSIAISTLIRELPNLRFAVPREEIHWRRGRLMRGLQSLPVLV

>CYP107JJ1(LIP_1054)Limnochorda pilosa

MADAAVLFDPNSEALLQERYSVYRLMRERAPVYEVPGFGFPVRMLFRYDDVGAVLKSPAFVRQARNAGRELPVREEMLPIQQIFATWMLLRDPPDHTRLRALVSKAFTPRTVQEMAGFIQKTAEALLDRVAGQSTFDLLRDFASPLPVMVIARLLGAPDEDRELFRGWAQLIAAFLGAWSIDDVPPESPRAALEMAGYMRRLLDERRRSPREDLLSALLAAEEDGNRLTHDEVIGTAIMLLTAGHETTVNLIANGTYALLKNPDQVERLRRSPELLPLAVEELLRYDSPVQMTARHCARPAEVGGVPFERGTGVFLVLGSANRDPGSFPDPDQLDVGRTPNNHLAFGHGIHYCVGAGLARLEAEIAFASLLPRLPVLALREEPRYVANMAFRALEALPVAWRA

>CYP109A1_ortholog(BSUW23_09845)Bacillus subtilis subsp. spizizenii W23

MTNQTARSSKKERYANLIPMEELHSEKDRLFPFPIYDKLRRESPVRYDPLRDCWDVFKYDDVQFVLKNPKLFSSKRGIQTESILTMDPPKHTKLRALVSRAFTPKAVKQLETRIKDVTAFLLQEARQKSTIDIIEDFAGPLPVIIIAEMLGAPIEDRHLIKTYSDVLVAGAKDSSDKAVADMVHNRRDGHAFLSDYFRDILSKRRAEPKEDLMTMLLQAEIDGEYLTEEQLIGFCILLLVAGNETTTNLIANAVRYLTEDSVVQQQVRQNTDNVANVIEETLRYYSPVQAIGRVATEDTELGGVFIKKGSSVISWIASANRDEDKFCKPDCFKIDRPSYPHLSFGFGIHFCLGAPLARLEANIALSSLLSMSACIEKAAHDEKLEAIPSPFVFGVKRLPVRITFK

>CYP109A1_ortholog(GYO_2222)Bacillus subtilis subsp. spizizenii TU-B-10

MTNQTARPSKEERYANLIPMEELHSEKDRLFPFPIYDKLRRESPVRYDQQRDCWDVFKYDDVQFVLKNPKFFSSKRGIQTESILTMDPPKHTKLRALVNRAFTPKAVKQLETRIKDVTAFLLQEARQKSTIDIIEDFAGPLPVIIIAEMLGAPIEDRHLIKTYSDVLVAGAKDSSDKAVADMLHNRRDGHAFLSDYFRDILSKRRTEPKEDLMTMLLQAEIDGEHLTEEQLIGFCILLLVAGNETTTNLIANAVRYLTEDSVVQQQVRQNTDNIANVIEETLRYYPPVQAIGRVAAEDTELGGVFIKKGSSVISWIASANRDEDKFCEPDCFKIDRPSYPHLSFGFGIHFCLGAPLARLEANIALSSLLSMSACIEKADHDEKLEAIPSPFVFGVKRLPVRITFK

>CYP109A2_ortholog(BMD_2035)Bacillus megaterium DSM 319

MNPKAVKRENRYANLIPMQEIKSVEQQLYPFDIYNSLRQEAPIRYDESRNCWDVFDYETVKYILKNPSLFSSKRAMEERQESILMMDPPKHTKLRNLVNKAFTPRAIQHLEGHIEEIADYLLDEVSSKEKFDIVEDFAGPLPIIVIAELLGVPIQDRALFKKYSDDLVSGAENNSDEAFAKMMQKRNEGVIFLQGYFKEIIAERQQNKQEDLISLLLEAEIDGEHLTEEEVLGFCILLLVAGNETTTNLITNGVRYMTEDVDVQNEVRRDISLVPNLVEETLRYYPPIQAIGRIAAEDVELGECKIKRGQQVISWAASANRDSAKFEWPDTFVVHRKTNPHVSFGFGIHFCLGAPLARMEGKIAFTKLLEKGGFSKVQNQSLKPIDSPFVFGVKKYEIAFNNA

>CYP109A2_ortholog(BG04_4382)Bacillus megaterium NBRC 15308 = ATCC 14581

MNPKAVKRENRYANLIPMQEIKSVEQQLYPFDIYNSLRQEAPIRYDESRNCWDVFDYETVKYILKNPSLFSSKRAMEERQESILMMDPPKHTKLRNLVNKAFTPRAIQHLEGHIEEIADYLLDEVSSKEKFDIVEDFAGPLPIIVIAELLGVPIQDRALFKKYSDDLVSGAENNSDEAFAKMMQKRNEGVIFLQGYFKEIIAERQQNKQEDLISLLLEAEIDGEHLTEEEVLGFCILLLVAGNETTTNLITNGVRYMTEDVDVQNEVRRDISLVPNLVEETLRYYPPIQAIGRIAAEDVELGECKIKRGQQVISWAASANRDSAKFEWPDTFVVHRKTNPHVSFGFGIHFCLGAPLARMEGKIAFTKLLEKGGFSKVQNQSLKPIDSPFVFGVKKYEIAFNNA

>CYP109A2_ortholog(BMQ_2078)Bacillus megaterium QM B1551

MNSKAVKRENRYANWIPMQEIKSVEQQLYPFDIYNSLRQEAPIRYDESRNCWDVFDYETVKYILKNPSLFSSKRAMEERQESILMMDPPKHTKLRNLVNKAFTPRAIQHLEGHIEEIADYLLDEVSSKKKFDIVEDFAGPLPIIVIAELLGVPIQDRALFKKYSDDLVSGAENNSDEAFAKMMQKRNEGVIFLQGYFKEIIAERQQNKQEDLISLLLEAEIDGEHLTEEEVLGFCILLLVAGNETTTNLITNGVRYMTEDVDVQNEVRRDISLVPNLVEETLRYYPPIQAIGRIAAEDVELGECKIKKGQQVISWAASANRDSAKFERPDTFVVHRKTNPHVSFGFGIHFCLGAPLARMEGKIAFTKLLEKGEFSKVQNQSLKPIGSPFVFGVKKYEITFNKA

>CYP109A2_ortholog(BMWSH_3183)Bacillus megaterium WSH-002

MNPKAVKRENRYANLIPMQEITSVEQQLYPFDVYNSLRQEAPIRYDESRNCWDVFDYETVKYILKNPSLFSSKRAMEERQESILMMDPPKHTKLRNLVNKAFTPRAIQHLEGHIEEIADYLLDEVSSKKKFDIVEDFAGPLPIIVIAELLGVPIQDRALFKKYSDDLVSGAENNSDEAFAKMMQKRNEGVLFLQGYFKEIIAQRQRKKQEDLISLLLEAEIDGEQLTEEEVLGFCILLLVAGNETTTNLITNGVRYMTEDLDVQNEVRQDISLVPNLVEETLRYYPPIQAIGRIAAEDVELGECKIKKGQQVISWAASANRDSVKFERPDTFVVHRKTNPHVSFGFGIHFCLGAPLARMEGKIAFTKLLEKGEFSKVQNQSLKPIDSPFVFGVKKYEIAFNNA

>CYP109A3_ortholog(BS614_13945)Paenibacillus xylanexedens

MKQAPRKYANYIPIRELETLEERLSPFKVYAELRDNTPVRYDEHRECWDVFGYEDVKYVLKNPKLFSSARDRANTSMLTTDPPKHKQLRDLVNQAFTPKAIEALAPRIQEITDELIAPHLSEGHMELIDDLATPLPVIVIAELIGVPAADRQKFKDWSDVLVKGARDDSEQAFQELLEEKKRNMQELHIYFTGIMEERRLQPKDDLISLLLAAEIDGQQLTSDEVVGFCILLLAAGNETTTNLITNAVRILSEQPELQQELREQPERITSAIEETLRYYPPIVAIGRVAKETLELNGQTIQAGEQVISWVGAANRDSAQFENPEDFISDRKPNRHMGFGFGIHFCLGAPLARLEARVVLHTLLQHMEHIQLVPGTALQPIQSAFVFGVKHYPIEFNLINK

>CYP109A4(AR543_20365)Paenibacillus bovis

MKKISNKYANYIPMRELNTVEHQLFPFDVYNELLEQSPVRYDENRSCWDVFRYEDVQYVLKNPKLFSSRRNRANRESMLTQDPPKHKQLRDLVNQAFTPRKIEELAPHIQNIADDLLAPHLQQGQLQLVYDLATPLPVIVIAELLGVPSSDRDRFKEWSDILVKGARNDSDEAFQELMEEKDRTNAELGAYFGRIIQERRQSAQDDLISLLIAADIEGEHLTEDEIISFCILLLAAGNETTTNLITNAVRILAEQPELQQQLREHPEMIPTAVEETLRYYPPIVAIGRVAKEDIELRGQQIREGEQVISWVGAANRDEQKFANSDTFIPDRKPNQHMGFGFGIHFCLGAPLARLEGRVVLNTLLRHMDRIALVEGTQLEPIQSAFVFGVKEYPITFQPIQPD

>CYP109A5(BGLY_2392)Bacillus glycinifermentans

MTNSIQTSNRYANWIPMKEISSSKDRLFPFPIYNRLRQTSPVRFDDDRKCWDIFAYEDVQYVLKSPKLFSSKRGGDMQGKTMLTMDPPRHTSMRAIVNKAFTPKAVKQLESHIGEVTAFLLDETRDRRSFDLVDDLAAPLPVIIIAELLGVPPEDRSMFKHYSDILVSGASDSSDEAVKLMSKRRKEGNDYLKDYFKRIIKERKKSPKEDLISLLIQAEVEGEKLTEEELLGFCTLLLVAGNETTTNLIANAVRYLTEDAHTQEAVRHDLSLVPHLVEETLRYYPPVQAIGRVAAQDTEIRGVSIPKGSSVISWVASANRDETKFDDPESFRLDRKSNPHMSFGFGIHFCLGAPLARLEANIALSHLLRHASVERDDRVKLEAIQSPFVFGVRRLPVRLLY

>CYP109A6(BaLi_c22470)Bacillus paralicheniformis

MANSNSLQSSKHYANWIPMKEISSSNDRLFPFPIYNRIRKTSPVRYDDERKCWDIFSYEDVQFVLKNPKLFSSKRGGNMEGKSILTMDPPKHTKMRAIVNKAFTPKAVKELEPHIEEVTAFLFNEAKQKDLFDVVDDLAAPLPVIIIAELLGVPAEDRLMFKHYSDILVAGAEDRSAEAAEQMYKRREEGNRFLADYFKNIIKQRKKEPKDDLISLLLRAEVDGKSLTEEELLNFCIILLVAGNETTTNLIANSVRYLTEDKATQEAVRQDLSLVPVFVEETLRYYPPVQAIGRTAAEDLDIKGVRIAKGSTVINWVASANRDELKFDDPDSFKLDRKSNPHMSFGFGIHFCLGAPLARLEAKVALDYLLRHASVEKDSSEELEAIQSPFVFGVRHLPVRLSQK

>CYP109A6_ortholog(BLi02170)Bacillus licheniformis DSM 13 = ATCC 14580

MANSNSLQSSKHYANWIPMKEISSSNDRLFPFPIYNRIRKTSPVRYDDERKCFDIFSYEDVQFVLKNPKLFSSKRGGNMEGKSILTMDPPRHTKMRAIVNKAFTPKAVKELEPHIEEVTAFLFNEAKQKELFDVVDDLAAPLPVIIIAELLGVPAEDRLMFKHYSDILVAGAEDRSAEAAERMYKRREEGNRFLADYFKNIIKQRKKEPKDDLISLLLRAEVDGKSLTEEELLHFCIILLVAGNETTTNLIANSVRYLTEDKITQEAVRQDPSLVPVFVEEMLRYYPPVQAIGRTAAEDVDIGGVRIAKGSTVISWVASANRDELKFDDPDSFKLDRKSNPHMSFGFGIHFCLGAPLARLEAKVALDYLLRRAYMERDSSKELEAIQSPFVFGVRHLPVQLSQK

>CYP109A6_ortholog(BL01999)Bacillus licheniformis ATCC 14580

MANSNSLQSSKHYANWIPMKEISSSNDRLFPFPIYNRIRKTSPVRYDDERKCFDIFSYEDVQFVLKNPKLFSSKRGGNMEGKSILTMDPPRHTKMRAIVNKAFTPKAVKELEPHIEEVTAFLFNEAKQKELFDVVDDLAAPLPVIIIAELLGVPAEDRLMFKHYSDILVAGAEDRSAEAAERMYKRREEGNRFLADYFKNIIKQRKKEPKDDLISLLLRAEVDGKSLTEEELLHFCIILLVAGNETTTNLIANSVRYLTEDKITQEAVRQDPSLVPVFVEEMLRYYPPVQAIGRTAAEDVDIGGVRIAKGSTVISWVASANRDELKFDDPDSFKLDRKSNPHMSFGFGIHFCLGAPLARLEAKVALDYLLRRAYMERDSSKELEAIQSPFVFGVRHLPVQLSQK

>CYP109A7(BSM4216_1205)Bacillus smithii

MATAKKTSYETKRYANLIPMKEIQSVQDQLFPFTIYNTLRKKTPVRYDPIRECWDVFRYEDVHFILKNPALFSSNRGIGDKRNSILIMDPPKHTKMRNLINKTFTPKAVNELSQRIQDVTTSLLDQAKEKETLEMIRDFAAPLPVIIIAEILGVPAKDRELFKNYSDILVSGAEDDSDEAFNMMMKRRREGAKFLNDYFKEIIQERQRSPKDDLISLLLAAEVDGERLKEEELLKFCILLLVAGNETTTNLIVNAVRYMVEDTNTQETVRKNFSLVPNLIEETLRFYPPIQAIGRTATADVEIGGHTIRKGSQVISWVAAANRDEQKFEQPDRFMLERHPNPHLGFGYGIHFCLGAPLARLEAKVALSVLLTTFSKLELAKHTELEPIQSPFVFGVKSFPIQFSL

>CYP109A8(UP12_09345)Bacillus pumilus SH-B9

MKVKENYINVIPMKEIRSTDDLLFPFPIYNELRAESDIRYDETRKCWDLFRYADIQSVLKQPKVFSSQRGRSTTKTSILTMDPPKHTKMRALVNKAFTPKAIKQLEDNIKDLTHDLLQQVKDQRTFDIVEDLAAPLPVMIIAELLGAEIKDRAFIKKHSDALVAGAKDESKEAIQAVVDMQKRAEEELSAYFAHLIQKRKEAPADDLISLLIQAEIDGERLTENELLGFCILLLVAGNETTTNLITNAVRLLTEQPHIAEAVRQDPSLIPQLTEETLRYYPPVQAIGRIAAEDVEIAGSKIKKGDYLINWVASANRDEQKFDDPDTFRLNRKSNPHMSFGFGIHFCLGAPLARLESNIALEVLLQSYQDITCAADQLKPIQSTFVFGVKEFPVQITRF

>CYP109A8_ortholog(BPUM_1825)Bacillus pumilus SAFR-032

MKVKENYINVIPMKEIRSTDDLLFPFPIYNELRAQSDIRYDETRKCWDLFRYADIQSVLKQPKVFSSQRGRSTTKTSILTMDPPKHTKMRALVNKAFTPKAIKQLEDNIRDLTHDLLQQVKDQRTFDIVQDLAAPLPVMIIAELLGAEVKDRAFIKKHSDALVAGAKDESKEAIQAVVDMQKRAEEELSAYFTHLIQKRKESPTDDLISLLIQAEIDGERLTEDELLGFCILLLVAGNETTTNLITNAVRLLTEQPHIAEAVRQDPSLIPQLTEETLRYYPPVQAIGRIAAEDVEIAGSKIKKGDYLISWVASANRDEQKFDDPDTFRLDRKSNPHMSFGFGIHFCLGAPLARLESNIALEVLLQTFQDITCVADQLKPIQSTFVFGVREFPVQVTRF

>CYP109A8_ortholog(QR42_09290)Bacillus sp. WP8

MKVKENYVNVIPMKEIRSTDDLLFPFPIYNELRAESDIRFDETRKCWDLFRYADIQSVLKQPKVFSSQRGRSTSKTSILTMDPPKHTKMRALVNKAFTPKAIKQLEDKIKDLTHDLLNQVKDQRTFDIVQDLAAPLPVMIIAELLGAEVQDRELIKKHSDALVAGAKDESKEAIQAVVDMQKRAEEELSVYFAQLIKKRKETPADDLISLLIQAEIDGERLTENELLGFCILLLVAGNETTTNLITNAVRLLTEQPHIAESVRQDPSLIPQLTEETLRYYPPVQAIGRIAAEDVEIAGARIEKGDYLIGWVASANRDELKFEDPDTFRLDRKSNPHMSFGFGIHFCLGAPLARLESNIALDVLLHTFQDITCAADQLKPIQSTFVFGVKEFPVQVTRF

>CYP109A9(UP17_09030)Bacillus simplex

MTQEQKSTSAVEQYANLIPMREIDSNADQLFPFPIFNDLRQKSPVRYDESRSCWDVFRYEDVHFILKNPKMFSSERGSGTLQGSILTMDPPRHTKMRNLVNKAFTPKAVKDLTNKIEEVTLYLLDQVKEKGTMDLVHDVAGPLPVIIIAELLGIPTKDRDLFKTYSDVLVEGAKDNSSEAFQRMTQKRKEGNEFLKEYFKKIIKERTNNPEEDLISLLIEAKIDGEKLTEEELLSFCVLLLVAGNETTTNLITNAVRYMTEDKKIQEQARTNLPLIPKLVEETLRFYPPIQAIGRIAKEHVKVGGKTIQKGEQVICWVASANRDEQKFAEPNRFTLERKLNPHLSFGFGIHFCLGAPLARLEAEVALVTLLSTFSKLEDVKGTKLEAIPSPFVFGVKSLPLKFTR

>CYP109A10(BW16_10010)Bacillus pumilus MTCC B6033

MKVKENYVNVIPMKEIRSTDDLLFPFPIYNELRSQGDIRYDETRKCWDLFRYADIQSVLKQPKVFSSQRGRSTSKTSILTMDPPKHTKMRALVNKAFTPKAIKQIEEKIKDLTHDLLHQVKDQRTFDIVQDLAAPLPVMIIAELLGAEMKDRELIKKHSDALVAGAKDDSKEAVQAVVDMQKQAEKELSAYFAHLIQKRKETPADDLISLLIQAEIDGERLSENELLGFCILLLVAGNETTTNLITNAVRLLTEQQHIAESVRQDLSLIPQLTEETLRYYPPVQAIGRVAAEDVQIAGSHIEKGDYIISWVASANRDERKFEDPDTFRLDRKSNPHLSFGFGIHFCLGAPLARLEAHLALEILLRTFREITCAADQLKPIQSTFVFGVKEFPVQVTRF

>CYP109A11(B2K_24915)Paenibacillus mucilaginosus K02

MNTGSPAKYANIIPLKELDTVQKQLYPFDFYREARTQTPVRYNEHRSAWDLFLYADVHRVLKDPAAFSSRRGMEMRTENLLVMDPPRHTQMRDLVNKAFTPKAIGALEEHIRRVTADLLDEAAGRGSMEMVHDLAAPLPVIIIAELLGVPTQDRKLFKEWSDVLVKGVDENTEAAFAKMHAERGQAAKELSLYFSGILKERLTQPQDDLISALLAAEIDGKKLSEEEVISFAILLLVAGNETTTNLITNGVRRLTEDPALQTLLQEQPELVPGFVEEVLRFYPPILAVGRVAAQDVEIGGQTIRTGDQVISWVASANRDERKFDDADAFRPDRKPNPHLAFGFGPHFCLGAPLARLEAKVAAEELLRRFRDLRLADGAELVPIPSPFVFGVKSYPVTFRTQA

>CYP109B1_ortholog(BS34A_13520)Bacillus sp. BS34A

MNVLNRRQALQRALLNGKNKQDAYHPFPWYESMRKDAPVSFDEENQVWSVFLYDDVKKVVGDKELFSSCMPQQTSSIGNSIINMDPPKHTKIRSVVNKAFTPRVMKQWEPRIQEITDELIQKFQGRSEFDLVHDFSYPLPVIVISELLGVPSAHMEQFKAWSDLLVSTPKDKSEEAEKAFLEERDKCEEELAAFFAGIIEEKRNKPEQDIISILVEAEETGEKLSGEELIPFCTLLLVAGNETTTNLISNAMYSILETPGVYEELRSHPELMPQAVEEALRFRAPAPVLRRIAKRDTEIGGHLIKEGDMVLAFVASANRDEAKFDRPHMFDIRRHPNPHIAFGHGIHFCLGAPLARLEANIALTSLISAFPHMECVSITPIENSVIYGLKSFRVKM

>CYP109B1_ortholog(BSU6051_12210)Bacillus subtilis subsp. subtilis 6051-HGW

MNVLNRRQALQRALLNGKNKQDAYHPFPWYESMRKDAPVSFDEENQVWSVFLYDDVKKVVGDKELFSSCMPQQTSSIGNSIINMDPPKHTKIRSVVNKAFTPRVMKQWEPRIQEITDELIQKFQGRSEFDLVHDFSYPLPVIVISELLGVPSAHMEQFKAWSDLLVSTPKDKSEEAEKAFLEERDKCEEELAAFFAGIIEEKRNKPEQDIISILVEAEETGEKLSGEELIPFCTLLLVAGNETTTNLISNAMYSILETPGVYEELRSHPELMPQAVEEALRFRAPAPVLRRIAKRDTEIGGHLIKEGDMVLAFVASANRDEAKFDRPHMFDIRRHPNPHIAFGHGIHFCLGAPLARLEANIALTSLISAFPHMECVSITPIENSVIYGLKSFRVKM

>CYP109B1_ortholog(U712_06325)Bacillus subtilis PY79

MNVLNRRQALQRALLNGKNKQDAYHPFPWYESMRKDAPVSFDEENQVWSVFLYDDVKKVVGDKELFSSCMPQQTSSIGNSIINMDPPKHTKIRSVVNKAFTPRVMKQWEPRIQEITDELIQKFQGRSEFDLVHDFSYPLPVIVISELLGVPSAHMEQFKAWSDLLVSTPKDKSEEAEKAFLEERDKCEEELAAFFAGIIEEKRNKPEQDIISILVEAEETGEKLSGEELIPFCTLLLVAGNETTTNLISNAMYSILETPGVYEELRSHPELMPQAVEEALRFRAPAPVLRRIAKRDTEIGGHLIKEGDMVLAFVASANRDEAKFDRPHMFDIRRHPNPHIAFGHGIHFCLGAPLARLEANIALTSLISAFPHMECVSITPIENSVIYGLKSFRVKM

>CYP109B1_ortholog(B657_12210)Bacillus subtilis QB928

MNVLNRRQALQRALLNGKNKQDAYHPFPWYESMRKDAPVSFDEENQVWSVFLYDDVKKVVGDKELFSSCMPQQTSSIGNSIINMDPPKHTKIRSVVNKAFTPRVMKQWEPRIQEITDELIQKFQGRSEFDLVHDFSYPLPVIVISELLGVPSAHMEQFKAWSDLLVSTPKDKSEEAEKAFLEERDKCEEELAAFFAGIIEEKRNKPEQDIISILVEAEETGEKLSGEELIPFCTLLLVAGNETTTNLISNAMYSILETPGVYEELRSHPELMPQAVEEALRFRAPAPVLRRIAKRDTEIGGHLIKEGDMVLAFVASANRDEAKFDRPHMFDIRRHPNPHIAFGHGIHFCLGAPLARLEANIALTSLISAFPHMECVSITPIENSVIYGLKSFRVKM

>CYP109B1_ortholog(BSU12210)Bacillus subtilis subsp. subtilis 168

MNVLNRRQALQRALLNGKNKQDAYHPFPWYESMRKDAPVSFDEENQVWSVFLYDDVKKVVGDKELFSSCMPQQTSSIGNSIINMDPPKHTKIRSVVNKAFTPRVMKQWEPRIQEITDELIQKFQGRSEFDLVHDFSYPLPVIVISELLGVPSAHMEQFKAWSDLLVSTPKDKSEEAEKAFLEERDKCEEELAAFFAGIIEEKRNKPEQDIISILVEAEETGEKLSGEELIPFCTLLLVAGNETTTNLISNAMYSILETPGVYEELRSHPELMPQAVEEALRFRAPAPVLRRIAKRDTEIGGHLIKEGDMVLAFVASANRDEAKFDRPHMFDIRRHPNPHIAFGHGIHFCLGAPLARLEANIALTSLISAFPHMECVSITPIENSVIYGLKSFRVKM

>CYP109B1_ortholog(QF06_05085)Bacillus sp. YP1

MNVLNRRQALQRALLNGKNKQDAYHPFPWYESMRKDAPVSFDEENQVWSVFLYDDVKKVVGDKELFSSCMPQQTSSIGNSIINMDPPKHTKIRSVVNKAFTPRVMKQWEPRIQEITDELIQKFQGRSEFDLVHDFSYPLPVIVISELLGVPSAHMDQFKAWSDLLVSTPKDKSEEAEKAFLEERDKCEEELAAFFAGIIEEKRNKPAQDIISILVEAEETGEKLSGEELIPFCTLLLVAGNETTTNLISNAMYSILETPGVYEELRSHPELMPQAVEEALRFRAPAPVLRRIAKRDTEIGGHLIKEGDMILAFVASANRDEAKFDRPHMFDIHRHPNPHIAFGHGIHFCLGAPLARLEANIALTSLISAFPHMECVSITPIENSVIYGLKSFRVKM

>CYP109B1_ortholog(A7A1_0230)Bacillus subtilis subsp. subtilis BSP1

MNVLNRRQALQRALLNGKNKQDAYHPFPWYESMRKDAPVSFDEENQVWSVFLYDDVKKVVGDKELFSSYMPQQTSSIGNSIINMDPPKHTKIRSVVNKAFTPRVMKQWEPRIQEITDELIQKCQGRSEFDLVHDFSYPLPVIVISELLGVPSAHMDQFKAWSDLLVSTPKDKSEEAEKAFLEERDKCEEELAAFFAGIIEEKRNKPAQDIISILVKAEETGEKLSGEELIPFCTLLLVAGNETTTNLISNAMYSILETPGVYEELRSHPELMPQAVEEALRFRAPAPVLRRIAKRDTEIGGHLIKEGDMVLAFVASANRDEAKFDRPHMFDIRRHPNPHIAFGHGIHFCLGAPLARLEANIALTSLISAFPHMECVSITPIENSVIYGLKSFRVKM

>CYP109B1_ortholog(Q433_06950)Bacillus subtilis subsp. subtilis OH 131.1

MSVLNRRQALQRALLNGKNKQDAYHPFPWYESMRKDSPVSFDEENQVWSVFLYDDVKKVVGDKELFSSYMPQQTSSIGNSIINMDPPKHTKIRSVVNKAFTPRVMKQWEPRIQEITDELIQKCQGRSEFDLVHDFSYPLPVIVISELLGVPSAHMDQFKAWSDLLVSTPKDKSEEAEKAFLEERDKCEEELAAFFAGIIEEKRNKPAQDIISILVEAEETGEKLSGEELIPFCTLLLVAGNETTTNLISNAMYSILETPGVYEELRSHPELMPQAVEEALRFRAPAPVLRRIAKRDTEIGGHLIKEGDMVLAFVASANRDEAKFDRPHMFDIHRHPNPHIAFGHGIHFCLGAPLARLEANIALTSLISAFPQMECVSITPIENSVIYGLKSFRVKM

>CYP109B1_ortholog(C663_1252)Bacillus subtilis XF-1

MNVLNRRQALQRALLNGKNKQDAYHPFPWYESMRADAPVSFDEENQVWSVFLYDDVKKVVGDKELFSSYMPQQTSSIGNSIINMDPPKHTKIRSVVNKAFTPRVMKQWEPRIQEITDELIQKFQERSEFDLVHDFSYPLPVIVISELLGVPSKHMDQFKAWSDLLVSTPKDKSEEAEKAFLEERDKCEEELAAFFAGIIEEKRNKPAQDIISILVEAEETGKKLSGEELIPFCTLLLVAGNETTTNLISNAMYSILETPGVYEELRSHPELMPQAVEEALRFRAPAPVLRRIAKRDTKIGGHLIKEGDMVLAFVASANRDEAKFDRPHMFDIRRHPNPHIAFGHGIHFCLGAPLARLEANIALTSLISAFPHMECVSITPIENSVIYGLKSFRVKM

>CYP109B1_ortholog(I653_06225)Bacillus subtilis subsp. subtilis BAB-1

MNVLNRRQALQRALLNGKNKQDAYHPFPWYESMRADAPVSFDEENQVWSVFLYDDVKKVVGDKELFSSYMPQQTSSIGNSIINMDPPKHTKIRSVVNKAFTPRVMKQWEPRIQEITDELIQKFQERSEFDLVHDFSYPLPVIVISELLGVPSKHMDQFKAWSDLLVSTPKDKSEEAEKAFLEERDKCEEELAAFFAGIIEEKRNKPAQDIISILVEAEETGKKLSGEELIPFCTLLLVAGNETTTNLISNAMYSILETPGVYEELRSHPELMPQAVEEALRFRAPAPVLRRIAKRDTKIGGHLIKEGDMVLAFVASANRDEAKFDRPHMFDIRRHPNPHIAFGHGIHFCLGAPLARLEANIALTSLISAFPHMECVSITPIENSVIYGLKSFRVKM

>CYP109B1_ortholog(BSn5_18065)Bacillus subtilis BSn5

MNVLNRRQALQRALLNGKNKQDAYHPFPWYESMRKDAPVSFDEENQVWSVFLYDDVKKVVGDKELFSSYMPQQTSSIGNSIINMDPPKHTKIRSVVNKAFTPRVMKQWEPRIQEITDELIQKCQGRSEFDLVHDFSYPLPVIVISELLGVPSAHMDQFKAWSDLLVSTPKDKSEEAEKAFLEERDKCEEELAAFFAGIIEEKRNKPAQDIISILVKAEETGEKLSGEELIPFCTLLLVAGNETTTNLISNAMYSILETPGVYEELRSHPELMPQAVEEALRFRAPAPVLRRIAKRDTEIGGHLIKEGDMVLAFVASANRDEVKFDRPHMFDIRRHPNPHIAFGHGIHFCLGAPLARLEANIALTSLISAFPQMECVSITPIENSVIYGLKSFRVKM

>CYP109B1_ortholog(I33_1373)Bacillus subtilis subsp. subtilis RO-NN-1

MEHSLLNFFKGVTIMSVLNRRQALQRALLNGKNKQDAYHPFPWYESMRKDAPVSFDEENQVWSVFLYDDVKKVVGDKELFSSYMPQQTSSIGNSIINMDPPKHTKIRSVVNKAFTPRVMKQWEPRIQEITDELIQKFQGRSEFDLVHDFSYPLPVIVISELLGVPSKHMDQFKAWSDLLVSTPKDKTEEAEKAFLEERDKCEEELAAFFAGIIEEKRNKPAQDIISILVEAEETGEKLSGEELIPFCTLLLVAGNETTTNLISNAMYSILETPGVYEELRSHPELVPQAVEEALRFRAPAPVLRRIAKRDTKIGGHLMKEGDMVLAFVASANRDEAKFDRPHMFDIRRHPNPHIAFGHGIHFCLGAPLARLEANIALTSLISAFPHMECVSITPIENSVIYGLKSFRVKM

>CYP109B1_ortholog(MY9_1333)Bacillus sp. JS

MNVLNRRQALQRALLNGKNKQDAYHPFPWYESMRKDAPVSFDEENQVWSVFLYDDVKKVIGDKELFSSYMPQQTSSIGNSIINMDPPRHTKIRSVVNKAFTPRVMKQWEPRIQEITDELIQSFQGRSEFDLVHDFSYPLPVIVISELLGVPSEHMDQFKAWSDLLVSTPKDKSEEAEKAYLEERDKCEKELAAFFAGIIEEKRNKPAQDIISILVEAEETGEKLSGEELIPFCTLLLVAGNETTTNLISNAMYSILETPGVYEELRSHPELMSQAVEEALRFRAPAPVLRRIAKRDTEIGGHLMKEGDMVLAFVASANRDEAKFDRPHMFDIRRHPNPHIAFGHGIHFCLGAPLARLEANIALTSLISAFPHMECLSITPIENSVIYGLKSFRVKM

>CYP109B1_ortholog(BSUW23_06210)Bacillus subtilis subsp. spizizenii W23

MSVLNRRQALQRALLNGKNKQDAYHPFPWYESMRKDAPVSFDEENQVWSVFLYDDVKKVIGDKELFSSYMPQQSSAIGNSIINMDPPRHTQIRSVVNKAFTPRVMKQWEPRIQEITDELIQKFQGRSEFDLVHDFSYPLPVIVISELLGVPSEHMDQFKTWSDLLVSTPKDKSEEAEEAFLEERNKCEEELAAFFANIIEEKRNKPAQDIISILVEAEETGEKLSGEELVPFCTLLLVAGNETTTNLISNAMYSILETPDVYNELRSHPELTPQAVEEALRFRAPAPVLRRIAKRDTEIGGHLIKEGDMVLAFVASANRDETKFDRAHLFDIHRHPNPHIAFGHGIHFCLGAPLARLEAKIALTSLISAFPHMECVSITPIENSVIYGLKSFRVNI

>CYP109B1_ortholog(GYO_1528)Bacillus subtilis subsp. spizizenii TU-B-10

MNVLNRRQALQRALLNGKNKQDAYHPFPWYESMRKDAPVSFDEENQVWSVFLYDDVKKVIGDKELFSSYMPQQSSSIGNSIINMDPPRHTQIRSVVNKAFTPRVMKQWEPRIQEITDELIQKFQGRREFDIVHDFSYPLPVIVISELLGVPSEHMDQFKTWSDLLVSTPKDKSEEAEKAFLEERNKCEEELAVFFANIIEEKRNKPAQDIISILVEAEETGEKLSGEELVPFCTLLLVAGNETTTNLISNAMYSILETPDVYDELRSHPELVPQAVEEALRFRAPAPVLRRIAKRDTEIGGHLIKEGDMVLAFVASANRDETKFDRAHLFDIHRHPNPHIAFGHGIHFCLGAPLARLEAKIALTSLISAFPHMESVSITPIENSVIYGLKSFRVKI

>CYP109B2_ortholog(BATR1942_03755)Bacillus atrophaeus 1942

MNVVNQRQALQRAILKGINKQDAYHPFPWYESMRRESPVHYDEENQVWSVFLYEDVKKIIGDKNAFSNYVKQQANSLGNSIINMDPPRHTQIRSVVNKAFTPRVLKQWESRIQGITDDLIDRLLGSQEFDLVQDFSYPLPVVVISELLGVPSEYMDQFKKWSDILVSTPKDGSEEAEKAFQEERNKCEQELAAFFAAIIEEKRKQPAEDLISILIKAEEEGEKLSEDELIPFCNLLLVAGNETTTNLISNAMYSILDMPGAYDELRDDPALIPQAVEEALRFRAPAPILRRIAKQDVEIRGHLIREGDMVLAFVASANRDEAKFEQAHIFDIHRHPNPHIAFGHGNHFCLGAPLARLEAQIALKALTNAFPGMERISISPIANSVIYGLKSFRVKV

>CYP109B2_ortholog(TD68_03315)Bacillus atrophaeus NRS 1221A

MNVVNQRQALQRAILKGINKQDAYHPFPWYESMRRESPVHYDEENQVWSVFLYEDVKKIIGDKNAFSNYVKQQANSLGNSIINMDPPRHTQIRSVVNKAFTPRVLKQWESRIQGITDDLIDRLLGSQEFDLVQDFSYPLPVVVISELLGVPSEYMDQFKKWSDILVSTPKDGSEEAEKAFQEERNKCEQELAAFFAAIIEEKRKQPAEDLISILIKAEEEGEKLSEDELIPFCNLLLVAGNETTTNLISNAMYSILDMPGAYDELRDDPALIPQAVEEALRFRAPAPILRRIAKQDVEIRGHLIREGDMVLAFVASANRDEAKFEQAHIFDIHRHPNPHIAFGHGNHFCLGAPLARLEAQIALKALTNAFPGMERISISPIANSVIYGLKSFRVKV

>CYP109B3(BAMF_1310)Bacillus amyloliquefaciens DSM 7

MSNLRSPRQAIQRTLMKGKDGLDVYNPFPWYEKMRRESPIQFDEETKVWSVFLYDDAKKVISDKETFSSLMSDMKSSIAKSMLNMDPPKHTQIRSAVNRAFTPRVLKEWEPRIEEITDNLLKQAKNKGRIDIVKDLSYPLPVIVISELLGVPSEHMDQFKKWSDILVSMPKDASPEEAEKNQKERDQCETELAAFFAEIIDSKRKQPGQDIISILIKEEEEGEKLSAEDLIPFCNLLLVAGNETTTNLISNAVYSILETPGLYEELRHDPSLIAQTVEETLRFRAPAPFVRRTVRHDTELCGRRLKSGDIVLCYIASANRDENKFEQADVFDIHRQSNPHLSFGFGVHFCLGAPLARLEAEVALRGIVKAFSHLEPVRVEPIQNSVMYGLDSLEAEINENRGEEKR

>CYP109B3_ortholog(BAMTA208_11040)Bacillus amyloliquefaciens TA208

MSNLRSPRQAIQRTLMKGKDGLDVYNPFPWYEKMRRESPIQFDEETKVWSVFLYDDAKKVISDKETFSSLMSDMKSSIAKSMLNMDPPKHTQIRSAVNRAFTPRVLKEWEPRIEEITDNLLKQAKNKGRIDIVKDLSYPLPVIVISELLGVPSEHMDQFKKWSDILVSMPKDASPEEAEKNQKERDQCETELAAFFAEIIDSKRKQPGQDIISILIKEEEEGEKLSAEDLIPFCNLLLVAGNETTTNLISNAVYSILETPGLYEELRHDPSLIAQTVEETLRFRAPAPFVRRTVRHDTELCGRRLKSGDIVLCYIASANRDENKFEQADVFDIHRQSNPHLSFGFGVHFCLGAPLARLEAEVALRGIVKAFSHLEPVRVEPIQNSVMYGLDSLEAEINENRGEEKR

>CYP109B3_ortholog(BAXH7_02260)Bacillus amyloliquefaciens XH7

MSNLRSPRQAIQRTLMKGKDGLDVYNPFPWYEKMRRESPIQFDEETKVWSVFLYDDAKKVISDKETFSSLMSDMKSSIAKSMLNMDPPKHTQIRSAVNRAFTPRVLKEWEPRIEEITDNLLKQAKNKGRIDIVKDLSYPLPVIVISELLGVPSEHMDQFKKWSDILVSMPKDASPEEAEKNQKERDQCETELAAFFAEIIDSKRKQPGQDIISILIKEEEEGEKLSAEDLIPFCNLLLVAGNETTTNLISNAVYSILETPGLYEELRHDPSLIAQTVEETLRFRAPAPFVRRTVRHDTELCGRRLKSGDIVLCYIASANRDENKFEQADVFDIHRQSNPHLSFGFGVHFCLGAPLARLEAEVALRGIVKAFSHLEPVRVEPIQNSVMYGLDSLEAEINENRGEEKR

>CYP109B3_ortholog(LL3_01316)Bacillus amyloliquefaciens LL3

MKGKDGLDVYNPFPWYEKMRRESPIQFDEETKVWSVFLYDDAKKVISDKETFSSLMSDMKSSIAKSMLNMDPPKHTQIRSAVNRAFTPRVLKEWEPRIEEITDNLLKQAKNKGRIDIVKDLSYPLPVIVISELLGVPSEHMDQFKKWSDILVSMPKDASPEEAEKNQKERDQCETELAAFFAEIIDSKRKQPGQDIISILIKEEEEGEKLSAEDLIPFCNLLLVAGNETTTNLISNAVYSILETPGLYEELRHDPSLIAQTVEETLRFRAPAPFVRRTVRHDTELCGRRLKSGDIVLCYIASANRDENKFEQADVFDIHRQSNPHLSFGFGVHFCLGAPLARLEAEVALRGIVKAFSHLEPVRVEPIQNSVMYGLDSLEAEINENRGEEKR

>CYP109B4(BGLY_0730)Bacillus glycinifermentans

MSQTKQQNPIQKALINGKNRQDPYDPFPWYEKMRTESPIHYDEDSKVWSVFRYNDVKRVISDKDFFSNQFPQIGTGNTFAKTMISMDPPKHTRIRSIVNRAFTPRVMKEWEPRIRELTNELLADVRGREEIDLVQNFSYPLPVIVISELLGVPSVYKHHFKEWSDLLVSLPKSDRPEDVNEWKNIRDQGEEELTAFFEKMIEEKRQNLGNDLISLLIKAEQEGDKLSPDELVPFCNLLLMAGNETTTNLVSNAVYSILETPGAYDELAGHPELIPQAVEEAVRFRAPAPMIVRFVKQDTEIRGVSLKKGEGVVAFLASANRDEAKFERAHEFDIHRHPNPHIGFGHGIHFCLGAPLARLEAAIALEALLKQYSSMEKLAIVPMADSSMYGLKHFRLRVKSAGKTAPYSF

>CYP109B4_ortholog(BLi00706)Bacillus licheniformis DSM 13 = ATCC 14580

MSQTKQQNPIQKALINGKNRQDPYDPFPWYKKMRTESPIHYDEDSKVWSVFRYDDVKRVISDKDFFSNQFPQLGTGNTFAKTMISMDPPKHTRIRSIVNKAFTPRVMKEWEPRIRELTNQLLADVRGREEIDLVQDFSYPLPVIVISELLGVPLVYKHHFKEWSDLLVSLPKSDRPEDVNEWKNIRDQGEEELTAFFEKMIEEKRQNLGNDLISLLIKAEQEGDKLSPDELVPFCNLLLMAGNETTTNLVSNAVYSILETPGVYDELARHPELIPQAVEEAVRFRAPAPMIVRFVKQDTEIRGVSLKKGEGVIAFLASANRDETKFERAHEFDIHRHPNPHIGFGHGIHFCLGAPLARLEAAIAIEALLKQYASMEKLAVVPMADSSMYGLKHFRLRVKSAGETAPN

>CYP109B4_ortholog(BL01488)Bacillus licheniformis ATCC 14580

MSQTKQQNPIQKALINGKNRQDPYDPFPWYKKMRTESPIHYDEDSKVWSVFRYDDVKRVISDKDFFSNQFPQLGTGNTFAKTMISMDPPKHTRIRSIVNKAFTPRVMKEWEPRIRELTNQLLADVRGREEIDLVQDFSYPLPVIVISELLGVPLVYKHHFKEWSDLLVSLPKSDRPEDVNEWKNIRDQGEEELTAFFEKMIEEKRQNLGNDLISLLIKAEQEGDKLSPDELVPFCNLLLMAGNETTTNLVSNAVYSILETPGVYDELARHPELIPQAVEEAVRFRAPAPMIVRFVKQDTEIRGVSLKKGEGVIAFLASANRDETKFERAHEFDIHRHPNPHIGFGHGIHFCLGAPLARLEAAIAIEALLKQYASMEKLAVVPMADSSMYGLKHFRLRVKSAGETAPN

>CYP109B5(BW16_19640)Bacillus pumilus MTCC B6033

MMETTTPSAVQKALLRGKNKQDPYHPFDWYAKMRQESPVHFDEHSQTWSVFTYEEAKRVTIDKDTFSSQPPQDHRKHSLMKTMVMMDPPKHTRIRSIVSKAFTPRVMKLWEPRIQELMDDLIAQIEGKEEIDLVQDISYPLPVIVIAELLGVPTEHKQSFKEWSDILVSMPKSEHEKDVVEWQKTRDQSEADMMAFFADIIEEKRQNLSDDLISLLIQAEEDGDKLSADELIPFCNLLLLAGNETTTNLISNMIFSLLENPGSYEALAQSPDLIPRAVEEAVRFRAPAPTIVRYVTEDTELGGKVLKKGDSVIVFLASANRDERQFPNAHEYDIHRHPNPHIGFGHGIHFCLGAPLARLEACTAIKAIQSRYESLELLSYVPMTSSGMYGLKALKLRVTPRS

>CYP109B6(UP12_18945)Bacillus pumilus SH-B9

METTTPSAVQKTLLRGKNKQDPYHPFDWYANMRQTSPVHFDEASRTWSVFTYEEAKRVTIDKDTFSSQPPKNQRKHSLMKTMVMMDPPNHTRIRSIVSKAFTPRVMKLWEPRIQELMDELMAQIEGKKEIDLVQDISYPLPVIVIAELLGVPSEHKQSFKEWSDILVSMPKSENEEDVAEWQKTRDKGEADMMAFFADTIEKKRHNLGDDLISLIIQAEENGEKLSADELIPFCNLLLLAGNETTTNLISNMIFSLLEQPGAYEALSQSPELIPRAVEEAVRFRAPAPAIVRYVTKDTELGGKVLKKGDNVIVFLASANRDERQFSNAHEYDIHRHPNPHIGFGHGIHFCLGAPLARLEACTAIKFLIERYEALELLSYVPMTSSSMYGLKELKLRVTPRS

>CYP109B6_ortholog(BPUM_3695)Bacillus pumilus SAFR-032

METTSPSAVQKTLLRGKNKQDPYHPFDWYANMRKTSPVHFDEASRTWSVFTYEEARRVTIDKDTFSSQPPKNQRKHSLMKTMVMMDPPNHTRIRSIVSKAFTPRVMKLWEPRIHELMDELMAQLEGKKEIDLVQDISYPLPVIVIAELLGVPSEHKQSFKEWSDILVSMPKSESEKDVAEWQKTRDKGEADMMAFFADTIEKKRHNLGDDLISLIIQAEENGDKLSADELIPFCNLLLLAGNETTTNLISNMMFSLLEQPGAYEALAQSPELIPRAVEEAVRFRAPAPAIVRYVTKDTELGEKVLKRGDNVIVFLASANRDERQFSNAHEYDIHRHPNPHIGFGHGIHFCLGAPLARLEACTAIKIFIERYEALELLSYVPMTSSSMYGLKELKLRVTPRS

>CYP109B7(BASU_1163)Bacillus velezensis UCMB5113

MSKLRSTRQAIQRTLMKGKNGLDVYNPFPWYEKMRRESPIHFDEETKVWSVFLYDDVKKVISDKETFSSLMTDVKSSIAKSMLNMDPPKHTQIRSAVNRAFTPRVLKEWEPRIKDITDHLLKQAKNKGRIDIVKDLSYPLPVMVISELLGVPSERMDQFKKWSDILVSMPKDASPEAAEKNQQERDQCEAELAAFFAEIIESKRKQPGQDIISILIKEEEEGEKLTAEDLIPFCNLLLVAGNETTTNLISNAVYSILETPGLYEELRQDPSLIAQTVEETLRFRAPAPFVRRTVRHDTELRGRRLKSGEIVLCYVASANRDENKFEKAGVFDIHRQSNPHLSFGFGVHFCLGAPLARLEAEVALKGIVKAFSHLEPVRLSRSETVSCTGWSL

>CYP109B7_ortholog(AJ82_06890)Bacillus velezensis TrigoCor1448

MSKLRSTRQAIQRTLMKGKNGLDVYNPFPWYEKMRRESPIHFDEETKVWSVFLYDDVKKVISDKETFSSLMTDVKSSIAKSMLNMDPPKHTQIRSAVNRAFTPRVLKEWEPRIKDITDHLLKQAKNKGRIDIVKDLSYPLPVMVISELLGVPSERMDQFKKWSDILVSMPKDASPEAAEKNQQERDQCEAELAAFFAEIIESKRKQPGQDIISILIKEEEEGEKLTAEDLIPFCNLLLVAGNETTTNLISNAVYSILETPGLYEELRQDPSLIAQTVEETLRFRAPAPFVRRTVRHDTELRGRRLKSGEIVLCYVASANRDENKFEKAGVFDIHRQSNPHLSFGFGVHFCLGAPLARLEAEVALKGIVKAFSHLEPVRIEPIRNSVMYGLESLEAEINENEGEEK

>CYP109B7_ortholog(SB24_03665)Bacillus sp. Pc3

MSKLRSTRQAIQRTLMKGKNGLDVYNPFPWYEKMRRESPIHFDEETKVWSVFLYDDVKKVISDKETFSSLMTDVKSSIAKSMLNMDPPKHTQIRSAVNRAFTPRVLKEWEPRIKDITDHLLKQAKNKGRIDIVKDLSYPLPVMVISELLGVPSERMDQFKKWSDILVSMPKDASPEAAEKNQQERDQCEAELAAFFAEIIESKRKQPGQDIISILIKEEEEGEKLTAEDLIPFCNLLLVAGNETTTNLISNAVYSILETPGLYEELRQDPSLIAQTVEETLRFRAPAPFVRRTVRHDTELRGRRLKSGEIVLCYVASANRDENKFEKAGVFDIHRQSNPHLSFGFGVHFCLGAPLARLEAEVALKGIVKAFSHLEPIRIEPIRNSVMYGLESLEAEINENEGEEK

>CYP109B7_ortholog(RBAU_1184)Bacillus velezensis UCMB5033

MSKLRSTRQAIQRTLMKGKNGLDVYNPFPWYEKMRRESPIHFDEETKVWSVFLYDDVKKVISDKETFSSLMTDVKSSIAKSMLNMDPPKHTQIRSAVNRAFTPRVLKEWEPRIKDITDHLLKQAKNKGRIDIVKDLSYPLPVMVISELLGVPSERMDQFKKWSDILVSMPKDASPEAAEKNQQERDQCEAELAAFFAEIIESKRKQPGQDIISILIKEEEEGEKLTAEDLIPFCNLLLVAGNETTTNLISNAVYSILETPGLYEELRQDPSLIAQTVEETLRFRAPAPFVRRTVRHDTELRGRRLKSGEIVLCYVASANRDENKFEKAGVFDIHRQSNPHLSFGFGVHFCLGAPLARLEAEVALKGIVKAFSHLEPVQIEPIRNSVMYGLESLEAEINENEGEEK

>CYP109B7_ortholog(BAM5036_1135)Bacillus velezensis UCMB5036

MSKLRSTRQAIQRTLMKGKNGLDVYNPFPWYEKMRRESPIHFDEETKVWSVFLYDDVKKVISDKETFSSLMTDVKSSIAKSMLNMDPPKHTQIRSAVNRAFTPRVLKEWEPRIKDITDHLLKQAKNKGRIDIVKDLSYPLPVMVISELLGVPSERMDQFKKWSDILVSMPKDASPEAAEKNQQERDQCEAELAAFFAEIIESKRKQPGQDIISILIKEEEEGEKLTAEDLIPFCNLLLVAGNETTTNLISNAVYSILETPGLYDELRQDPSLIAQTVEETLRFRAPAPFVRRTVRHDTELRGRRLKSGEIVLCYVASANRDENKFEKAGVFDIHRQSNPHLSFGFGVHFCLGAPLARLEAEVALKGIVKAFSHLEPVRIEPIRNSVMYGLESLEAEINENEGEEK

>CYP109B7_ortholog(V529_11720)Bacillus velezensis SQR9

MSKLRSTRQAIQRTLMKGKNGLDVYNPFPWYEKMRRESPIHFDEETKVWSVFLYDDVKKVISDKETFSSLMTDVKSSIAKSMLNMDPPKHTQIRSAVNRAFTPRVLKEWEPRIKDITDHLLKQAKNKGRIDIVKDLSYPLPVMVISELLGVPSERMDQFKKWSDILVSMPKDASPEAAEKNQQERDRCEAELAAFFAEIIKSKRKQPGQDIISILIKEEEEGEKLTAEDLIPFCNLLLVAGNETTTNLISNAVYSILETPGLYEELRQDPSLIAQTVEETLRFRAPAPFVRRTVRHDTELRGRRLKSGEIVLCYVASANRDENKFEKAGVFDIHRQSNPHLSFGFGVHFCLGAPLARLEAEAALKGIVKAFSHLEPVRIEPIRNSVMYGLESLEAEINENEGEEKR

>CYP109B7_ortholog(NG74_01251)Bacillus velezensis

MSKLRSTRQAIQRTLMKGKNGLDVYNPFPWYEKMRRESPIHFDEETKVWSVFLYDDVKKVISDKETFSSLMTDVKSSIAKSMLNMDPPKHTQIRSAVNRAFTPRVLKEWEPRIKDITDHLLKQAKNKGRIDIVKDLSYPLPVMVISELLGVPSERMDQFKKWSDILVSMPKDASPEAAEKNQQERDQCEAELAAFFAEIIESKRKKPGQDIISILIKEEEEGEKLTAEDLIPFCNLLLVAGNETTTNLISNAVYSILETPGLYEELRQDPSLIAQTVEETLRFRAPAPFVRRTVRHDTELRGRRLKSGEIVLCYVASANRDENKFEKAGVFDIHRQSNPHLSFGFGVHFCLGAPLARLEAEAALKGIVKTFSHLEPVGIEPIRNSVMYGLESLEAEINESEGEEKR

>CYP109B7_ortholog(BAPNAU_2562)Bacillus velezensis NAU-B3

MSKLRSTRQAIQRTLMKGKNGLDVYNPFPWYEKMRRESPIHFDEETKVWSVFLYDDVKKVISDKETFSSLMTDVKSSIAKSMLNMDPPKHTQIRSAVNRAFTPRVLKEWEPRIKDITDHLLKQAKNKGRIDIVKDLSYPLPVMVISELLGVPSERMDQFKKWSDILVSMPKDASPEAAEKNQQERDQCEAELAAFFAEIIESKRKKPGQDIISILIKEEEEGEKLTAEDLIPFCNLLLVAGNETTTNLISNAVYSILETPGLYEELRQDPSLIAQTVEETLRFRAPAPFVRRTVRHDTELRGRRLKSGEIVLCYVASANRDENKFEKAGVFDIHRQSNPHLSFGFGVHFCLGAPLARLEAEAALKGIVKTFSHLEPVGIEPIRNSVMYGLESLEAEINESEGEEKR

>CYP109B7_ortholog(OY17_09245)Bacillus sp. BH072

MSKLRSTRQAIQRTLMKGKNGLDVYNPFPWYEKMRRESPIHFDEETKVWSVFLYDDVKKVISDKETFSSLMTDVKSSIAKSMLNMDPPKHTQIRSAVNRAFTPRVLKEWEPRIKDITDHLLKQAKNKGRIDIVKDLSYPLPVMVISELLGVPSEHMDQFKKWSDILVSMPKDASPEAAEKNQQERDQCEAELAAFFAEIIESKRKKPGQDIISILIKEEEEGEKLTAEDLIPFCNLLLVAGNETTTNLISNAVYSILETPGLYEELRQDPSLIAQTVEETLRFRAPAPFVRRTVRHDTELRGRRLKSGEIVLCYVASANRDENKFEKAGVFDIHRQSNPHLSFGFGVHFCLGAPLARLEAEAALKGIVKTFSHLEPVGIEPIRNSVMYGLESLEAEINESEGEEKR

>CYP109B7_ortholog(BANAU_1161)Bacillus velezensis YAU B9601-Y2

MSKLRSTRQAIQRTLMKGKNGLDVYNPFPWYEKMRRESPIHFDEETKVWSVFLYDDVKKVISDKETFSSLMTDVKSSIAKSMLNMDPPKHTQIRSAVNRAFTPRVLKEWEPRIKDITDHLLKQAKNKGRIDIVKDLSYPLPVMVISELLGVPSEHMDQFKKWSDILVSMPKDASPEAAEKNQQERDQCEAELAAFFAEIIESKRKKPGQDIISILIKEEEEGEKLTAEDLIPFCNLLLVAGNETTTNLISNAVYSILETPGLYEELRQDPSLIAQTVEETLRFRAPAPFVRRTVRHDTELRGRRLKSGEIVLCYVASANRDENKFEKAGVFDIHRQSNPHLSFGFGVHFCLGAPLARLEAEAALKGIVKTFSHLEPVGIEPIRNSVMYGLESLEAEINESEGEEKR

>CYP109B7_ortholog(U722_06375)Bacillus amyloliquefaciens LFB112

MSKLRSTRQAIQRTLMKGKNGLDVYNPFPWYEKMRRESPIYFDEETKVWSVFLYDDVKKVISDKETFSSRMTDVKSSIAKSMLNMDPPKHTQIRSAVNRAFTPRVLKEWEPRIKDITDHLLKQAKNKGRIDIVKDLSYPLPVMVISELLGVPSERMDQFKKWSDILVSMPKDASPEAAEKNQQERDQCEAELAAFFAGIIESKRKQPGQDIISILIKEEEEGEKLTPEDLIPFCNLLLVAGNETTTNLISNAVYSILETPGLYEELRQDPSLIAQTVEETLRFRAPAPFVRRTVRHDTELRGRRLKSGEIVLCYVASANRDENKFEKAGVFDIHRQSNPHLSFGFGVHFCLGAPLARLEAEAALKGIVKAFSHLEPVRIEPIRNSVMYGLESLEAEINENEGEEKR

>CYP109B7_ortholog(BACAU_1180)Bacillus velezensis CAU B946

MSKLRSTRQAIQRTLMKGKNGLDVYNPFPWYEKMRRESPIYFDEETKVWSVFLYDDVKKVISDKETFSSRMTDVKSSIAKSMLNMDPPKHTQIRSAVNRAFTPRVLKEWEPRIKDITDHLLKQAKNKGRIDIVKDLSYPLPVMVISELLGVPSERMDQFKKWSDILVSMPKDASPEAAEKNQQERDQCEAELAAFFAGIIESKRKQPGQDIISILIKEEEEGEKLTPEDLIPFCNLLLVAGNETTTNLISNAVYSILETPGLYEELRQDPSLIAQTVEETLRFRAPAPFVRRTVRHDTELRGRRLKSGEIVLCYVASANRDENKFEKAGVFDIHRQSNSHLSFGFGVHFCLGAPLARLEAEAALKGIVKAFSHLEPVRIEPIRNSVMYGLESLEAEINENEGEEKR

>CYP109B7_ortholog(KSO_013430)Bacillus amyloliquefaciens IT-45

MSKLRSTRQAIQRTLMKGKNGLDVYNPFPWYEKMRRESPIYFDEETKVWSVFLYDDVKKVISDKETFSSRMTDVKSSIAKSMLNMDPPKHTQIRSAVNRAFTPRVLKEWEPRIKDITDHLLKQAKNKGRIDIVKDLSYPLPVMVISELLGVPSERMDQFKKWSDILVSMPKDASPEAAEKNQQERDQCEAELAAFFAGIIESKRKQPGQDIISILIKEEEEGEKLTPEDLIPFCNLLLVAGNETTTNLISNAVYSILETPGLYEELRQDPSLIAQTVEETLRFRAPAPFVRRTVRHDTELRGRRLKSGEIVLCYVASANRDENKFEKAGVFDIHRQSNSHLSFGFGVHFCLGAPLARLEAEAALKGIVKAFSHLEPVRIEPIRNSVMYGLESLEAEINENEGEEKR

>CYP109B7_ortholog(MUS_1309)Bacillus amyloliquefaciens Y2

MKGKNGLDVYNPFPWYEKMRRESPIHFDEETKVWSVFLYDDVKKVISDKETFSSLMTDVKSSIAKSMLNMDPPKHTQIRSAVNRAFTPRVLKEWEPRIKDITDHLLKQAKNKGRIDIVKDLSYPLPVMVISELLGVPSEHMDQFKKWSDILVSMPKDASPEAAEKNQQERDQCEAELAAFFAEIIESKRKKPGQDIISILIKEEEEGEKLTAEDLIPFCNLLLVAGNETTTNLISNAVYSILETPGLYEELRQDPSLIAQTVEETLRFRAPAPFVRRTVRHDTELRGRRLKSGEIVLCYVASANRDENKFEKAGVFDIHRQSNPHLSFGFGVHFCLGAPLARLEAEAALKGIVKTFSHLEPVGIEPIRNSVMYGLESLEAEINESEGEEKR

>CYP109E1_ortholog(BMD_3874)Bacillus megaterium DSM 319

MKTERENGIVRQVNTIQTKEERFNPFSWYEEMRNTAPVQWDEERQVWDVFHYDGVKEVLEQKNIFSSDRRPPQNQRQTALGTSLINIDPPKHAEMRALVNKAFTPKAMKAWEPKIARITNELLQEVEHLEDIDIVEHLSYPLPVMVIADILGVPIEDQRQFKDWSDIIVAGPSNNERETLEKLQQEKMKANDELETYFYRIIEEKRTRPGDDIISVLLQAKEEGKQLTDEEIVGFSILLLIAGNETTTNLISNTIYCLMEDKASFERLKREKELLPSGIEEVLRYRSPVQALHRIVKEDVTLAGKKLKAGEHVVPWMGSAHRDAEYFEDPEVFKIDRKPNVHMAFGRGIHFCLGAPLARIEAKIMLAELIDRYPQMDWSPSFELKPIESTFVYGLKELLIRKNV

>CYP109E1_ortholog(BG04_798)Bacillus megaterium NBRC 15308 = ATCC 14581

MKTERENGIVRQVNTIQTKEERFNPFSWYEEMRNTEPVQWDEKRQVWDVFHYDGVKEVLEQKNIFSSDRRPPQNQRQTALGTSLINIDPPKHAEMRALVNKAFTPKAMKAWEPKIARITNELLQEVEHLEDIDIVEHLSYPLPVMVIADILGVPIEDQRQFKDWSDIIVAGPSNNERETLEKLQQEKMKANDELETYFYRIIEEKRTRPGDDIISLLLQAKEEGKQLTDEEIVGFSILLLIAGNETTTNLISNTIYCLMEDKASFERLKREKELLPSAIEEVLRYRSPVQALHRIVKEDVTLAGKKLKAGEHVVPWMGSAHRDAEYFEDPDVFKIDRKPNVHMAFGRGIHFCLGAPLARIEAKIMLAELIDRYPQMDWSPSFELKPIESTFVYGLKELLIRKNV

>CYP109E1_ortholog(BMQ_3882)Bacillus megaterium QM B1551

MKTERENGIVRQVNTIQTKEERFNPFSWYEEMRNSAPVQWDEERQVWDVFHYDGVKEVLEQKNIFSSDRRPPQNQRQTALGTSLINIDPPKHAEMRSLVNKAFTPKAMKAWEPKIARITNELLQEVEHLEDIDIVEHLSYPLPVMVIADILGVPIEDQRQFKDWSDIIVAGPSNNERETLEKLQQEKMKANDELETYFYRIIEEKRTRPGDDIISVLLQAKEEGKQLTDEEIVGFSILLLIAGNETTTNLISNTIYCLMEDKASFERLKREKELLPSAIEEVLRYRSPVQALHRIVKEDVILAGQKLKAGEHVVPWMGSAHRDAEYFEDPDVFKIDRKPNVHMAFGRGIHFCLGAPLARIEAKIMLAELIDRYPQMDWSPAFELKPIESTFVYGLKELLIRKNV

>CYP109E1_ortholog(BMWSH_1341)Bacillus megaterium WSH-002

MKTERENGIVRQVNTIQSKEERFNPFSWYEEMRNSAPVQWDEERQVWDVFHYDGVKEVLEQKNIFSSDRRPPQNQRQTALGTSLINIDPPKHAEMRALVNKAFTPKAMKAWEPKIARITHELLQEVEHLEDIDIVEHLSYPLPVMVIADILGVPIEDQRQFKDWSDIIVAGPSNNERETLEKLQQDKMKANDELETYFYKIIEEKRTHPGADIISVLLQAKEEGKQLTDEEIVGFSILLLIAGNETTTNLISNTIYCLMEDKASFERLKREKELLPSAIEEVLRYRSPVQALHRIVKEDVVLAGKKLKAGEHVVPWMGSAHRDAQYFEDPDVFQIDRKPNIHMAFGRGIHFCLGAPLARIEAKVMLAELIDRYPHMDWSPAFELKPIESTFVYGLKELLIRKHV

>CYP109J5(PM3016_4925)Paenibacillus mucilaginosus 3016

MIPFLTPEILRNPYPIYEMMRASQPVMYIEPMRFWSVFRFDHVRTVLSDSARFSSAGGRPPASQGTPAPGQRDGFSLITTDPPRHTQLRSLVNQAFTPKAVAALEPRIAELAHELLDRVSGTGKIDLIQDFAYPLPVIVIAELLGIPSGDRDRFKHWSDEVVASADTLIGGSASGSLQAHREMNEYFSGIIAERRKAPKDDLISALIAAEEGDFHLSEGDILSFCALLLVAGNETTTNLIGNAVLTLLEHPEELAKLRSRPELLPSAIEEVLRFRSPVQAMFRTANEDVELGGQVIPAGSRVVAFIGSANRDEEKFPDAARFDIERTPNGHIAFGHGIHFCLGAPLARLEARIALAAVLDRLPELARVNDEPLTPARGFIVHGVSSMPLRFRPQEQGARR

>CYP109J5_ortholog(KNP414_05566)Paenibacillus mucilaginosus KNP414

MIPFLTPEILRNPYPIYEMMRASQPVMYIEPMRFWSVFRFDHVRTVLSDSARFSSAGGRPPASQGTPAPGQRDGFSLITTDPPRHTQLRSLVNQAFTPKAVAALEPRIAELAHELLDRVSGTGKIDLIQDFAYPLPVIVIAELLGIPSGDRDRFKHWSDEVVASADTLIGGSASGSLQAHREMNEYFSGIIAERRKAPKDDLISALIAAEEGDFHLSEGDILSFCALLLVAGNETTTNLIGNAVLTLLEHPEELAKLRSRPELLPSAIEEVLRFRSPVQAMFRTANEDVELGGQVIPAGSRVVAFIGSANRDEEKFSDAARFDIERTPNGHIAFGHGIHFCLGAPLARLEARIALAAVLDRLPELARVNDEPLTPARGFIVHGVSSMPLRFRPQEQGSRR

>CYP109J5_ortholog(B2K_25525)Paenibacillus mucilaginosus K02

MIPFLTPEILRNPYPIYEMMRASQPVMYIEPMRFWSVFRFDHVRTVLSDSARFSSAGGRPPASQGTPAPGQRDGFSLITTDPPRHTQLRSLVNQAFTPKAVAALEPRIAELAHELLDRVSGTGKIDLIQDFAYPLPVIVIAELLGIPSGDRDRFKHWSDEVVASADTLIGGSASGSLQAHREMNEYFSGIIAERRKAPKDDLISALIAAEEGDFHLSEGDILSFCALLLVAGNETTTNLIGNAVLTLLEHPEELAKLRSRPELLPSAIEEVLRFRSPVQAMFRTANEDVELGGQVIPASSRVVAFIGSANRDEEKFPDAARFDIERTPNGHIAFGHGIHFCLGAPLARLEARIALAAVLDRLPELARVNDEPLTPARGFIVHGVSSMPLRFRPQEQGSRR

>CYP109T1(BRLA_c006760)Brevibacillus laterosporus

MNDMKQSFIMIPSNYFRDEEVQLDPYHPFLWYEKMRHQSPVFYNEKADMWNVFLYHDVKRVLEDKQNFSSVLPPKRASPFNRSVIGMDQPMHTDIRSIVMHSFTPKMMKAWAPRIEEITKQLLENVQEQQEFDLVRDFSYPLPVIVIAEMLGVPSSEMSKFKEWSDIVVSSPDNDDPDHLTQFLSVRTKADQELTSFFTEIVEHKRKSPHPESDIISILIQAESEKSKISIDELVAFCKLLLVAGNETTTNFISNTMYSLLESPGAYRQIQQDLSLIPQALEESLRYRSPAQRVVRRVGADVQIGPHLLKKDQIIIAWIGSANRDETVFEQASTFDISRRVNPHLAFGQGIHFCLGAPLARLEAKISLTELFKNIKHISFHENNQPVPIANSTTIYGLKSFPVSVD

>CYP109T2(BCE33L0737)Bacillus cereus E33L

MLYMKTPYQSVVMIPTNKLMGKKALEDPYKPFAWYKEMREKEPICFNHQADMWNVFLYEDVKTVLEDKEYFSNIMPEKKKSPFSQSILGMDPPKHTQIRSIVNRSFTPKALREWEPRIQQITNDILNQLSNRKTFDIVRELFYPLPVIVIAEMLGVSAKDMERFKRWSDIIVSSPSHDDPDYLVEFFNIRLQAENELGEFFEEIIQFNRGKSKQDLNNIISLLVQSEADENISGKEIVPFCKLLLVAGNETTTNLLGNALYCFIEHPNVYDQLQQDISLIPKAIEEVLRYRSPVQRIVRRVKKEIQLKGQTLQTDQIISAWVGSANRDSQQFKDADSFNIYRRRNPHLTFGHGIHFCLGAPLARLEAKIVLTELIKRYKSFSFIDQNLPVPISNSSSIYGLHSFPVKSELN

>CYP109T2_ortholog(BCN_0828)Bacillus cereus NC7401

MKTPYQSVVMIPTNKLMGKKALEDPYKPFAWYKEMREKEPICFNHQADMWNVFLYEDVKTVLEDKEYFSSIMPEKKKSPFPQSILGMDPPKHTQIRSIVNRSFTPKALREWEPRIQQITNDILNQLSNRETFDIVRELFYPLPVIVIAEMLGVSAKDMERFKRWSDIIVSSPSHDDSDYLEEFFNTRLQAENELGEFFEEIIQSNRGKSKKDSNNIISLLVQSEADKNISGKEIVPFCKLLLVAGNETTTNLLGNALYCFIEHPNVYDQLQQDVSLVPKAIEEVLRYRSPVQRIVRRVKKEIQLKGQTLQVDQIISAWVGSANRDSHQFKDVDSFNIYRRRNPHLTFGHGIHFCLGAPLARLEAKIVLTELIKRYKSFSFIDQDLPVPISNSSSIYGLHSFPVKSELN

>CYP109T2_ortholog(BCAH187_A1008)Bacillus cereus AH820

MKTPYQSVVMIPTNKLMGKKALEDPYKPFAWYKEMREKEPICFNHQADMWNVFLYEDVKTVLEDKEYFSSIMPEKKKSPFPQSILGMDPPKHTQIRSIVNRSFTPKALREWEPRIQQITNDILNQLSNRETFDIVRELFYPLPVIVIAEMLGVSAKDMERFKRWSDIIVSSPSHDDSDYLEEFFNTRLQAENELGEFFEEIIQSNRGKSKKDSNNIISLLVQSEADKNISGKEIVPFCKLLLVAGNETTTNLLGNALYCFIEHPNVYDQLQQDVSLVPKAIEEVLRYRSPVQRIVRRVKKEIQLKGQTLQVDQIISAWVGSANRDSHQFKDVDSFNIYRRRNPHLTFGHGIHFCLGAPLARLEAKIVLTELIKRYKSFSFIDQDLPVPISNSSSIYGLHSFPVKSELN

>CYP109T2_ortholog(BACI_c08840)Bacillus cereus biovar anthracis CI

MKTPYQSVVMIPTNKLMGKKALEDPYKPFAWYKEMREKEPICFNHQADMWNVFLYEDVKTVLEDKEYFSSIMPEKKKSPFPQSILGMDPPRHTQIRSIVNRSFTPKALREWEPRIQQITNDILNQLSNRETFDIVRELFYPLPVIVIAEMLGVSAKDMERFKRWSDIIVSSPSHDDSDYLEEFFNTRLQAENELGEFFEEIIQSNRGKSKKDSNNIISLLVQSEADKNISRKEIVPFCKLLLVAGNETTTNLLGNALYCFIEHPNVYDQLQQDISLVPKAIEEVLRYRSPVQRIVRRVKKEIQLKGQTLQVDQIISAWVGSANRDSHQFKDADSFNIYRKRNPHLTFGHGIHFCLGAPLARLEAKIVLTELIKRYKSFSFIDQDLPVPISNSSSIYGLHSFPVKSELN

>CYP109T3(Bcer98_3663)Bacillus cytotoxicus

MKTPYQSIDMIPTNKLIGKQALEEPYKPFVWYKEMREKEPICFNHQADMWNVFLYEDVKTVLEDKEYFSNIMPEKKKSPFPQSILGMDPPKHTQMRSIVNRSFTPKALREWEPRIQQITNDILNQLSNHKTFDIVRELFYPLPVIVIAEMLGVSAKDMERFKKWSDIIVSSPSHEDPDYLKEFFHTRLQAENELGNFFEEIIQLNRGKAKKDSNDIISLLVQSEADINISSKELVAFCKLLLVAGNETTTNLLGNALYCLIEHPNVYEQLQQDLSLIPKTIEEVLRYRSPVQRVVRRVKKEIQLKGQTLQVDQIVSAWVGSANRDSYQFKDADSFNIYRRRNPHLTFGHGIHFCLGAPLARLEAKIVLTELIKRYKSFSFIDQNLPTPISNSSTIYGLSSFPVKSELLHIK

>CYP109T4(BcrFT9_02496)Bacillus cereus FT9

MKTPYQSIDMIPTSKLIGKKALEDPYKPFAWYKEMREKEPICFNHQADMWNVFLYEDVKTVLEDKEHFSNIMPEKKKSPFPQSILGMDPPKHTQIRSIVNRSFTPKSLREWEPRIQQITNDILNQLSNRKTFDIVRELFYPLPVIVIAEMLGVSAKDMERFKKWSDIIVSSPSHDDPDYLAEFFHTRLQAENELGDFFEEIIQLNREKSQKDANDIISLLVQSEAEKSISGKELVSFCKLLLVAGNETTTNLLGNALYCFIEYPNVYEQLQQDISLIPKAIEEVLRYRSPVQRITRRVKKEIQLKYQTLQVDQIISAWVGSANRDSHQFTDGDSFNIYRGRNPHLTFGHGIHFCLGAPLARLEAKIVLTELIKRYKSFSFIDENLPTPISNSSTIYGLSSFPVKSELLHIK

>CYP109T4_ortholog(BCE_3250)Bacillus cereus ATCC 10987

MKTPYQSIDMIPTSKLIGKKALEDPYKPFAWYKEMREKEPICFNHQADMWNVFLYEDVKTVLEDKEHFSNIMPEKKKSPFPQSILGMDPPKHTQIRSIVNRSFTPKSLREWEPRIQQITNDILNQLSNRKTFDIVRELFYPLPVIVIAEMLGVSAKDMERFKKWSDIIVSSPSHDDPDYLAEFFHTRLQAENELGDFFEEIIQLNREKSQKDANDIISLLVQSEAEKSISGKELVSFCKLLLVAGNETTTNLLGNALYCFIEYPNVYEQLQQDISLIPKAIEEVLRYRSPVQRITRRVKKEIQLKYQTLQVDQIISAWVGSANRDSHQFTDGDSFNIYRGRNPHLTFGHGIHFCLGAPLARLEAKIVLTELIKRYKSFSFIDENLPTPISNSSTIYGLSSFPVKSELLHIK

>CYP109T4_ortholog(BCK_18950)Bacillus cereus FRI-35

MKTPYQSIDMIPTSKLIGKKALEDPYKPFAWYKEMREKEPICFNHQADMWNVFLYEDVKTVLEDKEHFSNIMPEKKKSPFPQSILGMDPPKHTQIRSIVNRSFTPKSLREWEPRIQQITNDILNQLSNRKTFDIVRELFYPLPVIVIAEMLGVSAKDMERFKKWSDIIVSSPSHDDPDYLAEFFHTRLQAENELGDFFKEIIQLNREKSQKDANDIISLLVQSEAEKSISGKELVSFCKLLLVAGNETTTNLLGNALYCFIEYPNVYEQLQQDISLIPKSIEEVLRYRSPVQRITRRVKKEIQLKDQTLQVDQIISAWVGSANRDSHQFTDGDSFNIYRGRNPHLTFGHGIHFCLGAPLARLEAKIVLTELIKRYKSFSFIDENLPTPISNSSTIYGLSSFPVKSELLHIK

>CYP109T5(YBT020_15665)Bacillus thuringiensis serovar finitimus YBT-020

MKTPYESIIMIPTNKLIGKKALEDPYDPFAWYKEMREEEPICFNPQVDMWNVFLYDDVKRVLEDKEYFSNIMPEKKKPPFPQSILGMDQPKHTQIRSIVNRSFTPKALEVWEPRIQEITEHILTQLSNRENFDIVHELFYPLPVIVIAEMLGVSTNDMDRFKNWSDIIVSSPSHDDPDYLKEFFHTRLQAENELENFFEEILQLNRGNANKDSNDIISLLVQSEVDEKISGKEIVSFCKLLLVAGNETTTNLLGNLLYCLIEHPDVYKQIQQDTALIPKAIEEVLRYRSPAQRVVRRVKKEMQLNGQTLQVDQIISAWVGSANRDSNYFKDADSFNIHRRRNPHLAFGHGIHFCLGARLARLEARIVLTELIKKYKSFSFIDTNLPIPISNSSSVYGLKSFPVKSEMIQIK

>CYP109T5_ortholog(BCA_3206)Bacillus cereus 03BB102

MKTPYESIIMIPTNKLIGKKALEDPYDPFAWYKEMREEEPICFNPQADMWNVFLYDDVKRVLEDKEYFSNIMPEKKKPPFPQSILGMDQPKHTQIRSIVNRSFTPKALEVWEPRIQEITEDILTQLSNRKNFDIVQELFYPLPVIVIAEMLGVSTNDMDRFKNWSDIIVSSPSHDDPDYLKEFFHTRLQAENELENFFEEILQLNRGNSNKDSNDIISLLVQSEVDEKISGKEIVSFCKLLLVAGNETTTNLLGNLLYCLIEHPDVYKQIQQDTALIPKAIEEVLRYRSPAQRVVRRVKKEMQLNGQTLQVDQIISAWVGSANRDSNYFKDADSFNIHRKRNPHLAFGHGIHFCLGARLARLEATIVLMELIKKYKSFSFINTNLPIPISNSSSVYGLKSFPVKSEMIQIK

>CYP109T6(Bcer98_3665)Bacillus cytotoxicus

MKKTYESIVMIPTNKLIEKKALENPYEPFAWYKEMREKEPICFNPQADMWNVFLYDDVKIVLEDKEHFSKIMPEKKTLPFSKSILGMNPPKHTQIRSIVNRSFTPKVLEAWEPRIQKVTEDILIQLSNRETFDIVQELFYPLPVIVTAEMLGVSANDMERFKKWSDSILSSPNHDDQDYLTEFFHMRLQAENELGEFFEEILQIKRGNSKENSNDIISLLVQNEADNKISGEEIVPFCKLLLVAGNETTTNLLGNALYCLIEHPNVYEQLQQDLSLIPKVIEEVLRYRSPAQRIVRRVKKGMQLNGQTLKVNQIVSAWIGSANRDSQYFNDADSFNIHRPRNPHLGFGYGINFCLGTRLARLVATIVLTEIIKKYKSFSFIDHNLPIPISNSRSLYGLKTFPVKSEIIHIE

>CYP109U1(BEH_09020)Bacillus endophyticus

MKQKTVGFFSRHTFSTSEENWNGRFEWYEKMRKASPITYNEEEKCWDIFLYEDIEAIIKNKEIFSSQRPVQQGEPNILSLDPPRHTQLRSLVSKAFTPRELNLWKPRIEQITENLIDDMKGKATFDLIKDLAYPLPVMVIADILGVPEEDMGDFKRWSDLLVAGPEDPTEEALQRLQKLRTQSITEMDTYFEEIIKYKRTHPKEDIISILIDAEIEGQKLTDGEIVSFCRLLLAAGNETTTNLIGNTMYGLLEEPSHYQELQQNPEYITLAIEEGLRYRSPVQALHRIAKEDKEIKGQNIRKGDSVTLWIGSANRDENKFENANHFIINRKPNPHLSFGKGIHFCLGAPLARMEGQIAFEQLTKKFSKICIPHNFELLPIQSAFVFGLKEFPLEGQEN

>CYP109V1(IJ21_13960)Paenibacillus sp. 32O-W

MKWNEEHGLLPLAHFASMRATESVAYQNGAWHVYRYEDAKAIFADPGRFSSEMSESPSPDVPIEHSILRRDPPKHRQLRALVTQAFTPRAIEALAPRIETLAHELLDKAEARGGMDAIGDFAGPLPVLVIAEMLGIPSRDREQFKEWSDALVGTDYDRFMQCQREMSDYFGRIADERKRNPQDDLISRLVQAEADGSPLAPVELIGFCILLLVAGNETTTNLLGGALLCFDDRRDDWEAIRDDRSLLPGAIEETLRYCSPVLNMTRRVRQTAVVADRKLEAGEYVHLWIGSANHDEAVFERPDRFDIRRSPNQHLAFGHGIHFCLGAQLARLEARIALDALLDRFPNFRRDRTAPLERVDSSFVFGVKHLPVLLS

>CYP109V2(GYMC10_6284)Paenibacillus sp. Y412MC10

MLSMEWNKNGLLPLEWFRQMRTESPVTAIDGGGAWNVFKYEDVKAVFTNYEVFSSQGSSSSDDPIESSVLRQDPPKHRQLRKLVSHAFTPRMIESLAPKIQEITTSLLDEAEKKGKMDIVADLASPLPITVIAEMLGVSMEYRERFKAWSDALVGDNADAYYQCQREMSEYFSEIAEDRRRHPQDDLITKLVEARIDNEHLTELEIIGFCILLLVAGNETTTNLISSAVLAFDSLPEVRAAVLGDSQLLPGAIEEVFRYFSPVQLMFRSLKQDTVLRGQELKQGQFVYIWMASANHDEDVFDQPDVFNIHRNPNPHLGLGSGIHYCMGSQLARMETRIALQTLLDRYPQFRRDRSVELARMDSTMMFALKELPVILK

>CYP109W1(PM3016_4236)Paenibacillus mucilaginosus 3016

MSASTDLRLNPYPWYQKMLQHSPIVYSEPFGAYLVFRYDDVRSVFQDYKTFSSALYDGLSTELTFDNQIQGMDPPRHTQLRALAAHAFTPKAVADLEPRIREIADYLIDAMLAGQDIDFVQQFAVPFPVRVIAEMLGVPEEDFDRFKVWSDIIVEISERLLTGQTEELPEHVAAYKEMKQYFQTMIHQRRAEPRNDLISRLAAAEVDGQQLTDLEAINFCLILLVAGNETTTNLITNLIRTFAEHPKQWKLLRQRRDLIPQAVEEVMRYRTPVQLMFRLVTQETEISGTKLKAGDRVVLYLGAANRDPAKFERPDTFDITRPASPHLTFGHGIHFCLGAPLARLETSIALQVLLDRLDAFEIPPAEALEPLTEL

>CYP109W1_ortholog(KNP414_04829)Paenibacillus mucilaginosus KNP414

MSASTDLRLNPYPWYQKMLQHSPIVYSEPFGAYLVFRYDDVRSVFQDYKTFSSALYDGLSTELTFDNQIQGMDPPRHTQLRALAAHAFTPKAVADLEPRIREIADYLIDAMLAGQDIDFVQQFAVPFPVRVIAEMLGVPEEDFDRFKVWSDIIVEISERLLTGQTEELPEHVAAYKEMKQYFQTMIHQRRAEPRNDLISRLAAAEVDGQQLTDLEAINFCLILLVAGNETTTNLITNLIRTFAEHPKQWKLLRQRRDLIPQAVEEVMRYRTPVQLMFRLVTQETEISGTKLKAGDRVVLYLGAANRDPAKFERPDTFDITRPASPHLTFGHGIHFCLGAPLARLETSIALQVLLDRLDAFEIPPAEALEPLTSFNILALRKLPLAVKG

>CYP109W1_ortholog(B2K_21965)Paenibacillus mucilaginosus K02

MLQHSPIVYSEPFGAYLVFRYDDVRSVFQDYKTFSSALYDGLSTELTFDNQIQGMDPPRHTQLRALAAHAFTPKAVADLEPRIREIADYLIDAMLAGQDIDFVQQFAVPFPVRVIAEMLGVPEEDFDRFKVWSDIIVEISERLLTGQTEELPEHVAAYKEMKQYFQTMIHQRRAEPRNDLISRLAAAEVDGQQLTDLEAINFCLILLVAGNETTTNLITNLIRTFAEHPKQWKLLRQRRDLIPQAVEEVMRYRTPVQLMFRLVTQETEISGTKLKAGDRVVLYLGAANRDPAKFERPDTFDITRPASPHLTFGHGIHFCLGAPLARLETSIALQVLLDQLDAFEIPPAEALEPLTSFNILGLRKLPIAVKG

>CYP109X1(Bcer98_1831)Bacillus cytotoxicus

MENFKMKNKGLGLVKPVKELILEQAKLDPFTWFKEMRNNTPIRYDEERGCWDVFNYNDVLNILKDYNNFSSDRPEPVLVSSIIRMDPPRHTQMRGIISNAFTPRLIKDLEPRIQDIAKILINETLPNEEMEVIQDFSYALAIIVIADLLGVPSEDHYLFKKWSDIIAKGANDDSPNALREVIREKNEVREELNVYFSKIVSRRKENPKNDLITKLVEARIEGEGLTQIEILEFCHLLLVAGNETTTNLIANLIRRIAEDDNLENQLRLNPHLIKNAIEETLRFYPPVLNTSRFAANDFNLRGHQIKKGDQVILWIASANRDEKQFKNPDTFDINRVSIKHLTFGQSIHFCLGAPLARLEAEIAIQTLLKMVRDIKFSNSKLNPIQSCLVYGCTALRIKFKVSE

>CYP113L1_ortholog(OY17_14250)Bacillus sp. BH072

MTSLTKIRQQQPYKWYQTMRETSPVHYNEKEDCWEIFTYDEVKRVISDYSHFSSDHKYLSADKQEKMIRHINKDSLLKMDPPEHTVFRKLVNQPFMPKSVESLAPRIAAIADDLLQAVRSKGRMDIIEDYAFPLPIIVIAELLGFPPKDRDIFKSWVDQSQNVKDEKKMNEVQKQMIGYFMQFILQRRKQPQNDLISHLISADLDGEPLSDKQLIGFCGLLIVAGHVTTENVIGNSFLSLKEFPHILPRLLENKALLPDFIEEVIRLRPSIQRVTRYTAVESEIGGKTIPAGEKVYAWIGSANRDEKKFENADQIDLGRKPNQHLSFGQGSHYCLGAPLARLEAKIALSHFFEQMPAWRFTEDQEPNLVPSPVFHGVDRLLVEF

>CYP113L1_ortholog(BAPNAU_1390)Bacillus velezensis NAU-B3

MTSLTKIRQQQPYKWYQTMRETSPVHYNEKEDCWEIFTYDEVKRVISDYSHFSSDHKYLSADKQEKMIRHINKDSLLKMDPPEHTVFRKLVNQPFMPKSVESLAPRIAAIADDLLQAVRSKGRMDIIEDYAFPLPIIVIAELLGFPPKDRDIFKSWVDQSQNVKDEKKMNEVQKQMIGYFMQFILQRRKQPQNDLISHLISADLDGEPLSDKQLIGFCGLLIVAGHVTTENVIGNSFLSLKEFPHILPRLLENKALLPDFIEEVIRLRPSIQRVTRYTAVESEIGGKTIPAGEKVYAWIGSANRDEKKFENADQIDLGRKPNQHLSFGQGSHYCLGAPLARLEAKIALSHFFEQMPAWRFTEDQEPNLVPSPVFHGVDRLLVEF

>CYP113L1_ortholog(KSO_008415)Bacillus amyloliquefaciens IT-45

MTSLTKIRQQQPYKWYQTMRETSPVHYNEKEDCWEIFTYDEVKRVISDYSHFSSDHKYLSADKQEKMIRHINKDSLLKMDPPEHTVFRKLVNQPFMPKSVESLAPRIAAIADDLLQAVRSKGRMDIIEDYAFPLPIIVIAELLGFPPKDRDIFKSWVDQSQNVKDEKKMNEVQKQMIGYFMQFILQRRKQPQNDLISHLISADLDGEPLSDKQLIGFCGLLIVAGHVTTENVIGNSFLSLKEFPHILPRLLENKALLPDFIEEVIRLRPSIQRVTRYTAVESEIGGKTIPAGEKVYAWIGSANRDEKKFENADQIDLGRKPNQHLSFGQGSHYCLGAPLARLEAKIALSHFFEQMPAWRFTEDQEPNLVPSPVFHGVDRLLVEF

>CYP113L1_ortholog(BACAU_2207)Bacillus velezensis CAU B946

MTSLTKIRQQQPYKWYQTMRETSPVHYNEKEDCWEIFTYDEVKRVISDYSHFSSDHKYLSADKQEKMIRHINKDSLLKMDPPEHTVFRKLVNQPFMPKSVESLAPRIAAIADDLLQAVRSKGRMDIIEDYAFPLPIIVIAELLGFPPKDRDIFKSWVDQSQNVKDEKKMNEVQKQMIGYFMQFILQRRKQPQNDLISHLISADLDGEPLSDKQLIGFCGLLIVAGHVTTENVIGNSFLSLKEFPHILPRLLENKALLPDFIEEVIRLRPSIQRVTRYTAVESEIGGKTIPAGEKVYAWIGSANRDEKKFENADQIDLGRKPNQHLSFGQGSHYCLGAPLARLEAKIALSHFFEQMPAWRFTEDQEPNLVPSPVFHGVDRLLVEF

>CYP113L1_ortholog(NG74_02300)Bacillus velezensis

MTSLTKIRQQQPYKWYQTMRETSPVHYNEKEDCWEIFTYDEVKRVISDYSHFSSDHKYLSADKQEKMIRHINKDSLLKMDPPEHTVFRKLVNQPFMPKSVESLAPRIAAIADDLLQAVRSKGRMDIIEDYAFPLPIIVIAELLGFPPKDRDIFKSWVDQSQNVKDEKKMNEVQKQMIGYFMQFILQRRKQPQNDLISHLISADLDGEPLSDKQLIGFCGLLIVAGHVTTENVIGNSFLSLKEFPHILPRLLENKALLPDFIEEVIRLRPSIQRVTRYTAVESEIGGKTIPAGEKVYAWIGSANRDEKKFENADQIDLGRKPNQHLSFGQGSHYCLGAPLARLEAKIALSHFFEQMPAWRFTEDQEPNLVPSPVFHGVDRLLVEF

>CYP113L1_ortholog(MUS_2635)Bacillus amyloliquefaciens Y2

MKMTSLTKIRQQQPYKWYQTMRETSPVHYNEKEDCWEIFTYDEVKRVISDYSHFSSDHKYLSADKQEKMIRHINKDSLLKMDPPEHTVFRKLVNQPFMPKSVESLAPRIAAIADDLLQAVRSKGRMDIIEDYAFPLPIIVIAELLGFPPKDRDIFKSWVDQSQNVKDEKKMNEVQKQMIGYFMQFILQRRKQPQNDLISHLISADLDGEPLSDKQLIGFCGLLIVAGHVTTENVIGNSFLSLKEFPHILPRLLENKALLPDFIEEVIRLRPSIQRVTRYTAVESEIGGKTIPAGEKVYAWIGSANRDEKKFENADQIDLGRKPNQHLSFGQGSHYCLGAPLARLEAKIALSHFFEQMPAWRFTEDQEPNLVPSPVFHGVDRLLVEF

>CYP113L1_ortholog(BANAU_2348)Bacillus velezensis YAU B9601-Y2

MTSLTKIRQQQPYKWYQTMRETSPVHYNEKEDCWEIFTYDEVKRVISDYSHFSSDHKYLSADKQEKMIRHINKDSLLKMDPPEHTVFRKLVNQPFMPKSVESLAPRIAAIADDLLQAVRSKGRMDIIEDYAFPLPIIVIAELLGFPPKDRDIFKSWVDQSQNVKDEKKMNEVQKQMIGYFMQFILQRRKQPQNDLISHLISADLDGEPLSDKQLIGFCGLLIVAGHVTTENVIGNSFLSLKEFPHILPRLLENKALLPDFIEEVIRLRPSIQRVTRYTAVESEIGGKTIPAGEKVYAWIGSANRDEKKFENADQIDLGRKPNQHLSFGQGSHYCLGAPLARLEAKIALSHFFEQMPAWRFTEDQEPNLVPSPVFHGVDRLLVEF

>CYP113L1_ortholog(SB24_17745)Bacillus sp. Pc3

MTSLTKIRQQQPYKWYQTMRETSPVHYNEKEDCWEIFTYDEVKRVISDYSHFSSDHKYLSADKQEKMIRHINKDSLLKMDPPEHTVFRKLVNQPFMPKSVESLAPRIAAIADDLLQAVRSKDRMDIIEDYAFPLPIIVIAELLGFPPKDRDIFKSWVDQSQNVKDEKKMNEVQKQMIGYFMQFILQRRKQPQNDLISHLISADLDGEPLSDKQLIGFCGLLIVAGHVTTENVIGNSFLSLKEFPHILPRLLENKALLPDFIEEVIRLRPSIQRVTRYTAVESEIGGKTIPAGEKVYAWIGSANRDEKKFENADQIDLGRKPNQHLSFGQGSHYCLGAPLARLEAKIALSHFFEQMPAWRFTEDQEPNLVPSPVFHGVDRLLVEF

>CYP113L1_ortholog(U722_11960)Bacillus amyloliquefaciens LFB112

MTSLTKIRQQQPYKWYQTMRETSPVHYNEKEDCWEIFTYDEVKRVISDYSHFSSDHKYLSADKQEKMIRHINKDSLLKMDPPEHTVFRKLVNQPFMPKSVESLAPRIAAIADDLLQAVRSKGRMDIIEDYAFPLPIIVIAELLGFPPKDRDIFKSWVDQSQNVKDEKKMNEVQKQMIGYFMQFILQRRKQPQNDLISHLISADLDGEPLSDKQLIGFCGLLIVAGHVTTENVIGNSFLSLKEFPHILPRLLENKALLPDFIEEVIRLRPSIQRVTRYTAVESEIGGKTIPAGEKVYAWIGSANRDEKKFENPDEIDLGRKPNQHLSFGQGSHYCLGAPLARLEAKIALSHFFEQMPAWRFTEDQEPNLVPSPVFHGVDRLLVEF

>CYP113L1_ortholog(AJ82_12450)Bacillus velezensis TrigoCor1448

MTSLTKIRQQQPYKWYQTMRETSPVHYNEKEDCWEIFTYDEVKRVISDYSHFSSDHKYLSADKQEKMIRHINKDSLLKMDPPEHTVFRKLVNQPFMPKSVESLEPRIAAIADDLLQAVRSKGRMDIIEDYAFPLPIIVIAELLGFPPKDRDIFKSWVDQSQNVKDEKKMNEVQKQMIGYFMQFILQRRKQPQNDLISHLISADLDGEPLSDKQLIGFCGLLIVAGHVTTENVIGNSFLSLKEFPHILPRLLENKALLPDFIEEVIRLRPSIQRVTRYTAVESEIGGKTIPAGEKVYAWIGSANRDEKKFENADQIDLGRKPNQHLSFGQGSHYCLGAPLARLEAKIALSHFFEQVPAWRFTEDQEPNLVPSPVFHGVDRLLVEF

>CYP113L1_ortholog(B938_11350)Bacillus velezensis AS43.3

MTSLTKIRQQQPYKWYQTMRETSPVHYNEKEDCWEIFTYDEVKRVISDYSHFSSDHKYLSADKQEKMIRHINKDSLLKMDPPEHTVFRKLVNQPFMPKSVETLAPRIAAIADDLLQTVRSKDRMDIIEDYAFPLPIIVIAELLGFPPKDRDIFKSWVDQSQNVKDEKKMNEVQKQMIGYFMQFILQRRKQPQNDLISHLISADLDGEPLSDKQLIGFCGLLIVAGHVTTENVIGNSFLSLKEFPHILPRLLENKALLPDFIEEVIRLRPSIQRVTRYTAVESEIGGKTIPAGEKVYAWIGSANRDEKKFENADQIDLGRKPNQHLSFGQGSHYCLGAPLARLEAKIALSHFFEQVPAWRFTEDQEPNLVPSPVFHGVDRLLVEF

>CYP134A1_ortholog(BS34A_38160)Bacillus sp. BS34A

MSQSIKLFSVLSDQFQNNPYAYFSQLREEDPVHYEESIDSYFISRYHDVRYILQHPDIFTTKSLVERAEPVMRGPVLAQMHGKEHSAKRRIVVRSFIGDALDHLSPLIKQNAENLLAPYLERGKSDLVNDFGKTFAVCVTMDMLGLDKRDHEKISEWHSGVADFITSISQSPEARAHSLWCSEQLSQYLMPVIKERRVNPGSDLISILCTSEYEGMALSDKDILALILNVLLAATEPADKTLALMIYHLLNNPEQMNDVLADRSLVPRAIAETLRYKPPVQLIPRQLSQDTVVGGMEIKKDTIVFCMIGAANRDPEAFEQPDVFNIHREDLGIKSAFSGAARHLAFGSGIHNCVGAAFAKNEIEIVANIVLDKMRNIRLEEDFCYAESGLYTRGPVSLLVAFDGA

>CYP134A1_ortholog(U712_17560)Bacillus subtilis PY79

MSQSIKLFSVLSDQFQNNPYAYFSQLREEDPVHYEESIDSYFISRYHDVRYILQHPDIFTTKSLVERAEPVMRGPVLAQMHGKEHSAKRRIVVRSFIGDALDHLSPLIKQNAENLLAPYLERGKSDLVNDFGKTFAVCVTMDMLGLDKRDHEKISEWHSGVADFITSISQSPEARAHSLWCSEQLSQYLMPVIKERRVNPGSDLISILCTSEYEGMALSDKDILALILNVLLAATEPADKTLALMIYHLLNNPEQMNDVLADRSLVPRAIAETLRYKPPVQLIPRQLSQDTVVGGMEIKKDTIVFCMIGAANRDPEAFEQPDVFNIHREDLGIKSAFSGAARHLAFGSGIHNCVGAAFAKNEIEIVANIVLDKMRNIRLEEDFCYAESGLYTRGPVSLLVAFDGA

>CYP134A1_ortholog(B657_35060)Bacillus subtilis QB928

MSQSIKLFSVLSDQFQNNPYAYFSQLREEDPVHYEESIDSYFISRYHDVRYILQHPDIFTTKSLVERAEPVMRGPVLAQMHGKEHSAKRRIVVRSFIGDALDHLSPLIKQNAENLLAPYLERGKSDLVNDFGKTFAVCVTMDMLGLDKRDHEKISEWHSGVADFITSISQSPEARAHSLWCSEQLSQYLMPVIKERRVNPGSDLISILCTSEYEGMALSDKDILALILNVLLAATEPADKTLALMIYHLLNNPEQMNDVLADRSLVPRAIAETLRYKPPVQLIPRQLSQDTVVGGMEIKKDTIVFCMIGAANRDPEAFEQPDVFNIHREDLGIKSAFSGAARHLAFGSGIHNCVGAAFAKNEIEIVANIVLDKMRNIRLEEDFCYAESGLYTRGPVSLLVAFDGA

>CYP134A1_ortholog(BSU35060)Bacillus subtilis subsp. subtilis 168

MSQSIKLFSVLSDQFQNNPYAYFSQLREEDPVHYEESIDSYFISRYHDVRYILQHPDIFTTKSLVERAEPVMRGPVLAQMHGKEHSAKRRIVVRSFIGDALDHLSPLIKQNAENLLAPYLERGKSDLVNDFGKTFAVCVTMDMLGLDKRDHEKISEWHSGVADFITSISQSPEARAHSLWCSEQLSQYLMPVIKERRVNPGSDLISILCTSEYEGMALSDKDILALILNVLLAATEPADKTLALMIYHLLNNPEQMNDVLADRSLVPRAIAETLRYKPPVQLIPRQLSQDTVVGGMEIKKDTIVFCMIGAANRDPEAFEQPDVFNIHREDLGIKSAFSGAARHLAFGSGIHNCVGAAFAKNEIEIVANIVLDKMRNIRLEEDFCYAESGLYTRGPVSLLVAFDGA

>CYP134A1_ortholog(Q433_19190)Bacillus subtilis subsp. subtilis OH 131.1

MSQSIKLFSVLSDQFQNNPYAYFSQLREEDPVHYEESIDSYFISRYHDVRYILQHPDIFTTKSLVERAEPVMRGPVLAQMHGKEHSAKRRIVVRSFIGDALDHLSPLIKQNAENLLAPYLERGKIDLVNDFGKTFAVCVTMDMLGLDKRDHEKISEWHSGVADFITSISQSPEARAHSLWCSEQLSQYLMPVIKERRVNPGSDLISILCTSEYEGMALSDKDILALILNVLLAATEPADKTLALMIYHLLNNPEQMNDVLADRSLVPRAIAETLRYKPPVQLIPRQLSQDTVVGGMEIKKDTIVFCMIGAANRDPEAFEQPDVFNIHREDLGIKSAFSGGARHLAFGSGIHNCVGAAFAKNEIKIVANIVLDKMRNIRLEEDFCYAESGLYTRGPVSLLVAFDGA

>CYP134A1_ortholog(GYO_3843)Bacillus subtilis subsp. spizizenii TU-B-10

MNQSIKLFSVLSDQFQENPYAYFSQLREEDPVHYEESIDSYFISRYHDVRYILQHPDIFTTKSLVQRAEPVMRGPVLAQMHGKEHSAKRRIVVRSFVGDALDHLSPLIKQNAENLLAPYLERGRIDLVNDFGKTFAVCVTMDMLGLDKRDHEKIAEWHSGVADFITSISQTPEARAHSLWCSEQLSQYLMPVIEERRVNPGSDLISILCTSEYEGMAMSDKDILALILNVLLAATEPADKTLALMIYHLLNNPEQMNDVLADRSLVPRAIAETLRYKPPVQLIPRQLSQDTVVGGMEIKKDTIVFCMIGAANRDPEAFERPDVFYIHREDLGIKSAFSGAARHLAFGSGIHNCVGAAFAKTEIEIVANIVLDKMRNIRLEEGFRYAESGLYTRGPVSLHVAFDRA

>CYP134A1_ortholog(BSUW23_17185)Bacillus subtilis subsp. spizizenii W23

MNQSIKLFSVLSDQFQENPYAYFSQLREEDPVHYEESIDSYFISRYHDVRYILQHPDIFTTKSLVERAEPVMRGPVLAQMHGKEHSAKRRIVVRSFVGDALDHLSPLIKQNAENLLAPYLERGRIDLVNDFGKTFAVCVTMDMLGLDKRDHEKIAEWHSGVADFITSLSQTPEARAHSLWCSEQLSQYLMPVIEERRVNPGSDLISILCTSEYEGMAMSDKDILALILNVLLAATEPADKTLALMIYHLLNNPDQMNDVLADRSLVPRAIAETLRYKPPVQLIPRQLSQDTVIGGMEIKKDTIVFCMIGAANRDPEAFERPDVFHIHREDLGIKSAFSGAARHLAFGSGIHNCVGAAFAKTEIEIVANIVLDKMRNIRLEEGFRYAESGLYTRGPVSLHVAFDRA

>CYP134A5(BaLi_c36290)Bacillus paralicheniformis

MNQSLKTFSVLSEQYHENPYQYFSYLRESDPVHYEESLDSYFISRYQDVRRVLQNQDVFTTKSLAKRAEPVMRGPVLAQMKGKEHTAKRRIVLRRFIGESLDHLTPLIKENAQRLLAPHLEKGRIDLVNDFGKTFAVCVTMDILGLDKNDHQTVRNWHSGVADFITSLNQAPEDREHSLKCSEQLAEYLNPIIEERRKNPGHDLISILCTSEYEGVAMSDRDIRALILNILLAATEPADKTLALMIYHLLHHPDQMNDVLEDRTLLPQAIAETLRYKPPVQLIPRQLSEDAVIGGVELKEGTTVFCMIGAANRDPEAFEEPDEFNIHRTDLEVKSAFSGAARHLAFGSGIHNCVGAGFAKTEIELVANIVLDQLKNIQLEEDFVYRETGLYTRGPVSLNIRFDTKH

>CYP134A5_ortholog(BLi03567)Bacillus licheniformis DSM 13 = ATCC 14580

MNQSLKTFSVLSEQYHENPYQYFSYLRESDPVHYEESLDSYFISRYQDVRRVLQNQDVFTTKSLAKRAEPVMRGPVLAQMKGKEHTAKRRIVLRRFIGESLDHLTPLIKENAQRLLAPHVEKGRIDLVNDFGKTFAVCVTMDILGLDKNDHQTVRNWHSGVADFITSLNQAPEDREHSLKCSEQLAEYLNPIIEERRKNPGHDLISILCTSEYEGVAMSDRDIRALILNILLAATEPADKTLALMIYHLLHHPDQMNDVLEDRTLLPQAIAETLRYKPPVQLIPRQLSQDAEIGGVELKEGTTVFCMIGAANRDPEAFEDPDKFNIHRSDLEVKSAFSGAARHLAFGSGVHNCVGAGFAKTEIELVANIVLDQLKNIRLEEDFIYRETGLYTRGPVSLNIRFDAKH

>CYP134A5_ortholog(BL00818)Bacillus licheniformis ATCC 14580

MNQSLKTFSVLSEQYHENPYQYFSYLRESDPVHYEESLDSYFISRYQDVRRVLQNQDVFTTKSLAKRAEPVMRGPVLAQMKGKEHTAKRRIVLRRFIGESLDHLTPLIKENAQRLLAPHVEKGRIDLVNDFGKTFAVCVTMDILGLDKNDHQTVRNWHSGVADFITSLNQAPEDREHSLKCSEQLAEYLNPIIEERRKNPGHDLISILCTSEYEGVAMSDRDIRALILNILLAATEPADKTLALMIYHLLHHPDQMNDVLEDRTLLPQAIAETLRYKPPVQLIPRQLSQDAEIGGVELKEGTTVFCMIGAANRDPEAFEDPDKFNIHRSDLEVKSAFSGAARHLAFGSGVHNCVGAGFAKTEIELVANIVLDQLKNIRLEEDFIYRETGLYTRGPVSLNIRFDAKH

>CYP134A6(CY96_28935)Bacillus bombysepticus

MSNTIQTVNILTEEFQENPYKYFSYLRQNDPVHYEAEIDSYFISRYQDVRNILNDTETFTTKSLAERAEPVMRGPVLAQMRGKEHVAKRKIVLRSFMGDALQKLMPLIKKNAEDLLFPHLPNGKIDLINDFGRTFAVYVTMDMIGLDKKDHKKIGEWHSGVADFITSINQPPEAKKHSLWCSEQLANYLEPIIKERRLNPQEDLISKLCSAKYEGIAMTDTDILALILNILLAATEPGDKTLALLIYNLINQPKQLQDVLSDRSLVPLAIAETLRYNPPVQLIPRQLSKDTEISGIQLSKGTTVFCMIGAANRDPNAFERPDEFNIYRPDLDIKKAFSGAARHLAFGSGIHNCVGAAFAKSEIEIVVNVVLDNMKNIKLEEDFQYVEKGLYTRGPISMPILFDKLV

>CYP134C1(SPSE_0788)Staphylococcus pseudintermedius ED99

MLKQSKSIFDEAYEINPIPYFNYFREYAPIHYEESVNAYFVSNYEDVKFILKSDGIFTTKTLAERAEPVMKDRVLAQMTGHEHRSKKKEILKGMSGKYLDRLLPTIEKRTNNLIDKYINEKRIDLVNDFGKIFAVQSSMDLLGIDIRDSDKIREWHNGIAKFITSFNMSSEDVKYSLSCSDKLEKYLMPLIKERKGIKKLDLISVLNDYKDEKSQITDSEILALILNILLAATEPVDKTLAYLFYNLLNNPNQYQDILDNPSLLKNAIIETLRFNSPVQLIPRQLSMPYTFRDKKLNVDDVVICMIGAANRDPKAYTAPDEFNIYRKSKNNKPFTSYSQNLSFGFGAHTCIGATYALIQLELVVRTLLKRLKNIKLETNVLHERGIYTRGPKSLIISFD

>CYP134C1_ortholog(SPSINT_1707)Staphylococcus pseudintermedius HKU10-03

MLKQSKSIFDEAYEINPIPYFNYFREYAPIHYEESVNAYFVSNYEDVKFILKSDGIFTTKTLAERAEPVMKDRVLAQMTGHEHRSKKKEILKGMSGKYLDRLLPTIEKRTNNLIDKYINEKRIDLVNDFGKIFAVQSSMDLLGIDIRDSDKIREWHNGIAKFITSFNMSSEDVKYSLSCSDKLEKYLMPLIKERKGIKKLDLISVLNDYKDEKSQITDSEILALILNILLAATEPVDKTLAYLFYNLLNNPNQYQDILDNPSLLKNAIIETLRFNSPVQLIPRQLSMPYTFRDKKLNVDDVVICMIGAANRDPKAYTAPDEFNIYRKSKNNKPFTSYSQNLSFGFGAHTCIGATYALIQLELVVRTLLKRLKNIKLETNVLHERGIYTRGPKSLIINFD

>CYP134C2(pSHaeC07)Staphylococcus haemolyticus JCSC1435

MLKVSKSIFDKEYEINPISYFQFFRENDPVHYEKSVDAYFISNYTDVKYVLKNNDTFTTKTLAERAEPVMKDRVLAQMSGHEHKSKKKAILKGMTGKYLEKLMPILETRTNNIIDQFIDKKEIDIVNDFGKVFAVQSSMDLLGINVEDSEKIREWHNGIAKFITSFNLSDEEIKYSMHCSDKLEEYLMPLIEERKYSNKDDLISLLHEYKDAENEITDTEILALTLNILLAATEPVDKTLAYLFYNLLNSPNQYQDVIDNPKLLRSAITETLRFNSPVQLIPRQLSEPCTLHNKNLKAGDTVICMIGAANRDPKAYTNPDIFNIHRKSESNKPFTSHSQNLSFGSGVHTCVGASFSLIQLEMVTKILLKRLKNIKLKTMNLEEKGLYTRGPKSMVISFD

>CYP134C2_ortholog(DP17_2386)Staphylococcus epidermidis SEI

MLKVSKSIFDKEYEINPISYFQFFRENDPVHYEKSVDAYFISNYTDVKYVLKNNDTFTTKTLAERAEPVMKDRVLAQMSGHEHKSKKKAILKGMTGKYLEKLMPILETRTNNIIDQFIDKKEIDIVNDFGKVFAVQSSMDLLGINVEDSEKIREWHNGIAKFITSFNLSDEEIKYSMHCSDKLEEYLMPLIEERKYSNKDDLISLLHEYKDAENEITDTEILALTLNILLAATEPVDKTLAYLFYNLLNSPNQYQDVIDNPKLLRSAITETLRFNSPVQLIPRQLSEPCTLHNKNLKAGDTVICMIGAANRDPKAYTNPDIFNIHRKSESNKPFTSHSQNLSFGLGVHTCVGASFSLIQLEMVTKILLKRLKTMNLEEKGLYTRGPKSMVISFD

>CYP152A1_ortholog(BSU6051_02100)Bacillus subtilis subsp. subtilis 6051-HGW

MNEQIPHDKSLDNSLTLLKEGYLFIKNRTERYNSDLFQARLLGKNFICMTGAEAAKVFYDTDRFQRQNALPKRVQKSLFGVNAIQGMDGSAHIHRKMLFLSLMTPPHQKRLAELMTEEWKAAVTRWEKADEVVLFEEAKEILCRVACYWAGVPLKETEVKERADDFIDMVDAFGAVGPRHWKGRRARPRAEEWIEVMIEDARAGLLKTTSGTALHEMAFHTQEDGSQLDSRMAAIELINVLRPIVAISYFLVFSALALHEHPKYKEWLRSGNSREREMFVQEVRRYYPFGPFLGALVKKDFVWNNCEFKKGTSVLLDLYGTNHDPRLWDHPDEFRPERFAEREENLFDMIPQGGGHAEKGHRCPGEGITIEVMKASLDFLVHQIEYDVPEQSLHYSLARMPSLPESGFVMSGIRRKS

>CYP152A1_ortholog(B657_02100)Bacillus subtilis QB928

MNEQIPHDKSLDNSLTLLKEGYLFIKNRTERYNSDLFQARLLGKNFICMTGAEAAKVFYDTDRFQRQNALPKRVQKSLFGVNAIQGMDGSAHIHRKMLFLSLMTPPHQKRLAELMTEEWKAAVTRWEKADEVVLFEEAKEILCRVACYWAGVPLKETEVKERADDFIDMVDAFGAVGPRHWKGRRARPRAEEWIEVMIEDARAGLLKTTSGTALHEMAFHTQEDGSQLDSRMAAIELINVLRPIVAISYFLVFSALALHEHPKYKEWLRSGNSREREMFVQEVRRYYPFGPFLGALVKKDFVWNNCEFKKGTSVLLDLYGTNHDPRLWDHPDEFRPERFAEREENLFDMIPQGGGHAEKGHRCPGEGITIEVMKASLDFLVHQIEYDVPEQSLHYSLARMPSLPESGFVMSGIRRKS

>CYP152A1_ortholog(BSUA_00258)Bacillus subtilis subsp. subtilis JH642

MNEQIPHDKSLDNSLTLLKEGYLFIKNRTERYNSDLFQARLLGKNFICMTGAEAAKVFYDTDRFQRQNALPKRVQKSLFGVNAIQGMDGSAHIHRKMLFLSLMTPPHQKRLAELMTEEWKAAVTRWEKADEVVLFEEAKEILCRVACYWAGVPLKETEVKERADDFIDMVDAFGAVGPRHWKGRRARPRAEEWIEVMIEDARAGLLKTTSGTALHEMAFHTQEDGSQLDSRMAAIELINVLRPIVAISYFLVFSALALHEHPKYKEWLRSGNSREREMFVQEVRRYYPFGPFLGALVKKDFVWNNCEFKKGTSVLLDLYGTNHDPRLWDHPDEFRPERFAEREENLFDMIPQGGGHAEKGHRCPGEGITIEVMKASLDFLVHQIEYDVPEQSLHYSLARMPSLPESGFVMSGIRRKS

>CYP152A1_ortholog(BSUB_00258)Bacillus subtilis subsp. subtilis AG1839

MNEQIPHDKSLDNSLTLLKEGYLFIKNRTERYNSDLFQARLLGKNFICMTGAEAAKVFYDTDRFQRQNALPKRVQKSLFGVNAIQGMDGSAHIHRKMLFLSLMTPPHQKRLAELMTEEWKAAVTRWEKADEVVLFEEAKEILCRVACYWAGVPLKETEVKERADDFIDMVDAFGAVGPRHWKGRRARPRAEEWIEVMIEDARAGLLKTTSGTALHEMAFHTQEDGSQLDSRMAAIELINVLRPIVAISYFLVFSALALHEHPKYKEWLRSGNSREREMFVQEVRRYYPFGPFLGALVKKDFVWNNCEFKKGTSVLLDLYGTNHDPRLWDHPDEFRPERFAEREENLFDMIPQGGGHAEKGHRCPGEGITIEVMKASLDFLVHQIEYDVPEQSLHYSLARMPSLPESGFVMSGIRRKS

>CYP152A1_ortholog(BsLM_0214)Bacillus sp. LM 4-2

MNEQIPHDKSLDNSLILLKEGYLFIKNRTERYNSDLFQARLLGKNFICMTGAEAAKVFYDTDRFQRQNALPKRVQKSLFGVNAIQGMDGSAHIHRKMLFLSLMTPPHQKRLAELMTEEWKAAVTRWEKADEVVLFEEAKEILCRVACYWAGVPLKETEVKERADDFIDMVDAFGAVGPRHWKGRRARPRAEEWIEVMIEDARAGLLKTTSGTALHEMAFHTQEDGSQLDSRMAAIELINVLRPIVAISYFLVFSALALHEHPKYKEWLRSGNSREREMFVQEVRRYYPFGPFLGALVKKDFIWNNCEFKKGTSVLLDLYGTNHDPRLWDHPDEFRPERFAEREENLFDMIPQGGGHAEKGHRCPGEGITIEVMKASLDFLVHQIEYDVPEQSLHYSLARMPSLPESGFVMSGIRRKS

>CYP152A1_ortholog(QF06_00005)Bacillus sp. YP1

MNEQIPHDKSLDNSLTLLKEGYLFIKNRTERYNSDLFQARLLGKNFICMTGAEAAKVFYDTDRFQRQNALPKRVQKSLFGVNAIQGMDGSAHIHRKMLFLSLMTPPHQKRLAELMTEEWKAAVTRWEKADEVVLFEEAKEILCRVACYWAGVPLKETEVKERADDFIDMVDAFGAVGPRHWKGRRARPRAEEWIEVMIEDARAGLLKTTPGTALHEMAFHTQEDGSQLDSRMAAIELINVLRPIVAISYFLVFSALALHEHPKYKEWLRSGNSREREMFVQEVRRYYPFGPFLGALVKKDFVWNNCEFKKGTSVLLDVYGTNHDPRLWDHPDEFRPERFAEREENPFDMIPQGGGHAEKGHRCPGEGITIEVMKASLDFLVHQIEYDVPEQSLHYSLARMPSLPESGFVMSGIRRKS

>CYP152A1_ortholog(A7A1_3272)Bacillus subtilis subsp. subtilis BSP1

MNEQIPHDKSLDNSLTLLKEGYLFIKNRTERYNSDLFQARLLGKNFICMTGAEAAKVFYDTDRFQRQNALPKRVQKSLFGVNAIQGMDGSAHIHRKMLFLSLMTPPHQKRLAELMTEEWKAAVTRWEKADEVVLFEEAKEILCRVACYWAGVPLKETEVKERADDFIDMVDAFGAVGPRHWKGRRARPRAEEWIEVMIEDARAGLLKTTPGTALHEMAFHTQEDGSQLDSRMAAIELINVLRPIVAISYFLVFSALALHEHPKYKEWLRSGNSREREMFVQEVRRYYPFGPFLGALVKKDFVWNNCEFKKGTSVLLDLYGTNHDPRLWDHPNEFRPERFAEREENLFDMIPQGGGHAEKGHRCPGEGITIEVMKASLDLLVHQIEYDVPEQSLHYSLARMPSLPESGFVMSGIRRKR

>CYP152A1_ortholog(I33_0247)Bacillus subtilis subsp. subtilis RO-NN-1

MNEQIPHDKSLDNSLTLLKEGYLFIKNRTERYNSDLFQARLLGKNFICMTGADAAKVFYDTDRFQRQNALPKRVQKSLFGVNAIQGMDGSAHIHRKMLFLSLMTPPHQKRLAELMTEEWKAAVTRWEKADEVVLFEEAKEILCRVACYWAGVPLKETEVKERADDFIDMVDAFGAVGPRHWKGRRARPRAEEWIEVMIEDARAGLLKTTPGTALHEMAFHKQEDGSQLDSRMAAIELINVLRPIVAISYFLVFSALALHEHPKYKEWLRSGNSREREMFVQEVRRYYPFGPFLGALVKKDFVWNNCEFKKGTSVLLDVYGTNHDPRLWDHPDEFRPERFAEREENPFDMIPQGGGHAEKGHRCPGEGITIEVMKASLDFLVHQIEYDVPEQSLHYSLARMPSLPESGFVMSGIRRKS

>CYP152A1_ortholog(BSNT_06509)Bacillus subtilis subsp. natto BEST195

MNEQIPHDKSLDNSLTLLKEGYLFIKNRTERYNSDLFQARLLGKNFICMTGAEAAKVFYDTDRFQRQNALPKRVQKSLFGVNAIQGMDGSAHIHRKMLFLSLMTPPHQKRLAELMTEEWRAAVTRWEKADEVVLFEEAKEILCRVACYWAGVPLKETEVKERAADFIDMVDAFGAVGPRHWKGRRARPRAEEWIEVMIEDARAGLLKTTPGTALHEMAFHTQEDGSQLDSRMAAIELINVLRPIVAISYFLVFSALALHEHPKYKEWLRSGNSRKREMFVQEVRRYYPFGPFLGALVKKDFVWNNCEFKKGTSVLLDVYGTNHDPRLWDHPDEFRPERFAEREENPFDMIPQGGGHVEKGHRCPGEGITIEVMKASLDFLVHQIEYDVPEQSLHYSLARMPSLPESGFVMSGIRRKS

>CYP152A1_ortholog(Q433_01315)Bacillus subtilis subsp. subtilis OH 131.1

MNEQIPHDKSLDNSLTLLKEGYLFIKNRTERYNSDLFQARLLGKNFICMTGAEAAKVFYDTDRFQRQNALPKRVQKSLFGVNAIQGMDGSAHIHRKMLFLSLMTPPHQKRLAELMTEEWRAAVTRWEKTDEVVLFEEAKEILCRVACYWAGVPLKETEVKERADDFIDMVDAFGAVGPRHWKGRRARPRAEEWIEVMIEDARAGLLKTTPGTALHEMAFHTQEDGSQLDSRMAAIELINVLRPIVAISYFLVFSALALHEHPKYKEWLRSGNSREREMFVQEVRRYYPFGPFLGALVKKDFVWNNCEFKKGTSVLLDVYGTNHDPRLWEHPDEFRPERFAEREENPYDMIPQGGGHAEKGHRCPGEGITIEVMKASLDFLVHQIEYDVPEQSLHYSLARMPSLPESGFVMSGIRRKS

>CYP152A1_ortholog(BSn5_12635)Bacillus subtilis BSn5

MNEQIPHDKSLDNSLTLLKEGYLFIKNRTERYNSDLFQARLLGKNFICMTGAEAAKVFYDTDRFQRQNALPKRVQKSLFGVNAIQGMDGSAHIHRKMLFLSLMTPPHQKRLAELMTEEWKAAVTRWEKADEVVLFEEAKEILCRVACYWADVPLKETEVKERADDFIDMVDAFGAVGPRHWKGRRARPRAEEWIEVMIEDARAGLLKTTPGTALHEMAFHKQEDGSELDSRMAAIELINVLRPIVAISYFLVFSALALHEHPKYKEWLRSGSSREREMFVQEVRRYYPFGPFLGALVKKDFVWNNCEFKKGTSVLLDVYGTNHDPRLWDHPDEFRPERFAEREENPFDLIPQGGGHAEKGHRCPGEGITIEVMKASLDFLVNQIEYDVPEQSLYYSLARMPSLPESGFVMSGIRRKR

>CYP152A1_ortholog(MY9_0209)Bacillus sp. JS

MNEQIPHDKSLDNSLTLLKEGYLFIKNRTERYNSDLFQARLLGKNFICMTGAEAAKVFYDTNRFQRQNALPKRVQKSLFGVNAIQGMDGSAHIHRKMLFLSLMTPPHQKRLAELMTEEWKAAVTRWEKADQVVLFEEAKEILCRVACYWAGVPLKETEVKERADDFIDMVDAFGAVGPRHWKGRRARPRAEEWIEVMIEDVRAGLLKTPAGTALHEMAFHKQEDGSELDSRMASIELINVLRPIVAISYFLVFSALALHEHPKYKEWLRSGSSREREMFVQEVRRYYPFGPFLGALVKKDFVWNNCEFKKGTSVLLDVYGTNHDPRLWDHPDEFRPERFAEREENPFDLIPQGGGHAEKGHRCPGEGITIEVMKASLDFLVNQIEYDVPEQSLHYSLARMPSLPESGFIMSGIRQKR

>CYP152A1_ortholog(GYO_0400)Bacillus subtilis subsp. spizizenii TU-B-10

MNEQIPHDKSLDNSLTLMQEGYLFIKNRTERYHSDLFQARLLGKTFICMTGAEAAKLFYDTERFQRQNALPKRVQKTLFGVNAIQTMDGDAHIHRKLLFLSLMTPPHQKRLAELMTEEWEAAVTRWEKADQVVLFEEAKEILCRVACYWAGVPLKETEVKERADDFIDMVDAFGAVGPRHWKGRRARPRAEEWIERMIEDVRAGLLKIPAGTALHEMAFHTQEDGNQLDSRMAAIELINVLRPIVAISYFLVFSALALHKHPKYKEWLRSGSNREREMFVQEVRRYYPFGPFLGALVKKDFEWNTCEFKKGTSVLLDVYGTNHDPRLWDNPDEFRPERFEEREENPFDLIPQGGGHAEKGHRCPGEGITIEVMKASLDFLVNQIEYDVPEQSLHYSLARMPSLPESGFVMSGIRRKS

>CYP152A1_ortholog(BSUW23_01075)Bacillus subtilis subsp. spizizenii W23

MNEQIPHDKSLDNSVALMREGYLFIKNRKEHYHSDLFQARLLGKTFICMSGAEAAKIFYDTERFQRQGALPKRVQKSLFGVNAIQTMDGDAHIHRKLLFLSLMTPPHQKRLAELMTEEWEAAVTRWEKADQVVLFEEAKEILCRVACYWAGVPLKETEVKERADDFIDMVDAFGAVGPRHWKGRRARPRAEEWIERMIEDVRAGLLKIPAGTALHEMAFHTQEDGNQLDSRMAAIELINVLRPIVAISYFLVFSALALHKHPKYKEWLRSGSSREREMFVQEVRRYYPFGPFLGALVKKDFEWNNCEFKKGTSVLLDVYGTNHDPRLWDNPDEFRPERFAEWEENPFDLIPQGGGHAEKGHRCPGEGITIEVMKASLDFLVNQIEYEVPEQSLHYSLARMPSLPESGFVMSGIRRKR

>CYP152A2(CEA_G3333)Clostridium acetobutylicum EA 2018

MLLKENTAKDKGIDSTLDLLKEGYLFIKNRADHYQSDLFETRLMGQRIICMTGEEAARIFYDSDKFKRQGAAPKRVQETLLGENAIQTLDGESHLHRKKLFMLLTNQVQQKRLAELTTEKWEASASKWHTKSIVLFNEANEILCQVACHWAGVPLMESDIKNRAEDFSSMIDSFGAVGPRHWKGKKARNTIEAWIKEIIENVRSGRIRAEEGSPLHEIAFYIDVNGQQMPAEMAAIELINILRPIVAISTFITFSALALYEHSEYREKLQSKDIRYLEMFTQEVRRYYPFAPFVGARVRKDFLWNNCEFKKEMLVLLDIYGTNHDSRIWQKPYEFIPDRFRSYKGNLFDFIPQGGGDPSSTHRCPGEGITLEIMKTSLDFLSTKIDFTVPDQDLSYSLSKIPTLPKSGFIIDNINLKL

>CYP152A3(Clole_1959)Cellulosilyticum lentocellum

MSMNKQVPHDKCIDNTLALLQEGYLFIQNRVNRYQCHLFETRLLGKKVICMSGKEASKLFYNTDLFKRKGALPKRVQKTLFGVNAIQTMDDEGHIHRKMFFMSLLDQEYQKQLTEITKKRWQAAINNWESTQQIVLFDEVNNILSQSVCEWAGVPLQASEVKNRAKDFSTMVNTLASIGPMYWKGKRARTRIEKWIRGMIADTRSGKLNVEKSSVLYKLSFYEELDDKLLNTQMAAIELINILRPIVAISRYITFTALALYENEECIEKLRLGNENTREMFIQEVRRYYPFAPFLAAIVRKNFIWHGCKFKQGRLVLVDIYGTNHDPRIWVNPYEFRPERFKEKKNGLFDFIPQGGGDPSKGHRCPGEGVTIELMKVGLDFLINQIEFKIPAQDLSYSLTKIPALPASGFIMSNIRVKL

>CYP152A4_ortholog(OY17_08235)Bacillus sp. BH072

MDEQIPHEKGLDNSLALLRDGYVFVKNRAETYRSDVFRARLLGKTFICMSGAEAAKLFYDTERFQRQQALPKRVQKTLFGTGAIQAMDGERHKHRKLLFMSLMTPPRQKRLAEAVAKQWKASAEMWKGSNRIVLFDEAKKVLCRAACEWAGVPLKDSEVKERAEDFTDMVDAFGAVGPRHWKGRRARLKTEKWIEEVIEDVRSGKLQTPEGSALYEMAFHTELDGNRLDSHMAAVELINVLRPIAAISYFITFSALALHDYPEYRGKLRSRDDQEAERFAHEVRRYYPFAPFLGAVVKKDFVWKNCEFKKGASVMLDLYGTNHDSRLWEDPNEFRPERFQGREENKFDFIPQGGGDPANGHRCPGEGMTVEVMKTSLAFLANEIEYDVPSQDLSFSLSRMPALPESGFVISDVRRI

>CYP152A4_ortholog(BAPNAU_2689)Bacillus velezensis NAU-B3

MDEQIPHEKGLDNSLALLRDGYVFVKNRAETYRSDVFRARLLGKTFICMSGAEAAKLFYDTERFQRQQALPKRVQKTLFGTGAIQAMDGERHKHRKLLFMSLMTPPRQKRLAEAVAKQWKASAEMWKGSNRIVLFDEAKKVLCRAACEWAGVPLKDSEVKERAEDFTDMVDAFGAVGPRHWKGRRARLKTEKWIEEVIEDVRSGKLQTPEGSALYEMAFHTELDGNRLDSHMAAVELINVLRPIAAISYFITFSALALHDYPEYRGKLRSRDDQEAERFAHEVRRYYPFAPFLGAVVKKDFVWKNCEFKKGASVMLDLYGTNHDSRLWEDPNEFRPERFQGREENKFDFIPQGGGDPANGHRCPGEGMTVEVMKTSLAFLANEIEYDVPSQDLSFSLSRMPALPESGFVISDVRRI

>CYP152A4_ortholog(MUS_1146)Bacillus amyloliquefaciens Y2

MDEQIPHEKGLDNSLALLRDGYVFVKNRAETYRSDVFRARLLGKTFICMSGAEAAKLFYDTERFQRQQALPKRVQKTLFGTGAIQAMDGERHKHRKLLFMSLMTPPRQKRLAEAVAKQWKASAEMWKGSNRIVLFDEAKKVLCRAACEWAGVPLKDSEVKERAEDFTDMVDAFGAVGPRHWKGRRARLKTEKWIEEVIEDVRSGKLQTPEGSALYEMAFHTELDGNRLDSHMAAVELINVLRPIAAISYFITFSALALHDYPEYRGKLRSRDDQEAERFAHEVRRYYPFAPFLGAVVKKDFVWKNCEFKKGASVMLDLYGTNHDSRLWEDPNEFRPERFQGREENKFDFIPQGGGDPANGHRCPGEGMTVEVMKTSLAFLANEIEYDVPSQDLSFSLSRMPALPESGFVISDVRRI

>CYP152A4_ortholog(BANAU_1013)Bacillus velezensis YAU B9601-Y2

MDEQIPHEKGLDNSLALLRDGYVFVKNRAETYRSDVFRARLLGKTFICMSGAEAAKLFYDTERFQRQQALPKRVQKTLFGTGAIQAMDGERHKHRKLLFMSLMTPPRQKRLAEAVAKQWKASAEMWKGSNRIVLFDEAKKVLCRAACEWAGVPLKDSEVKERAEDFTDMVDAFGAVGPRHWKGRRARLKTEKWIEEVIEDVRSGKLQTPEGSALYEMAFHTELDGNRLDSHMAAVELINVLRPIAAISYFITFSALALHDYPEYRGKLRSRDDQEAERFAHEVRRYYPFAPFLGAVVKKDFVWKNCEFKKGASVMLDLYGTNHDSRLWEDPNEFRPERFQGREENKFDFIPQGGGDPANGHRCPGEGMTVEVMKTSLAFLANEIEYDVPSQDLSFSLSRMPALPESGFVISDVRRI

>CYP152A4_ortholog(RBAU_1067)Bacillus velezensis UCMB5033

MNEQIPHDKGLDNSLALLRDGYVFVKNRAENYRSDVFRARLLGKTFICMSGAEAAKLFYDTERFQREHALPKRVQKTLFGTGAIQSMDGERHKHRKLLFMSLMTPPRQKRLAEAVAKQWKASAEKWEGSNRIVLFDEAKKVLCRAACEWAGVPLKDSEVKERAEDFTDMVDAFGAVGPRHWKGRRARLKTEKWIEEVIEDVRSGKLQTPECSALYEMAFHTELDGNRLDSHMAAVELINVLRPIAAISYFITFSALALHDYPEYRGKLGSRDDQEAERFAHEVRRYYPFAPFLGAVVKKDFVWKNCEFKKGASVMLDLYGTNHDSRLWENPNEFRPERFQGREENKFDFIPQGGGDPANGHRCPGEGMTVEVMKTSLAFLANEIEYDVPSQDLSFSLSRMPALPESGFVISDVRRI

>CYP152A4_ortholog(AJ82_06225)Bacillus velezensis TrigoCor1448

MGEQIPHEKGLDNSLALLRDGYVFVKNRAENYRSDVFRARLLGKTFICMSGAEAAKLFYDTERFQREHALPKRVQKTLFGTGAIQSMDGERHKHRKLLFMSLLTPPRQKRLAEAVAKQWKASAEMWEGSNRIVLFDEAKKVLCRAACEWAGVPLKDSEVKERAEDFTDMVDAFGAVGPRHWKGRRARLKTEKWIEEVIEGVRSGKLQTPECSALYEMAFHTELDGNRLDSHMAAVELINVLRPIAAISYFITFSALALHDYPEYRGKLRSRDDQEAERFAHEVRRYYPFAPFLGAVVKKDFVWKNCEFKKGASVMLDLYGTNHDSRLWENPNEFRPERFQGREENKFEFIPQGGGDPANGHRCPGEGMTVEVMKTSLAFLANEIEYDVPSQDLSFSLSRMPALPESGFVISDVRRI

>CYP152A4_ortholog(BAM5036_1007)Bacillus velezensis UCMB5036

MNEQIPHDKGLDNSLALLRDGYVFVKNRAENYRSDVFRARLLGKTFICMSGAEAAKLFYDTERFQREHALPKRVQKTLFGTGAIQAMDGERHKHRKLLFMSLMTPPRQKRLAEAVAKQWKASAEKWEGSNRIVLFDEAKKVLCRAACEWAGVPLKDSEVKERAEDFTDMVDAFGAVGPRHWKGRRARLKTEKWIEEVIEDVRSGKLQTPECSALYEMAFHTELDGNRLDSHMAAVELINVLRPIAAISYFITFSALALHDYPEYRCKLRSRDDQEAERFAHEVRRYYPFAPFLGAVVKKDFVWKNCEFKKGASVMLDLYGTNHDSRLWEDPNEFRPERFQGREENKFDFIPQGGGDPANGHRCPGEGMTVEVMKTSLAFLANEIEYGVPSQDLSFSLSRMPALPESGFVISDVRRI

>CYP152A4_ortholog(BASU_1046)Bacillus velezensis UCMB5113

MNEQIPHDKGLDNSLALLRDGYVFVKNRAENYRSDVFRARLLGKTFICMSGAEAAKLFYDTERFQREHALPKRVQKTLFGTGAIQSMDGERHKHRKLLFMSLMTPPRQKRLAEAVAKQWKASAEKWEGSNRIVLFDEAKKVLCRAACEWAGVPLKDSEVKERAEDFTDMVDAFGAVGPRHWKGRRARLKTEKWIEEVIEDVRSGKLQTPECSALYEMAFHTELDGNRLDSHMAAVELINVLRPIAAISYFITFSALALHDYPEYRGKLRSRDDQEAERFVHEVRRYYPFAPFLGAVVKKDFVWKNCEFKKGASVMLDLYGTNHDSRLWENPNEFRPERFQGREENKFDFIPQGGGDSANGHRCPGEGMTVEVMKTSLAFLANEIEYDVPSQDLSFSLSRMPALPESGFVISDVRRI

>CYP152A4_ortholog(BAMTA208_05145)Bacillus amyloliquefaciens TA208

MTEQIPHEKGLDNSLALLRDGYVFVKNRAENYRSDVFRARLLGKTFICMSGAEAAKLFYDTERFQRQQALPKRVQKTLFGTGAIQSMDGERHKHRKLLFMSLMTPPRQKRLAEAVAKQWKASAEKWEGADRIVLFDEAKKVLCRAACEWAGVPLKDSEVKERAEDFTDMVDAFGAVGPRHWKGRRARPKTEKWVEEVIEDVRSGKLQTPEGSALYEMAVHTELDGSRLDSHMAAVELINVLRPIAAISYFIAFSALALHDHPEYRDKLRSGDDQEAERFVHEVRRYYPFAPFLGAVVKKDFVWKNCEFKKGASVMLDLYGTNHDSRLWENPNEFRPERFQGREENKFDFIPQGGGDPADGHRCPGEGMTVEVMKASLAFLTNEIEYDVPPQDLSFSLSRMPALPESGFVISDVRRK

>CYP152A4_ortholog(LL3_01185)Bacillus amyloliquefaciens LL3

MTEQIPHEKGLDNSLALLRDGYVFVKNRAENYRSDVFRARLLGKTFICMSGAEAAKLFYDTERFQRQQALPKRVQKTLFGTGAIQSMDGERHKHRKLLFMSLMTPPRQKRLAEAVAKQWKASAEKWEGADRIVLFDEAKKVLCRAACEWAGVPLKDSEVKERAEDFTDMVDAFGAVGPRHWKGRRARPKTEKWVEEVIEDVRSGKLQTPEGSALYEMAVHTELDGSRLDSHMAAVELINVLRPIAAISYFIAFSALALHDHPEYRDKLRSGDDQEAERFVHEVRRYYPFAPFLGAVVKKDFVWKNCEFKKGASVMLDLYGTNHDSRLWENPNEFRPERFQGREENKFDFIPQGGGDPADGHRCPGEGMTVEVMKASLAFLTNEIEYDVPPQDLSFSLSRMPALPESGFVISDVRRK

>CYP152A4_ortholog(BAXH7_01079)Bacillus amyloliquefaciens XH7

MTEQIPHEKGLDNSLALLRDGYVFVKNRAENYRSDVFRARLLGKTFICMSGAEAAKLFYDTERFQRQQALPKRVQKTLFGTGAIQSMDGERHKHRKLLFMSLMTPPRQKRLAEAVAKQWKASAEKWEGADRIVLFDEAKKVLCRAACEWAGVPLKDSEVKERAEDFTDMVDAFGAVGPRHWKGRRARPKTEKWVEEVIEDVRSGKLQTPEGSALYEMAVHTELDGSRLDSHMAAVELINVLRPIAAISYFIAFSALALHDHPEYRDKLRSGDDQEAERFVHEVRRYYPFAPFLGAVVKKDFVWKNCEFKKGASVMLDLYGTNHDSRLWENPNEFRPERFQGREENKFDFIPQGGGDPADGHRCPGEGMTVEVMKASLAFLTNEIEYDVPPQDLSFSLSRMPALPESGFVISDVRRK

>CYP152A4_ortholog(BAMF_1180)Bacillus amyloliquefaciens DSM 7

MTEQIPHEKGLDNSLALLRDGYVFVKNRAENYRSDVFRARLLGKTFICMSGAEAAKLFYDTERFQRQQALPKRVQKTLFGTGAIQSMDGERHKHRKLLFMSLMTPPRQKRLAEAVAKQWKASAEKWEGADRIVLFDEAKKVLCRAACEWAGVPLKDSEVKERAEDFTDMVDAFGAVGPRHWKGRRARPKTEKWVEEVIEDVRSGKLQTPEGSALYEMAVHTELDGSRLDSHMAAVELINVLRPIAAISYFIAFSALALHDHPEYRDKLRSGDDQEAERFVHEVRRYYPFAPFLGAVVKKDFVWKNCEFKKGASVMLDLYGTNHDSRLWENPNEFRPERFQGREENKFDFIPQGGGDPADGHRCPGEGMTVEVMKASLAFLTNEIEYDVPPQDLSFSLSRMPALPESGFVIIGDRVFSIPVSCLPGGFFRQAGSFSD

>CYP152A5_ortholog(CLM_1194)Clostridium botulinum A2

MLIEEHIPKDKGIDNTFALLQEGYLFIRNRVDRYQSNLFETHLFGQKVICMTGEEAAKLFYNEELFQRNGAAPKRIQKTLFGENAIQTMDDEEHIHRKHLFMSLMTPLSQKILAELVMEKWKSSIDKWEHDKEIVLFNEAKETLCQISCKWAGVPLHKSEIKNRAEDFNLMVNALGAVGPQYWKGKMARSRAEKWISGIIQDVRSSRLDAEESTPLYAMAFHRDLDGNQMDTSMAAIELINVLRPIVAISTFITFAALGLYEHPECKEKLISGGDSYVEMFAQEVRRYYPFAPFLGARVRKDFILNECELKKGMLVLLDMYGTNHDSQIWEKPNEFYPERFKEWKGSLFDFIPQGGGDPVKTHRCPGEGITMEIMKGSLDFLVNKIEFQVPKQDLSYSLIKIPTLPKSGFIMTNIKRKF

>CYP152A5_ortholog(H04402_01113)Clostridium botulinum H04402 065

MLIEEHIPKDKGIDNTFALLQEGYLFIRNRVDRYQSNLFETHLFGQKVICMTGEETAKLFYNEELFQRNGAAPKRIQKTLFGENAIQTMDDEEHIHRKHLFMSLMTPLSQKILAELVMEKWKSSIDKWEHDKEIVLFNEAKETLCQISCKWAGVPLHKSEIKNRAEDFNLMVNALGAVGPQYWKGKMARSRAEKWISGIIQDVRSSRLDAEESTPLYAMAFHRDLDGNQMDTSMAAIELINVLRPIIAISTFITFAALGLYEHPECKEKLISGGDSYVEMFAQEVRRYYPFAPFLGARVRKDFILNECELKKGMLVLLDMYGTNHDSQIWEKPNEFYPERFKEWKGSLFDFIPQGGGDPVKTHRCPGEGITMEIMKGSLDFLVNKIEFQVPKQDLSYSLIKIPTLPKSGFIMTNIKRKF

>CYP152A5_ortholog(CLB_1078)Clostridium botulinum A ATCC 19397

MLIEEHIPKDKGIDNTFALLQERYLFIRNRVDRYQSNLFETHLFGQKVICMTGEEAAKLFYNEELFQRNGAAPKRVQKTLFGENAIQTMDDEEHIHRKHLFMSLMTPLSQKILAELVMEKWKSSIDKWEHDKEIVLFNEAKETLCQISCKWAGVPLHKSEIKNRAEDFNLMVNALGAVGPQYWKGKMARSRAEKWISGIIQDVRSSRLDAEESTPLYAMAFHRDLDGNQMDTSMAAIELINVLRPIVAISTFITFAALGLYEHPECKEKLISGGDSYVEMFAQEVRRYYPFAPFLGARVRKDFILNECELKKGMLVLLDMYGTNHDLQIWEKPNEFYPERFKEWKGSLFDFIPQGGGDPVKTHRCPGEGITMEIMKGSLDFLVNKIEFQVPKQDLSYSLIKIPTLPKSGFIMTNIKRKF

>CYP152A5_ortholog(CLD_3520)Clostridium botulinum B1 Okra

MLIEEHIPKDKGIDNTFALLQEGYLFIRNRVDRYQSNLFETHLFGQKVICMTGEEAAKLFYNEELFQRNGAAPKRIQKTLFGENAIQTMDDEEHIHRKHLFMSLMAPLSQKILAELVMEKWKSSIDKWEHDKEIVLFNEAKETLCQISCKWAGVPLHKSEIKNRAEDFNLMVNALGAVGPQYWKGKMARSRAEKWISGIIRDVRSSRLDAEESTPLYAMAFHRDLDGNQMDTSMAAIELINVLRPIVAISTFITFAALGLYEHPECKEKLITGGDSYVEMFAQEVRRYYPFAPFLGARVRKDFILNECELKKGMLVLLDMYGTNHDSQIWEKPNEFYPERFKEWKGSLFDFIPQGGGDPVKTHRCPGEGITMEIMKGSLDFLVNKIEFQVPKQDLSYSLIKIPTLPKSGFIMTNIKRKF

>CYP152A5_ortholog(CLI_1129)Clostridium botulinum F Langeland

MLIEEHIPKDKGIDNTFALLQEGYLFIRNRVDRYQSNLFETHLFGQKVICMTGEKAAKLFYNEKLFQRNGAAPKRIQKTLFGENAIQTMDDEEHIHRKHLFMSLMTPLSQKILAELVMEKWKSSIDKWEHDKEIVLFNEAKETLCQISCKWAGVPLHKSEIKNRAEDFNLMVNALGAVGPQYWKGKMARSRAEKWISGIIQDVRSSRLDAEESTPLYAMAFHRDLDGNQMDTSMAAIELINVLRPIVAISTFITFAALGLYEHPECKEKLITGGDSYVEMFAQEVRRYYPFAPFLGARVRKDFILNECELKKGMLVLLDMYGTNHDSQIWEKPNEFYPERFKEWKGSLFDFIPQGGGDPVKTHRCPGEGITMEIMKGSLDFLVNKIEFQVPKQDLSYSLIKIPTLPKSGFIMTNIKRKF

>CYP152A5_ortholog(CLC_1090)Clostridium botulinum A Hall

MLIEEHIPKDKGIDNTFALLQERYLFIRNRVDRYQSNLFETHLFGQKVICMTGEEAAKLFYNEELFQRNGAAPKRVQKTLFGENAIQTMDDEEHIHRKHLFMSLMTPLSQKILAELVMEKWKSSIDKWEHDKEIVLFNEAKETLCQISCKWAGVPLHKSEIKNRAEDFNLMVNALGAVGPQYWKGKMARSRAEKWISGIIQDVRSSRLDAEESTPLYAMAFHRDLDGNQMDTSMAAIELINVLRPIVAISTFITFAALGLYEHPECKEKLISGGDSYVEMFAQEVRRYYPFAPFLGARVRKDFILNECELKKGMLVLLDMYGTNHDLQIWEKPNEFYPERFKEWKGSLFDFIPQGGGDPVKTHRCPGEGITMEIMKGSLDFLVNKIEFQVPKQDLSYSLIKIPTLPKSGFIMTNIKRKF

>CYP152A5_ortholog(CBF_1100)Clostridium botulinum F 230613

MLIEEHIPKDKGIDNTFALLQEGYLFIRNRVDRYQSNLFETHLFGQKVICMTGEKAAKLFYNEKLFQRNGAAPKRIQKTLFGENAIQTMDDEEHIHRKHLFMSLMTPLSQKILAELVMEKWKSSIDKWEHDKEIVLFNEAKETLCQISCKWAGVPLHKSEIKNRAEDFNLMVNALGAVGPQYWKGKMARSRAEKWISGIIQDVRSSRLDAEESTPLYAMAFHRDLDGNQMDTSMAAIELINVLRPIVAISTFITFAALGLYEHPECKEKLITGGDSYVEMFAQEVRRYYPFAPFLGARVRKDFILNECELKKGMLVLLDMYGTNHDSQIWEKPNEFYPERFKEWKGSLFDFIPQGGGDPVKTHRCPGEGITMEIMKGSLDFLVNKIEFQVPKQDLSYSLIKIPTLPKSGFIMTNIKRKF

>CYP152A5_ortholog(CBO1038)Clostridium botulinum A ATCC 3502

MLIEEHIPKDKGIDNTFALLQERYLFIRNRVDRYQSNLFETHLFGQKVICMTGEEAAKLFYNEELFQRNGAAPKRVQKTLFGENAIQTMDDEEHIHRKHLFMSLMTPLSQKILAELVMEKWKSSIDKWEHDKEIVLFNEAKETLCQISCKWAGVPLHKSEIKNRAEDFNLMVNALGAVGPQYWKGKMARSRAEKWISGIIQDVRSSRLDAEESTPLYAMAFHRDLDGNQMDTSMAAIELINVLRPIVAISTFITFAALGLYEHPECKEKLISGGDSYVEMFAQEVRRYYPFAPFLGARVRKDFILNECELKKGMLVLLDMYGTNHDLQIWEKPNEFYPERFKEWKGSLFDFIPQGGGDPVKTHRCPGEGITMEIMKGSLDFLVNKIEFQVPKQDLSYSLIKIPTLPKSGFIMTNIKRKF

>CYP152A5_ortholog(CLK_0485)Clostridium botulinum A3 Loch Maree

MLLEEHIPKDKGIDNTFALLQEGYLFIRNRVDRYQSNLFETHLFGQKVICMTGEEAAKLFYNEELFQRNGAAPKRIQKTLFGENAIQTMDDEEHIHRKHLFMSLMTPLSQKILAELVMEKWKSSIDKWEHDKEIVLFNEAKETLCQISCKWAGVPLHKSEIKNRAEDFNLMVNALGAAGPQYWKGKMARSRAEKWISGIIQDVRSSRLDAEESTPLYAMAFHRDLDSNQMDTSMAAIELINVLRPIVAISTFITFAALGLYEHPECKEKLISGGDSYVEMFAQEVRRYYPFAPFLGARVRKDSILNECELKKGMLVLLDMYGTNHDSQIWEKPNEFYPERFEEWKGSLFDFIPQGGGDPVKTHRCPGEGITMEIMKGSLNFLVNKIEFQVPKQDLSYSLIKIPTLPKSGFIMTNIKRKF

>CYP152A5_ortholog(CLJ_B1087)Clostridium botulinum Ba4

MLIEEHIPKDKGIDNTFALLQEGYLFIRNRVDRYQSNLFETHLFGQKVICMTGEEAAKLFYNKELFQRNGAAPKRIQKTLFGENAIQTMDDEEHIHRKHLFVSLMTPPSQKILAELVIEKWKSSIDKWEHGKEIVLFNEAKETLCQISCKWAGVPLHKLEIKNRAEDFNLMVNALGAVGPQYWKGKMARARAEKWISGIIQDVRSGILEAEEDSALYAIAFHRDLDGNQMDTSMAAIELINVLRPIVAISTFITFAALALYEHPECKEKLISGGDSYVEMFAQEVRRYYPFAPFLGARVRKDFILNECELKKGMLVLLDMYGTNHDSQIWEKPNEFYPERFKEWKGSLFDFIPQGGGDPVKTHRCPGEGITMEIMKKSLDFLVNKIEFEVPKQDLSYSLIKIPTLPKSGFIMTNIKRKL

>CYP152A7(Cphy_2911)Lachnoclostridium phytofermentans

MIVKKQIPRDKCIDNTFTLLKEGYLFIQNRTERYSSDVFETRLLGKKAICISGKEAPKLFYNPILMKKKGALPKRIQKTLFGVNAIQTMDGRRHLHRKKLFMTIMNQEEQDRLSKITTEKWQEAISRWEGASRVVLYDEVNRILCQSVCEWAGVPLPASEVKCRAKDFSTMVNTFTAIGPEYWKGKKARKRTEKWIRGIIEATRSGKLRPGRNSALHQIAYYKDLDGKLLSTQMAAVELINVLRPVVAISTFITFTAVALYEHREYIKILRSSDENMREMFVQEVRRYYPFTPFLGAITRKNFMWKGYNFKKGTLVILDVYGINHDARIWENPYKFRPERFSEKREHLFDFIPQGGGDPSKGHRCPGEGITIELMKLSVDFLVNKLEFKIPEQELRYSLIKIPSLPKSGFIMRKIKTRTSM

>CYP152A9(BATR1942_19655)Bacillus atrophaeus 1942

MSMDKQVPHDKALDNSLAMMREGYLFIKNRVDRYQSDLFETRLLGKKAICMSGEEAAKIFYDPERMKRNGALPKRLLKTLFGVDAIQTMDGDAHTHRKLLFMLLMTPPHQKRLAELAMDQWLAALSKWEGAEKVELFEEAKNVLCKIACQWAGVPLEESEVKERADDFSAMVDAFGAVGPRHWKGRRARPRAEEWIRDIIENVRAGKTEAEKGTALYEMAFHTELDGSRLDTQMAAVELINVLRPIVAISTFITFSALALHNHPEYKEKLKSGNGDDLEMFVQEVRRFYPFGPFLGAQARKDFVWNQCEFKEGMLVLLDLYGTNHDARLWEAPNEFRPERFKDRKDQLFDLIPQGGGDPAKGHRCPGEGITIEVMKVTLDFLINHIEYEVPEQDLSYSLVRMPSLPESGFVMSRIRRK

>CYP152A9_ortholog(TD68_18675)Bacillus atrophaeus NRS 1221A

MDKQVPHDKALDNSLAMMREGYLFIKNRVDRYQSDLFETRLLGKKAICMSGEEAAKIFYDPERMKRNGALPKRLLKTLFGVDAIQTMDGDAHTHRKLLFMLLMTPPHQKRLAELAMDQWLAALSKWEGAEKVELFEEAKNVLCKIACQWAGVPLEESEVKERADDFSAMVDAFGAVGPRHWKGRRARPRAEEWIRDIIENVRAGKTEAEKGTALYEMAFHTELDGSRLDTQMAAVELINVLRPIVAISTFITFSALALHNHPEYKEKLKSGNGDDLEMFVQEVRRFYPFGPFLGAQARKDFVWNQCEFKEGMLVLLDLYGTNHDARLWEAPNEFRPERFKDRKDQLFDLIPQGGGDPAKGHRCPGEGITIEVMKVTLDFLINHIEYEVPEQDLSYSLVRMPSLPESGFVMSRIRRK

>CYP152A10(Clopa_0939)Clostridium pasteurianum BC1

MLMKERVPHDKSHTLALSQEGYLFIKNRIDQYQSNLFEAHLLGEKVICMGGEEASKVFYDPERFQRNGAAPKRIQKTLFGMNAIQTMDGDAHIHRKLLFMSLMTPTHQKRLAELTMEKWQASIDKWKSSEKIILFDEAKNILCWAACHWAGVPLSESEVKDRAEDFSAMVDAFGAVGPRHWKGRRARSRAEEWISGVIEDVRVGKLKSEEGSALYAIAFHRELHGGELDTQMAAVELINILRPIVAISTFITFTALALHKHSEYKDKLLTGHSNDLEMFVQEVRRYYPFTPFLGARVRKDFIWNQCEFKQGMLVLLDIYGTNHDSRVWENPYEFRPERFKNRKDNLFDFIPQGGGDPAKGHRCPGEGITVEIMKTSLDFLVNKIEFEVPHQDLSYDMARIPTLPKSGFVMNNIRRKF

>CYP152A11(N288_02540)Bacillus infantis

MAMNGQIPHDKSLDNSLTLMKEGYEFIPNRKERYGTELFEARLLGQKVICMSGQEAARLFYDTDRFKRNGAAPKRIQKTLFGENAIQTMDGDAHIHRKLLFMSLMTPPHQKRLAGMVMNEWKAAAAKWQEDGREVVLMEEAKVILCRTACAWAGVPLKQEDEKERAEEFADMVDAFGAVGPRHWKGRHARGSSEDWIRGLIEDVRDGKLAAEEGTALYEMAFHKNLDGTHLDSQMAAVELINVLRPIVAISTFIAFSALALNGHPRYKEMLQTRSGSELEMFVQEVRRFYPFGPFLGAIVRKDFIWNEAEFKEGMLVLLDIYGTNHDPLLWEEPYEFRPERFENWDGSLFDLIPQGGGDASKGHRCPGEGITIEVMKASLDFLVNEIDYSVPENQDLSFSLSRMPTFPESGFIMSNIKKK

>CYP152A12(Closa_1866) Clostridium saccharolyticum WM1

MHDKGIDNTLKLLEEGYLFIKNRMDLYNCNIFETHIMGEKAICITGEEACKIFYDEELFQRKGAMPKRVQKTLFGVNAIQGMDDKAHIQRKLFFMSLMTEVHQKELAELFDKEMEASAYKWMNMNEIVLFHEAKNIICKVACFWTGVPLPESDVEKRADDFCSMVDGIGGIGPRYWKGKAARNRTEEWIRGIIEDVRQGKLIVKDDSVLNLMAIHREPDGNQMELPMAAKELINVIRPIVAISTYITFTALAIYEHPECYEELMRGDNNYFEMFVQEVRRYYPFTPFLGARVHKDFVWNQYEFKKGELVLLDVYGMNHDPNIWSNPDEFRPERFKEQKDRKFSFIPQGGGDPGKGHRCPGEGITIEIMKASLNFLVNKIDFEIPEQDASYSLSRIPTLPKSGLIMKNIRKKY

>CYP152A13(Bcoa_2050)Bacillus coagulans 36D1

MAGQIPNEKTIDDTIDLMKEGYLYIKNRTDDYHANLFETRLLGQKAVCISGEEAVKMFYDPEKFKRNGAVPKRVQKTLFGENAIQTLDGKEHLHRKALFLSLMGPDAQQHLAQMVRAAWEARIPEWEQKDRIVLFDEAKLLLCRCACKWAGVPLREEEAEKRAQEFIDMVYAFGAVGPRHWKGRTARNSTEDWIRTVIEDVRAGKIEAKDGSPLKEMAFYEDAQGRRFNAQMAAVELINVIRPVTAIATFITFAALALFEHPRARDWVQNGDKQHIDWFTREVRRFYPFGPFLGAKVRKSFTWNGYPFDEGLLVLLDMYGTNRDPAIWGDPDVFSPERFRDWDGSLYNFIPQGGADPAKGHRCPGEGITQEIIEASLDFLVNAIDYDVPAQDLSVPLNKMPTLPESGFILQHVKKKTPAS

>CYP152A14(BpOF4_07440)Bacillus pseudofirmus

MKTSQANIPSEKGLDHSLSLLKDGYLFIHKRAQDFQSDIFKTRLMGEEAICLRGEDGAKVFYDNDKFKREGAAPKRVQKTLFGEDAIQSMDGEAHRHRKQLFMSLMSRERLQELNEITRAQWLLYIKKWETQKKIVLFDEMEAMLTKVACEWAGVPLEEKEVEKRTKDFGDMIDAFGAVGVRHARGRSARKRTEAWITSLIEEVRSEQRSPRESTALYQMAWHKELDGTLMDAKMAAIELINVIRPTVAVGRFITFGAVGLHEFPKEREKLMQDQDGTYSHLFTQEVRRYYPFAPFTGARVKKDFTWNSHQFKENTLVLLDIYGTNRHPKLWESPNEFRPERFKGWEGSPFSFIPQGGGDEHKGHRCAGEWITIELMKISLSQLAQNITYDVPKQDLTYDLSRMPSIPKSRFIIQNVRRQQQ

>CYP152A15(OXB_3532)Bacillus sp. OxB-1

MQRPIERGLDHSLNLLTEGYSFIRNRTESFGAPLFETRLMGKKMICMTGKEAVQLFYDENRFQRHGAIPKRIQKSLFGVGGVQTMDGELHAVRKQLFLSLMTEEKLAELRSSTSAQWRLWAEAQNGREVVLFDETEKILCRVACLWAGVPLKEADVGGRAYDFGAMVDAFGGVGPRYQEGKNARKRAEQWIEQIIEKYRSGAIESEEGKAIQAVATHRDADGRLLDTHSAAVELINVLRPVVAVARFIIFGALALHDFPEYKDKLKECEEEFLEMFAQEVRRFYPFAPMLGAKTRYDFKWNGYRFAKGETVLLDLYGTNHDPELWENPDTFLPERFADRKEDLYDFVPQGGGDPNSGHRCPGEKATVEIMKASFRFLVEELDYEVIDDQDLTISHVRMPTLPKSRFIIRVK

>CYP152A16(HBHAL_1734)Halobacillus halophilus

MTVKVPKDRTIDNTLAIFKDGYEFISKRVQNNHLDVYETRVLGEKVALLSGEEGAKLFYDTNRMNRKNALPGRVLKTLFGEDAIQTMDGKAHTHRKLLFMSLMTPEALASLKVITNKHWDQFANKWEGKKKVVLYDETRELLCRTACEWAGVPLKEKEVEKRTRDLSNMIDGFSAVGPKHIESRRARKRTEKWIEDLILDVREGKQQAQKGTAIYEMAMHKDLHGNPLDARMAAIELINVIRPLVAISKFITFGALAFYQFPRTIAKAANEDDYLLRFTQEVRRYYPFVPFLGARVDHDFTWQQYDFKEGQLVMIDIYGTNHDPRLWENPDEFIPDRFLNWNGGLYDLIPQGGGDYYKGHRCPGEQPTIEILKTSFRYMTNHLDYEVPKQDYSYSLRKMPALPKSGFIIKNVHRQ

>CYP152A17(AAV35_010405)Salimicrobium jeotgali

MSYFPAKDKGPDRSLGILKDGYNFARKRLQKQHLDVYETKLLGERAALLGGEKGAELFYDNDKMKRGGAMPAFILKTLFGENGIQTMDGNPFEHRKRLFLSLMTPEALDRLFQITVEHWERASEVWEKKRKVVLYNEMLTLLMKVSCEWAGVPLKDKEVKKRADDFALMIDGFGSIGPKHLQSRKARQRSEQWFENLILDVREGKLEAQEGTAIFEMAMHRDLNGKHLDARMAAIELLNIVRPTVAVSKFITFGALAMEEFPGEKEKVAGSEDYLYYFAQEVRRFYPFVPYLPARTKKEFAWQGYNFSEGQLVLIDVYGNNHDPRIWEEPETFNPERFRNWNGGLFDLIPQGGGDYEKNHRCPGEWSTLEILKASFQYMAQHLEYKLPKQDLSYSLNKMPALPKDGFIMKSVKKK

>CYP152K2(SSIL_0949)Solibacillus silvestris StLB046

MTNSVPKEEGIDHSLNLLREGYLYILNRRQSFHSDLFETRLLGKKAVCMGGKEAADLFYDNSKFKRAGVAPNRVAETLFGKKGVQTLDGDAHKHRKKMFMSIMSQDRLKKMNEIAAKQWDTALTKWQQMDDVVLYEEALEIMCRSACEWAGVPVEEKDMKKLADDFRAMFESATAIGPSHWAGRHARNRVEKWMGNLIDQVREGKLNPEEGTALYTFSWHRDLEGNLLDPEVASVEVINILRPIVAIAVYITFSALAAHHYPEERKKLQSGNEKNTQMFVQEVRRFYPFFPFAAAEVKADFTWKDYTFEKGTLTLLDLYGTNHDTNIWGNPEVFQPSRFENWDGSPFSFIPQGGGDYYLGHRCAGEWVTIEMMKVSLDFLVNQMTYDVPEQDLSYSKVSIPSLPKSKVMINNVQRVLR

>CYP152K3(NY10_1909)Carnobacterium sp. CP1

MDEQSMPHEKGLDNTVHVLKEGYRYILNRRKSFQSDVFETRLLGQKAFCIGGEAAAELFYDTTKFKRAGAAPKRVQKTLFGETGVQTLDGEEHLHRKEMFMSLMSPEKLDKMDKILKKQWEATAEKWAEQDEIVLYHEAQEVLFRTACEWAGVPFEEREVAELAADLGKMIETPAEIGPAHWIGRHDRNQTEKWMENLIEQVRDKKLSPPEGTALAVFSWHQDLEGNLLDANTAAVEVLNIIRPIVAIAIYINFTALAVHEYPAAKEKLAAGDPKQLQAFVQEVRRFYPFFPFQGAIVKKDFTWQGYEFEEGTLTILDIYGTNHDPVLWKNPEVFDPERFMDWKESPFSFIPQGGGDFLGGHRCAGEWLTIKVMNTCLDYLANRMEYEVPQQDLSYSLDKMPSIPHSEMILKNIRMKN

>CYP152K4(X953_02135)Virgibacillus sp. SK37

MVEQKEIPKESGIEHSIGLLREGYMYIPNRRHSFQSDLFETRLLGQKAICMGGKEAAEIFYDNTKFKRSGAAPKRVQKTLFGENGVQTLDGEEHKHRKQMFMSLMSSERLEHLNNITYKHLDTVATDWEKKEQIILYEEAKRLMTRVACEWAGVPLWANEVDKRADDLSTIIESSVSMGINYRKGKQARNRSENWMKDLIAQVRNEERMPEEGTALYTIAWHRDLQGDLLEPDVAAVELLNILRPIVAIAIYISFTTLAVQQFPKQKNKLRENGSDYYQMFVQEVRRYYPFFPFAGARVKENFTWEGYSFKKGTLTLLDLYGTNHDHRLWDNPDRFDPERFKNWEGSPFDFIPQGGGDHYLGHRCAGEWVTVRVMKVVLDYFVNRMDYQVPTQDLSFSMVSMPSIPKSGIKLTHMKKV

>CYP152K5(BG04_5610)Bacillus megaterium NBRC 15308 = ATCC 14581

MCRTACQWAGVPIQEGKVRELTKDLGAMFESPAAIGPNHWLGRNTRNRVGKWIGELIDEVRNGNMNVPTNIILYRFTQYRDLEGNLLDTNTAAVEVINILRPIVAIAIFINFIMLSLHHFPEEKGKLKSRDEEYAQIFIQEVRRFYPFFPFVTALVKKDFTWNNYKFEEGTLTLLDLYGTNHDPKIWNNPDIFNPDRFTKWEGSPFSFIPQGGGDYFMGHRCAGEWVTLEVMKVSLDYLVNRIEYEIPDQDLNFSMVDIPSIPHSKIVIKNVVTAKVTWLFLTFGEGAL

>CYP152L3(AAT16_05610)Salinicoccus halodurans

MGTIKKDKGLDNTLKVMKQGYLYTANQRQRLGADNIFETRALGGKRVVVINGKEAAELFYDNDKTERSGTLPKRLVNTLFGKGAIHTTTGKKHIDRKALFMSLMTENNLQHLRELTRNHWYMNTHKMEQMDQINIYRESIVQLTKIGTKWAGVQAPEEKIEDIATDMDIMIDSFKGLGSAFKGYKESVNARKRVEDWLEEQIIETRKGKIFPPEGTALYEFAHWEDYQGNPMDSRLCAIDLMNTFRPLIAINRFVSFGLLAMHEHPISKEKIKTDDDYAYMFSQEVRRFYPFVPFLPGKAKTDIEFKGHKIEKDTMMVIDIYGTMHDEKVFENPDEFYPERFKEWDGSPFDLIPQGGGDYYTNHRCAGEWMTIIIMEETMKYFAERITYDVPEQDLTVDLNSIPGYVNSGFIIENVQENIDRK

>CYP152L4(SPSINT_0926)Staphylococcus pseudintermedius HKU10-03

MAKKLPKDTGLDNTLKMINEAYTYVPKRLEKFGTKAFETRALGMKPIVVISGKAAAELFYDNDKISRKGTLPKRIVHTLFGKGAIHTTEGKVHVDRKALFMSLMTEENLKYLRELTRNYWFMHTERMQNKDEVNVYQEAGLILTKVGFRWAGLKQTDEQAAQNAEDMNTMIDSFSGLGQSLKGYREAKKARARVEQFLQEQIEAVRVGQQYAEPGTALYEFAHWKDLNDQPMDPHLCAVDLMNIVRPLVAVNRFVSYGVKALIEFDQERKKLQVTNDPNYAYKFAQEVRRIFPFVPFLPGRLKKTVEFDGFKLKKGTLTVLDIFGTTHDPELFENPYQFNPDRFDNWDGSPFDLIPQGGGDFYTNHRCAGEWMTVIVMEETIQYFANKIDFVVPAQDLSVKLSQFPGKVTSGTMIKNVYPRI

>CYP152L5(EP23_02095)Staphylococcus agnetis

MAKQLPKDPGLDNTFKVLKEAYTYVPCRLEKFNSKAFQTTGMGMKPIAVINGKEAAELFYNNDVMQREKTLPKRVVNTLFGKGAIHTTKGKVHVDRKALFMSLMTEENLKYLRELTRNHWFMHTEHMQNQKEVNIYKESIYVLTKIGFRWAGIHQTAEEAEQNAQDMDIMIDSFKGLGQTIGGGYRKAKKARARVEQFLEKQIIAVRKGKINAEPGTALYEFAHWEDYKGNPMDARLCAIDLMNVVRPLAAVNRFVSYAVKAMIEYDQERIKLQVSDDPNYAYKFAQEVRRIFPFVPFLPGKLKKNIEFDGYRIKKGTFTLLDVFGTTHDPELFENPYQFNPDRFENWDGSPFDLIPQGGGDFYTNHRCAGEWMTIIVMEETIKYFANKIDFEAPSQDLFVKLDQFPGKVTSGTIIQNVRPRVQR

>CYP152L6(SHYC_08325)Staphylococcus hyicus

MAKKLPKDSGLDNTFKVLKEAYTYVPCRLEKFNTKAFQTKAMGMKPIVVISGKEAAELFYNNDVMQREKTLPKRVVNTLFGKGAIHTTKGKVHVDRKALFMSLMTEENLKYLRELARNHWFVHTEHMQTQDEVNIYKESIYVLTKIGFRWAGIRQTAEEAERNAKDMDVMIDSFKGLGQTLGSGYRKAKKARARVEQFLEDQIVAVRNGEIKAEPGTALYEFAHWEDYKGNQMDARLCAVDLMNVVRPLAAVNRFVSYAVKAMIEYDQERLKLQVTDDPNYAYKFAQEVRRIFPFVPFLPGKFKKTVEFDGYRIKKGTFTLLDVFGTTHDPDLFENPYQFNPDRFDNWDGSPFDLIPQGGGDFYTNHRCAGEWMTIIVMEETIKYFANKIDFDAPAQDLSVKLDQFPGKVTSGTLIKNVRPRLQ

>CYP152M1_ortholog(EFAU085_02579)Enterococcus faecium Aus0085

MKEVPVVDIKITDLKKLYQKGYNMLEELRHEADAPVVKAKIFNKEAITIYGSSAAKVFYDPRNFKRKGAMPKLVLKTLFGQGGVQTLDGAAHHHRKNIFMDLMTPERMEDYHRILDKNLTQALEAQHGQFELFDLSKMVFFTSICEWAGINLSAISKDEVEKLAEYQISMISGTFTSPIDHIKGVENRKKSEKWAQGLIEEARQNPVAGKENVALYAFANATDLDGQLLPLEVAAVELLNIIRPTVALTVWAALMGHALFSRPDLYQQLKNDFSTLQDPFIQEMRRYYPFFPMLPAISLKEVEVDGYRIPEGSWVILDLYGTDHDERTVEAPDSFMIKRYVGKAKDISYKEEYEMIAQGGGNFRQMHRCAGEWITLHSLRVFSDQLVNKFEFSVPEQDWTIPFNQFPTYPNSRALLYKN

>CYP152M1_ortholog(EFAU004_02498)Enterococcus faecium Aus0004

MKEVPVVDIKITDLKKLYQKGYNMLEELRHEADAPVVKAKIFNKEAITIYGSSAAKVFYDPRNFKRKGAMPKLVLKTLFGQGGVQTLDGAAHHHRKNIFMDLMTPERMEDYHRILDKNLTQALEAQHGQFELFDLSKMVFFTSICEWAGINLSAISKDEVEKLAEYQISMISGTFTSPIDHIKGVENRKKSEKWAQGLIEEARQNPVAGKENVALYAFANATDLDGQLLPLEVAAVELLNIIRPTVALTVWAALMGHALFSRPDLYQQLKNDFSTLQDPFIQEMRRYYPFFPMLPAISLKEVEVDGYRIPEGSWVILDLYGTDHDERTVEAPDSFMIKRYVGKAKDISYKEEYEMIAQGGGNFRQMHRCAGEWITLHSLRVFSDQLVNKFEFSVPEQDWTIPFNQFPTYPNSRALLYKN

>CYP152M1_ortholog(M7W_2456)Enterococcus faecium ATCC 8459 = NRRL B-2354

MKEVPVVDIKITDLKKLYQKGYNMLEELRHEADAPVVKAKIFNKEAITIYGSSAAKVFYDPRNFKRKGAMPKLVLKTLFGQGGVQTLDGAAHHHRKNIFMDLMTPERMEDYHRILDKNLTQALEAQHGQFELFDLSKMVFFTSICEWAGINLSAISKDEVEKLAEYQISMISGTFTSPIDHIKGVENRKKSEKWAQGLIEEARQNPVAGKENVALYAFANATDLDGQLLPLEVAAVELLNIIRPTVALTVWAALMGHALFSRPDLYQQLKNDFSTLQDPFIQEMRRYYPFFPMLPAISLKEVEVDGYRIPEGSWVILDLYGTDHDERTVEAPDSFMIKRYVGKAKDISYKEEYEMIAQGGGNFRQMHRCAGEWITLHSLRVFSDQLVNKFEFSVPEQDWTIPFNQFPTYPNSRALLYKN

>CYP152M1_ortholog(HMPREF0351_12437)Enterococcus faecium DO

MKEVPVVDIKITDLKKLYQKGYNMLEELRHEADAPVVKAKIFNKEAITIYGSSAAKVFYDPRNFKRKGAMPKLVLKTLFGQGGVQTLDGAAHHHRKNIFMDLMTPERMEDYHRILDKNLTQALEAQHGQFELFDLSKMVFFTSICEWAGINLSAISKDEVEKLAEYQISMISGTFTSPIDHIKGVENRKKSEKWAQGLIEEARQNPVAGKENVALYAFANATDLDGQLLPLEVAAVELLNIIRPTVALTVWAALMGHALFSRPDLYQQLKNDFSTLQDPFIQEMRRYYPFFPMLPAISLKEVEVDGYRIPEGSWVILDLYGTDHDERTVEAPDSFMIKRYVGKAKDISYKEEYEMIAQGGGNFRQMHRCAGEWITLHSLRVFSDQLVNKFEFSVPEQDWTIPFNQFPTYPNSRALLYKN

>CYP152M1_ortholog(M395_11575)Enterococcus faecium T110

MKEVPVVDVKITDLKRLYQKGYNMLEELRHEADAPVVKAKIFNKEAIVIYGSSAAKIFYDPRNFKRKGAMPKLVLKTLFGQGGVQTLDGAAHHHRKNIFMDLMTPERMEDYHRILDKNLTQALETQHGQFELFDLSKMVFFTSICEWAGINLSLLSKDEVEKLAEYQISMISGTFTSPIDHIKGVENRKKSEKWAQSLIEEARQNPVAGKENVALYAFANATDLDGQLLPLEVAAVELLNIIRPTVALTVWAALMGHALFSRPDLFQQLKNDFSTLQDPFIQEMRRYYPFFPMLPAISLKEVEVDGYRIPEDSWVILDLYGTDHDERTVEAPDSFMIKRYLGKAKDISYKEEYEMIAQGGGDFRQMHRCAGEWITLHSLRVFSDQLVNKFEFSVPEQDWTIPFNQFPTYPNSRALLYKN

>CYP152M2(EHR_06745)Enterococcus hirae

MKEVPVVDIKITEIKKRYEQGYHLLEELRKEANAPVVKAKIFNKEAIVIYGKEAAQIFYDPRNFKRKGAMPKLVLKTLFGKDGVQTLDGAQHHHRKSIFMDLMTPERMDTYHQILEKNLSQALNEQHGQFELFDLSKQVLFTSICEWSGINLAELTDEEINQFADYQISMISGTFTSPIDHIKGVENRKKSEQWAQRLIEDARANPVPGKEDVALYAFAQATDLDGNLLPIEVAAVELLNIIRPTVALTVWAALMGHALFSRPDLKQQLKNDFTEFQDPFIQEMRRYYPFFPMLPAIALKDVSVDGYLIPKDSWVILDLYGTNHDERTIDAPDSFMIKRYLGKAKDLSYEEEYEMIAQGGGDFRQMHRCAGEWITLHSLRVFSDQLVNHYEFSVPEQDWTIPSNQFPTYPNSRVLLYKE

>CYP152M3(EMQU_2599)Enterococcus mundtii

MQEIPVVDIKMTEIKKYYNEGYKMLGKLREEAQSPVVKAKIFNKEAVIIYGEEAAKHFYDPRNFKREGAMPKMVQKTLFGKDGVQSLDGAEHHHRKAIFMDLMTPERMEDYHQILDRVLAHELEQQHGTFELFDLSKKVLFTAICEWSGINLADYSEEEINQLADYQISMISGTVTSPLDHFKGVENRKKSEKWAQELLEEARENPVAGKENVALYAFANATDLKGNLLPLDIASVELLNIIRPTVALTVWAALMGHALFSRPDLYQQLKNDFKELQDPFIQEMRRYYPFFPMLPAFALQDVEVDGYRIPKDSWVILDLYGTNHDERTVDSPEAFLIKRYIGKAKKISYEEEYEMIAQGGGDFRKMHRCAGEWITLHSLRVFSDQLVNRFAFSVPEQDWTIPFNQFPTYPNSRGLLYKE

>CYP152M4(FOL01_1121)Weissella jogaejeotgali

MKQVPETKIKLTDVKELINKGYNLLGELREEADAPVAKAEFLTEEITTVYGEEAARKFYNPEDFKREGSMPKAVLKTLFGEDGVQTIDGKKHHQRKNYFMDLMTPERMEDYRAILDQNLATELDQQHGTFELFDLSKRVLFNSICEWAGINLAQYDPKEIDKLASNQISMISGAITSPTDHLKGVKDRNESEGWAQSLIKEARKNPVPGKENLALYTFAQAEDLEGELLPVEVAAVELLNIIRPTVALTVWMALMGHALFSKTNLYDQLKEDFDTLQDSFIQEMRRYYPFFPMLPAIALRDVEIDGYEIPKDSWVVLDIYGTNHDARTIDHPEKFDIKRYIGKTKEISYEEEYEMIAQGGGEFRNMHRCAGEWITLHSMRVFSDQLVNKYNFSIPEQDWTVPMNQFPTYPNSKALLFKE

>CYP152M5(SporoP37_04975)Sporosarcina sp. P37

MKQIPETKVTLTDVKELIDKGYNLLGELREEVDAPVVKATFLTKEITAIYGEEASRKFYNPENFKRAGAMPKPVLKTLFGEGGVQTLDGEEHHHRKNYFMDLMSPERMEAYHEILSEKLSKELTKQHGEFELFDLANKVLFTSISEWSGINLESFDSKTLDKLARNQISMISGAVTSPVDHIKGITDRKESEKWAQDLIKEARKHPVPGKEHLALYAFANATDLDGEVLPVEVAAVELLNIIRPTVALTVWVALMGHALFARRDIYEELKADFDGLQDSFIQEMRRYYPFFPMLPAIAVSDVEIDGYLIPKDSWVVLDIYGSNHDERTIASPEQFDVRRYAGRAKDISYEEEYEMIAQGGGKFREMHRCAGEWITLHSLRVFSDHLVNKYEFAVPEQDWTIPMNQFPTYPNSKVLLFKE

>CYP152M6(TEH_14060)Tetragenococcus halophilus

MSFKEIPETKVKLKEVKDLYNAGYEVLSELRNEVNAPVVKASLLNQEIIAVYGQNAAKKFYDADNFKRDSAMPTPILKTLQGEGGVQTLDGQQHYKRKSIFMDLMTPDRMEDYRKILEETLKETLDAQTGKFELYHLTKNVLFKAICKWAGVNLGNYTTEEIDELADTQVNMFEGTVNSVSDHLQGLEGRKKAEKWAQELLADARENPVPGKENVALYAFADARGVDGELLPLNIAAVDFLNIIRPTVAITVWVNLMGHALFGPNNVYKELKQNFDHLQDSFIQELRRYYPFFPMVPAISTRDVDIDGYLVPEGSWVVLDLYGTNHDGRSIDNPDSFVIDRYVGRTKEISYDEEYEMIAQGGGDFRAMHRCAGEWITLHTLRVFSDNLVNHYEFSVPEQDWTVPMNKFPTFPEDKVLLFKNE

>CYP152N1(EAT1b_2299)Exiguobacterium sp. AT1b

MGKVIPKQEGLDHSVDFLREGYLFVANRRKSFQSNIFESRLLGERVICLGGEEAAEVFYDANKFTRQDAAPKRLLKTLFGEGGVQTLDGSEHTHRKQMFMSLMTKENIDRLLRLTYREWNQIERMGEEIVLYDIAQEVLMKAVCEWSGVPLAKEEVGKRTEEMRLLFESGTSLGPTYLQGRKARSSAEVWIRQMVKEVRSNRLLPNEHTALYEFSWHRDESGELLPEEVVAVEVLNILRPTVAISVYVLFTVLALHQFPDVKEQVERGEVSKTEFVQEVRRFYPFFPVAAARVKTDFEWDGYAFPEGTLTLLDLYGTNHDVSIWTEPDRFDPSRFKDWKESPFNFIPQGGGDVDFGHRCAGEHVTIAILAQVIELFTKEYAYTVPPQDLSYSFVDMPSLPKSKLRLTHLTRNQ

>CYP152AC1(AAT16_12110)Salinicoccus halodurans

MSGEIPKDEGLDKTLSVLKEGYEFILNRKEDLNTDVFETRILGEKTICLTGSEGAELFYDNDRFRRSDAAPRRVEKTLFGEGGVQGLDGAEHHHRKAMFMTLMDDDSMMEIDRLMRKYWRRYFEAAGSLETIELYEAAKIVILRTACEWTGVPLREEEIEERARQISDLFESPAALGVQHWKGRISRSKANEWMEEVIEDVRKEKISVDEDRAVFAFSRARDEKGELLDKKTAAVEMLNLIRPMTAVSVWVAMIGLAMHEFPREKEKLKRTEHYKIEWFIQEVRRYYPFFPFAVARTDADFKWKGYEFKEGTLTLLDLYGTNRHPADWIRPEEFMPERFRGWQQTPFNFIPQGGGSYDFGHRCAGEFITIVMMKATVDFLVNEMDYDVPEQDFGFEFNDIPAVPNDKVKINNYRFQ

>CYP197A1(BH0579)Bacillus halodurans

MPTNTMPTGPKGNPVLGNTIEFGKDPLQFITRCSQEYGEIVRLRFERERDTFLLNDPKHIQYVFMNKGGEFSKGYQQDPIMGLVFGNGLLTSEGSFWLRQRRLSQPAFHPKRIADYADTMVGYCERMLNTWMDNDTRDINDEMMQLTMAIATKTLFDLDLHKGDTQEASRSLDTVMTAFNEQMTNVFRHVLHLIGLGKLVPPVSRELREAVESLDKMIYSIIEERRKHPGDRGDLLSMLISTYDEDDGSYMTDRQLRDEIITLFLAGHETTANTLSWAFYLLSQHPHVEEKLYQEVSQVLGNRPATLEDMPKLSYAEHVIKETLRVQPTVWLISRRAEKDVTLGDYHISAGSEIMISQWGMHRNPRYFNDPLTFLPERWDNNDNKPSKYVYFPFGGGPRVCIGERFALMEATLIMATIVREFRMELVDELPIKMEPSITLRPKHGVTMKLRKR

>CYP197S1(B2K_23970)Paenibacillus mucilaginosus K02

MTKTQAGVPGPRPVPLLGNLLSMGAEPHVFFAKCAEQYGPVVRIKLDPRRDTFLITRPQDIQHVLNQTQRYFAKGYHRDPILSRVLGNGLVTSEGSFWLRQRRLSQPAFHHHRIRSYADIMTAYAQRMLAAWEHEESRDIHADMMQCTMEIVAKTLFDVDLHAGDGRSNPVGEALDAVFHEYVKQYTSVMRRLLDLLPVSVPVPGDKKLQESVEQLNRIILDIIDRRQAEGTEDRGDLLSMLLLARDEDGTGMTREQLRDEIMTLFLAGHETTANVLSWTLYLLAREPEAEGKLLEELDRVLGGQPPAFEHIPLLTYTQSVVKESMRLYPPVWLISREPIEDVEIGGYTLPAGCEISVCQWVMHRLPEYFEEPERFQPERWTPEFEKSLPAGVYIPFGAGPRVCIGNQFAMMEAVLLLASIGQRFRLTLEPGHKVLLEPSITLRPQNGIRVRVWKRSAEQEKAD

>CYP197S1_ortholog(PM3016_4624)Paenibacillus mucilaginosus 3016

MTKTQAGVPGPRPVPLLGNLLSIGAEPHVFFAKCAEQYGPVVRIKLDPRRDTFLITRPQDIQHVLNQTQRYFAKGYHRDPILSRVLGNGLVTSEGSFWLRQRRLSQPAFHHHRIRSYADIMTAYAQRMLAAWEHEESRDIHADMMQCTMEIVAKTLFDVDLHAEDGRSNPVGEALDAVFHEYVKQYTSVMRRLLDLLPVSVPVPGDKKLQESVEQLNRIILDIIDRRQAEGTEDRGDLLSMLLLARDEDGTGMTREQLRDEIMTLFLAGHETTANVLSWTLYLLAREPEAEGKLLEELDRVLGGQPPAFEHIPLLTYTQSVVKESMRLYPPVWLISREPIEDVEIGGYTLPAGCEISVCQWVMHRLPEYFEEPEQFQPERWTPEFEKSLPAGVYIPFGAGPRVCIGNQFAMMEAVLLLASIGQRFRLTLEPGHKVLLEPSITLRPQNGIRVRVWKRSAEQEKAD

>CYP197S1_ortholog(KNP414_05230)Paenibacillus mucilaginosus KNP414

MTKTQAGVPGPRPVPLLGNLLSMGAEPHVFFAKCAEQYGPVVRIKIDPRRDTFLITRPQDIQHVLNQTQRYFAKGYHRDPILSRVLGNGLVTSEGSFWLRQRRLSQPAFHHHRIRSYADIMTAYAQRMLAAWEHEESRDIHADMMQCTMEIVAKTLFDVDLHAEDGRSNPVGEALDAVFHEYVKQYTSVMRRLLDLLPVSVPVPGDKKLQESVEQLNRIILDIIDRRQAEGTEDRGDLLSMLLLARDEDGTGMTREQLRDEIMTLFLAGHETTANVLSWTLYLLAREPEAEGKLLKELDRVLGGQPPAFEHIPLLTYTQSVVKESMRLYPPVWLISREPIEDVEIGGYTLPAGCEISVCQWVMHRLPEYFEEPERFQPERWTPEFEKSLPAGVYIPFGAGPRVCIGNQFAMMEAVLLLASIGQRFRLTLEPGHKVLLEPSITLRPQTGIRVRVWKRSAEQEKAD

>CYP1179A1(UB51_23875)Paenibacillus sp. IHBB 10380

MNSLPEGEDRHFKDIVDIISTWMIYNDRPDHTRLRSHMNRAFWTNEVEAIKPEIKNIVSQIIQKVVETKPDGFDFVVDIAHPIPAMVLCKMLGIPVQEIERFIKWSDDIAMFMQNFVVALVPDKEISEQIKKSLKEMYAFLSEAIADRRREKKNDLLSRLISDNPGENDGLRDDEIIAQTIHLIFGGHKIPQFMLSNTLHLLFKNPKVFNELKNDMSMLPKVQDECMRLEGPIQYITRHAAHDIEIHGQLIKEGDSVFFFLGSAGRDERKFENPEAFILERNSKHLGFGGGYHACIAASFARAEITEIMLEVIEKFPNISPLYDLNKPSWTMNPTFHGITTMPVKF

>CYP1179A3(GFC30_754)Anoxybacillus amylolyticus

METIIESQNVEQFSVYSDDYLHNRYNYYKRLRDNAPVYWNEEMRSWFITRYDDITKYLQGDTFITSPLIWHKMKNLPEGEDKHFKEIIDIISTWMIYNDRPIHTRLRKYMNRAFWKEEIEAITPEIKKIVTSVLDQVVEKHSNEFDFVSEVAHPIPALVLCKMLGIPGEEVERFIRWSDDIAQFMQNFVVSHVPDKDISDQTKKSMKEMYMFLSEAIAERRIKKRNDLLSRLISDNPGEEGELCDDELIAQTIHLIFGGHKIPQFMLSNTLHLLFKHPKVFDELKKDLSLIPKVLDEAMRLEGPIQYITRHAAQDITIHNQLIREGDSVYFFLGSAGRDERVFENPEKFDINRTGKQHIGFGGGYHACIAAAFARVEIAEILKEVIQRFSHITPLYNLERPEWTPNPTFHGIVTMPIKY

>CYP197AF1(BpOF4_07455)Bacillus pseudofirmus

MTNRFQRRKPIPGPKEHWLKGSLQAFTSDPLRFLSNQAETFGPVSSFRFGPFQEVYFVNDPDLIKEILVTKQKAFIKSRDIQMLKAVVGEGLLTNEKESHLKQRRLIQPAFKKTHIHQYAQDMIETTNAFIKGWKGEEERNIAADMMNIALGIITKTMFGMEMGQGADVIEQPMEAVMKLGIKRMRSLSPLPLWVPTQANRQLKKAVKELDDVLFSIISKRRLEDNQSEDLLGMLMKARDEENGAVMSDQQLRDELMTIFLAGHETTANLLAWTLYLLSEHPSADERLYAEIKEVTNGEALLPEHYTKLTYTQNVISESMRLYPPAYVIGRQVEEDIEIGPYLFNKGAMVLISQYVMHRNASFYHEPNIFKPERFDHNFLKTLPPFAYFPFGGGPRVCIGNHFAMMEATLALAAIAQNYKFTLTSSQQKVTPQPLITLRPKGGLMMKVEKRK

>CYP197AF2(Bcell_3878)Bacillus cellulosilyticus

MNNRRNSNYPPGPKEKWLTGSLRAFQSSPLKFLTSLSEKYGTVSKFRLGPFQDVYLVNDPDLIKEILVSKQQSFIKSRDIQSLKSIVGNGLLTSEKGFHLKQRRMIQPAFKKTHITTYAQDMIDTTNKYISRWSSRAERLVSDDMMDIALGIISKTMFSMEFEEGASVIGEPMEETMRTAVRRMRSILPLPLWIPVKQNRKYKQAIKELDNVLFRLIKERKETEVEHEDLLGVLMRAKDETDGLSMEDNQLRDELMTIFLAGHETTANALTWTLYLLSQHRKIQDKLFKEIASITRDGPVKPEHFGRLTYAQHVISESLRLYPPAYVIGRQAAEDTEINGYRIKKGDMILMSQYVMQRNRKYYEDPHTFIPERFENDFIKTIPEYAYFPFGGGPRVCIGNHFAFMEAVLVLACLSKQFKFTSPHEPQKIKPQPLITLRPKYGLTLLTTKRR

>CYP197AF8(UP17_03685)Bacillus simplex

MSDTDFSHDSPKGPKGRLITGHSKEFISDTLGFLTHLAKEYGDVAKIRFGPFQNVYFISNPDLIKQVLVTKQKSFLKSKDFHALKPLLGEGLLTSEKEIHMRQRRLIQPSFKKSHISNYAQDMIDITMNYISDWKNREERIITEDMMSLALGIISKTMFSMNLKEGYDELGEPIEASMRIAVKRMRTLLQLPLWVPTKNNREFKNSIQRLDTVIYNFIEKRRADAERHEDMLGILMDARDDEDGLAMTNQQVRDELMTIFLAGHETTANVLSWTLYLLSQHPEVETKLFNEIDSVIGNRNPTPGDYMKLTYTQNIINESMRIYPPGYIVSRKVEEDVVIGGYHFKKGDMLLLSQYVMHHKPEYFDNPESFNPERFENNFVKTLPPFAYFPFGGGARVCIGNHFAMMEAVLVLACIAQRYRIKLAPDHHEVKPLPSITLRPKRGLRMIVEDRKESFREPLIDD

>CYP197AH1(BBR47_09990)Brevibacillus brevis

MNITTTGPKGLPISGNLMAFRRSPLQFIREAAEEHGEVVHFRFGPSRHIYLLTNPDHIKEVLVSKQAHFRKGKGLQVARAVVGDGILTSEGKKHLRQRRLMQPAFHRDRIATYGDVMVRQAVDLMSDWKTGELRDIHSDMMKVTLAIITETMFGKTVKEGADQIGHAIDVGLKYVANKGSSFIDIPLSVPTKSNREFLESSELLDKTIYSLIEARRNSEGEEHKDLLGMLLAARDEDDGEGMTDEQVRDEVMTIFVAGHETTANTMSWIFYLLATHPEVEKKLHDELSTVLCDKLPTVEDLPQLKYTNLIVQETLRLYPAAWTINREVVDEVEIGGHTYKPGETLMMSQYVMHRDPRYYEQAEEFIPERFDSDLLKRNPAYAYFPFGGGPRVCIGNNFALMEAALLLATIAQRYRLRLAEPNQTVEPEPLVTLRPKNGLPMRLEKR

>CYP197AH2(ACH33_14890)Aneurinibacillus sp. XH2

MSEIEILGPKGLPITGNLFAFRKNPLEFLRRAAEEYGDIVHLRFGPRHLYLISNPDYIKEVLVTKQAHFKKGKGLQVAKAVVGEGILTSEGKFHMRQRRLMQPSFRQDHIVSYGDTMVEYGEKLISKWKDGEERLISKDMMELTLSIITKTMFGTSINKNLNDIEHAIDVGLKYVSRRASSFIDIPESIPTKSNREFKEAAETLDKVIFSIIEERRKSEDENRGDLLSMLLAARDEDDGTGMTDHQVRDEVMTIFIAGHETTANTLSWTLYLLAQHPEAEQKLWSEIEQVLGGRKVTVHDLPKLKYAENIIWETLRLYPAAWAINREVTDEIEIGGHTFKPGESIMMSQYVMHRNPKYFDNPDAFIPERFAGNLLKEIPQFAFFPFGGGPRVCIGNHFAIMEATLILVTIAQKFQLRLADPHNPVEPEPVVTLRPKNGLRMIVKKR

>CYP197AH3(IJ22_13460)Paenibacillus naphthalenovorans

MSTTMNPIPGPKGLPFSGNLFAFRKDPLGFLTKVQREYGDVVYIRFGPSRHVYLISDPEQIKDILLTKQHAFHKAKGLQTAKAVVGEGILTSEGEKHMRQRRLLQPSFRKDRIGQYAEAMVDYTEHMLQTWRPGETRIVTEDMMQLTLDIITHTMFGTGITSGINEIGRAIEVGMKYVSHKASSFLDIPESIPTKSNVEFKRSAQTLDRVILGIIEERRKKPDANREDLLSMLLEARDEETGTGMSDKQVRDEVMTIFLAGHETTANTLSWTWYLLSQNPEAEQKFHEELDRVLGGRRPTHDDIDQLKYTRQIVWESMRVYPAVWAVNRQVVKEVEIGGRLYKPGDTLMMSQFVMHRNPKYYEEADRFLPERFEGDLLKQIPQFGYFPFGGGPRVCIGNHFALMEATLLLATIGSRYKLRLAPDHHEVRPEPLVTLRPKNGLKMVVTARKE

>CYP197AD1_ortholog(KNP414_02324)Paenibacillus mucilaginosus KNP414

MNPLTETKAPSALPKETRIGGSLAAFRQDPLEFMTQLHRTYGHAAVFRLGPQRFHALFHPDLLKEVFVTKADSFTKAGTFDELKRLTGEGLVLSQGEFHQRQRRILQPKLTRTQIQRLADDMAASTLTRLDSWKDGDTRDLTHDLFAITFDIIASTMFSYDSRSELDKIGHAFDSVNRIASEKIRQLVRLPLFVPTRQNREYTWALRTLDDIVFRLIGERRAQGPGSRQDLLSALMSAVDEQDGSGMTDRQLRDEIMTMFLAGHETSAHTLAWAFDFLMRHPEVETKLLEEWERVLGGRRPAADDYPALEYTQNVLWETLRLRPAGYITSRTAVEDVVIGPLSLRKGEVIMISPYPLHTSDKYFDDPLAFRPERFENGLLKSLPQMAYFPFGAGPRSCIGNHFAMLEMAQILVVIGQRYRLRHVPGHPPAVPEALLTLAPKGGIRVTAEGRQR

>CYP197AD1_ortholog(B2K_13140)Paenibacillus mucilaginosus K02

MNPLTETKAPSALPKETRIGGSLAAFRQDPLEFMTQLHRTYGHAAVFRLGPQRFHALFHPDLLKEVFVTKADSFTKAGTFDELKRLTGEGLVLSQGELHQRQRRILQPKLTRAQIQRFSDDMAASTLTRLDSWKDGDTRDLTHDLFAITFDIIASTMFSYDSRSELDKIGQAFDSVNRIASEKIRQLVRLPLFVPTRQNREYTGALRTLDDIVFRLIGERRAQGPGSRQDLLSALMSAVDEQDGSGMTDRQLRDEIMTMFLAGHETSAHTLAWAFDFLMRHPEVETKLLEEWERVLGGRLPAADDFPALEYTQNVLWETLRLRPAGYITSRTAVEDVVIGPLSLRKGEVIMISPYPLHTSDKYFDAPLAFRPERFENGFLKSLPQMAYFPFGAGPRSCIGNHFAMLEMVQILVVIGQRYRLRHVPGHPPAVPEALLTLAPKGGIRVSAERRQR

>CYP197AD1_ortholog(PM3016_2592)Paenibacillus mucilaginosus 3016

MNPLTETKAPSALPKETRIGSSLAAFRQDPLEFMTQLHRTYGHAAVFRLGPQRFHALFHPDLLKEVFVTKADSFTKAGTFDELKRLTGEGLVLSQGEFHQRQRRILQPKLTRTQIQRFADNMAASTLTRLDSWKDGDTRDLTHDLFAITFDIIASTMFSYDSRSELDMIGQAFDSVNRIASEKIRQLVRLPLFVPTRQNREYTGALRTLDDIVFRLIGERRAQGPGSRQDLLSALMSAVDEQDGSGMTDRQLRDEIMTMFLAGHETSAHTLAWAFDFLMRHTEVETKLLEEWERVLGGRLPAADDYPALEYTQNVLWETLRLRPAGYITSRTALEDVVIGPLSLRQGEVIMISPYPLHTSDKYFDAPLAFRPERFENGLLKSLPQMAYFPFGAGPRSCIGNHFAMLEMVQILVVIGQRYRLRHVPGHPPAVPEALLTLAPKGGIRVTAERRQR

>CYP197AE1(BRLA_c023860)Brevibacillus laterosporus

MILTLPPGPNKPLYNVFRTLHNILDFIKENADKYGDVVYYPLLNERIYQLNKQELIEEVFIKKQSSFIKNDRLQAKLIKNILGEGLFTANGEHWLRNRRMMAPEFHQKRIESYGKTIIEVTNRYIENWQEGETREIKIDMLNLALDVVMKSLFGHNNTIDEGKLIQALQTILRYYAKAVMFPIPLPGYSNYKRCAKEMDDMILQLISSRRSSGIYGDDLLGMLLSMRDENGEGLDDRQIRDEVMTLIVAGHETTSAALAFAFYLLSENPDVTEKMAAEISEVLDGHLPHIQDLPNLQYTTMVVNETLRLRSPAYILLRQAAEDIQIGEYMIPKDSIVLVSQYVMHRDPRYFEDPLVFRPERWADGLEKKLPTFVYFPFGGGPRMCIGQRFAMAEAVLILATIVQRYKLTAVNQDKLEVNASITLSPKALHMVVNKRM

>CYP1255A1(TPY_0681)Sulfobacillus acidophilus TPY

MPSKRHETGDIDVKNEERQPVPGPSAGGWRHLVWFHRQPLYWLKSLADQYGPLTAFRLGPWPFLLASGPKTVGTILRRHGKEFVKRPGLKTDNPLIGAGLLTTEGSYWAEERRREAPLFTPTNVKRLLTADVMPWSVKWADALPTDRPVELHSLFLRGTLDLVIRTLFADSGPQPLAAYQEDLAWIMHHFYHRLRSPFRPPYHWPWPFNRRYHEHARRLETFFDHWHPEPKLYETLGTRFNPNDPGDRAKAVTLLLAGQETTANALTWTLWLLATHPHWQQRLWEQPEELDGVLQESLRLYPPVWLISRETTRSVTFEGYDLPPHTKILISPWVSHRNPDVFPDPAAFQPQRWQSAAPPLDGYFPFGGGPRRCIGQAFAVGEMRAVIQAILARYRIEPAGPEPTVFPAMTLEPGTPVYVQLKSR

>CYP1341C1(DJ92_3552) Bacillus pseudomycoides 219298

MLQKIELKYDPLDPGTLLNPYPIYKKLRENAPVYWHEGMKSWVLTRYDDCKEVLRNHEVFTTDRRRVGVEIPDVRHNVQSLDPPDNIPLRNLLTRAFNSQDINNVREKIHVLIKDIFKKHKSINEFDFMREVSAPLALSMTAITLGVDEPNLDSFLEISEAITRQMDSGLRPENIEPGNQAREKLNALVGEWFAAEDRPGIVSYIRKHAQNTNVPEHYIRNTTGTMFNASFGSLYAVFGNVVLALLEHPEVFDKLNDKSLIDTGVDELIRFDGPAQGTGRIAAKTTKIRDTTIQKGDVIVVLFAAANRDPEVFPEPDSIILDRSKNPHLGFGWGPHTCVGTFFGKLAIKELILCLLEESSRLRLLRRPTRRVTATSRGIELLPVSFS

>CYP1706B1(TC41_3167)Alicyclobacillus acidocaldarius subsp. acidocaldarius Tc-4-1

MLAPPGSPLMGLLSLHRTFASDEFELAAPRLRVRVAAGPASVKRVLHEQADAYSSRLEGDPVAALFGQGLLVIDGPRHGAVKDVIVRSSTRGVLESRLRDTLPALDEALQRTLGQGTISLPDMPRRMTWRALESVYFHHRLTPEEERAYLPDLTRLVYYIGPGWWLLLGKTPRLPKAYHRMRLRLTAMYEKALIRRDNAPIHALARAYGDEEMSFAVDQLMTLLVAGHDTATSLLSSLLILLAHRPDLQDALWGEIHGATGHERPEVRALDRLPLLDAVVKETLRLYPPIHSGLRSEVESGDRVMVSYFLLHRHQELWPDADSFVPERWLSGLAPGAYTYLPFGAGPRYCPGATFARLEVKWMLARLLQHVSLQPTSRPPRIAMRAALEHRPTVIAVRRR

>CYP1706B1_ortholog(Aaci_2821)Alicyclobacillus acidocaldarius subsp. acidocaldarius DSM 446

MREDIHRGIPSTRSLRPEEGWRALRNMLVPPGSPLMGLLSLHRTFASDEFELSAPRLRMRVAAGPASVKRVLHEQADGYRSRLEGDPVTALFGQGLLVIDGPRHGAVKDVIVRSSTRGVLESRLRDTLPALDEALQRALGQGTISLPDMPRRMTWRALESVYFHHRLTPEEERAHLSDLTHLVHYIGPGWWLLLGKTPRLPKAYHRMRLRLSAMYERALIRPDNAPIHALARACGDEETSFAVDQLMTLLVAGHDTATSLLSSLLILLAQRPGLQDALWGEIQGATGRERPEIRVLDRLPLLDAVVKETLRLYPPIHSGLRSEVESGDRVMVSYFLLHRHQEIWPDADSFVPERWLTGLAPGAYTYLPFGAGPRYCPGATFARLEVKWILARLLQHVSLQPMSRPPRIAMRAALEHRPTVIAVRRR

>CYP1731A2(Bsel_0730)Bacillus selenitireducens

MAGKSFKFPARLRSYLYFRKDPIGFFMNMQGDGDIVFLDTGVMPTYVVNGASYLQEILVTKDASFRKGRPSNVLKRTIGDGLLTTEKTAHKIQKSGMQPVFYKDRLKRYAEVMVSDAKETVRGLHDDKEIRMDDEMMALTLSVISKVMFATDAGTRKKKLADAVNVTIHQSARNLFSPVLLPMSVPTKGNRDHSKAIRVLEEEVHAILKEARRNPDAYKETMIGLLMDAKALDEQGLITDQELRDQMMTMLLAGHETSANLLTWIFYLLAEHPDVARKLQEEVDQVDLTEDPFAATRSLPYTHQVIKETLRLYPPAWLIYREADEDVELSGKTYKEGTVFMMSTYAIHRNPDVFDDPEAFRPDRFAGDQEKNLPPFTYIPFGAGSRSCIGYRFAMMETALILAVIAKSYHFERAGTETIKGDPLISLRVKDGLRMIAKERKQTKIPEKSS

>CYP1756A1(BMWSH_3237)Bacillus megaterium WSH-002

MITLPVIQGPSSYKLTGHLQKFRENPLGFLENLTQYGEIATFRVAHKRFYVTRDPQLIKDVVITNNKAFQKIKLTHMFKTLLGEEMLWTDEALYMSPIQPSQIKQHLTYNKEAIAKIIEKHTETWEEGQLRMIVKDIRQIVVAVLLQLVFGISIQDKDKIHYVQALMRKKEKLGKIYIRLPLHQLDSDEQLEQLLFERVQMRVQNKTEGKDLLQYILNSCGEDIDEREIYQQLNSIFLSMYEMITHVCSWSIYLLSQNTREHLQLHKEIQAYASGESLSTKNLTYMRKIIAESMRLYPPLWLFGRQAREDIQIDGYSIKKGEIMLISPYMMHRHEDYFLEPSEFLPDRFEKGGSIDVPSYMYMPLGIDHQAERGMDYITEIVTIFLSEMTKRFLFQLTKPESIAPMAGVMLNMKEELNVNVHKVHTQS

>CYP1756A1_ortholog(BMQ_2039)Bacillus megaterium QM B1551

MENLTQYGEIATFRVAHKRFYVTRDPQLIKDVVITNSKAFQKIKLTHMFKTLLGEEMLWTDEALYMSPIQPSQLKQHLTYNKEVIAKIIEKHTETWEEGQLRTIVKDIRQIVVAVLLQLVFGISIQDKDKIHYVQALMRKKEKLGKIYIRLPLHQPDSDEQLEQLLFERVQMRVQNKTEGNDLLEYILNSCGEEIDEREIYEQLKSIFLSMYEMITHVCSWSIHLLSQNTREHLQLHKEIQAYASGESLSTKNLTYMRKIIAESMRLYPPLWLFGRQAREDIQIDGYSIKKGEIMLISPYMMHRHEDYFLEPSEFLPDRFEKGGSIDVPSYMYMPLGIEHQAERGMDYITEIVTIFMSEMTKRFLFQLTKPESIAPMAGVMLNMKEELNVNVHKVHTQS

>CYP1756A1_ortholog(BG04_4340)Bacillus megaterium NBRC 15308 = ATCC 14581

MENLTQYGEIATFRVAHKRFYVTRDPQLIKDVVITNSKAFQKIKLTHMFKTLLGEEMLWTDEALYMSPIQPSQLKQHLTFNKEAIAKIIEKHTETWEEGQLRTIVKDIRQIVIAVLLQLVFGISIEEKDKIHYVQALMRKKEKLGKIYIRLPLHQPDSDEQLEQLLFERVQMRVQNKTAGNDLLQYILNSYGEDSDEREIYEQLNSIFLSMYEMITHVCSWSIHLLSQNTREHLQLHKEIQAYASGESLSTKNLTYMRKIIAESMRLYPPLWLFGRQAREDIQIDGYSIKKGEIMLISPYMMHRHEDYFLEPSEFLPDRFEKGGSIDVPSYMYMPLGIEHQAERGMDYITEIVTIFLSEMTKRFLFQLTKPESIAPMAGVMLNMKEELKVNVHKVHAQS

>CYP1756A1_ortholog(BMD_1995)Bacillus megaterium DSM 319

MENLTQYGEIATFRVAHKRFYVTRDPQLIKDVVITNSKAFQKIKLTHMFKTLLGEEMLWTDEALYMSPIQPSQLKQHLTYNKEAIAKIIEKHTETWEEGQLRTIVKDIRQIVVAVLLQLVFGISIEEKDKIHYVQALMRKKEKLGKIYIRLPLHQPDSDEQLEQLLFERVQMRIQNKTGGNDLLQYILNSYGEDSDEREIYEQLNSIFLSMYEMITHVCSWSIHLLSQNTREHLQLHKEIQAYASGESLSTKNLTYMRKIIAESMRLYPPLWLFGRQAREDIQIDGYNIKKGEIMLISSYMMHRHEDYFLEPSEFLPDRFEKGGSIDVPSYMYMPLGIEHQAERGMDYITEIVTIFLSEMTKRFLFQLTKPESIAPMAGVMLNMKEELKVNVHKVHAQS

>CYP152A2(AAK81262.1)Clostridium acetobutylicum ATCC 824

MLLKENTAKDKGIDSTLDLLKEGYLFIKNRADHYQSDLFETRLMGQRIICMTGEEAARIFYDSDKFKRQG

AAPKRVQETLLGENAIQTLDGESHLHRKKLFMLLTNQVQQKRLAELTTEKWEASASKWHTKSIVLFNEAN

EILCQVACHWAGVPLMESDIKNRAEDFSSMIDSFGAVGPRHWKGKKARNTIEAWIKEIIENVRSGRIRAE

EGSPLHEIAFYIDVNGQQMPAEMAAIELINILRPIVAISTFITFSALALYEHSEYREKLQSKDIRYLEMF

TQEVRRYYPFAPFVGARVRKDFLWNNCEFKKEMLVLLDIYGTNHDSRIWQKPYEFIPDRFRSYKGNLFDF

IPQGGGDPSSTHRCPGEGITLEIMKTSLDFLSTKIDFTVPDQDLSYSLSKIPTLPKSGFIIDNINLKL

>CYP152A2(AEI34134.1)Clostridium acetobutylicum DSM 1731

MLLKENTAKDKGIDSTLDLLKEGYLFIKNRADHYQSDLFETRLMGQRIICMTGEEAARIFYDSDKFKRQG

AAPKRVQETLLGENAIQTLDGESHLHRKKLFMLLTNQVQQKRLAELTTEKWEASASKWHTKSIVLFNEAN

EILCQVACHWAGVPLMESDIKNRAEDFSSMIDSFGAVGPRHWKGKKARNTIEAWIKEIIENVRSGRIRAE

EGSPLHEIAFYIDVNGQQMPAEMAAIELINILRPIVAISTFITFSALALYEHSEYREKLQSKDIRYLEMF

TQEVRRYYPFAPFVGARVRKDFLWNNCEFKKEMLVLLDIYGTNHDSRIWQKPYEFIPDRFRSYKGNLFDF

IPQGGGDPSSTHRCPGEGITLEIMKTSLDFLSTKIDFTVPDQDLSYSLSKIPTLPKSGFIIDNINLKL

>CYP152A2(ADZ22370.1)Clostridium acetobutylicum EA 2018

MLLKENTAKDKGIDSTLDLLKEGYLFIKNRADHYQSDLFETRLMGQRIICMTGEEAARIFYDSDKFKRQG

AAPKRVQETLLGENAIQTLDGESHLHRKKLFMLLTNQVQQKRLAELTTEKWEASASKWHTKSIVLFNEAN
[truncated: 50,250 more chars]
