# Supplementary material for: Impact of lifestyle on cytochrome P450 monooxygenase repertoire is clearly evident in the bacterial phylum Firmicutes
Source: Sci Rep. 2020 Aug 19;10:13982. doi: 10.1038/s41598-020-70686-8 (PMC7438502; doi:10.1038/s41598-020-70686-8)
Supplement: Supplementary file 2 — Supplementary file2 [file 41598_2020_70686_MOESM2_ESM.pdf]

Impact of lifestyle on cytochrome P450 monooxygenase repertoire is clearly evident in the bacterial phylum *Firmicutes*

Tiara Padayachee[1], Nomfundo Nzuza[1], Wanping Chen[2], David R Nelson[3]*, Khajamohiddin Syed[1*]

[1] Department of Biochemistry and Microbiology, Faculty of Science and Agriculture, University of Zululand, KwaDlangezwa 3886, South Africa.

[2] Department of Molecular Microbiology and Genetics, University of Göttingen, 37077 Göttingen, Germany

[3] Department of Microbiology, Immunology and Biochemistry, University of Tennessee Health Science Center, Memphis, TN, 38163; drnelson1@gmail.com

* Corresponding authors' email:

drnelson1@gmail.com and khajamohiddinsyed@gmail.com

Supplementary Dataset 2. A high-resolution phylogenetic tree of *Firmicutes* species P450s. Different P450 families are indicated with different colors.

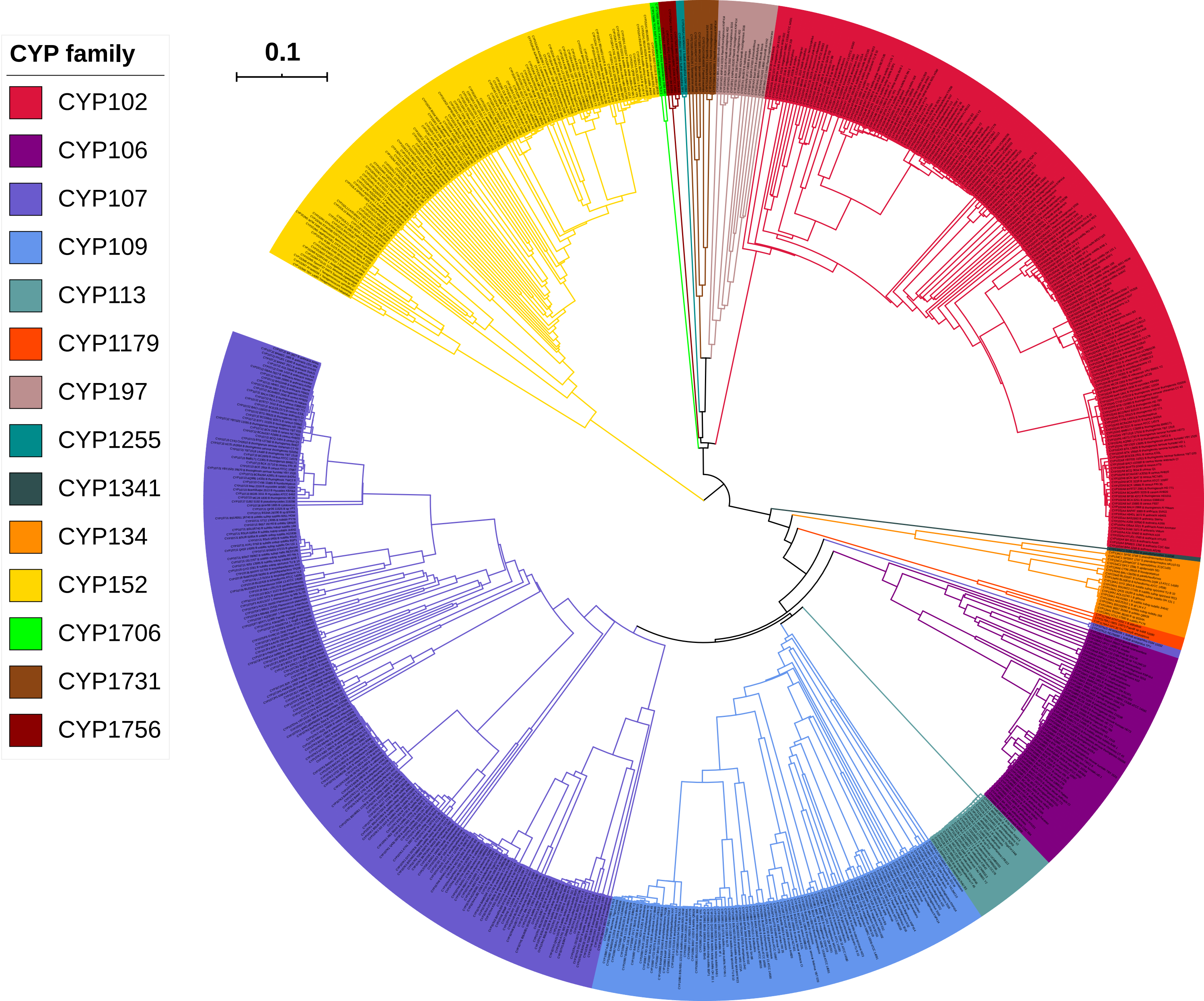
CYP family
CYP102
CYP106
CYP107
CYP109
CYP113
CYP1179
CYP197
CYP1255
CYP1341
CYP134
CYP152
CYP1706
CYP1731
CYP1756
0.1
