# Supplementary material for: Impact of lifestyle on cytochrome P450 monooxygenase repertoire is clearly evident in the bacterial phylum Firmicutes
Source: Sci Rep. 2020 Aug 19;10:13982. doi: 10.1038/s41598-020-70686-8 (PMC7438502; doi:10.1038/s41598-020-70686-8)
Supplement: Supplementary file 4 — Supplementary file4 [file 41598_2020_70686_MOESM4_ESM.docx]

**Impact of lifestyle on cytochrome P450 monooxygenase repertoire is clearly evident in the bacterial phylum *Firmicutes***

Tiara Padayachee^1^, Nomfundo Nzuza^1^, Wanping Chen^2^, David R Nelson^3^*, Khajamohiddin Syed^1*^

^1^ Department of Biochemistry and Microbiology, Faculty of Science and Agriculture, University of Zululand, KwaDlangezwa 3886, South Africa.

^2^ Department of Molecular Microbiology and Genetics, University of Göttingen, 37077 Göttingen, Germany

^3^ Department of Microbiology, Immunology and Biochemistry, University of Tennessee Health Science Center, Memphis, TN, 38163; drnelson1@gmail.com

* Corresponding authors’ email:

drnelson1@gmail.com and khajamohiddinsyed@gmail.com

**Table S1. Information on species used in the study.**

| **Genus** | **No of species analyzed** | **No of species have P450s** | **No of species don’t have P450s** | **Species code** | **Species name** | **Genome ID** |
| --- | --- | --- | --- | --- | --- | --- |
| *Streptococcus* | 143 | 0 | 143 | sagm | *Streptococcus agalactiae* 09mas018883 | HF952104 |
|  |  |  |  | sagp | *Streptococcus agalactiae* 138P | CP007482 |
|  |  |  |  | sagc | *Streptococcus agalactiae* 138spar | CP007565 |
|  |  |  |  | sagl | *Streptococcus agalactiae* 2-22 (serotype Ib) | FO393392 |
|  |  |  |  | sag | *Streptococcus agalactiae* 2603 (serotype V) | AE009948(NC_004116) |
|  |  |  |  | sak | *Streptococcus agalactiae* A909 (serotype Ia) | CP000114 |
|  |  |  |  | sagn | *Streptococcus agalactiae* CNCTC 10/84 | CP006910 |
|  |  |  |  | sagt | *Streptococcus agalactiae* COH1 | HG939456 |
|  |  |  |  | sgc | *Streptococcus agalactiae* GD201008-001 (serotype Ia) | CP003810 |
|  |  |  |  | sagi | *Streptococcus agalactiae* ILRI005 | HF952105 |
|  |  |  |  | sagr | *Streptococcus agalactiae* ILRI112 | HF952106 |
|  |  |  |  | san | *Streptococcus agalactiae* NEM316 (serotype III) | AL732656 |
|  |  |  |  | sage | *Streptococcus agalactiae* NGBS061 | CP007631 |
|  |  |  |  | sagg | *Streptococcus agalactiae* NGBS572 | CP007632 |
|  |  |  |  | sags | *Streptococcus agalactiae* SA20 | CP003919 |
|  |  |  |  | sang | *Streptococcus anginosus* C1051 | CP003860 |
|  |  |  |  | sanc | *Streptococcus anginosus* C238 | CP003861 |
|  |  |  |  | sans | *Streptococcus anginosus* SA1 | CP007573 |
|  |  |  |  | scg | *Streptococcus constellatus* subsp. *pharyngis* C1050 | CP003859 |
|  |  |  |  | scon | *Streptococcus constellatus* subsp. *pharyngis* C232 | CP003800 |
|  |  |  |  | scos | *Streptococcus constellatus* subsp. *pharyngis* C818 | CP003840 |
|  |  |  |  | soi | *Streptococcus cristatus* | CP004409 |
|  |  |  |  | sdq | *Streptococcus dysgalactiae* subsp. *equisimilis* 167 | AP012976 |
|  |  |  |  | sdc | *Streptococcus dysgalactiae* subsp. *equisimilis* AC-2713 | HE858529 |
|  |  |  |  | sdg | *Streptococcus dysgalactiae* subsp. *equisimilis* ATCC 12394 | CP002215 |
|  |  |  |  | sds | *Streptococcus dysgalactiae* subsp. *equisimilis* GGS_124 | AP010935 |
|  |  |  |  | sda | *Streptococcus dysgalactiae* subsp. *equisimilis* RE378 | AP011114 |
|  |  |  |  | seu | *Streptococcus equi* subsp. *equi* 4047 | FM204883 |
|  |  |  |  | sezo | *Streptococcus equi* subsp. *zooepidemicus* ATCC 35246 | CP002904 |
|  |  |  |  | sequ | *Streptococcus equi* subsp. *zooepidemicus* CY | CP006770 |
|  |  |  |  | seq | *Streptococcus equi* subsp. *zooepidemicus* H70 | FM204884 |
|  |  |  |  | sez | *Streptococcus equi* subsp. *zooepidemicus* MGCS10565 | CP001129 |
|  |  |  |  | sgt | *Streptococcus gallolyticus* subsp. *gallolyticus* ATCC 43143 | AP012053 |
|  |  |  |  | sgg | *Streptococcus gallolyticus* subsp. *gallolyticus* ATCC BAA-2069 | FR824043 |
|  |  |  |  | sga | *Streptococcus gallolyticus* UCN34 | FN597254 |
|  |  |  |  | sgo | *Streptococcus gordonii* | CP000725 |
|  |  |  |  | sif | *Streptococcus infantarius* | CP003295 |
|  |  |  |  | siq | *Streptococcus iniae* ISET0901 | CP007586 |
|  |  |  |  | sio | *Streptococcus iniae* ISNO | CP007587 |
|  |  |  |  | sik | *Streptococcus iniae* SF1 | CP005941 |
|  |  |  |  | siz | *Streptococcus iniae* SFST01-82 | CP010783 |
|  |  |  |  | sib | *Streptococcus intermedius* B196 | CP003857 |
|  |  |  |  | siu | *Streptococcus intermedius* C270 | CP003858 |
|  |  |  |  | sie | *Streptococcus intermedius* JTH08 | AP010969 |
|  |  |  |  | slu | *Streptococcus lutetiensis* | CP003025 |
|  |  |  |  | smn | *Streptococcus macedonicus* | HE613569 |
|  |  |  |  | smb | *Streptococcus mitis* | FN568063(NC_013853) |
|  |  |  |  | smut | *Streptococcus mutans* GS-5 (serotype c) | CP003686 |
|  |  |  |  | smj | *Streptococcus mutans* LJ23 (serotype k) | AP012336 |
|  |  |  |  | smc | *Streptococcus mutans* NN2025 (serotype c) | AP010655 |
|  |  |  |  | smu | *Streptococcus mutans* UA159 (serotype c) | AE014133(NC_004350) |
|  |  |  |  | smua | *Streptococcus mutans* UA159-FR | CP007016 |
|  |  |  |  | sor | *Streptococcus oralis* | FR720602 |
|  |  |  |  | spat | *Streptococcus pantholopis* | CP014699 |
|  |  |  |  | scp | *Streptococcus parasanguinis* ATCC 15912 | CP002843 |
|  |  |  |  | scf | *Streptococcus parasanguinis* FW213 | CP003122 |
|  |  |  |  | stk | *Streptococcus parauberis* | CP002471 |
|  |  |  |  | stb | *Streptococcus pasteurianus* | AP012054 |
|  |  |  |  | snb | *Streptococcus pneumoniae* 670-6B (serotype 6B) | CP002176 |
|  |  |  |  | snm | *Streptococcus pneumoniae* 70585 (serotype 5) | CP000918 |
|  |  |  |  | spnn | *Streptococcus pneumoniae* A026 (serotype 19F) | CP006844 |
|  |  |  |  | snp | *Streptococcus pneumoniae* AP200 (serotype 11A) | CP002121 |
|  |  |  |  | sne | *Streptococcus pneumoniae* ATCC 700669 (serotype 23F ST81 lineage) | FM211187 |
|  |  |  |  | spw | *Streptococcus pneumoniae* CGSP14 (serotype 14) | CP001033 |
|  |  |  |  | spd | *Streptococcus pneumoniae* D39 (virulent serotype 2) | CP000410 |
|  |  |  |  | spx | *Streptococcus pneumoniae* G54 (serotype 19F) | CP001015 |
|  |  |  |  | spng | *Streptococcus pneumoniae* gamPNI0373 (virulent serotype 1) | CP001845 |
|  |  |  |  | spv | *Streptococcus pneumoniae* Hungary19A 6 (serotype 19A) | CP000936 |
|  |  |  |  | sni | *Streptococcus pneumoniae* INV104 (serotype 1) | FQ312030 |
|  |  |  |  | snv | *Streptococcus pneumoniae* INV200 (serotype 14) | FQ312029 |
|  |  |  |  | sjj | *Streptococcus pneumoniae* JJA (serotype 14) | CP000919 |
|  |  |  |  | snx | *Streptococcus pneumoniae* OXC141 (serotype 3) | FQ312027 |
|  |  |  |  | spp | *Streptococcus pneumoniae* P1031 (serotype 1) | CP000920 |
|  |  |  |  | spr | *Streptococcus pneumoniae* R6 (avirulent serotype 2) | AE007317(NC_003098) |
|  |  |  |  | spne | *Streptococcus pneumoniae* SPN034156 (serotype 3) | FQ312045 |
|  |  |  |  | spnu | *Streptococcus pneumoniae* SPN034183 (serotype 3) | FQ312043 |
|  |  |  |  | spnm | *Streptococcus pneumoniae* SPN994038 (serotype 3) | FQ312041 |
|  |  |  |  | spno | *Streptococcus pneumoniae* SPN994039 (serotype 3) | FQ312044 |
|  |  |  |  | snu | *Streptococcus pneumoniae* SPNA45 (serotype 3) | HE983624 |
|  |  |  |  | snd | *Streptococcus pneumoniae* ST556 (serotype 19F) | CP003357 |
|  |  |  |  | snt | *Streptococcus pneumoniae* Taiwan19F-14 (serotype 19F) | CP000921 |
|  |  |  |  | snc | *Streptococcus pneumoniae* TCH8431/19A (serotype 19A) | CP001993 |
|  |  |  |  | spn | *Streptococcus pneumoniae* TIGR4 (virulent serotype 4) | AE005672 |
|  |  |  |  | std | *Streptococcus pseudopneumoniae* | CP002925 |
|  |  |  |  | spya | *Streptococcus pyogenes* A20 (serotype M1) | CP003901 |
|  |  |  |  | stz | *Streptococcus pyogenes* Alab49 (serotype M53) | CP003068 |
|  |  |  |  | spyh | *Streptococcus pyogenes* HSC5 (serotype M14) | CP006366 |
|  |  |  |  | spym | *Streptococcus pyogenes* M1 476 (serotype M1) | AP012491 |
|  |  |  |  | spy | *Streptococcus pyogenes* M1 GAS (serotype M1) | AE004092(NC_002737) |
|  |  |  |  | spf | *Streptococcus pyogenes* Manfredo (serotype M5) | AM295007 |
|  |  |  |  | sph | *Streptococcus pyogenes* MGAS10270 (serotype M2) | CP000260 |
|  |  |  |  | spa | *Streptococcus pyogenes* MGAS10394 (serotype M6) | CP000003 |
|  |  |  |  | spi | *Streptococcus pyogenes* MGAS10750 (serotype M4) | CP000262 |
|  |  |  |  | stg | *Streptococcus pyogenes* MGAS15252 (serotype M59) | CP003116 |
|  |  |  |  | stx | *Streptococcus pyogenes* MGAS1882 (serotype M59) | CP003121 |
|  |  |  |  | spj | *Streptococcus pyogenes* MGAS2096 (serotype M12) | CP000261 |
|  |  |  |  | spg | *Streptococcus pyogenes* MGAS315 (serotype M3) | AE014074 |
|  |  |  |  | spz | *Streptococcus pyogenes* MGAS5005 (serotype M1) | CP000017 |
|  |  |  |  | spb | *Streptococcus pyogenes* MGAS6180 (serotype M28) | CP000056 |
|  |  |  |  | spm | *Streptococcus pyogenes* MGAS8232 (serotype M18) | AE009949 |
|  |  |  |  | spk | *Streptococcus pyogenes* MGAS9429 (serotype M12) | CP000259 |
|  |  |  |  | soz | *Streptococcus pyogenes* NZ131 (serotype M49) | CP000829 |
|  |  |  |  | sps | *Streptococcus pyogenes* SSI-1 (serotype M3) | BA000034 |
|  |  |  |  | spyo | *Streptococcus pyogenes* STAB901 (serotype M44) | CP007024 |
|  |  |  |  | stf | *Streptococcus salivarius* 57.I | CP002888 |
|  |  |  |  | ssr | *Streptococcus salivarius* CCHSS3 | FR873481 |
|  |  |  |  | ssah | *Streptococcus salivarius* HSISS4 | CP013216 |
|  |  |  |  | stj | *Streptococcus salivarius* JIM8777 | FR873482 |
|  |  |  |  | strs | *Streptococcus salivarius* NCTC 8618 | CP009913 |
|  |  |  |  | ssa | *Streptococcus sanguinis* | CP000387(NC_009009) |
|  |  |  |  | ssob | *Streptococcus sobrinus* | CP029491 |
|  |  |  |  | stra | *Streptococcus* sp. A12 | CP013651 |
|  |  |  |  | sig | *Streptococcus* sp. I-G2 | CP006805 |
|  |  |  |  | sip | *Streptococcus* sp. I-P16 | CP006776 |
|  |  |  |  | strn | *Streptococcus* sp. NPS 308 | AP017652 |
|  |  |  |  | stv | *Streptococcus* sp. VT 162 | CP007628 |
|  |  |  |  | ssu | *Streptococcus suis* 05ZYH33 (serotype 2) | CP000407 |
|  |  |  |  | ssv | *Streptococcus suis* 98HAH33 (serotype 2) | CP000408 |
|  |  |  |  | ssf | *Streptococcus suis* A7 (serotype 2) | CP002570 |
|  |  |  |  | ssb | *Streptococcus suis* BM407 (serotype 2) | FM252032(NC_012926) |
|  |  |  |  | ssk | *Streptococcus suis* D12 (serotype 9) | CP002644 |
|  |  |  |  | ssq | *Streptococcus suis* D9 (serotype 7) | CP002641 |
|  |  |  |  | ssw | *Streptococcus suis* GZ1 (serotype 2) | CP000837 |
|  |  |  |  | sui | *Streptococcus suis* JS14 (serotype 14) | CP002465 |
|  |  |  |  | ssi | *Streptococcus suis* P1/7 (serotype 2) | AM946016 |
|  |  |  |  | sup | *Streptococcus suis* S735 (serotype 2) | CP003736 |
|  |  |  |  | ssus | *Streptococcus suis* SC070731 (serotype 2) | CP003922 |
|  |  |  |  | sss | *Streptococcus suis* SC84 (serotype 2) | FM252031 |
|  |  |  |  | suo | *Streptococcus suis* SS12 (serotype 1/2) | CP002640 |
|  |  |  |  | srp | *Streptococcus suis* ST1 (serotype 1) | CP002651 |
|  |  |  |  | sst | *Streptococcus suis* ST3 (serotype 3) | CP002633 |
|  |  |  |  | ssui | *Streptococcus suis* T15 | CP006246 |
|  |  |  |  | ssut | *Streptococcus suis* TL13 (serotype 16) | CP003993 |
|  |  |  |  | ssuy | *Streptococcus suis* YB51 (serotype 3) | CP006645 |
|  |  |  |  | sthe | *Streptococcus thermophilus* ASCC 1275 | CP006819 |
|  |  |  |  | stc | *Streptococcus thermophilus* CNRZ1066 | CP000024 |
|  |  |  |  | stu | *Streptococcus thermophilus* JIM 8232 | FR875178 |
|  |  |  |  | ste | *Streptococcus thermophilus* LMD-9 | CP000419 |
|  |  |  |  | stl | *Streptococcus thermophilus* LMG 18311 | CP000023 |
|  |  |  |  | stw | *Streptococcus thermophilus* MN-ZLW-002 | CP003499 |
|  |  |  |  | stn | *Streptococcus thermophilus* ND03 | CP002340 |
|  |  |  |  | sths | *Streptococcus thermophilus* S9 | CP013939 |
|  |  |  |  | sub | *Streptococcus uberis* | AM946015 |
| *Bacillus* | 129 | 118 | 11 | balt | *Bacillus altitudinis* | CP022319 |
|  |  |  |  | bamc | *Bacillus amyloliquefaciens* CC178 | CP006845 |
|  |  |  |  | bao | *Bacillus amyloliquefaciens* DSM 7 | FN597644 |
|  |  |  |  | bami | *Bacillus amyloliquefaciens* IT-45 | CP004065 |
|  |  |  |  | bamf | *Bacillus amyloliquefaciens* LFB112 | CP006952 |
|  |  |  |  | bql | *Bacillus amyloliquefaciens* LL3 | CP002634 |
|  |  |  |  | baz | *Bacillus amyloliquefaciens* TA208 | CP002627 |
|  |  |  |  | bxh | *Bacillus amyloliquefaciens* XH7 | CP002927 |
|  |  |  |  | bgy | *Bacillus amyloliquefaciens* Y2 | CP003332 |
|  |  |  |  | bai | *Bacillus anthracis* A0248 | CP001598 |
|  |  |  |  | bant | *Bacillus anthracis* A16 | CP001970 |
|  |  |  |  | banr | *Bacillus anthracis* A16R | CP001974 |
|  |  |  |  | ban | *Bacillus anthracis Ames* | (NC_003997) |
|  |  |  |  | bar | *Bacillus anthracis Ames Ancestor* | AE017334 |
|  |  |  |  | bah | *Bacillus anthracis* CDC 684 | CP001215 |
|  |  |  |  | bax | *Bacillus anthracis* H9401 | CP002091 |
|  |  |  |  | banh | *Bacillus anthracis* HYU01 | CP008846 |
|  |  |  |  | bat | *Bacillus anthracis Sterne* | (NC_005945) |
|  |  |  |  | bans | *Bacillus anthracis* SVA11 | CP006742 |
|  |  |  |  | banv | *Bacillus anthracis Vollum* | CP007666 |
|  |  |  |  | bae | *Bacillus atrophaeus* 1942 | CP002207 |
|  |  |  |  | batr | *Bacillus atrophaeus* NRS 1221A | CP010778 |
|  |  |  |  | bbev | *Bacillus beveridgei* | CP012502 |
|  |  |  |  | bby | *Bacillus bombysepticus* | CP007512 |
|  |  |  |  | bco | *Bacillus cellulosilyticus* | CP002394 |
|  |  |  |  | bcx | *Bacillus cereus* 03BB102 | CP001407 |
|  |  |  |  | bcr | *Bacillus cereus* AH187 | CP001177 |
|  |  |  |  | bcu | *Bacillus cereus* AH820 | CP001283 |
|  |  |  |  | bca | *Bacillus cereus* ATCC 10987 | AE017194 |
|  |  |  |  | bce | *Bacillus cereus* ATCC 14579 | (NC_004722) |
|  |  |  |  | bcb | *Bacillus cereus* B4264 | CP001176 |
|  |  |  |  | bal | *Bacillus cereus biovar anthracis* CI | CP001746 |
|  |  |  |  | bcz | *Bacillus cereus* E33L | CP000001 |
|  |  |  |  | bcf | *Bacillus cereus* F837/76 | CP003187 |
|  |  |  |  | bcer | *Bacillus cereus* FRI*-*35 | CP003187 |
|  |  |  |  | bcef | *Bacillus cereus* FT9 | CP008712 |
|  |  |  |  | bcg | *Bacillus cereus* G9842 | CP001186 |
|  |  |  |  | bnc | *Bacillus cereus* NC7401 | AP007209 |
|  |  |  |  | bcq | *Bacillus cereus* Q1 | CP000227 |
|  |  |  |  | bcl | *Bacillus clausii* | AP006627 |
|  |  |  |  | bag | *Bacillus coagulans* 36D1 | CP003056 |
|  |  |  |  | bcy | *Bacillus cytotoxicus* | CP000764 |
|  |  |  |  | beo | *Bacillus endophyticus* | CP011974 |
|  |  |  |  | bfx | *Bacillus flexus* | CP016790 |
|  |  |  |  | bgi | *Bacillus gibsonii* | CP017070 |
|  |  |  |  | bgy | *Bacillus glycinifermentans* | LT603683 |
|  |  |  |  | bha | *Bacillus halodurans* | CP001215 |
|  |  |  |  | bhk | *Bacillus horikoshii* | CP020880 |
|  |  |  |  | bif | *Bacillus infantis* | CP006643 |
|  |  |  |  | bko | *Bacillus kochii* | CP022983 |
|  |  |  |  | bkw | *Bacillus krulwichiae* | CP020814 |
|  |  |  |  | ble | *Bacillus lehensis* | CP003923 |
|  |  |  |  | bli | *Bacillus licheniformis* ATCC 14580 | CP000002 |
|  |  |  |  | bld | *Bacillus licheniformis* DSM 13 = ATCC 14580 | AE017333 |
|  |  |  |  | bmd | *Bacillus megaterium* DSM 319 | CP001982 |
|  |  |  |  | bmeg | *Bacillus megaterium* NBRC 15308 = ATCC 14581 | CP009920 |
|  |  |  |  | bmq | *Bacillus megaterium* QM B1551 | CP001983 |
|  |  |  |  | bmh | *Bacillus megaterium* WSH*-*002 | CP003017 |
|  |  |  |  | bmet | *Bacillus methanolicus* | CP007739 |
|  |  |  |  | bmyc | *Bacillus mycoides* 219298 | CP007626 |
|  |  |  |  | bmyo | *Bacillus mycoides* ATCC 6462 | CP009692 |
|  |  |  |  | bwe | *Bacillus mycoides* KBAB4 | CP000903 |
|  |  |  |  | bww | *Bacillus mycoides* WSBC 10204 | CP009746 |
|  |  |  |  | bon | *Bacillus oceanisediminis* | CP015506 |
|  |  |  |  | blh | *Bacillus paralicheniformis* | CP005965 |
|  |  |  |  | bpf | *Bacillus pseudofirmus* | CP001878 |
|  |  |  |  | bmyc | *Bacillus pseudomycoides* 219298 | CP007626 |
|  |  |  |  | bpum | *Bacillus pumilus* MTCC B6033 | CP007436 |
|  |  |  |  | bpu | *Bacillus pumilus* SAFR-032 | CP000813 |
|  |  |  |  | bpus | *Bacillus pumilus* SH-B9 | CP011007 |
|  |  |  |  | bsj | *Bacillus simplex* | CP011008 |
|  |  |  |  | bsm | *Bacillus smithii* | CP012024 |
|  |  |  |  | baci | *Bacillus sp.* 1NLA3E | CP005586 |
|  |  |  |  | bacb | *Bacillus sp.* BH072 | CP009938 |
|  |  |  |  | bacl | *Bacillus sp.* BS34A | LN680001 |
|  |  |  |  | bjs | *Bacillus sp.* JS | CP003492 |
|  |  |  |  | balm | *Bacillus sp.* LM 4-2 | CP011101 |
|  |  |  |  | baco | *Bacillus sp.* OxB-1 | AP013294 |
|  |  |  |  | bacp | *Bacillus sp.* Pc3 | CP010406 |
|  |  |  |  | bacs | *Bacillus sp.* SDLI1 | CP013950 |
|  |  |  |  | bacw | *Bacillus sp.* WP8 | CP010075 |
|  |  |  |  | bsg | *Bacillus sp.* X1(2014) | CP008855 |
|  |  |  |  | bacy | *Bacillus sp.* YP1 | CP010014 |
|  |  |  |  | bsn | *Bacillus subtilis* BSn5 | CP002468 |
|  |  |  |  | bsp | *Bacillus subtilis* PY79 | CP006881 |
|  |  |  |  | bsq | *Bacillus subtilis* QB928 | CP003783 |
|  |  |  |  | bso | *Bacillus subtilis* subsp*. natto* BEST195 | AP011541 |
|  |  |  |  | bst | *Bacillus subtilis* subsp*. spizizenii* TU-B-10 | CP002905 |
|  |  |  |  | bss | *Bacillus subtilis* subsp. *spizizenii* W23 | CP002183 |
|  |  |  |  | bsu | *Bacillus subtilis* subsp. *subtilis 1*68 | (NC_000964) |
|  |  |  |  | bsh | *Bacillus subtilis* subsp. *subtilis* 6051-HGW | CP003329 |
|  |  |  |  | bsut | *Bacillus subtilis* subsp. *subtilis* AG1839 | CP008698 |
|  |  |  |  | bsy | *Bacillus subtilis* subsp. *subtilis* BAB-1 | CP004405 |
|  |  |  |  | bsl | *Bacillus subtilis* subsp. *subtilis* BSP1 | CP003695 |
|  |  |  |  | bsul | *Bacillus subtilis* subsp. *subtilis* JH642 | CP007800 |
|  |  |  |  | bsus | *Bacillus subtilis* subsp. *subtilis* OH 131.1 | CP007409 |
|  |  |  |  | bsr | *Bacillus subtilis* subsp. s*ubtilis* RO-NN-1 | CP002906 |
|  |  |  |  | bsx | *Bacillus subtilis* XF-1 | CP004019 |
|  |  |  |  | btl | *Bacillus thuringiensis* Al Hakam | CP000485 |
|  |  |  |  | btb | *Bacillus thuringiensis* BMB171 | CP001903 |
|  |  |  |  | btg | *Bacillus thuringiensis* Bt407 | CP003687 |
|  |  |  |  | btw | *Bacillus thuringiensis* HD1011 | CP009335 |
|  |  |  |  | bti | *Bacillus thuringiensis* HD-771 | CP003752 |
|  |  |  |  | btn | *Bacillus thuringiensis* HD-789 | CP003763 |
|  |  |  |  | btm | *Bacillus thuringiensis* MC28 | CP003687 |
|  |  |  |  | btc | *Bacillus thuringiensis serovar chinensis* CT-43 | CP001907 |
|  |  |  |  | btf | *Bacillus thuringiensis serovar finitimus* YBT-020 | CP002508 |
|  |  |  |  | btk | *Bacillus thuringiensis serovar konkukian* 97-27 | (NC_005957) |
|  |  |  |  | bthi | *Bacillus thuringiensis serovar kurstaki* HD-1 | CP004870 |
|  |  |  |  | btt | *Bacillus thuringiensis serovar kurstaki* HD73 | CP004069 |
|  |  |  |  | bthr | *Bacillus thuringiensis serovar kurstaki* YBT-1520 | CP004858 |
|  |  |  |  | btht | *Bacillus thuringiensis serovar thuringiensis* IS5056 | CP004123 |
|  |  |  |  | bthu | *Bacillus thuringiensis* YBT*-*1518 | CP005935 |
|  |  |  |  | bthy | *Bacillus thuringiensis* YWC2-8 | CP013055 |
|  |  |  |  | bty | *Bacillus toyonensis* | CP006863 |
|  |  |  |  | bvm | *Bacillus vallismortis* | CP020893 |
|  |  |  |  | bmp | *Bacillus velezensis* | CP003838 |
|  |  |  |  | bamp | *Bacillus velezensis* AS43.3 | CP003838 |
|  |  |  |  | baq | *Bacillus velezensis* CAU B946 | HE617159 |
|  |  |  |  | bay | *Bacillus velezensis* FZB42 | CP000560 |
|  |  |  |  | bamb | *Bacillus velezensis* NAU-B3 | HG514499 |
|  |  |  |  | bamy | *Bacillus velezensis* SQR9 | CP006890 |
|  |  |  |  | bamt | *Bacillus velezensis TrigoCor*1448 | CP007244 |
|  |  |  |  | bama | *Bacillus velezensis* UCMB5033 | HG328253 |
|  |  |  |  | baml | *Bacillus velezensis* UCMB5036 | HF563562 |
|  |  |  |  | bamn | *Bacillus velezensis* UCMB5113 | HG328254 |
|  |  |  |  | bya | *Bacillus velezensis* YAU B9601-Y2 | HE774679 |
|  |  |  |  | bwh | *Bacillus weihaiensis* | CP016020 |
|  |  |  |  | bxi | *Bacillus xiamenensis* | CP017786 |
| *Lactobacillus* | 93 | 0 | 93 | lae | *Lactobacillus acetotolerans* | AP014808 |
|  |  |  |  | laca | *Lactobacillus acidipiscis* | LT630287 |
|  |  |  |  | lai | *Lactobacillus acidophilus* 30SC | CP002559 |
|  |  |  |  | laf | *Lactobacillus acidophilus* FSI4 | CP010432 |
|  |  |  |  | lad | *Lactobacillus acidophilus* La-14 | CP005926 |
|  |  |  |  | lac | *Lactobacillus acidophilus* NCFM | CP000033(NC_006814) |
|  |  |  |  | lagl | *Lactobacillus agilis* | CP016766 |
|  |  |  |  | lali | *Lactobacillus alimentarius* | CP018867 |
|  |  |  |  | lalw | *Lactobacillus allii* | CP019323 |
|  |  |  |  | lamy | *Lactobacillus amylolyticus* | CP020457 |
|  |  |  |  | lah | *Lactobacillus amylophilus* | CP018888 |
|  |  |  |  | lam | *Lactobacillus amylovorus* GRL 1112 | CP002338 |
|  |  |  |  | lay | *Lactobacillus amylovorus* GRL1118 | CP002609 |
|  |  |  |  | lbt | *Lactobacillus backii* | CP014623 |
|  |  |  |  | lbr | *Lactobacillus brevis* ATCC 367 | CP000416 |
|  |  |  |  | lbk | *Lactobacillus brevis* KB290 | AP012167 |
|  |  |  |  | lbn | *Lactobacillus buchneri* CD034 | CP003043 |
|  |  |  |  | lbh | *Lactobacillus buchneri* NRRL B-30929 | CP002652 |
|  |  |  |  | lcx | *Lactobacillus casei* 12A | CP006690 |
|  |  |  |  | lcs | *Lactobacillus casei* BD-II | CP002618 |
|  |  |  |  | lcb | *Lactobacillus casei* BL23 | FM177140 |
|  |  |  |  | lce | *Lactobacillus casei* LC2W | CP002616 |
|  |  |  |  | lcl | *Lactobacillus casei* LOCK919 | CP005486 |
|  |  |  |  | lcw | *Lactobacillus casei* W56 | HE970764 |
|  |  |  |  | lcy | *Lactobacillus coryniformis* | CP017697 |
|  |  |  |  | lcr | *Lactobacillus crispatus* | FN692037 |
|  |  |  |  | lct | *Lactobacillus crustorum* | CP017996 |
|  |  |  |  | lcu | *Lactobacillus curieae* | CP018906 |
|  |  |  |  | lcv | *Lactobacillus curvatus* | CP016028 |
|  |  |  |  | ldl | *Lactobacillus delbrueckii* subsp. *bulgaricus* 2038 | CP000156 |
|  |  |  |  | ldb | *Lactobacillus delbrueckii* subsp. *bulgaricus* ATCC 11842 | CR954253 |
|  |  |  |  | lbu | *Lactobacillus delbrueckii* subsp. *bulgaricus* ATCC BAA-365 | CP000412 |
|  |  |  |  | lde | *Lactobacillus delbrueckii* subsp. *bulgaricus* ND02 | CP002341 |
|  |  |  |  | lfr | *Lactobacillus fermentum* CECT 5716 | CP002033 |
|  |  |  |  | lff | *Lactobacillus fermentum* F-6 | CP005958 |
|  |  |  |  | lfe | *Lactobacillus fermentum* IFO 3956 | AP008937 |
|  |  |  |  | lgl | *Lactobacillus gallinarum* | CP012890 |
|  |  |  |  | lga | *Lactobacillus gasseri* ATCC 33323 | CP000413 |
|  |  |  |  | lgn | *Lactobacillus ginsenosidimutans* | CP012034 |
|  |  |  |  | lhi | *Lactobacillus heilongjiangensis* | CP012559 |
|  |  |  |  | lhv | *Lactobacillus helveticus* CNRZ32 | CP002081 |
|  |  |  |  | lhe | *Lactobacillus helveticus* DPC 4571 | CP000517 |
|  |  |  |  | lhl | *Lactobacillus helveticus* H10 | CP002429 |
|  |  |  |  | lhh | *Lactobacillus helveticus* H9 | CP002427 |
|  |  |  |  | lhd | *Lactobacillus helveticus* KLDS1.8701 | CP009907 |
|  |  |  |  | lhr | *Lactobacillus helveticus* R0052 | CP003799 |
|  |  |  |  | lho | *Lactobacillus hokkaidonensis* | AP014680 |
|  |  |  |  | lje | *Lactobacillus jensenii* | CP018809 |
|  |  |  |  | ljh | *Lactobacillus johnsonii* DPC 6026 | CP002464 |
|  |  |  |  | ljf | *Lactobacillus johnsonii* FI9785 | FN298497 |
|  |  |  |  | ljn | *Lactobacillus johnsonii* N6.2 | CP006811 |
|  |  |  |  | ljo | *Lactobacillus johnsonii* NCC 533 | AE017198 |
|  |  |  |  | lke | *Lactobacillus kefiranofaciens* | CP002764 |
|  |  |  |  | lko | *Lactobacillus koreensis* | CP012033 |
|  |  |  |  | lku | *Lactobacillus kunkeei* | CP012920 |
|  |  |  |  | lle | *Lactobacillus lindneri* | CP014907 |
|  |  |  |  | lmu | *Lactobacillus mucosae* | CP011013 |
|  |  |  |  | lor | *Lactobacillus oris* | CP014787 |
|  |  |  |  | lpar | *Lactobacillus parabuchneri* | CP018796 |
|  |  |  |  | lca | *Lactobacillus paracasei* ATCC 334 | CP000423(NC_008526) |
|  |  |  |  | lpq | *Lactobacillus paracasei* N1115 | CP007122 |
|  |  |  |  | lpi | *Lactobacillus paracasei* subsp. *paracasei* 8700:2 | CP002391 |
|  |  |  |  | lpap | *Lactobacillus paracasei* subsp. *paracasei* JCM 8130 | AP012541 |
|  |  |  |  | lcz | *Lactobacillus paracasei* Zhang | CP001084 |
|  |  |  |  | lpd | *Lactobacillus paracollinoides* | CP014915 |
|  |  |  |  | lpx | *Lactobacillus paraplantarum* | CP013130 |
|  |  |  |  | lpg | *Lactobacillus pentosus* | CP016491 |
|  |  |  |  | lpz | *Lactobacillus plantarum* 16 | CP006033 |
|  |  |  |  | lpb | *Lactobacillus plantarum* B21 | CP010528 |
|  |  |  |  | lpj | *Lactobacillus plantarum* JDM1 | CP001617 |
|  |  |  |  | lpr | *Lactobacillus plantarum* subsp. *plantarum* P-8 | CP005942 |
|  |  |  |  | lps | *Lactobacillus plantarum* subsp. *plantarum* ST-III |  |
|  |  |  |  | lpl | *Lactobacillus plantarum* WCFS1 | AL935263(NC_004567) |
|  |  |  |  | lpt | *Lactobacillus plantarum* ZJ316 | CP004082 |
|  |  |  |  | lre | *Lactobacillus reuteri* DSM 20016 | CP000705 |
|  |  |  |  | lrt | *Lactobacillus reuteri* I5007 | CP006011 |
|  |  |  |  | lrf | *Lactobacillus reuteri* JCM 1112 | AP007281 |
|  |  |  |  | lru | *Lactobacillus reuteri* SD2112 | CP002844 |
|  |  |  |  | lrr | *Lactobacillus reuteri* TD1 | CP006603 |
|  |  |  |  | lra | *Lactobacillus rhamnosus* ATCC 8530 | CP003094 |
|  |  |  |  | lrh | *Lactobacillus rhamnosus* GG | FM179322 |
|  |  |  |  | lrg | *Lactobacillus rhamnosus* GG | AP011548 |
|  |  |  |  | lrl | *Lactobacillus rhamnosus* Lc 705 | FM179323 |
|  |  |  |  | lro | *Lactobacillus rhamnosus* LOCK900 | CP005484 |
|  |  |  |  | lrc | *Lactobacillus rhamnosus* LOCK908 | CP005485 |
|  |  |  |  | lrm | *Lactobacillus ruminis* | CP003032 |
|  |  |  |  | lsa | *Lactobacillus sakei* | CR936503 |
|  |  |  |  | lsi | *Lactobacillus salivarius* CECT 5713 | CP002034 |
|  |  |  |  | lsj | *Lactobacillus salivarius* JCM1046 | CP007646 |
|  |  |  |  | lsl | *Lactobacillus salivarius* UCC118 | CP000233(NC_007929) |
|  |  |  |  | lsn | *Lactobacillus sanfranciscensis* | CP002461 |
|  |  |  |  | law | *Lactobacillus* sp. wkB8 | CP009531 |
|  |  |  |  | lzy | *Lactobacillus zymae* | LT854705 |
| *Staphylococcus* | 86 | 9 | 77 | sagq | *Staphylococcus agnetis* | CP009623 |
|  |  |  |  | suh | *Staphylococcus argenteus* | FR821777 |
|  |  |  |  | suy | *Staphylococcus aureus* 04-02981 (MRSA) | CP001844 |
|  |  |  |  | saub | *Staphylococcus aureus* 08BA02176 (LA-MRSA) | CP003808 |
|  |  |  |  | saud | *Staphylococcus aureus* 502A | CP007454 |
|  |  |  |  | saur | *Staphylococcus aureus* Bmb9393 (MRSA) | CP005288 |
|  |  |  |  | sauc | *Staphylococcus aureus* CA-347 (MRSA) | CP006044 |
|  |  |  |  | saum | *Staphylococcus aureus* M1 (MRSA) | HF937103 |
|  |  |  |  | sams | *Staphylococcus aureus* MS4 | CP009828 |
|  |  |  |  | sab | *Staphylococcus aureus* RF122 | AJ938182 |
|  |  |  |  | suz | *Staphylococcus aureus* subsp. *aureus* 11819-97 (CA-MRSA) | CP003194 |
|  |  |  |  | saua | *Staphylococcus aureus* subsp. *aureus* 55/2053 | CP002388 |
|  |  |  |  | saue | *Staphylococcus aureus* subsp. *aureus* 6850 (MSSA) | CP006706 |
|  |  |  |  | sud | *Staphylococcus aureus* subsp. *aureus* 71193 (MSSA) | CP003045 |
|  |  |  |  | saun | *Staphylococcus aureus* subsp. *aureus* CN1 (CA-MRSA) | CP003979 |
|  |  |  |  | sac | *Staphylococcus aureus* subsp. *aureus* COL (MRSA) | CP000046 |
|  |  |  |  | suc | *Staphylococcus aureus* subsp. *aureus* ECT-R 2 | FR714927 |
|  |  |  |  | sue | *Staphylococcus aureus* subsp. *aureus* ED133 | CP001996 |
|  |  |  |  | sad | *Staphylococcus aureus* subsp. *aureus* ED98 | CP001781 |
|  |  |  |  | sauf | *Staphylococcus aureus* subsp. *aureus* FDAARGOS_5 | CP007539 |
|  |  |  |  | sux | *Staphylococcus aureus* subsp. *aureus* HO 5096 0412 (MRSA) | HE681097 |
|  |  |  |  | sah | *Staphylococcus aureus* subsp. *aureus* JH1 (MRSA/VSSA) | CP000736 |
|  |  |  |  | saj | *Staphylococcus aureus* subsp. *aureus* JH9 (MRSA/VISA) | CP000703 |
|  |  |  |  | suk | *Staphylococcus aureus* subsp. *aureus* JKD6008 (MRSA/VISA) | CP002120 |
|  |  |  |  | suj | *Staphylococcus aureus* subsp. *aureus* JKD6159 (CA-MRSA) | CP002114 |
|  |  |  |  | suf | *Staphylococcus aureus* subsp. *aureus* LGA251 (MRSA) | FR821779 |
|  |  |  |  | suu | *Staphylococcus aureus* subsp. *aureus* M013 (CA-MRSA) | CP003166 |
|  |  |  |  | sar | *Staphylococcus aureus* subsp. *aureus* MRSA252 (MRSA) | BX571856 |
|  |  |  |  | sas | *Staphylococcus aureus* subsp. *aureus* MSSA476 (MSSA) | BX571857 |
|  |  |  |  | saw | *Staphylococcus aureus* subsp. *aureus Mu3* (MRSA/hetero-VISA) | AP009324 |
|  |  |  |  | sav | *Staphylococcus aureus subsp. aureus Mu50 (MRSA/VISA)* | BA000017 |
|  |  |  |  | sam | *Staphylococcus aureus* subsp. *aureus* MW2 (CA-MRSA) | BA000033 |
|  |  |  |  | sau | *Staphylococcus aureus* subsp. *aureus* N315 (MRSA/VSSA) | BA000018 |
|  |  |  |  | sao | *Staphylococcus aureus* subsp. *aureus* NCTC8325 | CP000253(NC_007795) |
|  |  |  |  | sae | *Staphylococcus aureus* subsp. *aureus* Newman | AP009351 |
|  |  |  |  | saug | *Staphylococcus aureus* subsp. *aureus* SA268 (CA-MRSA) | CP006630 |
|  |  |  |  | saus | *Staphylococcus aureus* subsp. *aureus* SA40 (CA-MRSA) | CP003604 |
|  |  |  |  | sauu | *Staphylococcus aureus* subsp. *aureus* SA957 (CA-MRSA) | CP003603 |
|  |  |  |  | saut | *Staphylococcus aureus* subsp. *aureus* ST228/10388 (MRSA) | HE579059 |
|  |  |  |  | sauj | *Staphylococcus aureus* subsp. *aureus* ST228/10497 (MRSA) | HE579061 |
|  |  |  |  | sauk | *Staphylococcus aureus* subsp. *aureus* ST228/15532 (MRSA) | HE579063 |
|  |  |  |  | sauq | *Staphylococcus aureus* subsp. *aureus* ST228/16035 (MRSA) | HE579065 |
|  |  |  |  | sauw | *Staphylococcus aureus* subsp. *aureus* ST228/16125 (MRSA) | HE579067 |
|  |  |  |  | saux | *Staphylococcus aureus* subsp. *aureus* ST228/18341 (MRSA) | HE579069 |
|  |  |  |  | sauv | *Staphylococcus aureus* subsp. *aureus* ST228/18412 (MRSA) | HE579071 |
|  |  |  |  | sauy | *Staphylococcus aureus* subsp. *aureus* ST228/18583 (MRSA) | HE579073 |
|  |  |  |  | sug | *Staphylococcus aureus* subsp. *aureus* ST398 (MRSA) | AM990992 |
|  |  |  |  | sut | *Staphylococcus aureus* subsp. *aureus* T0131 (MRSA) | CP002643 |
|  |  |  |  | suq | *Staphylococcus aureus* subsp. *aureus* TCH60 | CP002110 |
|  |  |  |  | suw | *Staphylococcus aureus* subsp. *aureus* TW20 (MRSA) | FN433596 |
|  |  |  |  | saa | *Staphylococcus aureus* subsp. *aureus* USA300_FPR3757 (CA-MRSA) | CP000255 |
|  |  |  |  | sax | *Staphylococcus aureus* subsp. *aureus* USA300_TCH1516 (CA-MRSA) | CP000730 |
|  |  |  |  | suv | *Staphylococcus aureus* subsp. *aureus* VC40 | CP003033 |
|  |  |  |  | sauz | *Staphylococcus aureus* subsp. *aureus* Z172 (MRSA/VISA) | CP006838 |
|  |  |  |  | saui | *Staphylococcus aureus* USA300-ISMMS1 (MRSA) | CP007176 |
|  |  |  |  | scap | *Staphylococcus capitis* | CP007601 |
|  |  |  |  | sca | *Staphylococcus carnosus* | AM295250 |
|  |  |  |  | scoh | *Staphylococcus cohnii* | CP019597 |
|  |  |  |  | scv | *Staphylococcus condimenti* | CP015114 |
|  |  |  |  | sep | *Staphylococcus epidermidis* ATCC 12228 | AE015929(NC_004461) |
|  |  |  |  | sepp | *Staphylococcus epidermidis* PM221 | HG813242 |
|  |  |  |  | ser | *Staphylococcus epidermidis* RP62A (MRSE) | CP000029 |
|  |  |  |  | seps | *Staphylococcus epidermidis* SEI | CP009046 |
|  |  |  |  | seqo | *Staphylococcus equorum* | CP013114 |
|  |  |  |  | sfq | *Staphylococcus felis* | CP027770 |
|  |  |  |  | sha | *Staphylococcus haemolyticus* JCSC1435 | AP006716 |
|  |  |  |  | shh | *Staphylococcus haemolyticus* Sh29/312/L2 | CP011116 |
|  |  |  |  | shu | *Staphylococcus hyicus* | CP008747 |
|  |  |  |  | skl | *Staphylococcus kloosii* | CP027846 |
|  |  |  |  | slg | *Staphylococcus lugdunensis* HKU09-01 | CP001837 |
|  |  |  |  | sln | *Staphylococcus lugdunensis* N920143 | FR870271 |
|  |  |  |  | slz | *Staphylococcus lutrae* | CP020773 |
|  |  |  |  | snl | *Staphylococcus nepalensis* | CP017460 |
|  |  |  |  | spas | *Staphylococcus pasteuri* | CP004014 |
|  |  |  |  | spet | *Staphylococcus pettenkoferi* | CP022096 |
|  |  |  |  | sdt | *Staphylococcus pseudintermedius* ED99 | CP002478 |
|  |  |  |  | ssd | *Staphylococcus pseudintermedius* HKU10-03 | CP002439 |
|  |  |  |  | ssp | *Staphylococcus saprophyticus* | AP008934 |
|  |  |  |  | ssch | *Staphylococcus schleiferi* 1360-13 | CP009470 |
|  |  |  |  | sscz | *Staphylococcus schleiferi* 2317-03 | CP010309 |
|  |  |  |  | sscu | *Staphylococcus sciuri* | CP022046 |
|  |  |  |  | ssif | *Staphylococcus simulans* | CP014016 |
|  |  |  |  | swa | *Staphylococcus warneri* | CP003668 |
|  |  |  |  | sxo | *Staphylococcus xylosus* C2a | LN554884 |
|  |  |  |  | sxy | *Staphylococcus xylosus* HKUOPL8 | CP007208 |
|  |  |  |  | sxl | *Staphylococcus xylosus* SMQ-121 | CP008724 |
| *Clostridium* | 52 | 18 | 34 | cace | *Clostridium aceticum* | CP009687 |
|  |  |  |  | cac | *Clostridium acetobutylicum* ATCC 824 | AE001437(NC_003030) |
|  |  |  |  | cae | *Clostridium acetobutylicum* DSM 1731 | CP002660 |
|  |  |  |  | cay | *Clostridium acetobutylicum* EA 2018 | CP002118 |
|  |  |  |  | carg | *Clostridium argentinense* | CP014176 |
|  |  |  |  | cah | *Clostridium autoethanogenum* | CP006763 |
|  |  |  |  | cbv | *Clostridium baratii* | CP006905 |
|  |  |  |  | cbz | *Clostridium beijerinckii* ATCC 35702 | CP006777 |
|  |  |  |  | cbei | *Clostridium beijerinckii* NCIMB 14988 | CP010086 |
|  |  |  |  | cbe | *Clostridium beijerinckii* NCIMB 8052 | CP000721 |
|  |  |  |  | clt | *Clostridium bornimense* | HG917868 |
|  |  |  |  | cba | *Clostridium botulinum* A ATCC 19397 | CP000726 |
|  |  |  |  | cbo | *Clostridium botulinum* A ATCC 3502 | AM412317(NC_009495) |
|  |  |  |  | cbh | *Clostridium botulinum* A Hall | CP000727(NC_009698) |
|  |  |  |  | cby | *Clostridium botulinum* A2 | CP001581 |
|  |  |  |  | cbl | *Clostridium botulinum* A3 Loch Maree | CP000962 |
|  |  |  |  | cbk | *Clostridium botulinum* B Eklund 17B (NRP) | CP001056 |
|  |  |  |  | cbb | *Clostridium botulinum* B1 Okra | CP000939 |
|  |  |  |  | cbi | *Clostridium botulinum* Ba4 | CP001083 |
|  |  |  |  | cbn | *Clostridium botulinum* BKT015925 | CP002410 |
|  |  |  |  | cbt | *Clostridium botulinum* E3 | CP001078 |
|  |  |  |  | cbm | *Clostridium botulinum* F 230613 | CP002011 |
|  |  |  |  | cbf | *Clostridium botulinum* F Langeland | CP000728 |
|  |  |  |  | cbj | *Clostridium botulinum* H04402 065 | FR773526 |
|  |  |  |  | cbut | *Clostridium butyricum* | CP013252 |
|  |  |  |  | cck | *Clostridium carboxidivorans* | CP011803 |
|  |  |  |  | ccb | *Clostridium cellulovorans* | CP002160 |
|  |  |  |  | cchv | *Clostridium chauvoei* | CP018630 |
|  |  |  |  | cdrk | *Clostridium drakei* | CP020953 |
|  |  |  |  | ceu | *Clostridium estertheticum* | CP015756 |
|  |  |  |  | cfm | *Clostridium formicaceticum* | CP017603 |
|  |  |  |  | cia | *Clostridium isatidis* | CP016786 |
|  |  |  |  | ckl | *Clostridium kluyveri* DSM 555 | CP000673 |
|  |  |  |  | ckr | *Clostridium kluyveri* NBRC 12016 | AP009049 |
|  |  |  |  | clj | *Clostridium ljungdahlii* | CP001666 |
|  |  |  |  | cno | *Clostridium novyi* | CP000382 |
|  |  |  |  | cpas | *Clostridium pasteurianum* BC1 | CP003261 |
|  |  |  |  | cpae | *Clostridium pasteurianum* DSM 525 = ATCC 6013 | CP009268 |
|  |  |  |  | cpat | *Clostridium pasteurianum* DSM 525 = ATCC 6013 | CP009267 |
|  |  |  |  | cpe | *Clostridium perfringens* 13 | BA000016 |
|  |  |  |  | cpf | *Clostridium perfringens* ATCC 13124 | CP000246 |
|  |  |  |  | cpr | *Clostridium perfringens* SM101 | CP000312 |
|  |  |  |  | csb | *Clostridium saccharobutylicum* | CP006721 |
|  |  |  |  | csr | *Clostridium saccharoperbutylacetonicum* | CP004121 |
|  |  |  |  | csq | *Clostridium scatologenes* | CP009933 |
|  |  |  |  | clb | *Clostridium* sp. BNL1100 | CP003259 |
|  |  |  |  | cls | *Clostridium* sp. SY8519 | AP012212 |
|  |  |  |  | cld | *Clostridium sporogenes* | CP009225 |
|  |  |  |  | ctae | *Clostridium taeniosporum* | CP017253 |
|  |  |  |  | ctet | *Clostridium tetani* 12124569 | HG530135 |
|  |  |  |  | ctc | *Clostridium tetani* E88 | AE015927 |
|  |  |  |  | ctyk | *Clostridium tyrobutyricum* | CP014170 |
| *Listeria* | 46 | 0 | 46 | lin | *Listeria innocua* (serotype 6a) | AL592022 |
|  |  |  |  | liv | *Listeria ivanovii* subsp. *ivanovii* PAM 55 | FR687253 |
|  |  |  |  | lii | *Listeria ivanovii* subsp. *ivanovii* WSLC 3010 | CP009577 |
|  |  |  |  | lio | *Listeria ivanovii* subsp. *londoniensis* WSLC 30151 | CP009576 |
|  |  |  |  | lia | *Listeria ivanovii* subsp. *londoniensis* WSLC 30167 | CP009575 |
|  |  |  |  | liw | *Listeria ivanovii* WSLC3009 | CP007172 |
|  |  |  |  | lmp | *Listeria monocytogenes* 07PF0776 (serotype 4b) | CP003414 |
|  |  |  |  | lmn | *Listeria monocytogenes* 08-5578 (serotype 1/2a) | CP001602 |
|  |  |  |  | lmy | *Listeria monocytogenes* 08-5923 (serotype 1/2a) | CP001604 |
|  |  |  |  | lmt | *Listeria monocytogenes* 10403S (serotype 1/2a) | CP002002 |
|  |  |  |  | lmoq | *Listeria monocytogenes* 6179 (serotype 1/2a) | HG813249 |
|  |  |  |  | lmoa | *Listeria monocytogenes* ATCC 19117 (serotype 4d) | FR733643 |
|  |  |  |  | lmv | *Listeria monocytogenes* CFSAN006122 | CP007600 |
|  |  |  |  | lmc | *Listeria monocytogenes* CLIP 80459 (serotype 4b) | FM242711 |
|  |  |  |  | lmod | *Listeria monocytogenes* EGD (serotype 1/2a) | HG421741 |
|  |  |  |  | lmo | *Listeria monocytogenes* EGD-e (serotype 1/2a) | AL591824(NC_003210) |
|  |  |  |  | lmf | *Listeria monocytogenes* F2365 (serotype 4b) | AE017262 |
|  |  |  |  | lms | *Listeria monocytogenes* Finland 1998 | CP002004 |
|  |  |  |  | lmg | *Listeria monocytogenes* FSL R2-561 | CP002003 |
|  |  |  |  | lmh | *Listeria monocytogenes* HCC23 (serotype 4a) | CP001175 |
|  |  |  |  | lmj | *Listeria monocytogenes* J0161 | CP002001 |
|  |  |  |  | lmoz | *Listeria monocytogenes* J1-220 (serotype 4b) | CP006046 |
|  |  |  |  | lmoj | *Listeria monocytogenes* J1816 (serotype 4b) | CP006047 |
|  |  |  |  | lmol | *Listeria monocytogenes* L312 (serotype 4b) | FR733642 |
|  |  |  |  | lml | *Listeria monocytogenes* L99 (serotype 4a) | FM211688 |
|  |  |  |  | lmoe | *Listeria monocytogenes* La111 (serotype 1/2a) | HE999704 |
|  |  |  |  | lmom | *Listeria monocytogenes* Lm60 (serotype 1/2a) | CP009258 |
|  |  |  |  | lmq | *Listeria monocytogenes* M7 (serotype 4a) | CP002816 |
|  |  |  |  | lmob | *Listeria monocytogenes* N53-1 (serotype 1/2a) | HE999705 |
|  |  |  |  | lmok | *Listeria monocytogenes* NE dc2014 | CP007492 |
|  |  |  |  | lmr | *Listeria monocytogenes* R479a (serotype 1/2a) | HG813247 |
|  |  |  |  | lmog | *Listeria monocytogenes* serotype 4b LL195 | HF558398 |
|  |  |  |  | lmz | *Listeria monocytogenes* serotype 7 SLCC2482 | FR720325 |
|  |  |  |  | lmx | *Listeria monocytogenes* SLCC2372 (serotype 1/2c) | FR733648 |
|  |  |  |  | lmon | *Listeria monocytogenes* SLCC2376 (serotype 4c) | FR733651 |
|  |  |  |  | lmoo | *Listeria monocytogenes* SLCC2378 (serotype 4e) | FR733644 |
|  |  |  |  | lmoy | *Listeria monocytogenes* SLCC2479 (serotype 3c) | FR733649 |
|  |  |  |  | lmot | *Listeria monocytogenes* SLCC2540 (serotype 3b) | FR733645 |
|  |  |  |  | lmw | *Listeria monocytogenes* SLCC2755 (serotype 1/2b) | FR733646 |
|  |  |  |  | lmoc | *Listeria monocytogenes* SLCC5850 (serotype 1/2a) | FR733647 |
|  |  |  |  | lmos | *Listeria monocytogenes* SLCC7179 (serotype 3a) | FR733650 |
|  |  |  |  | lmow | *Listeria monocytogenes* WSLC1001 (serotype 1/2a) | CP007160 |
|  |  |  |  | lmox | *Listeria monocytogenes* WSLC1042 (serotype 4b) | CP007210 |
|  |  |  |  | lsg | *Listeria seeligeri* | FN557490 |
|  |  |  |  | lwi | *Listeria weihenstephanensis* | CP011102 |
|  |  |  |  | lwe | *Listeria welshimeri* | AM263198 |
| *Paenibacillus* | 43 | 22 | 21 | pbj | *Paenibacillus beijingensis* | CP003235 |
|  |  |  |  | pbd | *Paenibacillus borealis* | CP009285 |
|  |  |  |  | pbv | *Paenibacillus bovis* | CP013023 |
|  |  |  |  | pcx | *Paenibacillus crassostreae* | CP017770 |
|  |  |  |  | pdh | *Paenibacillus donghaensis* | CP021780 |
|  |  |  |  | pdu | *Paenibacillus durus* | CP009288 |
|  |  |  |  | pgm | *Paenibacillus graminis* | CP009287 |
|  |  |  |  | pib | *Paenibacillus ihbetae* | CP016809 |
|  |  |  |  | pkb | *Paenibacillus kribbensis* | CP020028 |
|  |  |  |  | plv | *Paenibacillus larvae* | CP003355 |
|  |  |  |  | plw | *Paenibacillus lautus* | CP032412 |
|  |  |  |  | pmq | *Paenibacillus mucilaginosus* 3016 | CP003235 |
|  |  |  |  | pmw | *Paenibacillus mucilaginosus* K02 | CP003422 |
|  |  |  |  | pms | *Paenibacillus mucilaginosus* KNP414 | CP002869 |
|  |  |  |  | pnp | *Paenibacillus naphthalenovorans* | CP013652 |
|  |  |  |  | pod | *Paenibacillus odorifer* | CP009428 |
|  |  |  |  | ppeo | *Paenibacillus peoriae* | CP011512 |
|  |  |  |  | ppol | *Paenibacillus polymyxa* CR1 | CP006941 |
|  |  |  |  | ppy | *Paenibacillus polymyxa* E681 | CP000154 |
|  |  |  |  | ppo | *Paenibacillus polymyxa* M1 | HE577054 |
|  |  |  |  | ppoy | *Paenibacillus polymyxa* Sb3-1 | CP010268 |
|  |  |  |  | ppm | *Paenibacillus polymyxa* SC2 | CP002213 |
|  |  |  |  | ppq | *Paenibacillus polymyxa* SQR-21 | CP006872 |
|  |  |  |  | pri | *Paenibacillus riograndensis* | LN831776 |
|  |  |  |  | psab | *Paenibacillus sabinae* | CP004078 |
|  |  |  |  | pow | *Paenibacillus* sp. 32O-W | CP013653 |
|  |  |  |  | paeh | *Paenibacillus* sp. FSL H7-0357 | CP009241 |
|  |  |  |  | paej | *Paenibacillus* sp. FSL H7-0737 | CP009279 |
|  |  |  |  | paen | *Paenibacillus* sp. FSL P4-0081 | CP009280 |
|  |  |  |  | paef | *Paenibacillus* sp. FSL R5-0345 | CP009281 |
|  |  |  |  | paeq | *Paenibacillus* sp. FSL R5-0912 | CP009282 |
|  |  |  |  | paea | *Paenibacillus* sp. FSL R7-0273 | CP009283 |
|  |  |  |  | paee | *Paenibacillus* sp. FSL R7-0331 | CP009284 |
|  |  |  |  | paih | *Paenibacillus* sp. IHB B 3084 | CP013203 |
|  |  |  |  | pxl | *Paenibacillus* sp. IHBB 10380 | CP010976 |
|  |  |  |  | pjd | *Paenibacillus* sp. JDR-2 | CP001656 |
|  |  |  |  | gym | *Paenibacillus* sp. Y412MC10 | CP001793 |
|  |  |  |  | pste | *Paenibacillus stellifer* | CP009286 |
|  |  |  |  | pswu | *Paenibacillus swuensis* | CP011388 |
|  |  |  |  | pta | *Paenibacillus terrae* | CP003107 |
|  |  |  |  | pvo | *Paenibacillus vortex* |  |
|  |  |  |  | pxl | *Paenibacillus xylanexedens* | CP018620 |
|  |  |  |  | pyg | *Paenibacillus yonginensis* | CP014167 |
| *Enterococcus* | 21 | 8 | 13 | ecas | *Enterococcus casseliflavus* | CP004856 |
|  |  |  |  | edu | *Enterococcus durans* | CP012384 |
|  |  |  |  | efl | *Enterococcus faecalis* 62 | CP002491 |
|  |  |  |  | ene | *Enterococcus faecalis* 7L76 | FP929058 |
|  |  |  |  | efq | *Enterococcus faecalis* ATCC 29212 | CP008816 |
|  |  |  |  | efd | *Enterococcus faecalis* D32 | CP003726 |
|  |  |  |  | efn | *Enterococcus faecalis* DENG1 | CP004081 |
|  |  |  |  | efi | *Enterococcus faecalis* OG1RF | CP002621 |
|  |  |  |  | efs | *Enterococcus faecalis* Symbioflor 1 | HF558530 |
|  |  |  |  | efa | *Enterococcus faecalis* V583 | AE016830(NC_004668) |
|  |  |  |  | efm | *Enterococcus faecium* ATCC 8459 = NRRL B-2354 | CP004063 |
|  |  |  |  | efc | *Enterococcus faecium* Aus0004 | CP003351 |
|  |  |  |  | efau | *Enterococcus faecium* Aus0085 | CP006620 |
|  |  |  |  | efu | *Enterococcus faecium* DO | CP003583(NC_017960) |
|  |  |  |  | eft | *Enterococcus faecium* T110 | CP006030 |
|  |  |  |  | ega | *Enterococcus gallinarum* | CP014067 |
|  |  |  |  | egv | *Enterococcus gilvus* | CP030932 |
|  |  |  |  | ehr | *Enterococcus hirae* | CP003504 |
|  |  |  |  | emu | *Enterococcus mundtii* | AP013036 |
|  |  |  |  | ess | *Enterococcus silesiacus* | CP013614 |
|  |  |  |  | eth | *Enterococcus thailandicus* | CP023074 |
| *Geobacillus* | 18 | 0 | 18 | gjf | *Geobacillus genomosp*. 3 | CP006254 |
|  |  |  |  | gka | *Geobacillus kaustophilus* | BA000043 |
|  |  |  |  | gli | *Geobacillus lituanicus* | CP017692 |
|  |  |  |  | gea | *Geobacillus* sp. 12AMOR1 | CP011832 |
|  |  |  |  | gct | *Geobacillus* sp. C56-T3 | CP002050 |
|  |  |  |  | ggh | *Geobacillus* sp. GHH01 | CP004008 |
|  |  |  |  | gej | *Geobacillus* sp. JS12 | CP014749 |
|  |  |  |  | gel | *Geobacillus* sp. LC300 | CP008903 |
|  |  |  |  | gwc | *Geobacillus* sp. WCH70 | CP001638 |
|  |  |  |  | gmc | *Geobacillus* sp. Y4.1MC1 | CP002293 |
|  |  |  |  | gya | *Geobacillus* sp. Y412MC52 | CP002442 |
|  |  |  |  | gyc | *Geobacillus* sp. Y412MC61 | CP001794 |
|  |  |  |  | gse | *Geobacillus stearothermophilus* | CP008934 |
|  |  |  |  | gsr | *Geobacillus subterraneus* | CP014342 |
|  |  |  |  | gtm | *Geobacillus thermocatenulatus* | CP018058 |
|  |  |  |  | gtn | *Geobacillus thermodenitrificans* | CP000557 |
|  |  |  |  | gte | *Geobacillus thermoleovorans* CCB_US3_UF5 | CP003125 |
|  |  |  |  | gtk | *Geobacillus thermoleovorans* KCTC 3570 | CP014335 |
| *Lactococcus* | 18 | 0 | 18 | lgr | *Lactococcus garvieae* ATCC 49156 | AP009332 |
|  |  |  |  | lgv | *Lactococcus garvieae* Lg2 | AP009333 |
|  |  |  |  | llj | *Lactococcus lactis* AI06 | CP009472 |
|  |  |  |  | llr | *Lactococcus lactis* subsp. *cremoris* A76 | CP003132 |
|  |  |  |  | llw | *Lactococcus lactis* subsp. *cremoris* KW2 | CP004884 |
|  |  |  |  | llm | *Lactococcus lactis* subsp. *cremoris* MG1363 | AM406671 |
|  |  |  |  | lln | *Lactococcus lactis* subsp. *cremoris* NZ9000 | CP002094 |
|  |  |  |  | llc | *Lactococcus lactis* subsp. *cremoris* SK11 | CP000425 |
|  |  |  |  | lli | *Lactococcus lactis* subsp. *cremoris* UC509.9 | CP003157 |
|  |  |  |  | llt | *Lactococcus lactis* subsp. *lactis* CV56 | CP002365 |
|  |  |  |  | lla | *Lactococcus lactis* subsp. *lactis* Il1403 | AE005176(NC_002662) |
|  |  |  |  | lls | *Lactococcus lactis* subsp. *lactis* IO-1 | AP012281 |
|  |  |  |  | llk | *Lactococcus lactis* subsp. *lactis* KF147 | CP001834 |
|  |  |  |  | lld | *Lactococcus lactis* subsp. *lactis* KLDS 4.0325 | CP006766 |
|  |  |  |  | llx | *Lactococcus lactis* subsp. *lactis* NCDO 2118 | CP009054 |
|  |  |  |  | lpk | *Lactococcus piscium* | LN774769 |
|  |  |  |  | lrn | *Lactococcus raffinolactis* | CP023392 |
|  |  |  |  | lact | *Lactococcus* sp. 1JSPR-7 | CP032627 |
| *Leuconostoc* | 12 | 0 | 12 | lcn | *Leuconostoc carnosum* | CP003851 |
|  |  |  |  | lci | *Leuconostoc citreum* | DQ489736 |
|  |  |  |  | lgc | *Leuconostoc garlicum* | CP016329 |
|  |  |  |  | lge | *Leuconostoc gelidum* JB7 | CP003839 |
|  |  |  |  | lgs | *Leuconostoc gelidum* subsp. *gasicomitatum* | FN822744 |
|  |  |  |  | lki | *Leuconostoc kimchii* | CP001758 |
|  |  |  |  | llf | *Leuconostoc lactis* | CP016598 |
|  |  |  |  | lmk | *Leuconostoc mesenteroides* KFRI-MG | CP000574 |
|  |  |  |  | lme | *Leuconostoc mesenteroides* subsp. *mesenteroides* ATCC 8293 | CP000414 |
|  |  |  |  | lmm | *Leuconostoc mesenteroides* subsp. *mesenteroides* J18 | CP003101 |
|  |  |  |  | lsu | *Leuconostoc mesenteroides* subsp. *suionicum* | CP015247 |
|  |  |  |  | lec | *Leuconostoc* sp. C2 | CP002898 |
| *Planococcus* | 11 | 6 | 5 | pana | *Planococcus antarcticus* | CP016534 |
|  |  |  |  | pdg | *Planococcus donghaensis* | CP016543 |
|  |  |  |  | pfae | *Planococcus faecalis* | CP019401 |
|  |  |  |  | phc | *Planococcus halocryophilus* | CP016537 |
|  |  |  |  | pku | *Planococcus kocurii* | CP013661 |
|  |  |  |  | pmar | *Planococcus maritimus* | CP019640 |
|  |  |  |  | ppla | *Planococcus plakortidis* | CP016539 |
|  |  |  |  | prt | *Planococcus rifietoensis* | CP013659 |
|  |  |  |  | plx | *Planococcus* sp. MB-3u-03 | CP025135 |
|  |  |  |  | pln | *Planococcus* sp. PAMC 21323 | CP009129 |
|  |  |  |  | pll | *Planococcus versutus* | CP016540 |
| *Thermoanaerobacter* | 8 | 0 | 8 | tbo | *Thermoanaerobacter brockii* | CP002466 |
|  |  |  |  | tit | *Thermoanaerobacter italicus* | CP001936 |
|  |  |  |  | tki | *Thermoanaerobacter kivui* | CP009170 |
|  |  |  |  | tmt | *Thermoanaerobacter mathranii* | CP002032 |
|  |  |  |  | tpd | *Thermoanaerobacter pseudethanolicus* | CP000924 |
|  |  |  |  | thx | *Thermoanaerobacter* sp. X513 | CP002210 |
|  |  |  |  | tex | *Thermoanaerobacter* sp. X514 | CP000923 |
|  |  |  |  | twi | *Thermoanaerobacter wiegelii* | CP002991 |
| *Caldicellulosiruptor* | 8 | 0 | 8 | ate | *Caldicellulosiruptor bescii* | CP001393 |
|  |  |  |  | chd | *Caldicellulosiruptor hydrothermalis* | CP002219 |
|  |  |  |  | cki | *Caldicellulosiruptor kristjanssonii* | CP002326 |
|  |  |  |  | ckn | *Caldicellulosiruptor kronotskyensis* | CP002330 |
|  |  |  |  | clc | *Caldicellulosiruptor lactoaceticus* | CP003001 |
|  |  |  |  | cob | *Caldicellulosiruptor obsidiansis* | CP002164 |
|  |  |  |  | cow | *Caldicellulosiruptor owensensis* | CP002216 |
|  |  |  |  | csc | *Caldicellulosiruptor saccharolyticus* | CP000679 |
| *Weissella* | 7 | 1 | 6 | wce | *Weissella ceti* WS08 | CP007588 |
|  |  |  |  | wci | *Weissella ceti* WS105 | CP009224 |
|  |  |  |  | wct | *Weissella ceti* WS74 | CP009223 |
|  |  |  |  | wcb | *Weissella cibaria* | CP012873 |
|  |  |  |  | wjo | *Weissella jogaejeotgali* | CP014332 |
|  |  |  |  | wko | *Weissella koreensis* | CP002899 |
|  |  |  |  | wpa | *Weissella paramesenteroides* | CP023501 |
| *Aerococcus* | 7 | 0 | 7 | acg | *Aerococcus christensenii* | CP014159 |
|  |  |  |  | asan | *Aerococcus sanguinicola* | CP014160 |
|  |  |  |  | aur | *Aerococcus urinae* ACS-120-V-Col10a | CP002512 |
|  |  |  |  | aun | *Aerococcus urinae* CCUG36881 | CP014161 |
|  |  |  |  | aui | *Aerococcus urinaeequi* | CP013988 |
|  |  |  |  | auh | *Aerococcus urinaehominis* | CP014163 |
|  |  |  |  | avs | *Aerococcus viridans* | CP014164 |
| *Lysinibacillus* | 6 | 0 | 6 | lfu | *Lysinibacillus fusiformis* | CP010820 |
|  |  |  |  | lyz | *Lysinibacillus* sp. 2017 | CP029002 |
|  |  |  |  | lyb | *Lysinibacillus* sp. B2A1 | CP027224 |
|  |  |  |  | lys | *Lysinibacillus* sp. YS11 | CP026007 |
|  |  |  |  | lsp | *Lysinibacillus sphaericus* | CP000817 |
|  |  |  |  | lgy | *Lysinibacillus varians* | CP006837 |
| *Virgibacillus* | 6 | 4 | 2 | vpn | *Virgibacillus dokdonensis* | CP018622 |
|  |  |  |  | vhl | *Virgibacillus halodenitrificans* | CP017962 |
|  |  |  |  | vne | *Virgibacillus necropolis* | CP022437 |
|  |  |  |  | vil | *Virgibacillus phasianinus* | CP022315 |
|  |  |  |  | vig | *Virgibacillus* sp. 6R | CP017762 |
|  |  |  |  | vir | *Virgibacillus* sp. SK37 | CP007161 |
| *Pediococcus* | 6 | 0 | 6 | paci | *Pediococcus acidilactici* | CP015206 |
|  |  |  |  | pce | *Pediococcus claussenii* | CP003137 |
|  |  |  |  | pdm | *Pediococcus damnosus* | CP012288 |
|  |  |  |  | pio | *Pediococcus inopinatus* | CP019981 |
|  |  |  |  | ppe | *Pediococcus pentosaceus* ATCC 25745 | CP000422 |
|  |  |  |  | ppen | *Pediococcus pentosaceus* SL4 | CP006854 |
| *Anoxybacillus* | 5 | 1 | 4 | aamy | *Anoxybacillus amylolyticus* | CP015438 |
|  |  |  |  | aft | *Anoxybacillus flavithermus* | CP000922 |
|  |  |  |  | agn | *Anoxybacillus gonensis* | CP012152 |
|  |  |  |  | anm | *Anoxybacillus* sp. B2M1 | CP015435 |
|  |  |  |  | anl | *Anoxybacillus* sp. B7M1 | CP015436 |
| *Exiguobacterium* | 5 | 1 | 4 | ean | *Exiguobacterium antarcticum* | CP003063 |
|  |  |  |  | esi | *Exiguobacterium sibiricum* | CP001022 |
|  |  |  |  | eat | *Exiguobacterium* sp. AT1b | CP001615 |
|  |  |  |  | exm | *Exiguobacterium* sp. MH3 | CP006866 |
|  |  |  |  | exu | *Exiguobacterium* sp. U13-1 | CP015731 |
| *Sporosarcina* | 5 | 3 | 2 | spsy | *Sporosarcina psychrophila* | CP014616 |
|  |  |  |  | spor | *Sporosarcina* sp. P33 | CP015027 |
|  |  |  |  | spop | *Sporosarcina* sp. P37 | CP015349 |
|  |  |  |  | spos | *Sporosarcina* sp. PTS2304 | CP031230 |
|  |  |  |  | sure | *Sporosarcina ureae* | CP015348 |
| *Carnobacterium* | 5 | 1 | 4 | cdj | *Carnobacterium divergens* | CP016843 |
|  |  |  |  | caw | *Carnobacterium inhibens* | CP006812 |
|  |  |  |  | cml | *Carnobacterium maltaromaticum* | HE999757 |
|  |  |  |  | crn | *Carnobacterium* sp. 17-4 | CP002563 |
|  |  |  |  | carc | *Carnobacterium* sp. CP1 | CP010796 |
| *Lachnoclostridium* | 5 | 2 | 3 | cpy | *Lachnoclostridium phytofermentans* | CP000885 |
|  |  |  |  | lacy | *Lachnoclostridium* sp. YL32 | CP015399 |
|  |  |  |  | csh | *Clostridium saccharolyticum* WM1 | CP002109 |
|  |  |  |  | cso | *Clostridium cf. saccharolyticum* K10 | FP929037 |
|  |  |  |  | cbol | *Clostridium bolteae* | CP022464 |
| *Clostridioides* | 5 | 0 | 5 | cdf | *Clostridioides difficile* 630 | AM180355(NC_009089) |
|  |  |  |  | pdc | *Clostridioides difficile* 630 | CP010905 |
|  |  |  |  | pdf | *Clostridioides difficile* 630Derm | LN614756 |
|  |  |  |  | cdc | *Clostridioides difficile* CD196 | FN538970 |
|  |  |  |  | cdl | *Clostridioides difficile* R20291 | FN545816 |
| *Desulfitobacterium* | 5 | 0 | 5 | ddh | *Desulfitobacterium dehalogenans* | CP003348 |
|  |  |  |  | ddl | *Desulfitobacterium dichloroeliminans* | CP003344 |
|  |  |  |  | dhd | *Desulfitobacterium hafniense* DCB-2 | CP001336 |
|  |  |  |  | dsy | *Desulfitobacterium hafniense* Y51 | AP008230 |
|  |  |  |  | dmt | *Desulfitobacterium metallireducens* | CP007032 |
| *Selenomonas* | 5 | 0 | 5 | sri | *Selenomonas ruminantium* | AP012292 |
|  |  |  |  | selo | *Selenomonas* sp. oral taxon 136 | CP014240 |
|  |  |  |  | sele | *Selenomonas* sp. oral taxon 478 | CP012071 |
|  |  |  |  | selt | *Selenomonas* sp. oral taxon 920 | CP017043 |
|  |  |  |  | ssg | *Selenomonas sputigena* | CP002637 |
| *Ruminococcus* | 5 | 0 | 5 | ral | *Ruminococcus albus* | CP002403 |
|  |  |  |  | rus | *Ruminococcus bicirculans* | HF545616 |
|  |  |  |  | rch | *Ruminococcus champanellensis* | FP929052 |
|  |  |  |  | rum | *Ruminococcus* sp. SR1/5 | FP929053 |
|  |  |  |  | rto | *Ruminococcus torques* | FP929055 |
| *Arthromitus* | 4 | 0 | 4 | asf | *Candidatus Arthromitus* sp. SFB-mouse-Japan | AP012202 |
|  |  |  |  | aso | *Candidatus Arthromitus* sp. SFB-mouse-NL | CP008713 |
|  |  |  |  | asm | *Candidatus Arthromitus* sp. SFB-mouse-Yit | AP012209 |
|  |  |  |  | asb | *Candidatus Arthromitus* sp. SFB-rat-Yit | AP012210 |
| *Blautia* | 4 | 0 | 4 | bhan | *Blautia hansenii* | CP022413 |
|  |  |  |  | rob | *Blautia obeum* | FP929054 |
|  |  |  |  | blau | *Blautia* sp. N6H1-15 | CP030280 |
|  |  |  |  | byl | *Blautia* sp. YL58 | CP015405 |
| *Hungateiclostridium* | 4 | 0 | 4 | ccl | *Hungateiclostridium clariflavum* | CP003065 |
|  |  |  |  | hsc | *Hungateiclostridium saccincola* | CP025197 |
|  |  |  |  | cth | *Hungateiclostridium thermocellum* ATCC 27405 | CP000568 |
|  |  |  |  | ctx | *Hungateiclostridium thermocellum DSM 1313* | CP002416 |
| Unclassified *Ruminococcaceae* | 4 | 1 | 3 | rbp | *Ruminococcaceae bacterium* CPB6 | CP020705 |
|  |  |  |  | esr | *Eubacterium siraeum* V10Sc8a | FP929059 |
|  |  |  |  | esu | *Eubacterium siraeum* 70/3 | FP929044 |
|  |  |  |  | ccel | *Clostridium cellulosi* | LM995447 |
| Unclassified *Lachnospiraceae* | 4 | 0 | 4 | lbw | *Lachnospiraceae bacterium* oral taxon 500 | CP027241 |
|  |  |  |  | ere | *Eubacterium rectale* ATCC 33656 | CP001107 |
|  |  |  |  | ert | *Eubacterium rectale* DSM 17629 | FP929042 |
|  |  |  |  | era | *Eubacterium rectale* M104/1 | FP929043 |
| *Desulfotomaculum* | 4 | 0 | 4 | dfg | *Desulfotomaculum ferrireducens* | CP019698 |
|  |  |  |  | dca | *Desulfotomaculum nigrificans* | CP002736 |
|  |  |  |  | drm | *Desulfotomaculum reducens* | CP000612 |
|  |  |  |  | dru | *Desulfotomaculum ruminis* | CP002780 |
| Unclassified *Clostridiales* | 4 | 0 | 4 | cbar | *Clostridiales bacterium* 70B-A | LR130778 |
|  |  |  |  | bprm | *Butyrate-producing bacterium* SM4/1 | FP929060 |
|  |  |  |  | bprs | *Butyrate-producing bacterium* SS3/4 | FP929062 |
|  |  |  |  | euu | *Eubacterium sulci* | CP012068 |
| *Thermoanaerobacterium* | 4 | 0 | 4 | tsh | *Thermoanaerobacterium saccharolyticum* | CP003184 |
|  |  |  |  | ttm | *Thermoanaerobacterium thermosaccharolyticum* DSM 571 | CP002171 |
|  |  |  |  | tto | *Thermoanaerobacterium thermosaccharolyticum* M0795 | CP003066 |
|  |  |  |  | txy | *Thermoanaerobacterium xylanolyticum* | CP002739 |
| *Veillonella* | 4 | 0 | 4 | vat | *Veillonella atypica* | CP020566 |
|  |  |  |  | vdn | *Veillonella dispar* | LR134375 |
|  |  |  |  | vpr | *Veillonella parvula* | CP001820 |
|  |  |  |  | vrm | *Veillonella rodentium* | LT906470 |
| *Erysipelothrix* | 4 | 0 | 4 | erl | *Erysipelothrix larvae* | CP013213 |
|  |  |  |  | erh | *Erysipelothrix rhusiopathiae* Fujisawa | AP012027 |
|  |  |  |  | ers | *Erysipelothrix rhusiopathiae* SY1027 | CP005079 |
|  |  |  |  | eri | *Erysipelothrix* sp. 15TAL0474 | CP034234 |
| *Macrococcus* | 3 | 2 | 1 | mcak | *Macrococcus canis* | CP021059 |
|  |  |  |  | mcl | *Macrococcus caseolyticus* | AP009484 |
|  |  |  |  | macr | *Macrococcus* sp. IME1552 | CP017156 |
| *Gemella* | 3 | 0 | 3 | gmo | *Gemella morbillorum* | LS483440 |
|  |  |  |  | geq | *Gemella* sp. ND 6198 | CP022615 |
|  |  |  |  | got | *Gemella* sp. oral taxon 928 | CP014233 |
| *Brevibacillus* | 3 | 2 | 1 | bbe | *Brevibacillus brevis* | AP008955 |
|  |  |  |  | bfm | *Brevibacillus formosus* | CP018145 |
|  |  |  |  | blr | *Brevibacillus laterosporus* | CP007806 |
| *Solibacillus* | 3 | 2 | 1 | ssil | *Solibacillus silvestris* DSM 12223 | CP014609 |
|  |  |  |  | siv | *Solibacillus silvestris* StLB046 | AP012157 |
|  |  |  |  | sob | *Solibacillus* sp. R5-41 | CP024123 |
| *Tetragenococcus* | 3 | 3 | 0 | thl | *Tetragenococcus halophilus* | AP012046 |
|  |  |  |  | tkr | *Tetragenococcus koreensis* | CP027786 |
|  |  |  |  | too | *Tetragenococcus osmophilus* | AP012046 |
| *Oenococcus* | 3 | 0 | 3 | ooe | *Oenococcus oeni* | CP000411 |
|  |  |  |  | osi | *Oenococcus sicerae* | CP029684 |
|  |  |  |  | oen | *Oenococcus* sp. UCMA 16435 | CP030868 |
| *Jeotgalibaca* | 3 | 2 | 1 | jda | *Jeotgalibaca dankookensis* | CP019728 |
|  |  |  |  | jeh | *Jeotgalibaca* sp. H21T32 | CP034465 |
|  |  |  |  | jep | *Jeotgalibaca* sp. PTS2502 | CP019433 |
| *Butyrivibrio* | 3 | 0 | 3 | bfi | *Butyrivibrio fibrisolvens* | FP929036 |
|  |  |  |  | bhu | *Butyrivibrio hungatei* | CP017831 |
|  |  |  |  | bpb | *Butyrivibrio proteoclasticus* | CP001810 |
| *Roseburia* | 3 | 0 | 3 | rho | *Roseburia hominis* | CP003040 |
|  |  |  |  | rim | *Roseburia intestinalis* M50/1 | FP929049 |
|  |  |  |  | rix | *Roseburia intestinalis* XB6B4 | FP929050 |
| *Desulfosporosinus* | 3 | 0 | 3 | dai | *Desulfosporosinus acidiphilus* | CP003639 |
|  |  |  |  | dmi | *Desulfosporosinus meridiei* | CP003629 |
|  |  |  |  | dor | *Desulfosporosinus orientis* | CP003108 |
| *Dehalobacter* | 3 | 0 | 3 | drs | *Dehalobacter restrictus* | CP007033 |
|  |  |  |  | dec | *Dehalobacter* sp. CF | CP003870 |
|  |  |  |  | ded | *Dehalobacter* sp. DCA | CP003869 |
| *Megasphaera* | 3 | 0 | 3 | med | *Megasphaera elsdenii* | HE576794 |
|  |  |  |  | mhw | *Megasphaera hexanoica* | CP011940 |
|  |  |  |  | meg | *Megasphaera* sp. AJH120 | CP029462 |
| *Oceanobacillus* | 2 | 2 | 0 | oih | *Oceanobacillus iheyensis* | BA000028 |
|  |  |  |  | ocn | *Oceanobacillus* sp. 160 | CP024848 |
| *Parageobacillus* | 2 | 0 | 2 | gth | *Parageobacillus thermoglucosidasius* C56-YS93 | CP002835 |
|  |  |  |  | ptl | *Parageobacillus thermoglucosidasius* DSM 2542 | CP012712 |
| *Halobacillus* | 2 | 2 | 0 | hhd | *Halobacillus halophilus* | HE717023 |
|  |  |  |  | hmn | *Halobacillus mangrovi* | CP020772 |
| *Fictibacillus* | 2 | 0 | 2 | far | *Fictibacillus arsenicus* | CP016761 |
|  |  |  |  | fpn | *Fictibacillus phosphorivorans* | CP015378 |
| *Aneurinibacillus* | 2 | 2 | 0 | asoc | *Aneurinibacillus soli* | AP017312 |
|  |  |  |  | anx | *Aneurinibacillus* sp. XH2 | CP014140 |
| *Alicyclobacillus* | 2 | 2 | 0 | aac | *Alicyclobacillus acidocaldarius* subsp. *acidocaldarius* DSM 446 | CP001727 |
|  |  |  |  | aad | *Alicyclobacillus acidocaldarius* subsp. *acidocaldarius* Tc-4-1 | CP002902 |
| *Kyrpidia* | 2 | 1 | 1 | kyr | *Kyrpidia spormannii* | CP024955 |
|  |  |  |  | bts | *Kyrpidia tusciae* | CP002017 |
| *Tumebacillus* | 2 | 0 | 2 | tab | *Tumebacillus algifaecis* | CP022657 |
|  |  |  |  | tum | *Tumebacillus avium* | CP021434 |
| *Melissococcus* | 2 | 0 | 2 | mps | *Melissococcus plutonius* ATCC 35311 | AP012200 |
|  |  |  |  | mpx | *Melissococcus plutonius* DAT561 | AP012282 |
| *Vagococcus* | 2 | 0 | 2 | vpi | *Vagococcus penaei* | CP019609 |
|  |  |  |  | vte | *Vagococcus teuberi* | CP017267 |
| *Alkaliphilus* | 2 | 0 | 2 | amt | *Alkaliphilus metalliredigens* | CP000724 |
|  |  |  |  | aoe | *Alkaliphilus oremlandii* | CP000853 |
| *Thermoclostridium* | 2 | 0 | 2 | css | *Thermoclostridium stercorarium* subsp. *stercorarium* DSM 8532 | CP004044 |
|  |  |  |  | csd | *Thermoclostridium stercorarium* subsp. *stercorarium* DSM 8532 | CP003992 |
| *Faecalibacterium* | 2 | 0 | 2 | fpr | *Faecalibacterium prausnitzii* L2-6 | FP929045 |
|  |  |  |  | fpa | *Faecalibacterium prausnitzii* SL3/3 | FP929046 |
| *Coprococcus* | 2 | 0 | 2 | cct | *Coprococcus catus* | FP929038 |
|  |  |  |  | coo | *Coprococcus* sp. ART55/1 | FP929039 |
| *Eubacterium* | 2 | 0 | 2 | eel | *Eubacterium eligens* | CP001104 |
|  |  |  |  | elm | *Eubacterium limosum* | CP002273 |
| *Sulfobacillus* | 2 | 2 | 0 | sap | *Sulfobacillus acidophilus* DSM 10332 | CP003179 |
|  |  |  |  | say | *Sulfobacillus acidophilus* TPY | CP002901 |
| *Tepidanaerobacter* | 2 | 0 | 2 | tep | *Tepidanaerobacter acetatoxydans* Re1 | CP002728 |
|  |  |  |  | tae | *Tepidanaerobacter acetatoxydans* Re1 | HF563609 |
| *Thermodesulfobium* | 2 | 0 | 2 | taci | *Thermodesulfobium acidiphilum* | CP020921 |
|  |  |  |  | tnr | *Thermodesulfobium narugense* | CP002690 |
| *Halanaerobium* | 2 | 0 | 2 | has | *Halanaerobium hydrogeniformans* | CP002304 |
|  |  |  |  | hpk | *Halanaerobium praevalens* | CP002175 |
| *Peptoniphilus* | 2 | 0 | 2 | ebm | *Peptoniphilus harei* | LR134524 |
|  |  |  |  | ped | *Peptoniphilus* sp. ING2-D1G | LM997412 |
| *Pelosinus* | 2 | 0 | 2 | pft | *Pelosinus fermentans* | CP010978 |
|  |  |  |  | puf | *Pelosinus* sp. UFO1 | CP008852 |
| *Acidaminococcus* | 2 | 0 | 2 | afn | *Acidaminococcus fermentans* | CP001859 |
|  |  |  |  | ain | *Acidaminococcus intestini* | CP003058 |
| Unclassified *Erysipelotrichaceae* | 2 | 0 | 2 | erb | *Erysipelotrichaceae bacterium* SG0102 | AP019309 |
|  |  |  |  | ebm | *Peptoniphilus harei* | LR134524 |
| *Amphibacillus* | 1 | 0 | 1 | axl | *Amphibacillus xylanus* | AP012050 |
| *Terribacillus* | 1 | 1 | 0 | tap | *Terribacillus goriensis* | CP008876 |
| *Lentibacillus* | 1 | 1 | 0 | lao | *Lentibacillus amyloliquefaciens* | CP013862 |
| *Salimicrobium* | 1 | 1 | 0 | sje | *Salimicrobium jeotgali* | CP011361 |
| *Aeribacillus* | 1 | 0 | 1 | apak | *Aeribacillus pallidus* | CP017703 |
| *Sporolactobacillus* | 1 | 0 | 1 | stea | *Sporolactobacillus terrae* | CP025689 |
| Unclassified *Sporolactobacillaceae* | 1 | 1 | 0 | bse | *Bacillus selenitireducens* | CP001791 |
| *Salinicoccus* | 1 | 1 | 0 | shv | *Salinicoccus halodurans* | CP011366 |
| *Auricoccus* | 1 | 1 | 0 | sbac | *Auricoccus indicus* | CP019573 |
| *Brochothrix* | 1 | 0 | 1 | bths | *Brochothrix thermosphacta* | CP023483 |
| *Thermobacillus* | 1 | 0 | 1 | tco | *Thermobacillus composti* | CP003255 |
| *Cohnella* | 1 | 0 | 1 | coh | *Cohnella* sp. 18JY8-7 | CP033433 |
| *Jeotgalibacillus* | 1 | 0 | 1 | jeo | *Jeotgalibacillus malaysiensis* | CP009416 |
| *Kurthia* | 1 | 0 | 1 | kur | *Kurthia* sp. 11kri321 | CP013217 |
| *Rummeliibacillus* | 1 | 1 | 0 | rst | *Rummeliibacillus stabekisii* | CP014806 |
| *Paenisporosarcina* | 1 | 0 | 1 | paek | *Paenisporosarcina* sp. K2R23-3 | CP032418 |
| *Novibacillus* | 1 | 0 | 1 | ntr | *Novibacillus thermophilus* | CP019699 |
| *Laceyella* | 1 | 1 | 0 | lfb | *Laceyella sacchari* | CP025943 |
| Unclassified *Aerococcaceae* | 1 | 0 | 1 | abae | *Aerococcaceae bacterium* ZY16052 | CP023434 |
| *Marinilactibacillus* | 1 | 1 | 0 | marr | *Marinilactibacillus* sp. 15R | CP017761 |
| *Geosporobacter* | 1 | 0 | 1 | gfe | *Geosporobacter ferrireducens* | CP017269 |
| *Mageeibacillus* | 1 | 0 | 1 | clo | *Mageeibacillus indolicus* | CP001850 |
| *Fastidiosipila* | 1 | 0 | 1 | fsa | *Fastidiosipila sanguinis* | CP027226 |
| Unclassified *Hungateiclostridiaceae* | 1 | 0 | 1 | ruk | *Hungateiclostridiaceae bacterium* KB18 | CP015400 |
| *Ruminiclostridium* | 1 | 0 | 1 | cce | *Ruminiclostridium cellulolyticum* | CP001348 |
| *Pseudoclostridium* | 1 | 0 | 1 | cthd | *Pseudoclostridium thermosuccinogenes* | CP021850 |
| *Flavonifractor* | 1 | 0 | 1 | fpla | *Flavonifractor plautii* | CP015406 |
| *Ethanoligenens* | 1 | 0 | 1 | eha | *Ethanoligenens harbinense* | CP002400 |
| *Caproiciproducens* | 1 | 0 | 1 | capr | *Caproiciproducens* sp. NJN-50 | CP035283 |
| *Cellulosilyticum* | 1 | 1 | 0 | cle | *Cellulosilyticum lentocellum* | CP002582 |
| *Anaerostipes* | 1 | 0 | 1 | bprl | *Anaerostipes hadrus* | FP929061 |
| *Herbinix* | 1 | 1 | 0 | hsd | *Herbinix luporum* | LN879430 |
| *Anaerotignum* | 1 | 0 | 1 | cpro | *Anaerotignum propionicum* | CP014223 |
| *Lachnoanaerobaculum* | 1 | 0 | 1 | lua | *Lachnoanaerobaculum umeaense* | CP032364 |
| *Anaerobutyricum* | 1 | 0 | 1 | ehl | *Eubacterium hallii* | LT907978 |
| *Peptoclostridium* | 1 | 0 | 1 | eac | *Peptoclostridium acidaminophilum* | CP007452 |
| *Acetoanaerobium* | 1 | 0 | 1 | cst | *Acetoanaerobium sticklandii* | FP565809 |
| *Filifactor* | 1 | 0 | 1 | faa | *Filifactor alocis* | CP002390 |
| *Paeniclostridium* | 1 | 0 | 1 | psor | *Paeniclostridium sordellii* | CP014150 |
| Unclassified *Peptostreptococcaceae* | 1 | 0 | 1 | pbq | *Peptostreptococcaceae bacterium* oral taxon 929 | CP027242 |
| *Symbiobacterium* | 1 | 0 | 1 | sth | *Symbiobacterium thermophilum* | AP006840 |
| *Syntrophomonas* | 1 | 0 | 1 | sgy | *Syntrophobotulus glycolicus* | CP002547 |
| *Syntrophothermus* | 1 | 0 | 1 | slp | *Syntrophothermus lipocalidus* | CP002048 |
| *Desulfofarcimen* | 1 | 0 | 1 | dae | *Desulfofarcimen acetoxidans* | CP001720 |
| *Desulfofundulus* | 1 | 0 | 1 | dku | *Desulfofundulus kuznetsovii* | CP002770 |
| *Desulfallas* | 1 | 0 | 1 | dgi | *Desulfallas gibsoniae* | CP003273 |
| *Pelotomaculum* | 1 | 0 | 1 | pth | *Pelotomaculum thermopropionicum* | AP009389 |
| *Desulforudis* | 1 | 0 | 1 | dau | *Candidatus Desulforudis audaxviator* | CP000860 |
| *Thermincola* | 1 | 0 | 1 | tjr | *Thermincola potens* | CP002028 |
| *Syntrophobotulus* | 1 | 0 | 1 | sgy | *Syntrophobotulus glycolicus* | CP002547 |
| *Heliobacterium* | 1 | 0 | 1 | hmo | *Heliobacterium modesticaldum* | CP000930 |
| *Acetobacterium* | 1 | 0 | 1 | awo | *Acetobacterium woodii* | CP002987 |
| *Oscillibacter* | 1 | 0 | 1 | ova | *Oscillibacter valericigenes* | AP012044 |
| *Thermaerobacter* | 1 | 0 | 1 | tmr | *Thermaerobacter marianensis* | CP002344 |
| *Carboxydocella* | 1 | 0 | 1 | cthm | *Carboxydocella thermautotrophica* | CP028491 |
| *Christensenella* | 1 | 0 | 1 | cmiu | *Christensenella minuta* | CP029256 |
| *Intestinimonas* | 1 | 0 | 1 | ibu | *Intestinimonas butyriciproducens* | CP011307 |
| *Mogibacterium* | 1 | 0 | 1 | mdv | *Mogibacterium diversum* | CP027228 |
| *Aminipila* | 1 | 0 | 1 | amij | *Aminipila* sp. JN-39 | CP035281 |
| *Caldanaerobacter* | 1 | 0 | 1 | tte | *Caldanaerobacter subterraneus* subsp. *tengcongensis* | AE008691 |
| *Carboxydothermus* | 1 | 0 | 1 | chy |  |  |
| *Moorella* | 1 | 0 | 1 | mta | *Moorella thermoacetica* | CP000232(NC_007644) |
| *Ammonifex* | 1 | 0 | 1 | adg | *Ammonifex degensii* | CP001785 |
| *Thermacetogenium* | 1 | 0 | 1 | tpz | *Thermacetogenium phaeum* | CP003732 |
| *Thermosediminibacter* | 1 | 0 | 1 | toc | *Thermosediminibacter oceani* | CP002131 |
| *Mahella* | 1 | 0 | 1 | mas | *Mahella australiensis* | CP002360 |
| *Natranaerobius* | 1 | 0 | 1 | nth | *Natranaerobius thermophilus* | CP001034 |
| *Halothermothrix* | 1 | 0 | 1 | hor | *Halothermothrix orenii* | CP001098 |
| *Halocella* | 1 | 0 | 1 | hals | *Halocella* sp. SP3-1 | CP032760 |
| *Acetohalobium* | 1 | 0 | 1 | aar | *Acetohalobium arabaticum* | CP002105 |
| *Halobacteroides* | 1 | 0 | 1 | hhl | *Halobacteroides halobius* | CP003359 |
| *Anoxybacter* | 1 | 0 | 1 | aft | *Anoxybacter fermentans* | CP016379 |
| *Finegoldia* | 1 | 0 | 1 | fma | *Finegoldia magna* | AP008971 |
| *Anaerococcus* | 1 | 0 | 1 | apr | *Anaerococcus prevotii* | CP001708 |
| *Parvimonas* | 1 | 0 | 1 | pmic | *Parvimonas micra* | CP009761 |
| *Gottschalkia* | 1 | 0 | 1 | cad | *Gottschalkia acidurici* | CP003326 |
| *Sporanaerobacter* | 1 | 0 | 1 | spoa | *Sporanaerobacter* sp. NJN-17 | CP035282 |
| *Dialister* | 1 | 0 | 1 | dpn | *Dialister pneumosintes* | CP017037 |
| *Megamonas* | 1 | 0 | 1 | mhg | *Megamonas hypermegale* | FP929048 |
| *Methylomusa* | 1 | 0 | 1 | mana | *Methylomusa anaerophila* | AP018449 |
| *Phascolarctobacterium* | 1 | 0 | 1 | pfac | *Phascolarctobacterium faecium* | AP019004 |
| *Faecalitalea* | 1 | 0 | 1 | euc | *Faecalitalea cylindroides* | FP929041 |
| *Turicibacter* | 1 | 0 | 1 | tur | *Turicibacter* sp. H121 | CP013476 |
| *Faecalibaculum* | 1 | 0 | 1 | tur | *Faecalibaculum rodentium* | CP011391 |
| *Limnochorda* | 1 | 1 | 0 | lpil | *Limnochorda pilosa* | AP014924 |
| **Total number** | **972** | **229** | **743** |  |  |  |

**Table S2:** P450s that are part of secondary metabolite biosynthetic gene clusters (BGCs) in *Firmicutes* species. Standard abbreviations representing secondary metabolite BGCs as indicated in anti-SMASH ^1^ were used in the Table.

| **Species name** | **P450** | **Reference cluster information** | | |
| --- | --- | --- | --- | --- |
|  |  | Cluster type | Most similar known cluster | similarity |
| *Alicyclobacillus acidocaldarius* subsp. *acidocaldarius* DSM 446 | CYP1706B1 | Terpene | - | - |
| *Alicyclobacillus acidocaldarius* subsp. *acidocaldarius* Tc-4-1 | CYP1706B1 | Terpene | - | - |
| *Brevibacillus laterosporus* | CYP102A45 | NRPS-like | Merosterol | 10% |
|  | CYP102A50 | terpene,NRPS,T1PKS,transAT-PKS | Tauramamide | 36% |
|  | CYP197AE1 | terpene,NRPS,T1PKS,transAT-PKS | Tauramamide | 36% |
| *Brevibacillus brevis* | CYP106C1 | NRPS,transAT-PKS | Aurantinins B-D | 28% |
| *Lentibacillus amyloliquefaciens* | CYP152K13 | Terpene | - | - |
| *Paenibacillus lautus* | CYP152K10 | T3PKS | - | - |
|  | CYP109V2 | - | - | - |
| *Paenibacillus* sp. Y412MC10 | CYP102A31 | T3PKS | - | - |
| *Paenibacillus xylanexedens* | CYP102A19 | transAT-PKS | Basiliskamides | 9% |
| *Planococcus maritimus* | CYP107DF7 | T3PKS | - | - |
| *Solibacillus silvestris* DSM 12223 | CYP152K2 | NRPS | Bacillibactin | 100% |
| *Solibacillus silvestris* StLB046 | CYP152K2 | NRPS | Bacillibactin | 30% |
| *Staphylococcus pseudintermedius* ED99 | CYP134C1 | Other | Kijanimicin | 4% |
| *Staphylococcus pseudintermedius* HKU10-03 | CYP134C1 | Other | Kijanimicin | 4% |
| *Bacillus subtilis*subsp.*subtilis*168 | CYP134A1 | Nrps-Transatpks-Otherks | Bacillaene_biosynthetic_gene_cluster | 100% |
|  |  | Other | - | - |
| *Bacillus subtilis* subsp. *subtilis* RO-NN-1 | CYP107K1 | Nrps-Transatpks-Otherks | Bacillaene_biosynthetic_gene_cluster | 100% |
| *Bacillus subtilis* subsp. *subtilis* BSP1 | CYP107K1 | Transatpks-Otherks-Nrps | Bacillaene_biosynthetic_gene_cluster | 100% |
| *Bacillus subtilis*subsp.*subtilis* 6051-HGW | CYP107K1 | Nrps-Transatpks-Otherks | Bacillaene_biosynthetic_gene_cluster | 100% |
|  | CYP107K1 | Other | - | - |
| *Bacillus subtilis* subsp. *subtilis* BAB-1 | CYP107K1 | Nrps-Transatpks-Otherks | Bacillaene_biosynthetic_gene_cluster | 92% |
| *Bacillus subtilis*subsp.*subtilis*AG1839 | CYP107K1 | Nrps-Transatpks-Otherks | Bacillaene_biosynthetic_gene_cluster | 100% |
|  | CYP107K1 | Other | - | - |
| *Bacillus subtilis*subsp.*subtilis* JH642 | CYP107K1 | Nrps-Transatpks-Otherks | Bacillaene_biosynthetic_gene_cluster | 100% |
|  | CYP134A1 | Other | - | - |
| *Bacillus subtilis*subsp.*subtilis*OH 131.1 | CYP152A1 | Lantipeptide | Rhizocticin_biosynthetic_gene_cluster | 48% |
|  | CYP107K1 | Nrps-Transatpks-Otherks | Bacillaene_biosynthetic_gene_cluster | 100% |
|  | CYP134A1 | Other | - | - |
| *Bacillus subtilis*subsp.*spizizenii*W23 | CYP152A1 | Phosphonate | Rhizocticin_biosynthetic_gene_cluster | 100% |
|  | CYP107K1 | Nrps-Transatpks-Otherks | Bacillaene_biosynthetic_gene_cluster | 100% |
|  | CYP134A1 | Other | - | - |
| *Bacillus subtilis*subsp.*spizizenii*TU-B-10 | CYP107K1 | Nrps-Transatpks-Otherks | Bacillaene_biosynthetic_gene_cluster | 100% |
|  | CYP134A1 | Other | - | - |
| *Bacillus subtilis*BSn5 | CYP102A48 | Other | O-antigen_biosynthetic_gene_cluster | 14% |
|  | CYP152A1 | Lantipeptide | Rhizocticin_biosynthetic_gene_cluster | 51% |
|  | CYP107K1 | Nrps-Transatpks-Otherks | Bacillaene_biosynthetic_gene_cluster | 100% |
| *Bacillus subtilis*QB928 | CYP107K1 | Nrps-Transatpks-Otherks | Bacillaene_biosynthetic_gene_cluster | 100% |
|  | CYP134A1 | Other | - | - |
| *Bacillus subtilis*XF-1 | CYP107K1 | Nrps-Transatpks-Otherks | Bacillaene_biosynthetic_gene_cluster | 100% |
| *Bacillus subtilis*PY79 | CYP107K1 | Nrps-Transatpks-Otherks | Bacillaene_biosynthetic_gene_cluster | 100% |
|  | CYP134A1 | Other | - | - |
| *Bacillus licheniformis*ATCC 14580 | CYP134A5 | Other | Micrococcin_P1_biosynthetic_gene_cluster | 8% |
| *Bacillus licheniformis*DSM 13 = ATCC 14580 | CYP134A5 | Other | Bacillibactin_biosynthetic_gene_cluste | 53% |
| *Bacillus paralicheniformis* | CYP134A5 | Other | Bacillomycin_biosynthetic_gene_cluster | 20% |
| *Bacillus velezensis*FZB42 | CYP107K3 | Transatpks-Nrps | Bacillaene_biosynthetic_gene_cluster | 100% |
|  | CYP107H4 | Transatpks-Nrps | Fengycin_biosynthetic_gene_cluster | 100% |
|  | CYP113L1 | Transatpks | Difficidin_biosynthetic_gene_cluster | 100% |
| *Bacillus velezensis*CAU B946 | CYP107K3 | Transatpks-Nrps | Bacillaene_biosynthetic_gene_cluster | 100% |
|  | CYP107H4 | Transatpks-Nrps | Fengycin_biosynthetic_gene_cluster | 100% |
|  | CYP113L1 | Transatpks | Difficidin_biosynthetic_gene_cluster | 100% |
| *Bacillus velezensis*YAU B9601-Y2 | CYP107K3 | Transatpks | Macrolactin_biosynthetic_gene_cluster | 100% |
|  | CYP107K3 | Transatpks-Nrps | Bacillaene_biosynthetic_gene_cluster | 100% |
|  | CYP107H4 | Transatpks-Nrps | Fengycin_biosynthetic_gene_cluster | 100% |
|  | CYP113L1 | Transatpks | Difficidin_biosynthetic_gene_cluster | 100% |
| *Bacillus velezensis*AS43.3 | CYP107K3 | Transatpks | Macrolactin_biosynthetic_gene_cluster | 100% |
|  | CYP113L1 | Transatpks-Nrps | Fengycin_biosynthetic_gene_cluster | 100% |
|  | CYP113L1 | Transatpks | Difficidin_biosynthetic_gene_cluster | 100% |
| *Bacillus velezensis*UCMB5036 | CYP107K3 | Transatpks-Nrps | Bacillaene_biosynthetic_gene_cluster | 100% |
|  | CYP107H4 | Transatpks-Nrps | Fengycin_biosynthetic_gene_cluster | 100% |
|  | CYP113L1 | Transatpks | Difficidin_biosynthetic_gene_cluster | 100% |
| *Bacillus velezensis*UCMB5033 | CYP107K3 | Transatpks-Nrps | Bacillaene_biosynthetic_gene_cluster | 100% |
|  | CYP107H4 | Transatpks-Nrps | Fengycin_biosynthetic_gene_cluster | 93% |
|  | CYP113L1 | Transatpks | Difficidin_biosynthetic_gene_cluster | 100% |
| *Bacillus velezensis*UCMB5113 | CYP107K3 | Transatpks-Nrps | Bacillaene_biosynthetic_gene_cluster | 100% |
|  | CYP107H4 | Transatpks-Nrps | Fengycin_biosynthetic_gene_cluster | 100% |
|  | CYP113L1 | Transatpks | Difficidin_biosynthetic_gene_cluster | 100% |
| *Bacillus velezensis*NAU-B3 | CYP113L1 | Transatpks | Difficidin_biosynthetic_gene_cluster | 100% |
|  | CYP107H4 | Transatpks-Nrps | Fengycin_biosynthetic_gene_cluster | 100% |
|  | CYP107K3 | Transatpks-Nrps | Bacillaene_biosynthetic_gene_cluster | 100% |
| *Bacillus velezensis*TrigoCor1448 | CYP107K3 | Transatpks-Nrps | Bacillaene_biosynthetic_gene_cluster | 100% |
|  | CYP107H4 | Transatpks-Nrps | Fengycin_biosynthetic_gene_cluster | 100% |
| *Bacillus velezensis* SQR9 | CYP107K3 | Transatpks-Nrps | Bacillaene_biosynthetic_gene_cluster | 100% |
|  | CYP107H4 | Transatpks-Nrps | Fengycin_biosynthetic_gene_cluster | 100% |
|  | CYP113L1 | Transatpks | Difficidin_biosynthetic_gene_cluster | 100% |
| *Bacillus velezensis* | CYP107K3 | Transatpks-Nrps | Bacillaene_biosynthetic_gene_cluster | 100% |
|  | CYP107H4 | Transatpks-Nrps | Fengycin_biosynthetic_gene_cluster | 100% |
|  | CYP113L1 | Transatpks | Difficidin_biosynthetic_gene_cluster | 100% |
| *Bacillus amyloliquefaciens*DSM 7 | CYP107K3 | Transatpks-Nrps | Bacillaene_biosynthetic_gene_cluster | 100% |
|  | CYP107H2 | Transatpks-Nrps | Fengycin_biosynthetic_gene_cluster | 93% |
| *Bacillus amyloliquefaciens*TA208 | CYP107H2 | Transatpks-Nrps | Fengycin_biosynthetic_gene_cluster | 93% |
|  | CYP107K3 | Transatpks-Nrps | Bacillaene_biosynthetic_gene_cluster | 100% |
| *Bacillus amyloliquefaciens*LL3 | CYP107K3 | Transatpks-Nrps | Bacillaene_biosynthetic_gene_cluster | 100% |
|  | CYP107H2 | Transatpks-Nrps | Fengycin_biosynthetic_gene_cluster | 93% |
| *Bacillus amyloliquefaciens*XH7 | CYP107H2 | Transatpks-Nrps | Fengycin_biosynthetic_gene_cluster | 93% |
|  | CYP107K3 | Transatpks-Nrps | Bacillaene_biosynthetic_gene_cluster | 100% |
| *Bacillus amyloliquefaciens*Y2 | CYP107K3 | Transatpks-Nrps | Bacillaene_biosynthetic_gene_cluster | 100% |
|  | CYP107H4 | Transatpks-Nrps | Fengycin_biosynthetic_gene_cluster | 93% |
|  | CYP113L1 | Transatpks | Difficidin_biosynthetic_gene_cluster | 100% |
| *Bacillus amyloliquefaciens*IT-45 | CYP113L1 | Transatpks | Difficidin_biosynthetic_gene_cluster | 100% |
|  | CYP107H4 | Transatpks-Nrps | Fengycin_biosynthetic_gene_cluster | 100% |
|  | CYP107K3 | Transatpks-Nrps | Bacillaene_biosynthetic_gene_cluster | 100% |
| *Bacillus amyloliquefaciens*CC178 | CYP107H4 | Transatpks-Nrps | Fengycin_biosynthetic_gene_cluster | 100% |
|  | CYP113L1 | Transatpks | Difficidin_biosynthetic_gene_cluster | 100% |
| *Bacillus amyloliquefaciens*LFB112 | CYP107K3 | Transatpks-Nrps | Bacillaene_biosynthetic_gene_cluster | 100% |
|  | CYP107H4 | Transatpks-Nrps | Fengycin_biosynthetic_gene_cluster | 100% |
|  | CYP113L1 | Transatpks | Difficidin_biosynthetic_gene_cluster | 100% |
| *Bacillus atrophaeus*1942 | CYP107K2 | Nrps-Transatpks-Otherks | - | - |
|  | CYP152A9 | Nrps | Surfactin_biosynthetic_gene_cluster | 86% |
| *Bacillus atrophaeus* NRS 1221A | CYP107K2 | Nrps-Transatpks-Otherks | Bacillaene_biosynthetic_gene_cluster | 100% |
|  | CYP152A9 | Nrps | Rhizocticin_biosynthetic_gene_cluster | 35% |
| *Bacillus vallismortis* | CYP107K3 | Transatpks-Nrps | Bacillaene_biosynthetic_gene_cluster | 100% |
|  | CYP107H4 | Transatpks-Nrps | Fengycin_biosynthetic_gene_cluster | 100% |
|  | CYP113L1 | Transatpks | Difficidin_biosynthetic_gene_cluster | 100% |
| *Bacillus pumilus*SH-B9 | CYP109B6 | Nrps | Bacillibactin_biosynthetic_gene_cluster | 53% |
| *Bacillus*sp. JS | CYP107K1 | Nrps-Transatpks-Otherks | Bacillaene_biosynthetic_gene_cluster | 100% |
| *Bacillus*sp. Pc3 | CYP107H4 | Bacteriocin-Transatpks-Nrps | Fengycin_biosynthetic_gene_cluster | 100% |
|  | CYP107K3 | Transatpks-Nrps | Bacillaene_biosynthetic_gene_cluster | 100% |
|  | CYP113L1 | Bacteriocin-Nrps | Bacillibactin_biosynthetic_gene_cluster | 100% |
| *Bacillus*sp. BH072 | CYP107K3 | Transatpks-Nrps | Bacillaene_biosynthetic_gene_cluster | 100% |
|  | CYP107H4 | Transatpks-Nrps | Fengycin_biosynthetic_gene_cluster | 100% |
|  | CYP107H4 | Transatpks | Difficidin_biosynthetic_gene_cluster | 100% |
| *Bacillus*sp. YP1 | CYP107K1 | Nrps-Transatpks-Otherks | Bacillaene_biosynthetic_gene_cluster | 100% |
| *Bacillus*sp. BS34A | CYP107K1 | Nrps-Transatpks-Otherks | Bacillaene_biosynthetic_gene_cluster | 100% |
|  | CYP134A1 | Other | - | - |
| *Bacillus*sp. LM 4-2 | CYP107K1 | Nrps-Transatpks-Otherks | Bacillaene_biosynthetic_gene_cluster | 100% |
|  | CYP102A48 | Other | O-antigen_biosynthetic_gene_cluster | 14% |
|  | CYP134A1 | Other | - | - |
| *Bacillus gibsonii* | CYP107K1 | Nrps-Transatpks-Otherks | Bacillaene_biosynthetic_gene_cluster | 100% |
|  | CYP134A1 | Other | - | - |
|  | CYP152A1 | Lantipeptide | Rhizocticin_biosynthetic_gene_cluster | 51% |
| *Bacillus xiamenensis* | CYP1179A4 | Nrps | Lichenysin_biosynthetic_gene_cluster | 71% |
| *Bacillus altitudinis* | CYP1179A4 | Nrps | Lichenysin_biosynthetic_gene_cluster | 85% |
|  | CYP109B5 | Nrps | Bacillibactin_biosynthetic_gene_cluster | 53% |
| *Bacillus*sp. SDLI1 | CYP107H4 | Transatpks-Nrps | Fengycin_biosynthetic_gene_cluster | 100% |
|  | CYP107K3 | Transatpks-Nrps | Bacillaene_biosynthetic_gene_cluster | 100% |
|  | CYP113L1 | Transatpks | Difficidin_biosynthetic_gene_cluster | 100% |

**Table S3.** Information on *Firmicutes* species P450s and those associated with secondary metabolite biosynthetic gene clusters. Standard abbreviations representing type of clusters as indicated in anti-SMASH (antibiotics & Secondary Metabolite Analysis Shell) were used in the Table.

| Species name | Species code | P450 count | No of P450 families | No of P450 subfamilies | No of BGCs | No of BGCs with P450s | BGC type | No of P450s in BGC | P450 name |
| --- | --- | --- | --- | --- | --- | --- | --- | --- | --- |
| *Paenibacillus mucilaginosus* 3016 | pmq | 11 | 8 | 8 |  |  |  |  |  |
| *Paenibacillus mucilaginosus* KNP414 | pms | 10 | 8 | 7 |  |  |  |  |  |
| *Paenibacillus mucilaginosus* K02 | pmw | 10 | 7 | 7 |  |  |  |  |  |
| *Bacillus subtilis* subsp*. spizizenii* TU-B-10 | bst | 9 | 5 | 5 | 2 | 2 | Nrps-Transatpks-Otherks, other | 2 | CYP107K1,CYP134A1 |
| *Paenibacillus xylanexedens* | pxl | 9 | 4 | 5 | 1 | 1 | transAT-PKS | 1 | CYP102A19 |
| *Bacillus vallismortis* | bvm | 9 | 5 | 5 | 3 | 3 | Transatpks-Nrps,Transatpks-Nrps,Transatpks | 3 | CYP107K3,CYP107H4,CYP113L1 |
| *Bacillus subtilis* QB928 | bsq | 8 | 5 | 5 | 2 | 2 | Nrps-Transatpks-Otherks,Other | 2 | CYP107K1,CYP134A1 |
| *Bacillus subtilis* subsp. *spizizenii* W23 | bss | 8 | 5 | 4 | 3 | 3 | Nrps-Transatpks-Otherks,Other,Phosphonate | 3 | CYP107K1,CYP134A1,CYP152A1 |
| *Bacillus subtilis* subsp. *subtilis* OH 131.1 | bsus | 8 | 5 | 5 | 3 | 3 | Nrps-Transatpks-Otherks,Other,Lantipeptide | 3 | CYP107K1,CYP134A1,CYP152A1 |
| *Bacillus* sp. SDLI1 | bacs | 6 | 4 | 5 | 3 | 3 | Transatpks-Nrps,Transatpks-Nrps,Transatpks | 3 | CYP107H4,CYP107K3,CYP113L1 |
| *Bacillus amyloliquefaciens* LL3 | bql | 7 | 4 | 5 | 2 | 2 | Transatpks-Nrps,Transatpks-Nrps | 2 | CYP107H2,CYP107K3 |
| *Bacillus amyloliquefaciens* TA208 | baz | 7 | 4 | 5 | 2 | 2 | Transatpks-Nrps,Transatpks-Nrps | 2 | CYP107H2,CYP107K3 |
| *Bacillus amyloliquefaciens* XH7 | bxh | 7 | 4 | 5 | 2 | 2 | Transatpks-Nrps,Transatpks-Nrps | 2 | CYP107H2,CYP107K3 |
| *Bacillus amyloliquefaciens* Y2 | bgy | 7 | 5 | 5 | 3 | 3 | Transatpks-Nrps,Transatpks-Nrps,Transatpks | 3 | CYP107H4,CYP107K3,CYP113L1 |
| *Bacillus sp.* BH072 | bacb | 7 | 5 | 5 | 3 | 3 | Transatpks-Nrps,Transatpks,Transatpks-Nrps | 3 | CYP107H4,CYP107H4,CYP107K3 |
| *Bacillus sp.* BS34A | bacl | 7 | 4 | 5 | 2 | 2 | Nrps-Transatpks-Otherks,Other | 2 | CYP107K1,CYP134A1 |
| *Bacillus sp.* YP1 | bacy | 7 | 4 | 5 | 1 | 1 | Nrps-Transatpks-Otherks | 1 | CYP107K1 |
| *Bacillus subtilis* BSn5 | bsn | 7 | 4 | 5 | 3 | 3 | Other,Nrps-Transatpks-Otherks,Lantipeptide | 3 | CYP102A48,CYP107K1,CYP152A1 |
| *Bacillus subtilis* PY79 | bsp | 7 | 4 | 5 | 2 | 2 | Nrps-Transatpks-Otherks,Other | 2 | CYP107K1,CYP134A1 |
| *Bacillus subtilis* subsp. *subtilis* 6051-HGW | bsh | 7 | 4 | 6 | 2 | 2 | Nrps-Transatpks-Otherks,Other | 2 | CYP107K1,CYP107K1 |
| *Bacillus subtilis* subsp. *subtilis* BSP1 | bsl | 7 | 4 | 5 | 1 | 1 | Transatpks-Otherks-Nrps | 1 | CYP107K1 |
| *Bacillus subtilis* subsp. s*ubtilis* RO-NN-1 | bsr | 7 | 4 | 5 | 1 | 1 | Nrps-Transatpks-Otherks | 1 | CYP107K1 |
| *Bacillus velezensis* NAU-B3 | bamb | 7 | 5 | 5 | 3 | 3 | Transatpks-Nrps,Transatpks-Nrps,Transatpks | 3 | CYP107H4,CYP107K3,CYP113L1 |
| *Bacillus velezensis TrigoCor*1448 | bamt | 7 | 5 | 5 | 2 | 2 | Transatpks-Nrps,Transatpks-Nrps | 2 | CYP107H4,CYP107K3 |
| *Bacillus velezensis* YAU B9601-Y2 | bya | 7 | 5 | 5 | 4 | 4 | Transatpks-Nrps,Transatpks,Transatpks-Nrps,Transatpks | 4 | CYP107H4,CYP107K3,CYP107K3,CYP113L1 |
| *Bacillus subtilis* subsp. *subtilis 1*68 | bsu | 7 | 4 | 5 | 2 | 2 | Nrps-Transatpks-Otherks,Other | 2 | CYP134A1,CYP134A1 |
| *Bacillus pseudomycoides* 219298 | bmyc | 6 | 4 | 5 |  |  |  |  |  |
| *Bacillus subtilis* subsp. *subtilis* BAB-1 | bsy | 6 | 4 | 5 | 1 | 1 | Nrps-Transatpks-Otherks | 1 | CYP107K1 |
| *Bacillus subtilis* subsp. *subtilis* JH642 | bsul | 7 | 4 | 4 | 2 | 2 | Nrps-Transatpks-Otherks,Other | 2 | CYP107K1,CYP134A1 |
| *Bacillus velezensis* UCMB5033 | bama | 7 | 5 | 5 | 3 | 3 | Transatpks-Nrps,Transatpks-Nrps,Transatpks | 3 | CYP107H4,CYP107K3,CYP113L1 |
| *Bacillus velezensis* UCMB5036 | baml | 7 | 5 | 5 | 3 | 3 | Transatpks-Nrps,Transatpks-Nrps,Transatpks | 3 | CYP107H4,CYP107K3,CYP113L1 |
| *Bacillus velezensis* UCMB5113 | bamn | 7 | 5 | 5 | 3 | 3 | Transatpks-Nrps,Transatpks-Nrps,Transatpks | 3 | CYP107H4,CYP107K3,CYP113L1 |
| *Bacillus amyloliquefaciens* DSM 7 | bao | 6 | 4 | 4 | 2 | 2 | Transatpks-Nrps,Transatpks-Nrps | 2 | CYP107H2,CYP107K3 |
| *Bacillus amyloliquefaciens* IT-45 | bami | 6 | 4 | 5 | 3 | 3 | Transatpks-Nrps,Transatpks-Nrps,Transatpks | 3 | CYP107H4,CYP107K3,CYP113L1 |
| *Bacillus amyloliquefaciens* LFB112 | bamf | 6 | 4 | 5 | 3 | 3 | Transatpks-Nrps,Transatpks-Nrps,Transatpks | 3 | CYP107H4,CYP107K3,CYP113L1 |
| *Bacillus atrophaeus* 1942 | bae | 6 | 4 | 4 | 2 | 2 | Nrps-Transatpks-Otherks,Nrps | 2 | CYP107K2,CYP152A9 |
| *Bacillus atrophaeus* NRS 1221A | batr | 6 | 4 | 4 | 2 | 2 | Nrps-Transatpks-Otherks,Nrps | 2 | CYP107K2,CYP152A9 |
| *Bacillus licheniformis* ATCC 14580 | bli | 6 | 4 | 4 | 1 | 1 | Other | 1 | CYP134A5 |
| *Bacillus licheniformis* DSM 13 = ATCC 14580 | bld | 6 | 4 | 4 | 1 | 1 | Other | 1 | CYP134A5 |
| *Bacillus megaterium* NBRC 15308 = ATCC 14581 | bmeg | 6 | 5 | 3 |  |  |  |  |  |
| *Bacillus megaterium* QM B1551 | bmq | 6 | 5 | 3 |  |  |  |  |  |
| *Bacillus* sp. Pc3 | bacp | 6 | 4 | 5 | 3 | 3 | Bacteriocin-Transatpks-Nrps,Transatpks-Nrps,Bacteriocin-Nrps | 3 | CYP107H4,CYP107K3,CYP113L1 |
| *Bacillus subtilis* subsp*. natto* BEST195 | bso | 6 | 3 | 3 |  |  |  |  |  |
| *Bacillus subtilis* subsp. *subtilis* AG1839 | bsut | 6 | 3 | 4 | 2 | 1 | Nrps-Transatpks-Otherks,Other | 1 | CYP107K1 |
| *Bacillus velezensis* | bmp | 6 | 4 | 5 | 3 | 3 | Transatpks-Nrps,Transatpks-Nrps,Transatpks | 3 | CYP107H4,CYP107K3,CYP113L1 |
| *Bacillus velezensis* CAU B946 | baq | 6 | 4 | 5 | 3 | 3 | Transatpks-Nrps,Transatpks-Nrps,Transatpks | 3 | CYP107H4,CYP107K3,CYP109B7 |
| *Bacillus glycinifermentans* | bgy | 6 | 3 | 3 |  |  |  |  |  |
| *Bacillus anthracis* A16 | bant | 3 | 3 | 3 |  |  |  |  |  |
| *Bacillus anthracis Ames* | ban | 3 | 3 | 3 |  |  |  |  |  |
| *Bacillus cereus* AH820 | bcu | 6 | 4 | 4 |  |  |  |  |  |
| *Bacillus thuringiensis* HD-771 | bti | 6 | 3 | 3 |  |  |  |  |  |
| *Bacillus cereus* ATCC 10987 | bca | 5 | 4 | 5 |  |  |  |  |  |
| *Bacillus sp.* LM 4-2 | balm | 6 | 4 | 3 | 3 | 3 | Other,Nrps-Transatpks-Otherks,Other | 3 | CYP102A48,CYP107K1,CYP134A1 |
| *Bacillus bombysepticus* | bby | 5 | 4 | 4 |  |  |  |  |  |
| *Bacillus cereus* FRI*-*35 | bcer | 5 | 4 | 5 |  |  |  |  |  |
| *Bacillus endophyticus* | beo | 5 | 4 | 4 |  |  |  |  |  |
| *Bacillus megaterium* DSM 319 | bmd | 5 | 4 | 2 |  |  |  |  |  |
| *Bacillus megaterium* WSH*-*002 | bmh | 5 | 4 | 2 |  |  |  |  |  |
| *Bacillus paralicheniformis* | blh | 5 | 4 | 3 | 1 | 1 | Other | 1 | CYP134A5 |
| *Bacillus sp.* JS | bjs | 5 | 4 | 3 | 1 | 1 | Nrps-Transatpks-Otherks | 1 | CYP107K1 |
| *Planococcus maritimus* | pmar | 5 | 4 | 4 | 1 | 1 | T3PKS | 1 | CYP107DF7 |
| *Bacillus gibsonii* | bgi | 6 | 4 | 3 | 3 | 3 | Nrps-Transatpks-Otherks,Other,Lantipeptide | 3 | CYP107K1,CYP134A1,CYP152A1 |
| *Bacillus velezensis* SQR9 | bamy | 6 | 4 | 5 | 3 | 3 | Transatpks-Nrps,Transatpks-Nrps,Transatpks | 3 | CYP107K3,CYP107H4,CYP113L1 |
| *Bacillus cereus* 03BB102 | bcx | 4 | 4 | 4 |  |  |  |  |  |
| *Bacillus cereus biovar anthracis* CI | bal | 4 | 4 | 4 |  |  |  |  |  |
| *Bacillus cereus* E33L | bcz | 4 | 4 | 4 |  |  |  |  |  |
| *Bacillus cereus* FT9 | bcef | 4 | 4 | 4 |  |  |  |  |  |
| *Bacillus cytotoxicus* | bcy | 4 | 2 | 3 |  |  |  |  |  |
| *Bacillus simplex* | bsj | 4 | 4 | 1 |  |  |  |  |  |
| *Bacillus subtilis* XF-1 | bsx | 4 | 3 | 3 | 1 | 1 | Nrps-Transatpks-Otherks | 1 | CYP107K1 |
| *Bacillus thuringiensis* YBT*-*1518 | bthu | 4 | 3 | 4 |  |  |  |  |  |
| *Bacillus velezensis* AS43.3 | bamp | 4 | 3 | 3 | 3 | 3 | Transatpks,Transatpks-Nrps,Transatpks | 3 | CYP107K3,CYP113L1,CYP113L1 |
| *Virgibacillus phasianinus* | vil | 4 | 3 | 3 |  |  |  |  |  |
| *Paenibacillus* sp. Y412MC10 | gym | 4 | 4 | 3 | 1 | 1 | T3PKS | 1 | CYP102A31 |
| *Brevibacillus laterosporus* | blr | 4 | 3 | 3 | 3 | 3 | NRPS-like,terpene,NRPS,T1PKS,transAT-PKS,terpene,NRPS,T1PKS,transAT-PKS | 3 | CYP102A45,CYP102A50, CYP197AE1 |
| *Bacillus thuringiensis* YWC2-8 | bthy | 3 | 3 | 3 |  |  |  |  |  |
| *Bacillus amyloliquefaciens* CC178 | bamc | 5 | 3 | 4 | 2 | 2 | Transatpks-Nrps,Transatpks | 2 | CYP107H4,CYP113L1 |
| *Bacillus xiamenensis* | bxi | 2 | 2 | 1 |  |  |  |  |  |
| *Bacillus anthracis Ames Ancestor* | bar | 3 | 3 | 3 |  |  |  |  |  |
| *Bacillus anthracis* CDC 684 | bah | 3 | 3 | 3 |  |  |  |  |  |
| *Bacillus anthracis* HYU01 | banh | 3 | 3 | 3 |  |  |  |  |  |
| *Bacillus anthracis Sterne* | bat | 3 | 3 | 3 |  |  |  |  |  |
| *Bacillus anthracis Vollum* | banv | 3 | 3 | 3 |  |  |  |  |  |
| *Bacillus cereus* B4264 | bcb | 3 | 3 | 3 |  |  |  |  |  |
| *Bacillus cereus* G9842 | bcg | 3 | 3 | 3 |  |  |  |  |  |
| *Bacillus cereus* NC7401 | bnc | 3 | 3 | 3 |  |  |  |  |  |
| *Bacillus cereus* Q1 | bcq | 3 | 2 | 3 |  |  |  |  |  |
| *Bacillus pseudofirmus* | bpf | 3 | 3 | 2 |  |  |  |  |  |
| *Bacillus pumilus* MTCC B6033 | bpum | 3 | 2 | 2 |  |  |  |  |  |
| *Bacillus pumilus* SAFR-032 | bpu | 3 | 2 | 2 |  |  |  |  |  |
| *Bacillus pumilus* SH-B9 | bpus | 3 | 2 | 2 | 1 | 1 | Nrps | 1 | CYP109B6 |
| *Bacillus thuringiensis* Al Hakam | btl | 3 | 3 | 3 |  |  |  |  |  |
| *Bacillus thuringiensis* Bt407 | btg | 3 | 3 | 3 |  |  |  |  |  |
| *Bacillus thuringiensis* HD1011 | btw | 3 | 3 | 3 |  |  |  |  |  |
| *Bacillus thuringiensis* HD-789 | btn | 3 | 3 | 3 |  |  |  |  |  |
| *Bacillus thuringiensis* MC28 | btm | 3 | 3 | 3 |  |  |  |  |  |
| *Bacillus thuringiensis serovar chinensis* CT-43 | btc | 3 | 3 | 3 |  |  |  |  |  |
| *Bacillus thuringiensis serovar finitimus* YBT-020 | btf | 3 | 3 | 3 |  |  |  |  |  |
| *Bacillus thuringiensis serovar kurstaki* YBT-1520 | bthr | 3 | 3 | 3 |  |  |  |  |  |
| *Bacillus thuringiensis serovar thuringiensis* IS5056 | btht | 3 | 3 | 3 |  |  |  |  |  |
| *Bacillus velezensis* FZB42 | bay | 5 | 3 | 4 | 3 | 3 | Transatpks-Nrps,Transatpks-Nrps,Transatpks | 3 | CYP107K3,CYP107H4,CYP113L1 |
| *Bacillus thuringiensis serovar kurstaki* HD-1 | bthi | 3 | 2 | 2 |  |  |  |  |  |
| *Bacillus anthracis* H9401 | bax | 3 | 3 | 3 |  |  |  |  |  |
| *Bacillus anthracis* A16R | banr | 3 | 3 | 3 |  |  |  |  |  |
| *Bacillus mycoides* KBAB4 | bwe | 3 | 3 | 3 |  |  |  |  |  |
| *Bacillus mycoides* WSBC 10204 | bww | 3 | 3 | 3 |  |  |  |  |  |
| *Planococcus rifietoensis* | prt | 3 | 3 | 3 |  |  |  |  |  |
| *Planococcus plakortidis* | ppla | 3 | 3 | 3 |  |  |  |  |  |
| *Halobacillus halophilus* | hhd | 3 | 3 | 2 |  |  |  |  |  |
| *Bacillus altitudinis* | balt | 2 | 2 | 2 | 2 | 2 | Nrps,Nrps | 2 | CYP1179A4,CYP109B5 |
| *Bacillus cereus* ATCC 14579 | bce | 2 | 2 | 2 |  |  |  |  |  |
| *Bacillus anthracis* SVA11 | bans | 2 | 2 | 2 |  |  |  |  |  |
| *Bacillus infantis* | bif | 2 | 2 | 2 |  |  |  |  |  |
| *Bacillus sp.* WP8 | bacw | 2 | 2 | 1 |  |  |  |  |  |
| *Bacillus thuringiensis serovar kurstaki* HD73 | btt | 2 | 2 | 2 |  |  |  |  |  |
| *Bacillus anthracis* A0248 | bai | 2 | 2 | 2 |  |  |  |  |  |
| *Bacillus mycoides* ATCC 6462 | bmyo | 2 | 2 | 2 |  |  |  |  |  |
| *Bacillus thuringiensis* BMB171 | btb | 2 | 2 | 2 |  |  |  |  |  |
| *Staphylococcus pseudintermedius* HKU10-03 | ssd | 2 | 2 | 2 | 1 | 1 | Other | 1 | CYP134C1 |
| *Staphylococcus pseudintermedius* ED99 | sdt | 2 | 2 | 2 | 1 | 1 | Other | 1 | CYP134C1 |
| *Halobacillus mangrovi* | hmn | 2 | 2 | 2 |  |  |  |  |  |
| *Salimicrobium jeotgali* | sje | 2 | 2 | 2 |  |  |  |  |  |
| *Salinicoccus halodurans* | shv | 2 | 1 | 2 |  |  |  |  |  |
| *Brevibacillus brevis* | bbe | 2 | 2 | 1 | 1 | 1 | NRPS,transAT-PKS | 1 | CYP106C1 |
| *Paenibacillus bovis* | pbv | 2 | 2 | 1 |  |  |  |  |  |
| *Paenibacillus swuensis* | pswu | 2 | 2 | 1 |  |  |  |  |  |
| *Paenibacillus lautus* | plw | 2 | 2 | 2 | 1 | 1 | T3PKS | 1 | CYP152K10 |
| *Planococcus* sp. MB-3u-03 | plx | 2 | 2 | 2 |  |  |  |  |  |
| *Sporosarcina* sp. P37 | spop | 2 | 2 | 2 |  |  |  |  |  |
| *Laceyella sacchari* | lfb | 2 | 2 | 2 |  |  |  |  |  |
| *Paenibacillus polymyxa* CR1 | ppol | 2 | 2 | 2 |  |  |  |  |  |
| *Paenibacillus* sp. IHBB 10380 | pxl | 2 | 2 | 1 |  |  |  |  |  |
| *Paenibacillus* sp. 32O-W | pow | 2 | 2 | 1 |  |  |  |  |  |
| *Paenibacillus beijingensis* | pbj | 2 | 1 | 1 |  |  |  |  |  |
| *Bacillus cellulosilyticus* | bco | 1 | 1 | 1 |  |  |  |  |  |
| *Bacillus cereus* F837/76 | bcf | 1 | 1 | 1 |  |  |  |  |  |
| *Bacillus clausii* | bcl | 1 | 1 | 1 |  |  |  |  |  |
| *Bacillus coagulans* 36D1 | bag | 1 | 1 | 1 |  |  |  |  |  |
| *Bacillus flexus* | bfx | 1 | 1 | 1 |  |  |  |  |  |
| *Bacillus halodurans* | bha | 1 | 1 | 1 |  |  |  |  |  |
| *Bacillus lehensis* | ble | 1 | 1 | 1 |  |  |  |  |  |
| *Bacillus oceanisediminis* | bon | 1 | 1 | 1 |  |  |  |  |  |
| *Bacillus smithii* | bsm | 1 | 1 | 1 |  |  |  |  |  |
| *Bacillus sp.* OxB-1 | baco | 1 | 1 | 1 |  |  |  |  |  |
| *Bacillus toyonensis* | bty | 1 | 1 | 1 |  |  |  |  |  |
| *Paenibacillus larvae* | plv | 1 | 1 | 1 |  |  |  |  |  |
| *Oceanobacillus iheyensis* | oih | 1 | 1 | 1 |  |  |  |  |  |
| *Oceanobacillus* sp. 160 | ocn | 1 | 1 | 1 |  |  |  |  |  |
| *Anoxybacillus amylolyticus* | aamy | 1 | 1 | 1 |  |  |  |  |  |
| *Terribacillus goriensis* | tap | 1 | 1 | 1 |  |  |  |  |  |
| *Virgibacillus* sp. SK37 | vir | 1 | 1 | 1 |  |  |  |  |  |
| *Virgibacillus halodenitrificans* | vhl | 1 | 1 | 1 |  |  |  |  |  |
| *Virgibacillus necropolis* | vne | 1 | 1 | 1 |  |  |  |  |  |
| *Lentibacillus amyloliquefaciens* | lao | 1 | 1 | 1 | 1 | 1 | Terpene | 1 | CYP152K13 |
| *Bacillus selenitireducens* | bse | 1 | 1 | 1 |  |  |  |  |  |
| *Staphylococcus hyicus* | shu | 1 | 1 | 1 |  |  |  |  |  |
| *Staphylococcus agnetis* | sagq | 1 | 1 | 1 |  |  |  |  |  |
| *Staphylococcus lutrae* | slz | 1 | 1 | 1 |  |  |  |  |  |
| *Staphylococcus sciuri* | sscu | 1 | 1 | 1 |  |  |  |  |  |
| *Staphylococcus felis* | sfq | 1 | 1 | 1 |  |  |  |  |  |
| *Macrococcus caseolyticus* | mcl | 1 | 1 | 1 |  |  |  |  |  |
| *Macrococcus* sp. IME1552 | macr | 1 | 1 | 1 |  |  |  |  |  |
| *Auricoccus indicus* | sbac | 1 | 1 | 1 |  |  |  |  |  |
| *Exiguobacterium* sp. AT1b | eat | 1 | 1 | 1 |  |  |  |  |  |
| *Paenibacillus polymyxa* SC2 | ppm | 1 | 1 | 1 |  |  |  |  |  |
| *Paenibacillus polymyxa* M1 | ppo | 1 | 1 | 1 |  |  |  |  |  |
| *Paenibacillus polymyxa* SQR-21 | ppq | 1 | 1 | 1 |  |  |  |  |  |
| *Paenibacillus polymyxa* Sb3-1 | ppoy | 1 | 1 | 1 |  |  |  |  |  |
| *Paenibacillus riograndensis* | pri | 1 | 1 | 1 |  |  |  |  |  |
| *Paenibacillus peoriae* | ppeo | 1 | 1 | 1 |  |  |  |  |  |
| *Paenibacillus donghaensis* | pdh | 1 | 1 | 1 |  |  |  |  |  |
| *Paenibacillus ihbetae* | pib | 1 | 1 | 1 |  |  |  |  |  |
| *Aneurinibacillus* sp. XH2 | anx | 1 | 1 | 1 |  |  |  |  |  |
| *Aneurinibacillus soli* | asoc | 1 | 1 | 1 |  |  |  |  |  |
| *Alicyclobacillus acidocaldarius* subsp. *acidocaldarius* DSM 446 | aac | 1 | 1 | 1 | 1 | 1 | Terpene | 1 | CYP1706B1 |
| *Alicyclobacillus acidocaldarius* subsp. *acidocaldarius* Tc-4-1 | aad | 1 | 1 | 1 |  |  | Terpene | 1 | CYP1706B1 |
| *Kyrpidia spormannii* | kyr | 1 | 1 | 1 |  |  |  |  |  |
| *Solibacillus silvestris* StLB046 | siv | 1 | 1 | 1 | 1 | 1 | NRPS | 1 | CYP152K2 |
| *Solibacillus silvestris* DSM 12223 | ssil | 1 | 1 | 1 | 1 | 1 | NRPS | 1 | CYP152K2 |
| *Planococcus antarcticus* | pana | 1 | 1 | 1 |  |  |  |  |  |
| *Planococcus halocryophilus* | phc | 1 | 1 | 1 |  |  |  |  |  |
| *Sporosarcina* sp. P33 | spor | 1 | 1 | 1 |  |  |  |  |  |
| *Sporosarcina ureae* | sure | 1 | 1 | 1 |  |  |  |  |  |
| *Rummeliibacillus stabekisii* | rst | 1 | 1 | 1 |  |  |  |  |  |
| *Enterococcus faecium* Aus0085 | efau | 1 | 1 | 1 |  |  |  |  |  |
| *Enterococcus faecium* DO | efu | 1 | 1 | 1 |  |  |  |  |  |
| *Enterococcus faecium* T110 | eft | 1 | 1 | 1 |  |  |  |  |  |
| *Enterococcus hirae* | ehr | 1 | 1 | 1 |  |  |  |  |  |
| *Enterococcus mundtii* | emu | 1 | 1 | 1 |  |  |  |  |  |
| *Enterococcus gilvus* | egv | 1 | 1 | 1 |  |  |  |  |  |
| *Tetragenococcus halophilus* | thl | 1 | 1 | 1 |  |  |  |  |  |
| *Tetragenococcus osmophilus* | too | 1 | 1 | 1 |  |  |  |  |  |
| *Tetragenococcus koreensis* | tkr | 1 | 1 | 1 |  |  |  |  |  |
| *Weissella jogaejeotgali* | wjo | 1 | 1 | 1 |  |  |  |  |  |
| *Carnobacterium* sp. CP1 | carc | 1 | 1 | 1 |  |  |  |  |  |
| *Marinilactibacillus* sp. 15R | marr | 1 | 1 | 1 |  |  |  |  |  |
| *Jeotgalibaca dankookensis* | jda | 1 | 1 | 1 |  |  |  |  |  |
| *Jeotgalibaca* sp. H21T32 | jeh | 1 | 1 | 1 |  |  |  |  |  |
| *Staphylococcus epidermidis* SEI | seps | 1 | 1 | 1 |  |  |  |  |  |
| *Staphylococcus haemolyticus* JCSC1435 | sha | 1 | 1 | 1 |  |  |  |  |  |
| *Paenibacillus naphthalenovorans* | pnp | 1 | 1 | 1 |  |  |  |  |  |
| *Enterococcus faecium* Aus0004 | efc | 1 | 1 | 1 |  |  |  |  |  |
| *Enterococcus faecium* ATCC 8459 = NRRL B-2354 | efm | 1 | 1 | 1 |  |  |  |  |  |
| *Sulfobacillus acidophilus* TPY | say | 2 | 2 | 2 |  |  |  |  |  |
| *Sulfobacillus acidophilus* DSM 10332 | sap | 2 | 2 | 2 |  |  |  |  |  |
| *Clostridium acetobutylicum* EA 2018 | cay | 2 | 1 | 1 |  |  |  |  |  |
| *Clostridium acetobutylicum* ATCC 824 | cac | 1 | 1 | 1 |  |  |  |  |  |
| *Clostridium acetobutylicum* DSM 1731 | cae | 1 | 1 | 1 |  |  |  |  |  |
| *Clostridium botulinum* A ATCC 3502 | cbo | 1 | 1 | 1 |  |  |  |  |  |
| *Clostridium botulinum* A ATCC 19397 | cba | 1 | 1 | 1 |  |  |  |  |  |
| *Clostridium botulinum* A Hall | cbh | 1 | 1 | 1 |  |  |  |  |  |
| *Clostridium botulinum* A3 Loch Maree | cbl | 1 | 1 | 1 |  |  |  |  |  |
| *Clostridium botulinum* B1 Okra | cbb | 1 | 1 | 1 |  |  |  |  |  |
| *Clostridium botulinum* F Langeland | cbf | 1 | 1 | 1 |  |  |  |  |  |
| *Clostridium botulinum* F 230613 | cbm | 1 | 1 | 1 |  |  |  |  |  |
| *Clostridium botulinum* H04402 065 | cbj | 1 | 1 | 1 |  |  |  |  |  |
| *Clostridium pasteurianum* DSM 525 = ATCC 6013 | cpae | 1 | 1 | 1 |  |  |  |  |  |
| *Clostridium pasteurianum* DSM 525 = ATCC 6013 | cpat | 1 | 1 | 1 |  |  |  |  |  |
| *Clostridium bornimense* | clt | 1 | 1 | 1 |  |  |  |  |  |
| *Clostridium sporogenes* | cld | 1 | 1 | 1 |  |  |  |  |  |
| *Ruminococcaceae bacterium* CPB6 | rbp | 1 | 1 | 1 |  |  |  |  |  |
| *Lachnoclostridium phytofermentans* | cpy | 1 | 1 | 1 |  |  |  |  |  |
| *Herbinix luporum* | hsd | 1 | 1 | 1 |  |  |  |  |  |
| *Clostridium pasteurianum* BC1 | cpas | 1 | 1 | 1 |  |  |  |  |  |
| *Cellulosilyticum lentocellum* | cle | 1 | 1 | 1 |  |  |  |  |  |
| *Clostridium saccharolyticum* WM1 | csh | 1 | 1 | 1 |  |  |  |  |  |
| *Clostridium botulinum* Ba4 | cbi | 1 | 1 | 1 |  |  |  |  |  |
| *Clostridium botulinum* A2 | cby | 1 | 1 | 1 |  |  |  |  |  |
| *Limnochorda pilosa* | lpil | 1 | 1 | 1 |  |  |  |  |  |
| *Bacillus mycoides* 219298 | bmyc |  |  |  | 7 |  |  |  |  |
| *Bacillus cereus* AH187 | bcr |  |  |  | 7 |  |  |  |  |
| *Bacillus krulwichiae* | bkw |  |  |  | 3 |  |  |  |  |
| *Bacillus horikoshii* | bhk |  |  |  | 5 |  |  |  |  |
| *Bacillus methanolicus* | bmet |  |  |  | 5 |  |  |  |  |
| *Bacillus beveridgei* | bbev |  |  |  | 2 |  |  |  |  |
| *Bacillus kochii* | bko |  |  |  | 8 |  |  |  |  |
| *Bacillus sp.* 1NLA3E | baci |  |  |  | 9 |  |  |  |  |
| *Bacillus sp.* X1(2014) | bsg |  |  |  | 4 |  |  |  |  |
| *Bacillus thuringiensis serovar konkukian* 97-27 | btk |  |  |  |  |  |  |  |  |
| *Bacillus weihaiensis* | bwh |  |  |  | 5 |  |  |  |  |
| *Geobacillus kaustophilus* | gka |  |  |  | 5 |  |  |  |  |
| *Geobacillus thermoleovorans* CCB_US3_UF5 | gte |  |  |  | 6 |  |  |  |  |
| *Geobacillus thermoleovorans* KCTC 3570 | gtk |  |  |  | 4 |  |  |  |  |
| *Geobacillus thermocatenulatus* | gtm |  |  |  | 5 |  |  |  |  |
| *Geobacillus lituanicus* | gli |  |  |  | 5 |  |  |  |  |
| *Geobacillus thermodenitrificans* | gtn |  |  |  | 5 |  |  |  |  |
| *Geobacillus* sp. WCH70 | gwc |  |  |  | 3 |  |  |  |  |
| *Geobacillus* sp. Y412MC61 | gyc |  |  |  | 5 |  |  |  |  |
| *Geobacillus* sp. Y412MC52 | gya |  |  |  | 5 |  |  |  |  |
| *Geobacillus* sp. C56-T3 | gct |  |  |  | 6 |  |  |  |  |
| *Geobacillus* sp. Y4.1MC1 | gmc |  |  |  | 5 |  |  |  |  |
| *Geobacillus* sp. GHH01 | ggh |  |  |  | 3 |  |  |  |  |
| *Geobacillus genomosp*. 3 | gjf |  |  |  | 5 |  |  |  |  |
| *Geobacillus* sp. 12AMOR1 | gea |  |  |  | 2 |  |  |  |  |
| *Geobacillus* sp. LC300 | gel |  |  |  | 2 |  |  |  |  |
| *Geobacillus stearothermophilus* | gse |  |  |  | 2 |  |  |  |  |
| *Geobacillus subterraneus* | gsr |  |  |  |  |  |  |  |  |
| *Geobacillus* sp. JS12 | gej |  |  |  | 5 |  |  |  |  |
| *Parageobacillus thermoglucosidasius* C56-YS93 | gth |  |  |  | 6 |  |  |  |  |
| *Parageobacillus thermoglucosidasius* DSM 2542 | ptl |  |  |  | 6 |  |  |  |  |
| *Anoxybacillus flavithermus* | aft |  |  |  | 6 |  |  |  |  |
| *Anoxybacillus gonensis* | agn |  |  |  | 4 |  |  |  |  |
| *Anoxybacillus* sp. B2M1 | anm |  |  |  | 5 |  |  |  |  |
| *Anoxybacillus* sp. B7M1 | anl |  |  |  | 5 |  |  |  |  |
| *Amphibacillus xylanus* | axl |  |  |  | 4 |  |  |  |  |
| *Lysinibacillus sphaericus* | lsp |  |  |  | 9 |  |  |  |  |
| *Lysinibacillus varians* | lgy |  |  |  | 9 |  |  |  |  |
| *Lysinibacillus fusiformis* | lfu |  |  |  | 8 |  |  |  |  |
| *Lysinibacillus* sp. YS11 | lys |  |  |  | 6 |  |  |  |  |
| *Lysinibacillus* sp. B2A1 | lyb |  |  |  | 6 |  |  |  |  |
| *Lysinibacillus* sp. 2017 | lyz |  |  |  | 3 |  |  |  |  |
| *Virgibacillus* sp. 6R | vig |  |  |  | 6 |  |  |  |  |
| *Virgibacillus dokdonensis* | vpn |  |  |  | 5 |  |  |  |  |
| *Fictibacillus phosphorivorans* | fpn |  |  |  | 5 |  |  |  |  |
| *Fictibacillus arsenicus* | far |  |  |  | 4 |  |  |  |  |
| *Aeribacillus pallidus* | apak |  |  |  | 3 |  |  |  |  |
| *Sporolactobacillus terrae* | stea |  |  |  | 1 |  |  |  |  |
| *Staphylococcus aureus* subsp. *aureus* N315 (MRSA/VSSA) | sau |  |  |  |  |  |  |  |  |
| *Staphylococcus aureus subsp. aureus Mu50 (MRSA/VISA)* | sav |  |  |  | 5 |  |  |  |  |
| *Staphylococcus aureus* subsp. *aureus Mu3* (MRSA/hetero-VISA) | saw |  |  |  | 5 |  |  |  |  |
| *Staphylococcus aureus* subsp. *aureus* JH1 (MRSA/VSSA) | sah |  |  |  | 4 |  |  |  |  |
| *Staphylococcus aureus* subsp. *aureus* JH9 (MRSA/VISA) | saj |  |  |  | 4 |  |  |  |  |
| *Staphylococcus aureus* subsp. *aureus* MW2 (CA-MRSA) | sam |  |  |  |  |  |  |  |  |
| *Staphylococcus aureus* subsp. *aureus* MSSA476 (MSSA) | sas |  |  |  |  |  |  |  |  |
| *Staphylococcus aureus* subsp. *aureus* MRSA252 (MRSA) | sar |  |  |  | 6 |  |  |  |  |
| *Staphylococcus aureus* subsp. *aureus* COL (MRSA) | sac |  |  |  | 4 |  |  |  |  |
| *Staphylococcus aureus* subsp. *aureus* USA300_TCH1516 (CA-MRSA) | sax |  |  |  | 5 |  |  |  |  |
| *Staphylococcus aureus* subsp. *aureus* USA300_FPR3757 (CA-MRSA) | saa |  |  |  | 5 |  |  |  |  |
| *Staphylococcus aureus* subsp. *aureus* NCTC8325 | sao |  |  |  | 5 |  |  |  |  |
| *Staphylococcus aureus* subsp. *aureus* Newman | sae |  |  |  | 5 |  |  |  |  |
| *Staphylococcus aureus* subsp. *aureus* ED98 | sad |  |  |  | 4 |  |  |  |  |
| *Staphylococcus aureus* subsp. *aureus* M013 (CA-MRSA) | suu |  |  |  | 5 |  |  |  |  |
| *Staphylococcus aureus* subsp. *aureus* VC40 | suv |  |  |  | 5 |  |  |  |  |
| *Staphylococcus aureus* subsp. *aureus* ED133 | sue |  |  |  | 6 |  |  |  |  |
| *Staphylococcus aureus* subsp. *aureus* JKD6159 (CA-MRSA) | suj |  |  |  | 5 |  |  |  |  |
| *Staphylococcus aureus* subsp. *aureus* JKD6008 (MRSA/VISA) | suk |  |  |  | 4 |  |  |  |  |
| *Staphylococcus aureus* subsp. *aureus* ECT-R 2 | suc |  |  |  | 4 |  |  |  |  |
| *Staphylococcus aureus* subsp. *aureus* T0131 (MRSA) | sut |  |  |  | 5 |  |  |  |  |
| *Staphylococcus aureus* subsp. *aureus* TCH60 | suq |  |  |  | 4 |  |  |  |  |
| *Staphylococcus aureus* subsp. *aureus* 11819-97 (CA-MRSA) | suz |  |  |  | 5 |  |  |  |  |
| *Staphylococcus aureus* subsp. *aureus* 71193 (MSSA) | sud |  |  |  | 5 |  |  |  |  |
| *Staphylococcus aureus* subsp. *aureus* HO 5096 0412 (MRSA) | sux |  |  |  | 5 |  |  |  |  |
| *Staphylococcus aureus* subsp. *aureus* TW20 (MRSA) | suw |  |  |  |  |  |  |  |  |
| *Staphylococcus aureus* subsp. *aureus* ST398 (MRSA) | sug |  |  |  | 5 |  |  |  |  |
| *Staphylococcus aureus* subsp. *aureus* LGA251 (MRSA) | suf |  |  |  | 6 |  |  |  |  |
| *Staphylococcus aureus* subsp. *aureus* 55/2053 | saua |  |  |  | 4 |  |  |  |  |
| *Staphylococcus aureus* subsp. *aureus* 6850 (MSSA) | saue |  |  |  | 5 |  |  |  |  |
| *Staphylococcus aureus* subsp. *aureus* CN1 (CA-MRSA) | saun |  |  |  | 4 |  |  |  |  |
| *Staphylococcus aureus* subsp. *aureus* SA40 (CA-MRSA) | saus |  |  |  | 5 |  |  |  |  |
| *Staphylococcus aureus* subsp. *aureus* SA957 (CA-MRSA) | sauu |  |  |  | 5 |  |  |  |  |
| *Staphylococcus aureus* subsp. *aureus* SA268 (CA-MRSA) | saug |  |  |  | 5 |  |  |  |  |
| *Staphylococcus aureus* subsp. *aureus* Z172 (MRSA/VISA) | sauz |  |  |  | 5 |  |  |  |  |
| *Staphylococcus aureus* subsp. *aureus* ST228/10388 (MRSA) | saut |  |  |  | 4 |  |  |  |  |
| *Staphylococcus aureus* subsp. *aureus* ST228/10497 (MRSA) | sauj |  |  |  | 4 |  |  |  |  |
| *Staphylococcus aureus* subsp. *aureus* ST228/15532 (MRSA) | sauk |  |  |  | 4 |  |  |  |  |
| *Staphylococcus aureus* subsp. *aureus* ST228/16035 (MRSA) | sauq |  |  |  | 4 |  |  |  |  |
| *Staphylococcus aureus* subsp. *aureus* ST228/18412 (MRSA) | sauv |  |  |  | 4 |  |  |  |  |
| *Staphylococcus aureus* subsp. *aureus* ST228/16125 (MRSA) | sauw |  |  |  | 4 |  |  |  |  |
| *Staphylococcus aureus* subsp. *aureus* ST228/18341 (MRSA) | saux |  |  |  | 4 |  |  |  |  |
| *Staphylococcus aureus* subsp. *aureus* ST228/18583 (MRSA) | sauy |  |  |  | 4 |  |  |  |  |
| *Staphylococcus aureus* subsp. *aureus* FDAARGOS_5 | sauf |  |  |  | 6 |  |  |  |  |
| *Staphylococcus aureus* RF122 | sab |  |  |  | 6 |  |  |  |  |
| *Staphylococcus aureus* 04-02981 (MRSA) | suy |  |  |  | 5 |  |  |  |  |
| *Staphylococcus aureus* 08BA02176 (LA-MRSA) | saub |  |  |  | 5 |  |  |  |  |
| *Staphylococcus aureus* M1 (MRSA) | saum |  |  |  | 5 |  |  |  |  |
| *Staphylococcus aureus* CA-347 (MRSA) | sauc |  |  |  | 4 |  |  |  |  |
| *Staphylococcus aureus* Bmb9393 (MRSA) | saur |  |  |  | 5 |  |  |  |  |
| *Staphylococcus aureus* USA300-ISMMS1 (MRSA) | saui |  |  |  | 5 |  |  |  |  |
| *Staphylococcus aureus* 502A | saud |  |  |  | 4 |  |  |  |  |
| *Staphylococcus aureus* MS4 | sams |  |  |  | 5 |  |  |  |  |
| *Staphylococcus argenteus* | suh |  |  |  | 5 |  |  |  |  |
| *Staphylococcus epidermidis* ATCC 12228 | sep |  |  |  | 3 |  |  |  |  |
| *Staphylococcus epidermidis* RP62A (MRSE) | ser |  |  |  | 3 |  |  |  |  |
| *Staphylococcus epidermidis* PM221 | sepp |  |  |  | 3 |  |  |  |  |
| *Staphylococcus haemolyticus* Sh29/312/L2 | shh |  |  |  | 2 |  |  |  |  |
| *Staphylococcus saprophyticus* | ssp |  |  |  | 3 |  |  |  |  |
| *Staphylococcus carnosus* | sca |  |  |  |  |  |  |  |  |
| *Staphylococcus lugdunensis* HKU09-01 | slg |  |  |  | 5 |  |  |  |  |
| *Staphylococcus lugdunensis* N920143 | sln |  |  |  | 6 |  |  |  |  |
| *Staphylococcus warneri* | swa |  |  |  | 2 |  |  |  |  |
| *Staphylococcus pasteuri* | spas |  |  |  | 2 |  |  |  |  |
| *Staphylococcus xylosus* HKUOPL8 | sxy |  |  |  | 5 |  |  |  |  |
| *Staphylococcus xylosus* SMQ-121 | sxl |  |  |  | 7 |  |  |  |  |
| *Staphylococcus xylosus* C2a | sxo |  |  |  | 5 |  |  |  |  |
| *Staphylococcus capitis* | scap |  |  |  | 4 |  |  |  |  |
| *Staphylococcus schleiferi* 1360-13 | ssch |  |  |  | 3 |  |  |  |  |
| *Staphylococcus schleiferi* 2317-03 | sscz |  |  |  | 3 |  |  |  |  |
| *Staphylococcus equorum* | seqo |  |  |  | 7 |  |  |  |  |
| *Staphylococcus simulans* | ssif |  |  |  | 4 |  |  |  |  |
| *Staphylococcus condimenti* | scv |  |  |  | 4 |  |  |  |  |
| *Staphylococcus pettenkoferi* | spet |  |  |  | 4 |  |  |  |  |
| *Staphylococcus cohnii* | scoh |  |  |  | 3 |  |  |  |  |
| *Staphylococcus nepalensis* | snl |  |  |  | 4 |  |  |  |  |
| *Staphylococcus kloosii* | skl |  |  |  | 4 |  |  |  |  |
| *Macrococcus canis* | mcak |  |  |  |  |  |  |  |  |
| *Listeria monocytogenes* EGD-e (serotype 1/2a) | lmo |  |  |  |  |  |  |  |  |
| *Listeria monocytogenes* 08-5578 (serotype 1/2a) | lmn |  |  |  | 1 |  |  |  |  |
| *Listeria monocytogenes* 08-5923 (serotype 1/2a) | lmy |  |  |  | 1 |  |  |  |  |
| *Listeria monocytogenes* 10403S (serotype 1/2a) | lmt |  |  |  | 1 |  |  |  |  |
| *Listeria monocytogenes* SLCC5850 (serotype 1/2a) | lmoc |  |  |  | 1 |  |  |  |  |
| *Listeria monocytogenes* La111 (serotype 1/2a) | lmoe |  |  |  | 1 |  |  |  |  |
| *Listeria monocytogenes* N53-1 (serotype 1/2a) | lmob |  |  |  | 1 |  |  |  |  |
| *Listeria monocytogenes* EGD (serotype 1/2a) | lmod |  |  |  | 1 |  |  |  |  |
| *Listeria monocytogenes* WSLC1001 (serotype 1/2a) | lmow |  |  |  | 1 |  |  |  |  |
| *Listeria monocytogenes* 6179 (serotype 1/2a) | lmoq |  |  |  | 1 |  |  |  |  |
| *Listeria monocytogenes* R479a (serotype 1/2a) | lmr |  |  |  | 1 |  |  |  |  |
| *Listeria monocytogenes* Lm60 (serotype 1/2a) | lmom |  |  |  | 1 |  |  |  |  |
| *Listeria monocytogenes* F2365 (serotype 4b) | lmf |  |  |  | 2 |  |  |  |  |
| *Listeria monocytogenes* CLIP 80459 (serotype 4b) | lmc |  |  |  | 2 |  |  |  |  |
| *Listeria monocytogenes* serotype 4b LL195 | lmog |  |  |  | 2 |  |  |  |  |
| *Listeria monocytogenes* 07PF0776 (serotype 4b) | lmp |  |  |  | 2 |  |  |  |  |
| *Listeria monocytogenes* L312 (serotype 4b) | lmol |  |  |  | 2 |  |  |  |  |
| *Listeria monocytogenes* J1816 (serotype 4b) | lmoj |  |  |  | 2 |  |  |  |  |
| *Listeria monocytogenes* J1-220 (serotype 4b) | lmoz |  |  |  | 1 |  |  |  |  |
| *Listeria monocytogenes* WSLC1042 (serotype 4b) | lmox |  |  |  | 2 |  |  |  |  |
| *Listeria monocytogenes* HCC23 (serotype 4a) | lmh |  |  |  | 1 |  |  |  |  |
| *Listeria monocytogenes* M7 (serotype 4a) | lmq |  |  |  | 1 |  |  |  |  |
| *Listeria monocytogenes* L99 (serotype 4a) | lml |  |  |  | 1 |  |  |  |  |
| *Listeria monocytogenes* FSL R2-561 | lmg |  |  |  | 1 |  |  |  |  |
| *Listeria monocytogenes* Finland 1998 | lms |  |  |  | 1 |  |  |  |  |
| *Listeria monocytogenes* J0161 | lmj |  |  |  | 1 |  |  |  |  |
| *Listeria monocytogenes* SLCC2755 (serotype 1/2b) | lmw |  |  |  | 2 |  |  |  |  |
| *Listeria monocytogenes* SLCC2372 (serotype 1/2c) | lmx |  |  |  | 1 |  |  |  |  |
| *Listeria monocytogenes* serotype 7 SLCC2482 | lmz |  |  |  | 2 |  |  |  |  |
| *Listeria monocytogenes* SLCC2376 (serotype 4c) | lmon |  |  |  | 1 |  |  |  |  |
| *Listeria monocytogenes* SLCC7179 (serotype 3a) | lmos |  |  |  | 1 |  |  |  |  |
| *Listeria monocytogenes* SLCC2378 (serotype 4e) | lmoo |  |  |  | 2 |  |  |  |  |
| *Listeria monocytogenes* SLCC2479 (serotype 3c) | lmoy |  |  |  | 1 |  |  |  |  |
| *Listeria monocytogenes* SLCC2540 (serotype 3b) | lmot |  |  |  | 1 |  |  |  |  |
| *Listeria monocytogenes* ATCC 19117 (serotype 4d) | lmoa |  |  |  | 1 |  |  |  |  |
| *Listeria monocytogenes* NE dc2014 | lmok |  |  |  | 2 |  |  |  |  |
| *Listeria monocytogenes* CFSAN006122 | lmv |  |  |  | 2 |  |  |  |  |
| *Listeria innocua* (serotype 6a) | lin |  |  |  |  |  |  |  |  |
| *Listeria welshimeri* | lwe |  |  |  | 1 |  |  |  |  |
| *Listeria seeligeri* | lsg |  |  |  | 1 |  |  |  |  |
| *Listeria ivanovii* subsp. *ivanovii* PAM 55 | liv |  |  |  | 1 |  |  |  |  |
| *Listeria ivanovii* subsp. *ivanovii* WSLC 3010 | lii |  |  |  | 1 |  |  |  |  |
| *Listeria ivanovii* WSLC3009 | liw |  |  |  | 1 |  |  |  |  |
| *Listeria ivanovii* subsp. *londoniensis* WSLC 30167 | lia |  |  |  | 1 |  |  |  |  |
| *Listeria ivanovii* subsp. *londoniensis* WSLC 30151 | lio |  |  |  | 1 |  |  |  |  |
| *Listeria weihenstephanensis* | lwi |  |  |  |  |  |  |  |  |
| *Brochothrix thermosphacta* | bths |  |  |  | 3 |  |  |  |  |
| *Exiguobacterium sibiricum* | esi |  |  |  | 2 |  |  |  |  |
| *Exiguobacterium antarcticum* | ean |  |  |  | 2 |  |  |  |  |
| *Exiguobacterium* sp. MH3 | exm |  |  |  | 3 |  |  |  |  |
| *Exiguobacterium* sp. U13-1 | exu |  |  |  | 3 |  |  |  |  |
| *Gemella* sp. oral taxon 928 | got |  |  |  | 1 |  |  |  |  |
| *Gemella morbillorum* | gmo |  |  |  | 2 |  |  |  |  |
| *Gemella* sp. ND 6198 | geq |  |  |  | 1 |  |  |  |  |
| *Brevibacillus formosus* | bfm |  |  |  | 17 |  |  |  |  |
| *Paenibacillus* sp. JDR-2 | pjd |  |  |  | 4 |  |  |  |  |
| *Paenibacillus polymyxa* E681 | ppy |  |  |  | 11 |  |  |  |  |
| *Paenibacillus terrae* | pta |  |  |  | 12 |  |  |  |  |
| *Paenibacillus sabinae* | psab |  |  |  | 1 |  |  |  |  |
| *Paenibacillus durus* | pdu |  |  |  | 7 |  |  |  |  |
| *Paenibacillus borealis* | pbd |  |  |  | 5 |  |  |  |  |
| *Paenibacillus graminis* | pgm |  |  |  | 7 |  |  |  |  |
| *Paenibacillus odorifer* | pod |  |  |  | 4 |  |  |  |  |
| *Paenibacillus* sp. FSL P4-0081 | paen |  |  |  | 6 |  |  |  |  |
| *Paenibacillus* sp. FSL R5-0345 | paef |  |  |  | 2 |  |  |  |  |
| *Paenibacillus* sp. FSL R5-0912 | paeq |  |  |  | 5 |  |  |  |  |
| *Paenibacillus stellifer* | pste |  |  |  | 3 |  |  |  |  |
| *Paenibacillus* sp. FSL R7-0273 | paea |  |  |  | 6 |  |  |  |  |
| *Paenibacillus* sp. FSL R7-0331 | paee |  |  |  | 5 |  |  |  |  |
| *Paenibacillus* sp. FSL H7-0357 | paeh |  |  |  | 4 |  |  |  |  |
| *Paenibacillus* sp. FSL H7-0737 | paej |  |  |  | 3 |  |  |  |  |
| *Paenibacillus yonginensis* | pyg |  |  |  | 3 |  |  |  |  |
| *Paenibacillus crassostreae* | pcx |  |  |  | 5 |  |  |  |  |
| *Paenibacillus kribbensis* | pkb |  |  |  | 14 |  |  |  |  |
| *Paenibacillus* sp. IHB B 3084 | paih |  |  |  | 8 |  |  |  |  |
| *Paenibacillus vortex* | pvo |  |  |  |  |  |  |  |  |
| *Cohnella* sp. 18JY8-7 | coh |  |  |  | 4 |  |  |  |  |
| *Kyrpidia tusciae* | bts |  |  |  | 3 |  |  |  |  |
| *Tumebacillus avium* | tum |  |  |  | 17 |  |  |  |  |
| *Tumebacillus algifaecis* | tab |  |  |  | 17 |  |  |  |  |
| *Solibacillus* sp. R5-41 | sob |  |  |  | 4 |  |  |  |  |
| *Planococcus* sp. PAMC 21323 | pln |  |  |  | 2 |  |  |  |  |
| *Planococcus kocurii* | pku |  |  |  | 2 |  |  |  |  |
| *Planococcus versutus* | pll |  |  |  | 2 |  |  |  |  |
| *Planococcus donghaensis* | pdg |  |  |  | 2 |  |  |  |  |
| *Planococcus faecalis* | pfae |  |  |  | 2 |  |  |  |  |
| *Jeotgalibacillus malaysiensis* | jeo |  |  |  | 2 |  |  |  |  |
| *Kurthia* sp. 11kri321 | kur |  |  |  | 2 |  |  |  |  |
| *Sporosarcina psychrophila* | spsy |  |  |  | 3 |  |  |  |  |
| *Sporosarcina* sp. PTS2304 | spos |  |  |  | 4 |  |  |  |  |
| *Paenisporosarcina* sp. K2R23-3 | paek |  |  |  | 1 |  |  |  |  |
| *Novibacillus thermophilus* | ntr |  |  |  | 5 |  |  |  |  |
| *Lactococcus lactis* subsp. *lactis* Il1403 | lla |  |  |  | 5 |  |  |  |  |
| *Lactococcus lactis* subsp. *lactis* KF147 | llk |  |  |  | 5 |  |  |  |  |
| *Lactococcus lactis* subsp. *lactis* CV56 | llt |  |  |  | 7 |  |  |  |  |
| *Lactococcus lactis* subsp. *lactis* IO-1 | lls |  |  |  | 7 |  |  |  |  |
| *Lactococcus lactis* subsp. *lactis* KLDS 4.0325 | lld |  |  |  | 5 |  |  |  |  |
| *Lactococcus lactis* subsp. *lactis* NCDO 2118 | llx |  |  |  | 5 |  |  |  |  |
| *Lactococcus lactis* subsp. *cremoris* SK11 | llc |  |  |  | 3 |  |  |  |  |
| *Lactococcus lactis* subsp. *cremoris* MG1363 | llm |  |  |  | 4 |  |  |  |  |
| *Lactococcus lactis* subsp. *cremoris* A76 | llr |  |  |  | 3 |  |  |  |  |
| *Lactococcus lactis* subsp. *cremoris* NZ9000 | lln |  |  |  | 4 |  |  |  |  |
| *Lactococcus lactis* subsp. *cremoris* UC509.9 | lli |  |  |  | 3 |  |  |  |  |
| *Lactococcus lactis* subsp. *cremoris* KW2 | llw |  |  |  | 4 |  |  |  |  |
| *Lactococcus lactis* AI06 | llj |  |  |  | 6 |  |  |  |  |
| *Lactococcus garvieae* ATCC 49156 | lgr |  |  |  |  |  |  |  |  |
| *Lactococcus garvieae* Lg2 | lgv |  |  |  |  |  |  |  |  |
| *Lactococcus piscium* | lpk |  |  |  | 1 |  |  |  |  |
| *Lactococcus raffinolactis* | lrn |  |  |  | 1 |  |  |  |  |
| *Lactococcus* sp. 1JSPR-7 | lact |  |  |  | 1 |  |  |  |  |
| *Streptococcus pyogenes* M1 GAS (serotype M1) | spy |  |  |  | 3 |  |  |  |  |
| *Streptococcus pyogenes* MGAS5005 (serotype M1) | spz |  |  |  | 3 |  |  |  |  |
| *Streptococcus pyogenes* M1 476 (serotype M1) | spym |  |  |  |  |  |  |  |  |
| *Streptococcus pyogenes* A20 (serotype M1) | spya |  |  |  | 3 |  |  |  |  |
| *Streptococcus pyogenes* MGAS8232 (serotype M18) | spm |  |  |  | 3 |  |  |  |  |
| *Streptococcus pyogenes* MGAS10270 (serotype M2) | sph |  |  |  | 4 |  |  |  |  |
| *Streptococcus pyogenes* MGAS10750 (serotype M4) | spi |  |  |  | 5 |  |  |  |  |
| *Streptococcus pyogenes* MGAS2096 (serotype M12) | spj |  |  |  | 4 |  |  |  |  |
| *Streptococcus pyogenes* MGAS9429 (serotype M12) | spk |  |  |  | 4 |  |  |  |  |
| *Streptococcus pyogenes* Manfredo (serotype M5) | spf |  |  |  |  |  |  |  |  |
| *Streptococcus pyogenes* MGAS6180 (serotype M28) | spb |  |  |  | 4 |  |  |  |  |
| *Streptococcus pyogenes* MGAS15252 (serotype M59) | stg |  |  |  | 3 |  |  |  |  |
| *Streptococcus pyogenes* MGAS1882 (serotype M59) | stx |  |  |  | 3 |  |  |  |  |
| *Streptococcus pyogenes* NZ131 (serotype M49) | soz |  |  |  | 3 |  |  |  |  |
| *Streptococcus pyogenes* Alab49 (serotype M53) | stz |  |  |  | 3 |  |  |  |  |
| *Streptococcus pyogenes* HSC5 (serotype M14) | spyh |  |  |  | 3 |  |  |  |  |
| *Streptococcus pyogenes* STAB901 (serotype M44) | spyo |  |  |  | 3 |  |  |  |  |
| *Streptococcus pneumoniae* TIGR4 (virulent serotype 4) | spn |  |  |  | 6 |  |  |  |  |
| *Streptococcus pneumoniae* D39 (virulent serotype 2) | spd |  |  |  | 5 |  |  |  |  |
| *Streptococcus pneumoniae* R6 (avirulent serotype 2) | spr |  |  |  | 6 |  |  |  |  |
| *Streptococcus pneumoniae* CGSP14 (serotype 14) | spw |  |  |  | 7 |  |  |  |  |
| *Streptococcus pneumoniae* JJA (serotype 14) | sjj |  |  |  | 6 |  |  |  |  |
| *Streptococcus pneumoniae* INV200 (serotype 14) | snv |  |  |  | 7 |  |  |  |  |
| *Streptococcus pneumoniae* G54 (serotype 19F) | spx |  |  |  | 6 |  |  |  |  |
| *Streptococcus pneumoniae* Taiwan19F-14 (serotype 19F) | snt |  |  |  | 5 |  |  |  |  |
| *Streptococcus pneumoniae* ST556 (serotype 19F) | snd |  |  |  | 5 |  |  |  |  |
| *Streptococcus pneumoniae* A026 (serotype 19F) | spnn |  |  |  | 5 |  |  |  |  |
| *Streptococcus pneumoniae* ATCC 700669 (serotype 23F ST81 lineage) | sne |  |  |  |  |  |  |  |  |
| *Streptococcus pneumoniae* Hungary19A 6 (serotype 19A) | spv |  |  |  | 7 |  |  |  |  |
| *Streptococcus pneumoniae* TCH8431/19A (serotype 19A) | snc |  |  |  | 5 |  |  |  |  |
| *Streptococcus pneumoniae* 70585 (serotype 5) | snm |  |  |  | 8 |  |  |  |  |
| *Streptococcus pneumoniae* P1031 (serotype 1) | spp |  |  |  | 5 |  |  |  |  |
| *Streptococcus pneumoniae* INV104 (serotype 1) | sni |  |  |  | 6 |  |  |  |  |
| *Streptococcus pneumoniae* gamPNI0373 (virulent serotype 1) | spng |  |  |  | 5 |  |  |  |  |
| *Streptococcus pneumoniae* 670-6B (serotype 6B) | snb |  |  |  | 5 |  |  |  |  |
| *Streptococcus pneumoniae* AP200 (serotype 11A) | snp |  |  |  | 6 |  |  |  |  |
| *Streptococcus pneumoniae* OXC141 (serotype 3) | snx |  |  |  | 6 |  |  |  |  |
| *Streptococcus pneumoniae* SPNA45 (serotype 3) | snu |  |  |  | 5 |  |  |  |  |
| *Streptococcus pneumoniae* SPN034156 (serotype 3) | spne |  |  |  | 5 |  |  |  |  |
| *Streptococcus pneumoniae* SPN034183 (serotype 3) | spnu |  |  |  | 6 |  |  |  |  |
| *Streptococcus pneumoniae* SPN994038 (serotype 3) | spnm |  |  |  | 6 |  |  |  |  |
| *Streptococcus pneumoniae* SPN994039 (serotype 3) | spno |  |  |  | 6 |  |  |  |  |
| *Streptococcus agalactiae* 2603 (serotype V) | sag |  |  |  | 2 |  |  |  |  |
| *Streptococcus agalactiae* NEM316 (serotype III) | san |  |  |  | 2 |  |  |  |  |
| *Streptococcus agalactiae* A909 (serotype Ia) | sak |  |  |  | 2 |  |  |  |  |
| *Streptococcus agalactiae* GD201008-001 (serotype Ia) | sgc |  |  |  | 2 |  |  |  |  |
| *Streptococcus agalactiae* SA20 | sags |  |  |  | 3 |  |  |  |  |
| *Streptococcus agalactiae* 2-22 (serotype Ib) | sagl |  |  |  | 2 |  |  |  |  |
| *Streptococcus agalactiae* 09mas018883 | sagm |  |  |  | 2 |  |  |  |  |
| *Streptococcus agalactiae* ILRI005 | sagi |  |  |  | 2 |  |  |  |  |
| *Streptococcus agalactiae* ILRI112 | sagr |  |  |  | 2 |  |  |  |  |
| *Streptococcus agalactiae* 138P | sagp |  |  |  | 2 |  |  |  |  |
| *Streptococcus agalactiae* 138spar | sagc |  |  |  | 2 |  |  |  |  |
| *Streptococcus agalactiae* COH1 | sagt |  |  |  | 2 |  |  |  |  |
| *Streptococcus agalactiae* NGBS061 | sage |  |  |  | 2 |  |  |  |  |
| *Streptococcus agalactiae* NGBS572 | sagg |  |  |  | 2 |  |  |  |  |
| *Streptococcus agalactiae* CNCTC 10/84 | sagn |  |  |  | 3 |  |  |  |  |
| *Streptococcus mutans* UA159 (serotype c) | smu |  |  |  | 7 |  |  |  |  |
| *Streptococcus mutans* NN2025 (serotype c) | smc |  |  |  | 8 |  |  |  |  |
| *Streptococcus mutans* GS-5 (serotype c) | smut |  |  |  | 8 |  |  |  |  |
| *Streptococcus mutans* LJ23 (serotype k) | smj |  |  |  | 7 |  |  |  |  |
| *Streptococcus mutans* UA159-FR | smua |  |  |  | 7 |  |  |  |  |
| *Streptococcus thermophilus* CNRZ1066 | stc |  |  |  | 4 |  |  |  |  |
| *Streptococcus thermophilus* LMG 18311 | stl |  |  |  | 5 |  |  |  |  |
| *Streptococcus thermophilus* LMD-9 | ste |  |  |  | 5 |  |  |  |  |
| *Streptococcus thermophilus* ND03 | stn |  |  |  | 5 |  |  |  |  |
| *Streptococcus thermophilus* JIM 8232 | stu |  |  |  | 7 |  |  |  |  |
| *Streptococcus thermophilus* MN-ZLW-002 | stw |  |  |  | 6 |  |  |  |  |
| *Streptococcus thermophilus* ASCC 1275 | sthe |  |  |  | 3 |  |  |  |  |
| *Streptococcus thermophilus* S9 | sths |  |  |  | 3 |  |  |  |  |
| *Streptococcus sanguinis* | ssa |  |  |  | 1 |  |  |  |  |
| *Streptococcus suis* BM407 (serotype 2) | ssb |  |  |  | 3 |  |  |  |  |
| *Streptococcus suis* 05ZYH33 (serotype 2) | ssu |  |  |  | 3 |  |  |  |  |
| *Streptococcus suis* 98HAH33 (serotype 2) | ssv |  |  |  | 2 |  |  |  |  |
| *Streptococcus suis* P1/7 (serotype 2) | ssi |  |  |  | 2 |  |  |  |  |
| *Streptococcus suis* SC84 (serotype 2) | sss |  |  |  | 4 |  |  |  |  |
| *Streptococcus suis* A7 (serotype 2) | ssf |  |  |  | 2 |  |  |  |  |
| *Streptococcus suis* GZ1 (serotype 2) | ssw |  |  |  | 2 |  |  |  |  |
| *Streptococcus suis* S735 (serotype 2) | sup |  |  |  | 2 |  |  |  |  |
| *Streptococcus suis* SC070731 (serotype 2) | ssus |  |  |  | 3 |  |  |  |  |
| *Streptococcus suis* ST3 (serotype 3) | sst |  |  |  | 1 |  |  |  |  |
| *Streptococcus suis* YB51 (serotype 3) | ssuy |  |  |  | 1 |  |  |  |  |
| *Streptococcus suis* D12 (serotype 9) | ssk |  |  |  | 5 |  |  |  |  |
| *Streptococcus suis* D9 (serotype 7) | ssq |  |  |  | 2 |  |  |  |  |
| *Streptococcus suis* JS14 (serotype 14) | sui |  |  |  | 3 |  |  |  |  |
| *Streptococcus suis* SS12 (serotype 1/2) | suo |  |  |  | 2 |  |  |  |  |
| *Streptococcus suis* ST1 (serotype 1) | srp |  |  |  | 1 |  |  |  |  |
| *Streptococcus suis* TL13 (serotype 16) | ssut |  |  |  | 2 |  |  |  |  |
| *Streptococcus suis* T15 | ssui |  |  |  | 1 |  |  |  |  |
| *Streptococcus gordonii* | sgo |  |  |  | 2 |  |  |  |  |
| *Streptococcus equi* subsp. *zooepidemicus* MGCS10565 | sez |  |  |  | 7 |  |  |  |  |
| *Streptococcus equi* subsp. *zooepidemicus* H70 | seq |  |  |  | 10 |  |  |  |  |
| *Streptococcus equi* subsp. *zooepidemicus* ATCC 35246 | sezo |  |  |  | 8 |  |  |  |  |
| *Streptococcus equi* subsp. *zooepidemicus* CY | sequ |  |  |  | 7 |  |  |  |  |
| *Streptococcus equi* subsp. *equi* 4047 | seu |  |  |  | 8 |  |  |  |  |
| *Streptococcus uberis* | sub |  |  |  | 3 |  |  |  |  |
| *Streptococcus dysgalactiae* subsp. *equisimilis* GGS_124 | sds |  |  |  | 4 |  |  |  |  |
| *Streptococcus dysgalactiae* subsp. *equisimilis* ATCC 12394 | sdg |  |  |  | 6 |  |  |  |  |
| *Streptococcus dysgalactiae* subsp. *equisimilis* RE378 | sda |  |  |  | 3 |  |  |  |  |
| *Streptococcus dysgalactiae* subsp. *equisimilis* AC-2713 | sdc |  |  |  | 5 |  |  |  |  |
| *Streptococcus dysgalactiae* subsp. *equisimilis* 167 | sdq |  |  |  | 5 |  |  |  |  |
| *Streptococcus gallolyticus* UCN34 | sga |  |  |  | 2 |  |  |  |  |
| *Streptococcus gallolyticus* subsp. *gallolyticus* ATCC BAA-2069 | sgg |  |  |  | 4 |  |  |  |  |
| *Streptococcus gallolyticus* subsp. *gallolyticus* ATCC 43143 | sgt |  |  |  | 2 |  |  |  |  |
| *Streptococcus mitis* | smb |  |  |  | 2 |  |  |  |  |
| *Streptococcus oralis* | sor |  |  |  | 3 |  |  |  |  |
| *Streptococcus parauberis* | stk |  |  |  | 2 |  |  |  |  |
| *Streptococcus pasteurianus* | stb |  |  |  | 2 |  |  |  |  |
| *Streptococcus parasanguinis* ATCC 15912 | scp |  |  |  | 2 |  |  |  |  |
| *Streptococcus parasanguinis* FW213 | scf |  |  |  | 2 |  |  |  |  |
| *Streptococcus salivarius* CCHSS3 | ssr |  |  |  | 5 |  |  |  |  |
| *Streptococcus salivarius* 57.I | stf |  |  |  | 3 |  |  |  |  |
| *Streptococcus salivarius* JIM8777 | stj |  |  |  | 3 |  |  |  |  |
| *Streptococcus salivarius* NCTC 8618 | strs |  |  |  | 4 |  |  |  |  |
| *Streptococcus salivarius* HSISS4 | ssah |  |  |  | 4 |  |  |  |  |
| *Streptococcus pseudopneumoniae* | std |  |  |  | 6 |  |  |  |  |
| *Streptococcus macedonicus* | smn |  |  |  |  |  |  |  |  |
| *Streptococcus infantarius* | sif |  |  |  | 3 |  |  |  |  |
| *Streptococcus intermedius* JTH08 | sie |  |  |  | 5 |  |  |  |  |
| *Streptococcus intermedius* B196 | sib |  |  |  | 6 |  |  |  |  |
| *Streptococcus intermedius* C270 | siu |  |  |  | 3 |  |  |  |  |
| *Streptococcus anginosus* C1051 | sang |  |  |  | 2 |  |  |  |  |
| *Streptococcus anginosus* C238 | sanc |  |  |  | 4 |  |  |  |  |
| *Streptococcus anginosus* SA1 | sans |  |  |  | 3 |  |  |  |  |
| *Streptococcus constellatus* subsp. *pharyngis* C1050 | scg |  |  |  | 5 |  |  |  |  |
| *Streptococcus constellatus* subsp. *pharyngis* C232 | scon |  |  |  | 5 |  |  |  |  |
| *Streptococcus constellatus* subsp. *pharyngis* C818 | scos |  |  |  | 5 |  |  |  |  |
| *Streptococcus cristatus* | soi |  |  |  | 1 |  |  |  |  |
| *Streptococcus iniae* SF1 | sik |  |  |  | 3 |  |  |  |  |
| *Streptococcus iniae* ISET0901 | siq |  |  |  | 2 |  |  |  |  |
| *Streptococcus iniae* ISNO | sio |  |  |  | 2 |  |  |  |  |
| *Streptococcus iniae* SFST01-82 | siz |  |  |  | 3 |  |  |  |  |
| *Streptococcus lutetiensis* | slu |  |  |  | 2 |  |  |  |  |
| *Streptococcus* sp. I-G2 | sig |  |  |  | 3 |  |  |  |  |
| *Streptococcus* sp. I-P16 | sip |  |  |  | 3 |  |  |  |  |
| *Streptococcus* sp. VT 162 | stv |  |  |  | 2 |  |  |  |  |
| *Streptococcus pantholopis* | spat |  |  |  | 4 |  |  |  |  |
| *Streptococcus* sp. A12 | stra |  |  |  | 2 |  |  |  |  |
| *Streptococcus* sp. NPS 308 | strn |  |  |  | 2 |  |  |  |  |
| *Streptococcus sobrinus* | ssob |  |  |  | 6 |  |  |  |  |
| *Lactobacillus plantarum* WCFS1 | lpl |  |  |  | 4 |  |  |  |  |
| *Lactobacillus plantarum* JDM1 | lpj |  |  |  | 3 |  |  |  |  |
| *Lactobacillus plantarum* ZJ316 | lpt |  |  |  | 3 |  |  |  |  |
| *Lactobacillus plantarum* subsp. *plantarum* ST-III | lps |  |  |  | 3 |  |  |  |  |
| *Lactobacillus plantarum* subsp. *plantarum* P-8 | lpr |  |  |  | 4 |  |  |  |  |
| *Lactobacillus plantarum* 16 | lpz |  |  |  | 4 |  |  |  |  |
| *Lactobacillus plantarum* B21 | lpb |  |  |  |  |  |  |  |  |
| *Lactobacillus johnsonii* NCC 533 | ljo |  |  |  | 1 |  |  |  |  |
| *Lactobacillus johnsonii* FI9785 | ljf |  |  |  |  |  |  |  |  |
| *Lactobacillus johnsonii* DPC 6026 | ljh |  |  |  | 2 |  |  |  |  |
| *Lactobacillus johnsonii* N6.2 | ljn |  |  |  | 2 |  |  |  |  |
| *Lactobacillus acidophilus* NCFM | lac |  |  |  | 1 |  |  |  |  |
| *Lactobacillus acidophilus* 30SC | lai |  |  |  | 1 |  |  |  |  |
| *Lactobacillus acidophilus* La-14 | lad |  |  |  |  |  |  |  |  |
| *Lactobacillus acidophilus* FSI4 | laf |  |  |  | 1 |  |  |  |  |
| *Lactobacillus salivarius* UCC118 | lsl |  |  |  | 1 |  |  |  |  |
| *Lactobacillus salivarius* CECT 5713 | lsi |  |  |  | 1 |  |  |  |  |
| *Lactobacillus salivarius* JCM1046 | lsj |  |  |  | 1 |  |  |  |  |
| *Lactobacillus delbrueckii* subsp. *bulgaricus* ATCC 11842 | ldb |  |  |  | 1 |  |  |  |  |
| *Lactobacillus delbrueckii* subsp. *bulgaricus* ATCC BAA-365 | lbu |  |  |  | 1 |  |  |  |  |
| *Lactobacillus delbrueckii* subsp. *bulgaricus* ND02 | lde |  |  |  |  |  |  |  |  |
| *Lactobacillus delbrueckii* subsp. *bulgaricus* 2038 | ldl |  |  |  |  |  |  |  |  |
| *Lactobacillus brevis* ATCC 367 | lbr |  |  |  | 2 |  |  |  |  |
| *Lactobacillus brevis* KB290 | lbk |  |  |  | 2 |  |  |  |  |
| *Lactobacillus paracasei* ATCC 334 | lca |  |  |  | 2 |  |  |  |  |
| *Lactobacillus paracasei* Zhang | lcz |  |  |  | 1 |  |  |  |  |
| *Lactobacillus paracasei* N1115 | lpq |  |  |  | 2 |  |  |  |  |
| *Lactobacillus paracasei* subsp. *paracasei* 8700:2 | lpi |  |  |  | 2 |  |  |  |  |
| *Lactobacillus paracasei* subsp. *paracasei* JCM 8130 | lpap |  |  |  | 1 |  |  |  |  |
| *Lactobacillus casei* BL23 | lcb |  |  |  | 1 |  |  |  |  |
| *Lactobacillus casei* BD-II | lcs |  |  |  | 1 |  |  |  |  |
| *Lactobacillus casei* LC2W | lce |  |  |  | 1 |  |  |  |  |
| *Lactobacillus casei* W56 | lcw |  |  |  | 1 |  |  |  |  |
| *Lactobacillus casei* LOCK919 | lcl |  |  |  | 1 |  |  |  |  |
| *Lactobacillus casei* 12A | lcx |  |  |  | 2 |  |  |  |  |
| *Lactobacillus gasseri* ATCC 33323 | lga |  |  |  |  |  |  |  |  |
| *Lactobacillus reuteri* DSM 20016 | lre |  |  |  | 1 |  |  |  |  |
| *Lactobacillus reuteri* JCM 1112 | lrf |  |  |  | 1 |  |  |  |  |
| *Lactobacillus reuteri* SD2112 | lru |  |  |  | 1 |  |  |  |  |
| *Lactobacillus reuteri* I5007 | lrt |  |  |  | 1 |  |  |  |  |
| *Lactobacillus reuteri* TD1 | lrr |  |  |  | 1 |  |  |  |  |
| *Lactobacillus helveticus* DPC 4571 | lhe |  |  |  | 1 |  |  |  |  |
| *Lactobacillus helveticus* H10 | lhl |  |  |  |  |  |  |  |  |
| *Lactobacillus helveticus* R0052 | lhr |  |  |  | 2 |  |  |  |  |
| *Lactobacillus helveticus* CNRZ32 | lhv |  |  |  |  |  |  |  |  |
| *Lactobacillus helveticus* H9 | lhh |  |  |  |  |  |  |  |  |
| *Lactobacillus helveticus* KLDS1.8701 | lhd |  |  |  |  |  |  |  |  |
| *Lactobacillus fermentum* IFO 3956 | lfe |  |  |  | 1 |  |  |  |  |
| *Lactobacillus fermentum* CECT 5716 | lfr |  |  |  |  |  |  |  |  |
| *Lactobacillus fermentum* F-6 | lff |  |  |  |  |  |  |  |  |
| *Lactobacillus rhamnosus* GG | lrh |  |  |  | 2 |  |  |  |  |
| *Lactobacillus rhamnosus* GG | lrg |  |  |  | 2 |  |  |  |  |
| *Lactobacillus rhamnosus* Lc 705 | lrl |  |  |  | 2 |  |  |  |  |
| *Lactobacillus rhamnosus* ATCC 8530 | lra |  |  |  | 2 |  |  |  |  |
| *Lactobacillus rhamnosus* LOCK900 | lro |  |  |  | 1 |  |  |  |  |
| *Lactobacillus rhamnosus* LOCK908 | lrc |  |  |  | 2 |  |  |  |  |
| *Lactobacillus crispatus* | lcr |  |  |  | 1 |  |  |  |  |
| *Lactobacillus amylovorus* GRL 1112 | lam |  |  |  |  |  |  |  |  |
| *Lactobacillus amylovorus* GRL1118 | lay |  |  |  |  |  |  |  |  |
| *Lactobacillus buchneri* NRRL B-30929 | lbh |  |  |  | 2 |  |  |  |  |
| *Lactobacillus buchneri* CD034 | lbn |  |  |  | 1 |  |  |  |  |
| *Lactobacillus kefiranofaciens* | lke |  |  |  | 2 |  |  |  |  |
| *Lactobacillus ruminis* | lrm |  |  |  | 2 |  |  |  |  |
| *Lactobacillus sanfranciscensis* | lsn |  |  |  | 1 |  |  |  |  |
| *Lactobacillus* sp. wkB8 | law |  |  |  | 2 |  |  |  |  |
| *Lactobacillus hokkaidonensis* | lho |  |  |  | 2 |  |  |  |  |
| *Lactobacillus mucosae* | lmu |  |  |  |  |  |  |  |  |
| *Lactobacillus acetotolerans* | lae |  |  |  |  |  |  |  |  |
| *Lactobacillus ginsenosidimutans* | lgn |  |  |  | 1 |  |  |  |  |
| *Lactobacillus koreensis* | lko |  |  |  | 1 |  |  |  |  |
| *Lactobacillus heilongjiangensis* | lhi |  |  |  |  |  |  |  |  |
| *Lactobacillus kunkeei* | lku |  |  |  | 1 |  |  |  |  |
| *Lactobacillus gallinarum* | lgl |  |  |  |  |  |  |  |  |
| *Lactobacillus paraplantarum* | lpx |  |  |  | 2 |  |  |  |  |
| *Lactobacillus oris* | lor |  |  |  | 1 |  |  |  |  |
| *Lactobacillus parabuchneri* | lpar |  |  |  | 2 |  |  |  |  |
| *Lactobacillus lindneri* | lle |  |  |  | 3 |  |  |  |  |
| *Lactobacillus paracollinoides* | lpd |  |  |  | 2 |  |  |  |  |
| *Lactobacillus jensenii* | lje |  |  |  | 1 |  |  |  |  |
| *Lactobacillus curieae* | lcu |  |  |  | 1 |  |  |  |  |
| *Lactobacillus crustorum* | lct |  |  |  | 1 |  |  |  |  |
| *Lactobacillus backii* | lbt |  |  |  | 1 |  |  |  |  |
| *Lactobacillus curvatus* | lcv |  |  |  |  |  |  |  |  |
| *Lactobacillus amylophilus* | lah |  |  |  | 1 |  |  |  |  |
| *Lactobacillus amylolyticus* | lamy |  |  |  |  |  |  |  |  |
| *Lactobacillus agilis* | lagl |  |  |  | 1 |  |  |  |  |
| *Lactobacillus zymae* | lzy |  |  |  | 1 |  |  |  |  |
| *Lactobacillus pentosus* | lpg |  |  |  | 2 |  |  |  |  |
| *Lactobacillus coryniformis* | lcy |  |  |  |  |  |  |  |  |
| *Lactobacillus acidipiscis* | laca |  |  |  |  |  |  |  |  |
| *Lactobacillus allii* | lalw |  |  |  | 2 |  |  |  |  |
| *Lactobacillus alimentarius* | lali |  |  |  |  |  |  |  |  |
| *Pediococcus pentosaceus* ATCC 25745 | ppe |  |  |  | 2 |  |  |  |  |
| *Pediococcus pentosaceus* SL4 | ppen |  |  |  | 1 |  |  |  |  |
| *Pediococcus claussenii* | pce |  |  |  | 1 |  |  |  |  |
| *Pediococcus damnosus* | pdm |  |  |  | 1 |  |  |  |  |
| *Pediococcus acidilactici* | paci |  |  |  |  |  |  |  |  |
| *Pediococcus inopinatus* | pio |  |  |  | 1 |  |  |  |  |
| *Enterococcus faecalis* V583 | efa |  |  |  | 2 |  |  |  |  |
| *Enterococcus faecalis* 62 | efl |  |  |  |  |  |  |  |  |
| *Enterococcus faecalis* OG1RF | efi |  |  |  |  |  |  |  |  |
| *Enterococcus faecalis* D32 | efd |  |  |  |  |  |  |  |  |
| *Enterococcus faecalis* Symbioflor 1 | efs |  |  |  |  |  |  |  |  |
| *Enterococcus faecalis* DENG1 | efn |  |  |  | 1 |  |  |  |  |
| *Enterococcus faecalis* ATCC 29212 | efq |  |  |  | 1 |  |  |  |  |
| *Enterococcus faecalis* 7L76 | ene |  |  |  | 2 |  |  |  |  |
| *Enterococcus casseliflavus* | ecas |  |  |  | 2 |  |  |  |  |
| *Enterococcus durans* | edu |  |  |  | 3 |  |  |  |  |
| *Enterococcus gallinarum* | ega |  |  |  | 2 |  |  |  |  |
| *Enterococcus silesiacus* | ess |  |  |  | 5 |  |  |  |  |
| *Enterococcus thailandicus* | eth |  |  |  | 3 |  |  |  |  |
| *Melissococcus plutonius* ATCC 35311 | mps |  |  |  |  |  |  |  |  |
| *Melissococcus plutonius* DAT561 | mpx |  |  |  |  |  |  |  |  |
| *Vagococcus teuberi* | vte |  |  |  |  |  |  |  |  |
| *Vagococcus penaei* | vpi |  |  |  | 2 |  |  |  |  |
| *Oenococcus oeni* | ooe |  |  |  | 2 |  |  |  |  |
| *Oenococcus* sp. UCMA 16435 | oen |  |  |  | 1 |  |  |  |  |
| *Oenococcus sicerae* | osi |  |  |  | 1 |  |  |  |  |
| *Leuconostoc mesenteroides* subsp. *mesenteroides* ATCC 8293 | lme |  |  |  | 4 |  |  |  |  |
| *Leuconostoc mesenteroides* subsp. *mesenteroides* J18 | lmm |  |  |  | 3 |  |  |  |  |
| *Leuconostoc mesenteroides* KFRI-MG | lmk |  |  |  | 3 |  |  |  |  |
| *Leuconostoc citreum* | lci |  |  |  | 2 |  |  |  |  |
| *Leuconostoc kimchii* | lki |  |  |  | 3 |  |  |  |  |
| *Leuconostoc* sp. C2 | lec |  |  |  | 3 |  |  |  |  |
| *Leuconostoc carnosum* | lcn |  |  |  | 1 |  |  |  |  |
| *Leuconostoc gelidum* subsp. *gasicomitatum* | lgs |  |  |  | 3 |  |  |  |  |
| *Leuconostoc gelidum* JB7 | lge |  |  |  | 3 |  |  |  |  |
| *Leuconostoc lactis* | llf |  |  |  | 2 |  |  |  |  |
| *Leuconostoc garlicum* | lgc |  |  |  | 3 |  |  |  |  |
| *Leuconostoc mesenteroides* subsp. *suionicum* | lsu |  |  |  | 3 |  |  |  |  |
| *Weissella koreensis* | wko |  |  |  | 1 |  |  |  |  |
| *Weissella ceti* WS08 | wce |  |  |  | 1 |  |  |  |  |
| *Weissella ceti* WS74 | wct |  |  |  | 1 |  |  |  |  |
| *Weissella ceti* WS105 | wci |  |  |  | 1 |  |  |  |  |
| *Weissella cibaria* | wcb |  |  |  | 1 |  |  |  |  |
| *Weissella paramesenteroides* | wpa |  |  |  | 2 |  |  |  |  |
| *Aerococcus urinae* ACS-120-V-Col10a | aur |  |  |  | 1 |  |  |  |  |
| *Aerococcus urinae* CCUG36881 | aun |  |  |  | 1 |  |  |  |  |
| *Aerococcus urinaeequi* | aui |  |  |  | 1 |  |  |  |  |
| *Aerococcus sanguinicola* | asan |  |  |  |  |  |  |  |  |
| *Aerococcus christensenii* | acg |  |  |  |  |  |  |  |  |
| *Aerococcus viridans* | avs |  |  |  |  |  |  |  |  |
| *Aerococcus urinaehominis* | auh |  |  |  | 2 |  |  |  |  |
| *Aerococcaceae bacterium* ZY16052 | abae |  |  |  | 2 |  |  |  |  |
| *Carnobacterium* sp. 17-4 | crn |  |  |  | 2 |  |  |  |  |
| *Carnobacterium maltaromaticum* | cml |  |  |  | 2 |  |  |  |  |
| *Carnobacterium inhibens* | caw |  |  |  | 1 |  |  |  |  |
| *Carnobacterium divergens* | cdj |  |  |  | 2 |  |  |  |  |
| *Jeotgalibaca* sp. PTS2502 | jep |  |  |  | 2 |  |  |  |  |
| *Clostridium perfringens* 13 | cpe |  |  |  |  |  |  |  |  |
| *Clostridium perfringens* ATCC 13124 | cpf |  |  |  | 1 |  |  |  |  |
| *Clostridium perfringens* SM101 | cpr |  |  |  | 2 |  |  |  |  |
| *Clostridium tetani* E88 | ctc |  |  |  |  |  |  |  |  |
| *Clostridium tetani* 12124569 | ctet |  |  |  | 1 |  |  |  |  |
| *Clostridium novyi* | cno |  |  |  | 1 |  |  |  |  |
| *Clostridium botulinum* B Eklund 17B (NRP) | cbk |  |  |  | 1 |  |  |  |  |
| *Clostridium botulinum* BKT015925 | cbn |  |  |  | 1 |  |  |  |  |
| *Clostridium botulinum* E3 | cbt |  |  |  | 1 |  |  |  |  |
| *Clostridium beijerinckii* NCIMB 8052 | cbe |  |  |  | 2 |  |  |  |  |
| *Clostridium beijerinckii* ATCC 35702 | cbz |  |  |  | 2 |  |  |  |  |
| *Clostridium beijerinckii* NCIMB 14988 | cbei |  |  |  | 3 |  |  |  |  |
| *Clostridium kluyveri* DSM 555 | ckl |  |  |  | 4 |  |  |  |  |
| *Clostridium kluyveri* NBRC 12016 | ckr |  |  |  | 3 |  |  |  |  |
| *Clostridium ljungdahlii* | clj |  |  |  | 1 |  |  |  |  |
| *Clostridium cellulovorans* | ccb |  |  |  | 17 |  |  |  |  |
| *Clostridium* sp. SY8519 | cls |  |  |  | 2 |  |  |  |  |
| *Clostridium* sp. BNL1100 | clb |  |  |  | 13 |  |  |  |  |
| *Clostridium saccharoperbutylacetonicum* | csr |  |  |  | 8 |  |  |  |  |
| *Clostridium saccharobutylicum* | csb |  |  |  | 9 |  |  |  |  |
| *Clostridium autoethanogenum* | cah |  |  |  | 1 |  |  |  |  |
| *Clostridium baratii* | cbv |  |  |  | 1 |  |  |  |  |
| *Clostridium scatologenes* | csq |  |  |  | 3 |  |  |  |  |
| *Clostridium aceticum* | cace |  |  |  | 1 |  |  |  |  |
| *Clostridium carboxidivorans* | cck |  |  |  | 2 |  |  |  |  |
| *Clostridium butyricum* | cbut |  |  |  | 1 |  |  |  |  |
| *Clostridium tyrobutyricum* | ctyk |  |  |  | 2 |  |  |  |  |
| *Clostridium estertheticum* | ceu |  |  |  | 4 |  |  |  |  |
| *Clostridium taeniosporum* | ctae |  |  |  | 1 |  |  |  |  |
| *Clostridium formicaceticum* | cfm |  |  |  | 2 |  |  |  |  |
| *Clostridium chauvoei* | cchv |  |  |  | 1 |  |  |  |  |
| *Clostridium argentinense* | carg |  |  |  | 4 |  |  |  |  |
| *Clostridium drakei* | cdrk |  |  |  | 3 |  |  |  |  |
| *Clostridium isatidis* | cia |  |  |  | 2 |  |  |  |  |
| *Alkaliphilus metalliredigens* | amt |  |  |  | 7 |  |  |  |  |
| *Alkaliphilus oremlandii* | aoe |  |  |  | 1 |  |  |  |  |
| *Candidatus Arthromitus* sp. SFB-mouse-Japan | asf |  |  |  | 1 |  |  |  |  |
| *Candidatus Arthromitus* sp. SFB-mouse-Yit | asm |  |  |  | 1 |  |  |  |  |
| *Candidatus Arthromitus* sp. SFB-mouse-NL | aso |  |  |  | 1 |  |  |  |  |
| *Candidatus Arthromitus* sp. SFB-rat-Yit | asb |  |  |  | 1 |  |  |  |  |
| *Geosporobacter ferrireducens* | gfe |  |  |  | 3 |  |  |  |  |
| *Mageeibacillus indolicus* | clo |  |  |  |  |  |  |  |  |
| *Fastidiosipila sanguinis* | fsa |  |  |  |  |  |  |  |  |
| *Hungateiclostridium thermocellum* ATCC 27405 | cth |  |  |  | 5 |  |  |  |  |
| *Hungateiclostridium thermocellum DSM 1313* | ctx |  |  |  | 5 |  |  |  |  |
| *Hungateiclostridium clariflavum* | ccl |  |  |  | 5 |  |  |  |  |
| *Hungateiclostridium saccincola* | hsc |  |  |  | 2 |  |  |  |  |
| *Hungateiclostridiaceae bacterium* KB18 | ruk |  |  |  | 1 |  |  |  |  |
| *Ruminiclostridium cellulolyticum* | cce |  |  |  | 9 |  |  |  |  |
| *Thermoclostridium stercorarium* subsp. *stercorarium* DSM 8532 | css |  |  |  | 2 |  |  |  |  |
| *Thermoclostridium stercorarium* subsp. *stercorarium* DSM 8532 | csd |  |  |  | 2 |  |  |  |  |
| *Pseudoclostridium thermosuccinogenes* | cthd |  |  |  | 5 |  |  |  |  |
| *Eubacterium siraeum* V10Sc8a | esr |  |  |  | 1 |  |  |  |  |
| *Eubacterium siraeum* 70/3 | esu |  |  |  | 1 |  |  |  |  |
| *Clostridium cellulosi* | ccel |  |  |  | 6 |  |  |  |  |
| *Flavonifractor plautii* | fpla |  |  |  | 1 |  |  |  |  |
| *Ethanoligenens harbinense* | eha |  |  |  | 2 |  |  |  |  |
| *Ruminococcus albus* | ral |  |  |  | 2 |  |  |  |  |
| *Ruminococcus champanellensis* | rch |  |  |  | 2 |  |  |  |  |
| *Ruminococcus* sp. SR1/5 | rum |  |  |  | 1 |  |  |  |  |
| *Ruminococcus bicirculans* | rus |  |  |  | 1 |  |  |  |  |
| *Faecalibacterium prausnitzii* L2-6 | fpr |  |  |  | 2 |  |  |  |  |
| *Faecalibacterium prausnitzii* SL3/3 | fpa |  |  |  | 1 |  |  |  |  |
| *Caproiciproducens* sp. NJN-50 | capr |  |  |  | 2 |  |  |  |  |
| *Butyrivibrio proteoclasticus* | bpb |  |  |  | 4 |  |  |  |  |
| *Butyrivibrio fibrisolvens* | bfi |  |  |  | 2 |  |  |  |  |
| *Butyrivibrio hungatei* | bhu |  |  |  | 1 |  |  |  |  |
| *Roseburia hominis* | rho |  |  |  | 1 |  |  |  |  |
| *Roseburia intestinalis* XB6B4 | rix |  |  |  | 1 |  |  |  |  |
| *Roseburia intestinalis* M50/1 | rim |  |  |  | 1 |  |  |  |  |
| *Coprococcus* sp. ART55/1 | coo |  |  |  | 2 |  |  |  |  |
| *Coprococcus catus* | cct |  |  |  | 2 |  |  |  |  |
| *Blautia obeum* | rob |  |  |  | 2 |  |  |  |  |
| *Ruminococcus torques* | rto |  |  |  | 4 |  |  |  |  |
| *Blautia hansenii* | bhan |  |  |  | 2 |  |  |  |  |
| *Blautia* sp. N6H1-15 | blau |  |  |  | 2 |  |  |  |  |
| *Lachnoclostridium* sp. YL32 | lacy |  |  |  | 6 |  |  |  |  |
| *Clostridium cf. saccharolyticum* K10 | cso |  |  |  | 1 |  |  |  |  |
| *Clostridium bolteae* | cbol |  |  |  | 3 |  |  |  |  |
| *Anaerostipes hadrus* | bprl |  |  |  | 1 |  |  |  |  |
| *Anaerotignum propionicum* | cpro |  |  |  | 1 |  |  |  |  |
| *Lachnoanaerobaculum umeaense* | lua |  |  |  | 1 |  |  |  |  |
| *Eubacterium rectale* ATCC 33656 | ere |  |  |  | 3 |  |  |  |  |
| *Eubacterium rectale* DSM 17629 | ert |  |  |  | 3 |  |  |  |  |
| *Eubacterium rectale* M104/1 | era |  |  |  | 1 |  |  |  |  |
| *Lachnospiraceae bacterium* oral taxon 500 | lbw |  |  |  | 1 |  |  |  |  |
| *Clostridioides difficile* 630 | cdf |  |  |  | 2 |  |  |  |  |
| *Clostridioides difficile* 630 | pdc |  |  |  | 2 |  |  |  |  |
| *Clostridioides difficile* CD196 | cdc |  |  |  | 1 |  |  |  |  |
| *Clostridioides difficile* R20291 | cdl |  |  |  | 1 |  |  |  |  |
| *Clostridioides difficile* 630Derm | pdf |  |  |  |  |  |  |  |  |
| *Peptoclostridium acidaminophilum* | eac |  |  |  | 2 |  |  |  |  |
| *Acetoanaerobium sticklandii* | cst |  |  |  | 2 |  |  |  |  |
| *Paeniclostridium sordellii* | psor |  |  |  | 1 |  |  |  |  |
| *Peptostreptococcaceae bacterium* oral taxon 929 | pbq |  |  |  | 1 |  |  |  |  |
| *Symbiobacterium thermophilum* | sth |  |  |  | 1 |  |  |  |  |
| *Syntrophomonas wolfei* | swo |  |  |  | 2 |  |  |  |  |
| *Syntrophothermus lipocalidus* | slp |  |  |  | 1 |  |  |  |  |
| *Desulfitobacterium hafniense* Y51 | dsy |  |  |  | 3 |  |  |  |  |
| *Desulfitobacterium hafniense* DCB-2 | dhd |  |  |  | 3 |  |  |  |  |
| *Desulfitobacterium dehalogenans* | ddh |  |  |  | 1 |  |  |  |  |
| *Desulfitobacterium dichloroeliminans* | ddl |  |  |  | 2 |  |  |  |  |
| *Desulfitobacterium metallireducens* | dmt |  |  |  | 1 |  |  |  |  |
| *Desulfotomaculum reducens* | drm |  |  |  | 1 |  |  |  |  |
| *Desulfotomaculum nigrificans* | dca |  |  |  |  |  |  |  |  |
| *Desulfotomaculum ruminis* | dru |  |  |  | 1 |  |  |  |  |
| *Desulfotomaculum ferrireducens* | dfg |  |  |  | 1 |  |  |  |  |
| *Desulfofarcimen acetoxidans* | dae |  |  |  | 6 |  |  |  |  |
| *Desulfofundulus kuznetsovii* | dku |  |  |  | 1 |  |  |  |  |
| *Desulfallas gibsoniae* | dgi |  |  |  | 6 |  |  |  |  |
| *Pelotomaculum thermopropionicum* | pth |  |  |  | 2 |  |  |  |  |
| *Candidatus Desulforudis audaxviator* | dau |  |  |  | 1 |  |  |  |  |
| *Thermincola potens* | tjr |  |  |  | 2 |  |  |  |  |
| *Syntrophobotulus glycolicus* | sgy |  |  |  | 4 |  |  |  |  |
| *Desulfosporosinus orientis* | dor |  |  |  | 6 |  |  |  |  |
| *Desulfosporosinus acidiphilus* | dai |  |  |  | 1 |  |  |  |  |
| *Desulfosporosinus meridiei* | dmi |  |  |  | 3 |  |  |  |  |
| *Dehalobacter* sp. DCA | ded |  |  |  | 1 |  |  |  |  |
| *Dehalobacter* sp. CF | dec |  |  |  | 1 |  |  |  |  |
| *Dehalobacter restrictus* | drs |  |  |  | 2 |  |  |  |  |
| *Heliobacterium modesticaldum* | hmo |  |  |  | 2 |  |  |  |  |
| *Eubacterium eligens* | eel |  |  |  | 2 |  |  |  |  |
| *Eubacterium limosum* | elm |  |  |  | 6 |  |  |  |  |
| *Eubacterium hallii* | ehl |  |  |  | 3 |  |  |  |  |
| *Acetobacterium woodii* | awo |  |  |  | 4 |  |  |  |  |
| *Oscillibacter valericigenes* | ova |  |  |  | 1 |  |  |  |  |
| *Thermaerobacter marianensis* | tmr |  |  |  |  |  |  |  |  |
| *Carboxydocella thermautotrophica* | cthm |  |  |  | 2 |  |  |  |  |
| *Christensenella minuta* | cmiu |  |  |  | 2 |  |  |  |  |
| *Intestinimonas butyriciproducens* | ibu |  |  |  | 1 |  |  |  |  |
| *Mogibacterium diversum* | mdv |  |  |  | 2 |  |  |  |  |
| *Aminipila* sp. JN-39 | amij |  |  |  | 1 |  |  |  |  |
| *Eubacterium sulci* | euu |  |  |  | 2 |  |  |  |  |
| *Butyrate-producing bacterium* SM4/1 | bprm |  |  |  | 1 |  |  |  |  |
| *Butyrate-producing bacterium* SS3/4 | bprs |  |  |  | 1 |  |  |  |  |
| *Clostridiales bacterium* 70B-A | cbar |  |  |  | 2 |  |  |  |  |
| *Caldanaerobacter subterraneus* subsp. *tengcongensis* | tte |  |  |  |  |  |  |  |  |
| *Thermoanaerobacter* sp. X514 | tex |  |  |  |  |  |  |  |  |
| *Thermoanaerobacter* sp. X513 | thx |  |  |  | 1 |  |  |  |  |
| *Thermoanaerobacter pseudethanolicus* | tpd |  |  |  | 1 |  |  |  |  |
| *Thermoanaerobacter italicus* | tit |  |  |  | 1 |  |  |  |  |
| *Thermoanaerobacter mathranii* | tmt |  |  |  | 2 |  |  |  |  |
| *Thermoanaerobacter brockii* | tbo |  |  |  | 1 |  |  |  |  |
| *Thermoanaerobacter wiegelii* | twi |  |  |  | 1 |  |  |  |  |
| *Thermoanaerobacter kivui* | tki |  |  |  | 3 |  |  |  |  |
| *Carboxydothermus hydrogenoformans* | chy |  |  |  | 1 |  |  |  |  |
| *Tepidanaerobacter acetatoxydans* Re1 | tep |  |  |  | 2 |  |  |  |  |
| *Tepidanaerobacter acetatoxydans* Re1 | tae |  |  |  | 2 |  |  |  |  |
| *Moorella thermoacetica* | mta |  |  |  |  |  |  |  |  |
| *Ammonifex degensii* | adg |  |  |  | 1 |  |  |  |  |
| *Thermacetogenium phaeum* | tpz |  |  |  | 1 |  |  |  |  |
| *Caldicellulosiruptor saccharolyticus* | csc |  |  |  | 3 |  |  |  |  |
| *Caldicellulosiruptor bescii* | ate |  |  |  | 7 |  |  |  |  |
| *Caldicellulosiruptor obsidiansis* | cob |  |  |  | 3 |  |  |  |  |
| *Caldicellulosiruptor hydrothermalis* | chd |  |  |  | 1 |  |  |  |  |
| *Caldicellulosiruptor owensensis* | cow |  |  |  | 1 |  |  |  |  |
| *Caldicellulosiruptor kristjanssonii* | cki |  |  |  | 3 |  |  |  |  |
| *Caldicellulosiruptor kronotskyensis* | ckn |  |  |  | 3 |  |  |  |  |
| *Caldicellulosiruptor lactoaceticus* | clc |  |  |  | 3 |  |  |  |  |
| *Thermosediminibacter oceani* | toc |  |  |  | 1 |  |  |  |  |
| *Thermoanaerobacterium thermosaccharolyticum* DSM 571 | ttm |  |  |  | 1 |  |  |  |  |
| *Thermoanaerobacterium thermosaccharolyticum* M0795 | tto |  |  |  | 2 |  |  |  |  |
| *Thermoanaerobacterium xylanolyticum* | txy |  |  |  | 1 |  |  |  |  |
| *Thermoanaerobacterium saccharolyticum* | tsh |  |  |  |  |  |  |  |  |
| *Thermodesulfobium narugense* | tnr |  |  |  |  |  |  |  |  |
| *Thermodesulfobium acidiphilum* | taci |  |  |  |  |  |  |  |  |
| *Mahella australiensis* | mas |  |  |  | 2 |  |  |  |  |
| *Natranaerobius thermophilus* | nth |  |  |  | 2 |  |  |  |  |
| *Halothermothrix orenii* | hor |  |  |  | 2 |  |  |  |  |
| *Halanaerobium hydrogeniformans* | has |  |  |  | 2 |  |  |  |  |
| *Halanaerobium praevalens* | hpk |  |  |  | 2 |  |  |  |  |
| *Halocella* sp. SP3-1 | hals |  |  |  | 1 |  |  |  |  |
| *Acetohalobium arabaticum* | aar |  |  |  |  |  |  |  |  |
| *Halobacteroides halobius* | hhl |  |  |  | 2 |  |  |  |  |
| *Anoxybacter fermentans* | aft |  |  |  | 1 |  |  |  |  |
| *Finegoldia magna* | fma |  |  |  |  |  |  |  |  |
| *Anaerococcus prevotii* | apr |  |  |  | 1 |  |  |  |  |
| *Parvimonas micra* | pmic |  |  |  |  |  |  |  |  |
| *Peptoniphilus* sp. ING2-D1G | ped |  |  |  | 1 |  |  |  |  |
| *Gottschalkia acidurici* | cad |  |  |  | 1 |  |  |  |  |
| *Sporanaerobacter* sp. NJN-17 | spoa |  |  |  | 4 |  |  |  |  |
| *Veillonella parvula* | vpr |  |  |  | 1 |  |  |  |  |
| *Veillonella atypica* | vat |  |  |  | 1 |  |  |  |  |
| *Veillonella rodentium* | vrm |  |  |  | 3 |  |  |  |  |
| *Megasphaera elsdenii* | med |  |  |  | 1 |  |  |  |  |
| *Megasphaera hexanoica* | mhw |  |  |  |  |  |  |  |  |
| *Megasphaera* sp. AJH120 | meg |  |  |  | 1 |  |  |  |  |
| *Dialister pneumosintes* | dpn |  |  |  |  |  |  |  |  |
| *Selenomonas sputigena* | ssg |  |  |  | 1 |  |  |  |  |
| *Selenomonas ruminantium* | sri |  |  |  | 3 |  |  |  |  |
| *Selenomonas* sp. oral taxon 478 | sele |  |  |  |  |  |  |  |  |
| *Selenomonas* sp. oral taxon 136 | selo |  |  |  |  |  |  |  |  |
| *Selenomonas* sp. oral taxon 920 | selt |  |  |  |  |  |  |  |  |
| *Megamonas hypermegale* | mhg |  |  |  | 1 |  |  |  |  |
| *Pelosinus* sp. UFO1 | puf |  |  |  | 4 |  |  |  |  |
| *Pelosinus fermentans* | pft |  |  |  | 11 |  |  |  |  |
| *Methylomusa anaerophila* | mana |  |  |  | 10 |  |  |  |  |
| *Acidaminococcus fermentans* | afn |  |  |  | 1 |  |  |  |  |
| *Acidaminococcus intestini* | ain |  |  |  | 1 |  |  |  |  |
| *Phascolarctobacterium faecium* | pfac |  |  |  | 2 |  |  |  |  |
| *Erysipelothrix rhusiopathiae* Fujisawa | erh |  |  |  | 1 |  |  |  |  |
| *Erysipelothrix rhusiopathiae* SY1027 | ers |  |  |  | 1 |  |  |  |  |
| *Erysipelothrix larvae* | erl |  |  |  | 1 |  |  |  |  |
| *Erysipelothrix* sp. 15TAL0474 | eri |  |  |  |  |  |  |  |  |
| *Faecalitalea cylindroides* | euc |  |  |  |  |  |  |  |  |
| *Turicibacter* sp. H121 | tur |  |  |  | 1 |  |  |  |  |
| *Faecalibaculum rodentium* | tur |  |  |  |  |  |  |  |  |
| *Erysipelotrichaceae bacterium* I46 | fro |  |  |  |  |  |  |  |  |
| *Erysipelotrichaceae bacterium* SG0102 | erb |  |  |  | 1 |  |  |  |  |
| *Peptoniphilus harei* | ebm |  |  |  | 1 |  |  |  |  |
| *Veillonella dispar* | vdn |  |  |  | 1 |  |  |  |  |

1 Blin, K. *et al.* The antiSMASH database version 2: a comprehensive resource on secondary metabolite biosynthetic gene clusters. *Nucleic acids research* **47**, D625-d630, doi:10.1093/nar/gky1060 (2019).
